# Supplementary material for: Quantifying the influence of mutation detection on tumour subclonal reconstruction
Source: Nat Commun. 2020 Dec 7;11:6247. doi: 10.1038/s41467-020-20055-w (PMC7721877; doi:10.1038/s41467-020-20055-w)
Supplement: Supplementary file 3 — Description of Additional Supplementary Files [file 41467_2020_20055_MOESM3_ESM.pdf]

**Description of Additional Supplementary Files**

**File Name: Supplementary Data 1-20**
**Description: Single-Region Subclonal Reconstruction Data.** Subclonal reconstruction results for 293 tumours with single-region sequencing based on sixteen subclonal reconstruction pipelines and four pipelines using PhyloWGS and the union and intersection of mutation detection tool outputs. Information is displayed in separate Supplementary Data files for all PhyloWGS-comprised pipelines and all pipelines using each of the subclonal reconstruction algorithms PyClone, DPClust and SciClone. Phylogenetic clone tree variables from PhyloWGS-comprising pipelines include tree type, tree lineage type, number of subclones, tumour cellular prevalence, tree root width, tree depth, clonality and Shannon index score for subclonal diversity. Mutational variables include proportion of clonal SNVs, proportion of clonal CNAs, number of SNVs, number of CNAs, overall percent genome altered (PGA) and PGA due to clonal CNAs. Additional information includes the random number generator seed used for PhyloWGS reconstruction, coverage of sequencing sample, cellular prevalence obtained from the CNA detection tool used, clonal and subclonal CNA subtype assignment, biochemical recurrence event and time to event, and multi-modal and unified biomarker group. For all other pipelines, subclonal reconstruction variables include number of subclones, tumour cellularity, cellular prevalence of each subclone, number of SNVs in each cluster, clonality, total number of SNVs and proportion of clonal SNVs.

**File Name: Supplementary Data 21**
**Description: Failed Reconstructions.** Number of reconstructions attempted, number of successful reconstructions and failure rate for all single- and multi-region subclonal reconstruction pipelines. Information is displayed separately for each of the twenty-two different mutation detection tool and subclonal reconstruction algorithm combinations. Summary information is calculated for single-region reconstruction of 293 tumours, single-region reconstruction of 30 samples from 10 tumours with multi-region sequencing, and multi-region reconstructions of the 10 tumours. Each sample that failed reconstruction is listed with the reason of failure.

**File Name: Supplementary Data 22-33**
**Description: CNA Data.** CNA data in 1.0 Mbp genomic bins. Each number represents the copy number of the CNA overlapping the genomic bin. Information is displayed in separate Supplementary Data files for clonal and subclonal CNAs and for each of the six subclonal reconstruction pipelines using PhyloWGS. Information from single-region reconstruction of 293 samples are presented first in each table, followed by single-region reconstruction results of 30 samples from 10 tumours with multi-region sequencing and multi-region reconstructions of the 10 tumours.

**File Name: Supplementary Data 34-37**
**Description: Differentially Altered Genes.** Genes tested for bias towards clonal or subclonal CNAs. All genes are ordered by genomic position and the number of samples affected by clonal and subclonal CNAs are recorded. Pearson's $\chi^2$ test was used to test for bias, with p-values adjusted using FDR. Information is presented in separate Supplementary Data files for each PhyloWGS-comprising pipeline.

**File Name: Supplementary Data 38-49**

**Description: Multi-Region Subclonal Reconstruction Data.** Subclonal reconstruction results for 10 tumours with multi-region sequencing. Results are included for reconstructions of each individual region and results from the multi-region reconstructions. Information is displayed in separate Supplementary Data files for all six pipelines using PhyloWGS and all six pipelines using PyClone. Phylogenetic clone tree variables from PhyloWGS solutions include tree type, tree lineage type, number of subclones, tumour cellular prevalence, tree root width and clonality. Mutational variables include proportion of clonal SNVs, proportion of clonal CNAs, number of SNVs, number of CNAs, overall percent genome altered (PGA) and PGA due to clonal CNAs. Additional information includes the random number generator seed used for PhyloWGS reconstruction. PyClone subclonal architecture variables include number of subclones, tumour cellularity, cellular prevalence of each subclone, number of SNVs in each cluster, total number of SNVs and proportion of SNVs that are clonal.
