# Supplementary material for: Quantifying the influence of mutation detection on tumour subclonal reconstruction
Source: Nat Commun. 2020 Dec 7;11:6247. doi: 10.1038/s41467-020-20055-w (PMC7721877; doi:10.1038/s41467-020-20055-w)
Supplement: Supplementary file 5 — Reporting Summary [file 41467_2020_20055_MOESM5_ESM.pdf]

nature research

Corresponding author(s): Dr. Paul C Boutros
Last updated by author(s): Nov 5, 2020

Reporting Summary

Nature Research wishes to improve the reproducibility of the work that we publish. This form provides structure for consistency and transparency in reporting. For further information on Nature Research policies, see our Editorial Policies and the Editorial Policy Checklist.

Statistics

For all statistical analyses, confirm that the following items are present in the figure legend, table legend, main text, or Methods section.

n/a	Confirmed	
☐	☒	The exact sample size (n) for each experimental group/condition, given as a discrete number and unit of measurement
☐	☒	A statement on whether measurements were taken from distinct samples or whether the same sample was measured repeatedly
☐	☒	The statistical test(s) used AND whether they are one- or two-sided<br>*Only common tests should be described solely by name; describe more complex techniques in the Methods section.*
☐	☒	A description of all covariates tested
☐	☒	A description of any assumptions or corrections, such as tests of normality and adjustment for multiple comparisons
☐	☒	A full description of the statistical parameters including central tendency (e.g. means) or other basic estimates (e.g. regression coefficient) AND variation (e.g. standard deviation) or associated estimates of uncertainty (e.g. confidence intervals)
☐	☒	For null hypothesis testing, the test statistic (e.g. F, t, r) with confidence intervals, effect sizes, degrees of freedom and P value noted<br>*Give P values as exact values whenever suitable.*
☒	☐	For Bayesian analysis, information on the choice of priors and Markov chain Monte Carlo settings
☐	☒	For hierarchical and complex designs, identification of the appropriate level for tests and full reporting of outcomes
☐	☒	Estimates of effect sizes (e.g. Cohen's d, Pearson's r), indicating how they were calculated

Our web collection on statistics for biologists contains articles on many of the points above.

Software and code

Policy information about availability of computer code

Data collection	No software was used to collect data.
Data analysis	Published and open-source software include: R statistical environment (v3.2.5 or v3.5.3), bwa-aln (v0.5.7), picard (v1.92 or v1.107), GATK (v2.4.9), SAMtools (v0.1.9), ContEst (v1.0.24530), Battenberg (v2.2.6), TITAN (v1.11.0), FACETS (v0.5.14), ASCAT (v2.5), cgpBattenberg (v3.1.0), Kronos (v1.12.0), HMMcopy (v0.1.1), bowtie (v2.2.6), MutationSeq (v4.3.7), snp-pileup (v434b5ce), htslib (v1.9), MuTect (v1.1.4), SomaticSniper (v1.0.2), ANNOVAR (v2015-06-17), vcftools (v0.1.15), GenomicRanges (v1.28.6), bedtools (v2.27.1), bedr (v1.0.6), PhyloWGS (v3b75ba9), PyClone (v0.13.0), DPClust (v2.2.5), sciClone (v1.0.7), dpclust3p (v1.0.6), gprofiler2 (v0.1.9), Cytoscape (v3.4.0), lattice (v0.20-34), latticeExtra (v0.6-28), VennDiagram (v1.6.21), BPG (v5.3.4), Inkscape (v0.91). No custom algorithms or software were developed or utilized in this study. Custom data analysis & data visualization code is available upon request.

For manuscripts utilizing custom algorithms or software that are central to the research but not yet described in published literature, software must be made available to editors and reviewers. We strongly encourage code deposition in a community repository (e.g. GitHub). See the Nature Research guidelines for submitting code & software for further information.

Data

Policy information about availability of data

All manuscripts must include a data availability statement. This statement should provide the following information, where applicable:

- Accession codes, unique identifiers, or web links for publicly available datasets
- A list of figures that have associated raw data
- A description of any restrictions on data availability

Published data analyzed in this study, publicly available with appropriate Data Access Compliance Office authorization, include:
WGS Data – Baca et al., 2013: dbGaP, phs000447.v1.p1 [https://www.ncbi.nlm.nih.gov/projects/gap/cgi-bin/study.cgi?study_id=phs000447.v1.p1]

WGS Data – Berger et al., 2011: dbGaP, phs000330.v1.p1 [https://www.ncbi.nlm.nih.gov/projects/gap/cgi-bin/study.cgi?study_id=phs000330.v1.p1]
WGS Data – CPC-GENE Espiritu et al., 2018: EGA, EGAD00001001094 [https://www.ebi.ac.uk/ega/datasets/EGAD00001001094]
WGS Data – CPC-GENE Fraser et al., 2017: EGA, EGAD00001001094 [https://www.ebi.ac.uk/ega/datasets/EGAD00001001094]
WGS Data – CPC-GENE Taylor et al., 2017: EGA, EGAD00001002739 [https://www.ebi.ac.uk/ega/datasets/EGAD00001002739]
WGS Data – The Cancer Genome Atlas Research Network, 2015: https://portal.gdc.cancer.gov/projects/TCGA-PRAD
WGS Data – Weischenfeldt et al., 2013: EGA, EGAS00001000400 [https://www.ebi.ac.uk/ega/studies/EGAS00001000400]
Data supporting the conclusions of this article is included within it and its additional files, and at: ICGC Data Portal under the project PRAD-CA [https://dcc.icgc.org/projects/PRAD-CA], available with appropriate ICGC Data Access Compliance Office approval.
Source data for Figures 5, 7 and Supplementary Figures 3B-E, 4A-B, 5, 6, 8AC, 14 are provided in Source Data.

Field-specific reporting

Please select the one below that is the best fit for your research. If you are not sure, read the appropriate sections before making your selection.

- [x] Life sciences
- [ ] Behavioural & social sciences
- [ ] Ecological, evolutionary & environmental sciences

For a reference copy of the document with all sections, see nature.com/documents/nr-reporting-summary-flat.pdf

Life sciences study design

All studies must disclose on these points even when the disclosure is negative.

Sample size	We evaluated 293 localized prostate tumours with single-region whole genome sequencing (Espiritu et al., Cell 2018) and 10 prostate tumours with multi-region whole-genome sequencing (a total of 30 regions sequenced from the 10 samples; Boutros et al., Nature Genetics 2015). These samples represent publicly available samples of localized prostate cancer with whole-genome sequencing. Previous subclonal reconstruction studies have focused on small cohorts (~10 tumours) and this cohort allows us to evaluate various subclonal reconstruction pipelines on a large cohort of primary tumour samples linked to rich clinical data.
Data exclusions	Samples classified as polytumour were excluded from the subclonality analyses. This was done because subclonal reconstructions of polytumour samples are challenging (please see Espiritu et al., Cell 2018 for more details). This exclusion criteria was established before the analysis was carried out. Tumours designated to have failed reconstructions due to post-processing heuristics were excluded from further analysis. The post-processing heuristics were designed to minimize bias in subclonal reconstruction and provide a lower bound of intra-tumoural heterogeneity (please see Espiritu et al., Cell 2018 for more details), and this exclusion criteria was established before the analysis was carried out. Multi-region reconstructions by pipelines using SciClone were excluded for downstream analysis due to the fact that only 4/40 samples successfully completed reconstruction, and there is little to be gleaned from downstream comparison between single- and multi-region reconstructions. This exclusion criteria was unexpected and was not established before the analysis.
Replication	No replication in additional cohorts were attempted in this study.
Randomization	Samples were never allocated into experimental groups.
Blinding	There was no sample group allocations and no recruitment in this study.

Reporting for specific materials, systems and methods

We require information from authors about some types of materials, experimental systems and methods used in many studies. Here, indicate whether each material, system or method listed is relevant to your study. If you are not sure if a list item applies to your research, read the appropriate section before selecting a response.

Materials & experimental systems

n/a	Involved in the study	
x		Antibodies
x		Eukaryotic cell lines
x		Palaeontology and archaeology
x		Animals and other organisms
	x	Human research participants
x		Clinical data
x		Dual use research of concern

Methods

n/a	Involved in the study	
x		ChIP-seq
x		Flow cytometry
x		MRI-based neuroimaging

Human research participants

Policy information about studies involving human research participants

Population characteristics	Prostate cancer patients ranged in age from 38 – 81 years. All prostate cancer patients were diagnosed with localized disease and were primarily classified as having intermediate risk prostate cancer. All prostate cancer patients were male.
Recruitment	We obtained Informed consent, consistent with the guidelines of the local Research Ethics Board (REB) and International Cancer Genome Consortium at the time of clinical follow-up. Previously collected tumour tissues were used, following University Health Network REB-approved study protocols (UHN 06-0822-CE, UHN 11-0024-CE, CHUQ 2012-913:H12-03-192). The CPC-GENE study focused on patients with localized prostate cancer. All prostate cancer samples were obtained from the University Health Network (UHN) Pathology BioBank or from the Genito-Urinary BioBank of the Centre Hospitalier Universitaire de Québec (CHUQ). Most patients enrolled into CPC-GENE were those with intermediate risk prostate cancer. While the TCGA prostate cancer patients span the different risk categories for prostate cancer, some of the results presented may be biased to reflect the biology of intermediate risk prostate cancer.
Ethics oversight	All tumour samples in this study were obtained with patient informed consent, with approvals by the University Health Network Institutional Research Ethics Board, the Centre Hospitalier Universitaire de Québec Institutional Research Ethics Board and the University of California Los Angeles Institutional Research Ethics Board, and following ICGC guidelines.

Note that full information on the approval of the study protocol must also be provided in the manuscript.
