# Supplementary material for: Quantifying the influence of mutation detection on tumour subclonal reconstruction
Source: Nat Commun. 2020 Dec 7;11:6247. doi: 10.1038/s41467-020-20055-w (PMC7721877; doi:10.1038/s41467-020-20055-w)
Supplement: Supplementary file 2 — Peer Review File [file 41467_2020_20055_MOESM2_ESM.pdf]

Reviewers' comments:

Reviewer #1 (Remarks to the Author): Expertise in phylogenetics and bioinformatics

The authors evaluate six pipelines with respect to their variability and consistency in detecting intra-tumor heterogeneity (ITH). Using predominantly single-sample and some multi-region WGS data of prostrate cancers, the authors find extensive variability among pipelines in determining clonality status of SNVs and CNAs. This is a timely paper, and of interest to the readership of this journal.

1. Define subclonal architecture

The authors refer to PhyloWGS, PyClone and DPClust as algorithms to reconstruct subclonal architecture. While such a broad term enables one to classify these three algorithms in the same group, I do encourage the authors to be explicit in their definition of "subclonal architecture". Based on the performed analyses, it seems that subclonal architecture is defined as clusters of somatic mutations (containing SNVs and/or CNAs) with similar cancer cell fraction. Such as a clustering does not fully resolve evolutionary relationships of mutation clusters. In particular, it only enables the designation of clusters as either subclonal or clonal. To fully resolve evolutionary relationships, a tree inference algorithm is required (e.g. PhyloWGS) as opposed to a mutation clustering algorithm (e.g. DPClust and PyClone). It would be good to clarify this in the paper.

As an additional discussion point, multiple phylogenetic trees may be inferred from the same data (e.g. [1,2]), also when using PhyloWGS (i.e., there are multiple trees with maximum or near-maximum likelihood). I wonder how inclusion of alternative trees affects Figure 6B.

[1] Y. Qi, D. Pradhan and M. El-Kebir. Implications of non-uniqueness in phylogenetic deconvolution of bulk DNA samples of tumors. Algorithms for Molecular Biology, 14:19, 2019. https://doi.org/10.1186/s13015-019-0155-6

[2] Jamal-Hanjani, M., Wilson, G. A., McGranahan, N., Birkbak, N. J., Watkins, T. B. K., Veeriah, S., et al. (2017). Tracking the Evolution of Non-Small-Cell Lung Cancer. New England Journal of Medicine, 376(22), 2109–2121. http://doi.org/10.1056/NEJMoa1616288

2. What are the downstream effects of incorrect clonality determination?

To illustrate the importance of accurate quantification of ITH, it would be good if the authors could demonstrate how variability in clonality status affects the conclusions of the orginal papers. At the very least it would be good to mention in the Discussion in which ways current analyses would be affected by this variability. Some examples, migration analysis in metastatic cancers based on shared subclonal mutations in distinct anatomical locations, identification of subclonal driver mutations, identification of repeated evolutionary trajectories, etc.

3. SomaticSniper vs. MuTect

Looking at Figures 4 and 6, it seems that difference in clonal/subclonal ratio and number of clusters across the pipelines is mainly due to the SNV caller. That is, SomaticSniper calls fewer SNVs than Mutect, and the majority of these SNVs is clonal. It would be good if the authors could dig in more into this. Do SNVs called by SomaticSniper have a larger VAF than those that are not called? Are there parameters that can be set to increase sensitivity?

Minor:

* Introduction: Good to cite https://doi.org/10.1016/j.trecan.2017.06.004, which states that sample trees are not clone trees.

* It would be good to make the used pipelines publicly available.

* Do you expect similar conclusion to hold for whole-exome data?

Reviewer #2 (Remarks to the Author): Expertise in prostate cancer genomics, phylogenetics

In 'Quantifying the Influence of Mutation Detection on Tumour Subclonal Reconstruction', PhyloWGS is used to carry out subclonal reconstruction of tumour phylogenies from sequencing data, based on SNV and CNA calls made by different callers. The authors found that more heterogeneity was identified when using MuTect to call SNVs, compared to SomaticSniper, and that more heterogeneity was identified from multi-sample analyses than single-sample analysis.

The potential value of the paper, summarized by the authors (lines 84-85), is 'generating useful guidance for the community and a resource for improving existing methods and benchmarking new ones.' Unfortunately, the manuscript does not deliver either of these two objectives. The only guidance offered by the paper is the suggestion that readers could use a consensus approach to reconstruct subclonal architectures (lines 352-254), but they offer no evidence that this would give superior results to using a single method. Further, it is unclear what 'resource' this paper presents, as all data used has been previously published.

There is little novelty in the findings. Specifically:
• It is widely reported that more subclones are always identified from multi-sample than single-sample studies.
• Larger bench-making studies have been performed previously (many of them by some of the authors of this paper) on both mutation calling and the identification of subclonality. Previous studies have been more comprehensive, as they have compared a much larger number of callers. They have also been more informative, since most previous studies have used simulated data, and are therefore more useful for bench-marking than this study.
• It has been previously reported that Mutect has greater sensitivity for calling low VAF mutations than other callers. It is therefore unsurprising that more subclones are identified when using this caller.

Although the authors test different SNV and CNA callers, nearly all subclonal reconstruction is carried out with a single algorithm, PhyloWGS. As a result, it is difficult to judge how generalizable the results may be to other algorithms.

On p.10, a 'union' of CNA calls is constructed. It is unclear what is meant by this. For example, how do the authors deal with the situation where Battenberg calls a gain and TITAN calls a loss for the same region?

In the comparison of single and multi-sample reconstruction, it is unclear what is being compared. Specifically, it is likely that some mutations will be clonal in individual samples, but absent from some samples and therefore not clonal across all samples. How is it determined that the multi- and single-sample calls 'agree' in situations like this?
Related to this point, it is NOT surprising that single sample analysis identifies the same number of subclones as multi-sample analysis of the same sample (lines 313-319).

On lines 294-296, the authors state that multi-sample reconstructions are better able to define subclonal populations. This statement is rather misleading, since it is not the reconstruction that leads to the loss of information, but rather the lack of sampling.

The meaning of the categories in Figure 7A ('Match in Multi and Single', etc.) is very unclear.

Reviewer #3 (Remarks to the Author): Expertise in prostate cancer genomics and evolution

The authors used 6 different pipelines to analyse the subclonal structure in a set of 293 primary prostate cancers and 10 patients with multiple index samples sequences. In each pipeline, they used a different combination of different SNV and CNA callers and three different subclonal reconstruction algorithms. The aim is to assess the agreement on the predicted phylogenies. This is a study of interest to the field of cancer genomics pointing to the most important factors when studying clonal structure:

1. Number of tumors sequenced: The authors compared the clonal architectures identified from the analysis of a single lesion and multiple lesions from the same tumor and show that whilst heterogeneity at the tumor level is definitely under-estimated when a single sample is used, the number of clones within a single lesion does not differ between the two analyses. Could this be because the lesions were all taken prior to chemotherapy and from the same tumor site? Did the authors perform a similar analysis from matched pre-/post-therapy or primary/relapse samples? After a clonal sweep, tumors may have a monoclonal architecture with a long trunk but the addition of samples from earlier time points might help identify intermediate clones that were subclonal before therapy/relapse etc.
2. Mutation caller: The authors used SomaticSniper and MuTect to call SNVs and Battenberg TITAN to call CNAs. MuTect has high sensitivity but might also suffer from low specificity. The authors say multiples times that pipelines employing MuTect are more likely to detect more subclones since MuTect can identify variants at lower VAF. However this also makes MuTect to call artefacts as well. Did the authors use an unmatched normal panel to filter MuTect output? If so, what was the effect? Did the authors compare the mutational patterns in the set of substitutions called by both algorithms vs those called by a single caller? How did the mutational signatures differ in the two sets? This would help understand if subclonal mutations called by MuTect are enriched for false positives.
3. Clonal reconstruction algorithm: The authors used PyClone, DPClust and PhyloWGS to show that PyClone tends to call less clones while the agreement between DPClust and PhyloWGS is higher. This is the weakest point of the manuscript as only three tools are considered. A thorough benchmarking exercise would fix the mutation calls (potentially using a high-confidence consensus set from multiple callers) and include more algorithms.

Summary of Revisions

We thank all three reviewers for very thoughtful commentary. Their suggestions triggered a substantial set of experimental changes, and textual edits throughout, including:

- Addition of 10 pipelines for single-sample subclonal reconstruction (new total: 16)
- Addition of 14 pipelines for multi-sample subclonal reconstruction (new total: 18)
- Analysis of SomaticSniper and MuTect SNV detection results and error characteristics
- Quantitation of the influence of algorithmic choices on translational endpoints like outcome

These changes, along with other important textual, visualization and statistical enhancements suggested, are weaved throughout the text and have led us to update effectively every figure. We greatly appreciate the reviewer advice in helping us improve the generalizability of this study!

Reviewer #1

The authors evaluate six pipelines with respect to their variability and consistency in detecting intra-tumor heterogeneity (ITH). Using predominantly single-sample and some multi-region WGS data of prostate cancers, the authors find extensive variability among pipelines in determining clonality status of SNVs and CNAs. This is a timely paper, and of interest to the readership of this journal.

We thank the reviewer for their fair recapitulation of our findings and their thoughtful review.

1. Define subclonal architecture

The authors refer to PhyloWGS, PyClone and DPClust as algorithms to reconstruct subclonal architecture. While such a broad term enables one to classify these three algorithms in the same group, I do encourage the authors to be explicit in their definition of "subclonal architecture". Based on the performed analyses, it seems that subclonal architecture is defined as clusters of somatic mutations (containing SNVs and/or CNAs) with similar cancer cell fraction. Such as a clustering does not fully resolve evolutionary relationships of mutation clusters. In particular, it only enables the designation of clusters as either subclonal or clonal. To fully resolve evolutionary relationships, a tree inference algorithm is required (e.g. PhyloWGS) as opposed to a mutation clustering algorithm (e.g. DPClust and PyClone). It would be good to clarify this in the paper.

We agree that the term "subclonal architecture" has been applied broadly in this context to encompass all algorithms, and exactly as the reviewer suggests was defined as identifying clusters of somatic mutations with similar cancer cell fractions and their cellular prevalence. We absolutely agree that to fully resolve evolutionary relationships, tree inference is required

subsequent to this. Accordingly, we now explicitly define "subclonal architecture" and our choice of "subclonal reconstruction algorithms" in the introduction, which now reads:

> "A variety of algorithms have been developed to reconstruct the subclonal architecture of cancers from single-region or multi-region bulk DNA sequencing data. These algorithms broadly attempt to infer cancer cell populations based on cancer cell fractions (the fraction of cancer cells in which each variant is present) of somatic single nucleotide variants (SNVs) and/or somatic copy number aberrations (CNAs). Several employ Bayesian models to cluster mutations, and estimate the number and prevalence of cancer cell populations. Some algorithms are further able to infer phylogenetic clone trees, thus resolving the evolutionary relationship between mutation clusters."

As an additional discussion point, multiple phylogenetic trees may be inferred from the same data (e.g. [1,2]), also when using PhyloWGS (i.e., there are multiple trees with maximum or near-maximum likelihood). I wonder how inclusion of alternative trees affects Figure 6B.

[1] Y. Qi, D. Pradhan and M. El-Kebir. Implications of non-uniqueness in phylogenetic deconvolution of bulk DNA samples of tumors. Algorithms for Molecular Biology, 14:19, 2019. https://doi.org/10.1186/s13015-019-0155-6

[2] Jamal-Hanjani, M., Wilson, G. A., McGranahan, N., Birkbak, N. J., Watkins, T. B. K., Veeriah, S., et al. (2017). Tracking the Evolution of Non-Small-Cell Lung Cancer. New England Journal of Medicine, 376(22), 2109–2121. http://doi.org/10.1056/NEJMoa1616288

That's a great point, and one we hadn't thought of at all previously! As suggested, we directly assessed the stability of phylogenetic tree inference using PhyloWGS in the new **Supplementary Figure 3**, and identified several interesting patterns in the set of potential trees and the convergence patterns. Further, while tree inference is not directly part of the subclonal reconstruction results that are the core of this study, we have now included this demonstration of how multiple phylogenetic trees may be inferred from the same data.

2. What are the downstream effects of incorrect clonality determination?

To illustrate the importance of accurate quantification of ITH, it would be good if the authors could demonstrate how variability in clonality status affects the conclusions of the original papers. At the very least it would be good to mention in the Discussion in which ways current analyses would be affected by this variability. Some examples, migration analysis in metastatic cancers based on shared subclonal mutations in distinct anatomical locations, identification of subclonal driver mutations, identification of repeated evolutionary trajectories, etc.

Yes, this is an important point to demonstrate how variability in subclonal reconstruction solutions affects downstream analyses, particularly for potential end-users of these types of algorithms. We previously addressed this through analyses 1) and 2), but have now added a third key type of analysis:

1) Evaluation of how the identification of clonal or subclonal mutations in prostate cancer driver genes differs across pipelines (**Supplementary Figure 8**)
2) Quantitation of how algorithm choice influences the identification of genes affected by CNAs biased to occur early or late in tumour evolution (**Supplementary Figure 9**)
3) Reproducibility analysis of the biomarker potential of clonality both in isolation (**Supplementary Figure 10**) and in combination with other prognostic features (**Supplementary Figure 11**) across subclonal reconstruction pipelines.

We also agree with the reviewer regarding the ways that downstream analyses would be affected by clonality status variability and now outline these in the Discussion:

> "The potential translational and clinical impact of these technical variabilities is considerable. For example, technological differences between analysis pipelines were larger than the effect-size of the association between evolutionary complexity and patient survival. This suggests that estimates of technical variability should be provided for analyses dependent on subclonal architecture, such as in studies mapping evolutionary and migration trajectories between primary and metastatic tumours. Studies identifying clonal and especially subclonal driver mutations should be interpreted with such variability estimates for reference as subclonal mutational landscapes were found to be especially vulnerable to pipelines changes when clonal ones were less so. Articulating how these algorithmic differences relate to the clinical effect-size will greatly improve interpretability of these types of data."

3. SomaticSniper vs. MuTect

Looking at Figures 4 and 6, it seems that the difference in clonal/subclonal ratio and number of clusters across the pipelines is mainly due to the SNV caller. That is, SomaticSniper calls fewer SNVs than Mutect, and the majority of these SNVs are clonal. It would be good if the authors could dig in more into this. Do SNVs called by SomaticSniper have a larger VAF than those that are not called? Are there parameters that can be set to increase sensitivity?

We agree: our results suggest that varying the SNV detection tool has more influence on the clonality result than varying the CNA detection tool. We further investigated this and show that SNVs detected uniquely by SomaticSniper have a larger VAF than those detected uniquely by MuTect, and SNVs detected by both tools have the highest VAFs (**Figure 4**; **Supplementary Figure 5**). For the purposes of this end-user benchmarking study, we used default parameters throughout to give a fair evaluation of the baseline performance of each tool, and now articulate this in the Discussion:

"Differences between SNV detection tools led to major divergences in subclonal reconstruction: pipelines using MuTect found extensive subclonal diversity, at least partly due to the greater number of low VAF mutations detected. SNV detection benchmarking efforts could aid in the further characterization of the error profiles of SNV detection tools and optimize parameter tuning to improve subclonal reconstruction."

Minor:

* Introduction: Good to cite https://doi.org/10.1016/j.trecan.2017.06.004, which states that sample trees are not clone trees.

Great point, we now cite this when we distinguish sample and clone trees in the Introduction.

* It would be good to make the used pipelines publicly available.

We agree with the reviewer that reproducible pipelines are valuable for the support of conclusions of any benchmarking study. All of the tools used in our pipelines are publicly available and any additional data processing has been described in detail in the methods section, with any custom analysis code available upon request. We have included exact version numbers for each tool used in our pipelines for reproducibility, but also encourage users to stay up to date as each tool is maintained by their developers.

* Do you expect similar conclusions to hold for whole-exome data?

Subclonal reconstruction from whole-exome sequencing data has its own advantages and disadvantages. We focused solely on whole-genome sequencing data, but the SNV detection tools used (SomaticSniper and MuTect) and some of the subclonal reconstruction algorithms (PyClone, sciClone) can also be applied to whole-exome sequencing data. We expect similar variability between subclonal reconstruction algorithms given the same set of inputs, as we observed in this study, due to the different model architecture and assumptions of each algorithm. Similarly, since our findings suggest that SNV detection tool choice has a larger effect on subclonal reconstruction than CNA detection tool choice, we would hypothesize that subclonal reconstruction results would differ if the results of SNV detection also differ between tools in whole-exome sequencing data.

Reviewer #2

In 'Quantifying the Influence of Mutation Detection on Tumour Subclonal Reconstruction', PhyloWGS is used to carry out subclonal reconstruction of tumour phylogenies from sequencing data, based on SNV and CNA calls made by different callers. The authors found that more heterogeneity was identified when using MuTect to call SNVs, compared to SomaticSniper, and that more heterogeneity was identified from multi-sample analyses than single-sample analysis.

The potential value of the paper, summarized by the authors (lines 84-85), is 'generating useful guidance for the community and a resource for improving existing methods and benchmarking new ones.' Unfortunately, the manuscript does not deliver either of these two objectives. The only guidance offered by the paper is the suggestion that readers could use a consensus approach to reconstruct subclonal architectures (lines 352-254), but they offer no evidence that this would give superior results to using a single method. Further, it is unclear what 'resource' this paper presents, as all data used has been previously published.

We thank the reviewer for their comments and appreciate their thoughtful review, and agree these are key findings of our work. We apologize for the lack of clarity on the guidance on subclonal reconstruction for the community, and have offered a summary of our findings in different parts of the updated Discussion to serve as considerations for subclonal reconstruction pipeline selection:

> "Subclonal reconstruction algorithms differ substantially in their prediction of subclonal architecture across all mutation detection tool combinations, with no pair of algorithms consistently achieving similar results in cellularity estimates, prediction of subclone number and assignment of mutation clonality."
>
> "While the subclonal CNA detection tool used mostly influenced cellularity estimates but no other aspects of subclonal architecture, large differences were driven by changing the SNV detection approach."
>
> "Future studies might benefit from merging multiple subclonal reconstruction pipelines, for example to provide a potential envelope of upper and lower bounds on different features of the reconstruction."
>
> "This suggests that estimates of technical variability should be provided for analyses dependent on subclonal architecture, such as in studies mapping evolutionary and migration trajectories between primary and metastatic tumours. Studies identifying clonal and especially subclonal driver mutations should be interpreted with such variability estimates for reference as subclonal mutational landscapes were found to be especially vulnerable to pipelines changes when clonal ones were less so."
>
> "Future studies also need to carefully consider the failure-rates of different reconstruction algorithms, as algorithms leveraging clonal or neutral copy number regions might not be suitable for tumour types characterized by large numbers of CNAs and might call for specific CNA detection strategies."

We agree that the resource value of our work does not lie in the previously published sequencing data for the samples analyzed in this study, but in the subclonal reconstruction results from multiple pipelines reported for the first time here. These results not only provide developers an unbiased comparison of their algorithms on a large cohort, but also provide users of any individual subclonal reconstruction algorithm an expectation of the variability of reconstruction results and how that might be influenced by their choice of mutation detection tools. Indeed, given the computational demands of many of these methods, this represents a significant investment of time and computational resources that we believe the community working on both algorithm development and considering ensembles of multiple methods may be able to build upon.

There is little novelty in the findings. Specifically:
• It is widely reported that more subclones are always identified from multi-sample than single-sample studies.
• Larger bench-making studies have been performed previously (many of them by some of the authors of this paper) on both mutation calling and the identification of subclonality. Previous studies have been more comprehensive, as they have compared a much larger number of callers. They have also been more informative, since most previous studies have used simulated data, and are therefore more useful for bench-marking than this study.
• It has been previously reported that Mutect has greater sensitivity for calling low VAF mutations than other callers. It is therefore unsurprising that more subclones are identified when using this caller.

The reviewer has raised a series of excellent points that reflect the current knowledge of the field. We believe the strength of our study lies in the use of a real clinical cohort and we are glad to have recapitulated key findings of the field. Simulated data are of great value for benchmarking efforts because they provide a ground truth, but may not fully capture the biases and characteristics of experimental data. This study complements other benchmarking efforts by demonstrating a lower-bound of subclonal reconstruction variability in real samples. Further, our study focused on the assessment of subclonal reconstruction pipelines, and our results demonstrate that the choice of SNV detection tool, CNA detection tool and subclonal reconstruction algorithm each have different influences on the subclonal reconstruction results. This differs from previous studies that have considered a single mutation detection tool or have compared subclonal reconstruction algorithms based on consensus SNV and CNA inputs, and highlights the need for deliberate decision making at each step of the subclonal reconstruction process.

Although the authors test different SNV and CNA callers, nearly all subclonal reconstruction is carried out with a single algorithm, PhyloWGS. As a result, it is difficult to judge how generalizable the results may be to other algorithms.

We agree that it would be valuable to evaluate different SNV and CNA detection tool combinations for multiple subclonal reconstruction algorithms to see how the results generalize. We have addressed this by further evaluating PyClone, DPClust and sciClone as subclonal reconstruction algorithms using inputs from four combinations of SNV and CNA detection tools, increasing the number of pipelines evaluated for single-region reconstructions to sixteen. For multi-region reconstructions, we have included an additional CNA detection tool (FACETS) and further evaluated PyClone and sciClone as subclonal reconstruction algorithms using inputs from six combinations of SNV and CNA detection tools, increasing the number pipelines evaluated to eighteen. We have updated **Figure 1** and all aspects of the manuscript to present our additional findings.

On p.10, a 'union' of CNA calls is constructed. It is unclear what is meant by this. For example, how do the authors deal with the situation where Battenberg calls a gain and TITAN calls a loss for the same region?

We thank the reviewer for pointing this out and have clarified this in the text by adding a reference to the Methods. A union of CNA detection results, specifically, is constructed as follows:

> "The intersection of CNAs, based on genomic coordinates and major and minor copy number, was determined using the GenomicRanges (v1.28.6) package in R (v3.2.5). Regions with disagreeing copy number were identified using bedtools (v2.27.1) and bedr (v1.0.6). A region is defined to have a tool-unique CNA if one tool detected a copy number aberration for the region while the other identified it as copy number neutral (major and minor copy number of 1). Regions were both algorithms detected different copy number aberrations were classified as disagreements. The union set of CNAs thus contained the intersection of CNAs and CNAs unique to either tool, and regions of disagreement were excluded as there was no natural way to resolve discrepancies."

In the comparison of single and multi-sample reconstruction, it is unclear what is being compared. Specifically, it is likely that some mutations will be clonal in individual samples, but absent from some samples and therefore not clonal across all samples. How is it determined that the multi- and single-sample calls 'agree' in situations like this?
Related to this point, it is NOT surprising that single sample analysis identifies the same number of subclones as multi-sample analysis of the same sample (lines 313-319).

The focus of our comparison of single- and multi-sample reconstructions is on the agreement of mutation clonality assignment and the number of subclones predicted in each sample. We defined agreement in SNV clonality assignment strictly as cases where both single- and multi-sample reconstructions assigned the SNV as clonal or subclonal in that sample of the tumour. For SNVs that are clonal in individual samples but subclonal in multi-region reconstructions, they were assigned the category "Subclonal in multi-region" to indicate SNV was identified in both single-

and multi-region reconstructions, but the clonality of the SNV was assigned to be subclonal in the multi-region reconstruction, and clonal in the single-region reconstruction.
We have also updated both the statistical analysis and the manuscript to more objectively reflect our findings for comparing the number of subclones detected in single- and multi-region reconstructions of the same sample, which now reads:

> "On the other hand, multi-sample reconstructions also predicted significantly more subclones within the index lesion sample compared to single-sample reconstruction of the index lesion in pipelines using PhyloWGS (p = 2.4 x 10-4, Wilcoxon signed-rank test; **Supplementary Figure 12A**), but not those using PyClone (p ≈ 1, Wilcoxon signed-rank test; **Supplementary Figure 12B**)."

On lines 294-296, the authors state that multi-sample reconstructions are better able to define subclonal populations. This statement is rather misleading, since it is not the reconstruction that leads to the loss of information, but rather the lack of sampling.

Yes, absolutely true, we didn't realize our phrasing on this was poor. We have updated the manuscript to more clearly reflect this critical point:

> "Consistent with simulations and previous observations, multi-region reconstructions are able to better define subclonal populations of cells by identifying and disambiguating those missed or merged by single-region sampling."

The meaning of the categories in Figure 7A ('Match in Multi and Single', etc.) is very unclear.

We apologize for the lack of clarity. This figure is aimed at comparing SNV clonality assignment between single-sample and multi-sample reconstructions, thus 'Match in Multi and Single' refers to the proportion of SNVs that have matched clonality assignment in both single- and multi-region reconstructions (*i.e.* SNV in the clonal cluster of both single- and multi-region reconstruction, or in subclonal clusters of both). The remaining categories describe cases of discordance in SNV clonality assignment, as defined in the **Methods**. We have updated the figure legend of **Figure 6B** and **Figure 7B** to include the definition of these categories.

Reviewer #3

The authors used 6 different pipelines to analyse the subclonal structure in a set of 293 primary prostate cancers and 10 patients with multiple index samples sequences. In each pipeline, they used a different combination of different SNV and CNA callers and three different subclonal reconstruction algorithms. The aim is to assess the agreement on the predicted phylogenies. This is a study of interest to the field of cancer genomics pointing to the most important factors when studying clonal structure:

We thank the reviewer for their fair summary of our work and its aims, and their thoughtful review.

1. Number of tumors sequenced: The authors compared the clonal architectures identified from the analysis of a single lesion and multiple lesions from the same tumor and show that whilst heterogeneity at the tumor level is definitely under-estimated when a single sample is used, the number of clones within a single lesion does not differ between the two analyses. Could this be because the lesions were all taken prior to chemotherapy and from the same tumor site? Did the authors perform a similar analysis from matched pre-/post-therapy or primary/relapse samples? After a clonal sweep, tumors may have a monoclonal architecture with a long trunk but the addition of samples from earlier time points might help identify intermediate clones that were subclonal before therapy/relapse etc.

This is a very interesting point. We fully agree that tumours may have a monoclonal architecture at the time of sample acquisition due to a recent complete clonal sweep. We also agree that levels of intra-tumour heterogeneity could be influenced by the presence of additional selection such as from chemotherapy. This cohort is, as the reviewer correctly surmised, entirely pre-chemotherapy. That being said they were intentionally spatially quite distant within the primary gland, and several showed substantial morphological differences (for example grade and sub-histology). Our results suggest that the accuracy of single-region reconstructions for resolving intra-tumoural heterogeneity in both single samples and for the entire tumour is limited by the level of spatial heterogeneity. We do not (yet) have matched pre- and post-therapy or primary and relapse samples because of the long natural life-history of prostate cancer (median time to metastasis in this clinical setting is >10 years), but we absolutely agree with the speculation that the relapsed tumours will likely have very long trunks (which may indeed lengthen during subsequent courses of androgen deprivation therapy).

2. Mutation caller: The authors used SomaticSniper and MuTect to call SNVs and Battenberg TITAN to call CNAs. MuTect has high sensitivity but might also suffer from low specificity. The authors say multiple times that pipelines employing MuTect are more likely to detect more subclones since MuTect can identify variants at lower VAF. However this also makes MuTect to

call artefacts as well. Did the authors use an unmatched normal panel to filter MuTect output? If so, what was the effect? Did the authors compare the mutational patterns in the set of substitutions called by both algorithms vs those called by a single caller? How did the mutational signatures differ in the two sets? This would help understand if subclonal mutations called by MuTect are enriched for false positives.

We thank the reviewer raising these excellent points and agree that it would be valuable to assess whether mutations detected by MuTect have a different error profile than SomaticSniper. In this study we have applied the same set of allow- and deny-list filtering on MuTect and SomaticSniper detected SNVs to remove known germline contaminants and sequencing artifacts and preserve COSMIC variants. We have now evaluated the effect of filtering on the VAF of mutations detected by MuTect and SomaticSniper in new **Figure 4** and **Supplementary Figure 5**. Though we have no ground truth, we have also investigated the trinucleotide profiles pre- and post-filtering, for mutations detected by both tools compared to those by a single tool.

3. Clonal reconstruction algorithm: The authors used PyClone, DPClust and PhyloWGS to show that PyClone tends to call less clones while the agreement between DPClust and PhyloWGS is higher. This is the weakest point of the manuscript as only three tools are considered. A thorough benchmarking exercise would fix the mutation calls (potentially using a high-confidence consensus set from multiple callers) and include more algorithms.

Indeed. Very good point and this was also discussed by Reviewer #2. We fully agree that it would be valuable to evaluate more subclonal reconstruction algorithms, and have now evaluated all 293 samples using PyClone, DPClust and PhyloWGS in pipelines with all four combinations of SNV and CNA detection tools, as well as with sciClone as an additional subclonal reconstruction algorithm that also considers both SNVs and CNAs as inputs. This increases the number of subclonal reconstruction pipelines evaluated for single-sample reconstruction to sixteen. We have further included the subclonal CNA detection tool FACETS for multi-region reconstructions, and evaluated PyClone, sciClone and PhyloWGS in pipelines with six combinations of mutation detection tools. This increases the number of subclonal reconstruction pipelines evaluated for multi-sample reconstruction to eighteen. We have updated **Figure 1** and all aspects of the manuscript to present this more thorough benchmarking effort. We did not pursue the creation of a high-confidence consensus mutation input set as we believe that evaluating subclonal reconstruction algorithms in pipelines with the same SNV and CNA mutation detection tool combination provides a fair comparison of their performance. A strength of our study also lies in demonstrating how different subclonal reconstruction algorithms are differentially affected by the mutational detection tools used, and how each step of the subclonal reconstruction pipeline (SNV detection tool, CNA detection tool, subclonal reconstruction algorithm) impacts the final solutions.

Reviewer #1 (Remarks to the Author):

My previous comments have been satisfactorily addressed. The changes are substantial and greatly improve the generalizability of this work. The revised manuscript evaluates a larger number of common pipelines (with varying SNV & CNA callers and mutation clustering algorithms), showing consistent biases and differences in results that in turn affect downstream analyses. These findings will be of interest to the cancer genomics community as well as the readership of the journal.

Reviewer #2 (Remarks to the Author):

The manuscript is now considerably improved. I have just a few minor comments:

Lines 262-264 refer to 'subclone number', but the whole of the previous paragraph has described the number of mutations that are identified as clonal. The authors should clarify whether 'subclone number' actually refers to the number of subclones identified within a sample or the number of subclonal mutations.

Lines 381-384 state that 'most' mutations identified as subclonal from sample reconstruction are also subclonal from multi-sample reconstruction. This is unsurprising, but the low percentage of mutations within this category, 12.2%, should not be described as 'most'.

Figure 6C has a typo 'CNA Assignemnt'

Summary of Revisions

The major revisions made are:

- Reformatting the manuscript, figures and supplementary materials to meet *Nature Communications* formatting guidelines
- Rewording areas of the main text for clarity and typo correction

Reviewer #1

My previous comments have been satisfactorily addressed. The changes are substantial and greatly improve the generalizability of this work. The revised manuscript evaluates a larger number of common pipelines (with varying SNV & CNA callers and mutation clustering algorithms), showing consistent biases and differences in results that in turn affect downstream analyses. These findings will be of interest to the cancer genomics community as well as the readership of the journal.

We thank the reviewer for their comments and appreciate the time they took to review our work.

Reviewer #2

The manuscript is now considerably improved. I have just a few minor comments:

We thank the reviewer for their comments and appreciate the thoughtful review.

Lines 262-264 refer to 'subclone number', but the whole of the previous paragraph has described the number of mutations that are identified as clonal. The authors should clarify whether 'subclone number' actually refers to the number of subclones identified within a sample or the number of subclonal mutations.

We thank the reviewer for pointing this out and apologize for the sudden change in focus and lack of clarity. "Subclone number" indeed refers to the number of subclones predicted in a sample and we have modified the sentence to improve clarity:

> "Given the lack of correlation between subclonal reconstruction algorithms in estimating the number of subclones present in a sample, this could suggest that disagreements between subclonal reconstruction algorithms mostly fall in defining the subclonal populations."

Lines 381-384 state that 'most' mutations identified as subclonal from sample reconstruction are also subclonal from multi-sample reconstruction. This is unsurprising, but the low percentage of mutations within this category, 12.2%, should not be described as 'most'.

We apologize for the confusion surrounding this sentence due to the lack of explanation of what the 12.2% figure represents. As the 12.2% quoted here refers to the percentage of subclonal SNVs in single-region reconstruction that have been found to be *clonal* in multi-region reconstruction, we wrote that *most* SNVs identified as subclonal from single-sample reconstruction are also *subclonal* in multi-sample reconstruction. We have modified the sentence in order to improve readability:

> "A similar analysis of subclonal SNVs showed that, as expected, only a small proportion of subclonal SNVs defined by single-region reconstructions of the index lesion was clonal in multi-region reconstructions in pipelines using PhyloWGS and MuTect (12.2 ± 17.5%, mean ± SD)."

Figure 6C has a typo 'CNA Assignemnt'

We thank the reviewer for pointing this out, the typo has been corrected.
