# Supplementary material for: Quantifying the influence of mutation detection on tumour subclonal reconstruction
Source: Nat Commun. 2020 Dec 7;11:6247. doi: 10.1038/s41467-020-20055-w (PMC7721877; doi:10.1038/s41467-020-20055-w)

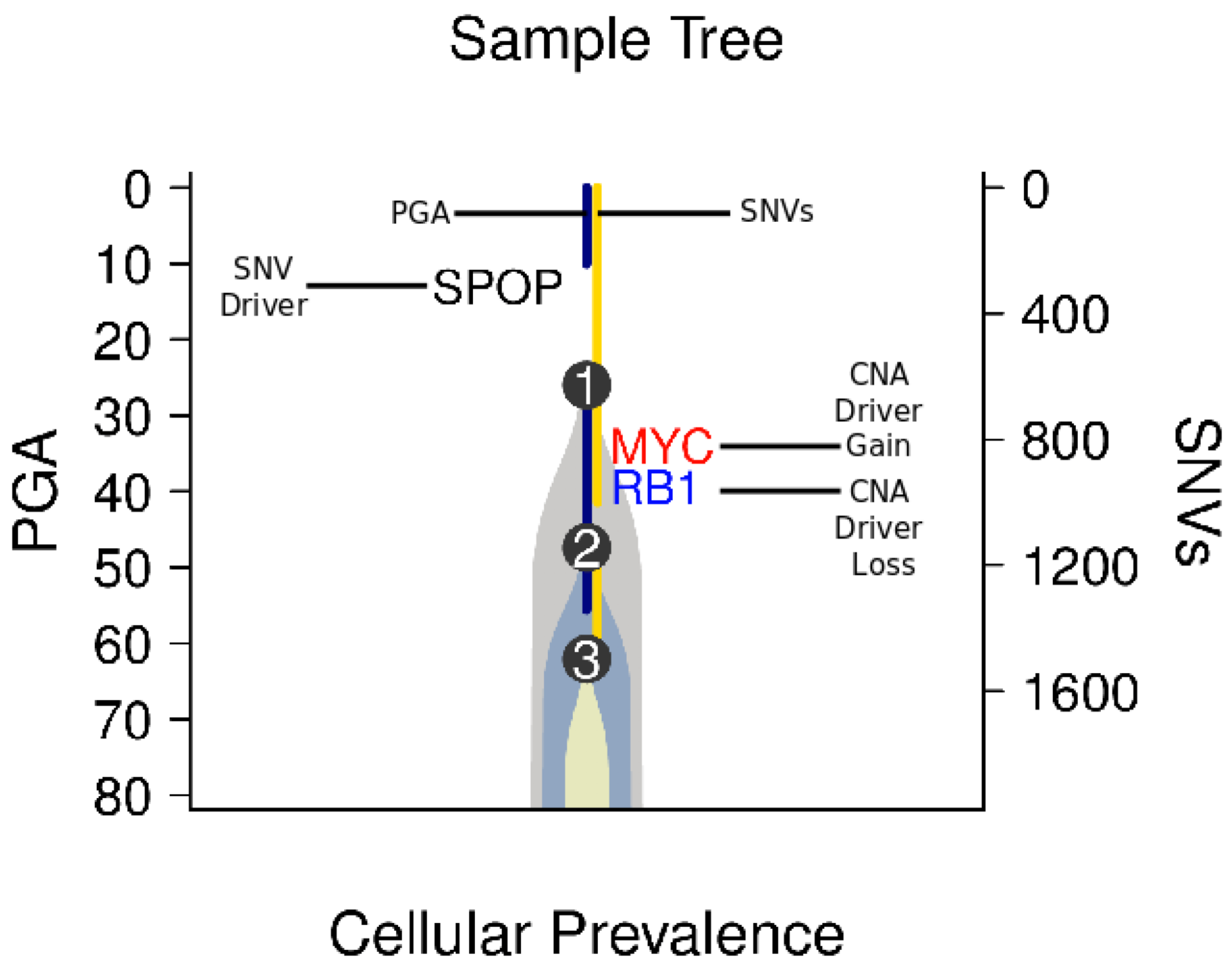
Sample Tree
PGA
SNVs
SNV Driver
SPOP
CNA Driver Gain
MYC
RB1
CNA Driver Loss
PGA
0
10
20
30
40
50
60
70
80
SNVs
0
400
800
1200
1600
1
2
3
Cellular Prevalence

Baca_01−28_T_wgs

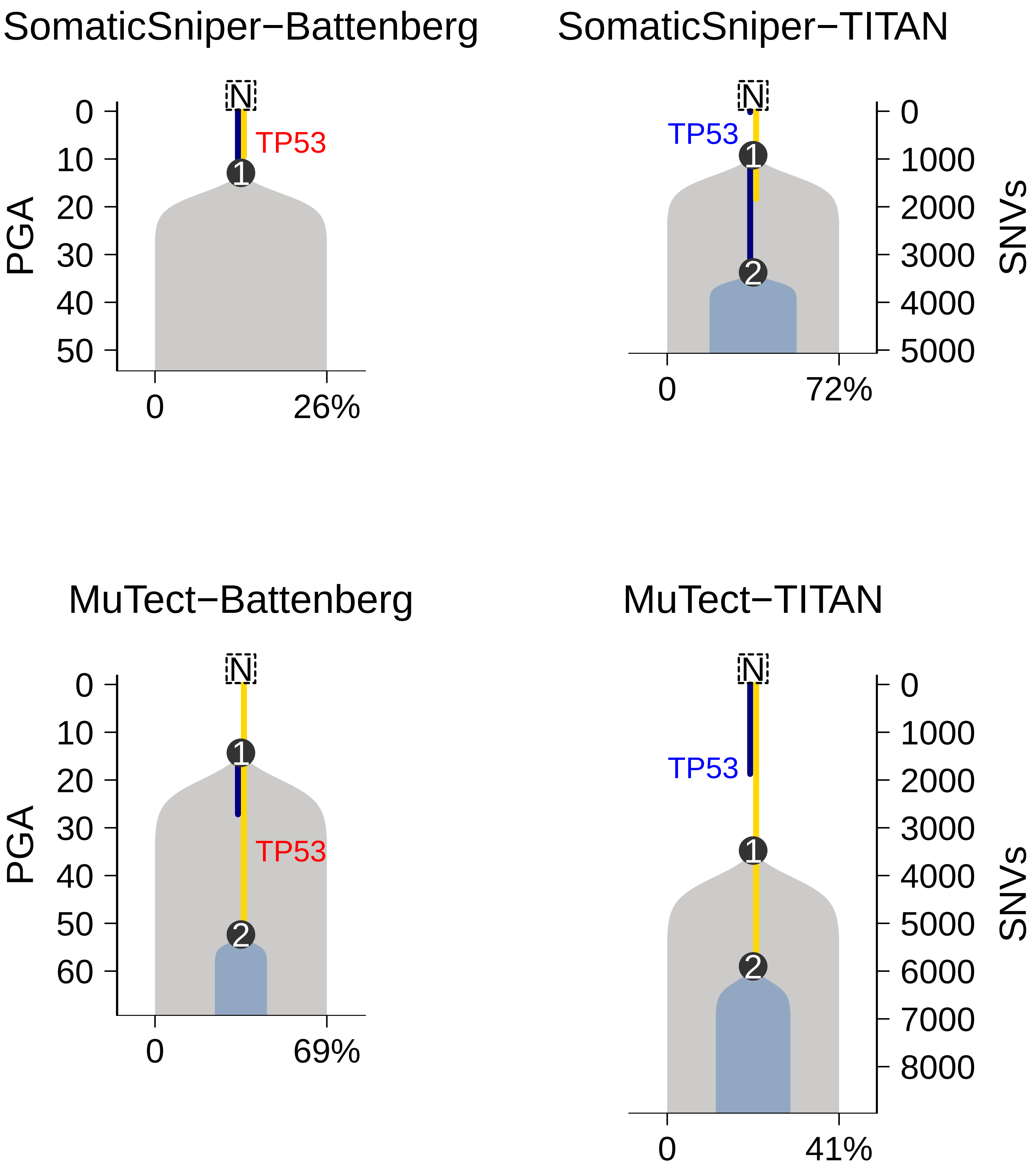

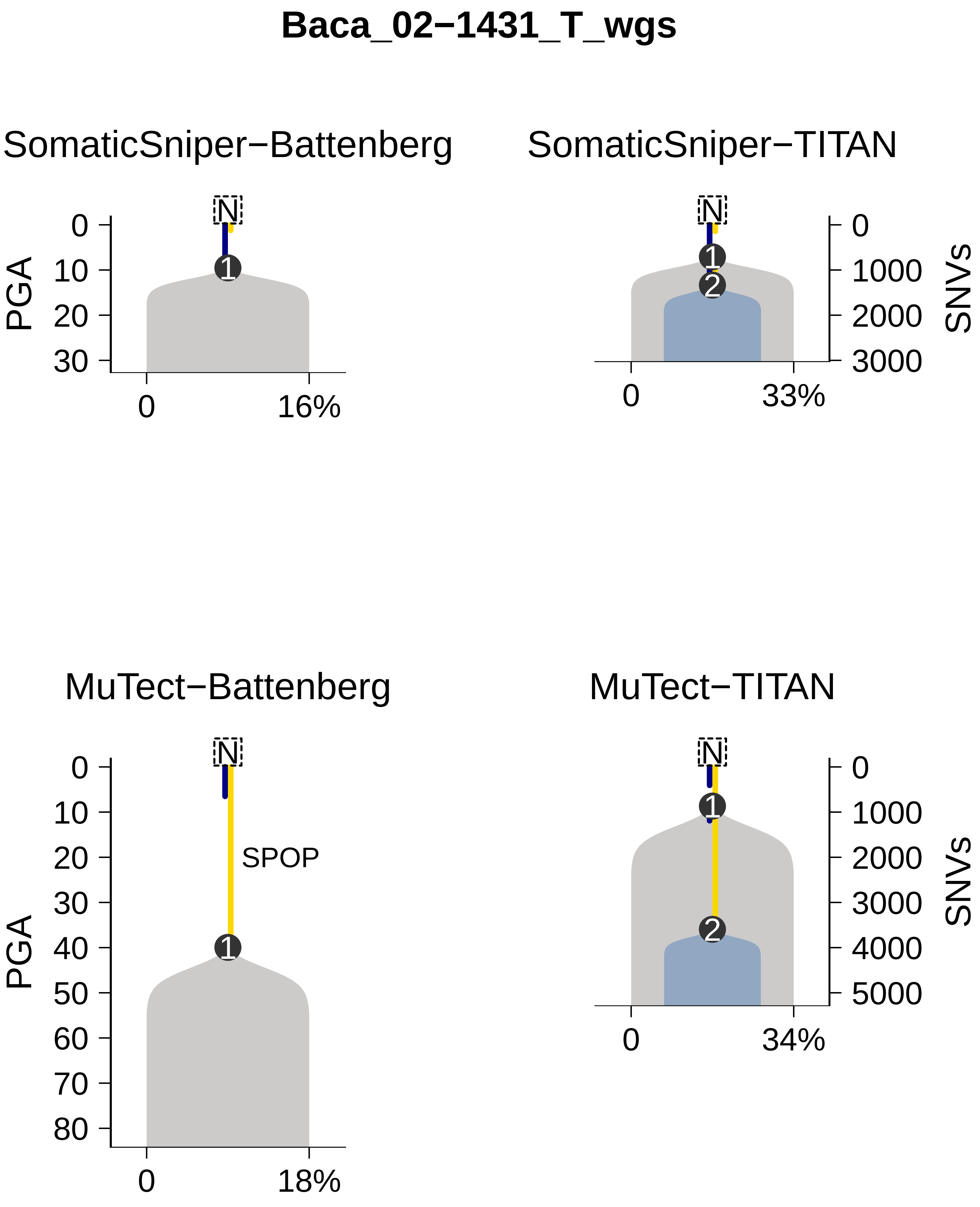
Baca_02−1431_T_wgs
SomaticSniper−Battenberg
SomaticSniper−TITAN
MuTect−Battenberg
MuTect−TITAN
N
1
2
PGA
SNVs
0
10
20
30
40
50
60
70
80
16%
33%
18%
34%
1000
2000
3000
4000
5000
SPOP

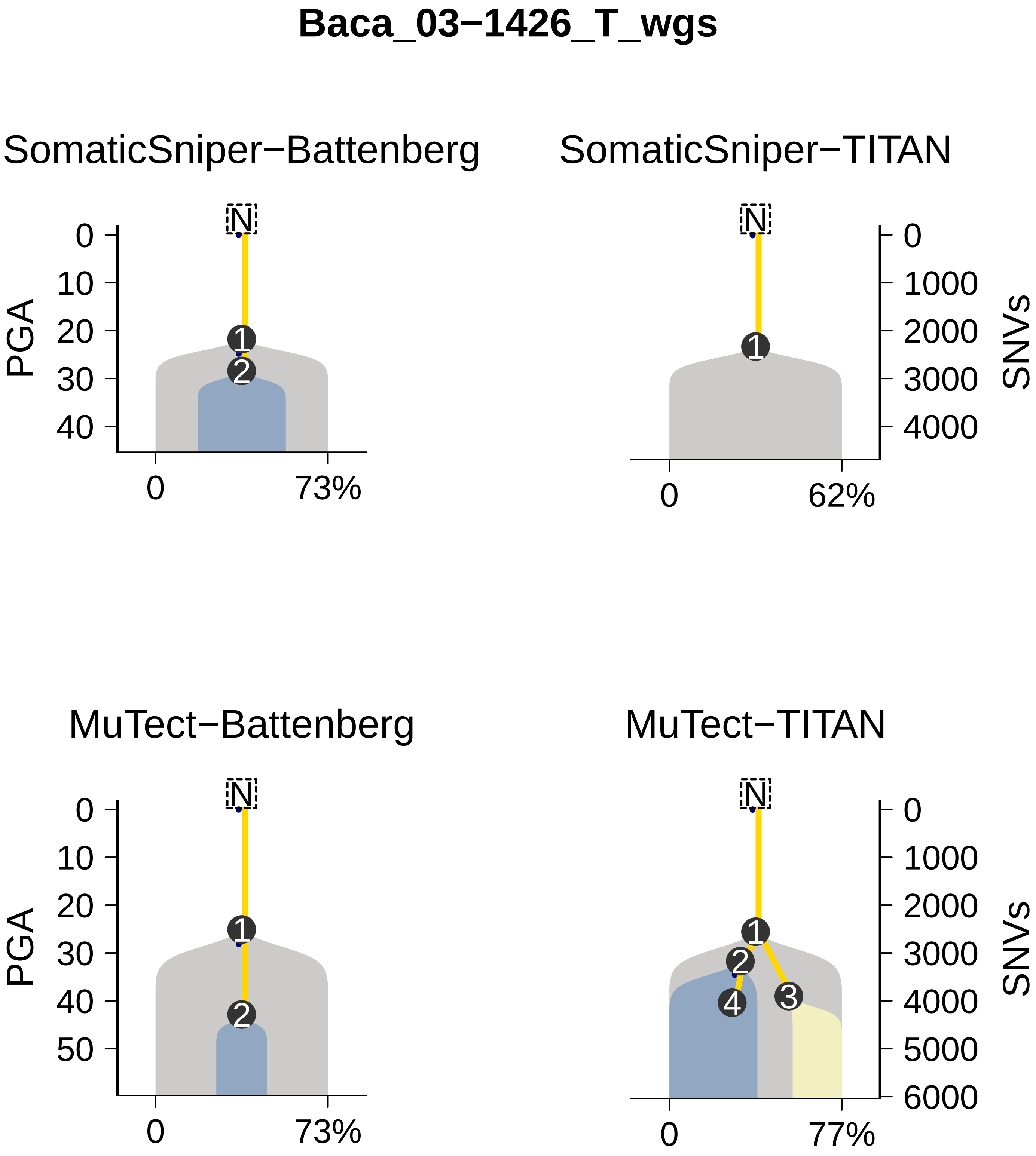

Baca_03−1426_T_wgs
SomaticSniper−Battenberg
SomaticSniper−TITAN
MuTect−Battenberg
MuTect−TITAN
PGA
SNVs
N
1
2
3
4
0
10
20
30
40
50
1000
2000
3000
4000
5000
6000
73%
62%
77%

Baca_03−2345_T_wgs

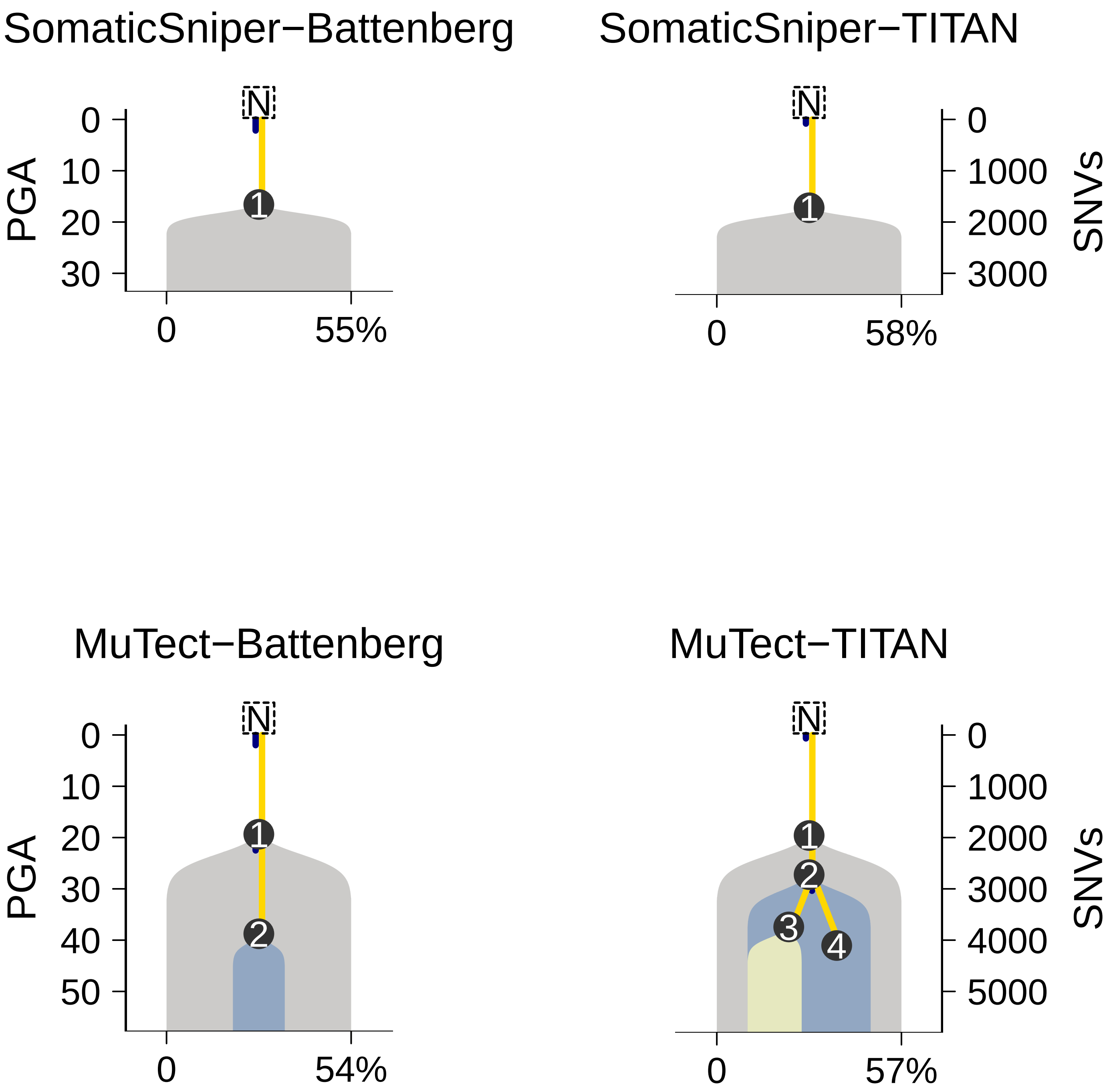

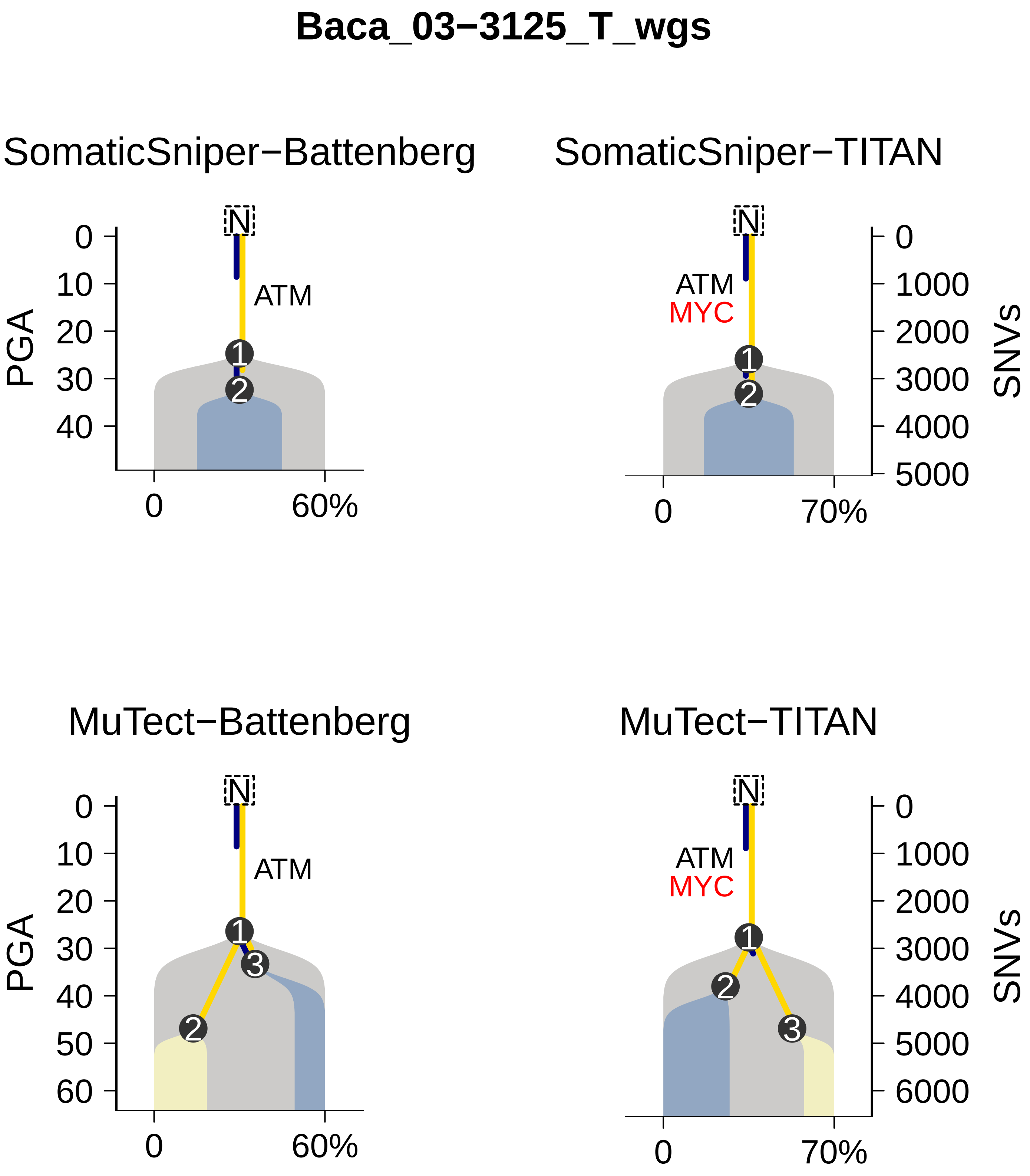
Baca_03−3125_T_wgs
SomaticSniper−Battenberg
SomaticSniper−TITAN
MuTect−Battenberg
MuTect−TITAN
N
ATM
MYC
PGA
SNVs
0
10
20
30
40
50
60
1000
2000
3000
4000
5000
6000
60%
70%

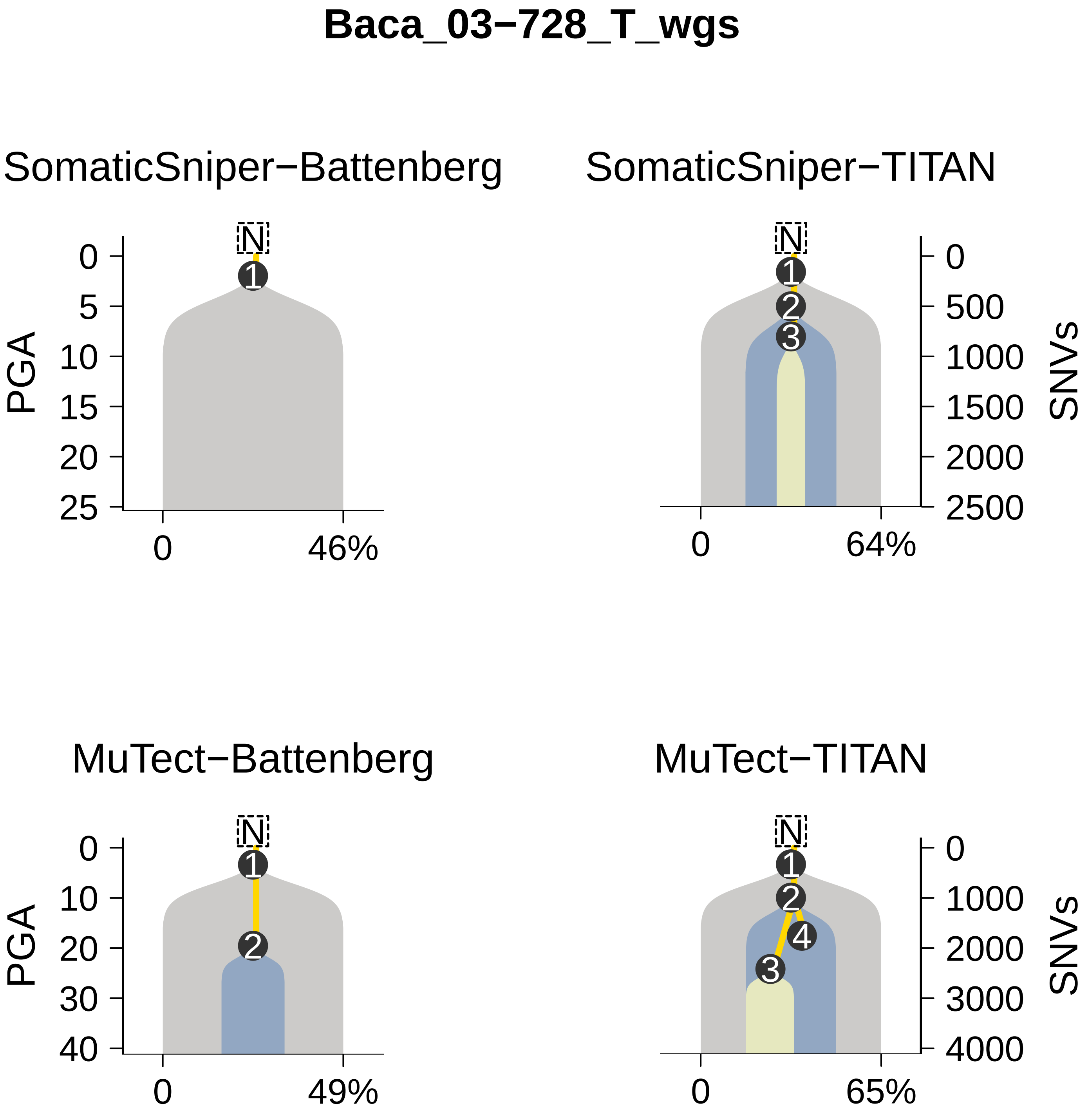

Baca_03−728_T_wgs
SomaticSniper−Battenberg
SomaticSniper−TITAN
MuTect−Battenberg
MuTect−TITAN
N
1
2
3
4
PGA
SNVs
0
5
10
15
20
25
30
40
500
1000
1500
2000
2500
3000
4000
46%
64%
49%
65%

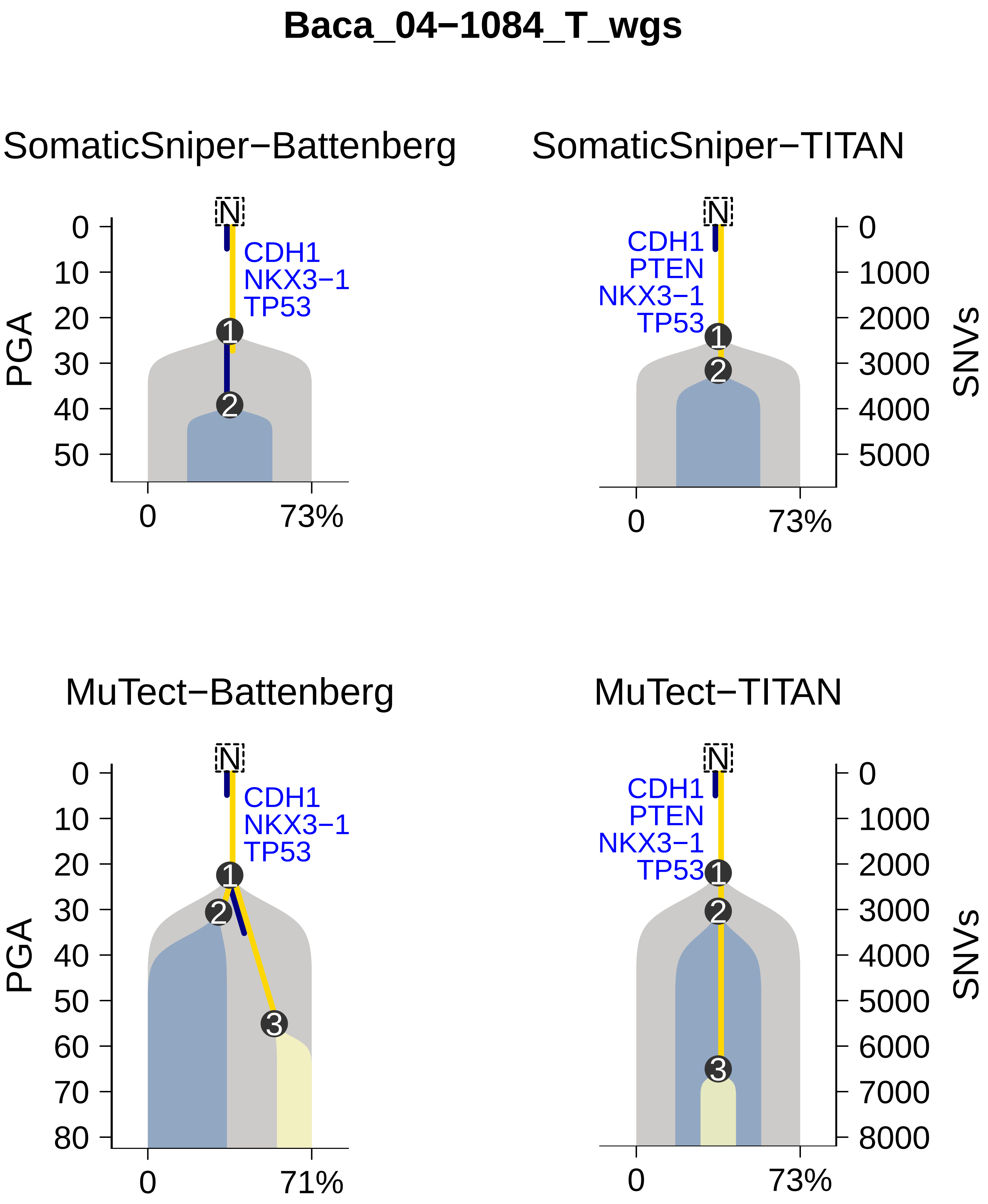

Baca_04−1084_T_wgs
SomaticSniper−Battenberg
SomaticSniper−TITAN
MuTect−Battenberg
MuTect−TITAN
N
CDH1
NKX3−1
TP53
PTEN
PGA
SNVs
0
10
20
30
40
50
60
70
80
1000
2000
3000
4000
5000
6000
7000
8000
73%
71%
1
2
3

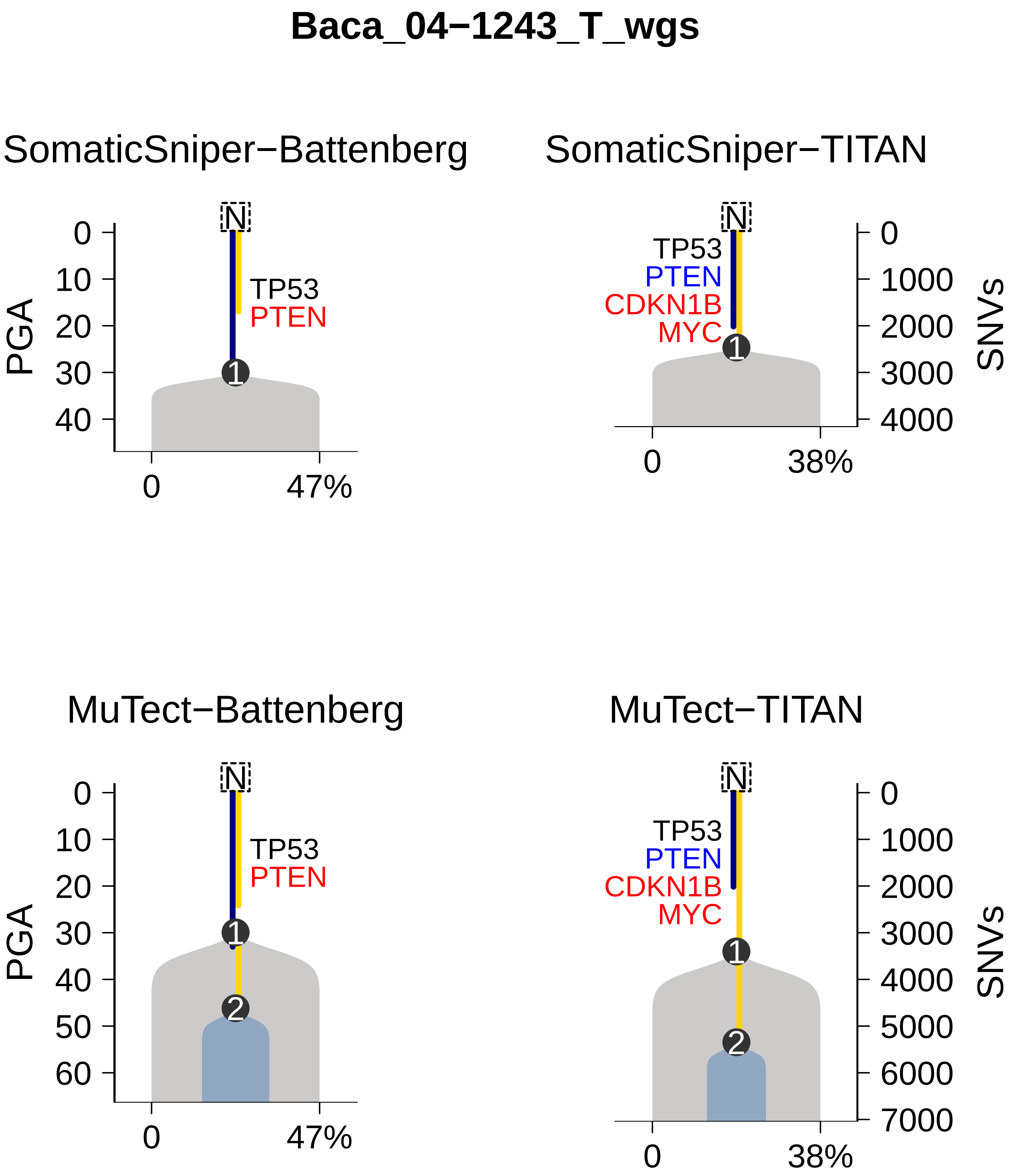
Baca_04−1243_T_wgs
SomaticSniper−Battenberg
SomaticSniper−TITAN
N
PGA
0
10
20
30
40
TP53
PTEN
1
0
47%
TP53
PTEN
CDKN1B
MYC
1
0
1000
2000
3000
4000
SNVs
0
38%
MuTect−Battenberg
MuTect−TITAN
0
10
20
30
40
50
60
PGA
TP53
PTEN
1
2
0
47%
TP53
PTEN
CDKN1B
MYC
1
2
0
1000
2000
3000
4000
5000
6000
7000
SNVs
0
38%

Baca_05−1657_T_wgs

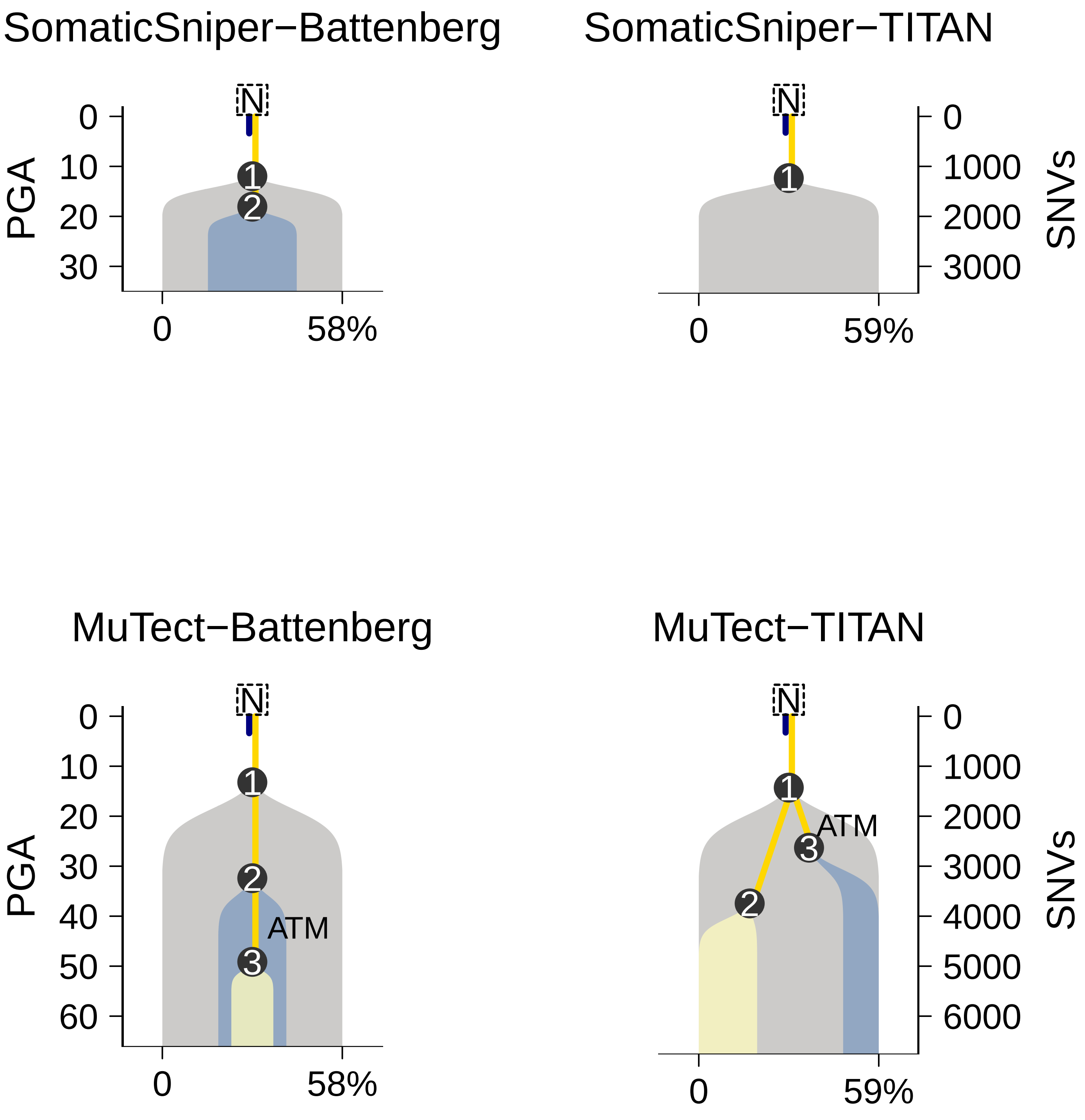

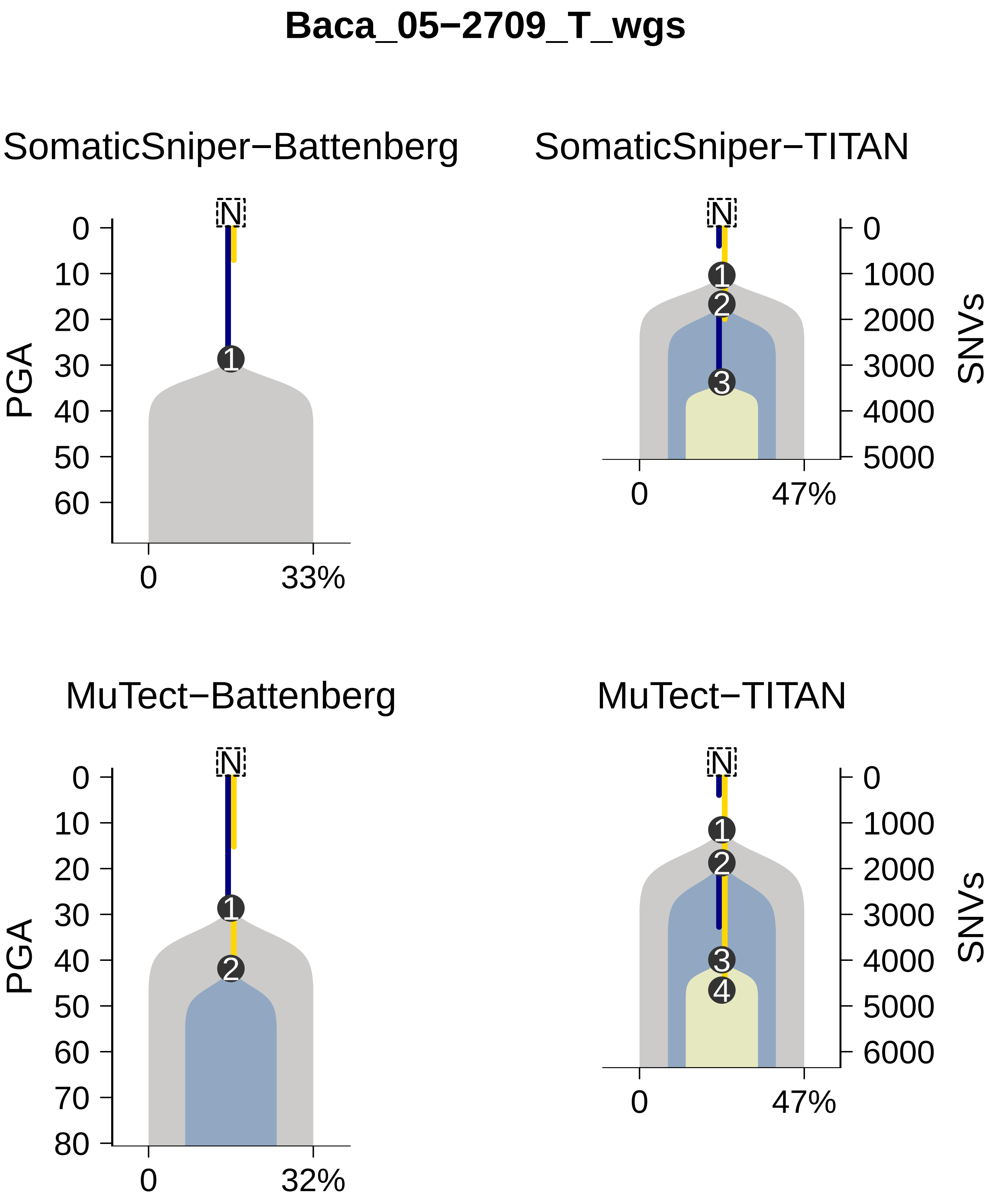
Baca_05−2709_T_wgs
SomaticSniper−Battenberg
SomaticSniper−TITAN
MuTect−Battenberg
MuTect−TITAN
PGA
SNVs
N
0
10
20
30
40
50
60
70
80
0
1000
2000
3000
4000
5000
6000
33%
47%
32%

Baca_05−3595_T_wgs

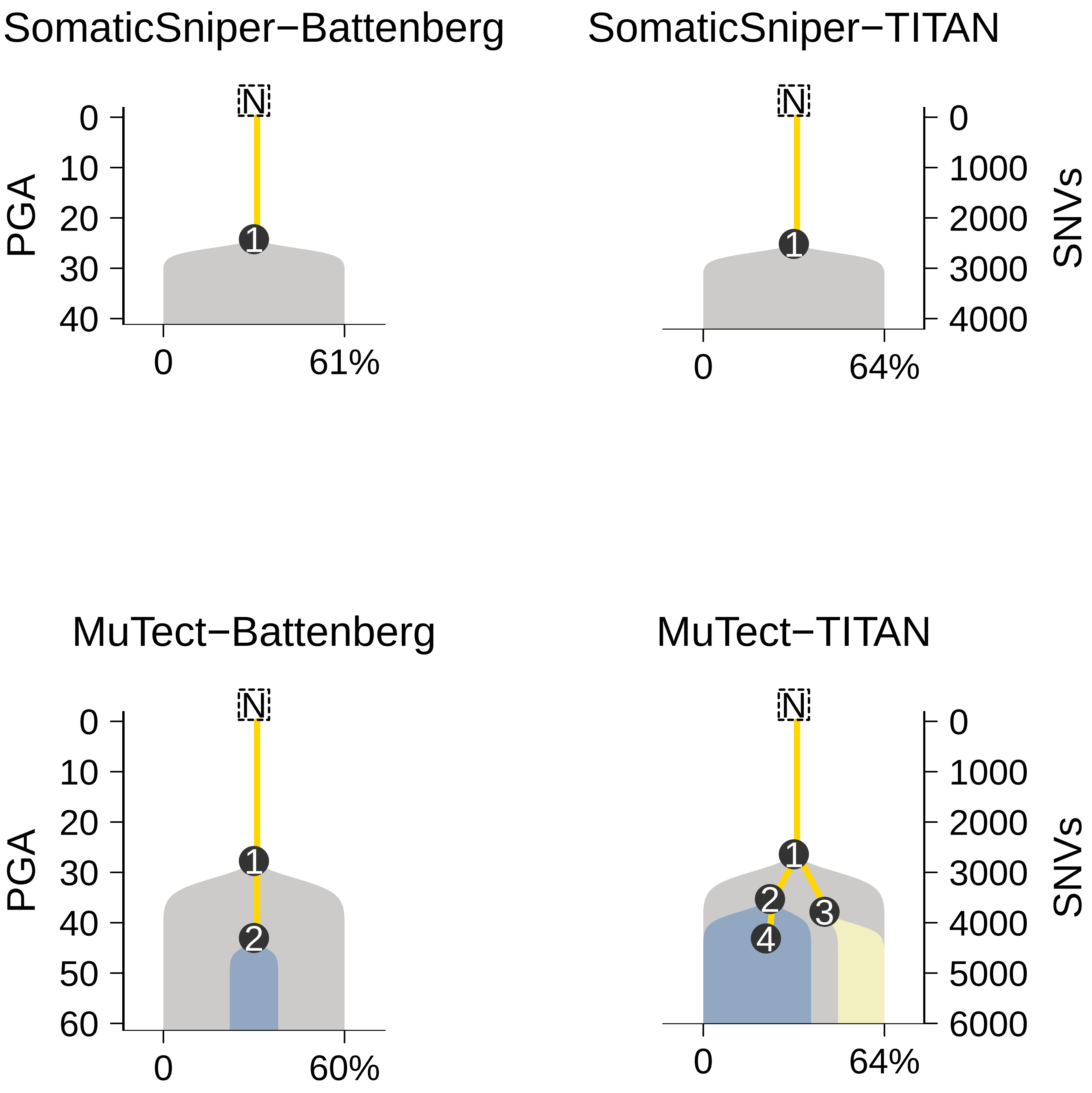

Baca_05−3852_T_wgs

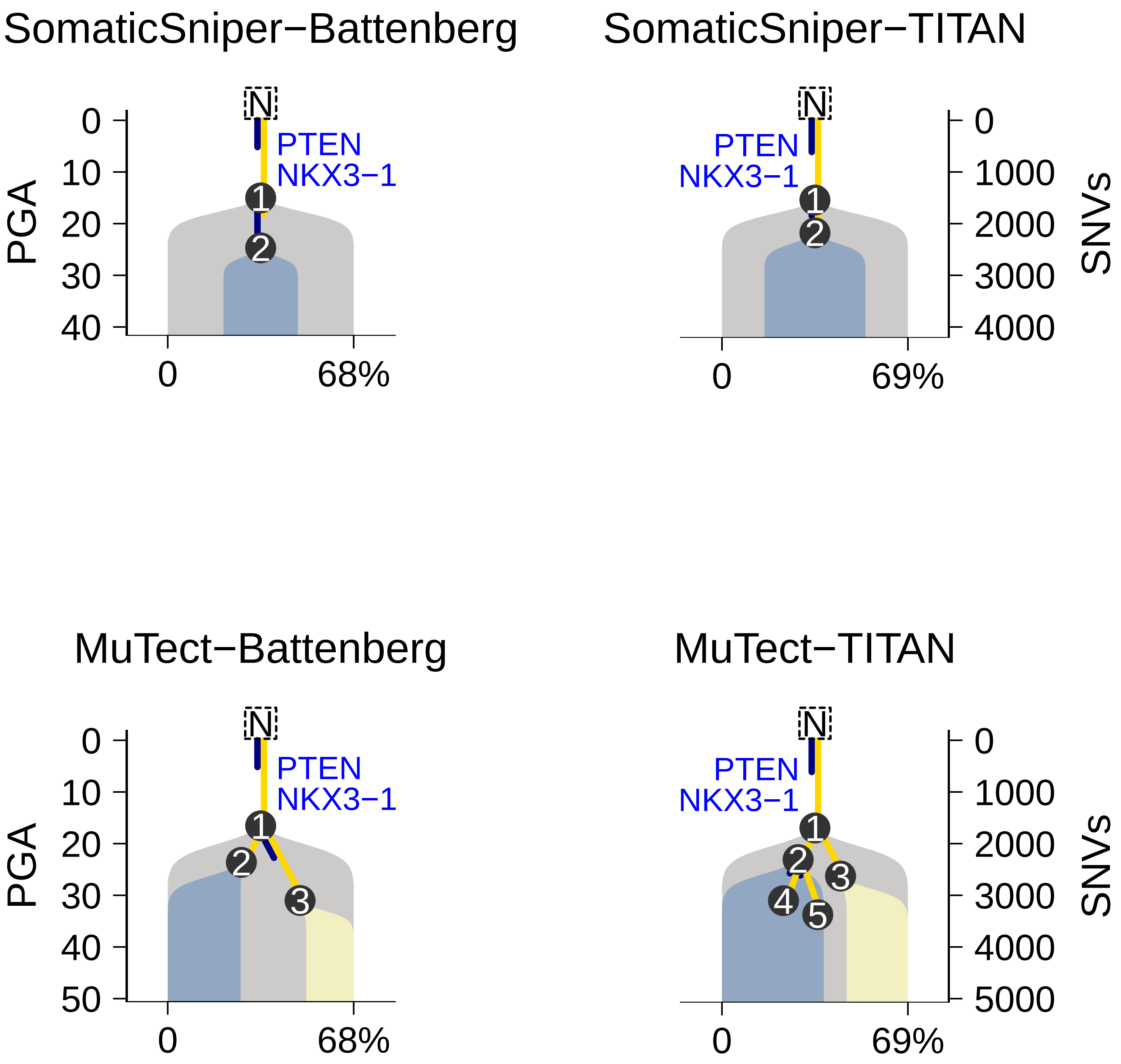

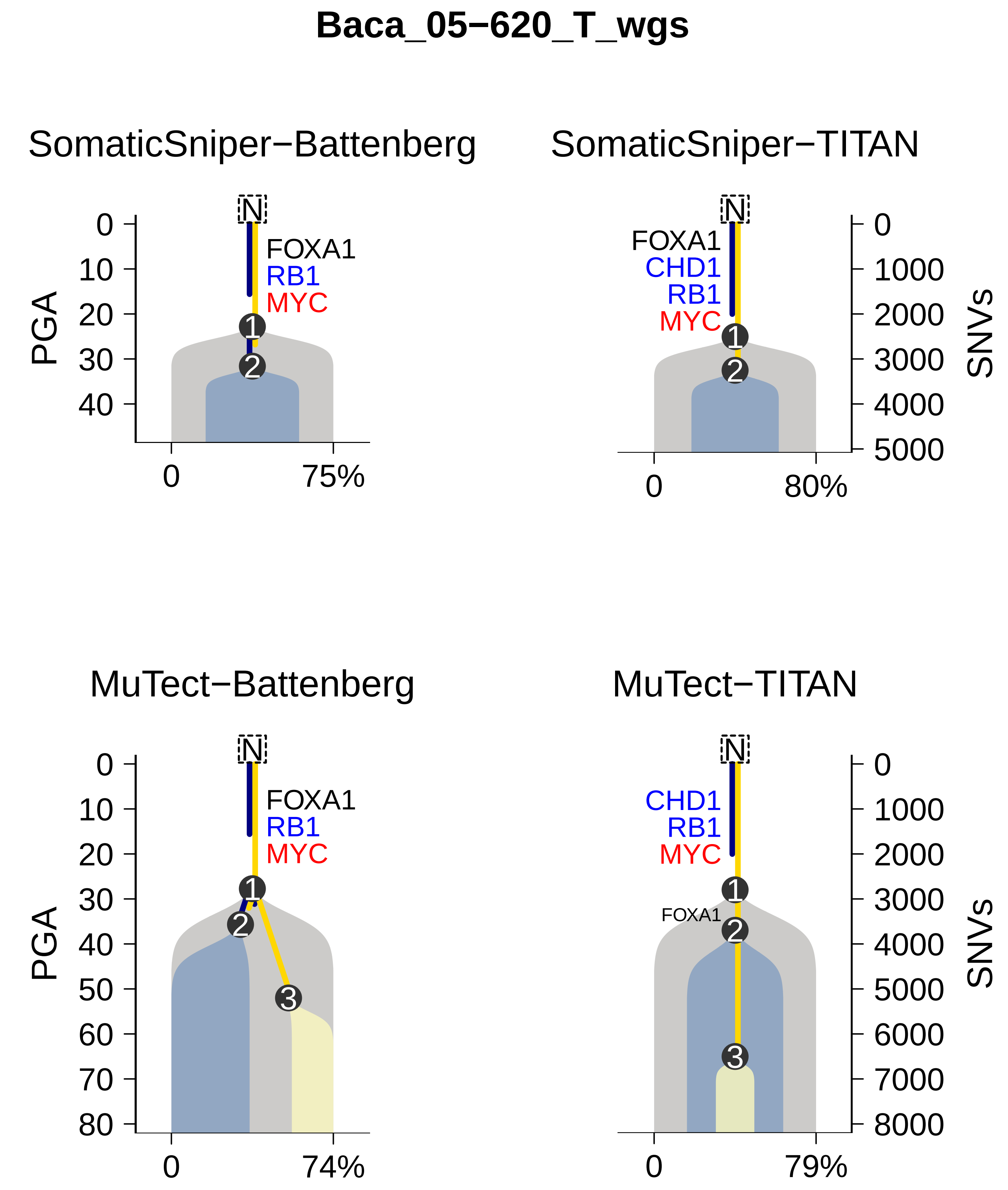
Baca_05−620_T_wgs
SomaticSniper−Battenberg
N
FOXA1
RB1
MYC
1
2
PGA
0
10
20
30
40
0
75%
SomaticSniper−TITAN
N
FOXA1
CHD1
RB1
MYC
1
2
0
1000
2000
3000
4000
5000
SNVs
0
80%
MuTect−Battenberg
N
FOXA1
RB1
MYC
1
2
3
PGA
0
10
20
30
40
50
60
70
80
0
74%
MuTect−TITAN
N
CHD1
RB1
MYC
1
FOXA1
2
3
0
1000
2000
3000
4000
5000
6000
7000
8000
SNVs
0
79%

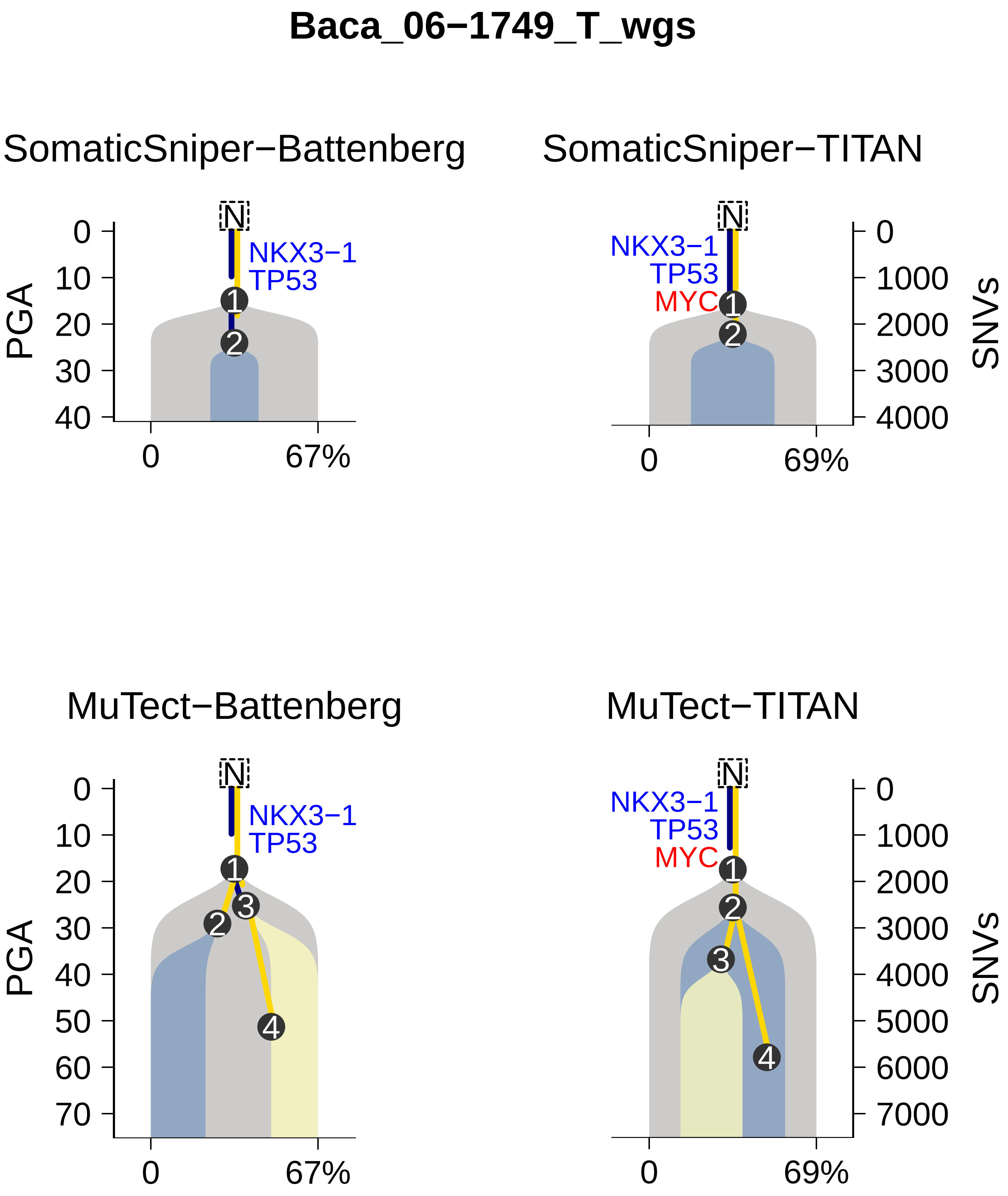

Baca_06−1749_T_wgs
SomaticSniper−Battenberg
SomaticSniper−TITAN
MuTect−Battenberg
MuTect−TITAN
N
NKX3−1
TP53
MYC
PGA
SNVs
0
10
20
30
40
50
60
70
0
1000
2000
3000
4000
5000
6000
7000
67%
69%

Baca_06−3199_T_wgs

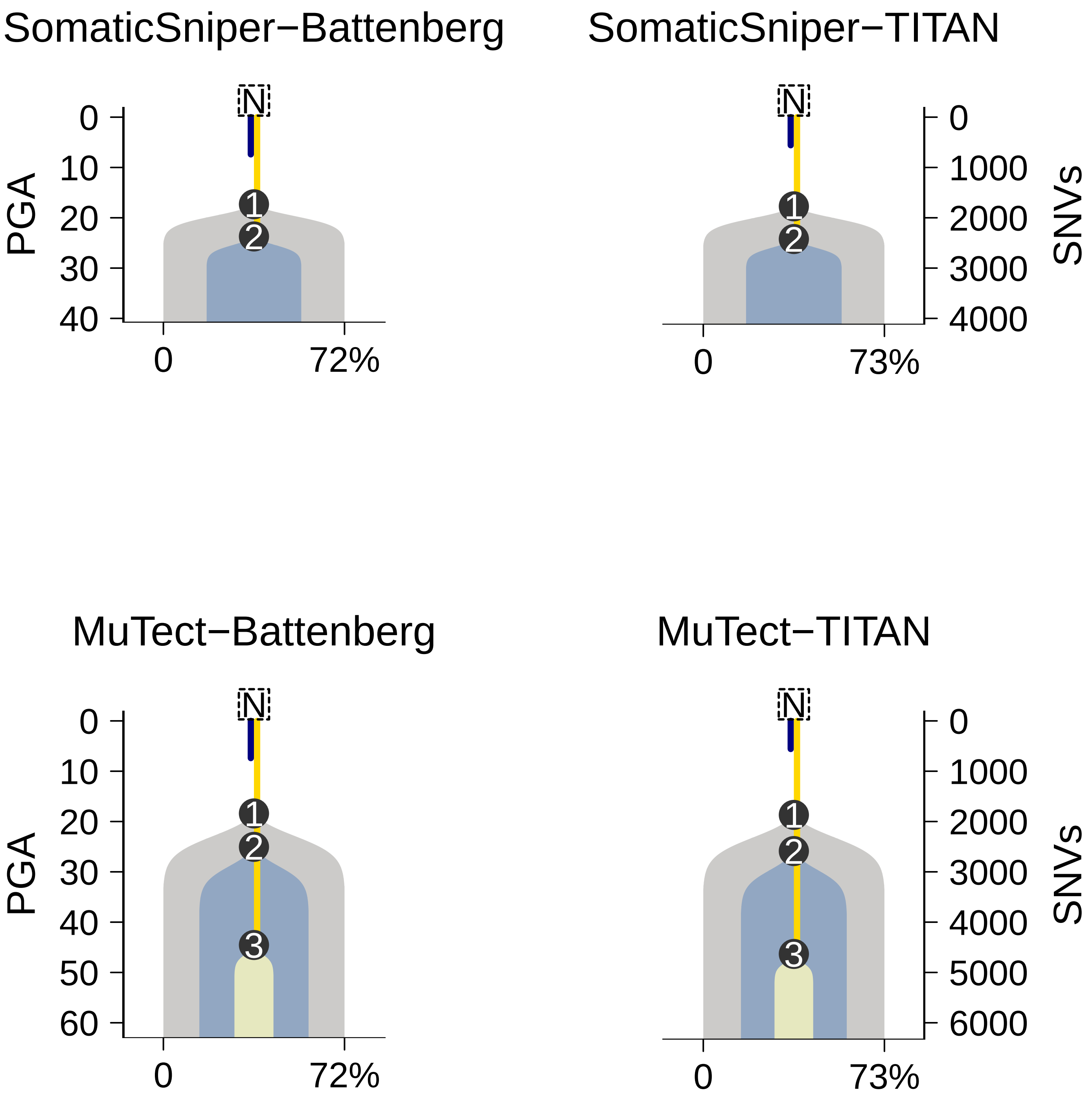

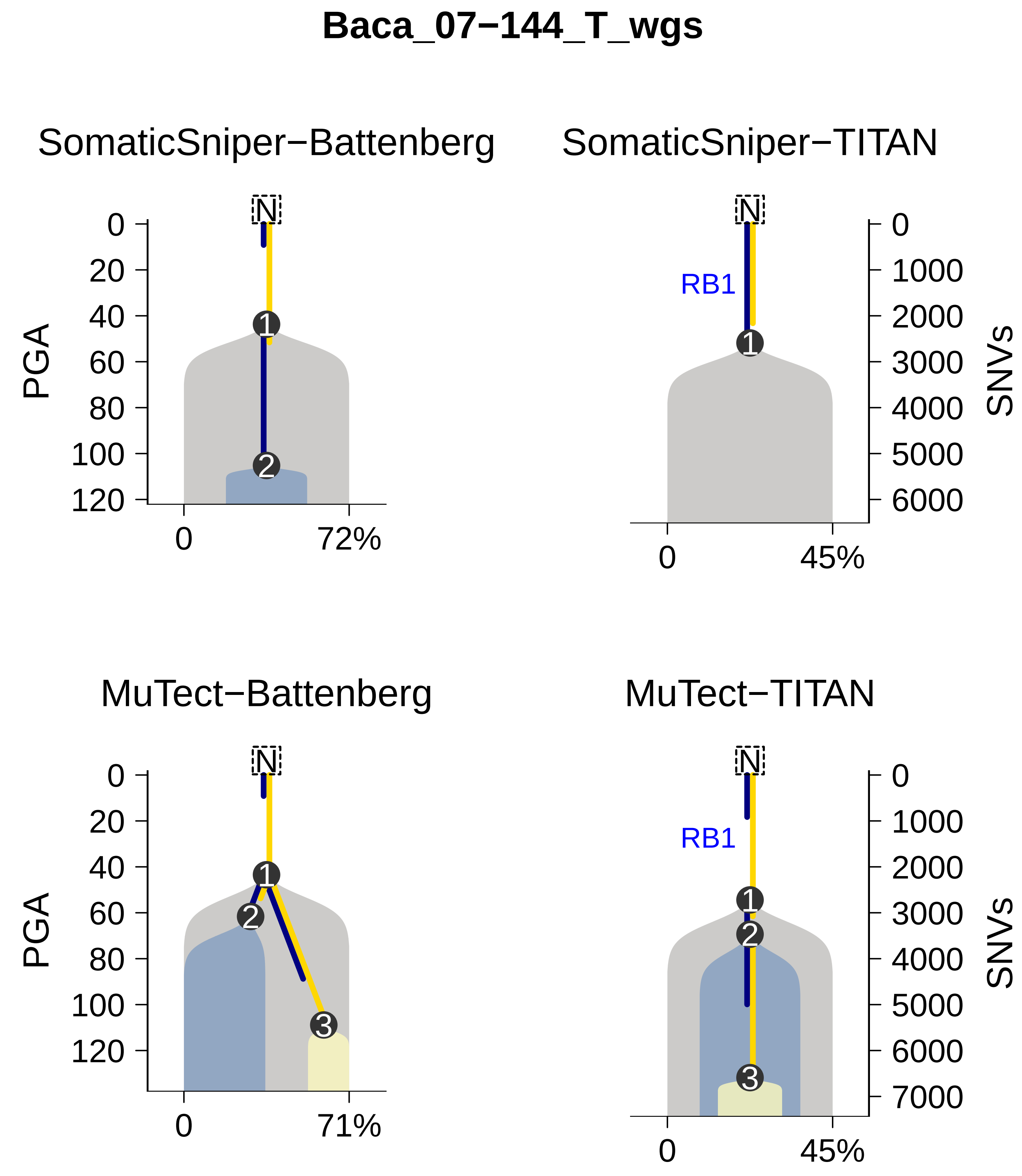
Baca_07−144_T_wgs
SomaticSniper−Battenberg
SomaticSniper−TITAN
MuTect−Battenberg
MuTect−TITAN
N
1
2
3
RB1
PGA
SNVs
0
20
40
60
80
100
120
1000
2000
3000
4000
5000
6000
7000
72%
45%
71%

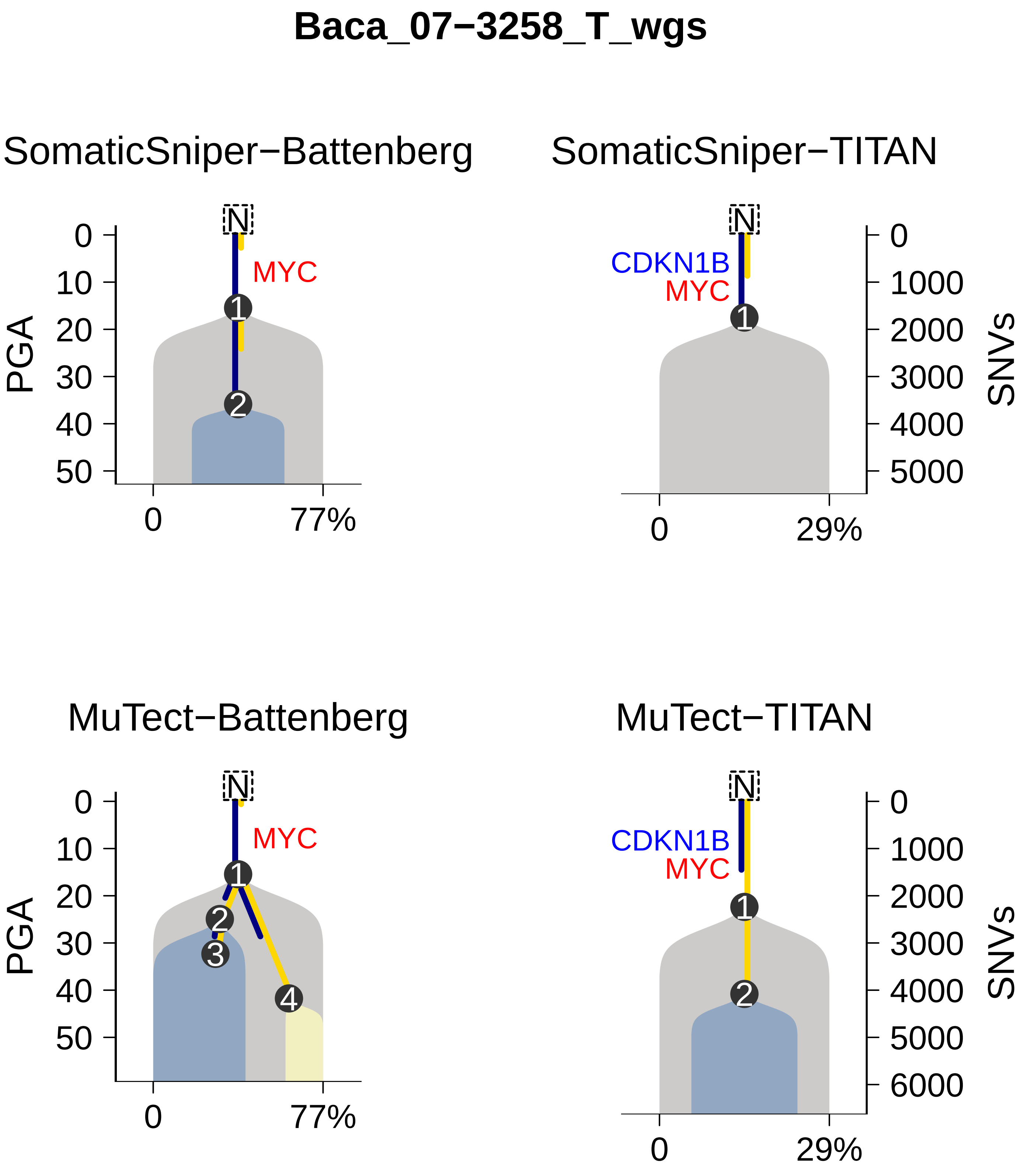
Baca_07−3258_T_wgs
SomaticSniper−Battenberg
SomaticSniper−TITAN
MuTect−Battenberg
MuTect−TITAN
PGA
SNVs
N
MYC
CDKN1B
0
10
20
30
40
50
0
1000
2000
3000
4000
5000
6000
77%
29%

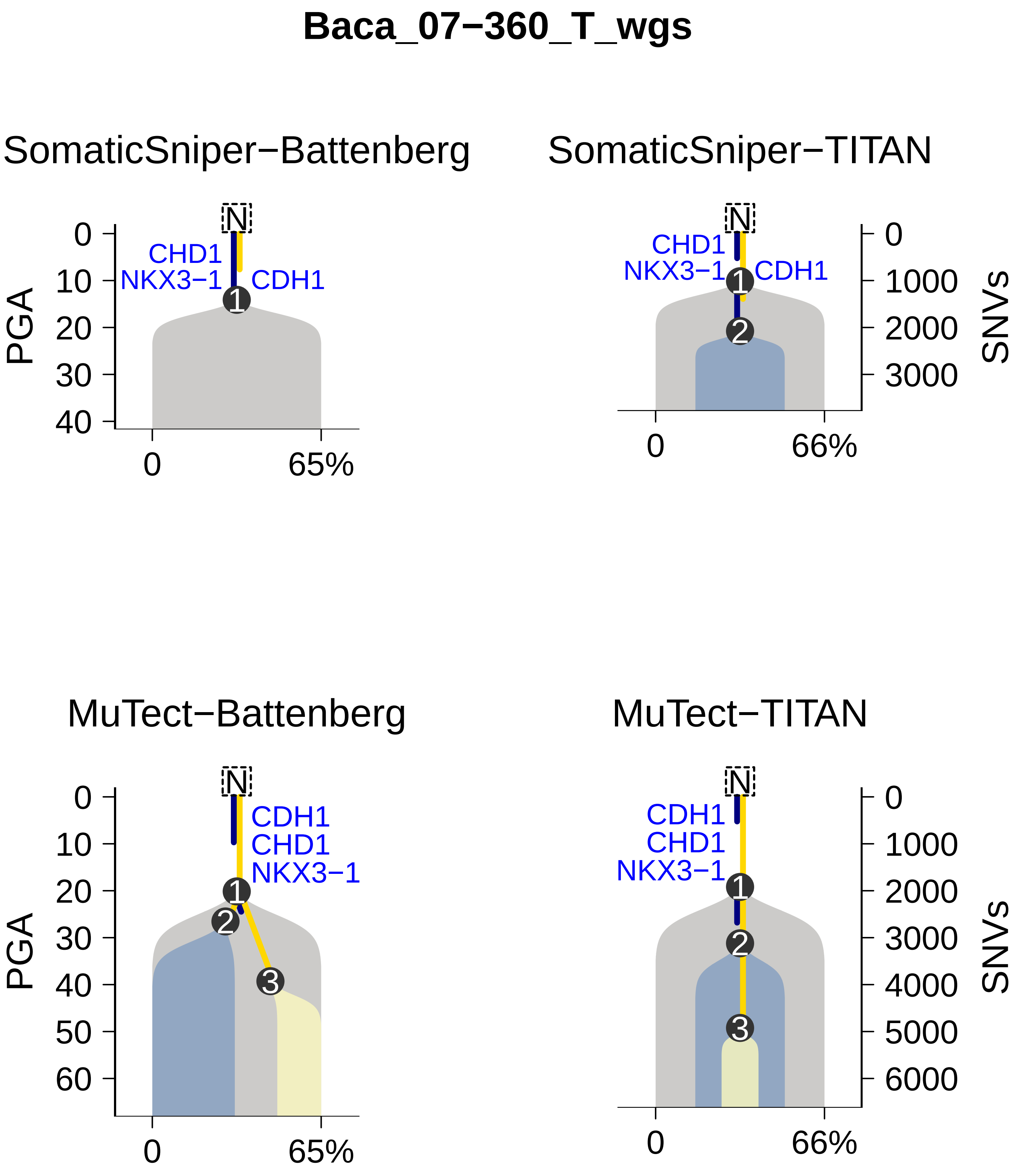
Baca_07−360_T_wgs
SomaticSniper−Battenberg
SomaticSniper−TITAN
MuTect−Battenberg
MuTect−TITAN
N
CHD1
NKX3−1
CDH1
PGA
SNVs
0
10
20
30
40
50
60
1000
2000
3000
4000
5000
6000
65%
66%
1
2
3

Baca_07−4610_T_wgs

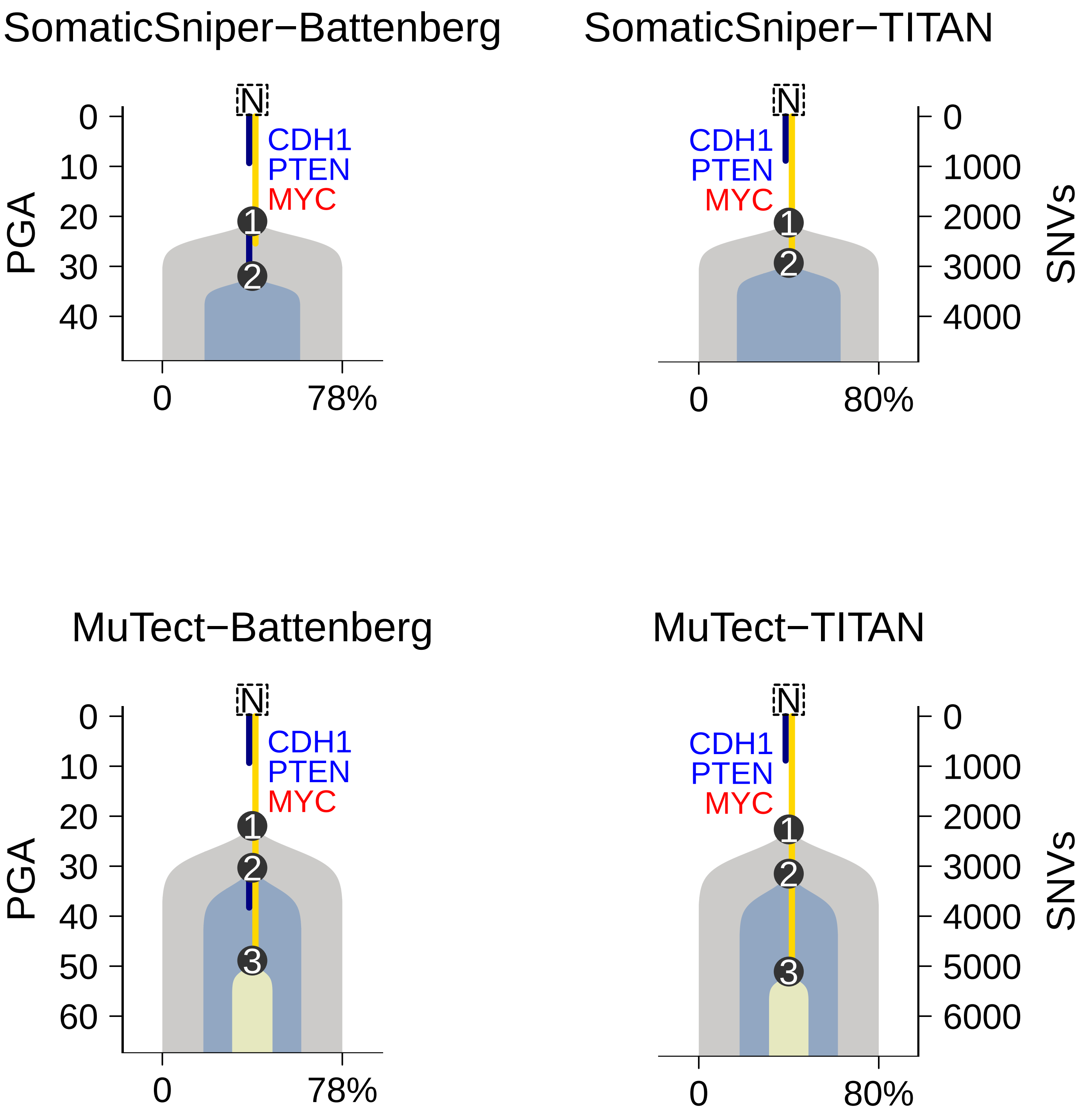

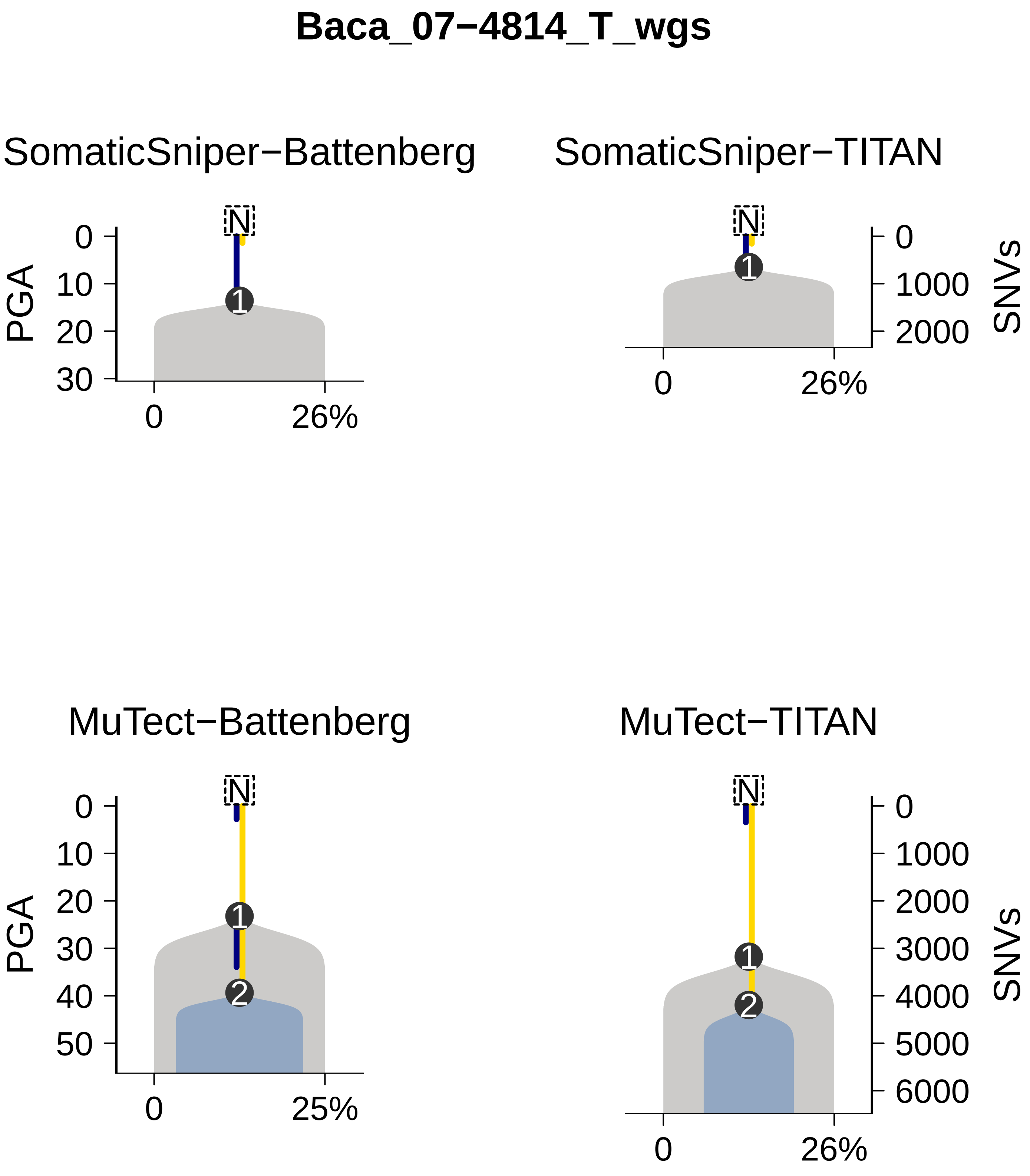

Baca_07−4814_T_wgs
SomaticSniper−Battenberg
SomaticSniper−TITAN
N
1
PGA
0
10
20
30
0
26%
SNVs
1000
2000
MuTect−Battenberg
MuTect−TITAN
2
40
50
25%
3000
4000
5000
6000

Baca_07−4941_T_wgs

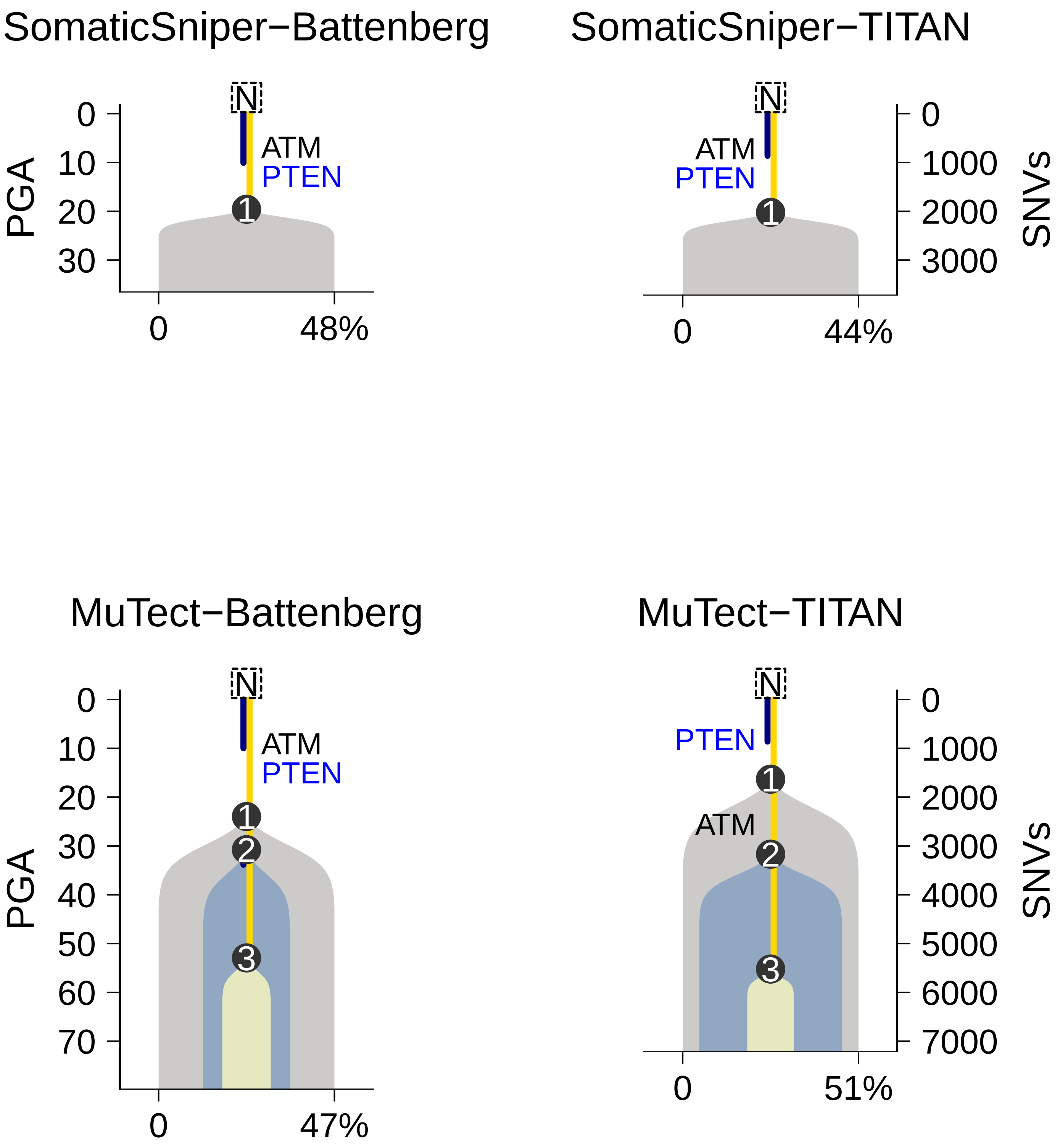

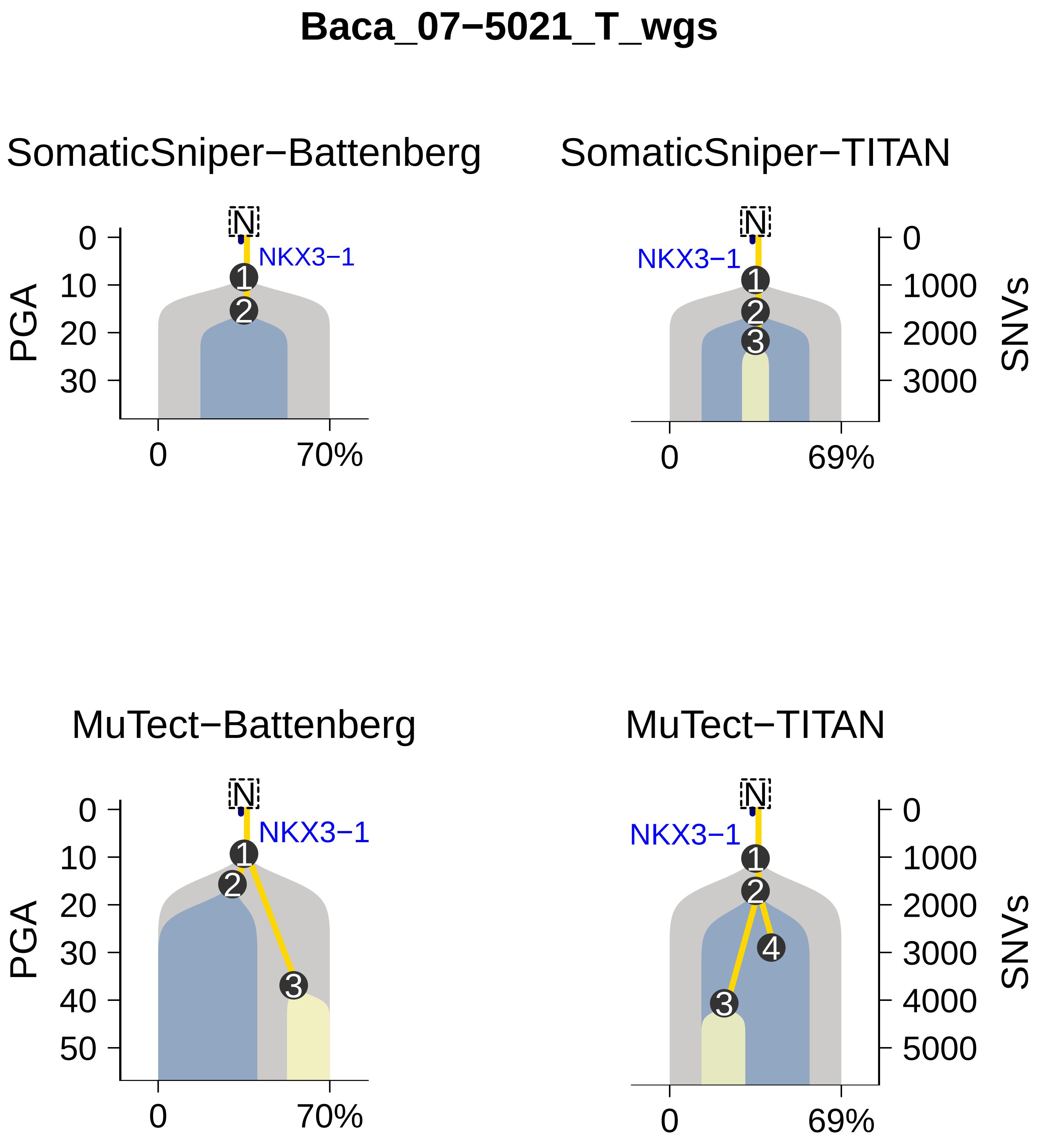
Baca_07−5021_T_wgs
SomaticSniper−Battenberg
SomaticSniper−TITAN
MuTect−Battenberg
MuTect−TITAN
N
NKX3−1
1
2
3
4
PGA
SNVs
0
10
20
30
40
50
1000
2000
3000
4000
5000
70%
69%

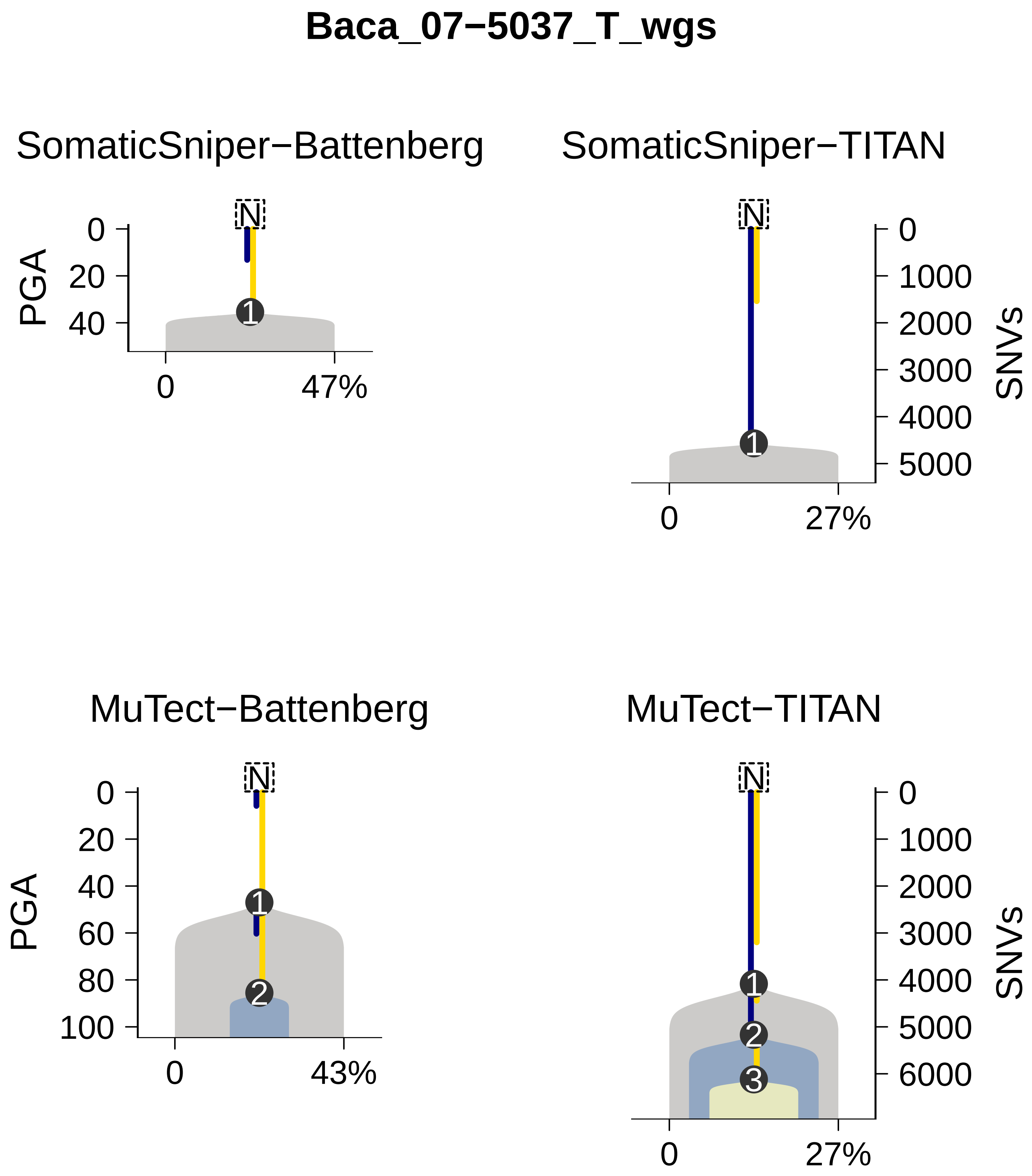

Baca_07−5037_T_wgs
SomaticSniper−Battenberg
SomaticSniper−TITAN
MuTect−Battenberg
MuTect−TITAN
N
1
2
3
PGA
SNVs
0
20
40
60
80
100
1000
2000
3000
4000
5000
6000
47%
27%
43%

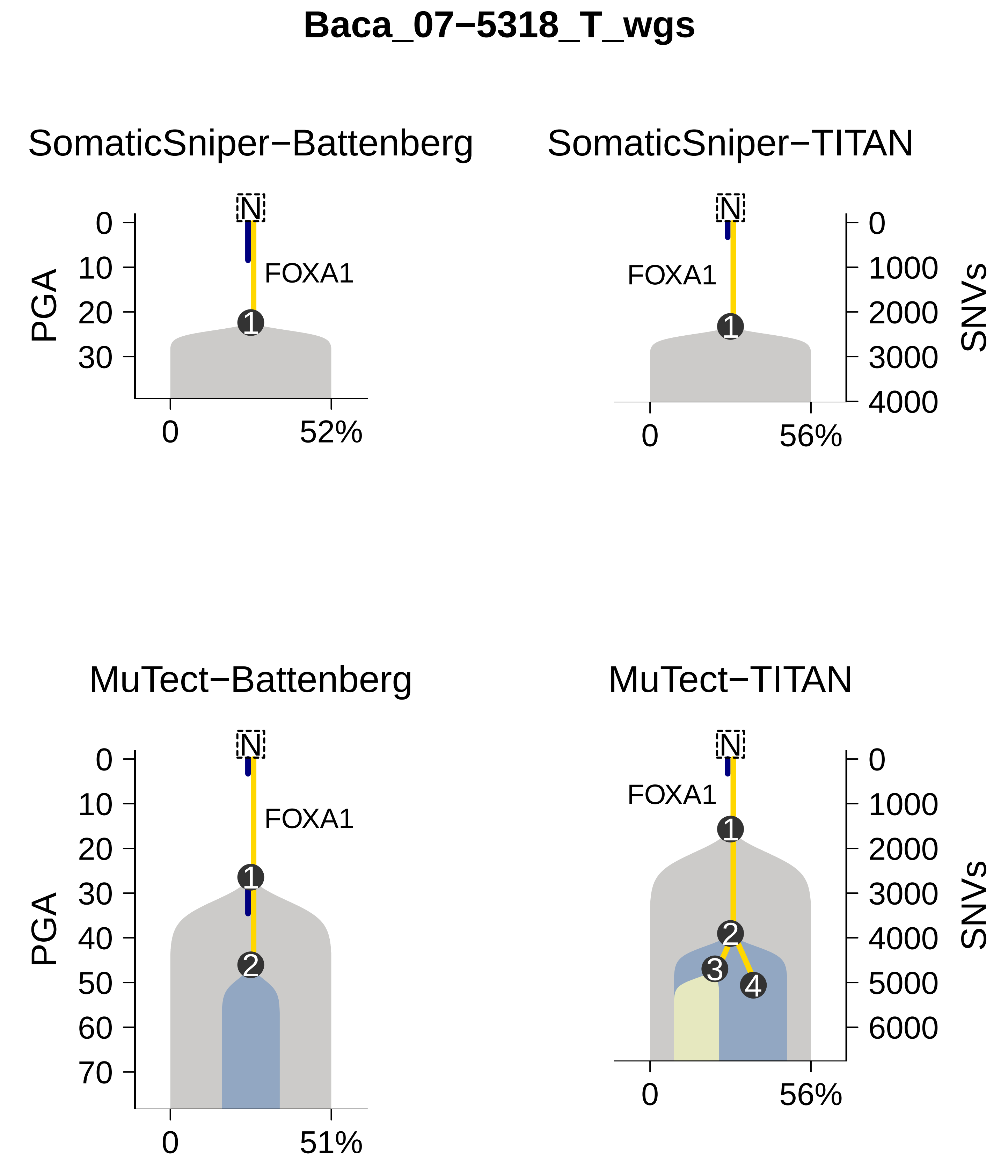

Baca_07−5318_T_wgs
SomaticSniper−Battenberg
SomaticSniper−TITAN
MuTect−Battenberg
MuTect−TITAN
N
FOXA1
PGA
SNVs
0
10
20
30
40
50
60
70
1000
2000
3000
4000
5000
6000
52%
56%
51%
1
2
3
4

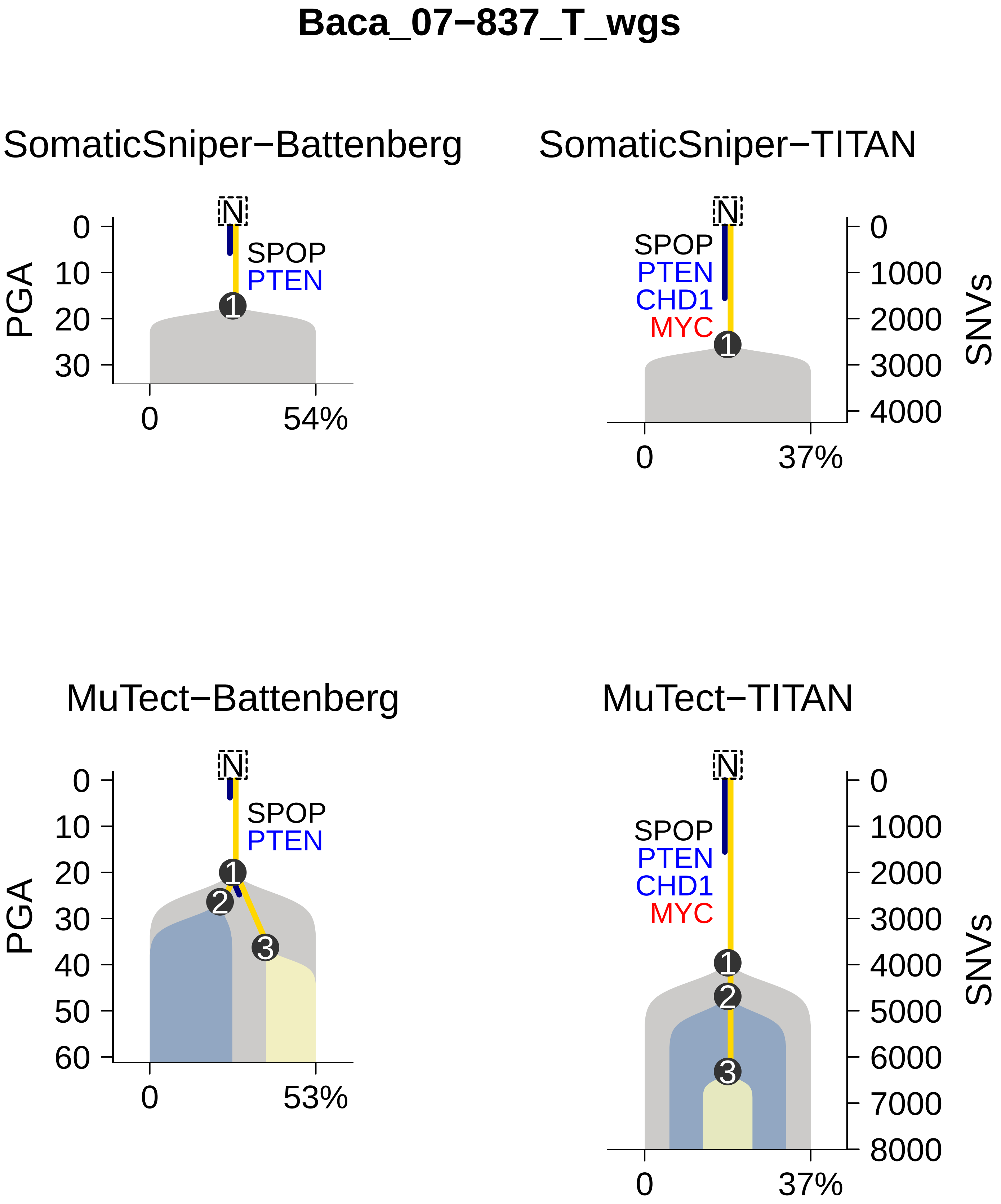
Baca_07−837_T_wgs
SomaticSniper−Battenberg
N
SPOP
PTEN
1
PGA
0
10
20
30
0
54%
SomaticSniper−TITAN
N
SPOP
PTEN
CHD1
MYC
1
0
1000
2000
3000
4000
SNVs
0
37%
MuTect−Battenberg
N
SPOP
PTEN
1
2
3
PGA
0
10
20
30
40
50
60
0
53%
MuTect−TITAN
N
SPOP
PTEN
CHD1
MYC
1
2
3
0
1000
2000
3000
4000
5000
6000
7000
8000
SNVs
0
37%

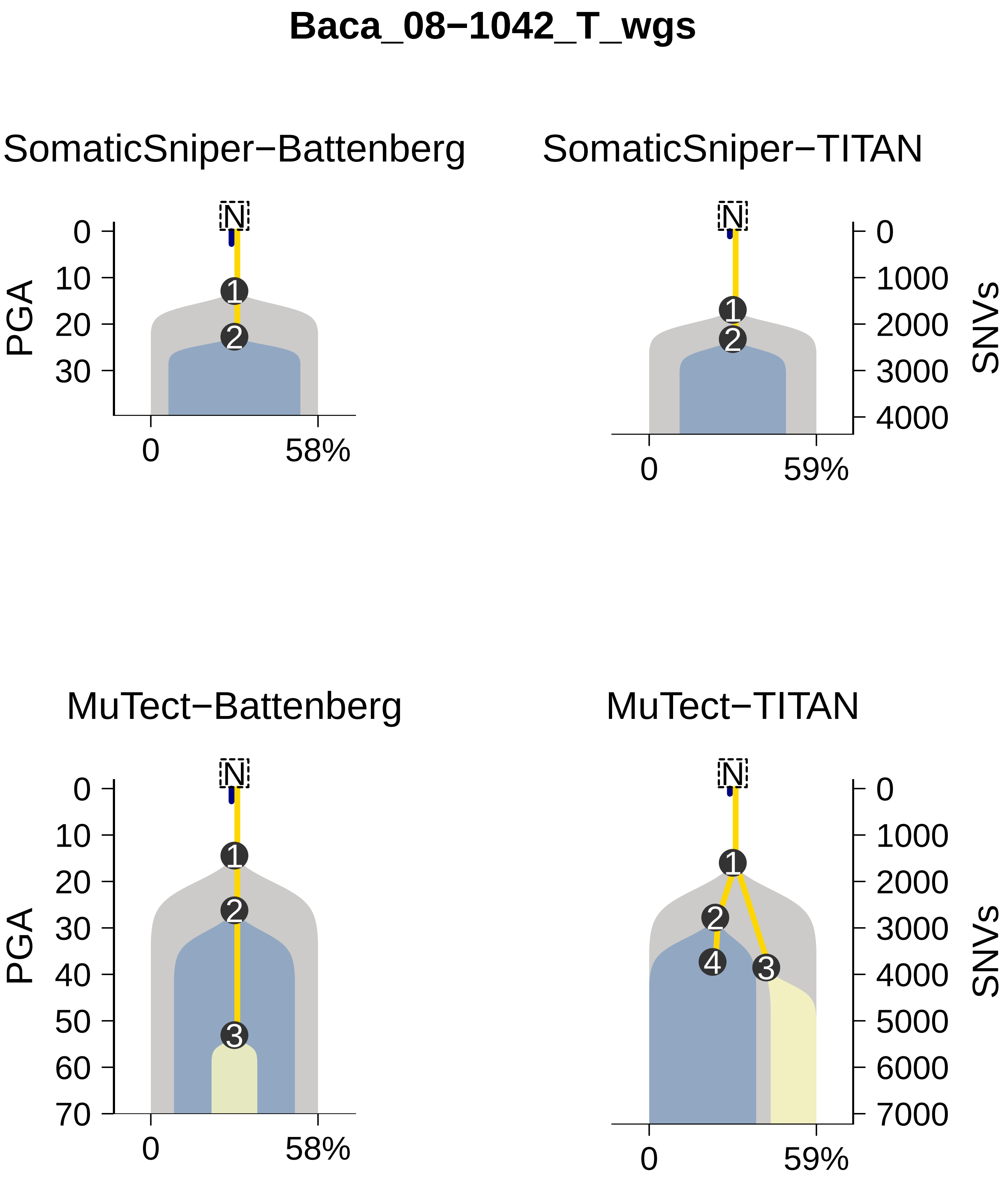
Baca_08−1042_T_wgs
SomaticSniper−Battenberg
SomaticSniper−TITAN
MuTect−Battenberg
MuTect−TITAN
N
1
2
3
4
PGA
0
10
20
30
40
50
60
70
SNVs
0
1000
2000
3000
4000
5000
6000
7000
0
58%
59%

Baca_08−1541_T_wgs

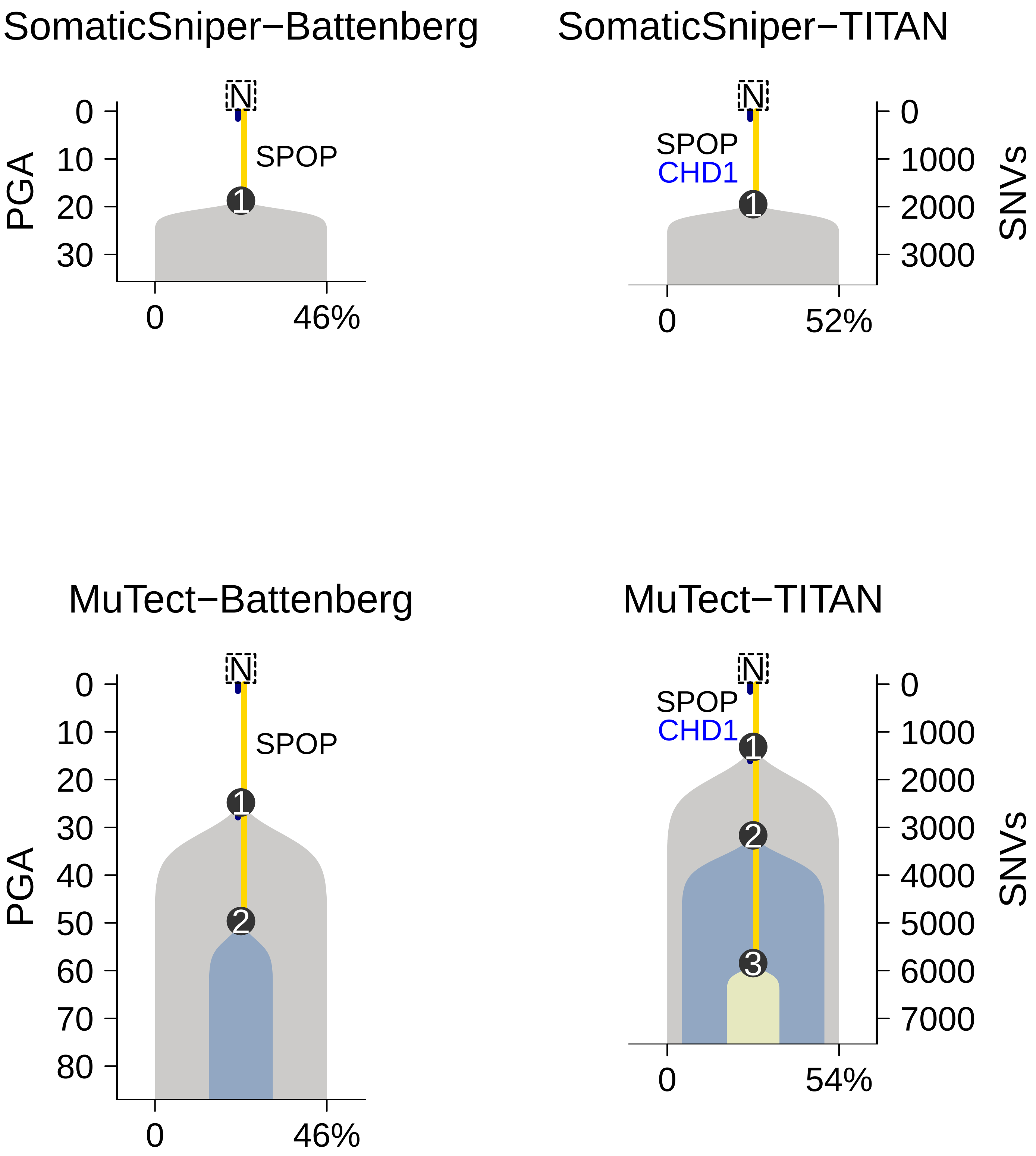

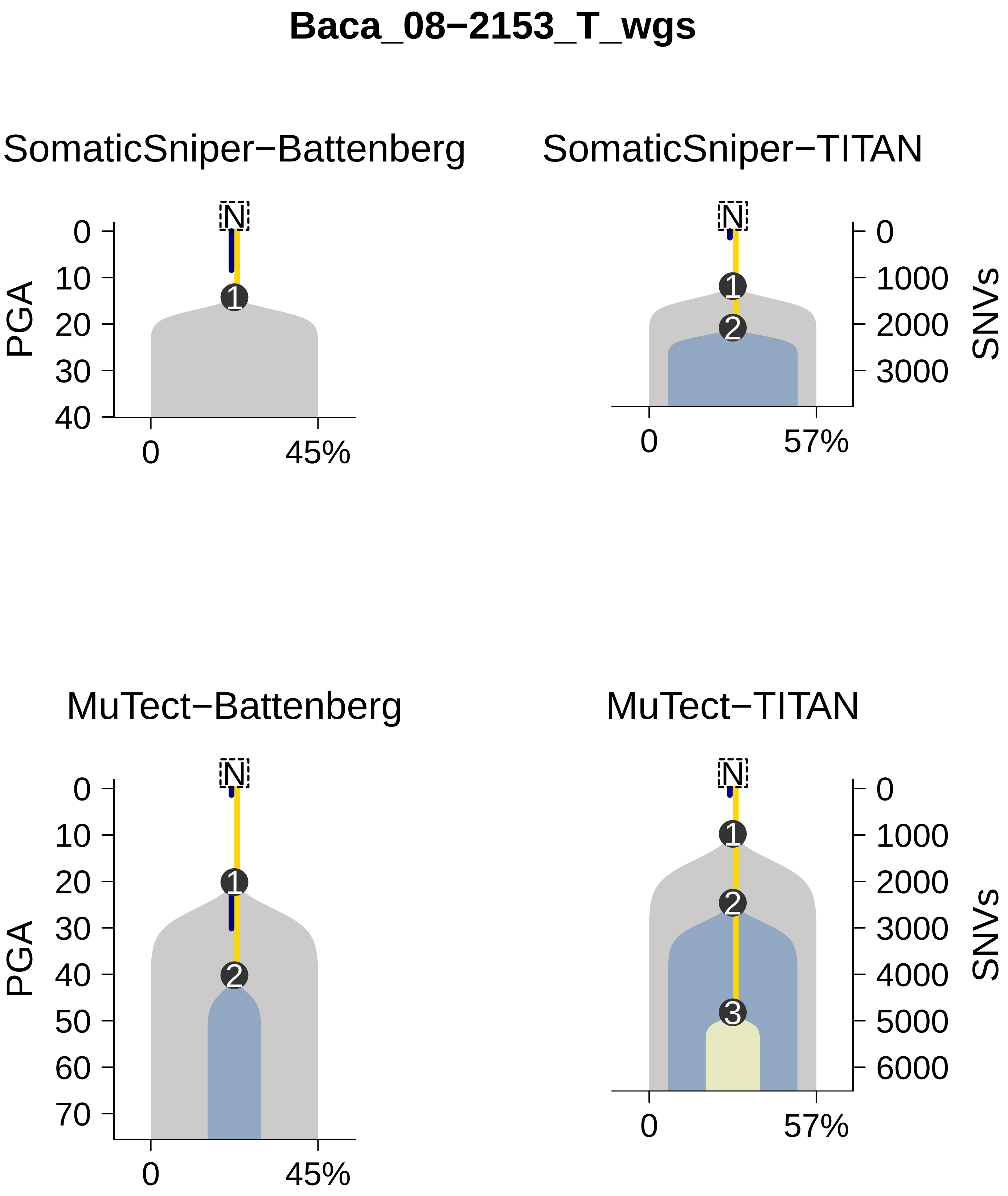
Baca_08−2153_T_wgs
SomaticSniper−Battenberg
SomaticSniper−TITAN
MuTect−Battenberg
MuTect−TITAN
N
1
2
3
PGA
SNVs
0
10
20
30
40
50
60
70
1000
2000
3000
4000
5000
6000
45%
57%

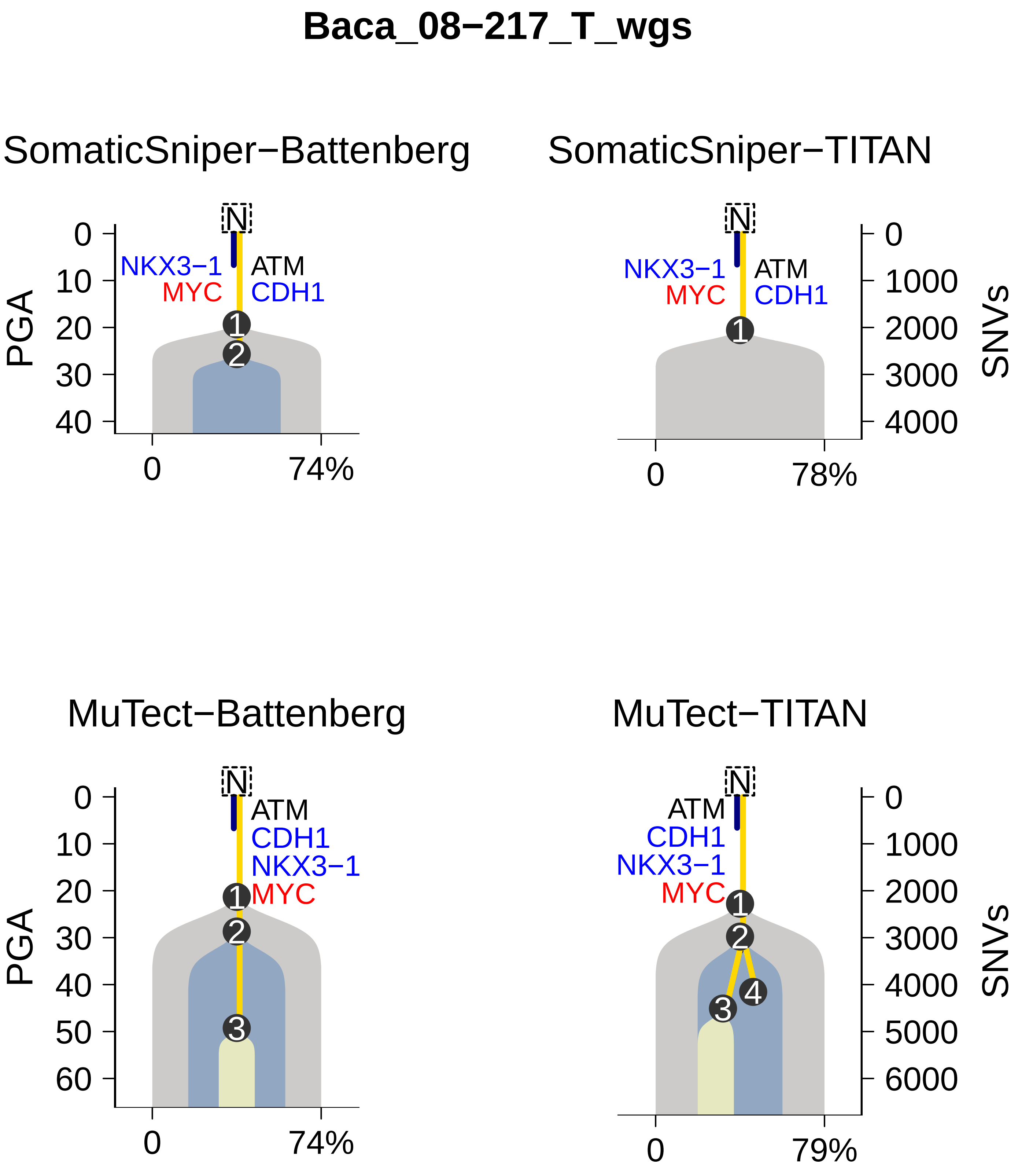

Baca_08−217_T_wgs
SomaticSniper−Battenberg
SomaticSniper−TITAN
MuTect−Battenberg
MuTect−TITAN
N
NKX3−1
MYC
ATM
CDH1
PGA
SNVs
0
10
20
30
40
50
60
1000
2000
3000
4000
5000
6000
74%
78%
79%
1
2
3
4

Baca_08−2455_T_wgs

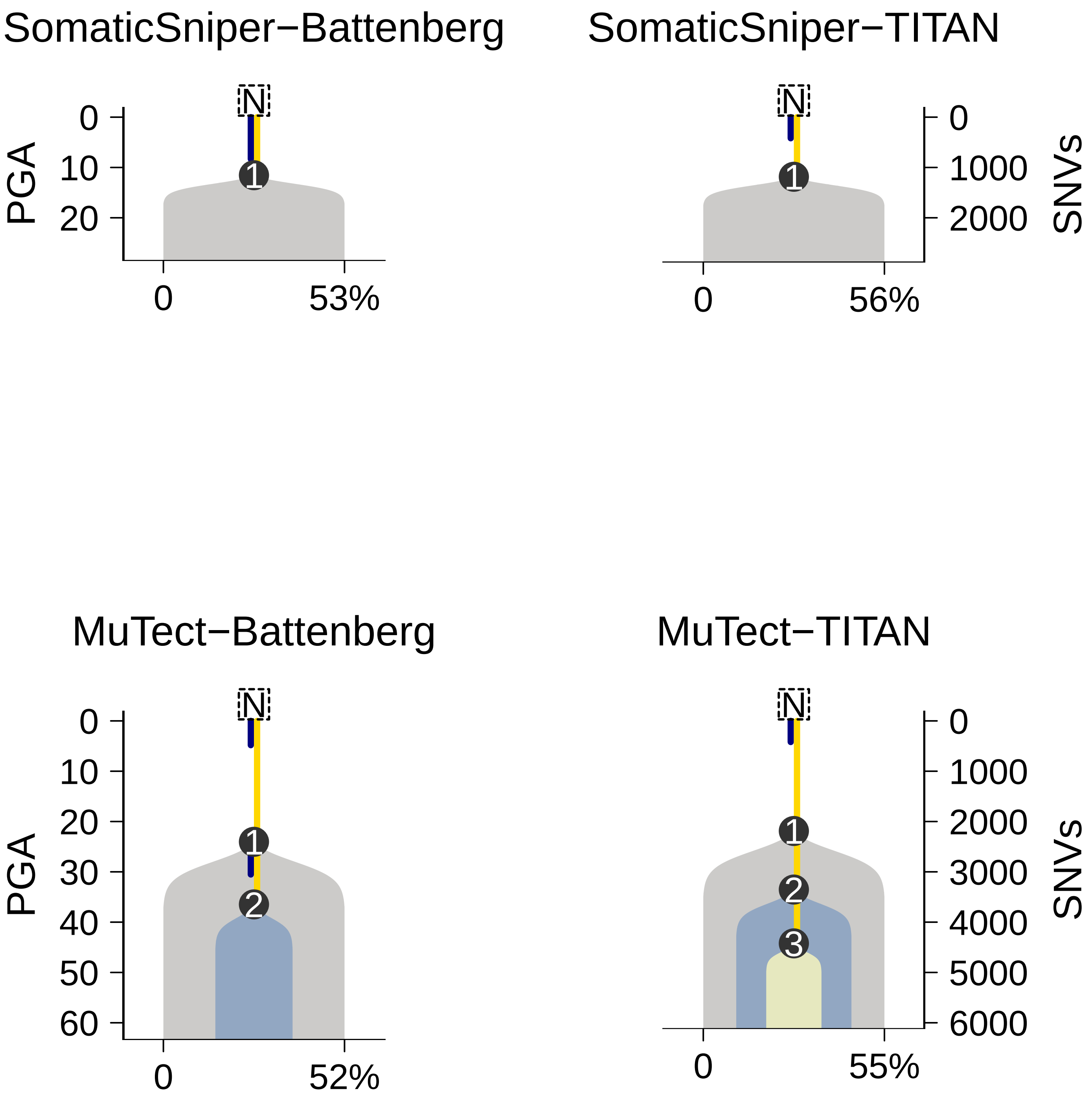

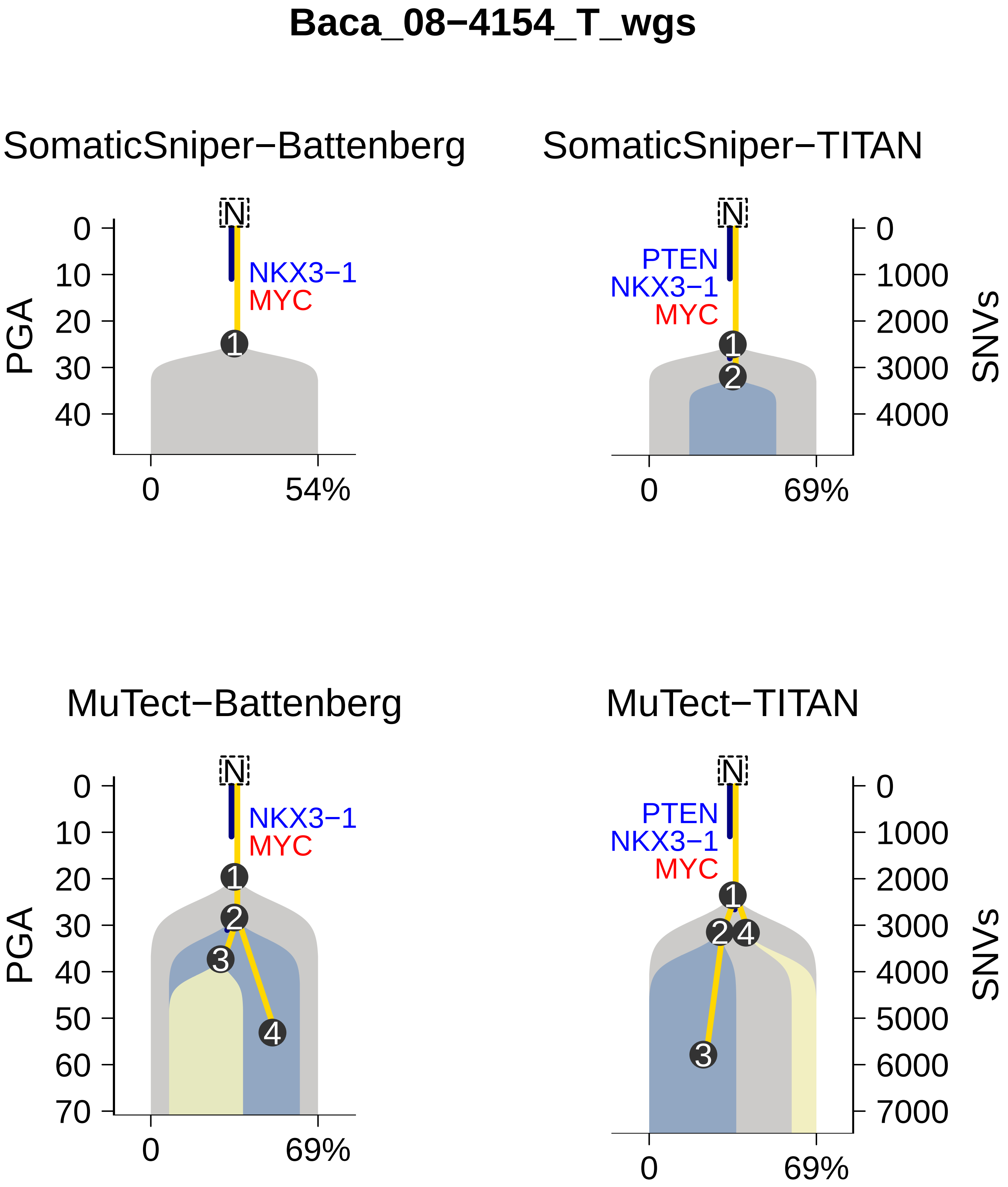
Baca_08−4154_T_wgs
SomaticSniper−Battenberg
SomaticSniper−TITAN
MuTect−Battenberg
MuTect−TITAN
N
NKX3−1
MYC
PTEN
PGA
SNVs
0
10
20
30
40
50
60
70
1000
2000
3000
4000
5000
6000
7000
54%
69%
1
2
3
4

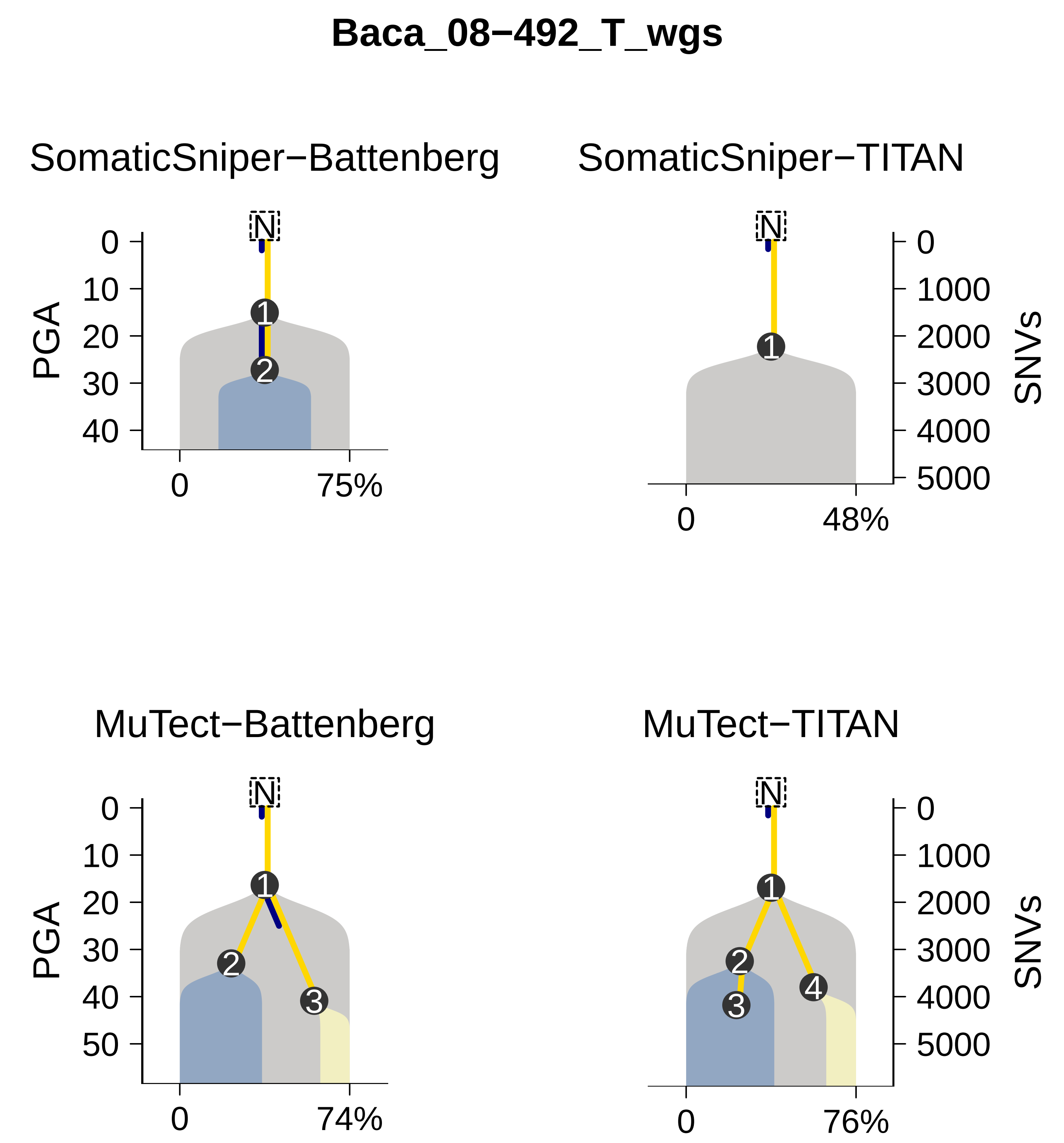
Baca_08−492_T_wgs
SomaticSniper−Battenberg
SomaticSniper−TITAN
MuTect−Battenberg
MuTect−TITAN
PGA
SNVs
N
0
10
20
30
40
50
1000
2000
3000
4000
5000
75%
48%
74%
76%

Baca_08−501_T_wgs

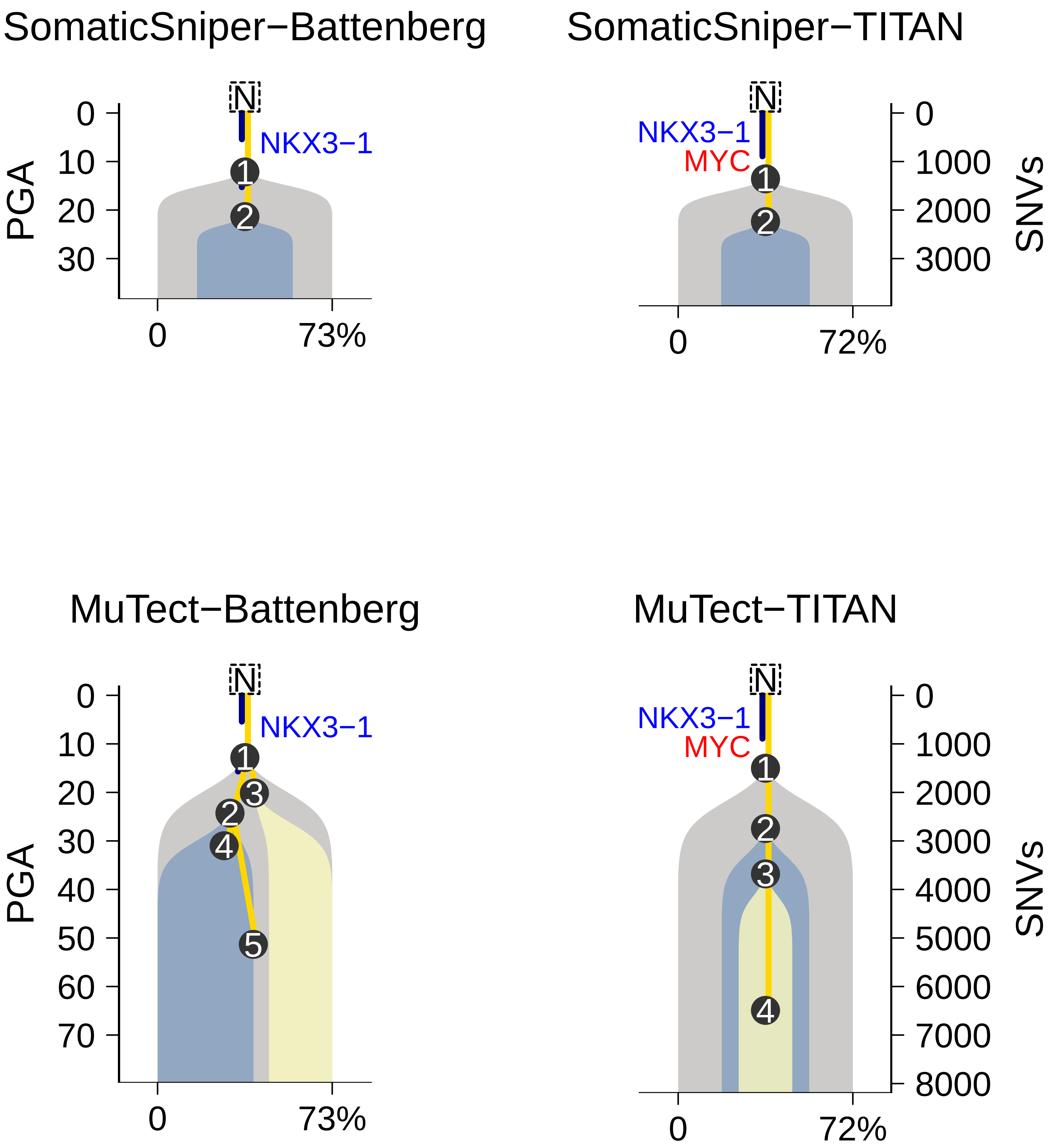

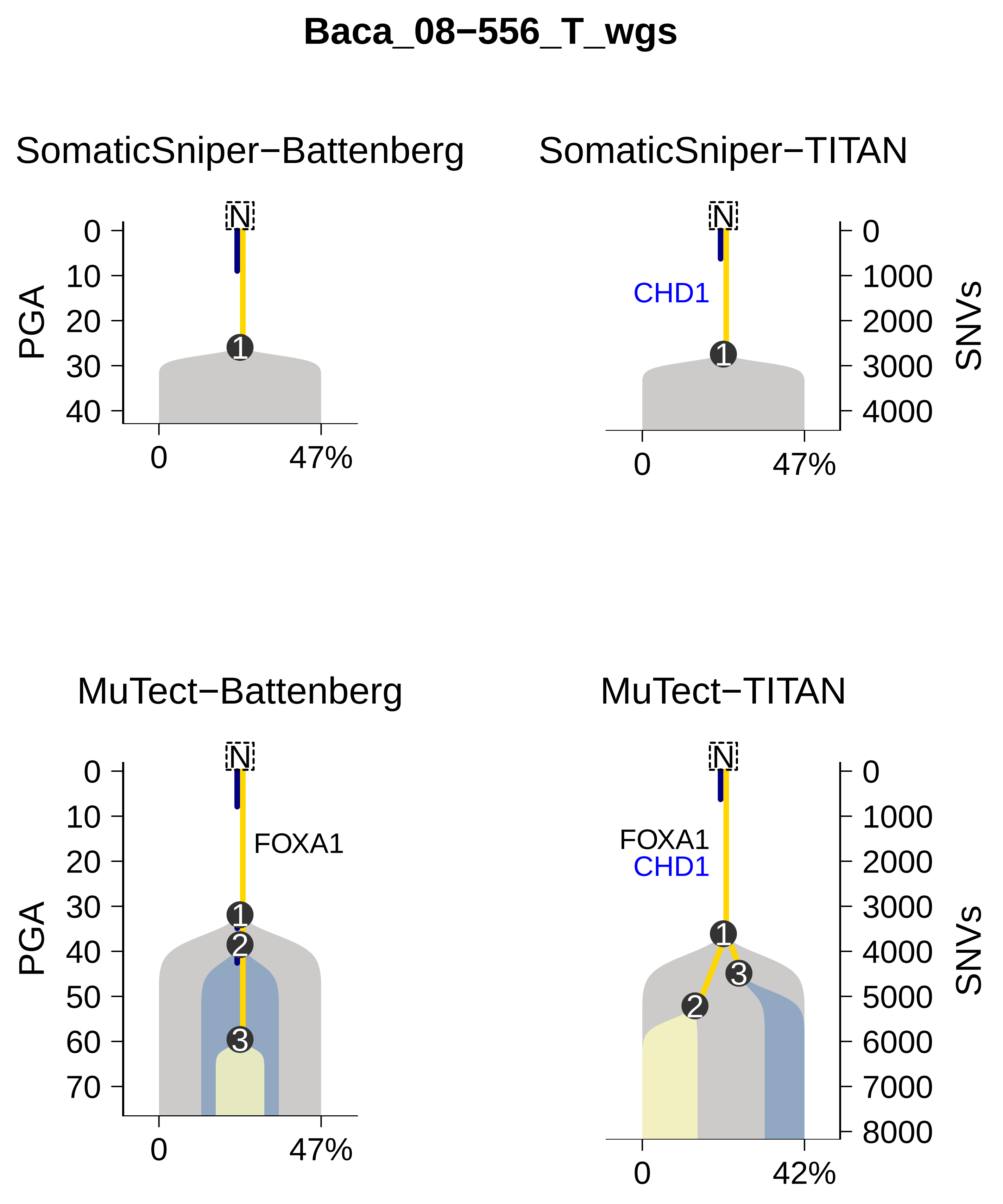
Baca_08−556_T_wgs
SomaticSniper−Battenberg
SomaticSniper−TITAN
MuTect−Battenberg
MuTect−TITAN
N
1
2
3
PGA
SNVs
CHD1
FOXA1
0
10
20
30
40
50
60
70
1000
2000
3000
4000
5000
6000
7000
8000
47%
42%

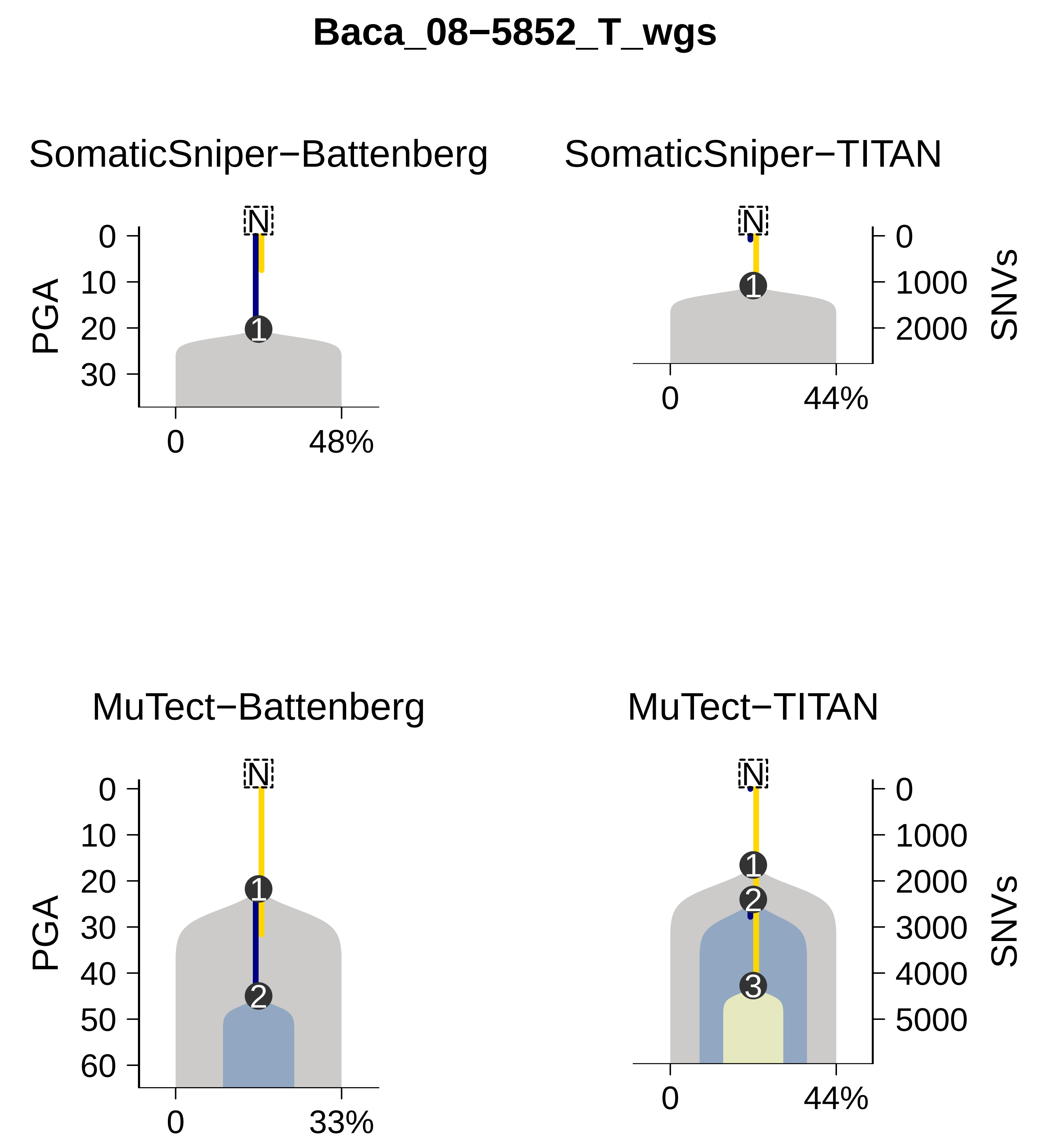
Baca_08−5852_T_wgs
SomaticSniper−Battenberg
SomaticSniper−TITAN
MuTect−Battenberg
MuTect−TITAN
N
1
2
3
PGA
SNVs
0
10
20
30
40
50
60
1000
2000
3000
4000
5000
48%
44%
33%

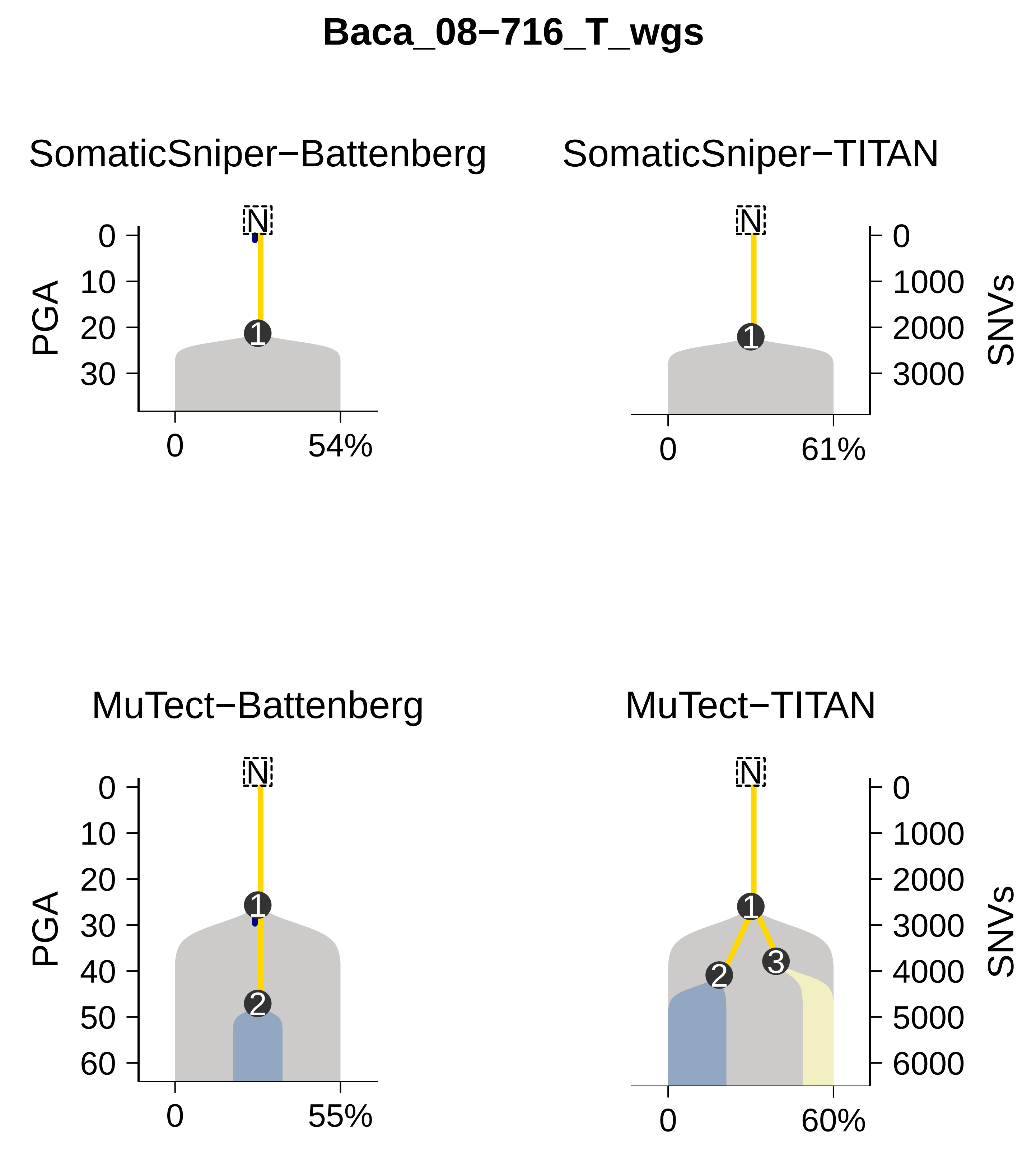
Baca_08−716_T_wgs
SomaticSniper−Battenberg
SomaticSniper−TITAN
MuTect−Battenberg
MuTect−TITAN
PGA
SNVs
N
1
2
3
0
10
20
30
40
50
60
1000
2000
3000
4000
5000
6000
54%
61%
55%
60%

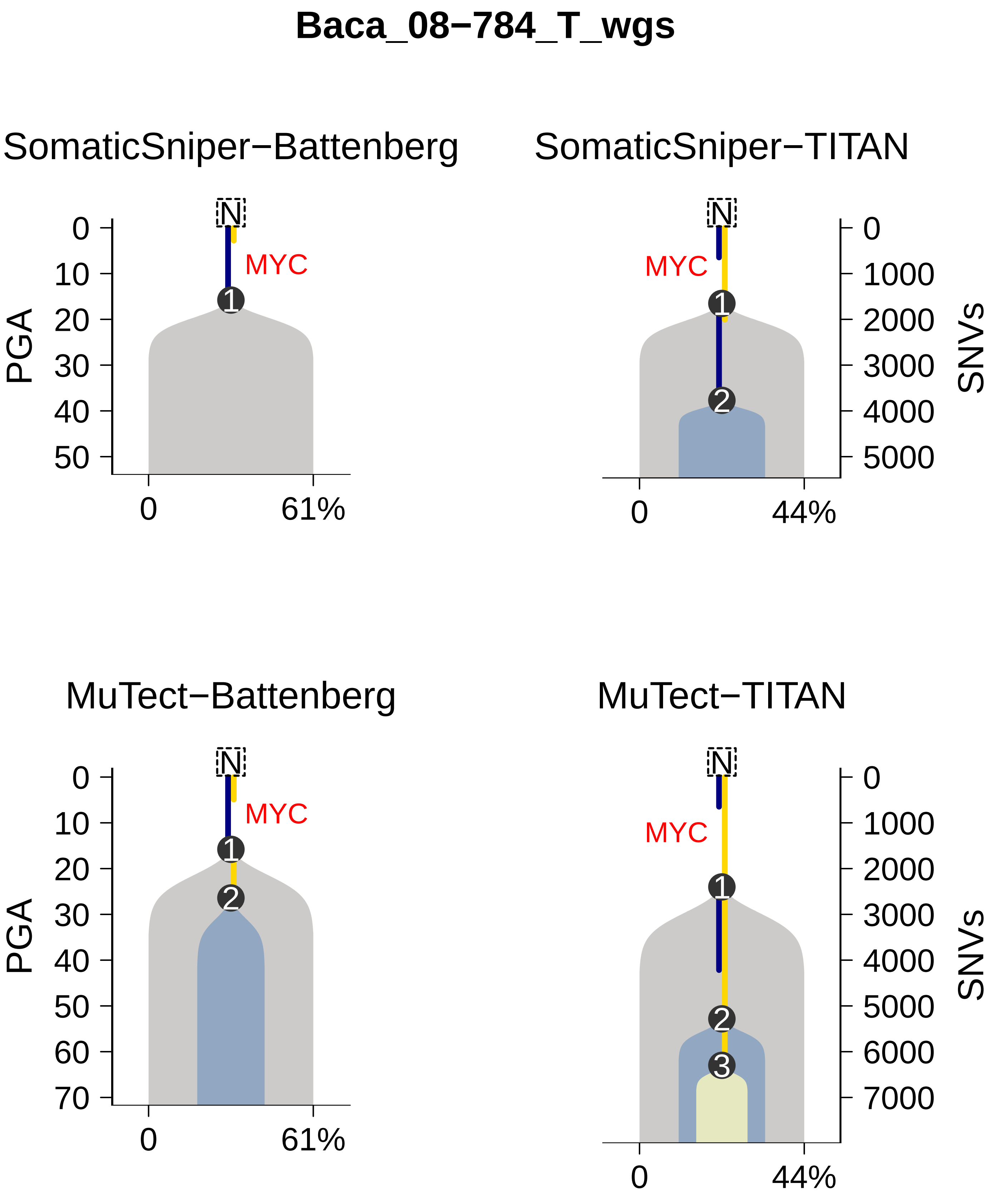
Baca_08−784_T_wgs
SomaticSniper−Battenberg
SomaticSniper−TITAN
MuTect−Battenberg
MuTect−TITAN
N
MYC
PGA
SNVs
0
10
20
30
40
50
60
70
0
1000
2000
3000
4000
5000
6000
7000
0
61%
44%
1
2
3

Baca_09−146_T_wgs

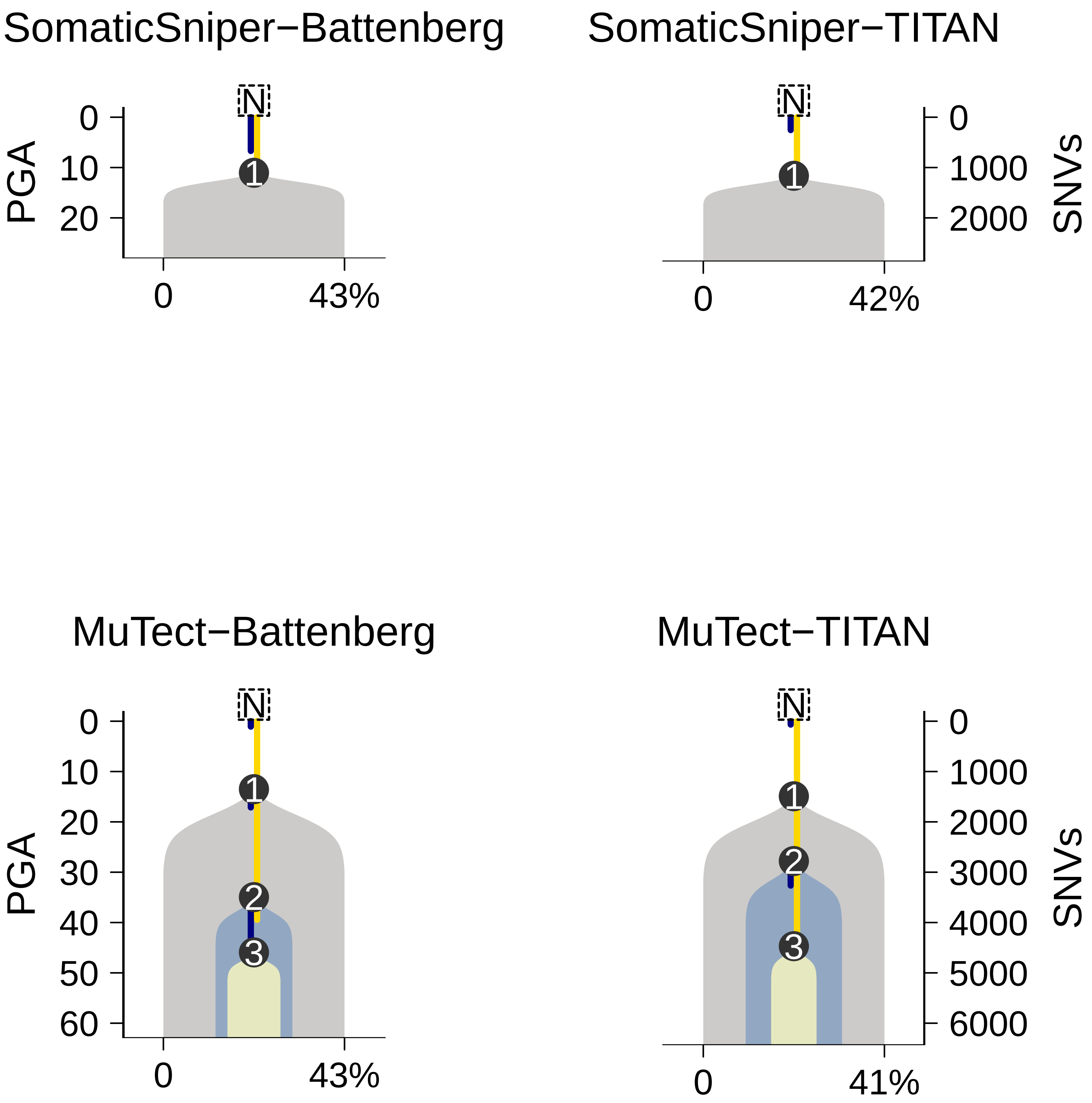

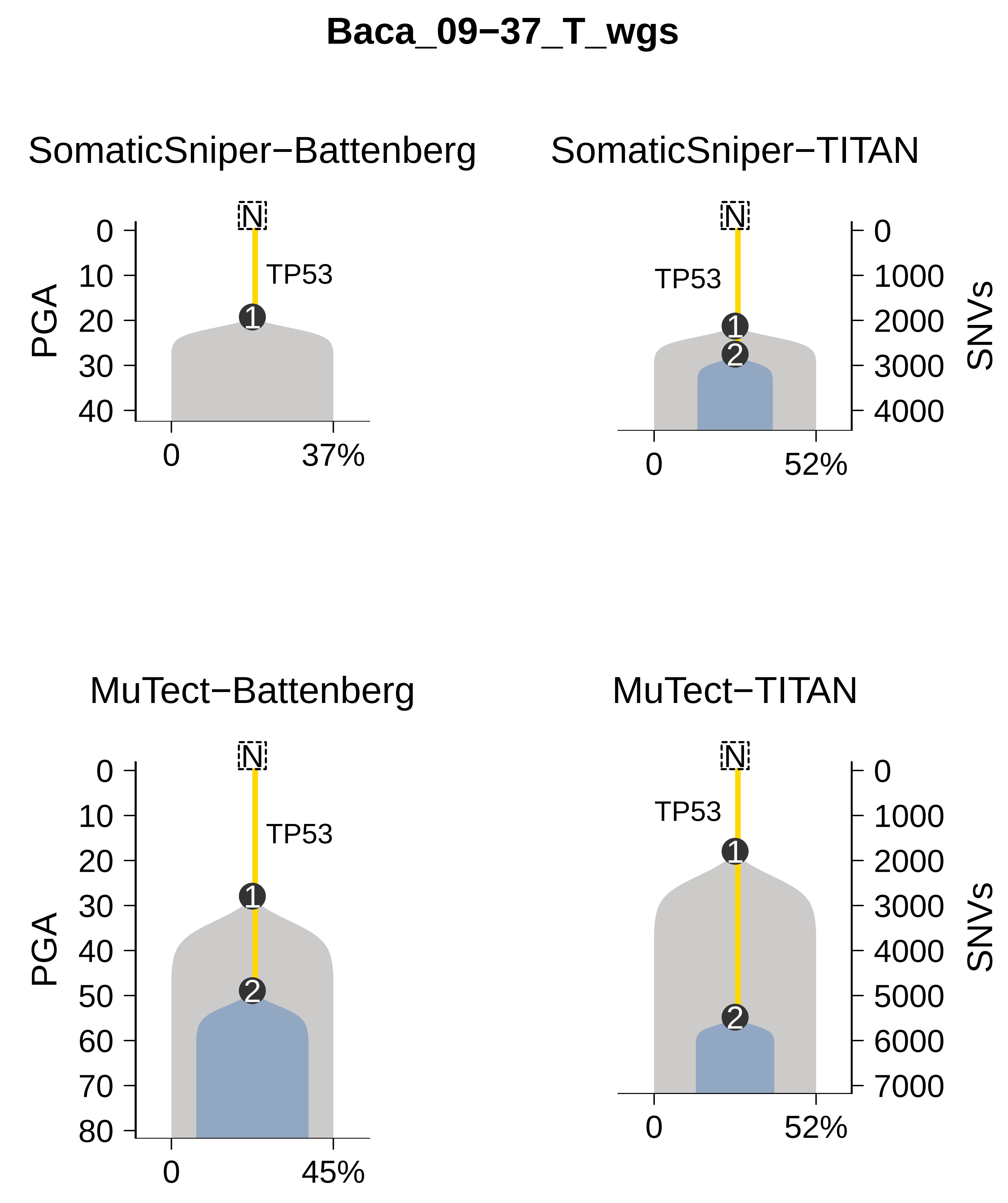
Baca_09−37_T_wgs
SomaticSniper−Battenberg
SomaticSniper−TITAN
MuTect−Battenberg
MuTect−TITAN
N
TP53
1
2
PGA
SNVs
0
10
20
30
40
50
60
70
80
1000
2000
3000
4000
5000
6000
7000
37%
52%
45%

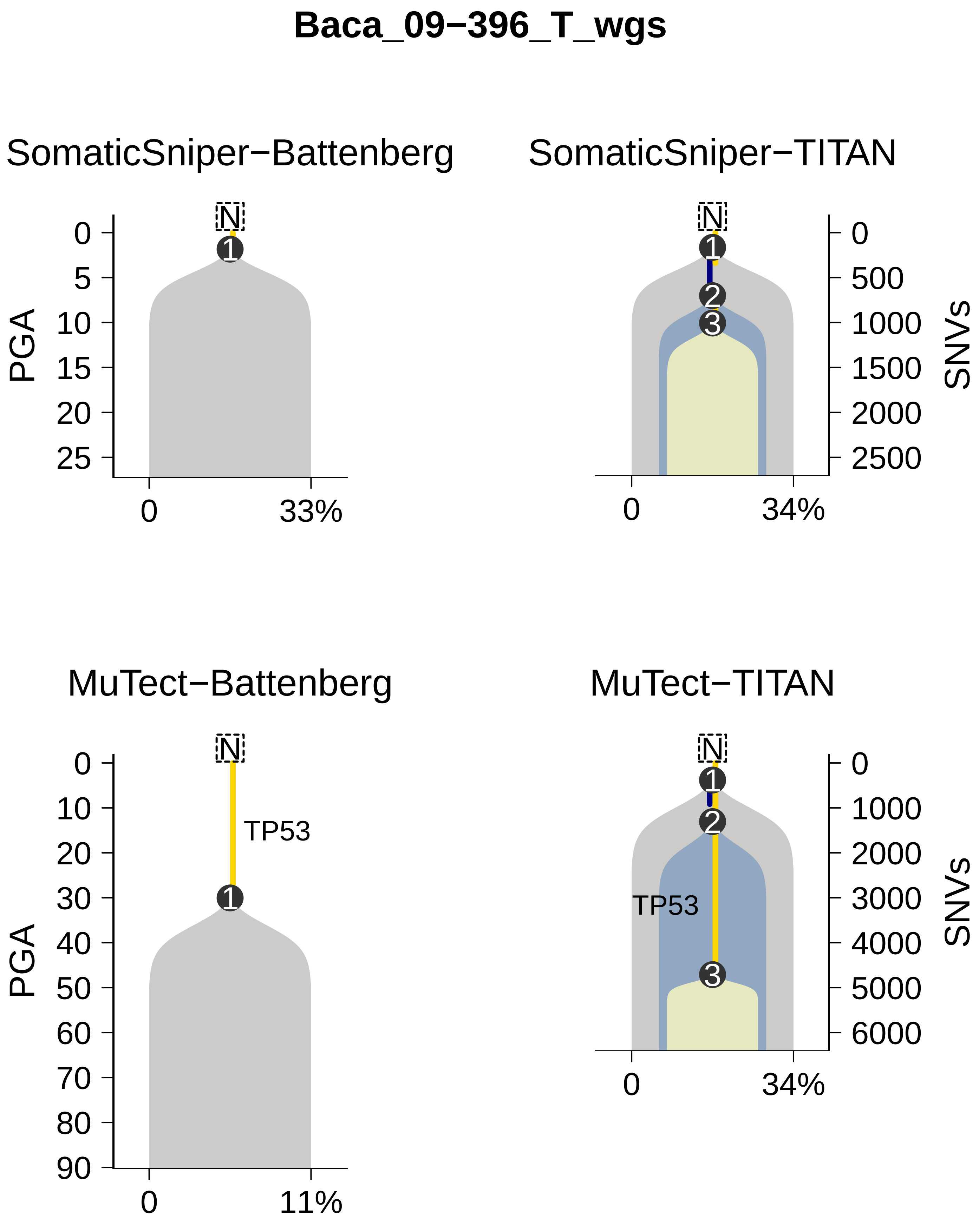

Baca_09−396_T_wgs
SomaticSniper−Battenberg
SomaticSniper−TITAN
MuTect−Battenberg
MuTect−TITAN
N
1
2
3
PGA
SNVs
0
5
10
15
20
25
33%
34%
500
1000
1500
2000
2500
30
40
50
60
70
80
90
11%
TP53
3000
4000
5000
6000

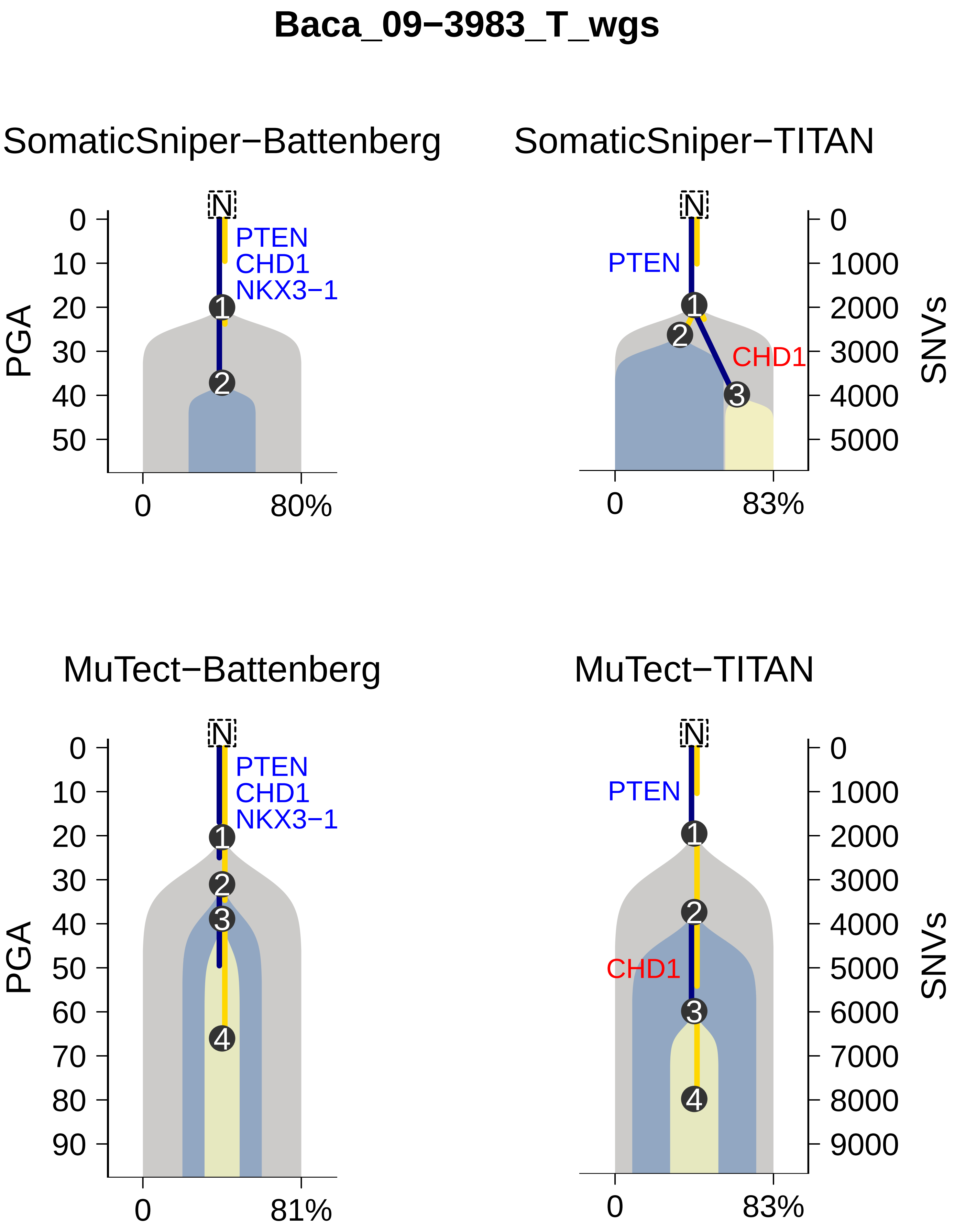

Baca_09−3983_T_wgs
SomaticSniper−Battenberg
SomaticSniper−TITAN
MuTect−Battenberg
MuTect−TITAN
N
PTEN
CHD1
NKX3−1
PGA
SNVs
0
10
20
30
40
50
60
70
80
90
0
1000
2000
3000
4000
5000
6000
7000
8000
9000
80%
83%
81%
1
2
3
4

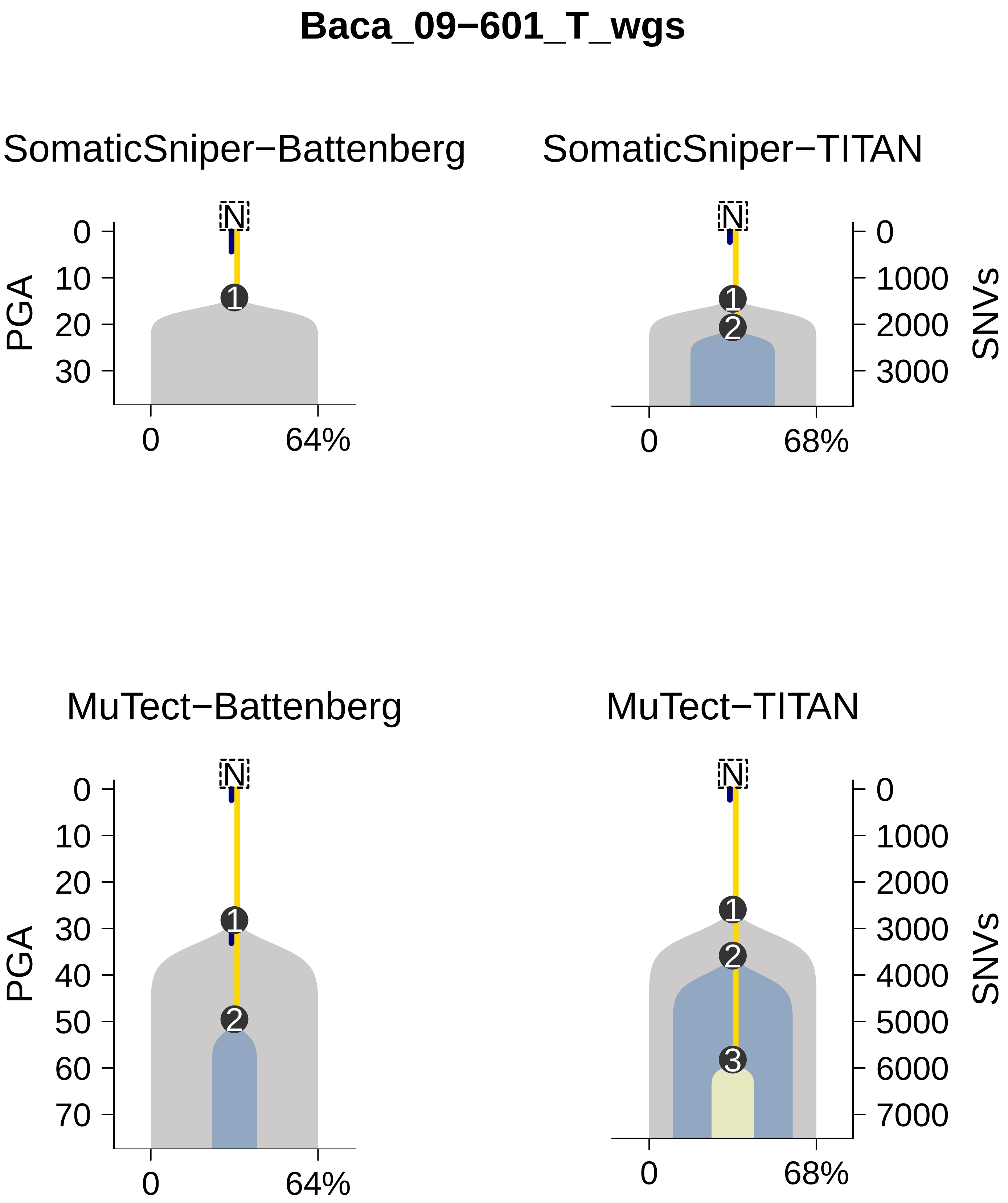
Baca_09−601_T_wgs
SomaticSniper−Battenberg
SomaticSniper−TITAN
MuTect−Battenberg
MuTect−TITAN
N
1
2
3
PGA
SNVs
0
10
20
30
40
50
60
70
1000
2000
3000
4000
5000
6000
7000
64%
68%

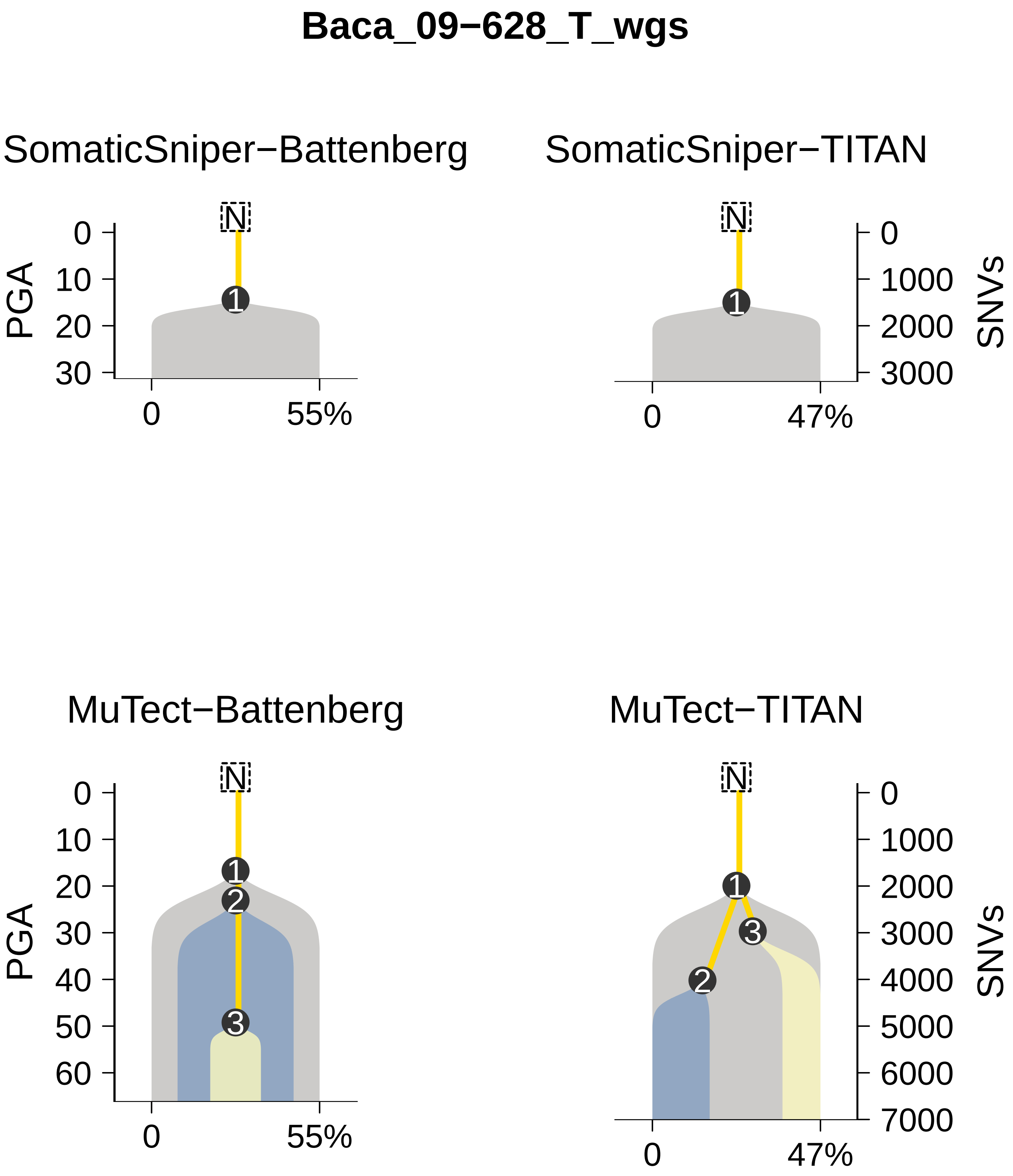

Baca_09−628_T_wgs
SomaticSniper−Battenberg
SomaticSniper−TITAN
MuTect−Battenberg
MuTect−TITAN
N
1
2
3
PGA
SNVs
0
10
20
30
40
50
60
1000
2000
3000
4000
5000
6000
7000
55%
47%

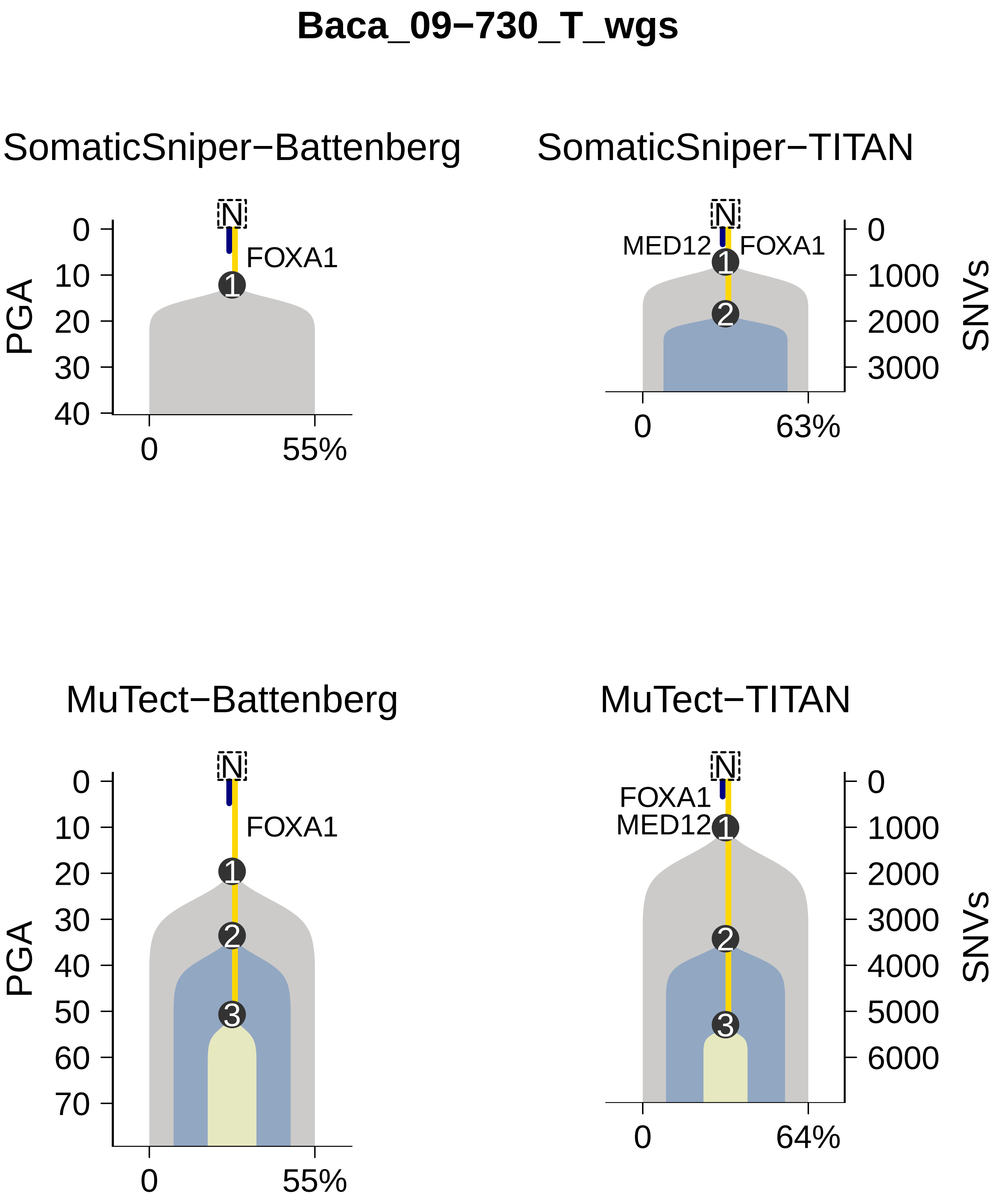

Baca_09−730_T_wgs
SomaticSniper−Battenberg
SomaticSniper−TITAN
MuTect−Battenberg
MuTect−TITAN
N
FOXA1
MED12
PGA
SNVs
0
10
20
30
40
50
60
70
1000
2000
3000
4000
5000
6000
55%
63%
64%

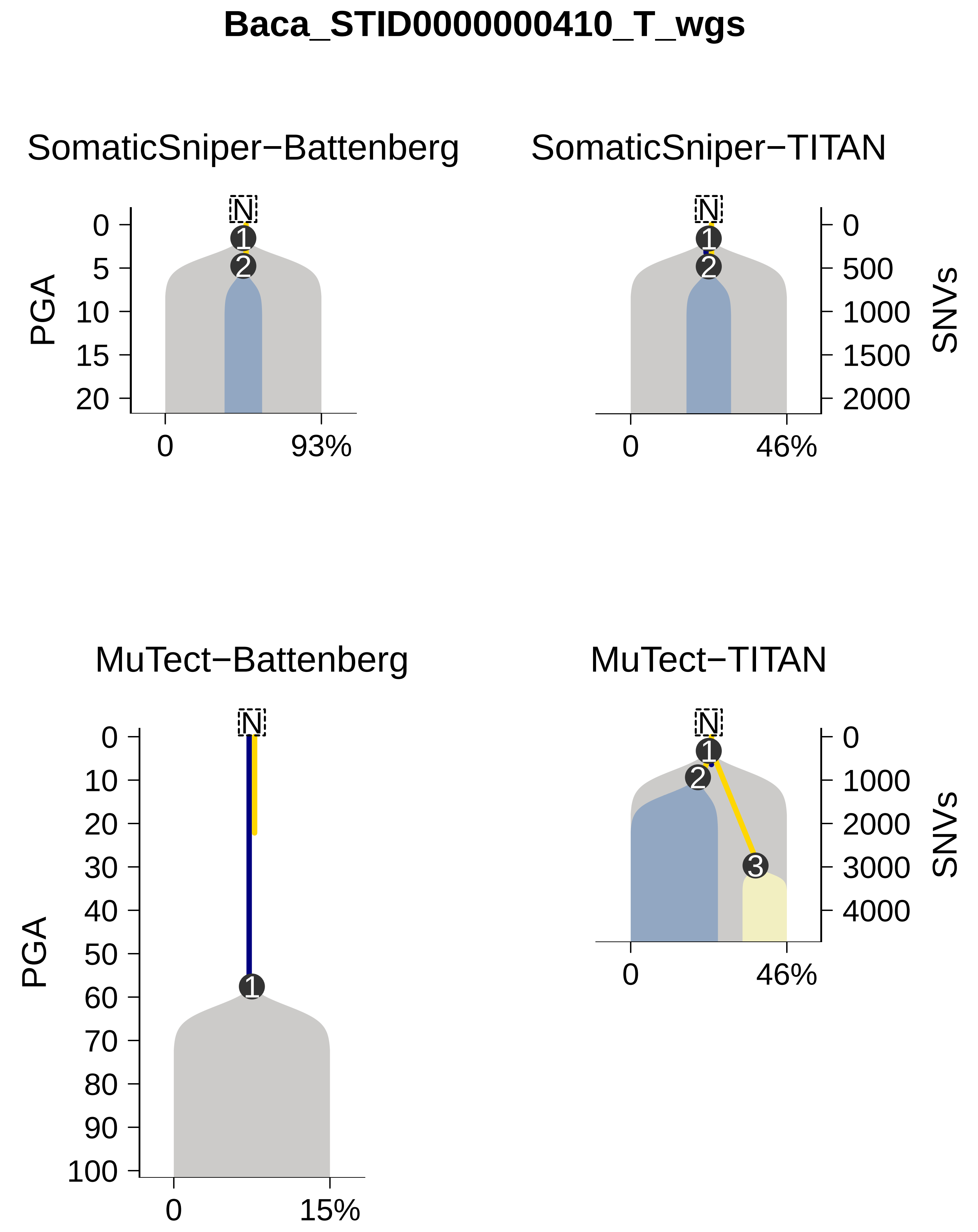
Baca_STID0000000410_T_wgs
SomaticSniper−Battenberg
SomaticSniper−TITAN
MuTect−Battenberg
MuTect−TITAN
PGA
SNVs
N
1
2
3
0
5
10
15
20
500
1000
1500
2000
30
40
50
60
70
80
90
100
3000
4000
93%
46%
15%

Baca_STID0000000415_T_wgs

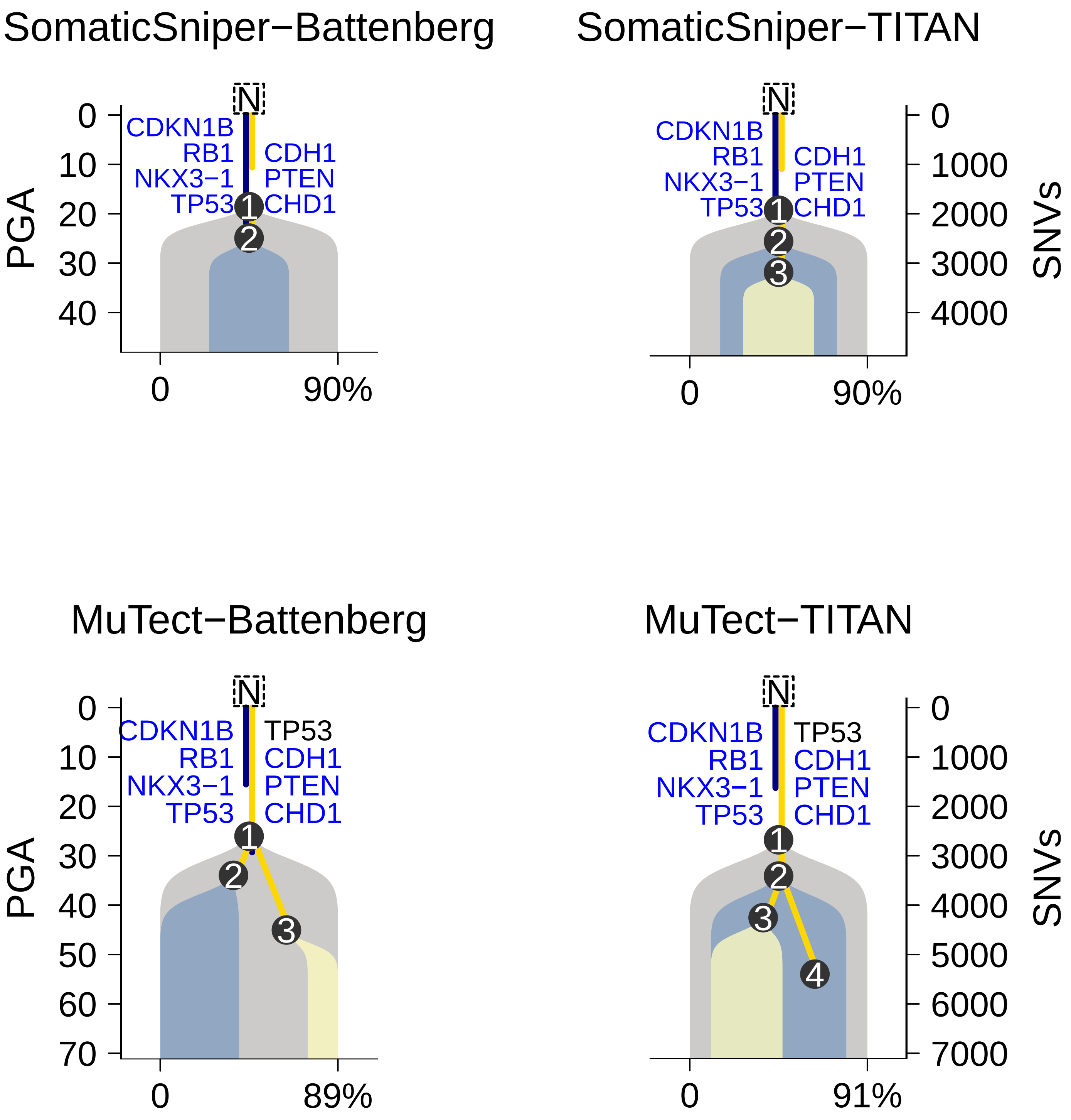

Baca_STID0000002525_T_wgs

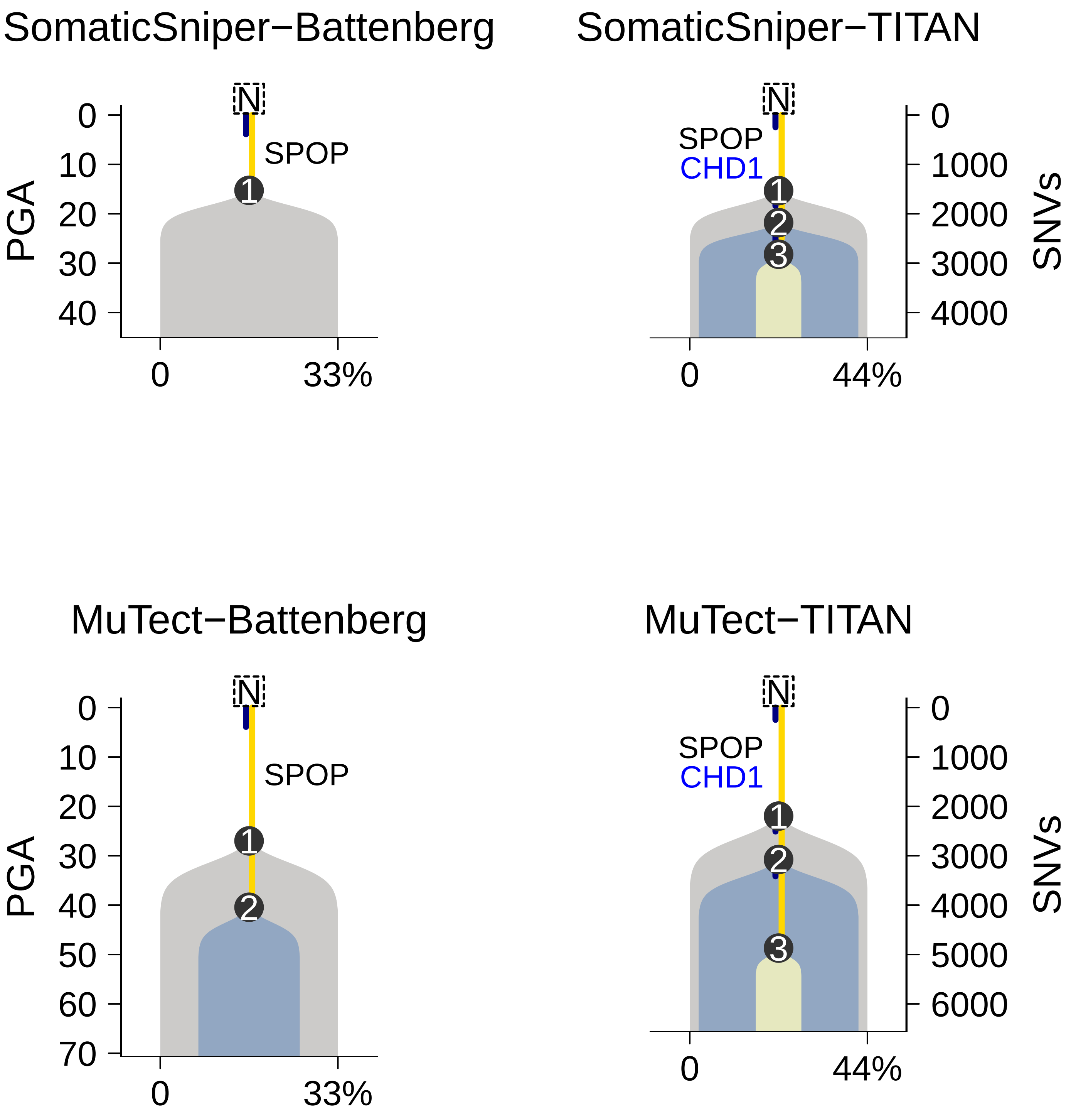

Baca_STID0000002621_T_wgs

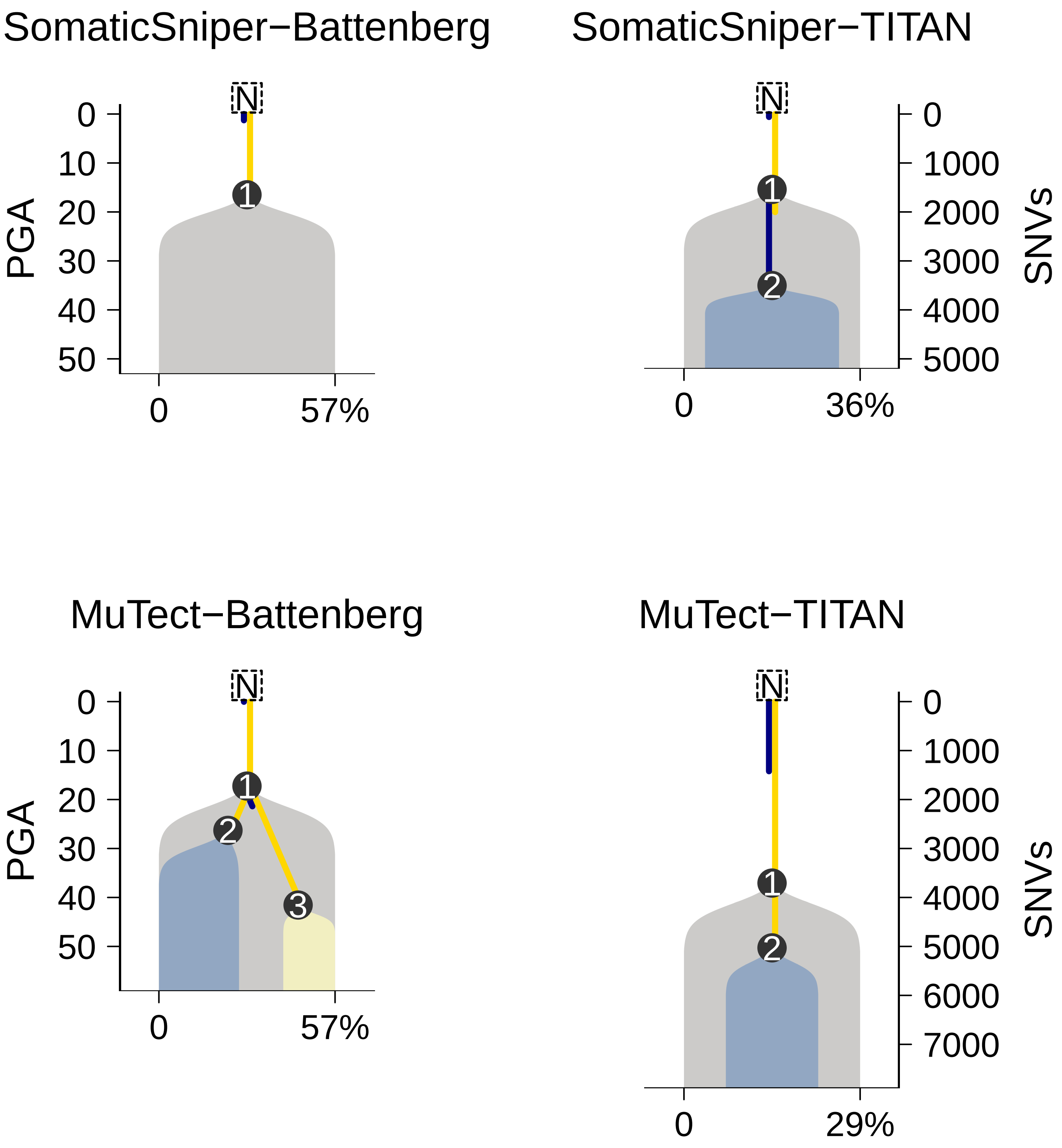

Baca_STID0000002682_T_wgs

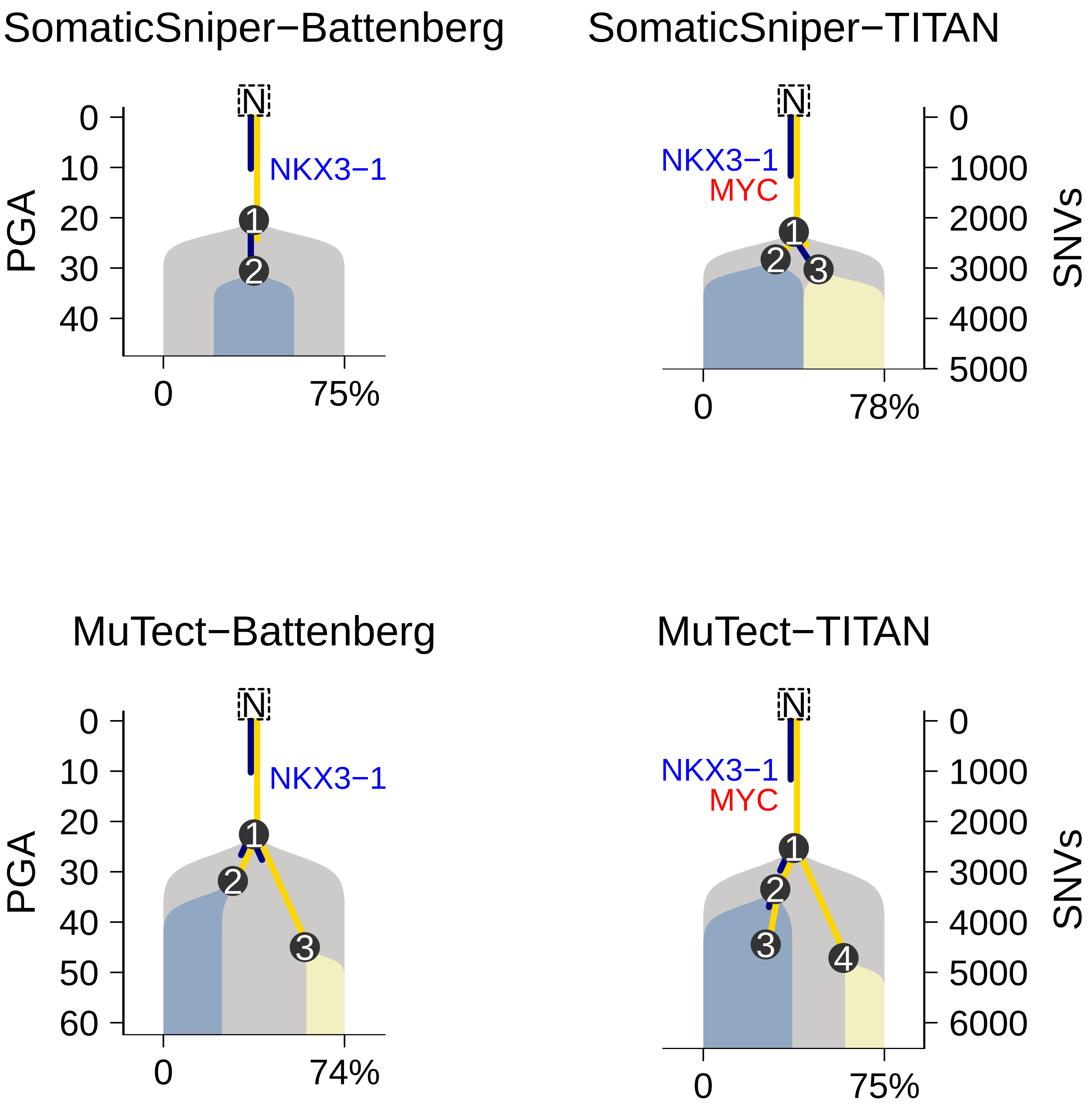

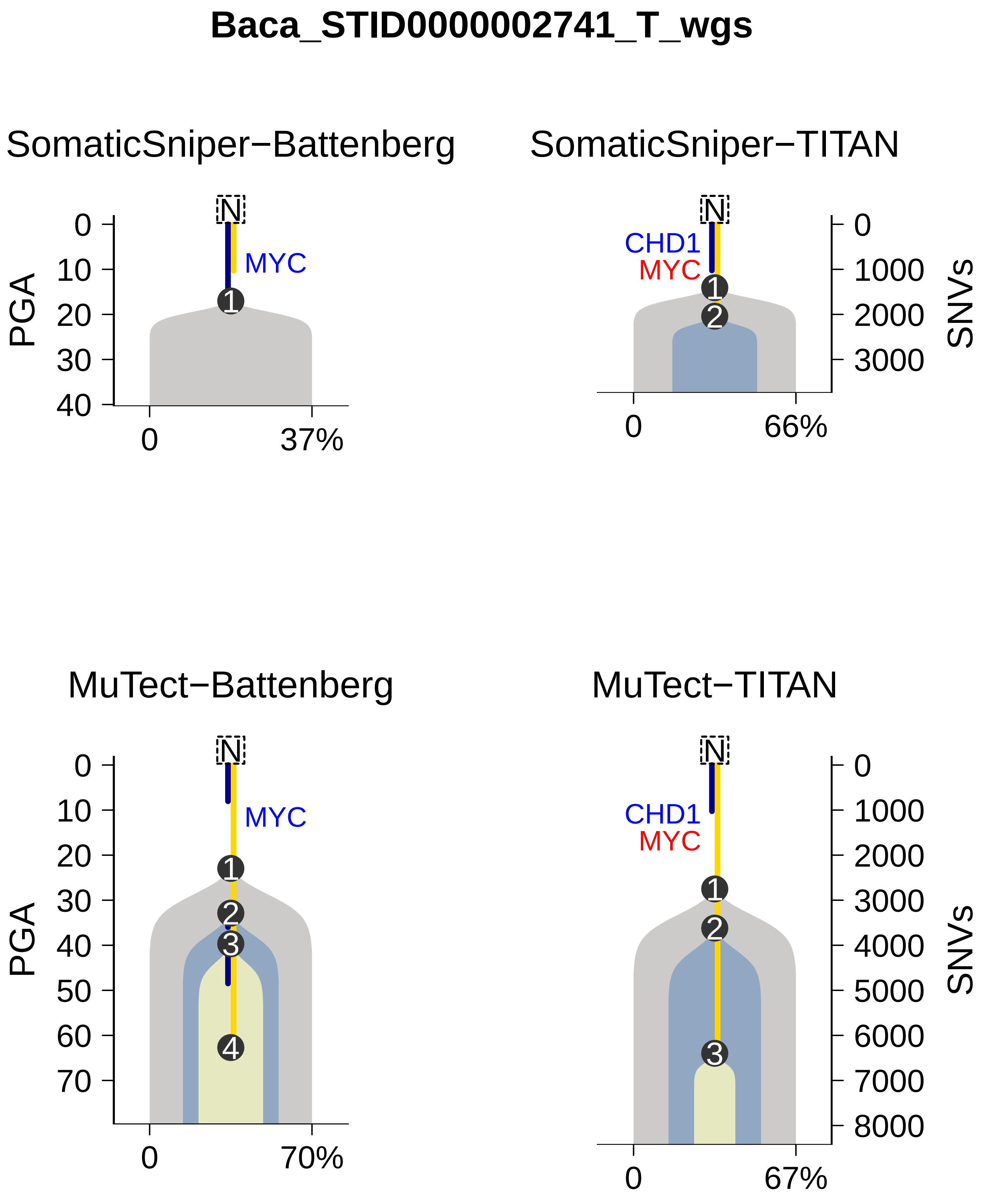
Baca_STID0000002741_T_wgs
SomaticSniper−Battenberg
SomaticSniper−TITAN
MuTect−Battenberg
MuTect−TITAN
N
MYC
CHD1
PGA
SNVs
0
10
20
30
40
50
60
70
1000
2000
3000
4000
5000
6000
7000
8000
37%
66%
70%
67%

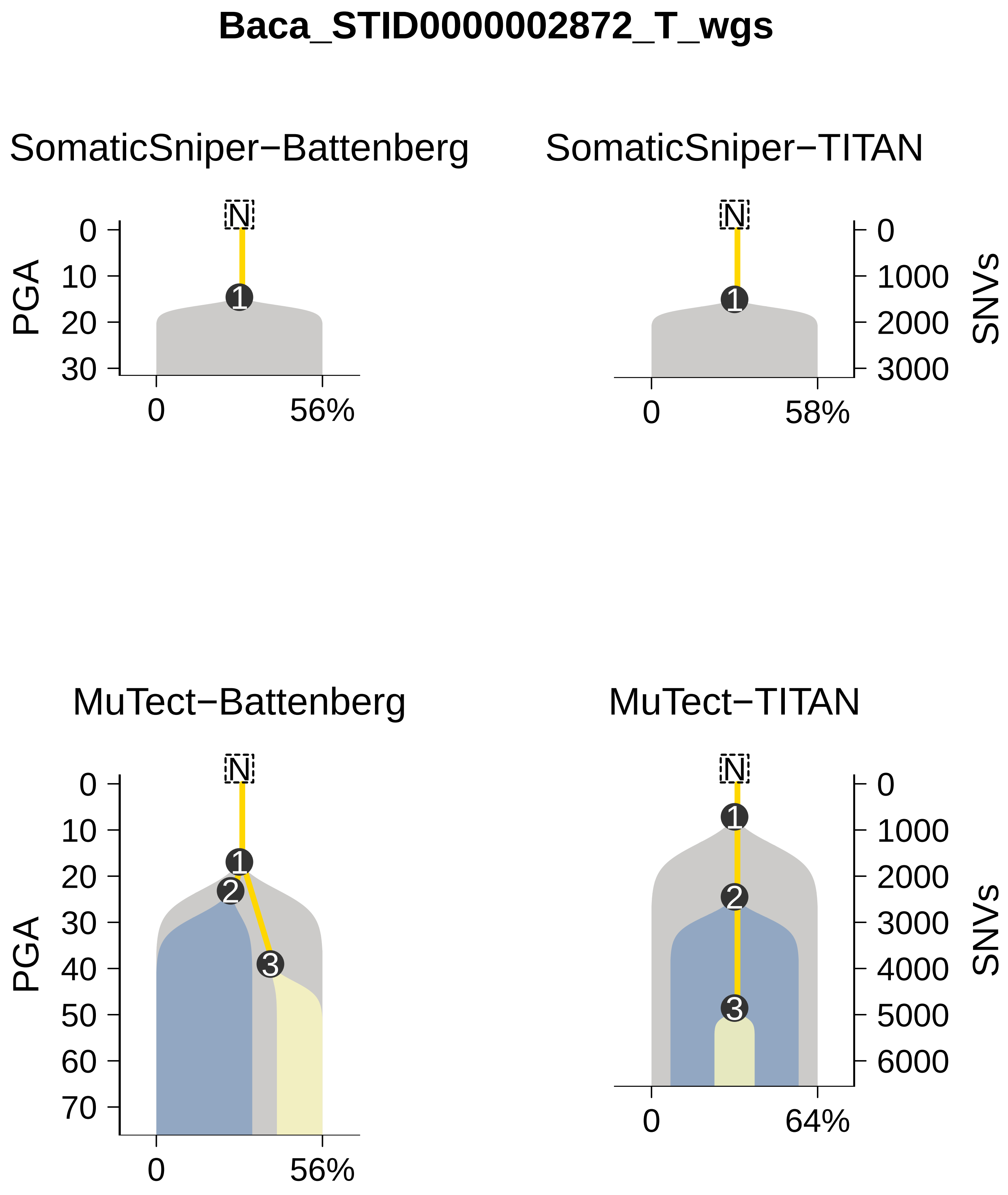

Baca_STID0000002872_T_wgs
SomaticSniper−Battenberg
SomaticSniper−TITAN
MuTect−Battenberg
MuTect−TITAN
N
1
2
3
PGA
SNVs
0
10
20
30
40
50
60
70
1000
2000
3000
4000
5000
6000
56%
58%
64%

Baca_STID0000003042_T_wgs

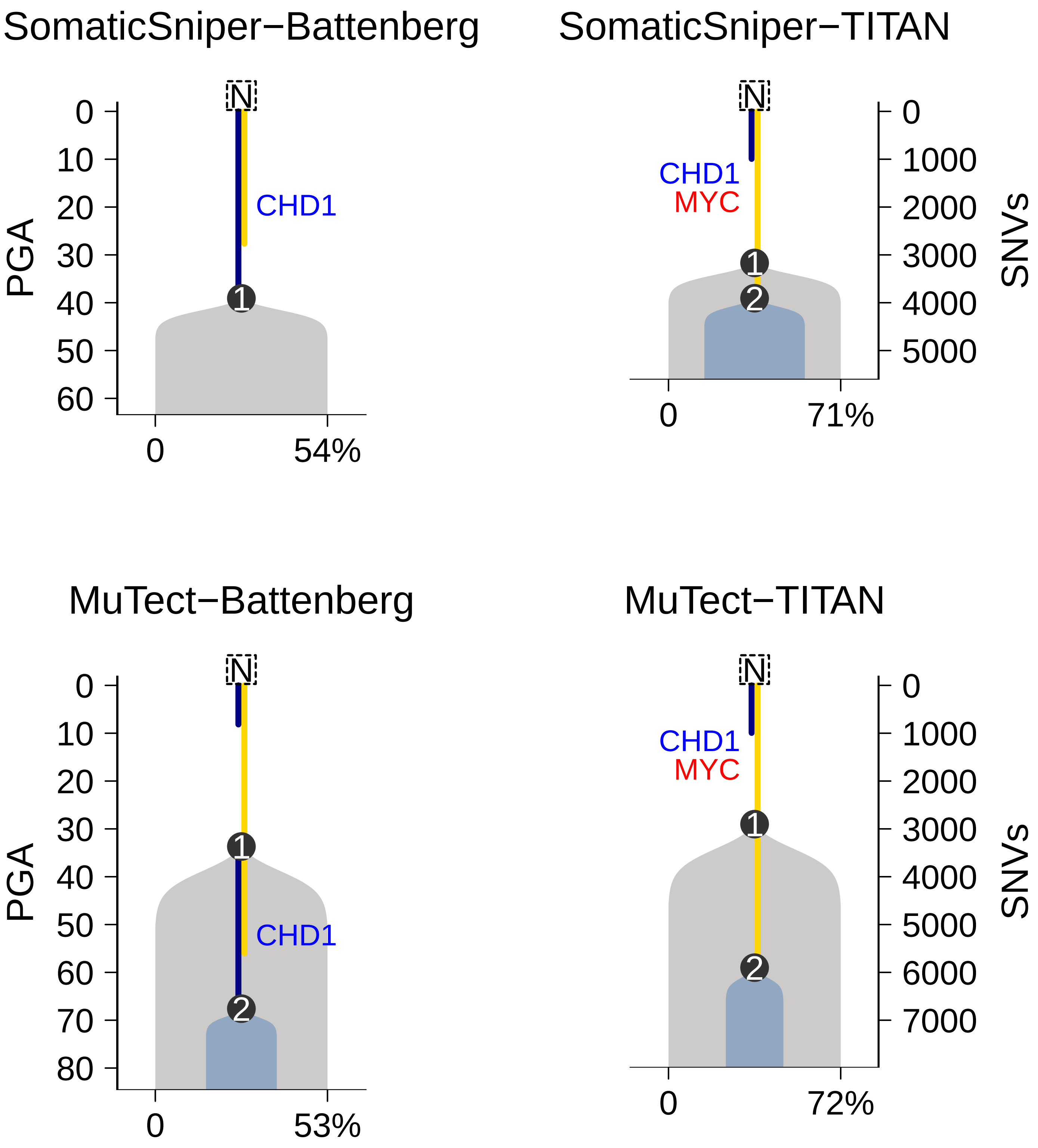

Baca_STID0000003127_T_wgs

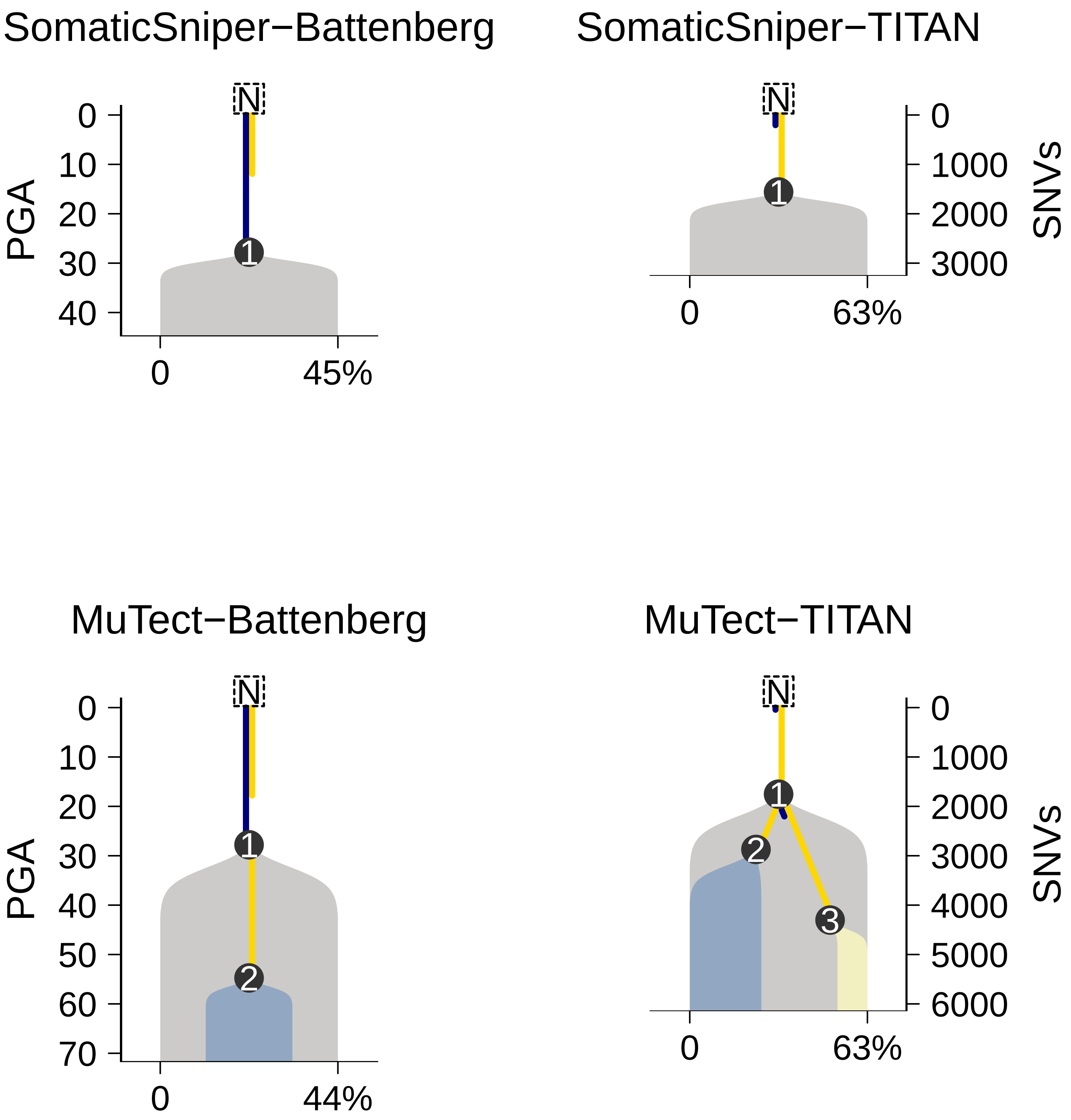

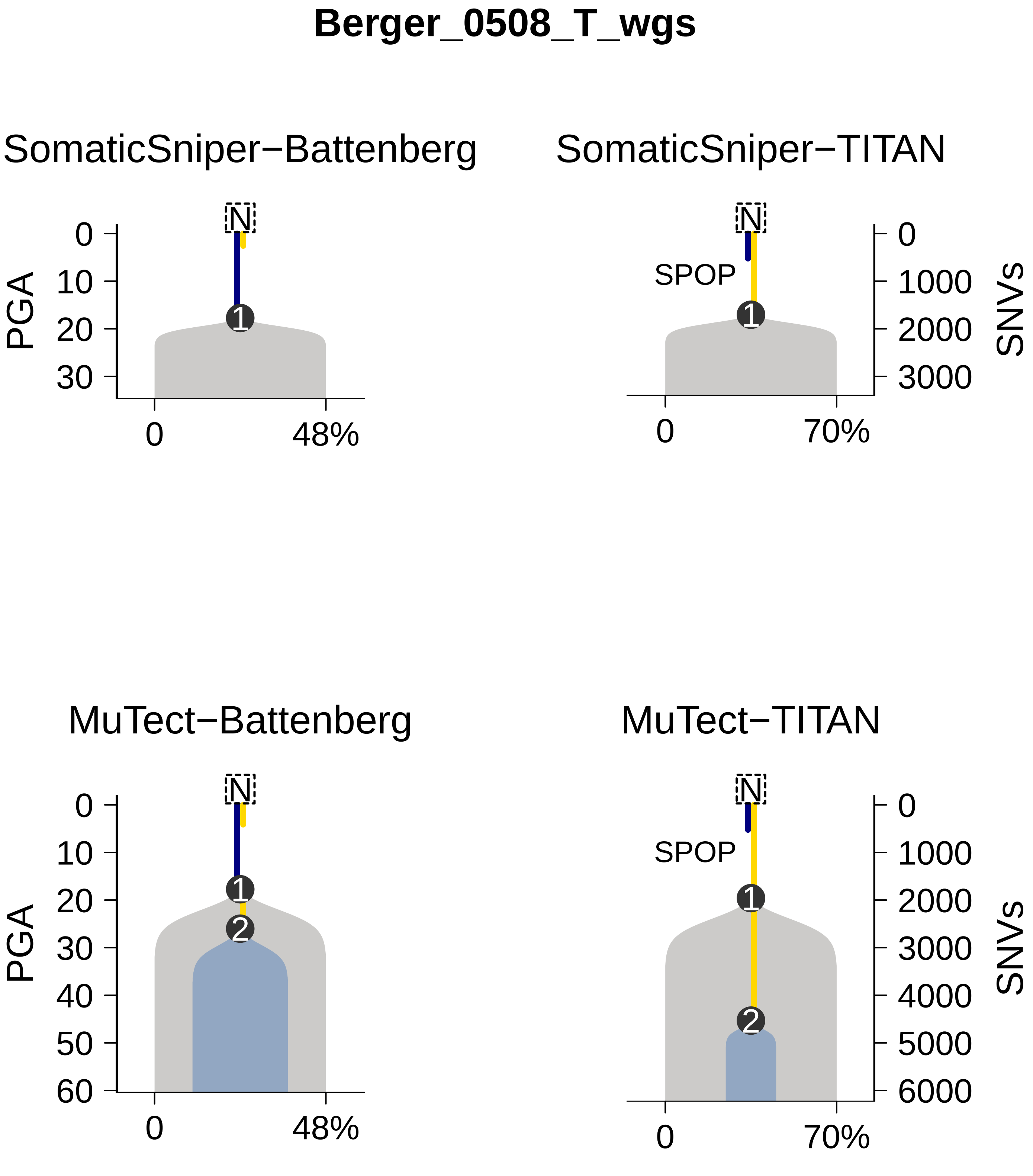
Berger_0508_T_wgs
SomaticSniper−Battenberg
SomaticSniper−TITAN
MuTect−Battenberg
MuTect−TITAN
N
SPOP
PGA
SNVs
0
10
20
30
40
50
60
48%
70%
1000
2000
3000
4000
5000
6000
1
2

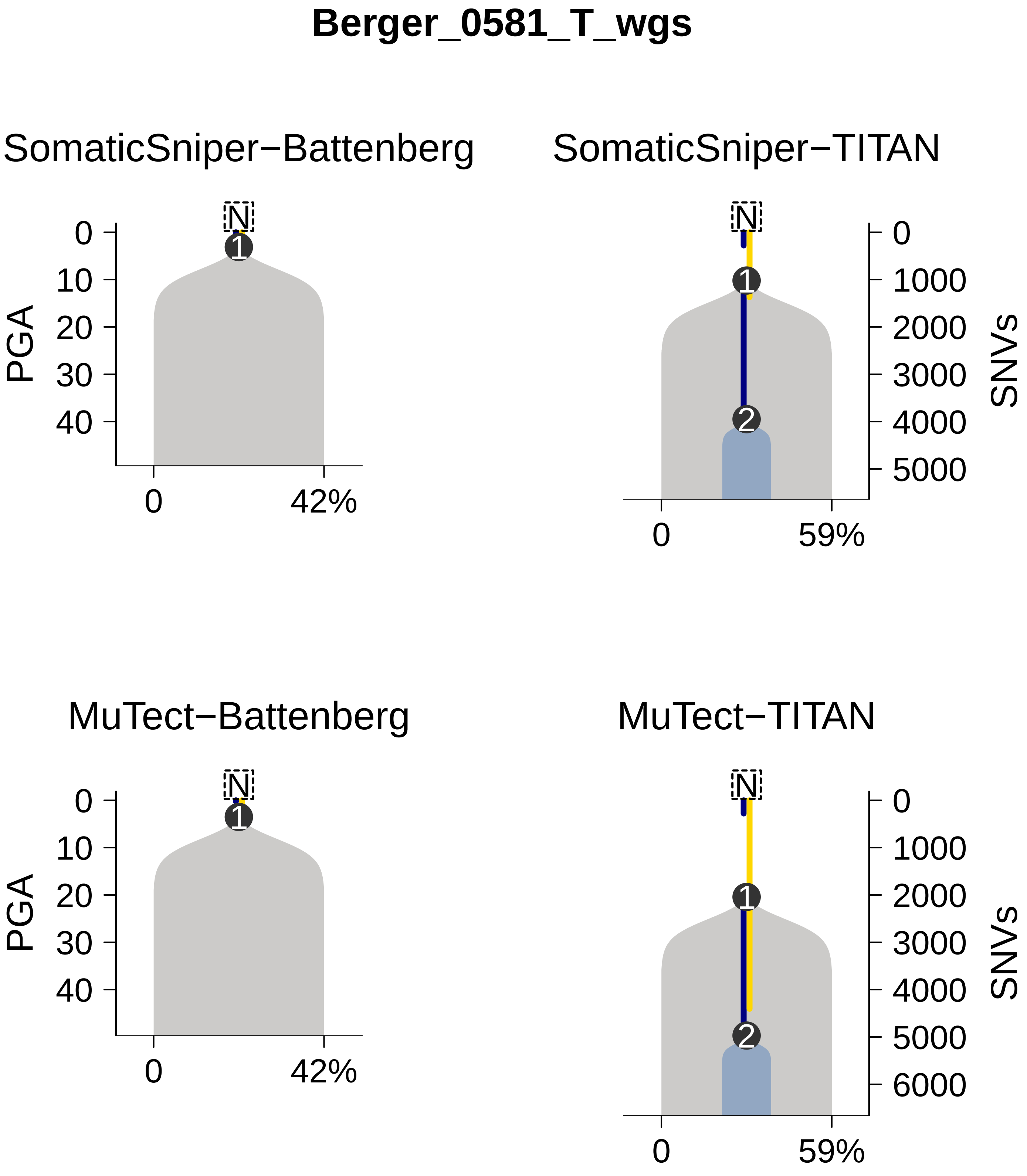
Berger_0581_T_wgs
SomaticSniper−Battenberg
SomaticSniper−TITAN
MuTect−Battenberg
MuTect−TITAN
N
1
2
PGA
0
10
20
30
40
42%
59%
SNVs
1000
2000
3000
4000
5000
6000

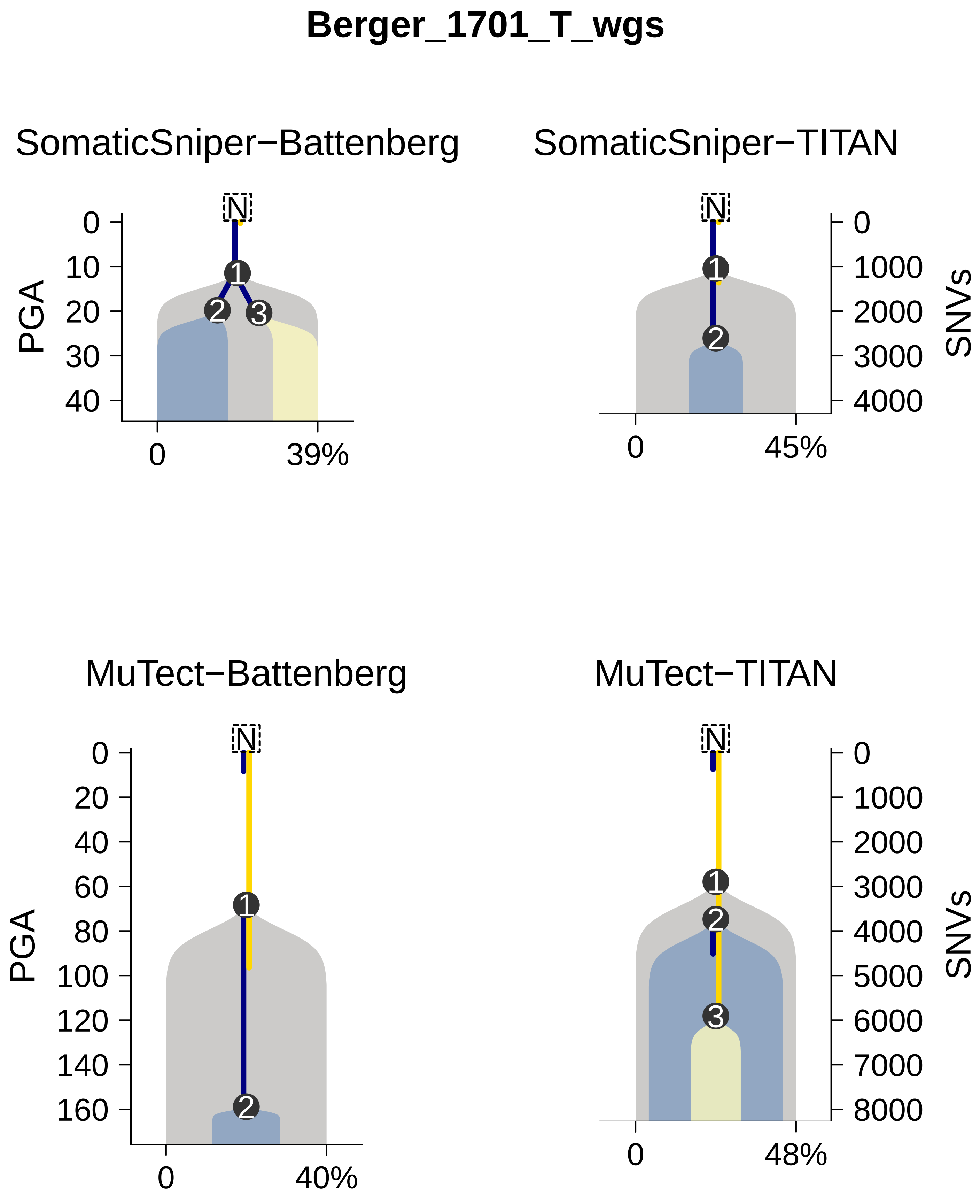
Berger_1701_T_wgs
SomaticSniper−Battenberg
SomaticSniper−TITAN
MuTect−Battenberg
MuTect−TITAN
PGA
SNVs
N
1
2
3
0
39%
45%
40%
48%

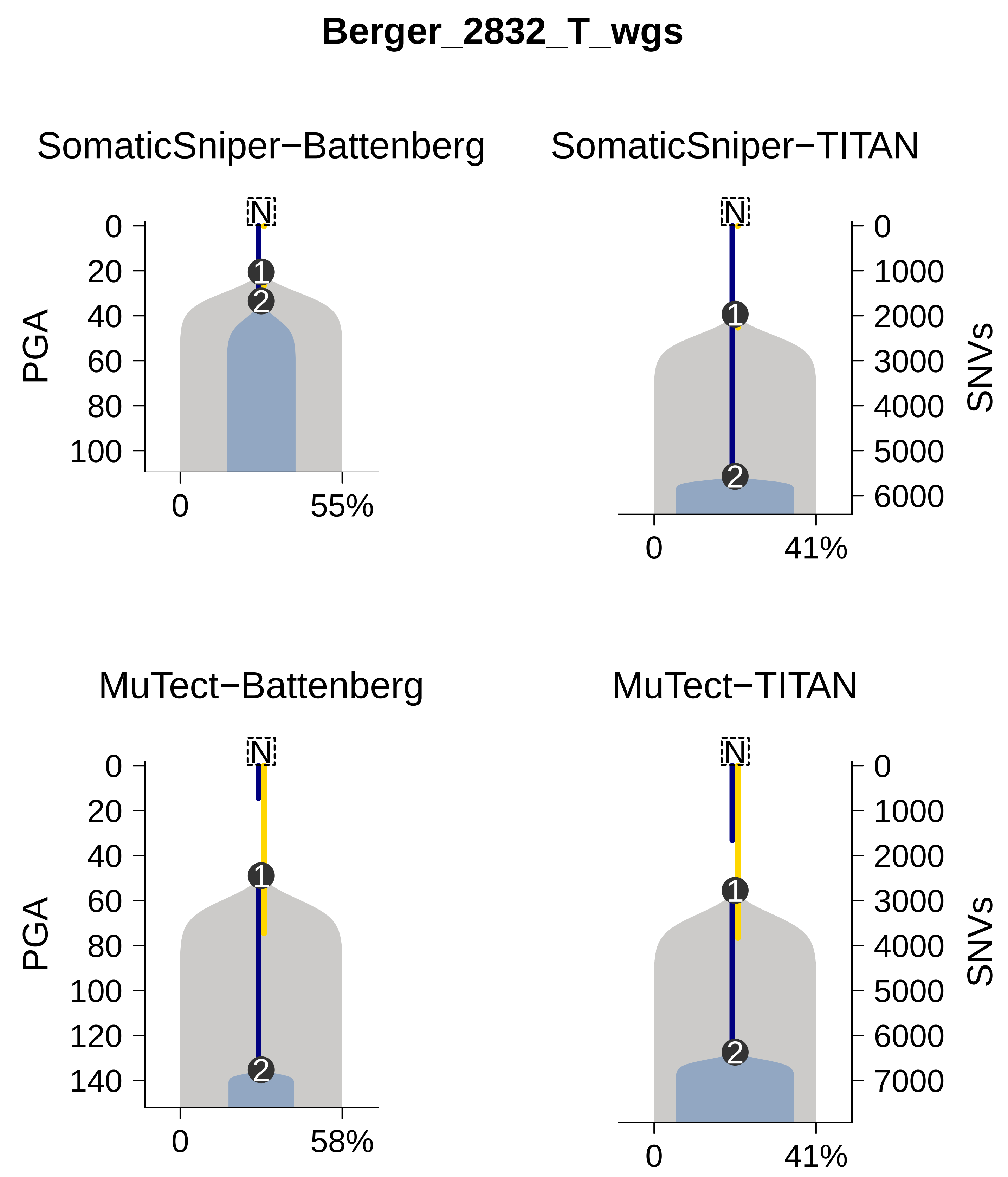
Berger_2832_T_wgs
SomaticSniper−Battenberg
SomaticSniper−TITAN
MuTect−Battenberg
MuTect−TITAN
PGA
SNVs
N
1
2
0
20
40
60
80
100
120
140
55%
41%
58%
1000
2000
3000
4000
5000
6000
7000

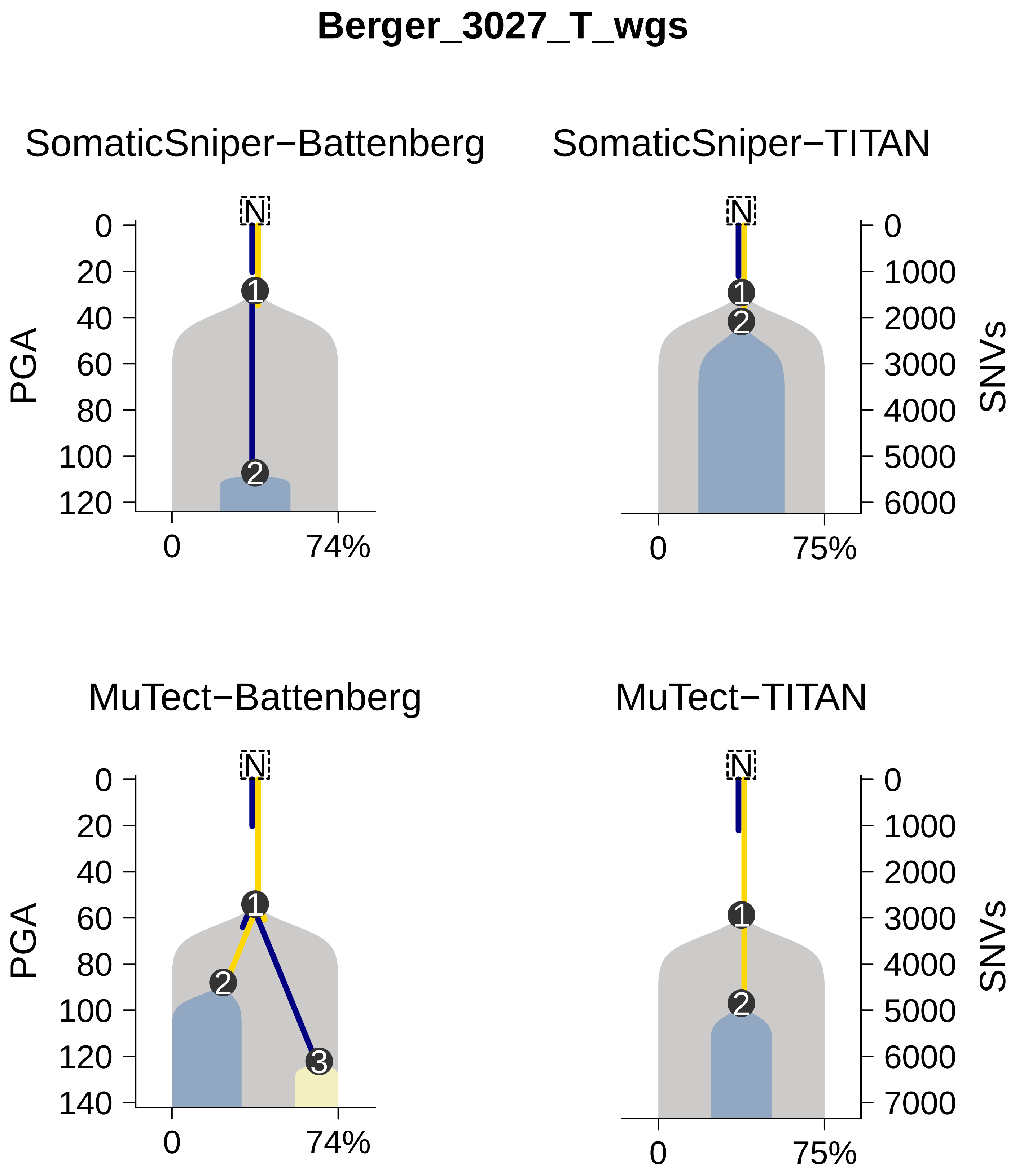
Berger_3027_T_wgs
SomaticSniper−Battenberg
SomaticSniper−TITAN
MuTect−Battenberg
MuTect−TITAN
PGA
SNVs
N
0
20
40
60
80
100
120
140
0
1000
2000
3000
4000
5000
6000
7000
74%
75%
1
2
3

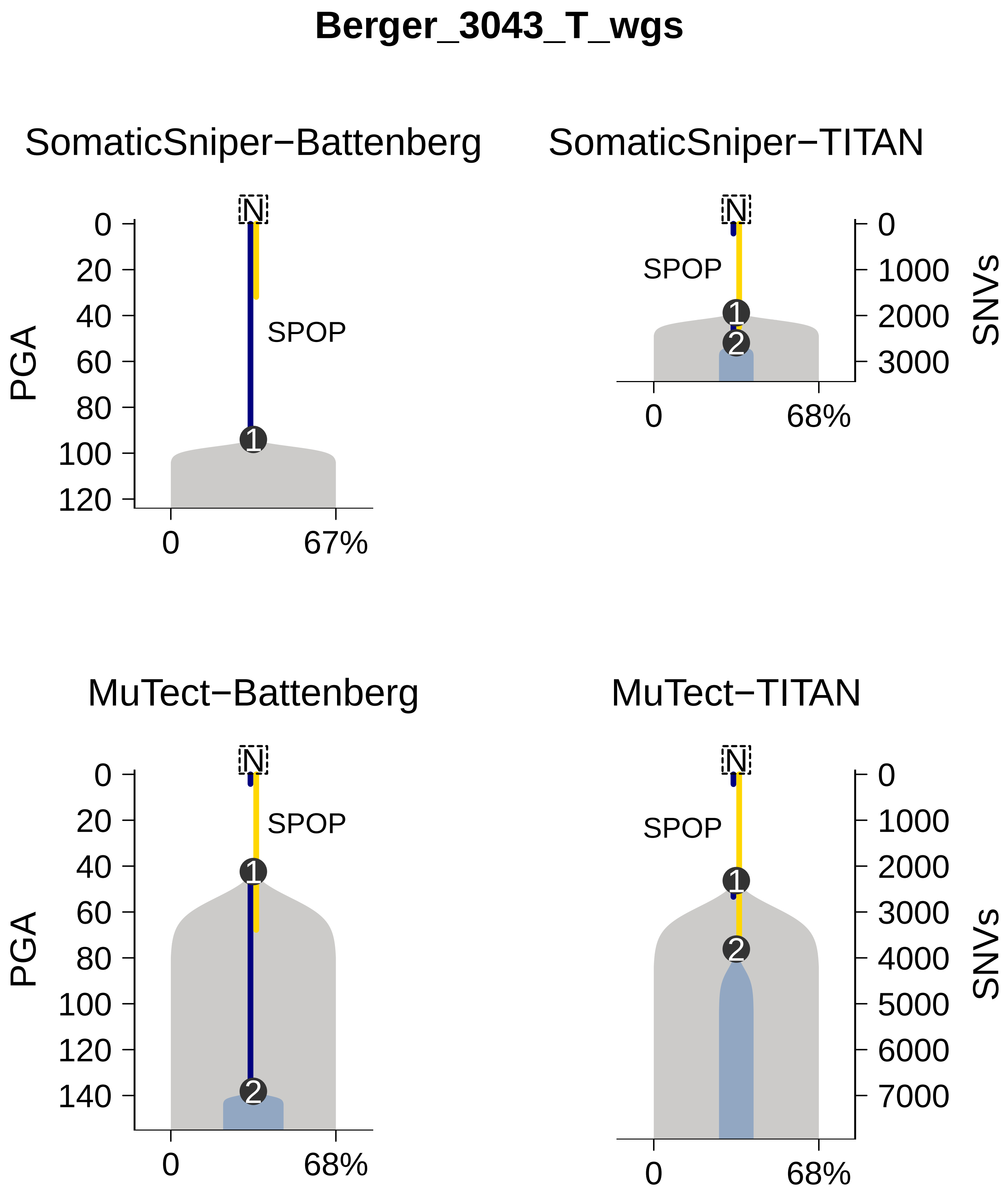
Berger_3043_T_wgs
SomaticSniper−Battenberg
SomaticSniper−TITAN
MuTect−Battenberg
MuTect−TITAN
N
1
2
SPOP
PGA
SNVs
0
20
40
60
80
100
120
140
1000
2000
3000
4000
5000
6000
7000
67%
68%

CPCG0001

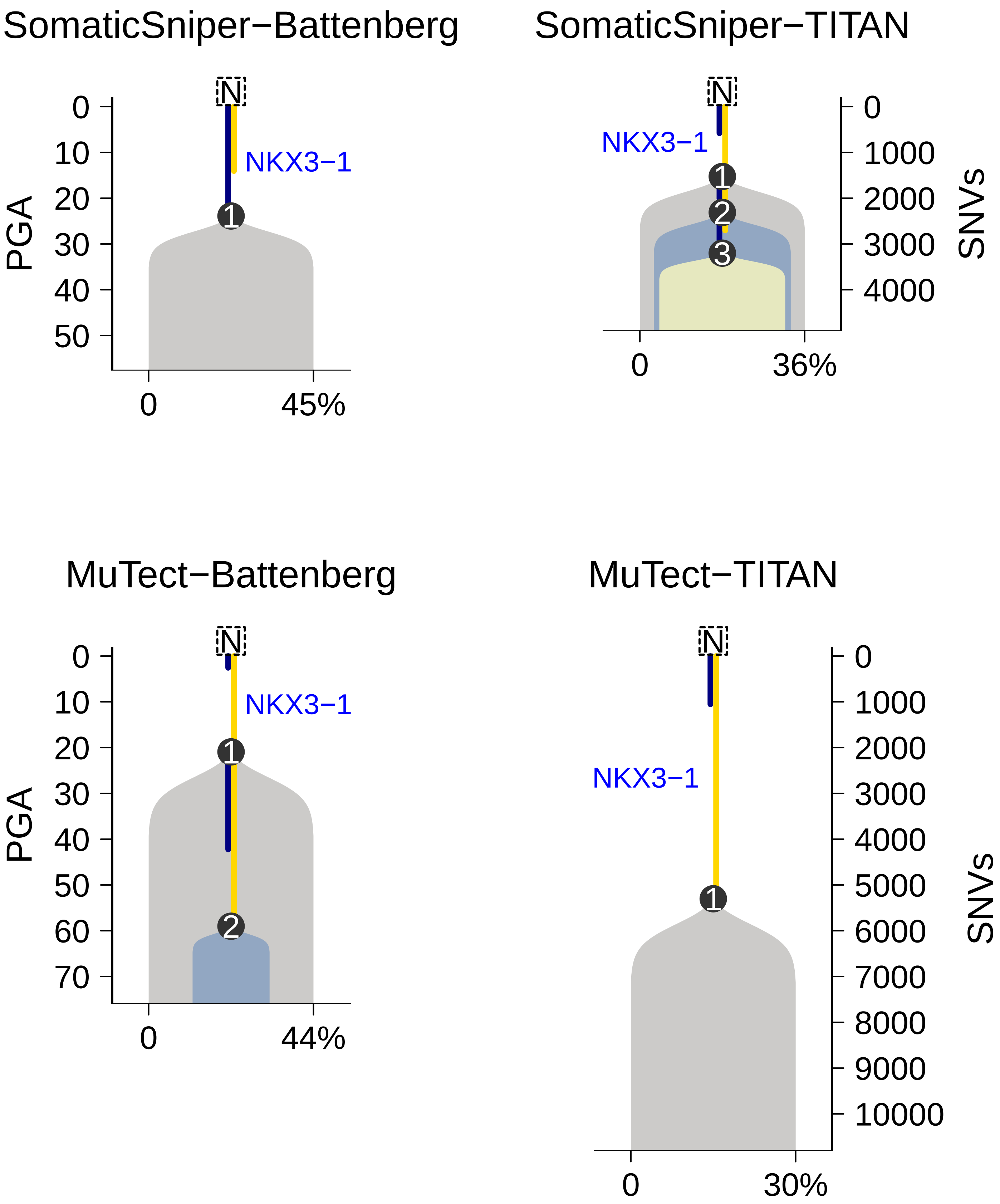

SomaticSniper−Battenberg
SomaticSniper−TITAN
MuTect−Battenberg
MuTect−TITAN
N
NKX3−1
PGA
SNVs
0
10
20
30
40
50
60
70
1000
2000
3000
4000
5000
6000
7000
8000
9000
10000
45%
36%
44%
30%
1
2
3

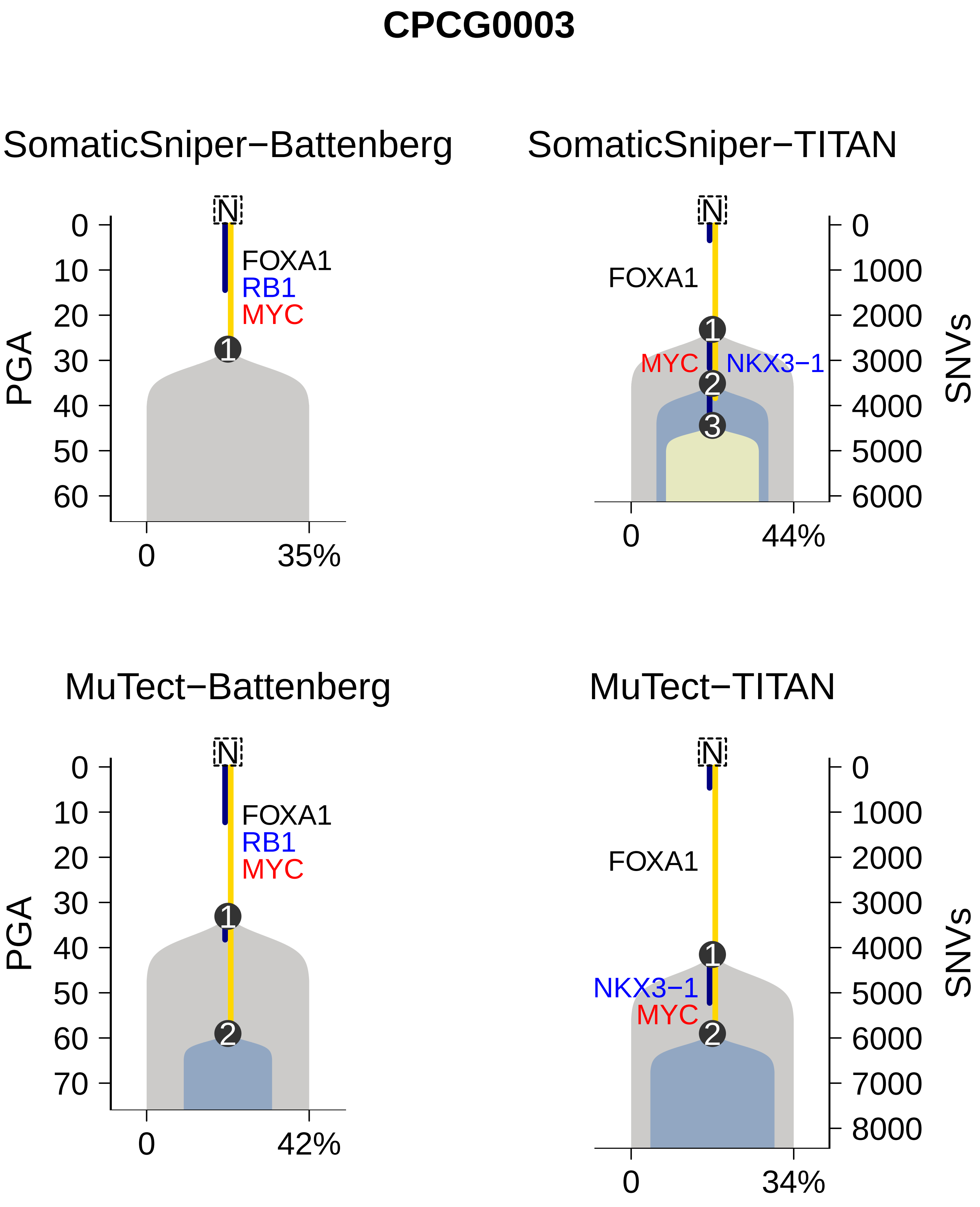
CPCG0003
SomaticSniper−Battenberg
SomaticSniper−TITAN
MuTect−Battenberg
MuTect−TITAN
N
FOXA1
RB1
MYC
NKX3−1
PGA
SNVs
0
10
20
30
40
50
60
70
1000
2000
3000
4000
5000
6000
7000
8000
35%
44%
42%
34%
1
2
3

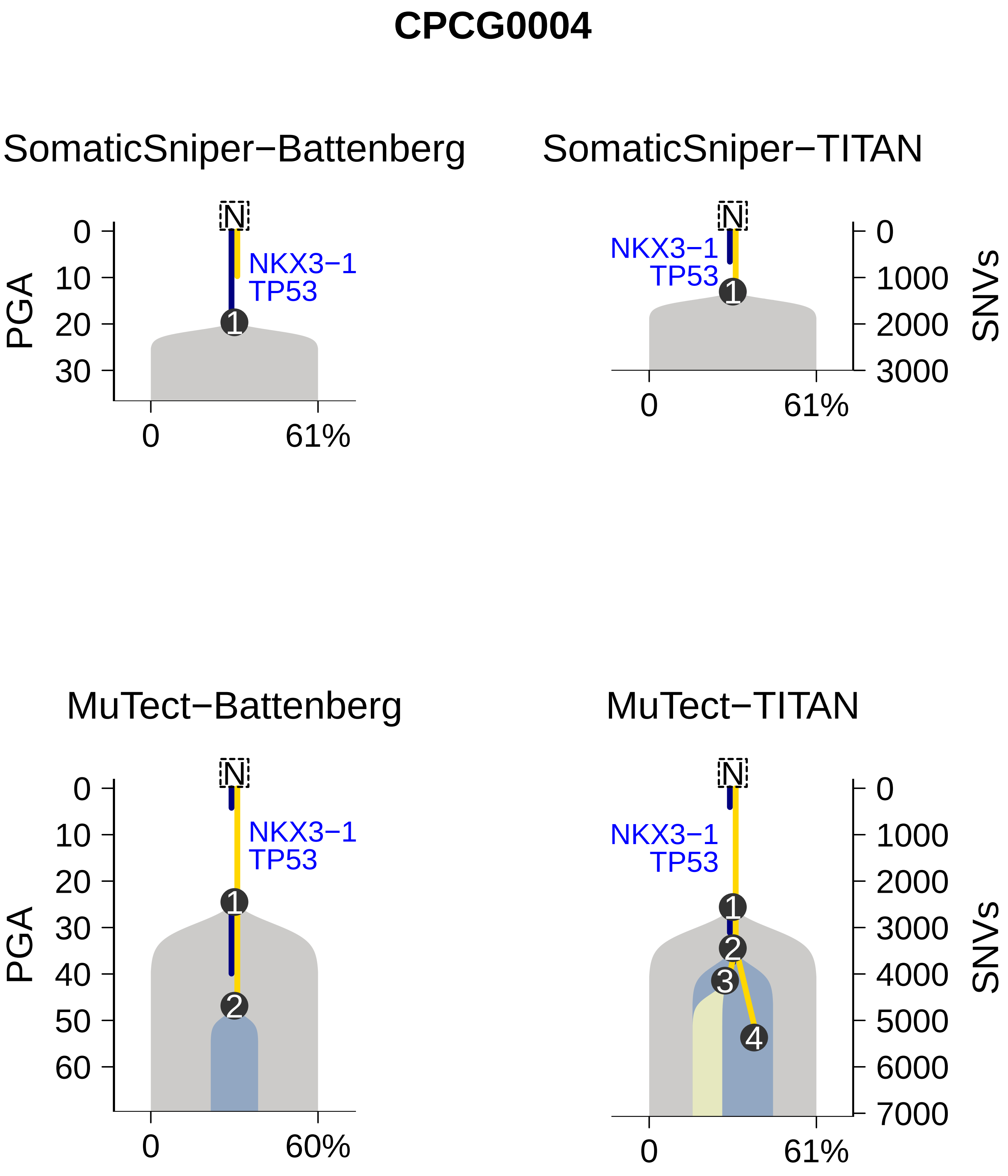
CPCG0004
SomaticSniper−Battenberg
SomaticSniper−TITAN
MuTect−Battenberg
MuTect−TITAN
N
NKX3−1
TP53
PGA
SNVs
0
10
20
30
40
50
60
1000
2000
3000
4000
5000
6000
7000
61%
60%
1
2
3
4

CPCG0007

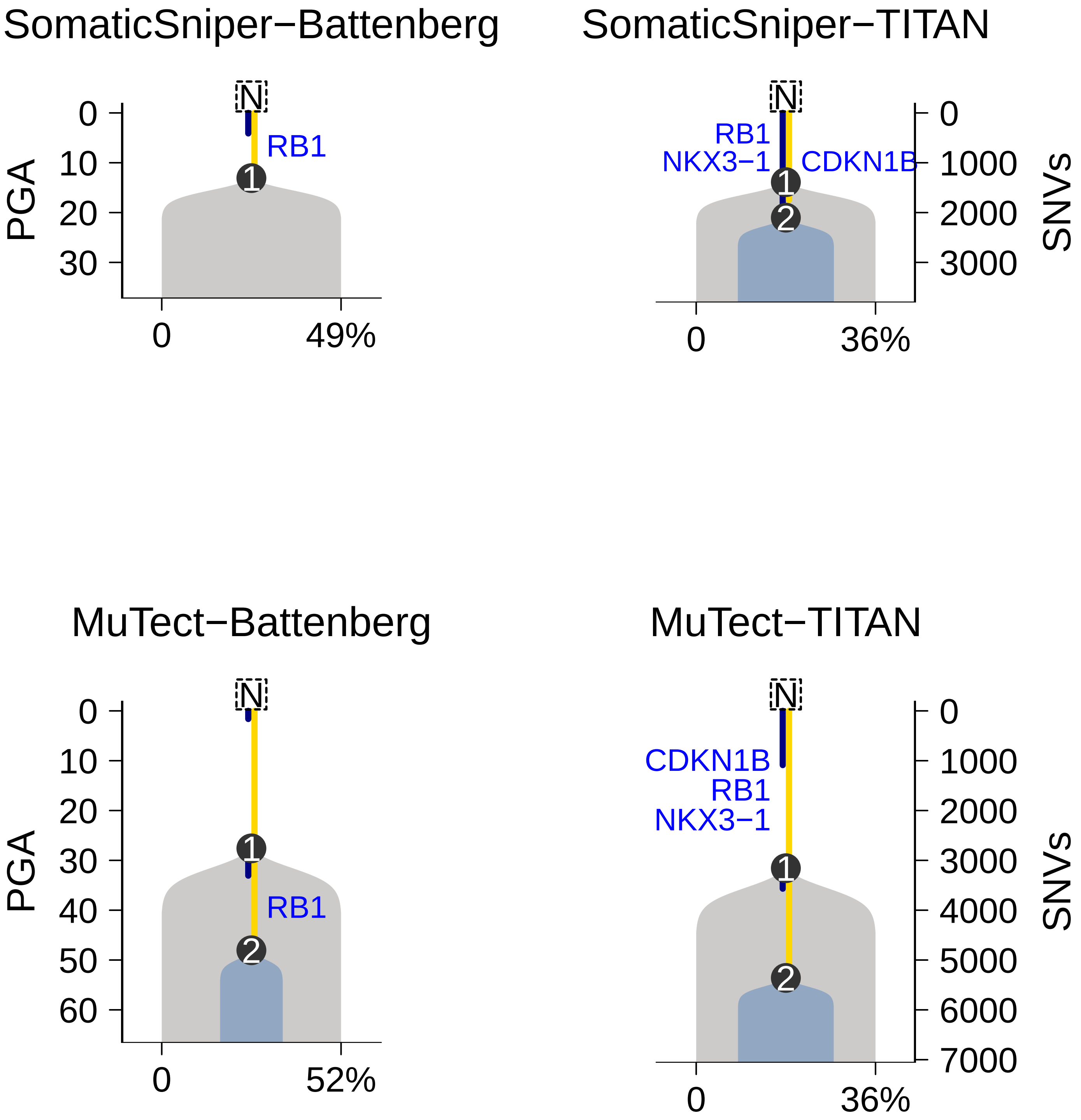
SomaticSniper−Battenberg
SomaticSniper−TITAN
MuTect−Battenberg
MuTect−TITAN
PGA
SNVs
N
1
2
RB1
NKX3−1
CDKN1B
0
10
20
30
40
50
60
1000
2000
3000
4000
5000
6000
7000
49%
52%
36%

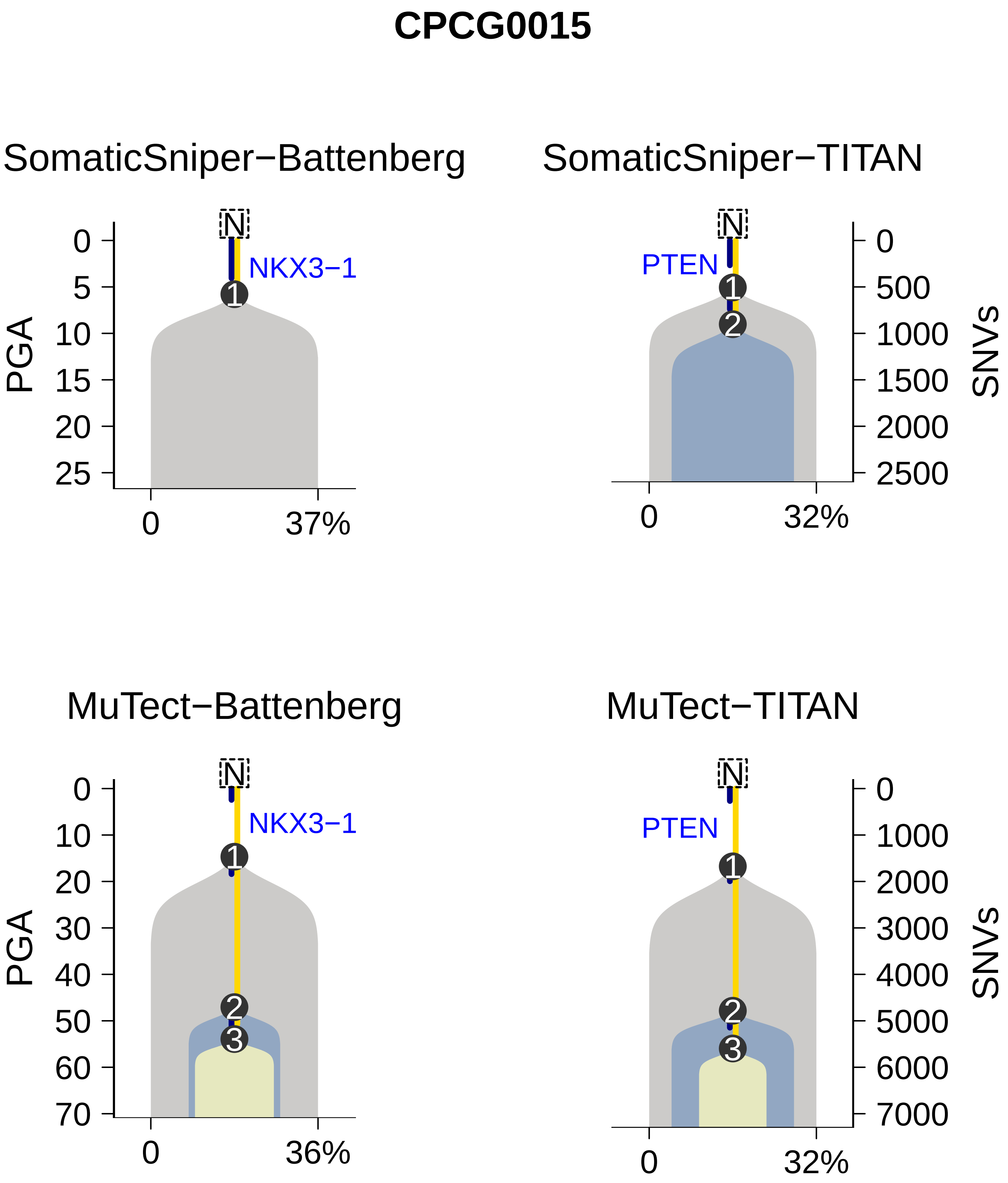
CPCG0015
SomaticSniper−Battenberg
SomaticSniper−TITAN
MuTect−Battenberg
MuTect−TITAN
N
NKX3−1
PTEN
1
2
3
PGA
SNVs
0
5
10
15
20
25
30
40
50
60
70
500
1000
1500
2000
2500
3000
4000
5000
6000
7000
37%
36%
32%

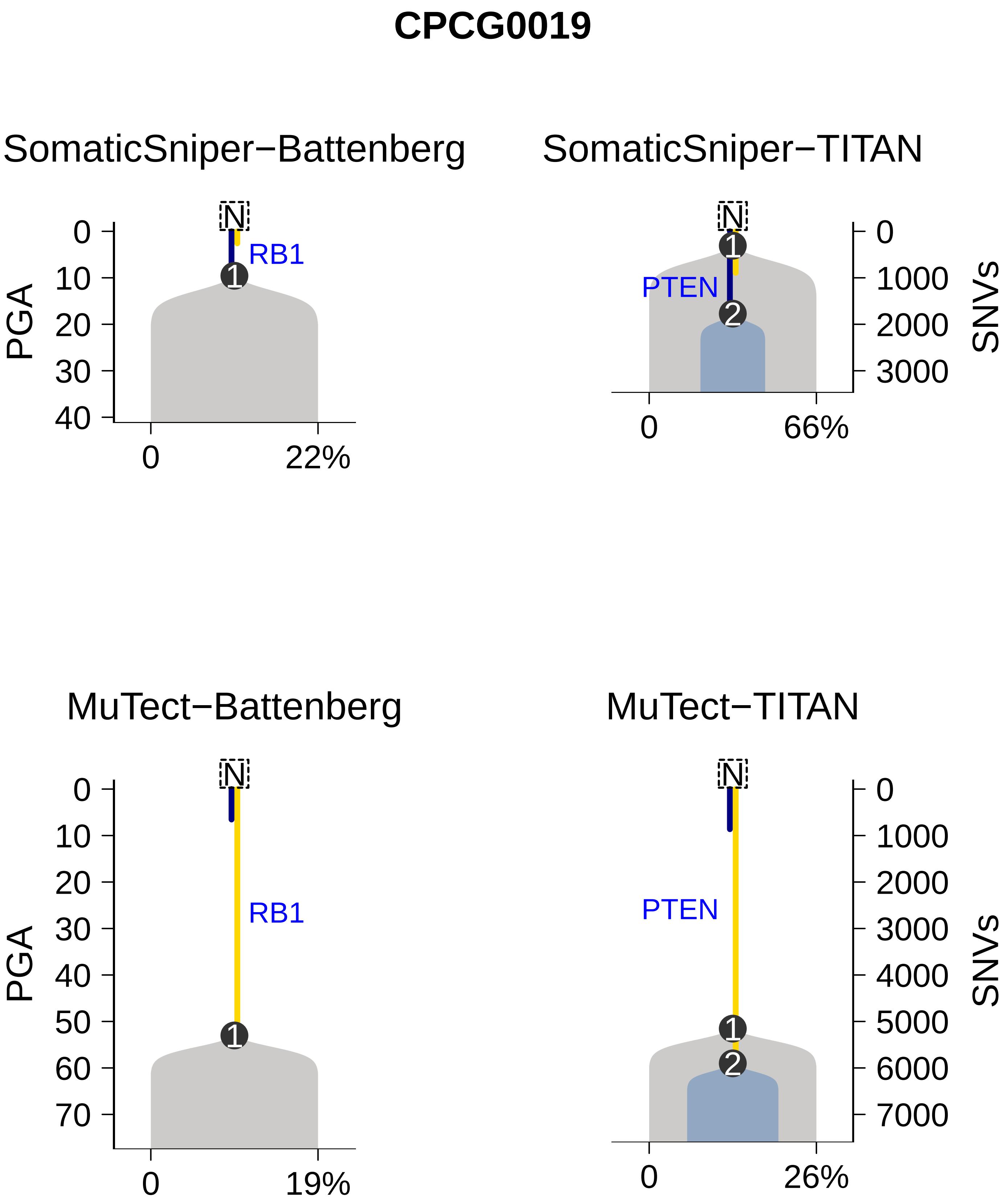
CPCG0019
SomaticSniper−Battenberg
SomaticSniper−TITAN
MuTect−Battenberg
MuTect−TITAN
N
RB1
PTEN
PGA
SNVs
0
10
20
30
40
50
60
70
1000
2000
3000
4000
5000
6000
7000
22%
66%
19%
26%
1
2

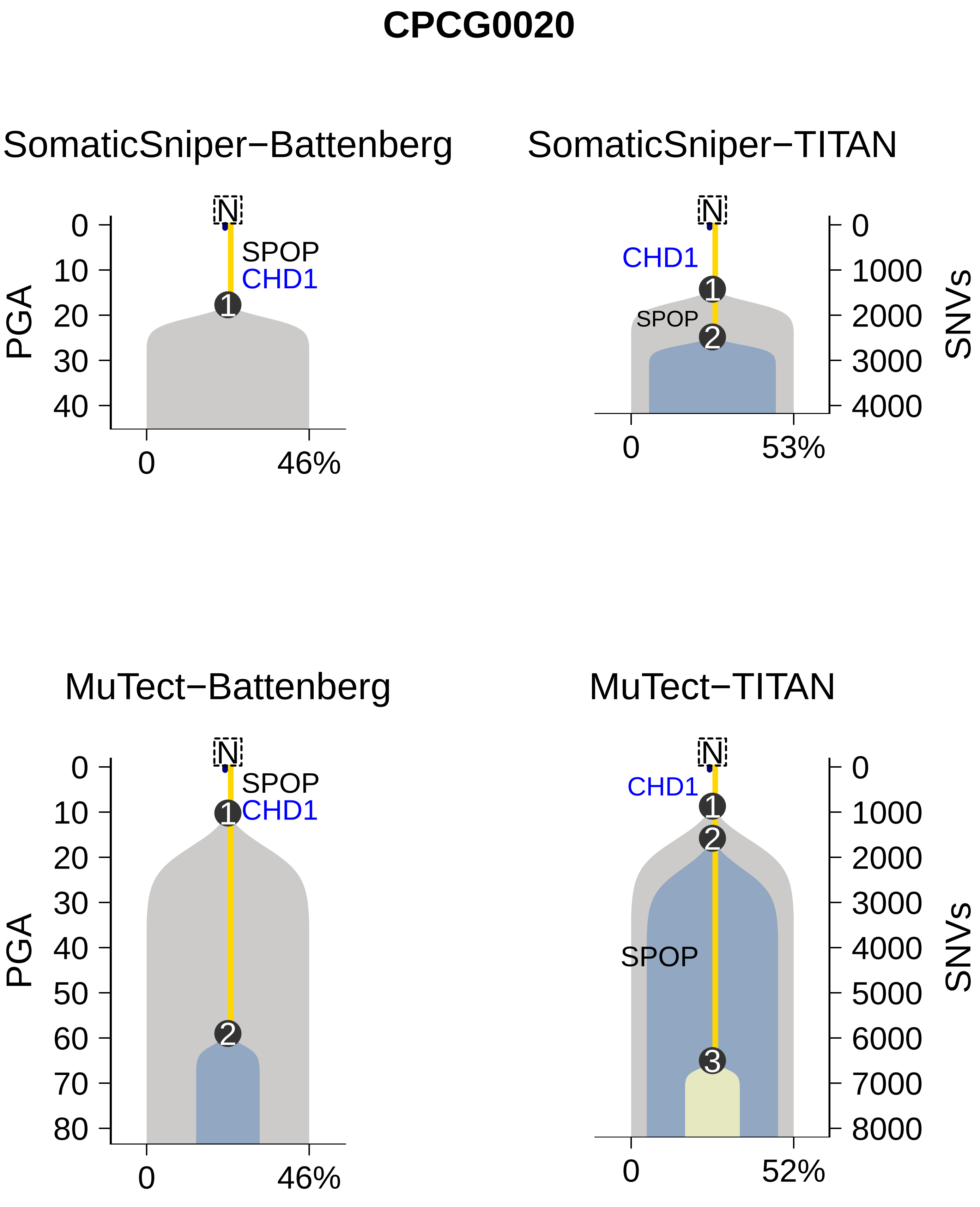
CPCG0020
SomaticSniper−Battenberg
SomaticSniper−TITAN
MuTect−Battenberg
MuTect−TITAN
N
SPOP
CHD1
PGA
SNVs
0
10
20
30
40
50
60
70
80
0
1000
2000
3000
4000
5000
6000
7000
8000
46%
53%
52%
1
2
3

CPCG0022

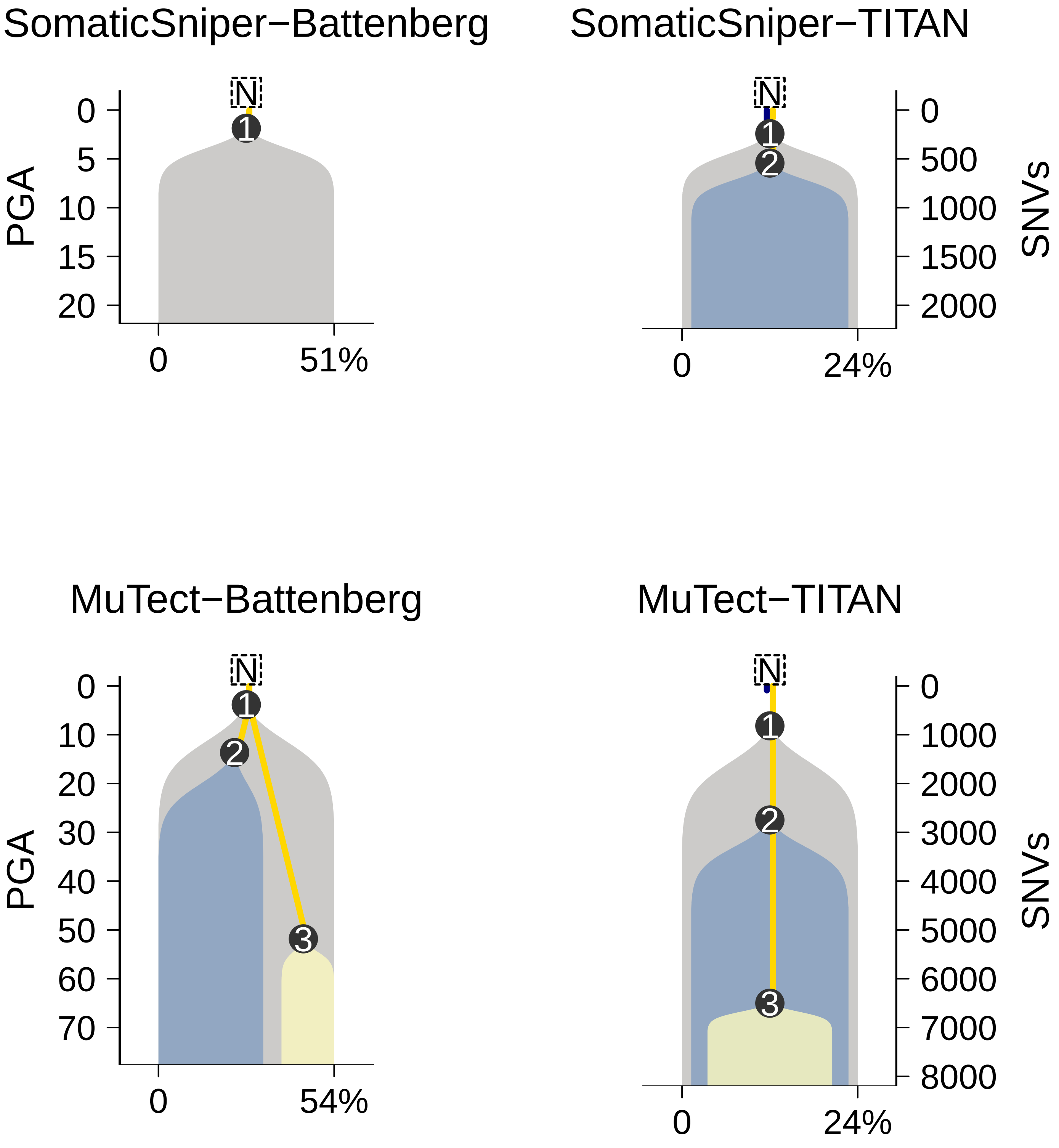
SomaticSniper−Battenberg
SomaticSniper−TITAN
MuTect−Battenberg
MuTect−TITAN
PGA
SNVs
N
1
2
3
0
5
10
15
20
51%
500
1000
1500
2000
24%
30
40
50
60
70
54%
3000
4000
5000
6000
7000
8000

CPCG0027

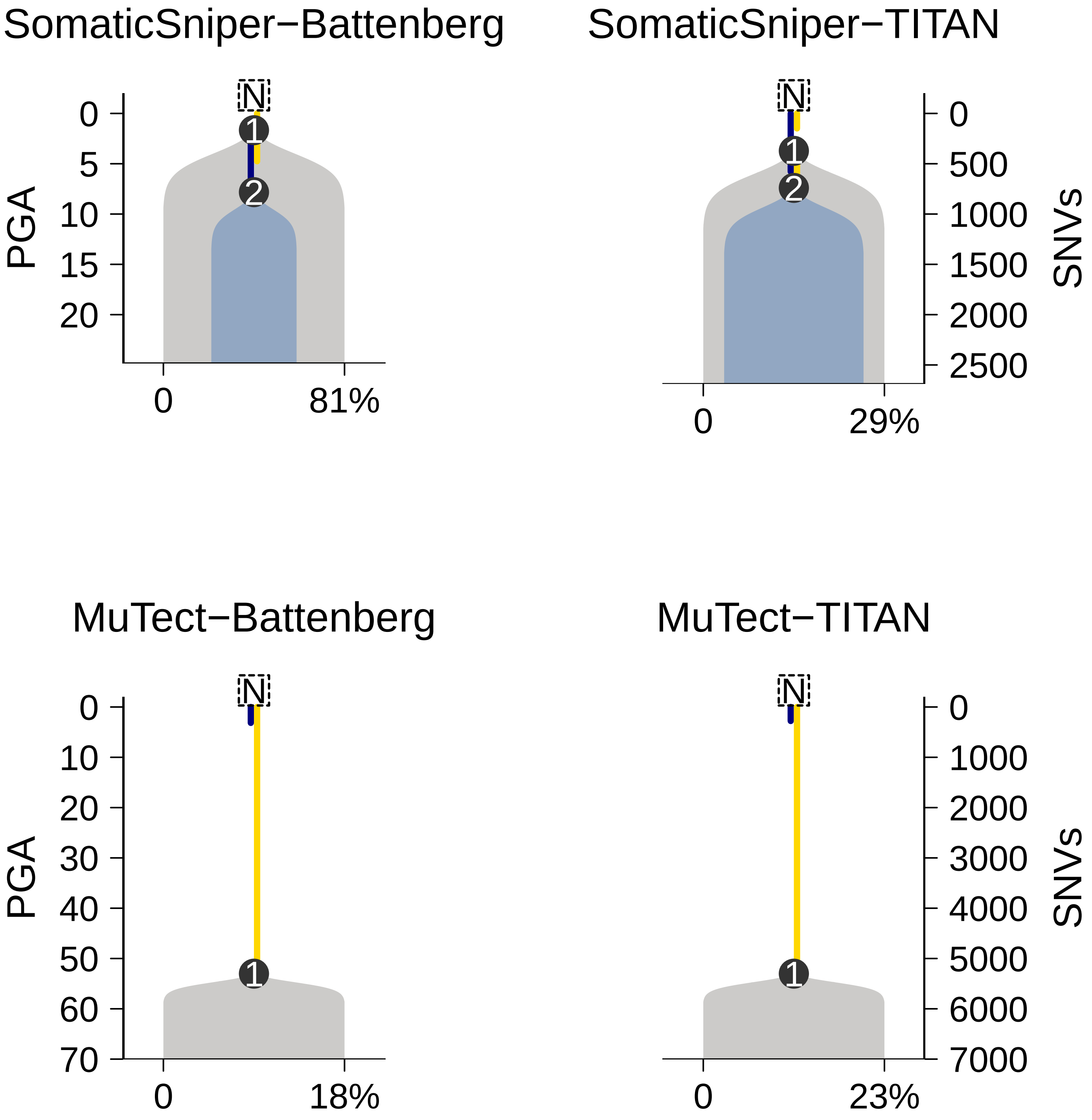
SomaticSniper−Battenberg
SomaticSniper−TITAN
MuTect−Battenberg
MuTect−TITAN
PGA
SNVs
N
1
2
0
5
10
15
20
81%
29%
18%
23%
500
1000
1500
2000
2500
30
40
50
60
70
3000
4000
5000
6000
7000

CPCG0030

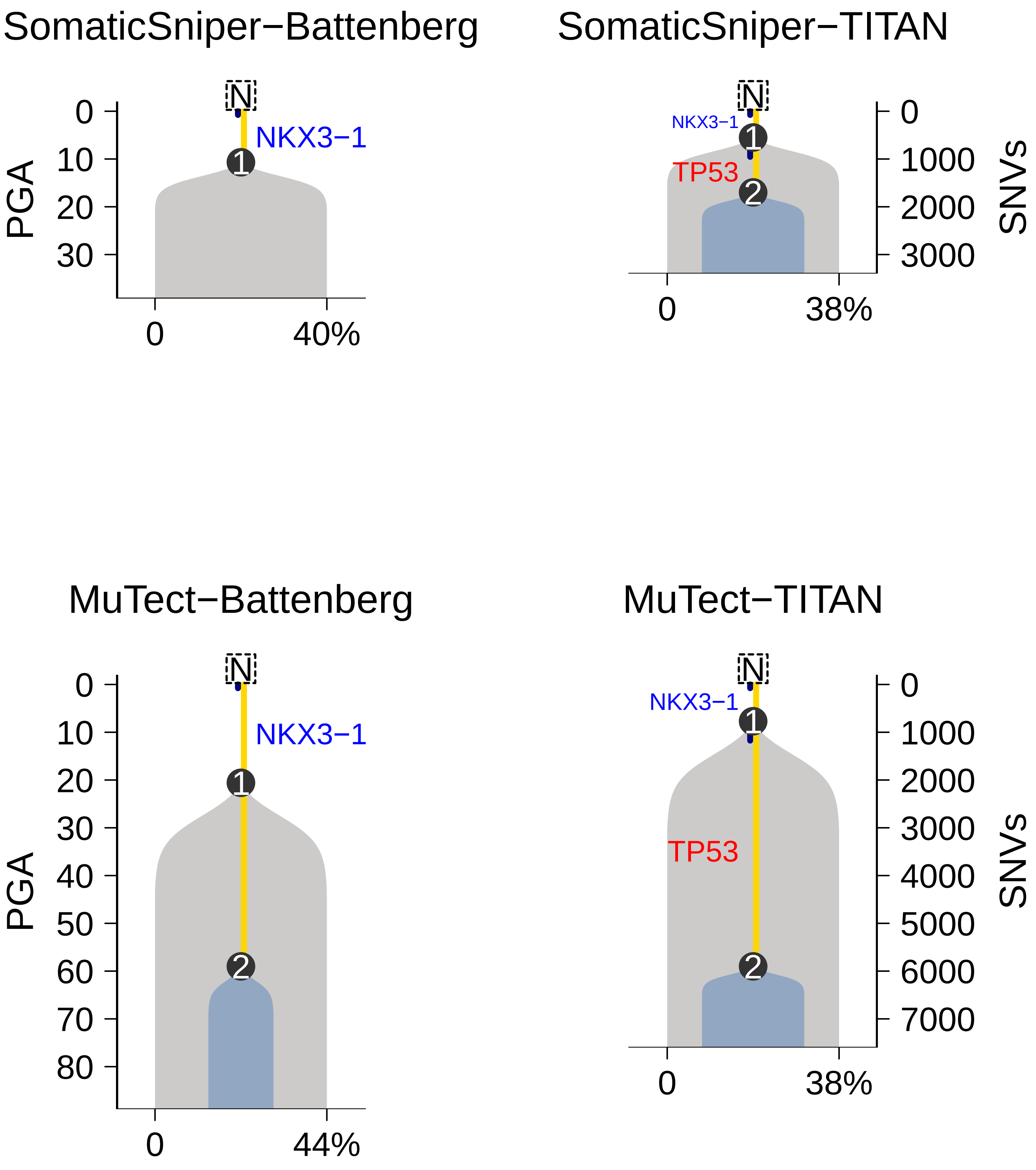

SomaticSniper−Battenberg
N
NKX3−1
1
PGA
0
10
20
30
0
40%
SomaticSniper−TITAN
N
NKX3−1
1
TP53
2
0
1000
2000
3000
SNVs
0
38%
MuTect−Battenberg
N
NKX3−1
1
2
PGA
0
10
20
30
40
50
60
70
80
0
44%
MuTect−TITAN
N
NKX3−1
1
TP53
2
0
1000
2000
3000
4000
5000
6000
7000
SNVs
0
38%

CPCG0040

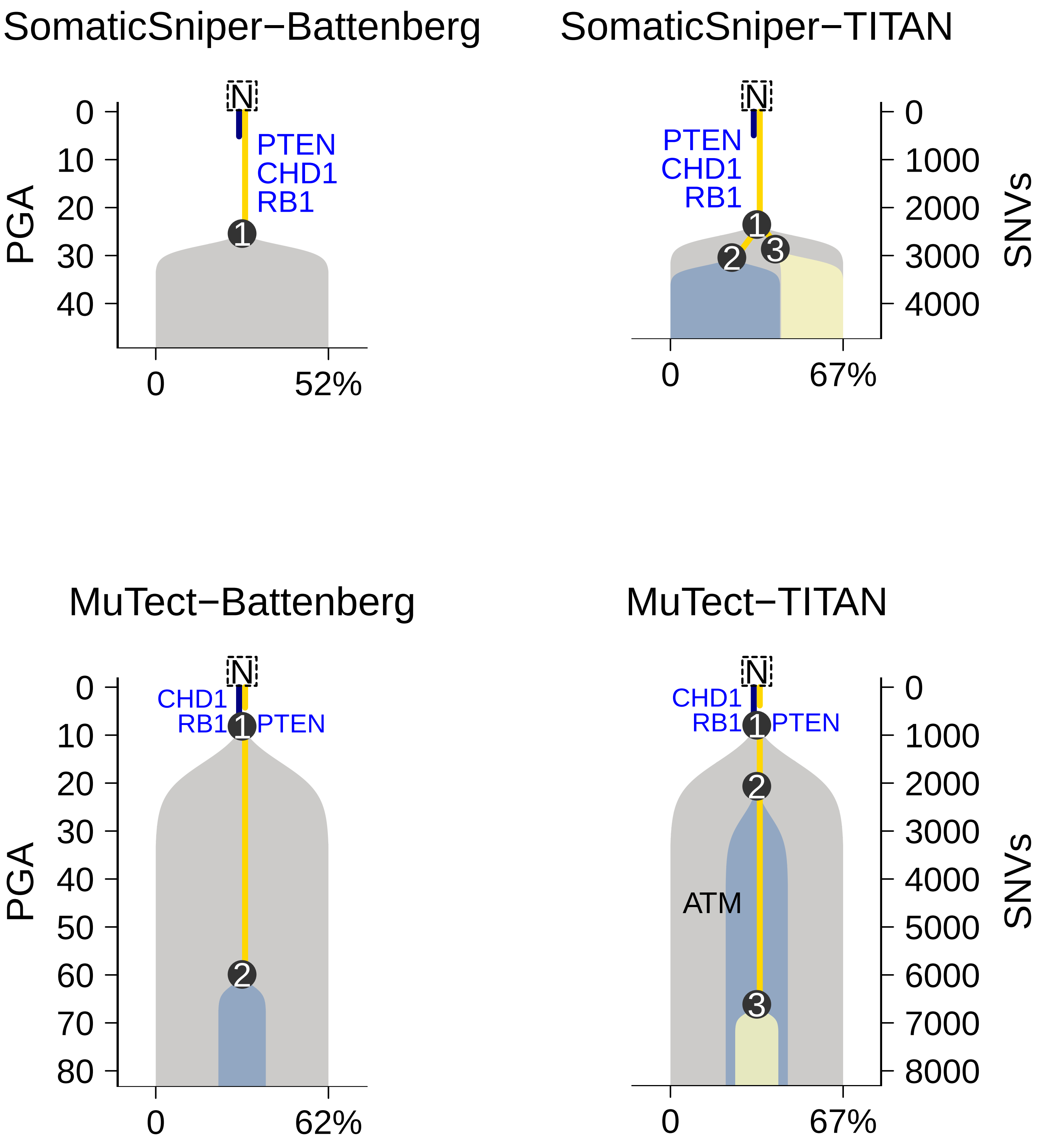

SomaticSniper−Battenberg
SomaticSniper−TITAN
MuTect−Battenberg
MuTect−TITAN
N
PTEN
CHD1
RB1
ATM
PGA
SNVs
0
10
20
30
40
50
60
70
80
1000
2000
3000
4000
5000
6000
7000
8000
52%
62%
67%

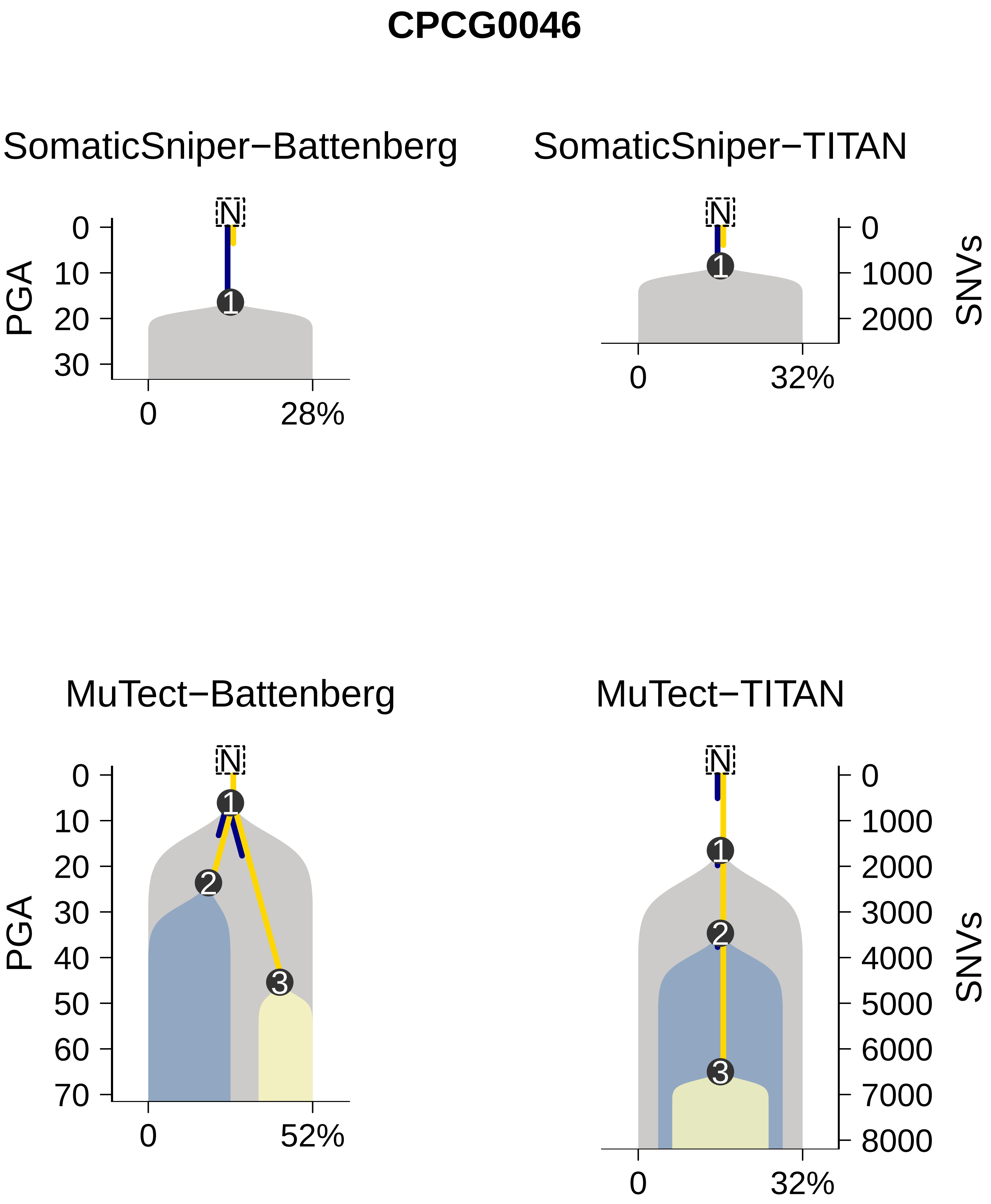
CPCG0046
SomaticSniper−Battenberg
SomaticSniper−TITAN
MuTect−Battenberg
MuTect−TITAN
N
1
2
3
PGA
SNVs
0
10
20
30
40
50
60
70
1000
2000
3000
4000
5000
6000
7000
8000
28%
32%
52%

CPCG0047

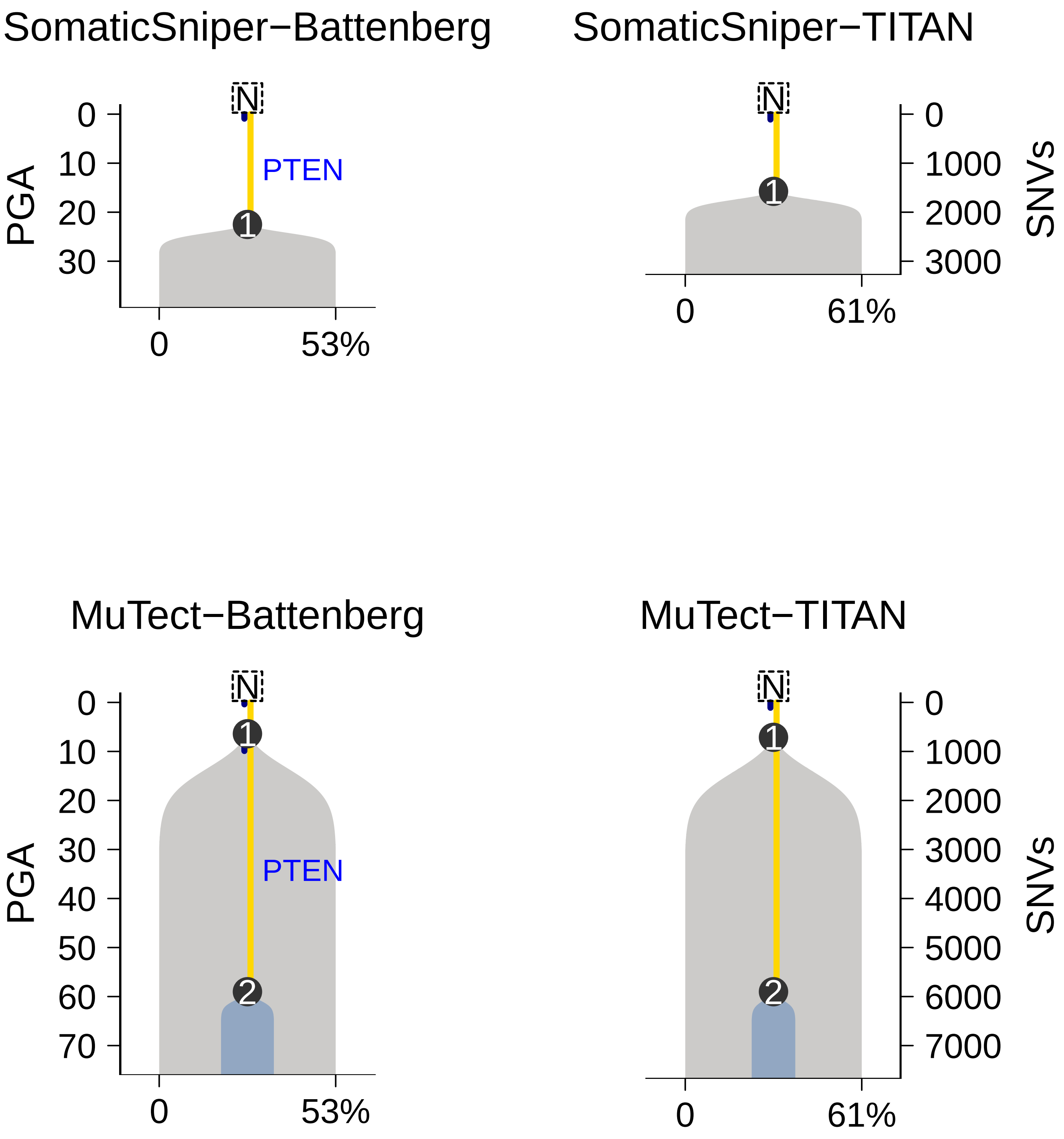

SomaticSniper−Battenberg
SomaticSniper−TITAN
MuTect−Battenberg
MuTect−TITAN
N
1
2
PTEN
PGA
SNVs
0
10
20
30
40
50
60
70
1000
2000
3000
4000
5000
6000
7000
53%
61%

CPCG0048

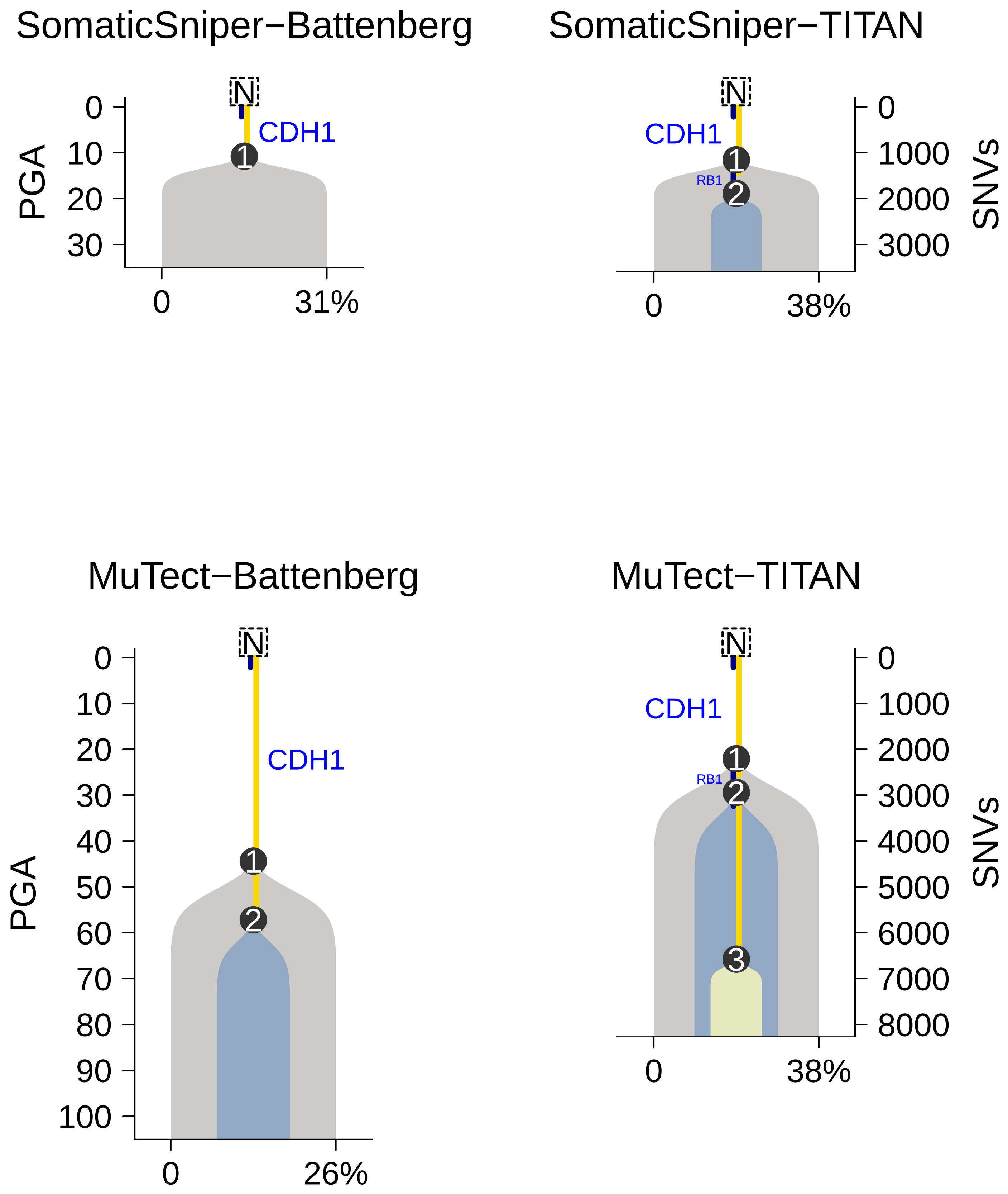
SomaticSniper−Battenberg
SomaticSniper−TITAN
MuTect−Battenberg
MuTect−TITAN
N
CDH1
RB1
1
2
3
PGA
SNVs
0
10
20
30
40
50
60
70
80
90
100
1000
2000
3000
4000
5000
6000
7000
8000
31%
38%
26%

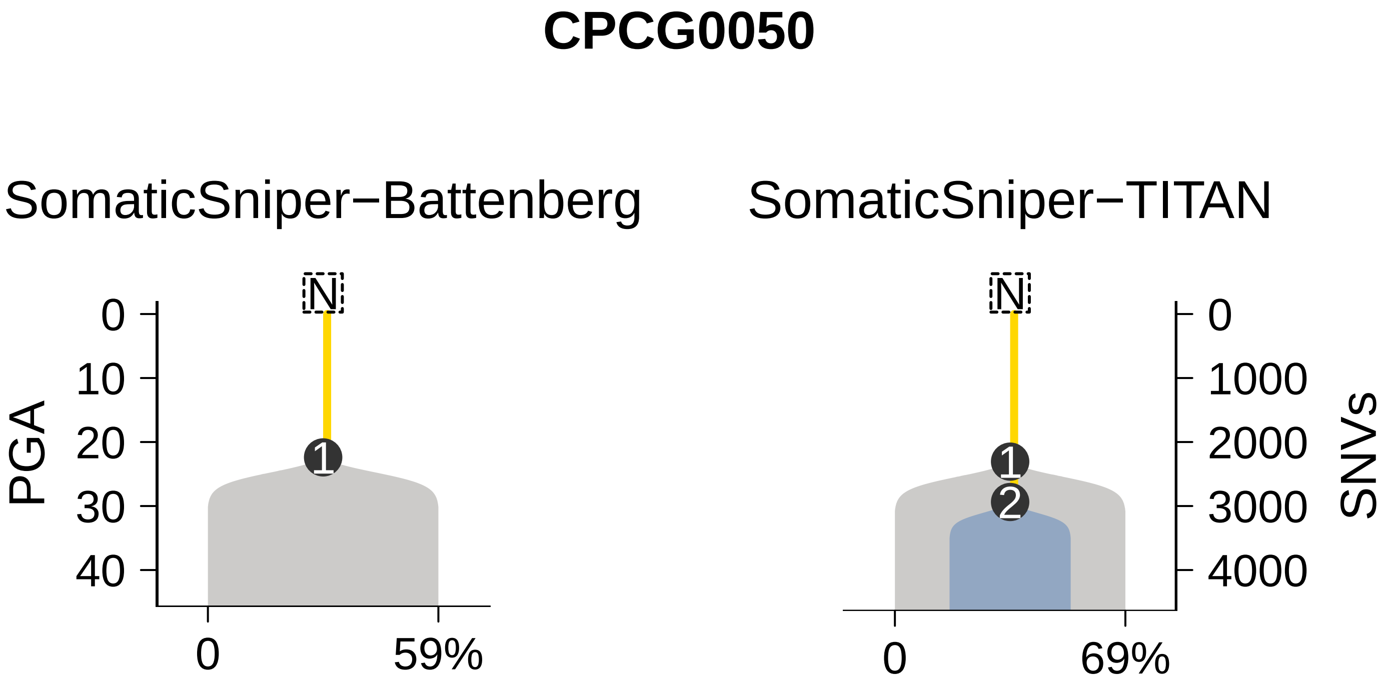

CPCG0050
SomaticSniper−Battenberg
SomaticSniper−TITAN
N
1
2
PGA
0
10
20
30
40
0
59%
0
69%
0
1000
2000
3000
4000
SNVs

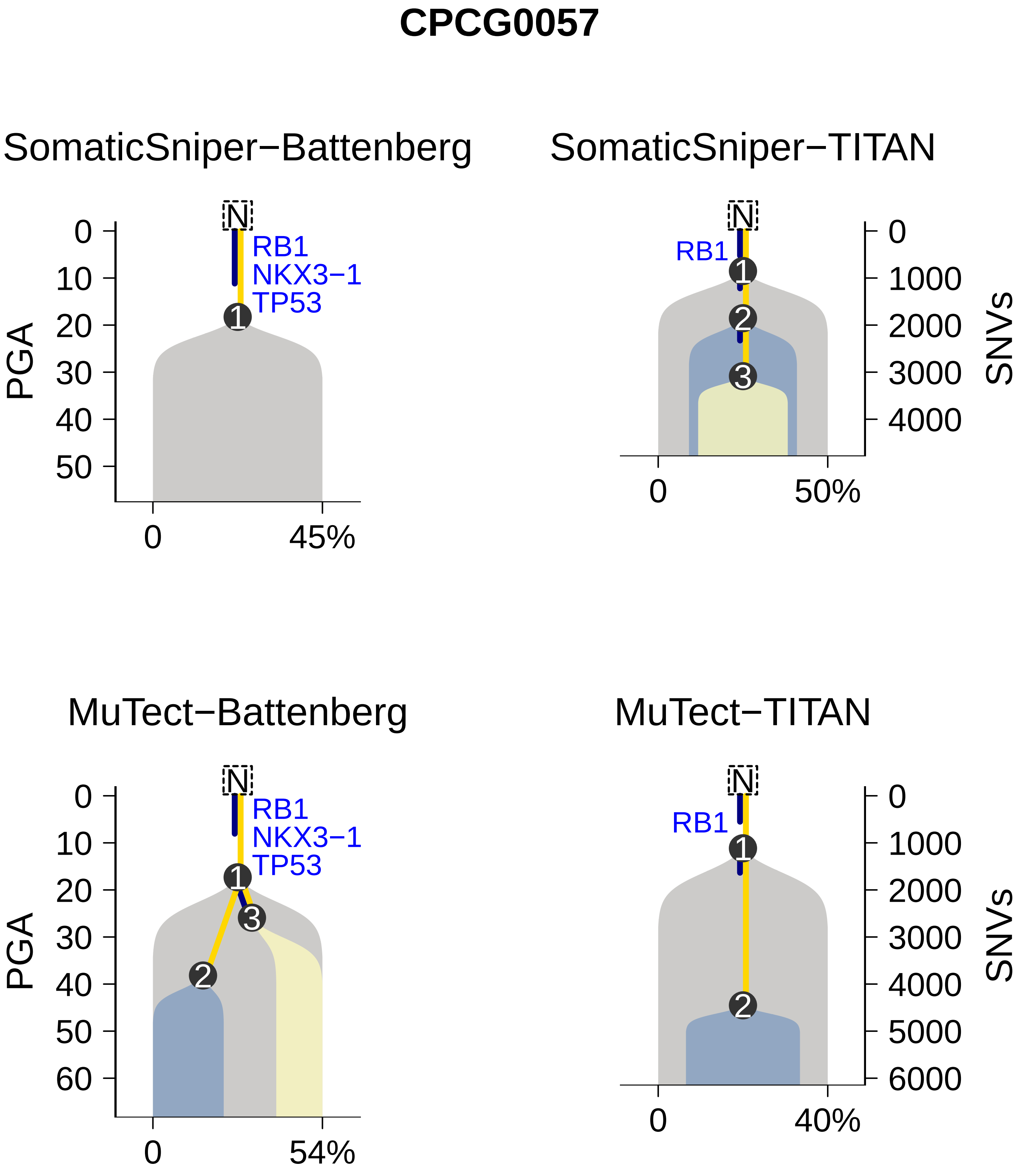
CPCG0057
SomaticSniper−Battenberg
SomaticSniper−TITAN
MuTect−Battenberg
MuTect−TITAN
N
RB1
NKX3−1
TP53
PGA
SNVs
0
10
20
30
40
50
60
1000
2000
3000
4000
5000
6000
45%
50%
54%
40%
1
2
3

CPCG0072

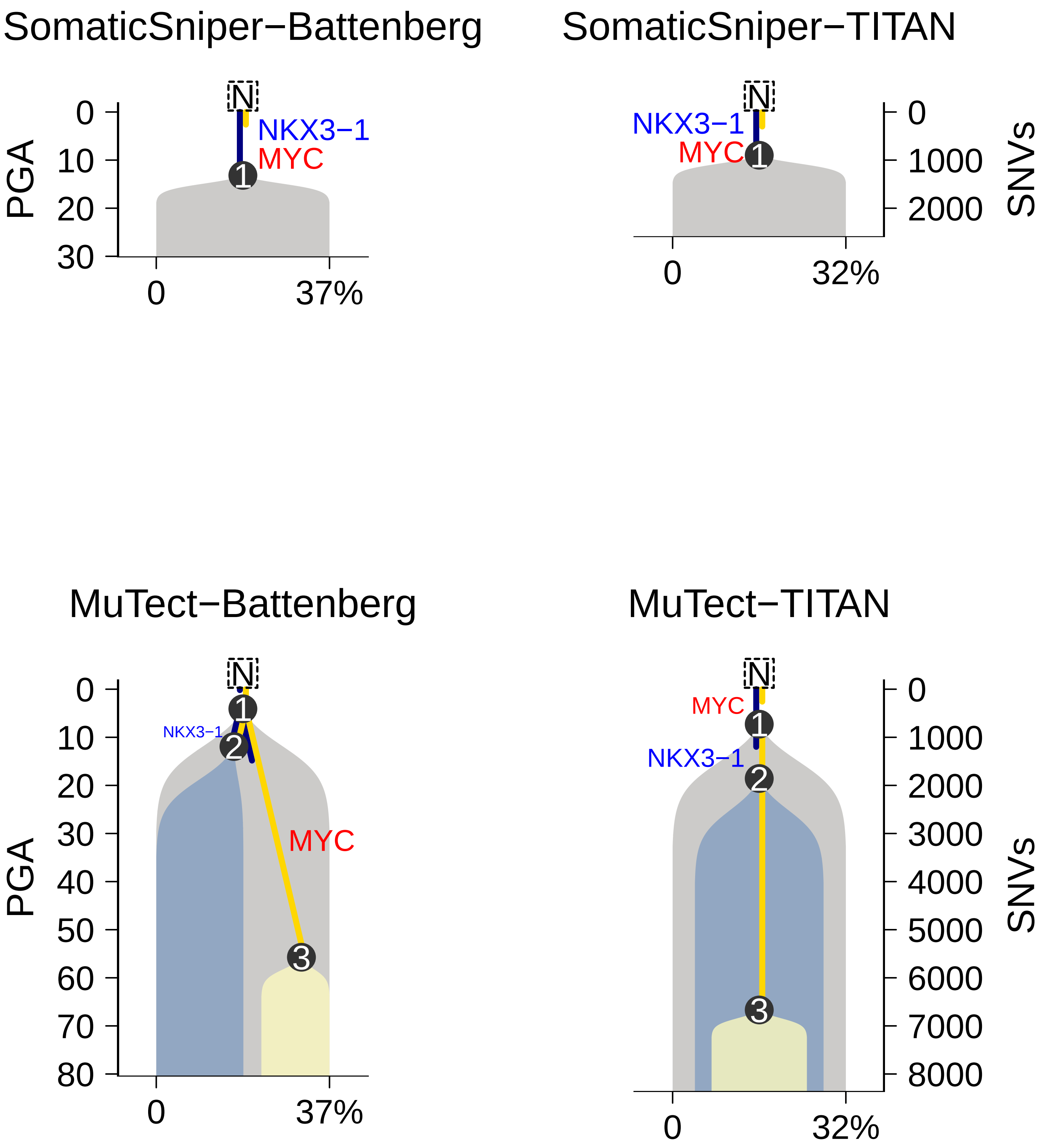

SomaticSniper−Battenberg
SomaticSniper−TITAN
MuTect−Battenberg
MuTect−TITAN
PGA
SNVs
N
NKX3−1
MYC
1
2
3
0
10
20
30
40
50
60
70
80
1000
2000
3000
4000
5000
6000
7000
8000
37%
32%

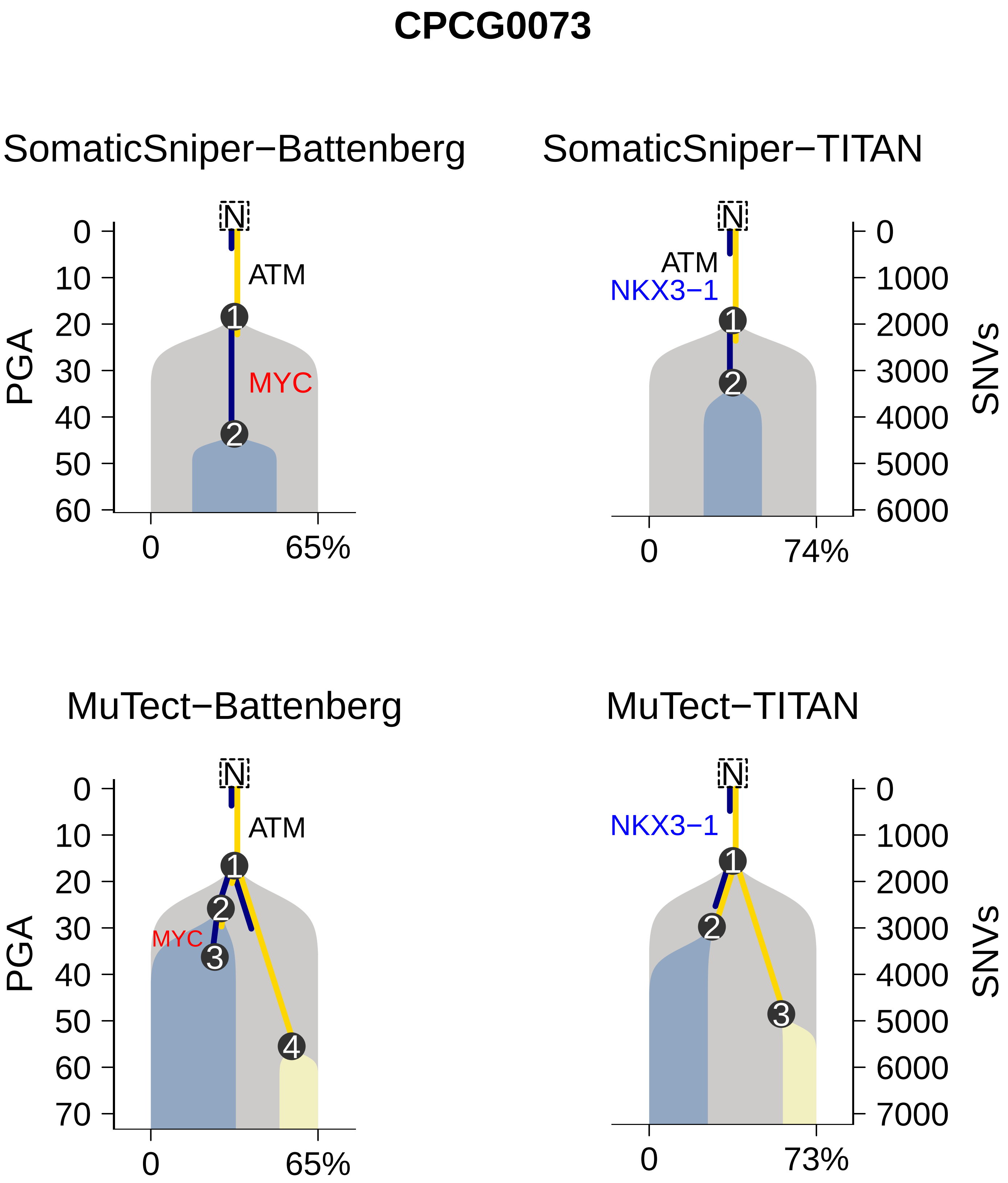

CPCG0073
SomaticSniper−Battenberg
SomaticSniper−TITAN
MuTect−Battenberg
MuTect−TITAN
PGA
SNVs
N
ATM
MYC
NKX3−1
0
65%
74%
73%

CPCG0075

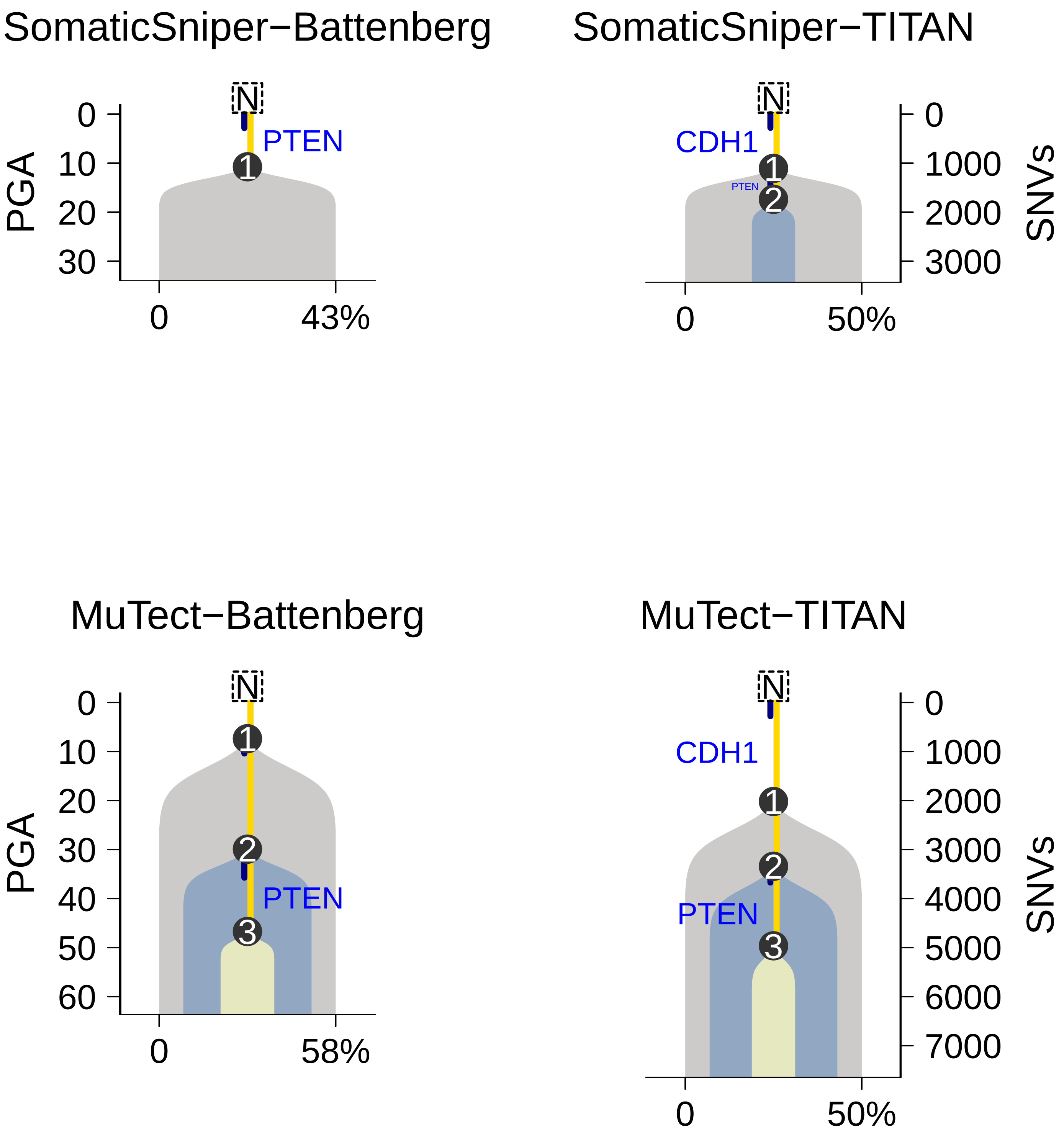

SomaticSniper−Battenberg
SomaticSniper−TITAN
MuTect−Battenberg
MuTect−TITAN
N
1
2
3
PTEN
CDH1
PGA
SNVs
0
10
20
30
40
50
60
1000
2000
3000
4000
5000
6000
7000
43%
50%
58%

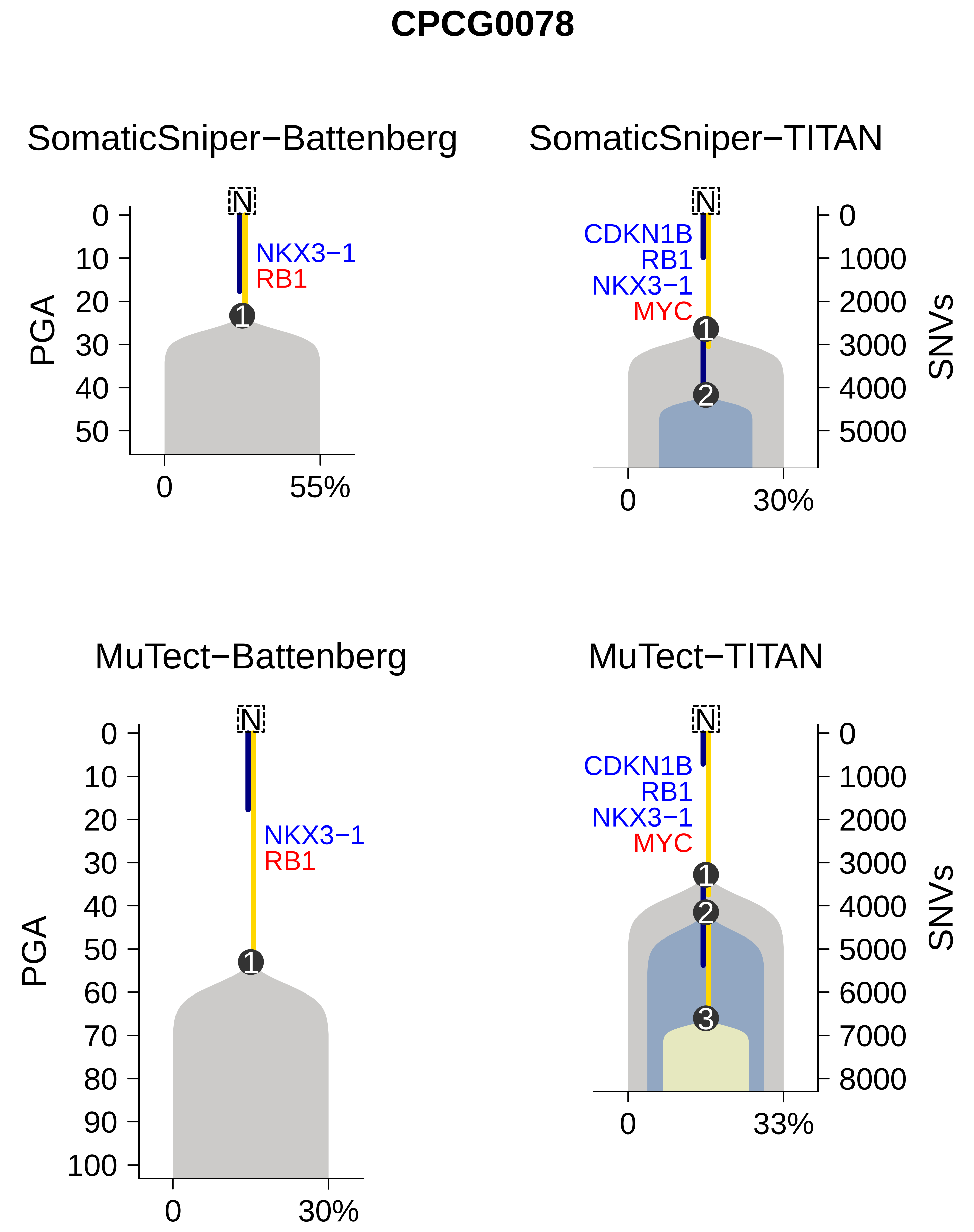
CPCG0078
SomaticSniper−Battenberg
N
NKX3−1
RB1
1
PGA
0
10
20
30
40
50
0
55%
SomaticSniper−TITAN
N
CDKN1B
RB1
NKX3−1
MYC
1
2
0
1000
2000
3000
4000
5000
SNVs
0
30%
MuTect−Battenberg
N
NKX3−1
RB1
1
PGA
0
10
20
30
40
50
60
70
80
90
100
0
30%
MuTect−TITAN
N
CDKN1B
RB1
NKX3−1
MYC
1
2
3
0
1000
2000
3000
4000
5000
6000
7000
8000
SNVs
0
33%

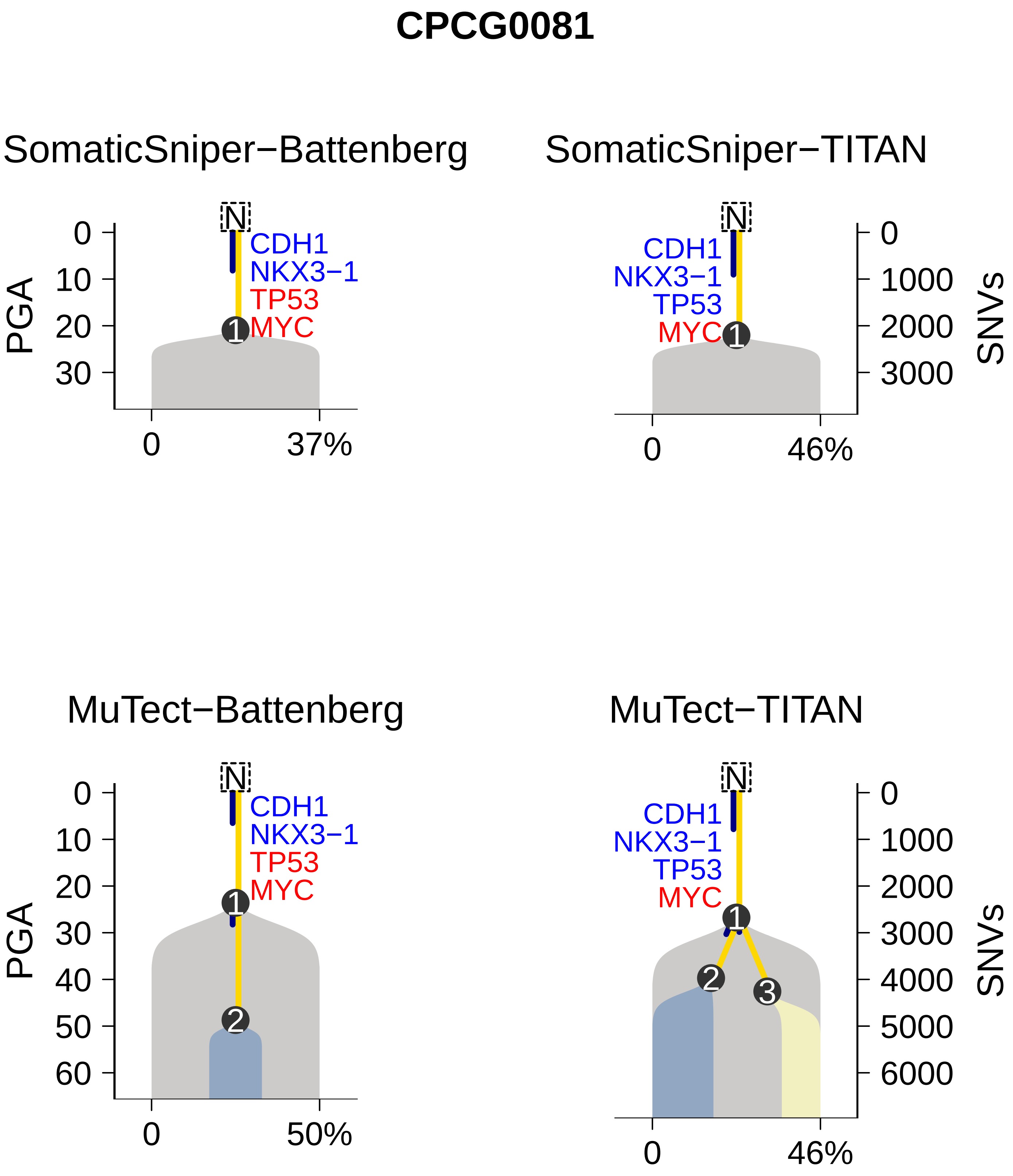
CPCG0081
SomaticSniper−Battenberg
SomaticSniper−TITAN
MuTect−Battenberg
MuTect−TITAN
PGA
SNVs
N
CDH1
NKX3−1
TP53
MYC
1
2
3
0
10
20
30
40
50
60
1000
2000
3000
4000
5000
6000
37%
46%
50%

CPCG0083

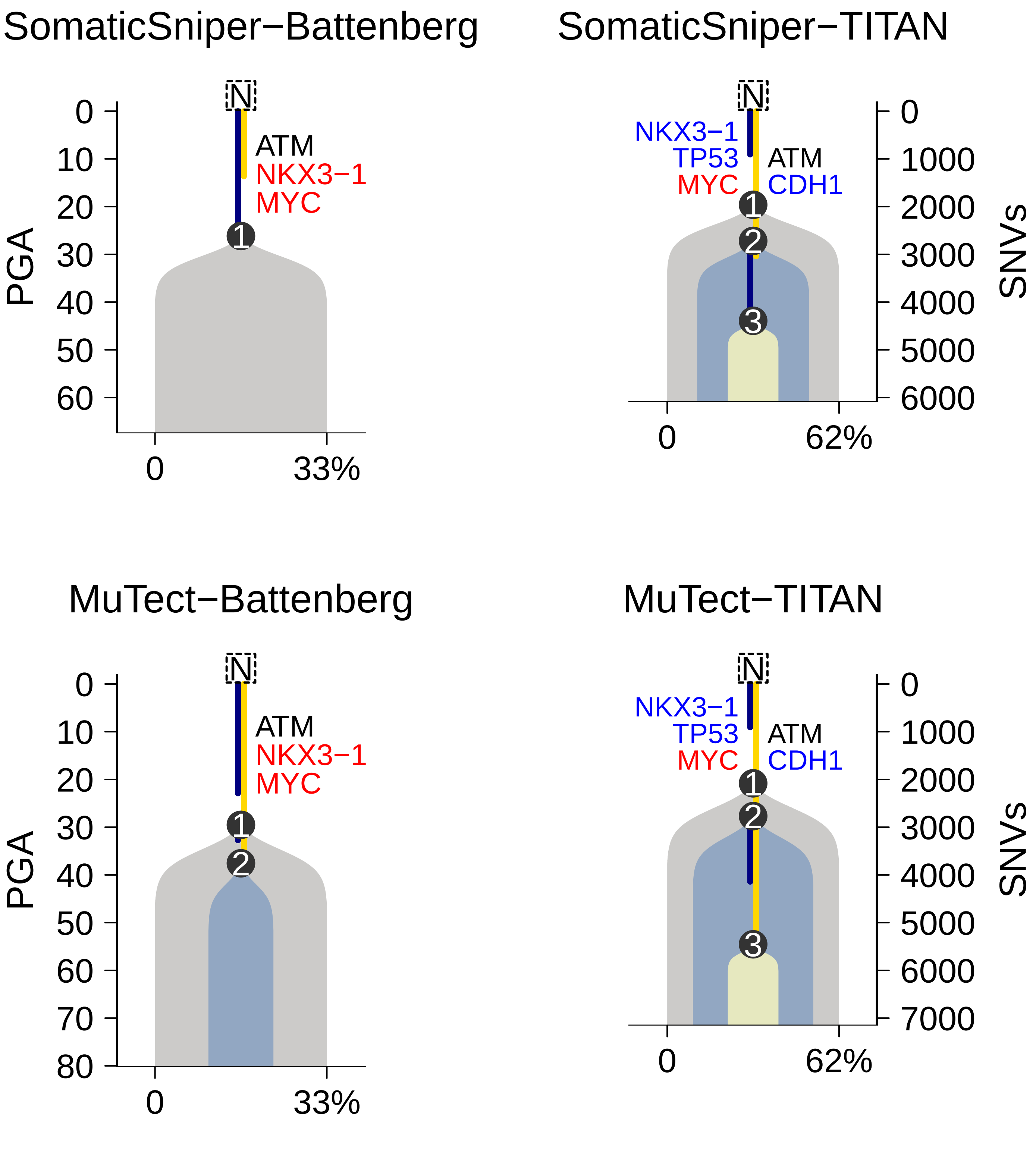

SomaticSniper−Battenberg
N
ATM
NKX3−1
MYC
1
PGA
0
10
20
30
40
50
60
0
33%
SomaticSniper−TITAN
N
NKX3−1
TP53
MYC
ATM
CDH1
1
2
3
0
1000
2000
3000
4000
5000
6000
SNVs
0
62%
MuTect−Battenberg
N
ATM
NKX3−1
MYC
1
2
PGA
0
10
20
30
40
50
60
70
80
0
33%
MuTect−TITAN
N
NKX3−1
TP53
MYC
ATM
CDH1
1
2
3
0
1000
2000
3000
4000
5000
6000
7000
SNVs
0
62%

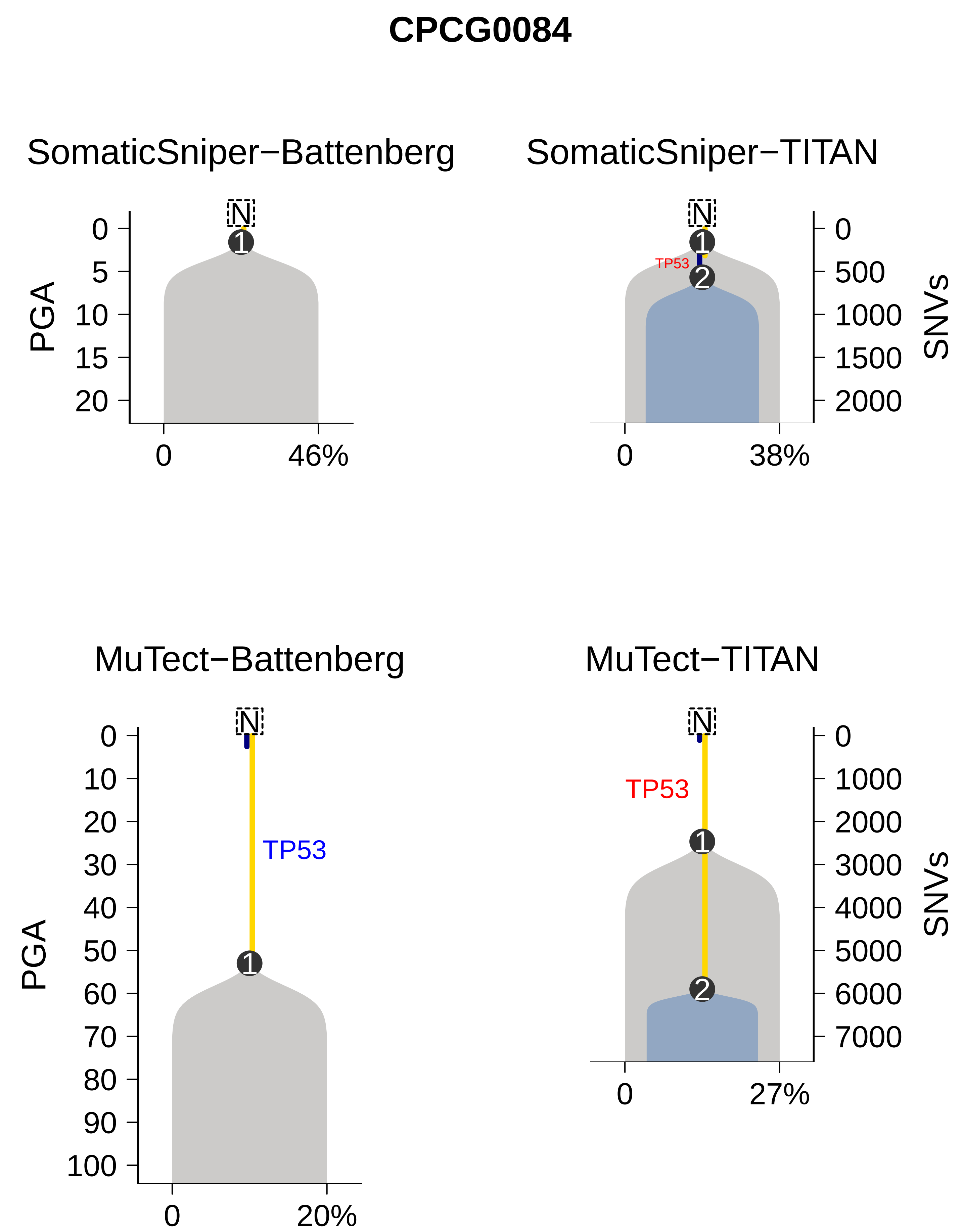
CPCG0084
SomaticSniper−Battenberg
SomaticSniper−TITAN
MuTect−Battenberg
MuTect−TITAN
N
1
2
TP53
PGA
SNVs
0
5
10
15
20
500
1000
1500
2000
30
40
50
60
70
80
90
100
3000
4000
5000
6000
7000
46%
38%
20%
27%

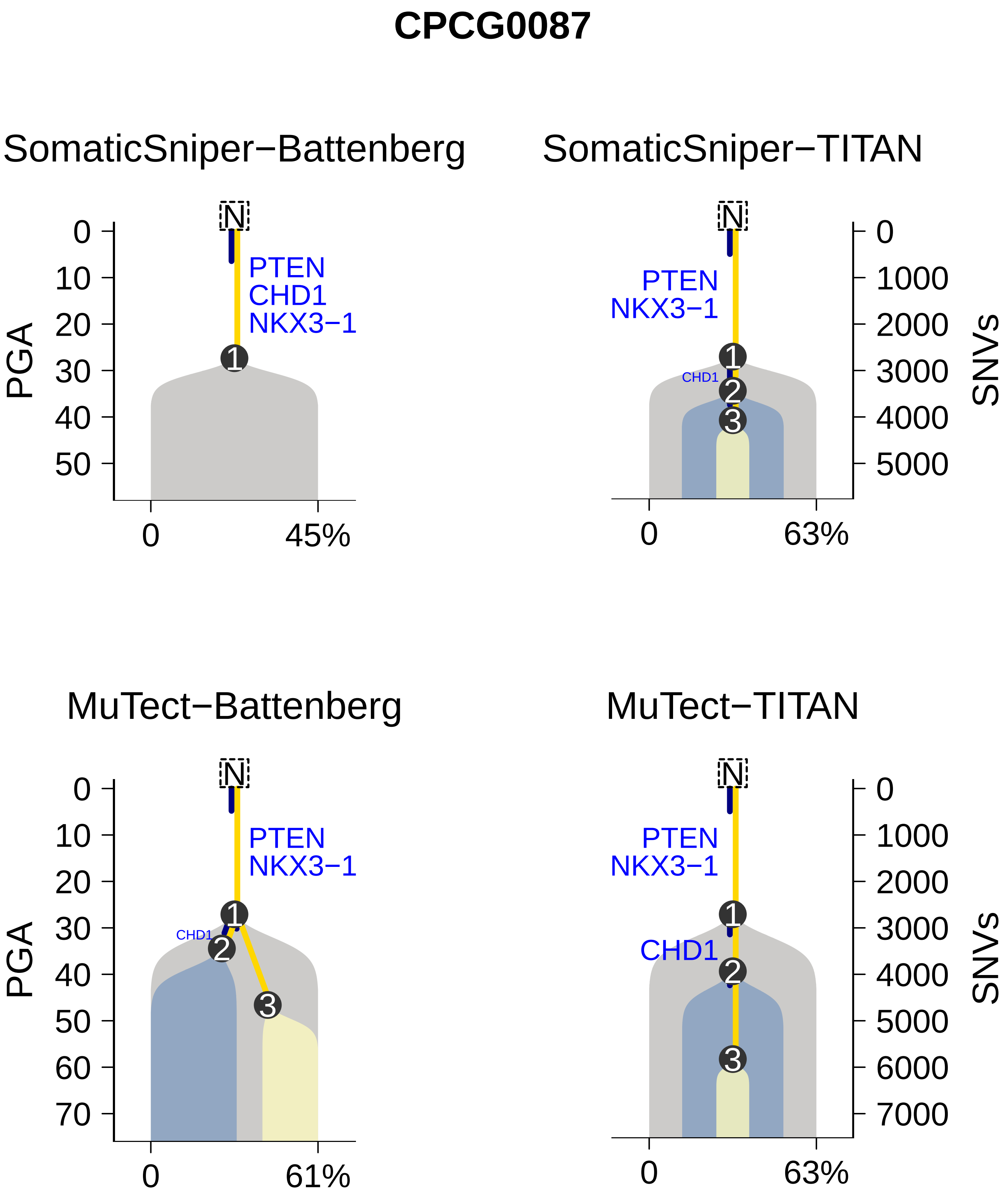
CPCG0087
SomaticSniper−Battenberg
SomaticSniper−TITAN
MuTect−Battenberg
MuTect−TITAN
PGA
SNVs
N
PTEN
CHD1
NKX3−1
1
2
3
0
10
20
30
40
50
60
70
1000
2000
3000
4000
5000
6000
7000
45%
63%
61%

CPCG0089

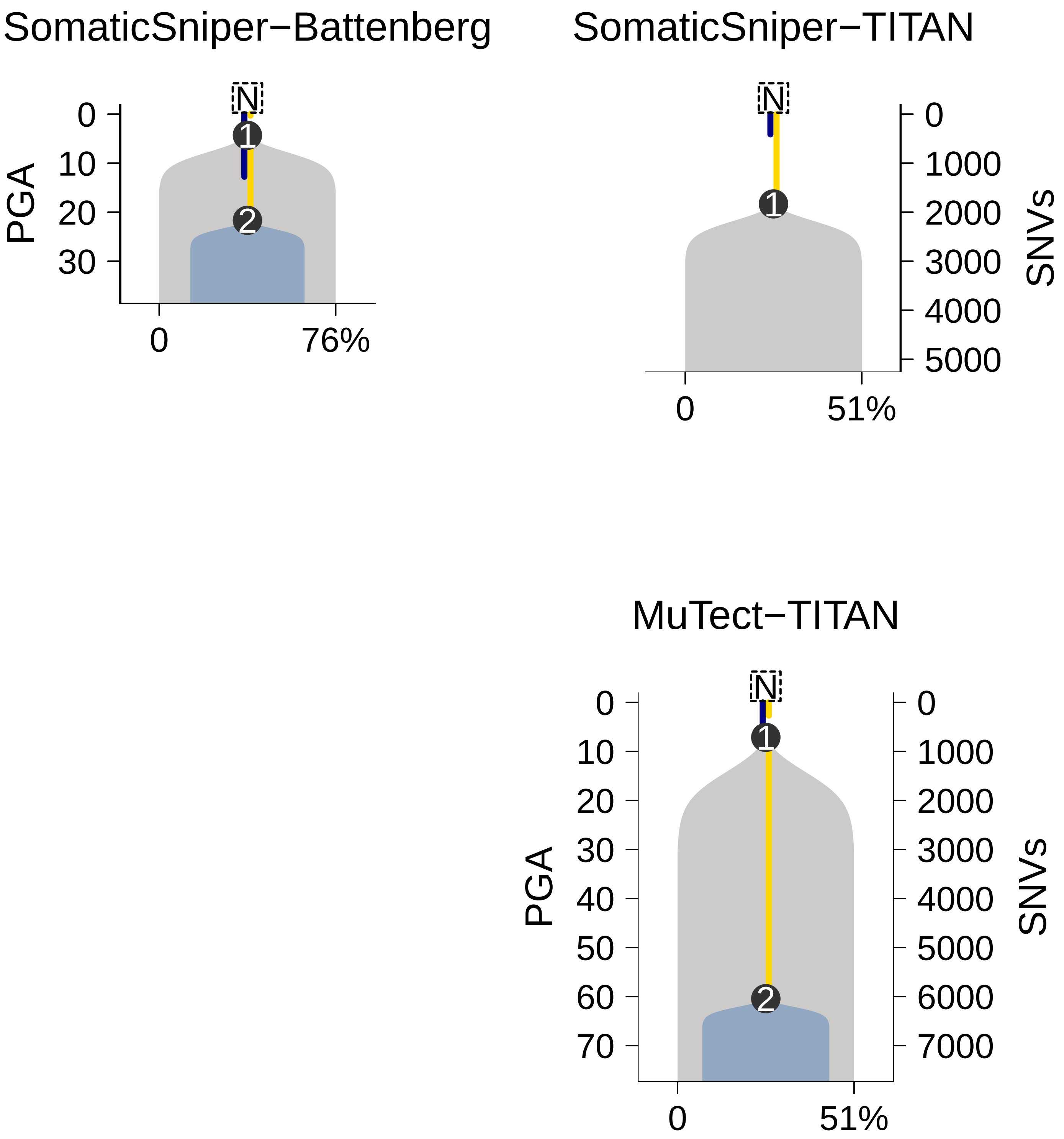

SomaticSniper−Battenberg
SomaticSniper−TITAN
MuTect−TITAN
N
1
2
PGA
SNVs
0
10
20
30
40
50
60
70
1000
2000
3000
4000
5000
6000
7000
76%
51%

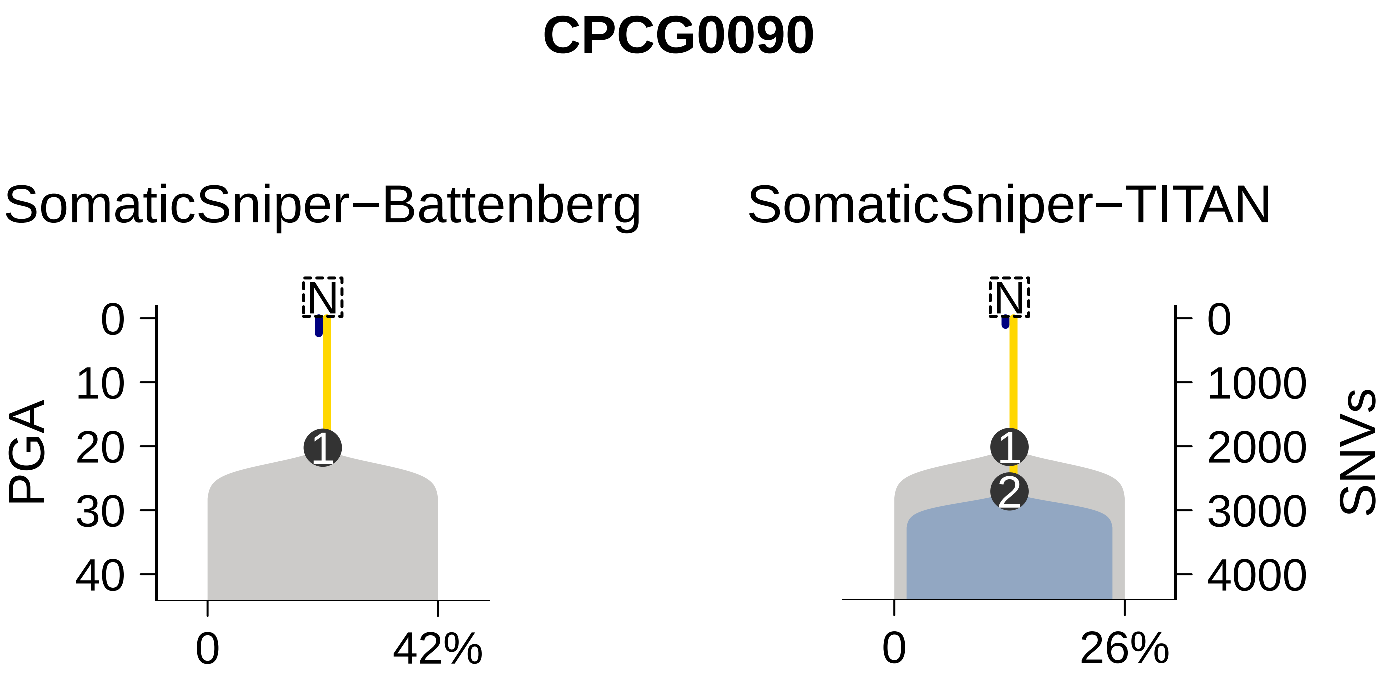
CPCG0090
SomaticSniper−Battenberg
SomaticSniper−TITAN
N
1
2
PGA
0
10
20
30
40
42%
26%
SNVs
1000
2000
3000
4000

CPCG0094

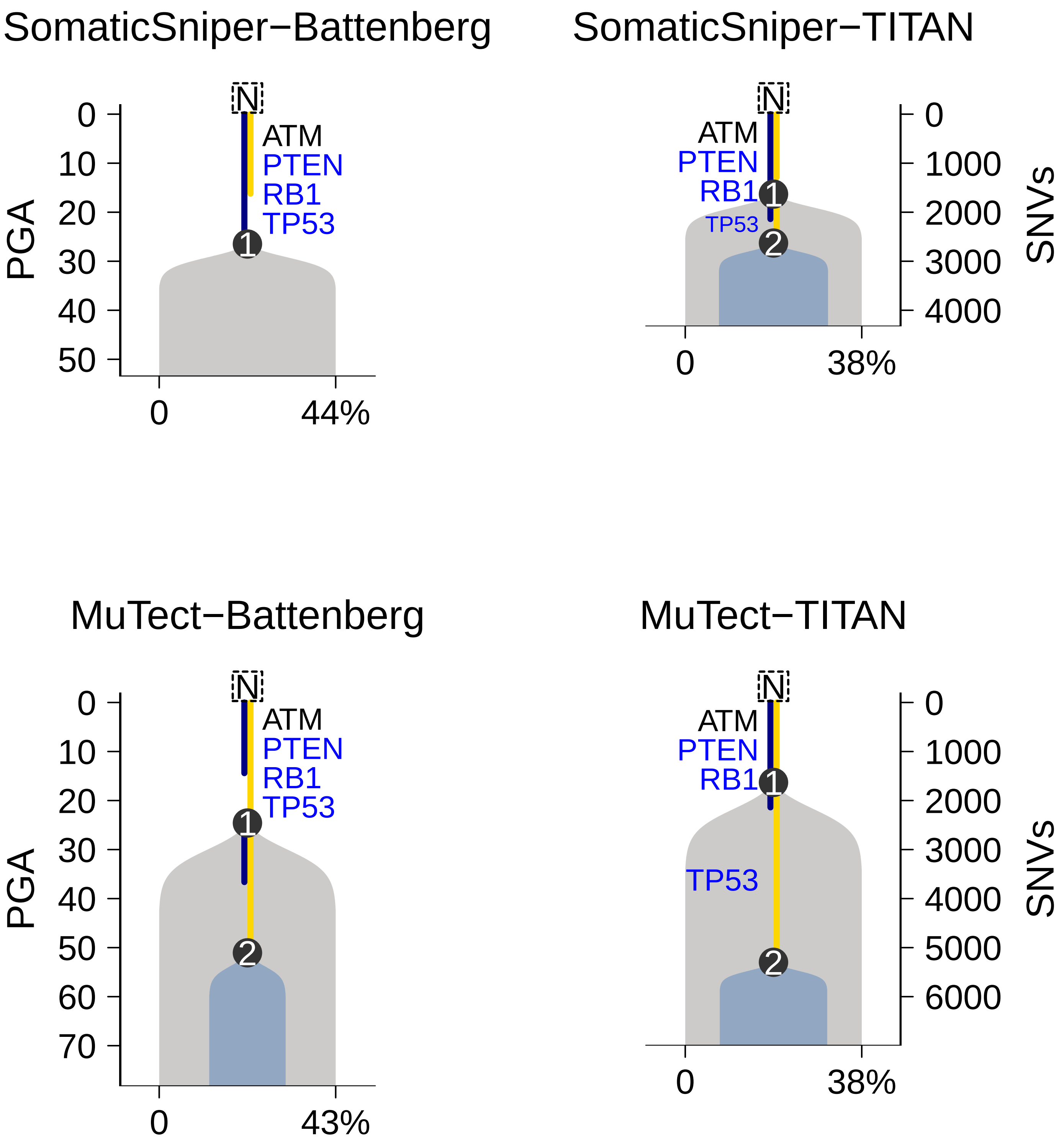

SomaticSniper−Battenberg
SomaticSniper−TITAN
MuTect−Battenberg
MuTect−TITAN
N
ATM
PTEN
RB1
TP53
1
2
PGA
SNVs
0
10
20
30
40
50
60
70
1000
2000
3000
4000
5000
6000
44%
38%
43%

CPCG0095

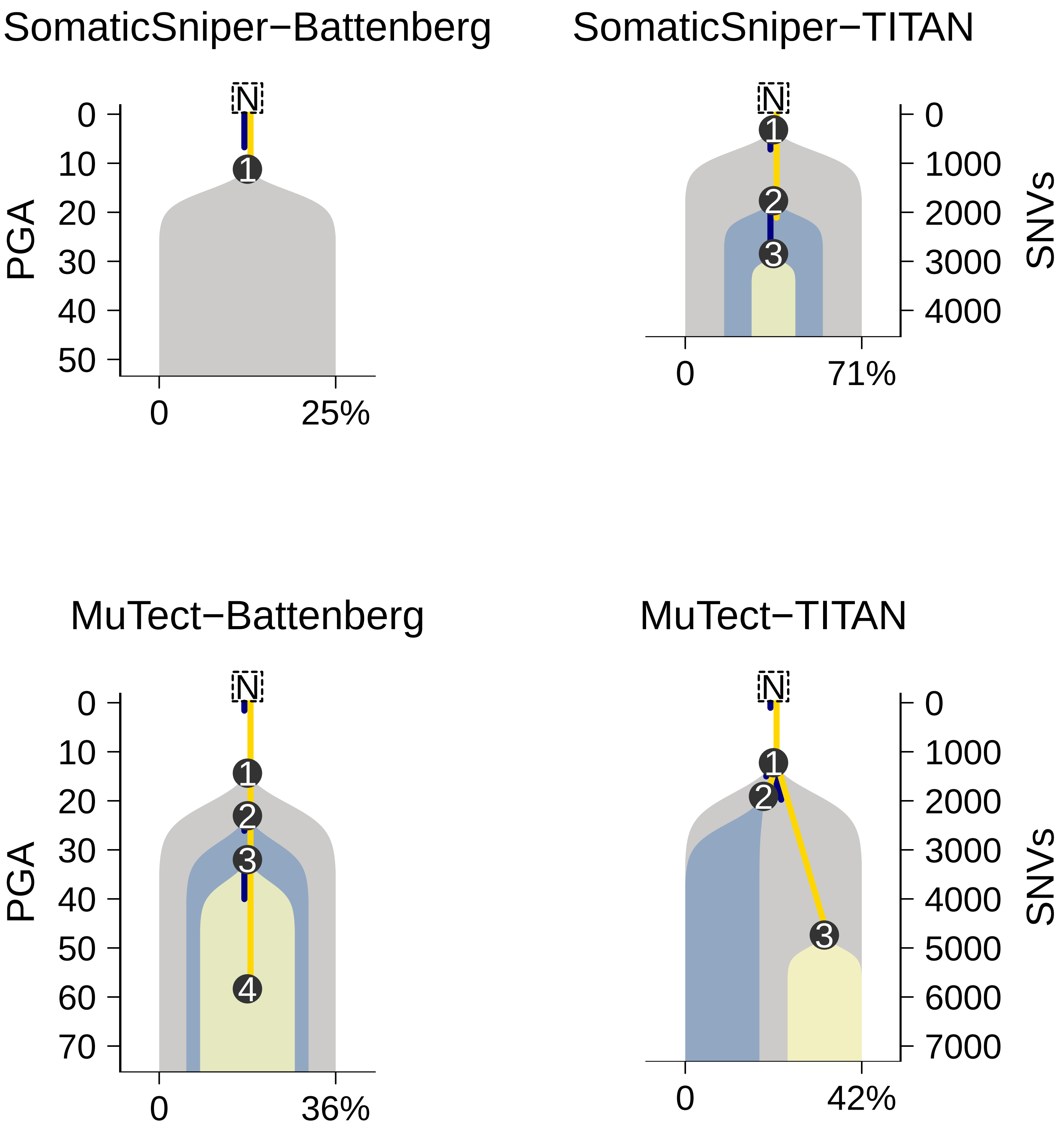

SomaticSniper−Battenberg
SomaticSniper−TITAN
MuTect−Battenberg
MuTect−TITAN
N
1
2
3
4
PGA
SNVs
0
10
20
30
40
50
60
70
1000
2000
3000
4000
5000
6000
7000
25%
71%
36%
42%

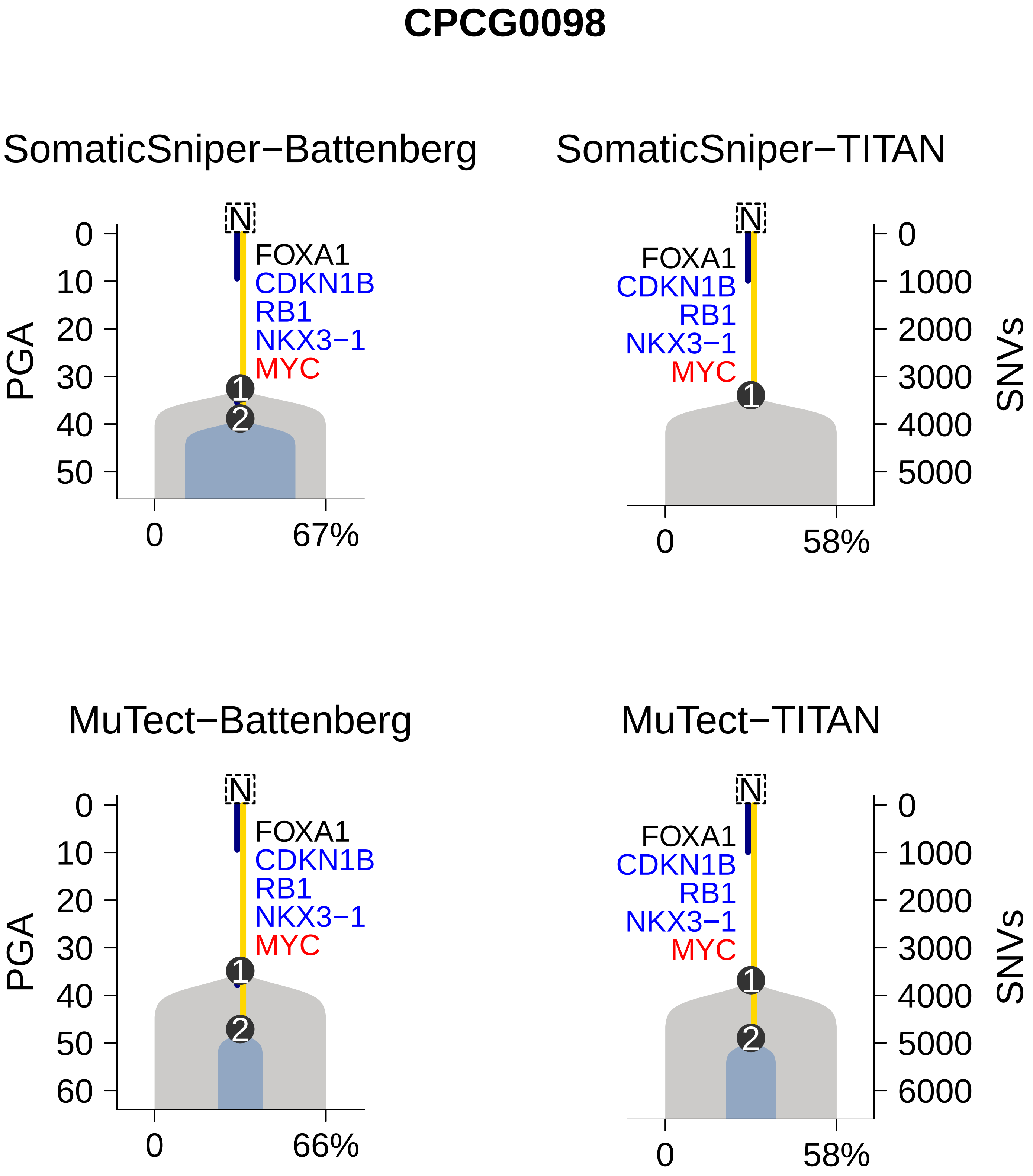
CPCG0098
SomaticSniper−Battenberg
SomaticSniper−TITAN
MuTect−Battenberg
MuTect−TITAN
N
FOXA1
CDKN1B
RB1
NKX3−1
MYC
1
2
PGA
SNVs
0
10
20
30
40
50
60
1000
2000
3000
4000
5000
6000
67%
58%
66%

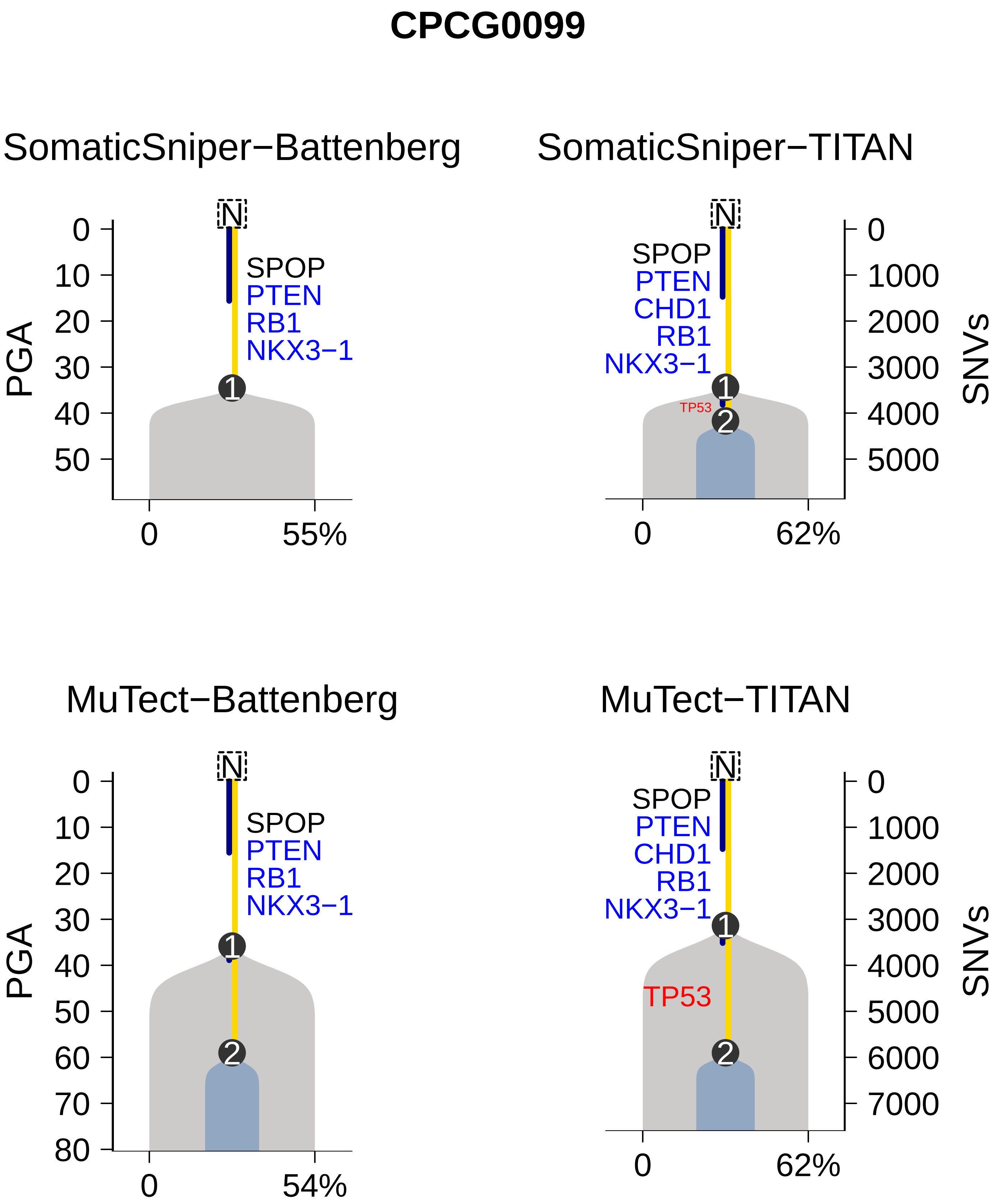
CPCG0099
SomaticSniper−Battenberg
SomaticSniper−TITAN
MuTect−Battenberg
MuTect−TITAN
N
1
2
SPOP
PTEN
CHD1
RB1
NKX3−1
TP53
PGA
SNVs
0
10
20
30
40
50
60
70
80
1000
2000
3000
4000
5000
6000
7000
55%
62%
54%

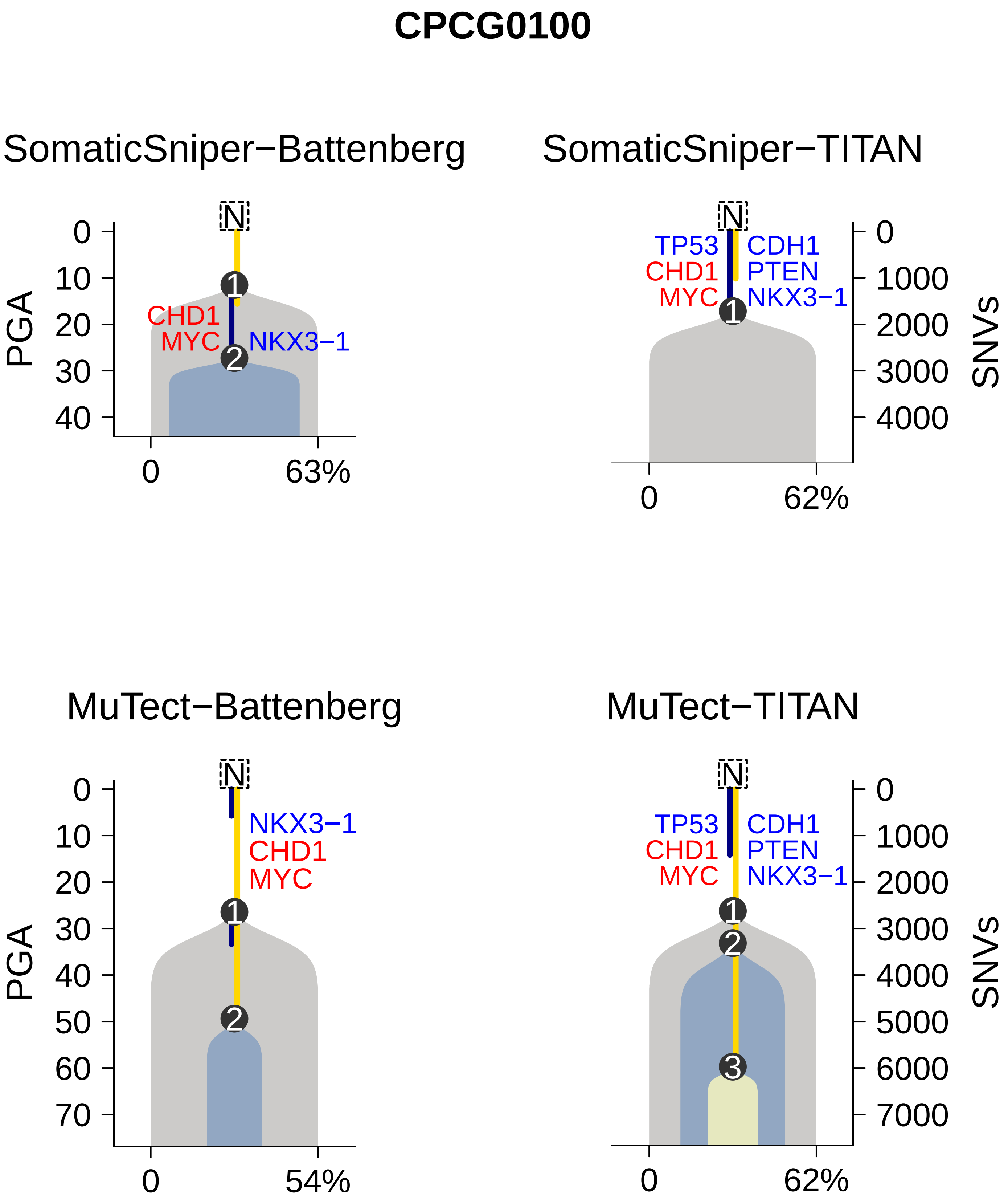
CPCG0100
SomaticSniper−Battenberg
SomaticSniper−TITAN
MuTect−Battenberg
MuTect−TITAN
N
1
2
3
CHD1
MYC
NKX3−1
TP53
CDH1
PTEN
PGA
SNVs
0
10
20
30
40
50
60
70
1000
2000
3000
4000
5000
6000
7000
63%
62%
54%

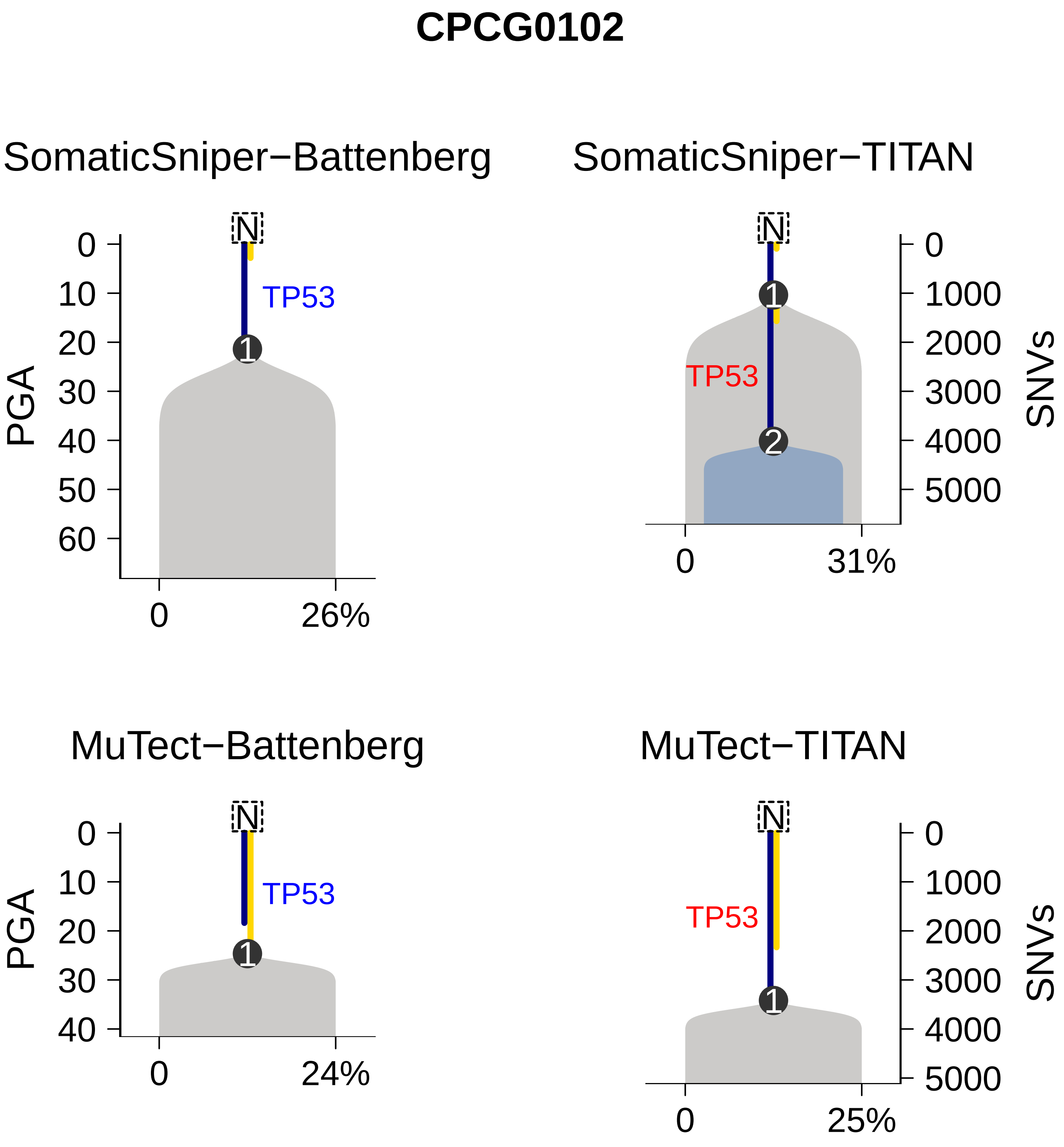
CPCG0102
SomaticSniper−Battenberg
SomaticSniper−TITAN
MuTect−Battenberg
MuTect−TITAN
N
1
2
TP53
PGA
SNVs
0
10
20
30
40
50
60
1000
2000
3000
4000
5000
26%
31%
24%
25%

CPCG0114

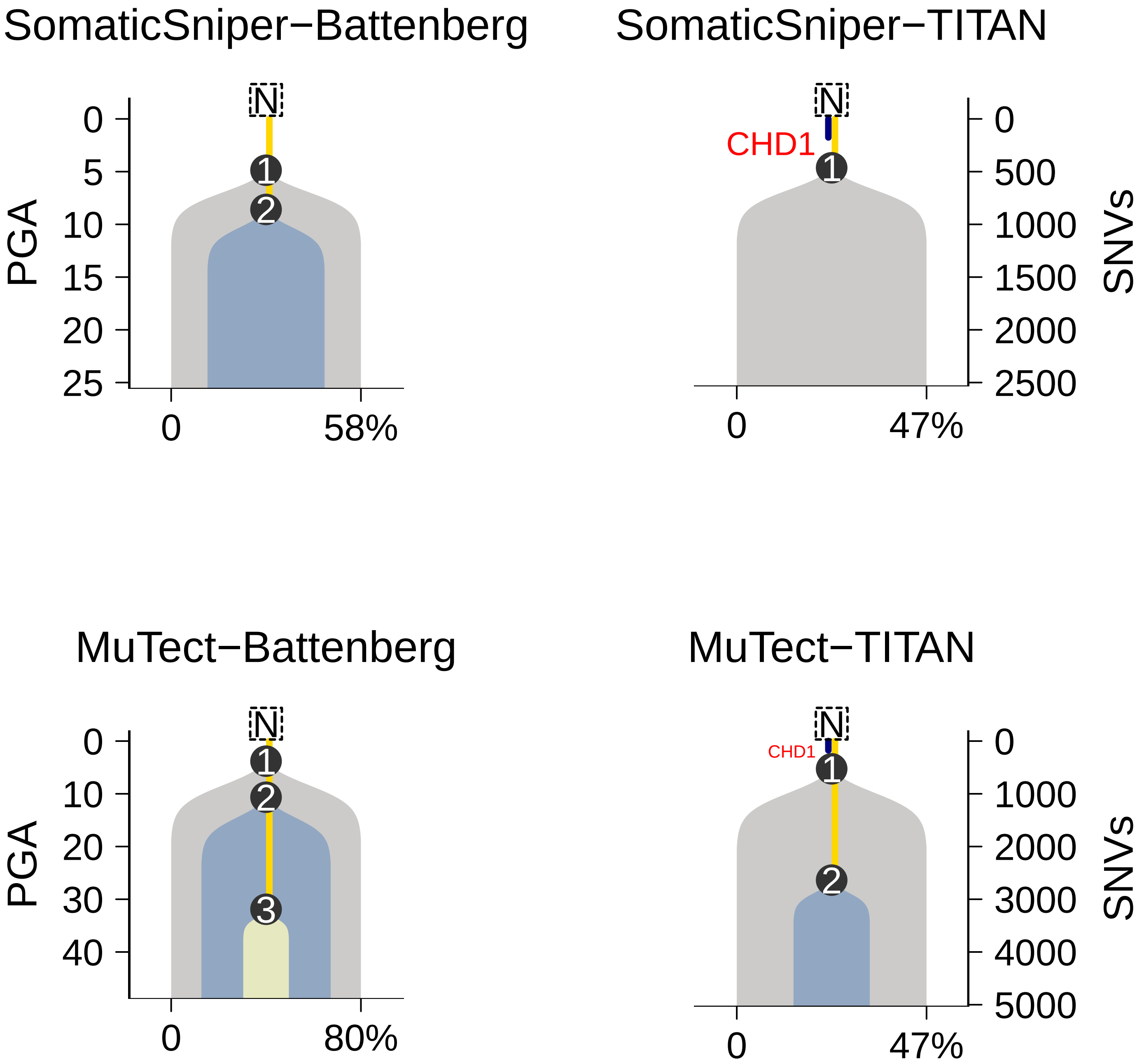

SomaticSniper−Battenberg
SomaticSniper−TITAN
MuTect−Battenberg
MuTect−TITAN
N
1
2
3
CHD1
PGA
SNVs
0
5
10
15
20
25
30
40
500
1000
1500
2000
2500
3000
4000
5000
58%
47%
80%

CPCG0117

SomaticSniper−TITAN

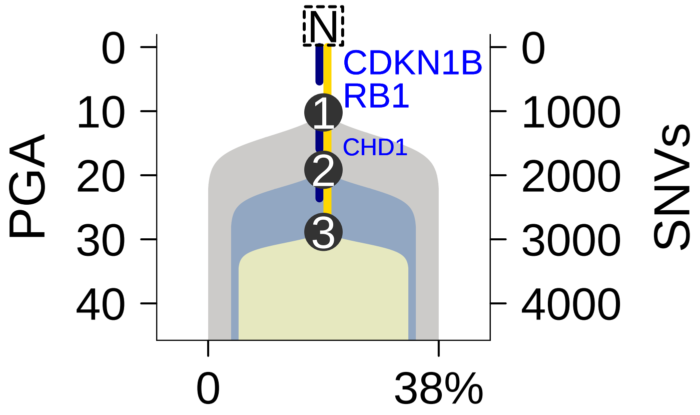

MuTect−TITAN

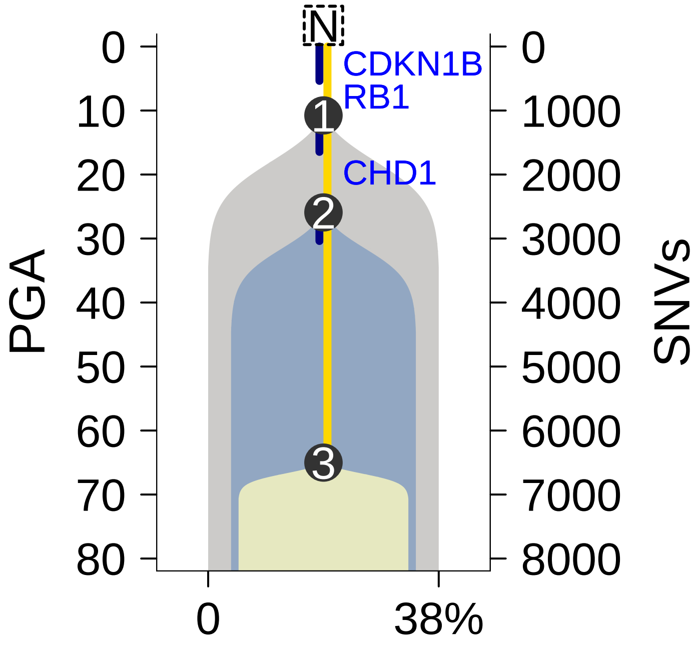

CPCG0120

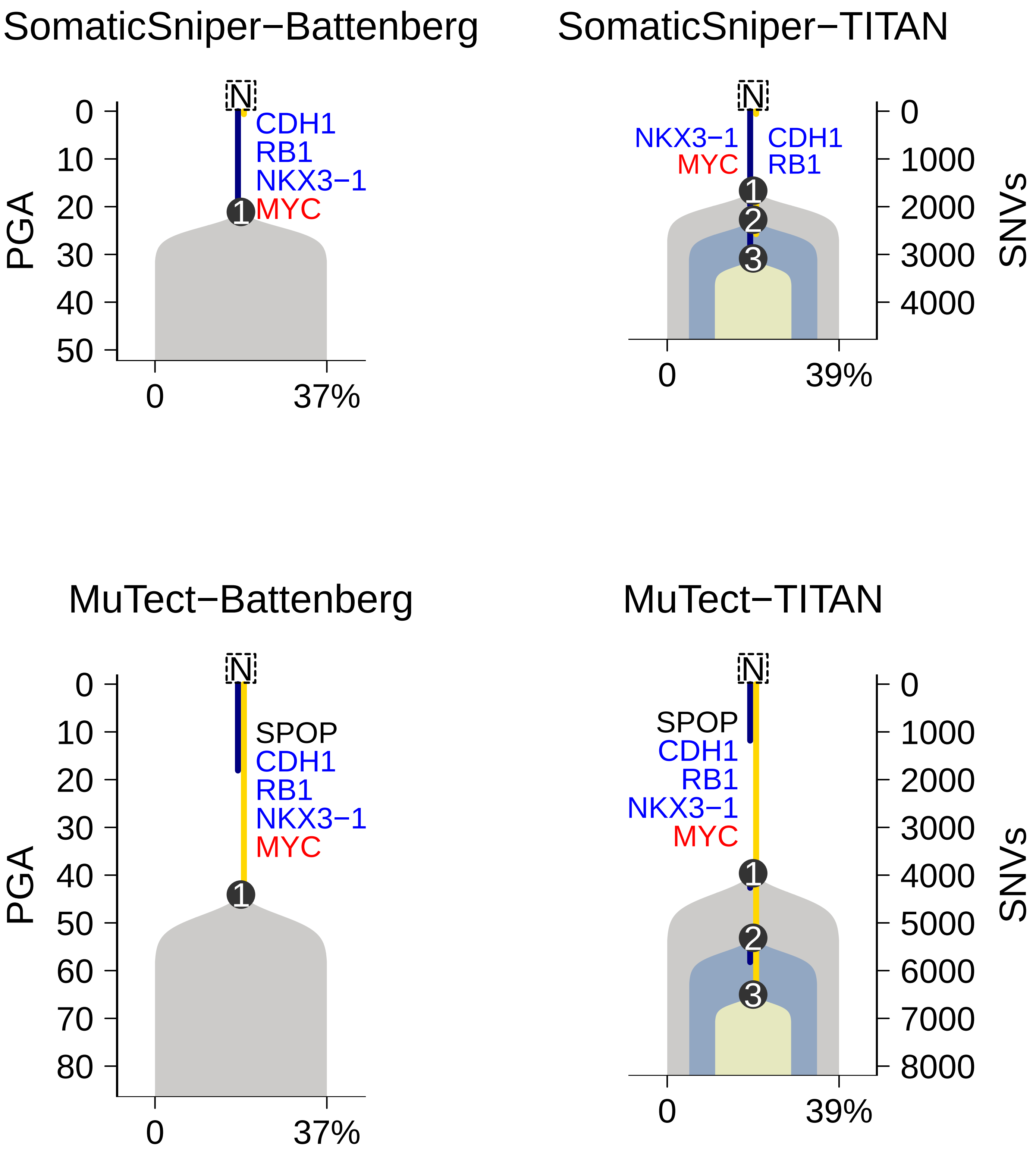

SomaticSniper−Battenberg
N
CDH1
RB1
NKX3−1
MYC
1
PGA
0
10
20
30
40
50
0
37%
SomaticSniper−TITAN
N
NKX3−1
MYC
CDH1
RB1
1
2
3
0
1000
2000
3000
4000
SNVs
0
39%
MuTect−Battenberg
N
SPOP
CDH1
RB1
NKX3−1
MYC
1
PGA
0
10
20
30
40
50
60
70
80
0
37%
MuTect−TITAN
N
SPOP
CDH1
RB1
NKX3−1
MYC
1
2
3
0
1000
2000
3000
4000
5000
6000
7000
8000
SNVs
0
39%

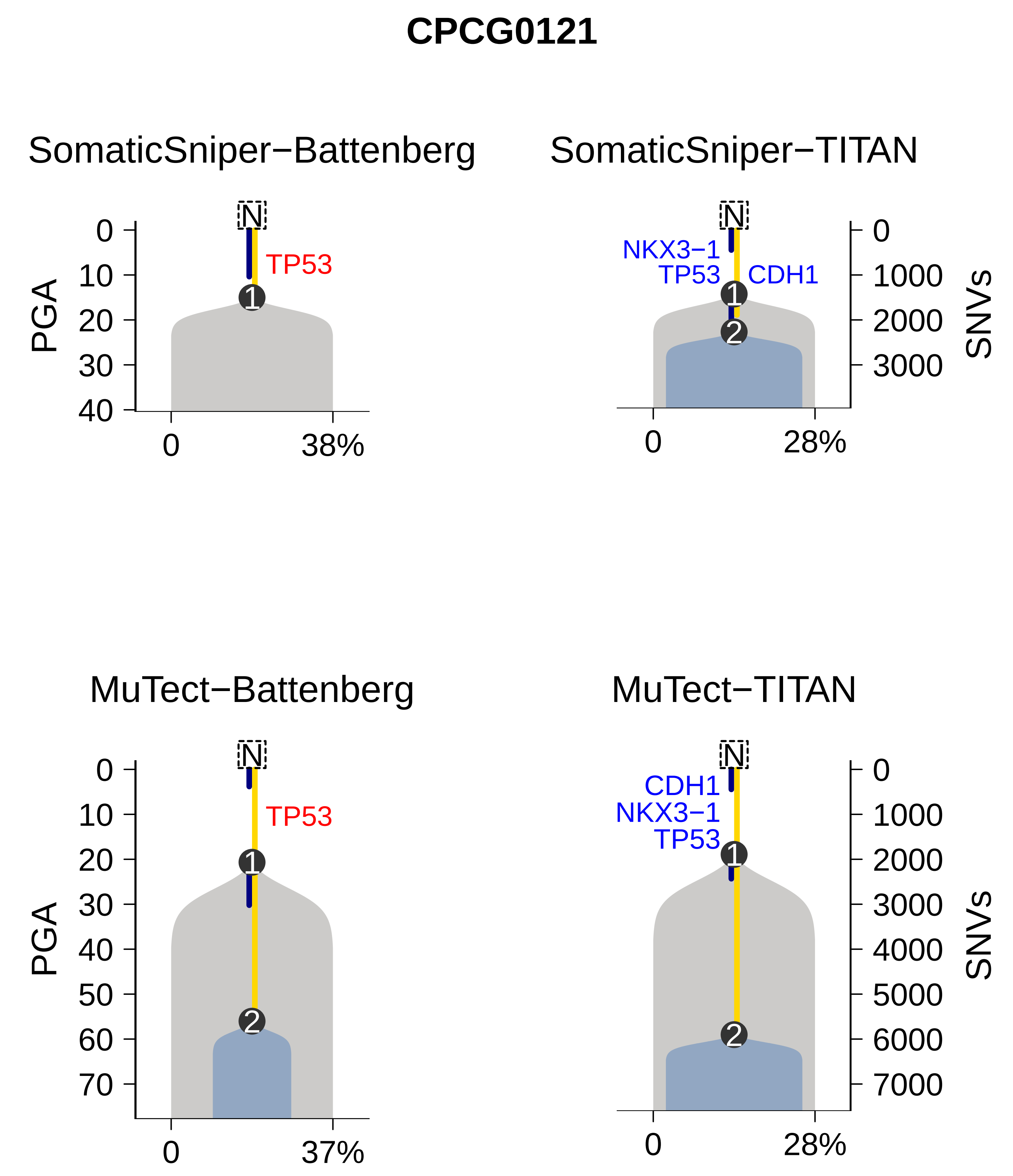
CPCG0121
SomaticSniper−Battenberg
SomaticSniper−TITAN
MuTect−Battenberg
MuTect−TITAN
N
TP53
NKX3−1
CDH1
1
2
PGA
SNVs
0
10
20
30
40
50
60
70
1000
2000
3000
4000
5000
6000
7000
38%
28%
37%

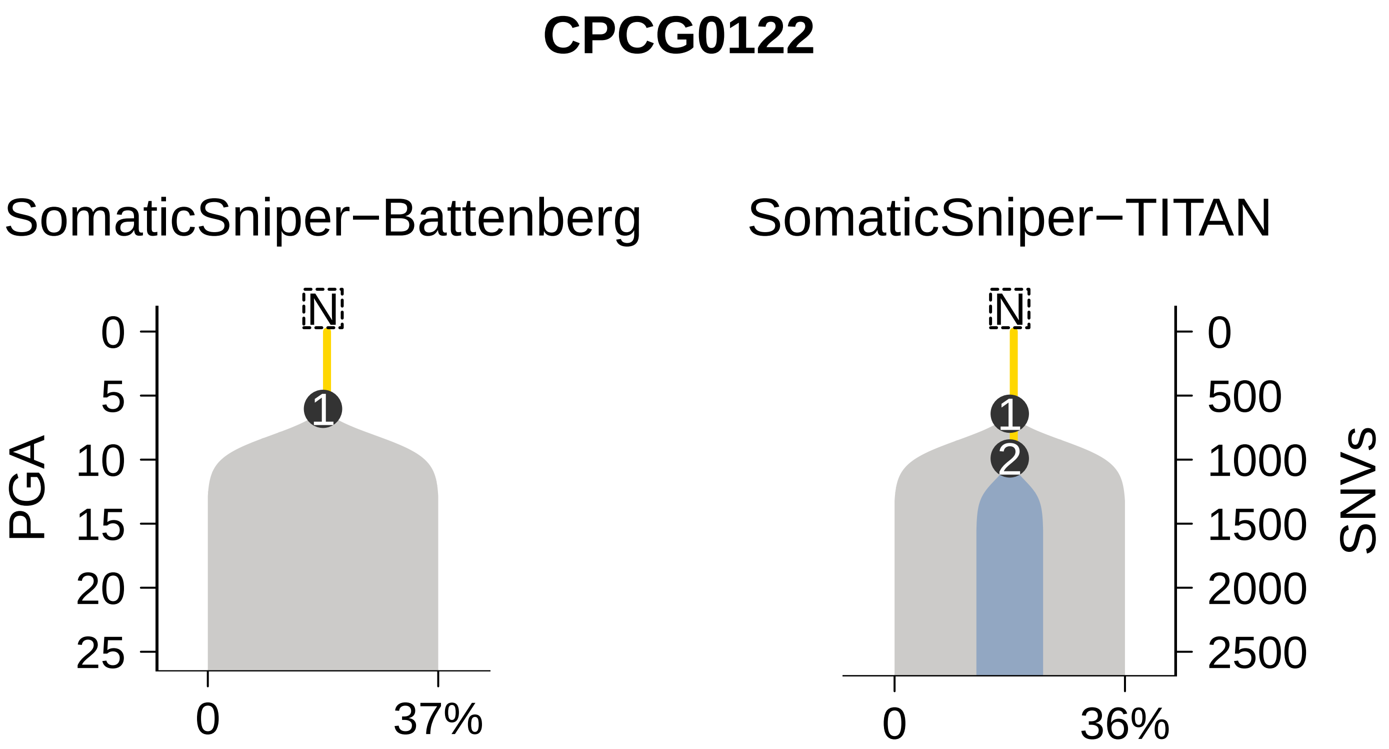

CPCG0122
SomaticSniper−Battenberg
SomaticSniper−TITAN
N
1
2
PGA
0
5
10
15
20
25
SNVs
500
1000
1500
2000
2500
37%
36%

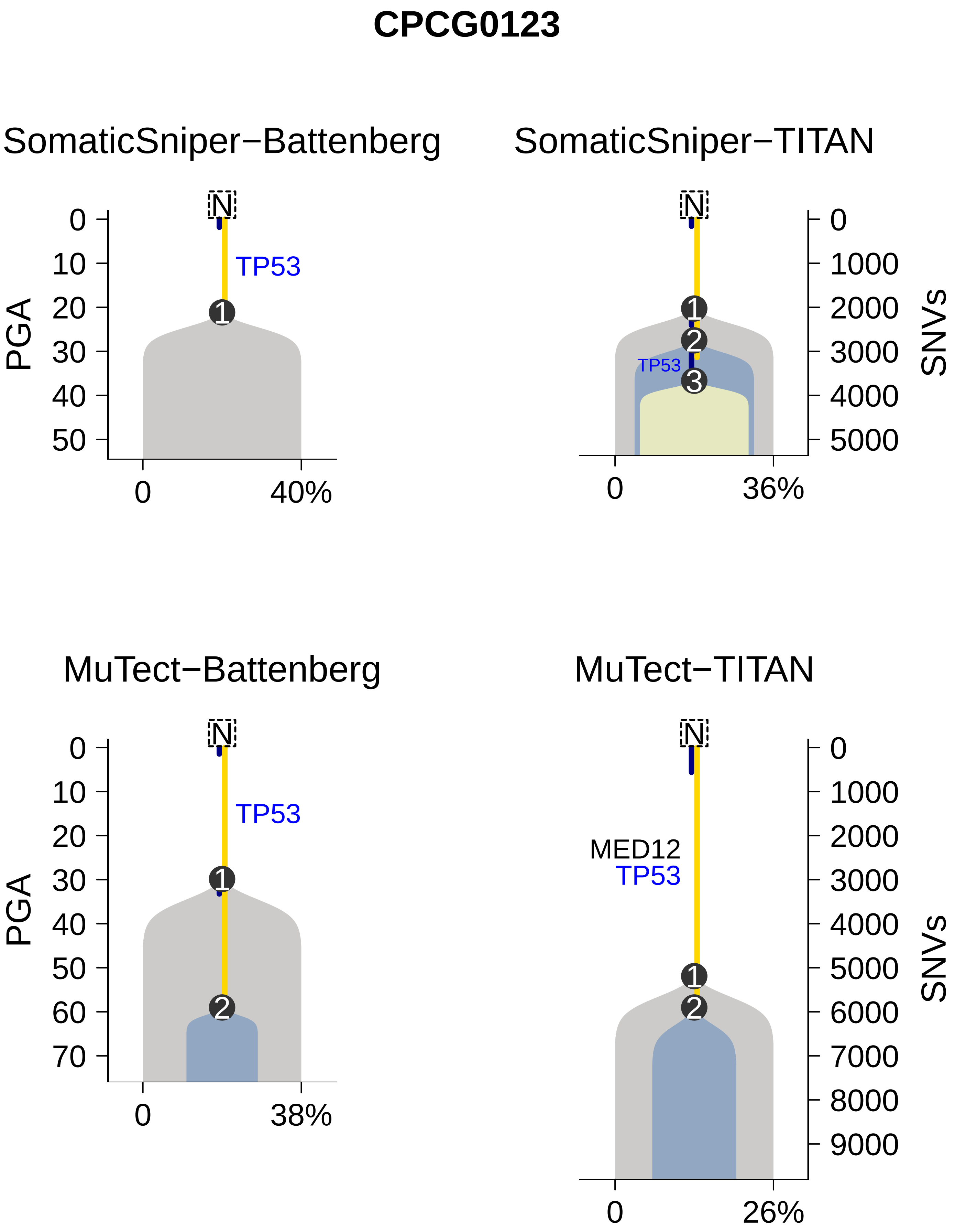
CPCG0123
SomaticSniper−Battenberg
SomaticSniper−TITAN
MuTect−Battenberg
MuTect−TITAN
N
1
2
3
TP53
MED12
PGA
SNVs
0
10
20
30
40
50
60
70
0
1000
2000
3000
4000
5000
6000
7000
8000
9000
40%
36%
38%
26%

CPCG0124

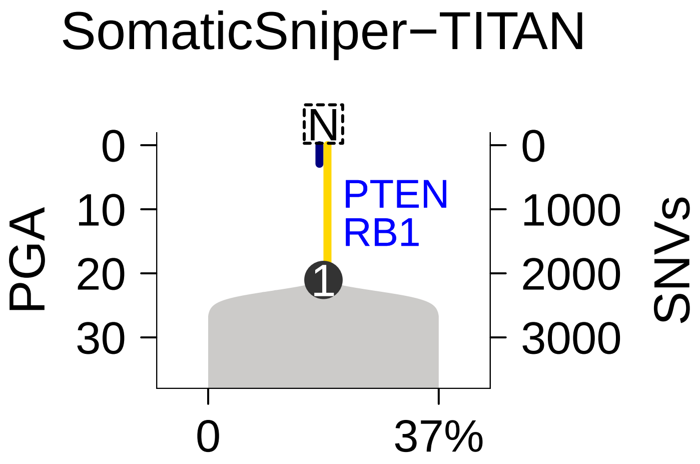
SomaticSniper−TITAN
N
PTEN
RB1
1
PGA
0
10
20
30
SNVs
0
1000
2000
3000
0
37%

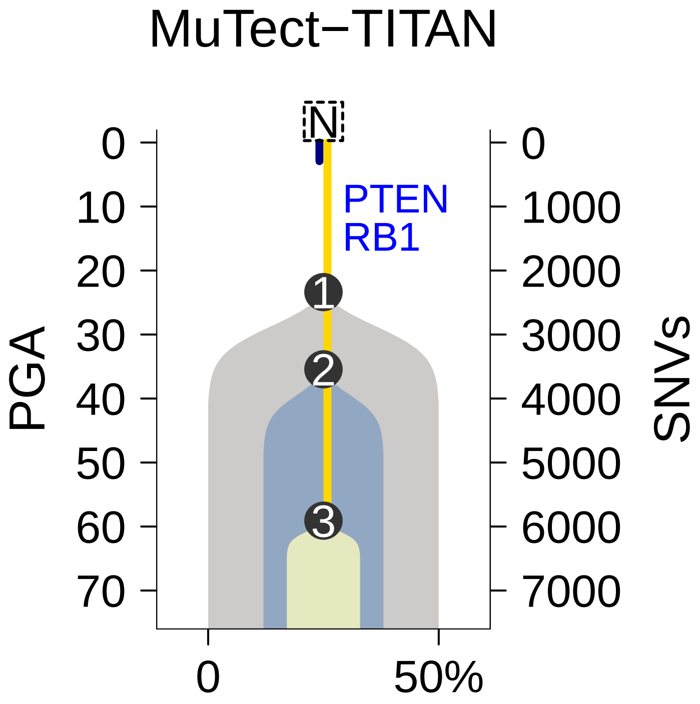
MuTect−TITAN
N
PTEN
RB1
1
2
3
PGA
0
10
20
30
40
50
60
70
SNVs
0
1000
2000
3000
4000
5000
6000
7000
0
50%

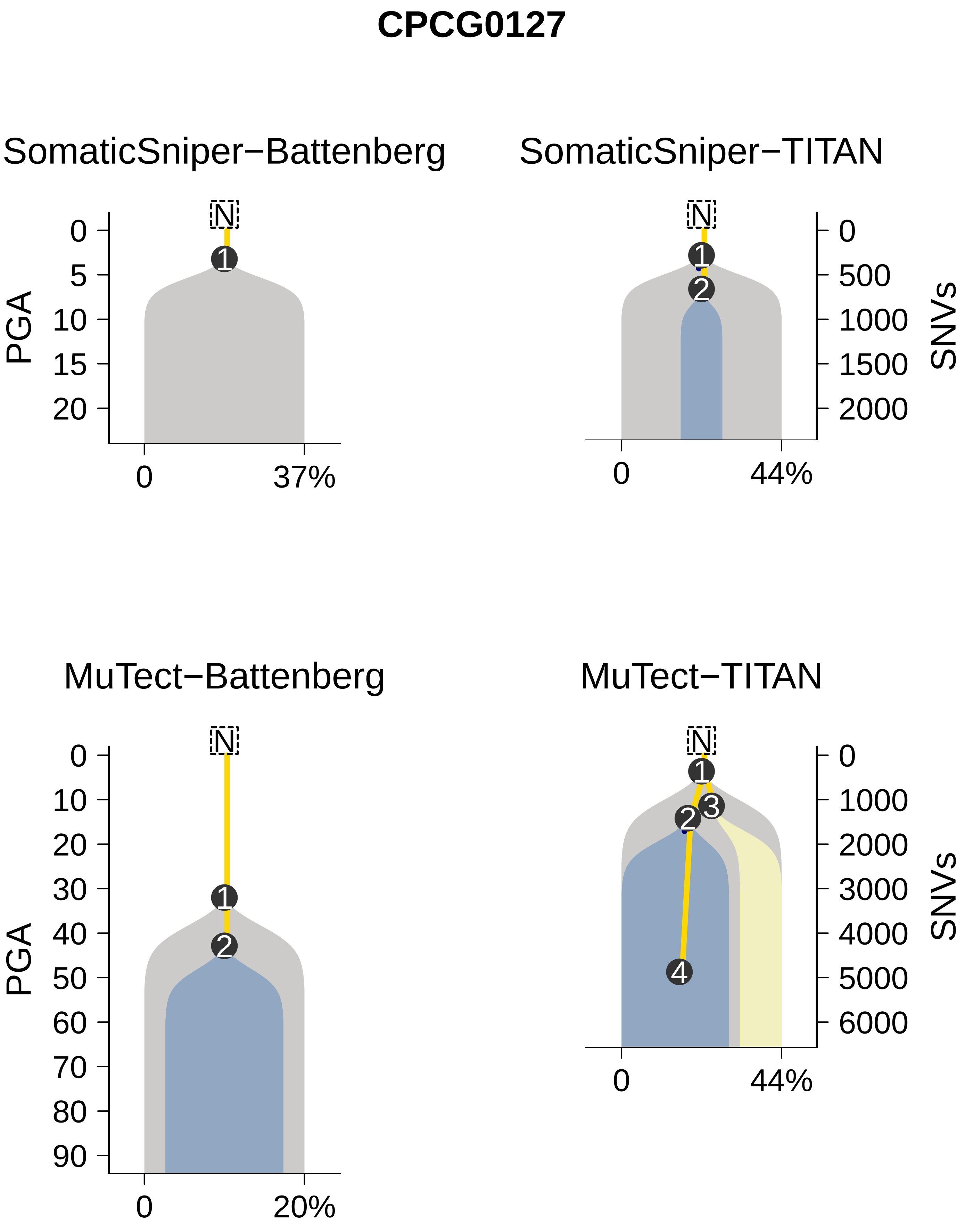

CPCG0127
SomaticSniper−Battenberg
SomaticSniper−TITAN
MuTect−Battenberg
MuTect−TITAN
PGA
SNVs
N
0
5
10
15
20
37%
44%
500
1000
1500
2000
20%
30
40
50
60
70
80
90
3000
4000
5000
6000

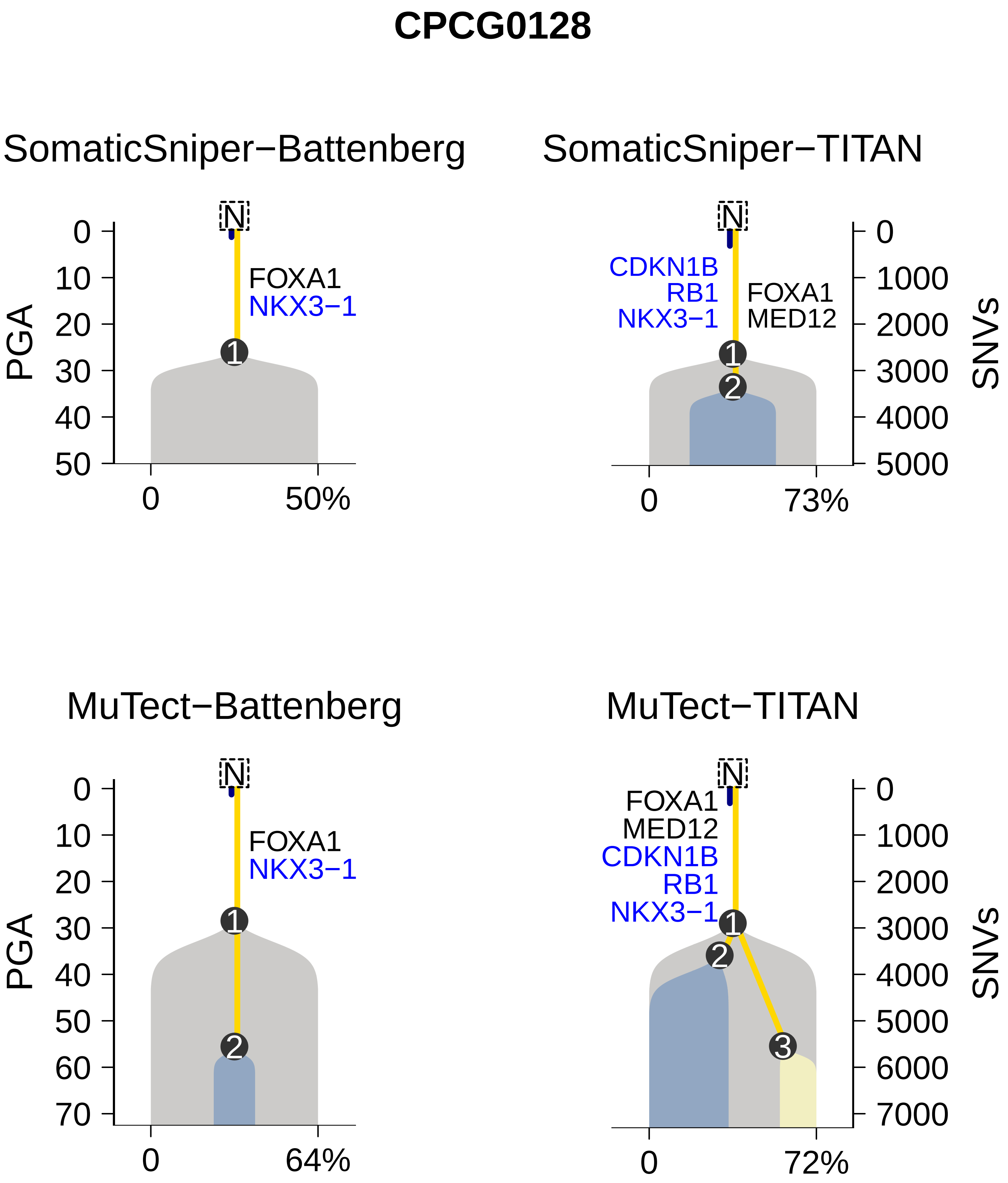
CPCG0128
SomaticSniper−Battenberg
N
FOXA1
NKX3−1
1
PGA
0
10
20
30
40
50
0
50%
SomaticSniper−TITAN
N
CDKN1B
RB1
NKX3−1
FOXA1
MED12
1
2
SNVs
0
1000
2000
3000
4000
5000
0
73%
MuTect−Battenberg
N
FOXA1
NKX3−1
1
2
PGA
0
10
20
30
40
50
60
70
0
64%
MuTect−TITAN
N
FOXA1
MED12
CDKN1B
RB1
NKX3−1
1
2
3
SNVs
0
1000
2000
3000
4000
5000
6000
7000
0
72%

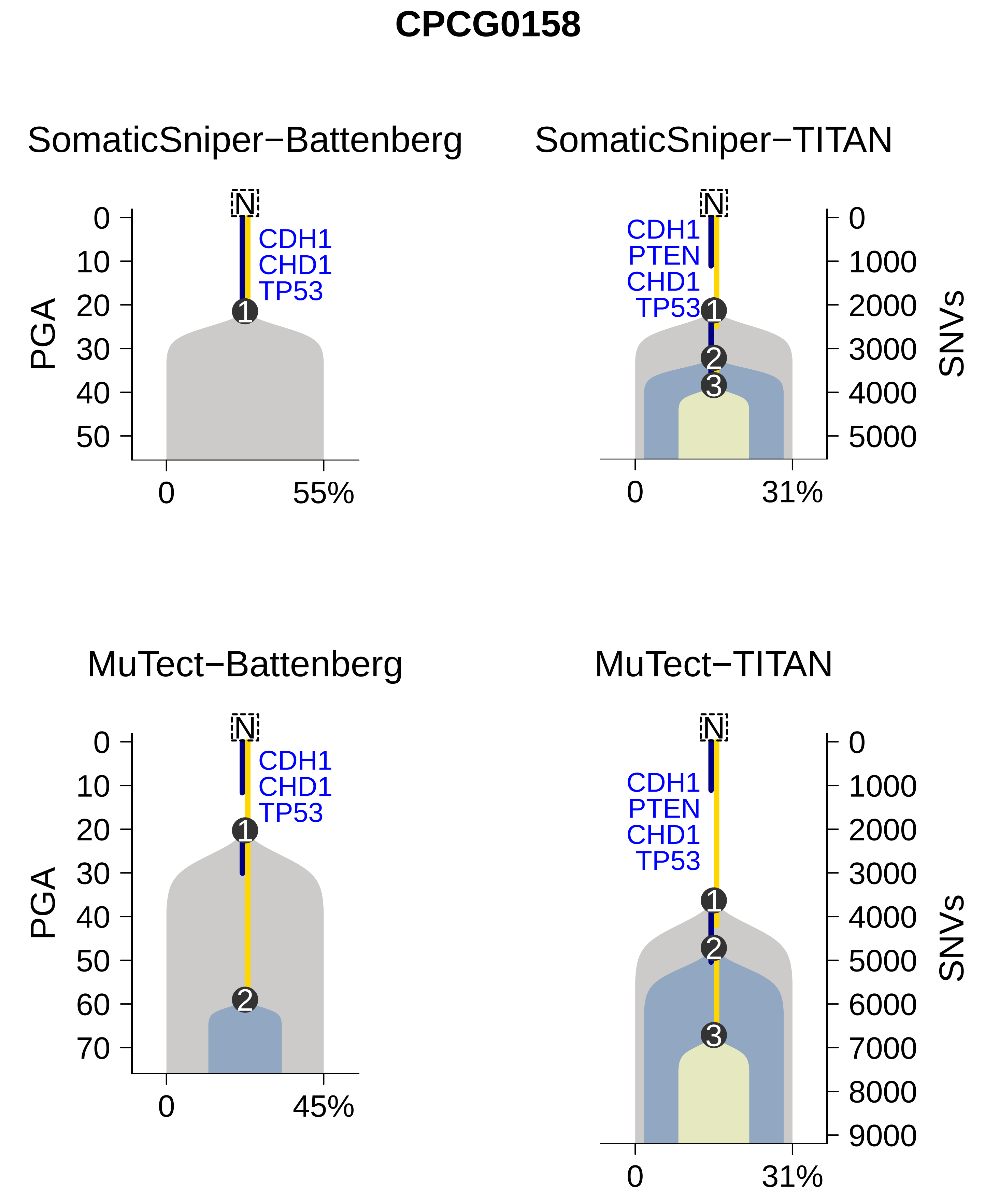
CPCG0158
SomaticSniper−Battenberg
SomaticSniper−TITAN
MuTect−Battenberg
MuTect−TITAN
PGA
SNVs
N
CDH1
CHD1
TP53
CDH1
PTEN
CHD1
TP53
0
55%
0
31%
0
45%
0
31%

CPCG0166

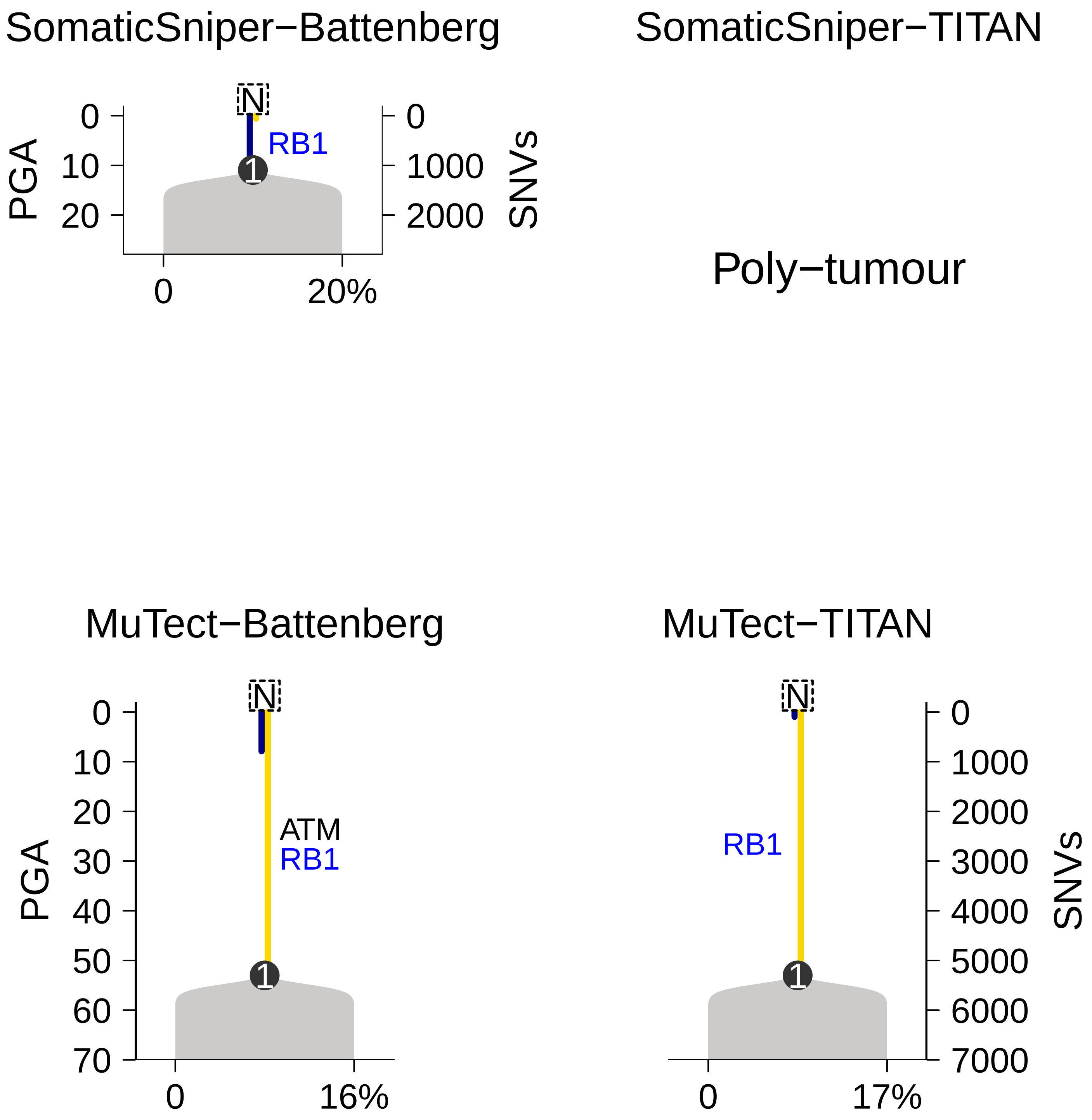
SomaticSniper−Battenberg
N
RB1
1
PGA
0
10
20
SNVs
0
1000
2000
0
20%
SomaticSniper−TITAN
Poly−tumour
MuTect−Battenberg
N
ATM
RB1
1
PGA
0
10
20
30
40
50
60
70
0
16%
MuTect−TITAN
N
RB1
1
SNVs
0
1000
2000
3000
4000
5000
6000
7000
0
17%

CPCG0182

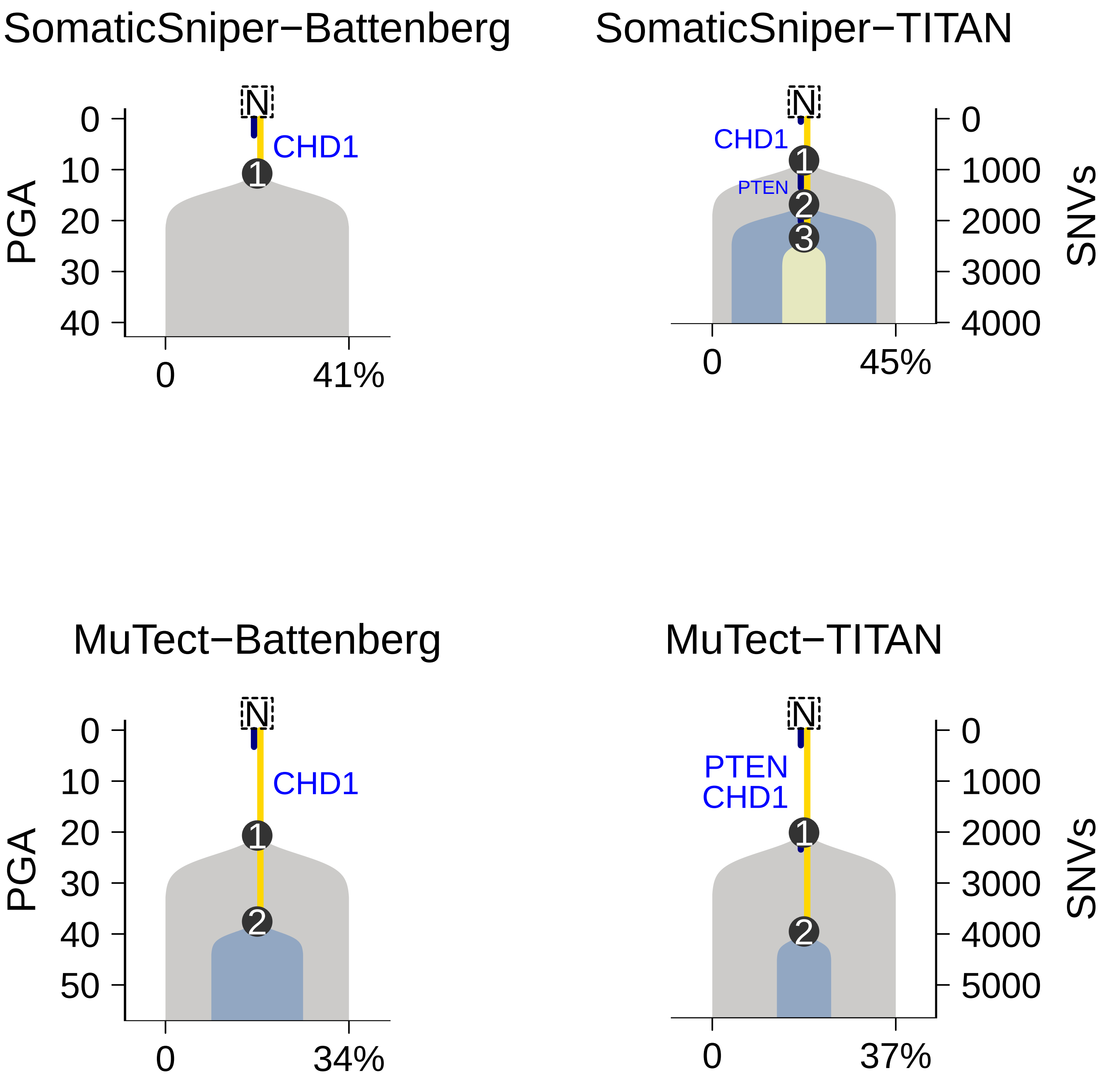
SomaticSniper−Battenberg
SomaticSniper−TITAN
MuTect−Battenberg
MuTect−TITAN
N
CHD1
PTEN
PGA
SNVs
0
10
20
30
40
50
41%
45%
34%
37%
1000
2000
3000
4000
5000

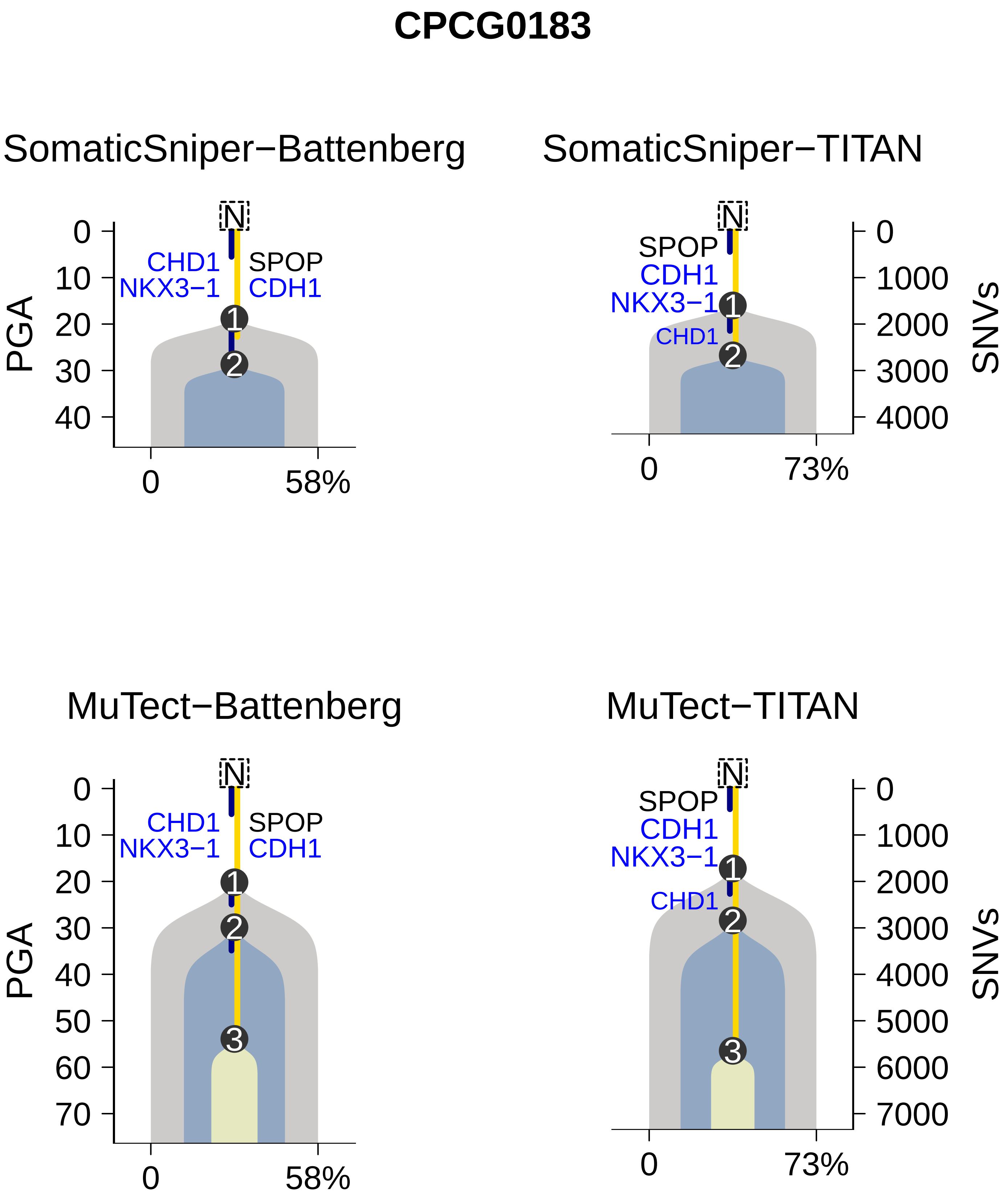
CPCG0183
SomaticSniper−Battenberg
SomaticSniper−TITAN
MuTect−Battenberg
MuTect−TITAN
N
CHD1
NKX3−1
SPOP
CDH1
PGA
SNVs
0
58%
73%

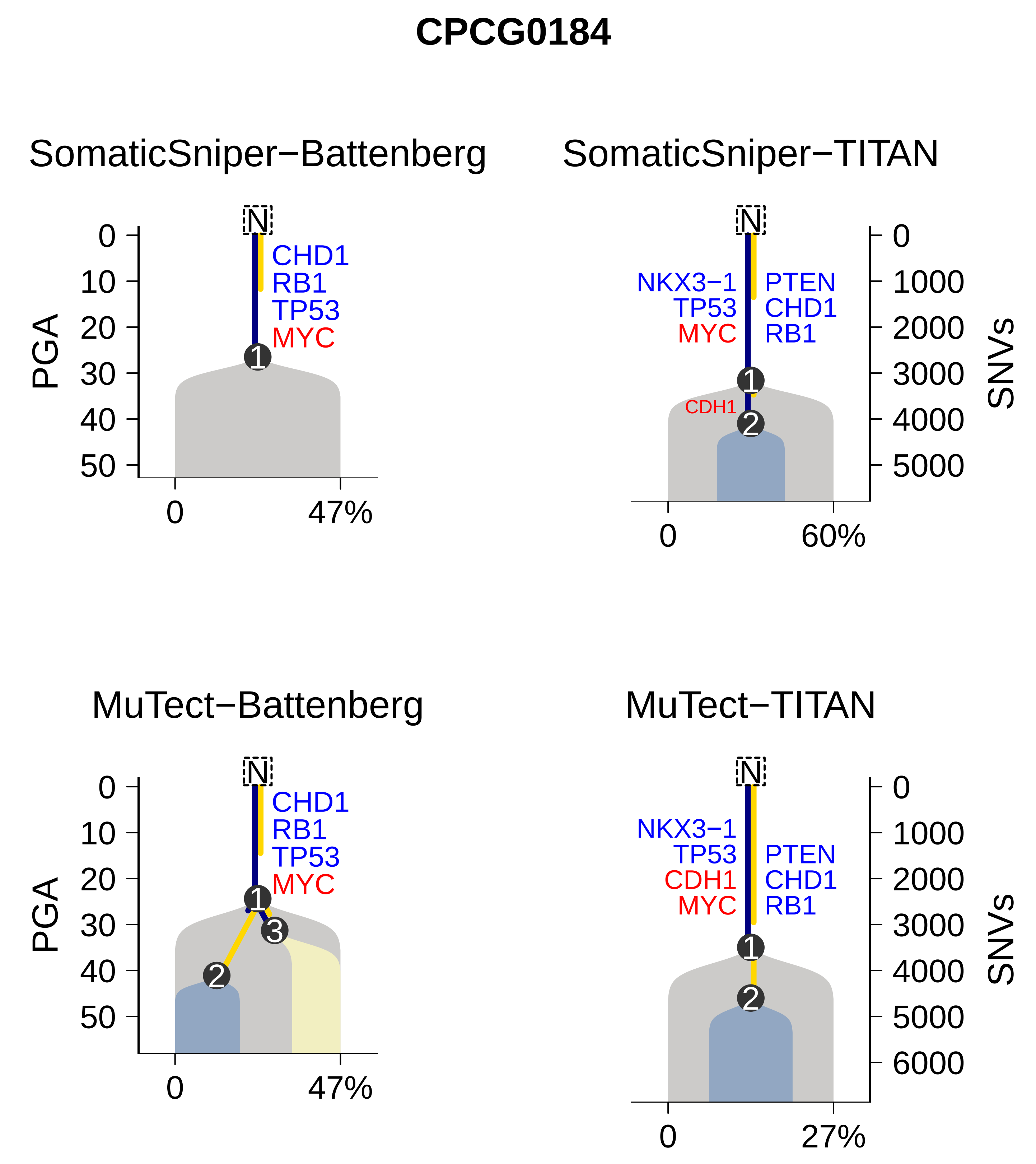
CPCG0184
SomaticSniper−Battenberg
N
CHD1
RB1
TP53
MYC
1
PGA
0
10
20
30
40
50
0
47%
SomaticSniper−TITAN
N
NKX3−1
TP53
MYC
PTEN
CHD1
RB1
CDH1
1
2
0
1000
2000
3000
4000
5000
SNVs
0
60%
MuTect−Battenberg
N
CHD1
RB1
TP53
MYC
1
2
3
PGA
0
10
20
30
40
50
0
47%
MuTect−TITAN
N
NKX3−1
TP53
CDH1
MYC
PTEN
CHD1
RB1
1
2
0
1000
2000
3000
4000
5000
6000
SNVs
0
27%

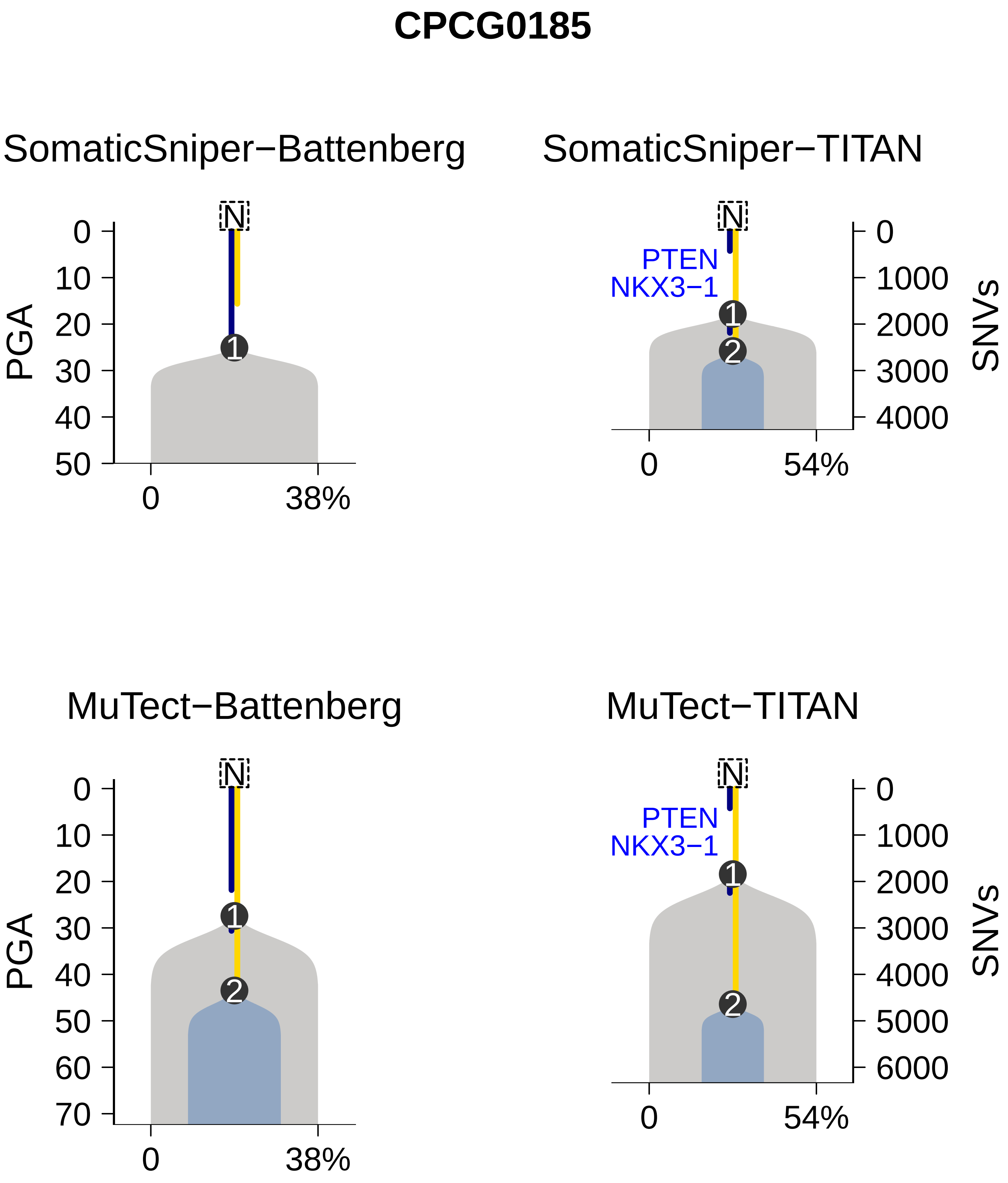
CPCG0185
SomaticSniper−Battenberg
SomaticSniper−TITAN
MuTect−Battenberg
MuTect−TITAN
N
1
2
PTEN
NKX3−1
PGA
SNVs
0
10
20
30
40
50
60
70
1000
2000
3000
4000
5000
6000
38%
54%

CPCG0188

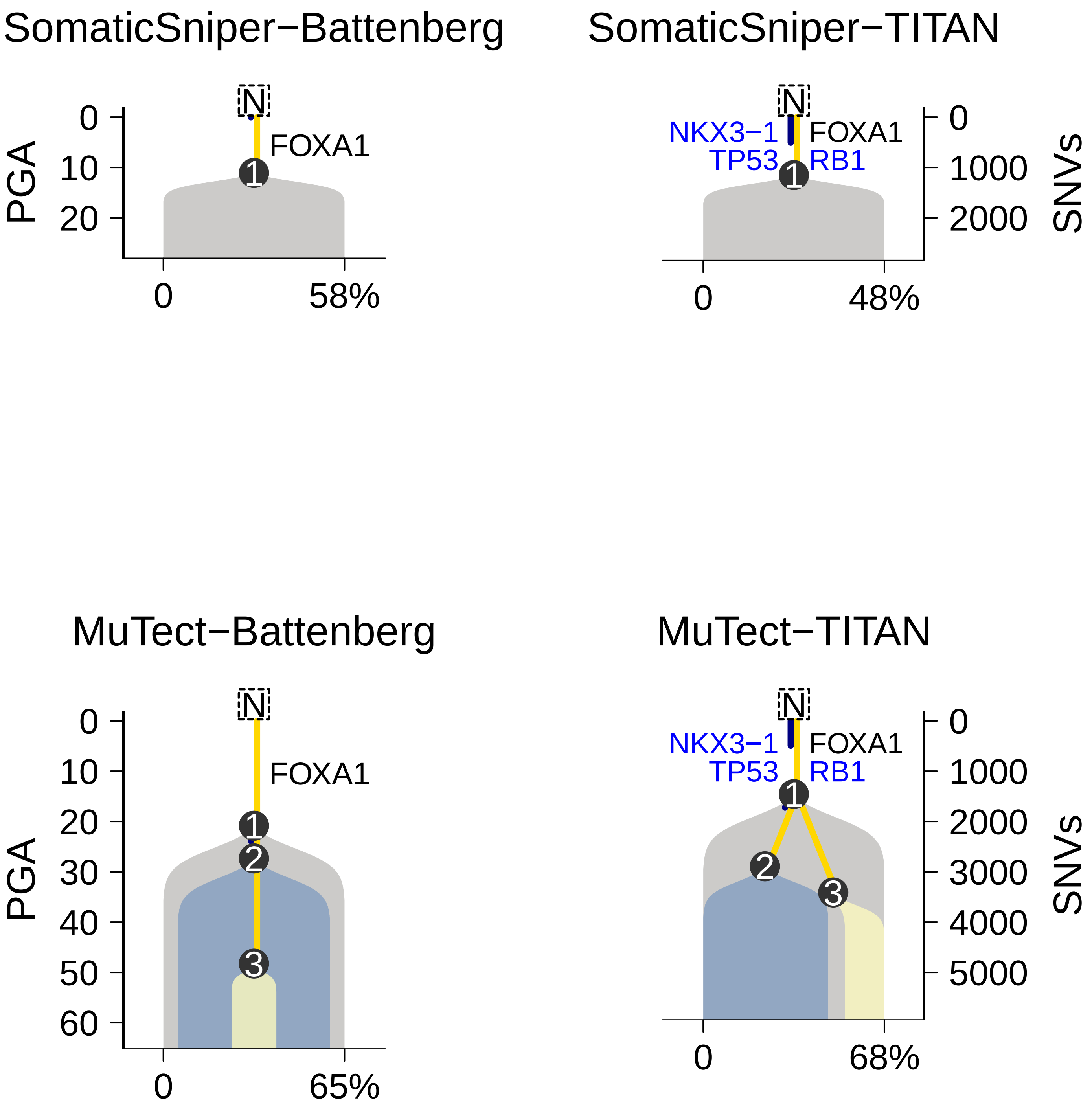
SomaticSniper−Battenberg
SomaticSniper−TITAN
N
FOXA1
PGA
0
10
20
0
58%
NKX3−1
TP53
RB1
1
0
1000
2000
SNVs
48%
MuTect−Battenberg
MuTect−TITAN
30
40
50
60
65%
2
3
3000
4000
5000
68%

CPCG0189

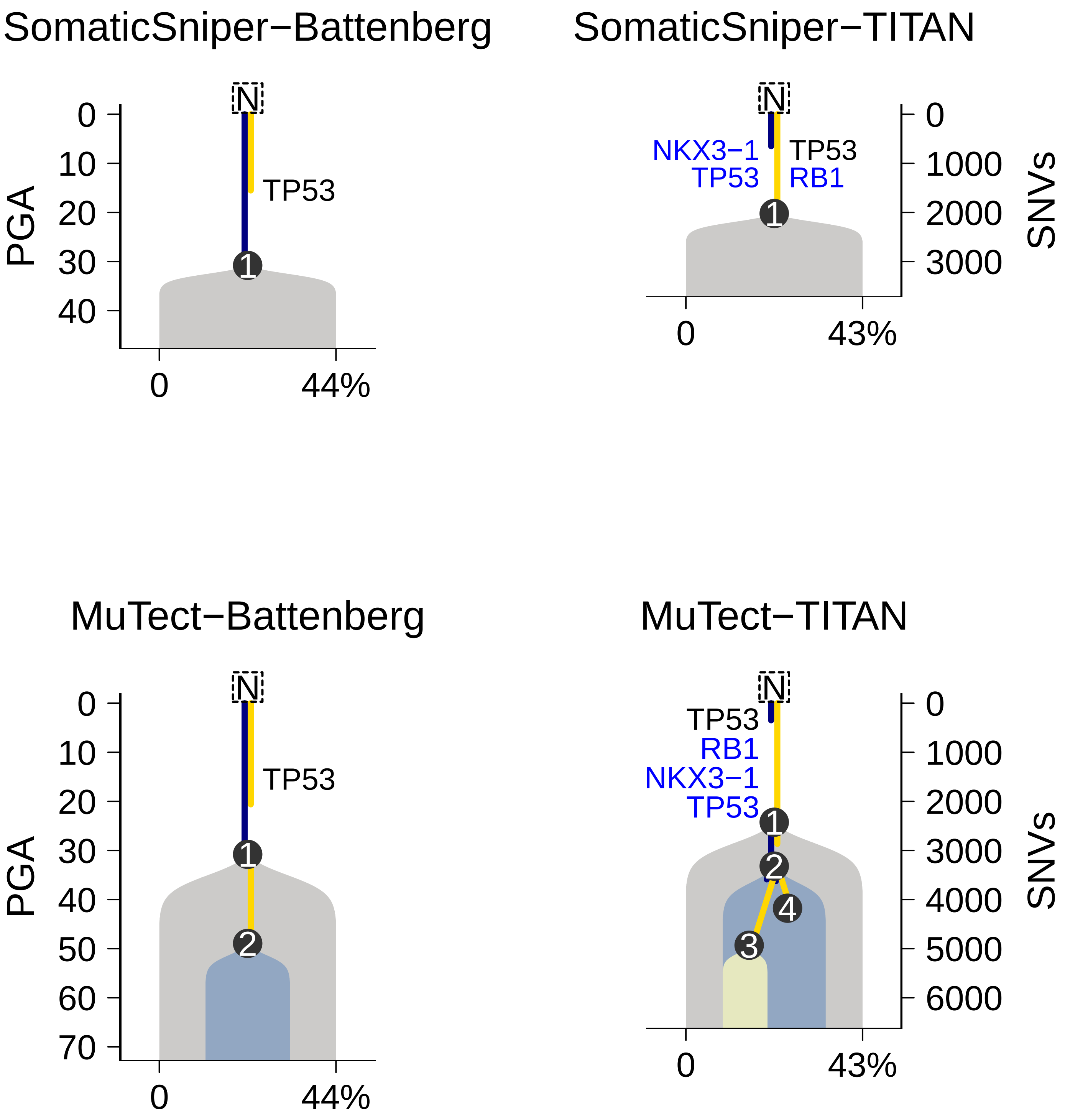
SomaticSniper−Battenberg
SomaticSniper−TITAN
MuTect−Battenberg
MuTect−TITAN
N
1
2
3
4
TP53
NKX3−1
RB1
PGA
SNVs
0
10
20
30
40
50
60
70
1000
2000
3000
4000
5000
6000
44%
43%

CPCG0190

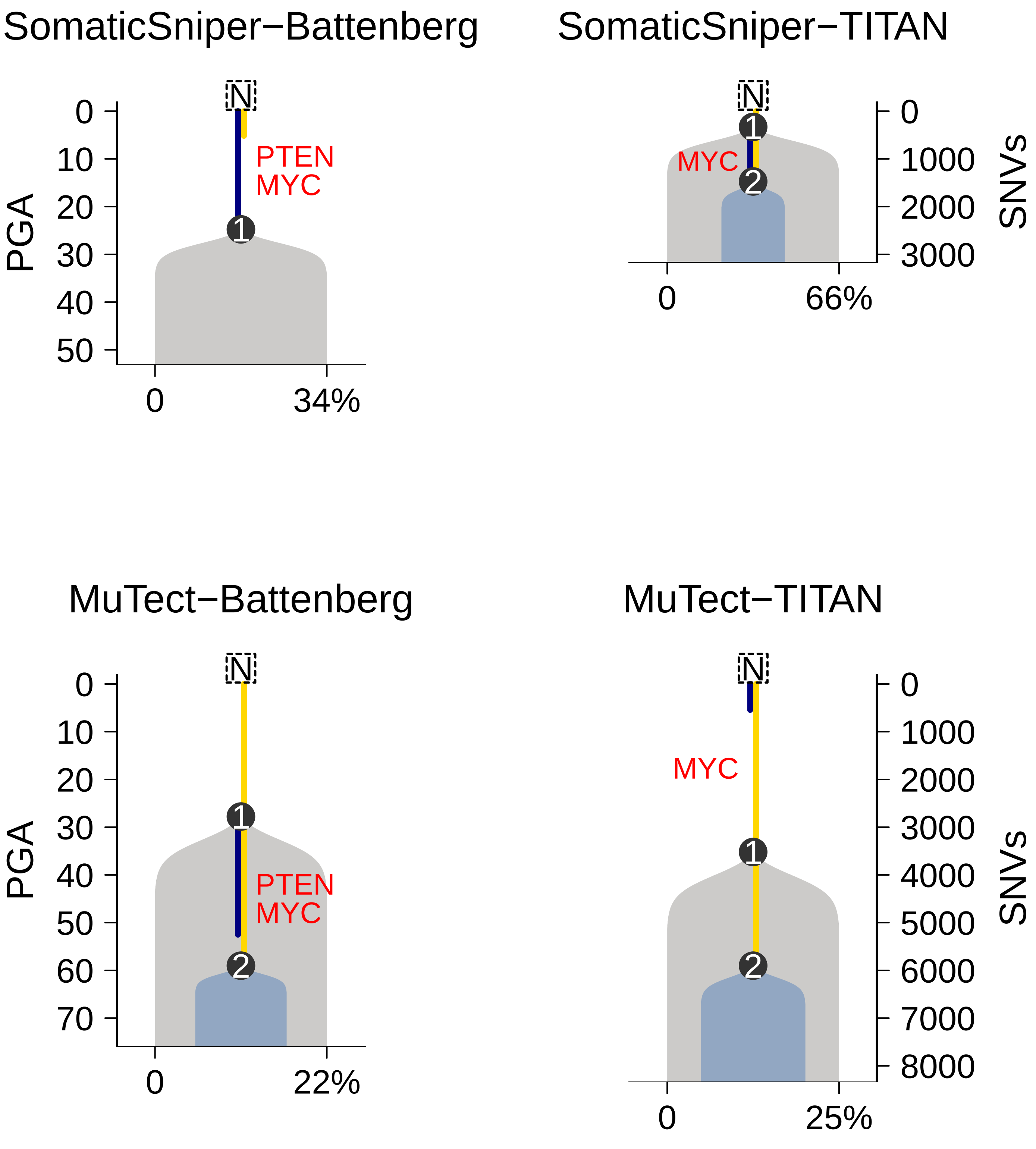

SomaticSniper−Battenberg
N
PTEN
MYC
1
PGA
0
10
20
30
40
50
0
34%
SomaticSniper−TITAN
N
1
MYC
2
0
1000
2000
3000
SNVs
0
66%
MuTect−Battenberg
N
1
PTEN
MYC
2
PGA
0
10
20
30
40
50
60
70
0
22%
MuTect−TITAN
N
MYC
1
2
0
1000
2000
3000
4000
5000
6000
7000
8000
SNVs
0
25%

CPCG0191

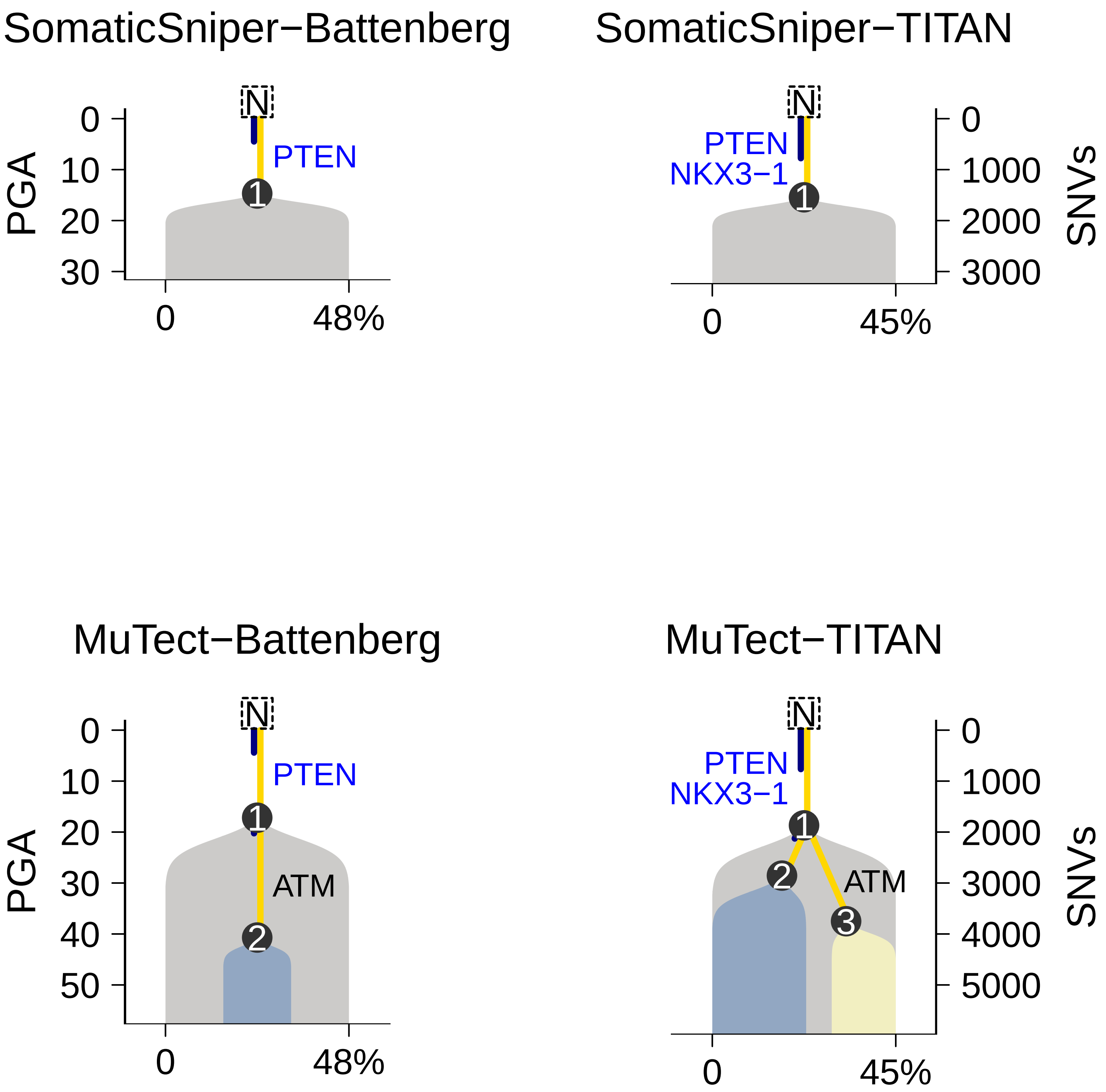
SomaticSniper−Battenberg
SomaticSniper−TITAN
MuTect−Battenberg
MuTect−TITAN
N
1
2
3
PTEN
NKX3−1
ATM
PGA
SNVs
0
10
20
30
40
50
48%
45%
1000
2000
3000
4000
5000

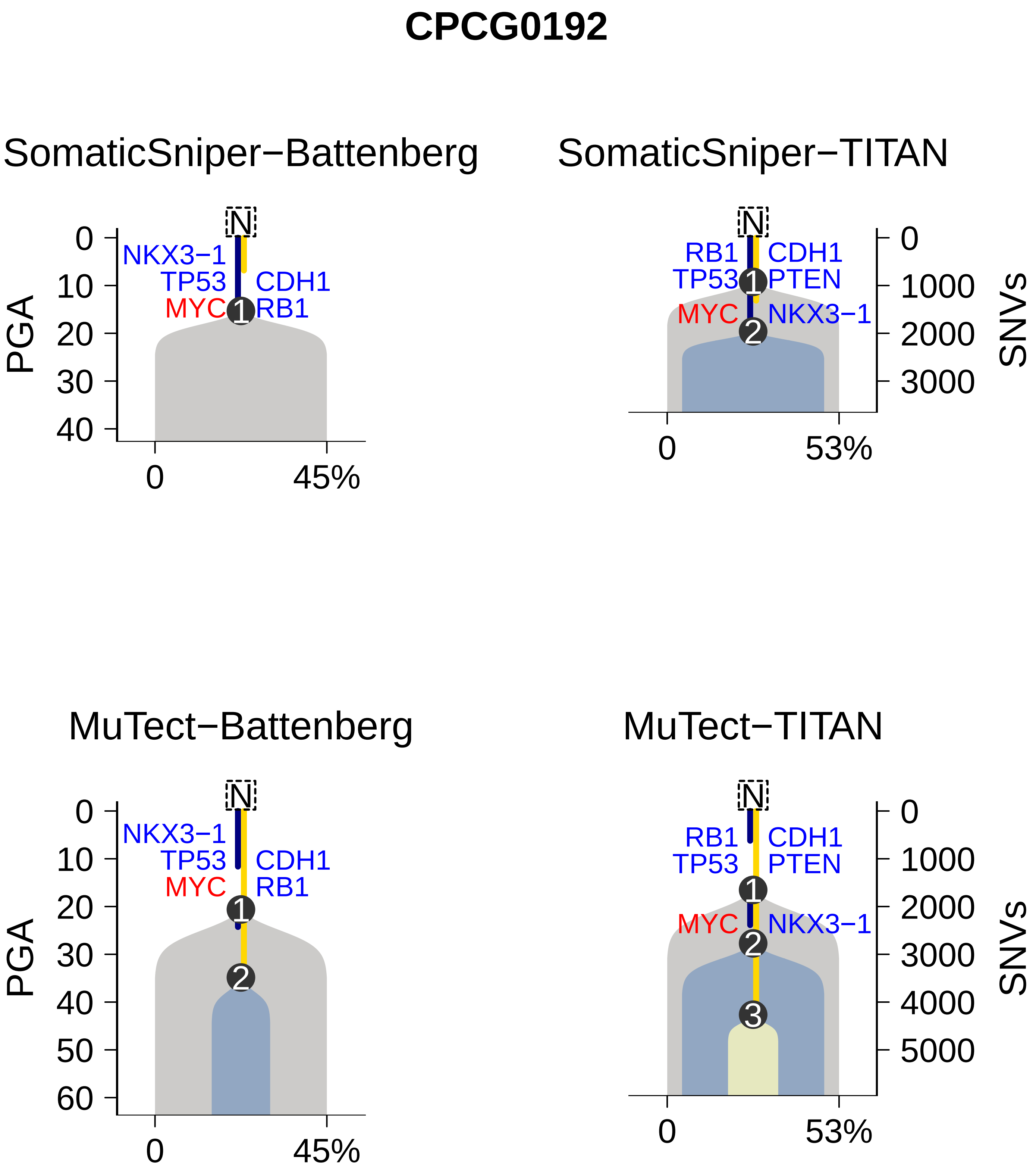
CPCG0192
SomaticSniper−Battenberg
N
NKX3−1
TP53
MYC
CDH1
RB1
1
PGA
0
10
20
30
40
0
45%
SomaticSniper−TITAN
N
RB1
TP53
MYC
CDH1
PTEN
NKX3−1
1
2
0
1000
2000
3000
SNVs
0
53%
MuTect−Battenberg
N
NKX3−1
TP53
MYC
CDH1
RB1
1
2
PGA
0
10
20
30
40
50
60
0
45%
MuTect−TITAN
N
RB1
TP53
MYC
CDH1
PTEN
NKX3−1
1
2
3
0
1000
2000
3000
4000
5000
SNVs
0
53%

CPCG0194

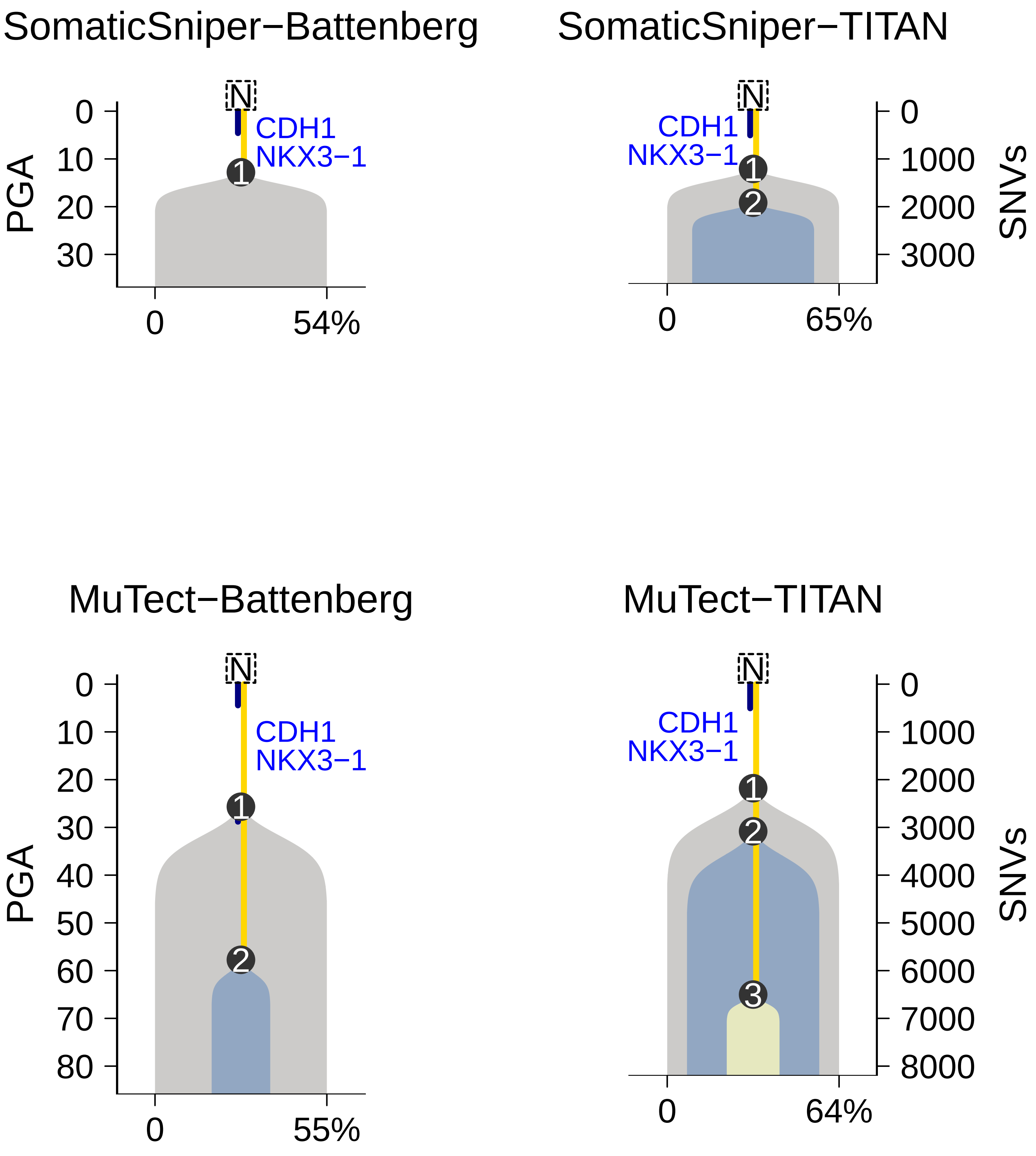

SomaticSniper−Battenberg
SomaticSniper−TITAN
MuTect−Battenberg
MuTect−TITAN
N
CDH1
NKX3−1
PGA
SNVs
0
10
20
30
40
50
60
70
80
1000
2000
3000
4000
5000
6000
7000
8000
54%
65%
55%
64%
1
2
3

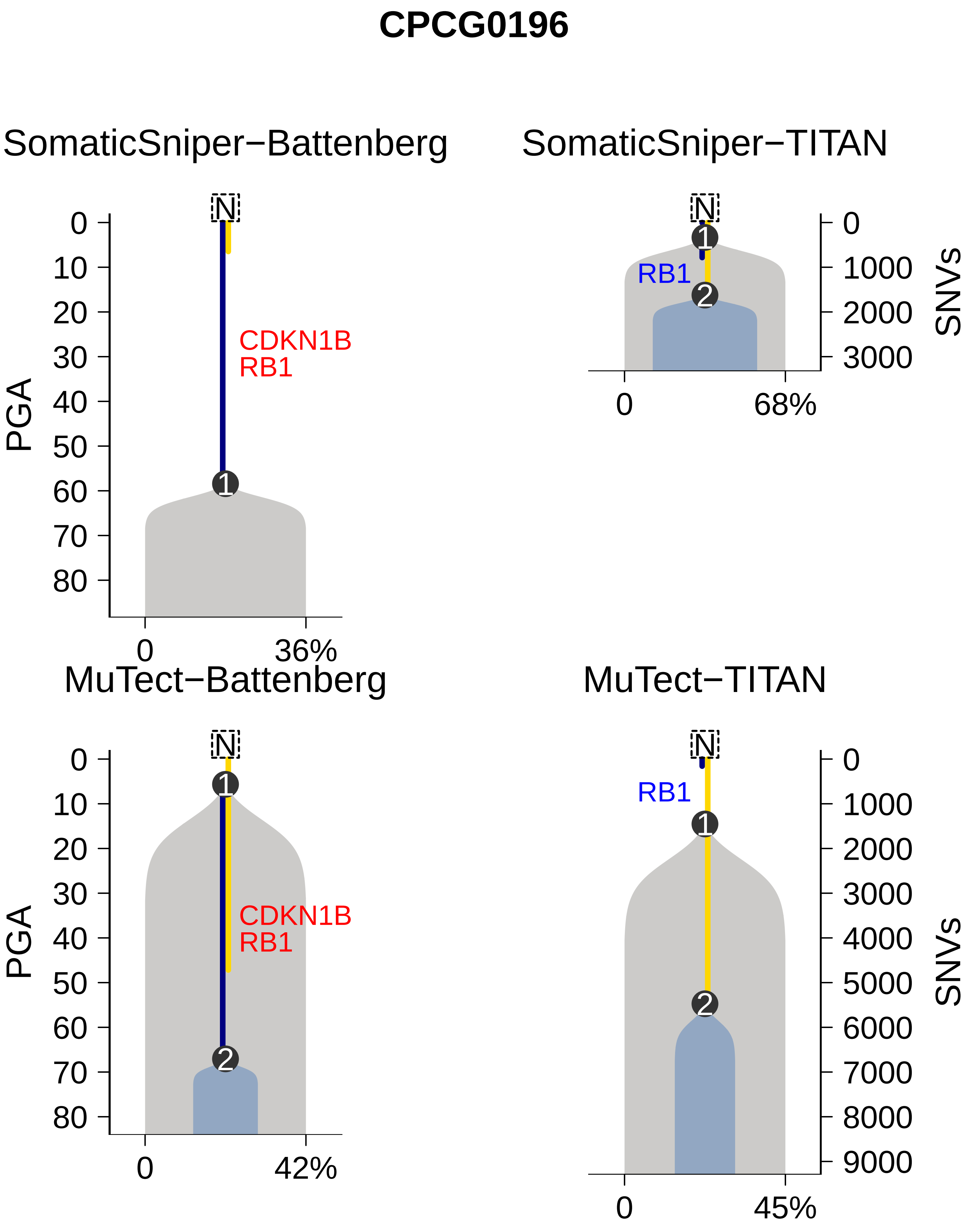
CPCG0196
SomaticSniper−Battenberg
SomaticSniper−TITAN
MuTect−Battenberg
MuTect−TITAN
N
1
2
CDKN1B
RB1
PGA
SNVs
0
10
20
30
40
50
60
70
80
36%
68%
42%
45%
1000
2000
3000
4000
5000
6000
7000
8000
9000

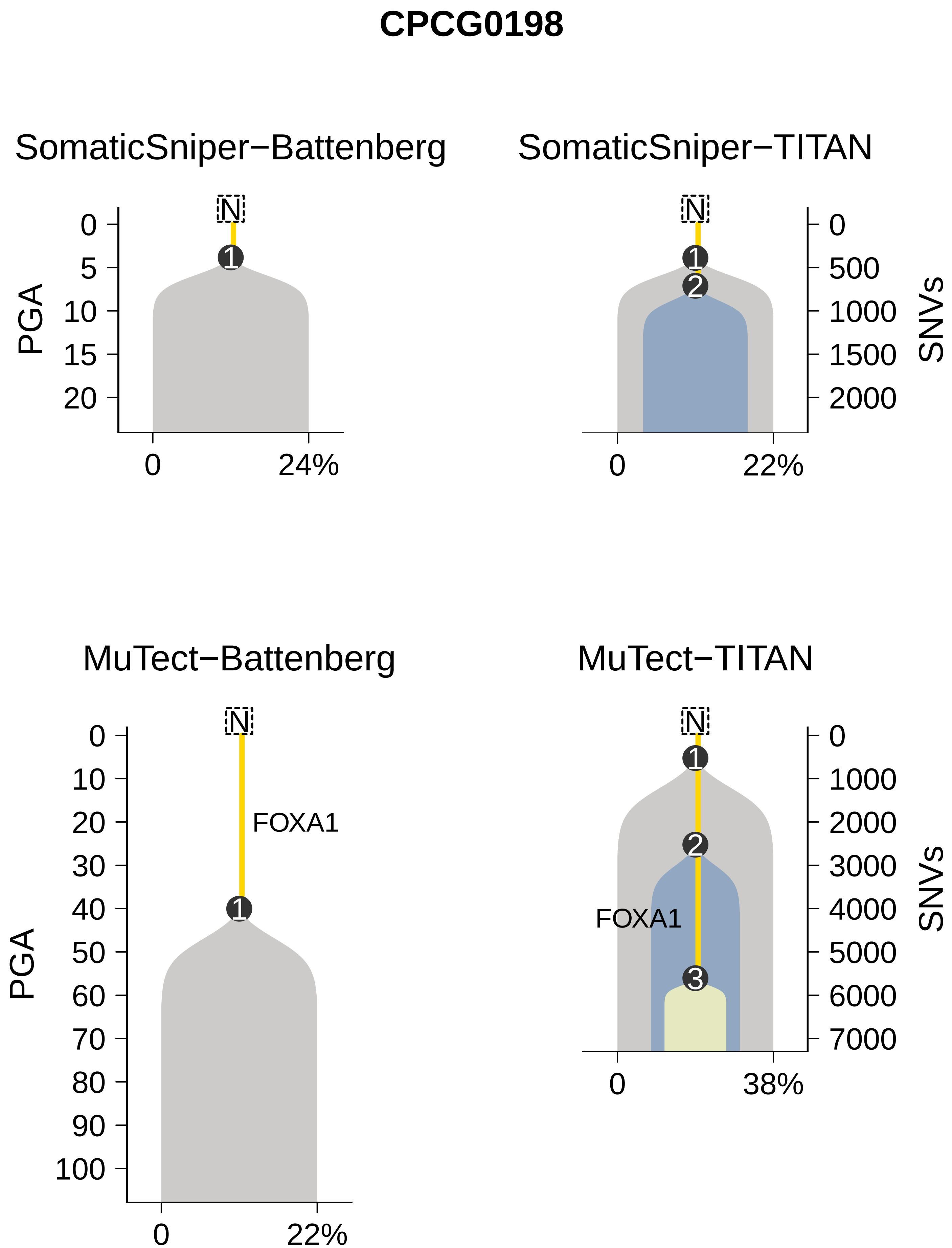
CPCG0198
SomaticSniper−Battenberg
SomaticSniper−TITAN
MuTect−Battenberg
MuTect−TITAN
N
1
2
3
PGA
SNVs
0
5
10
15
20
24%
22%
38%
500
1000
1500
2000
30
40
50
60
70
80
90
100
3000
4000
5000
6000
7000
FOXA1

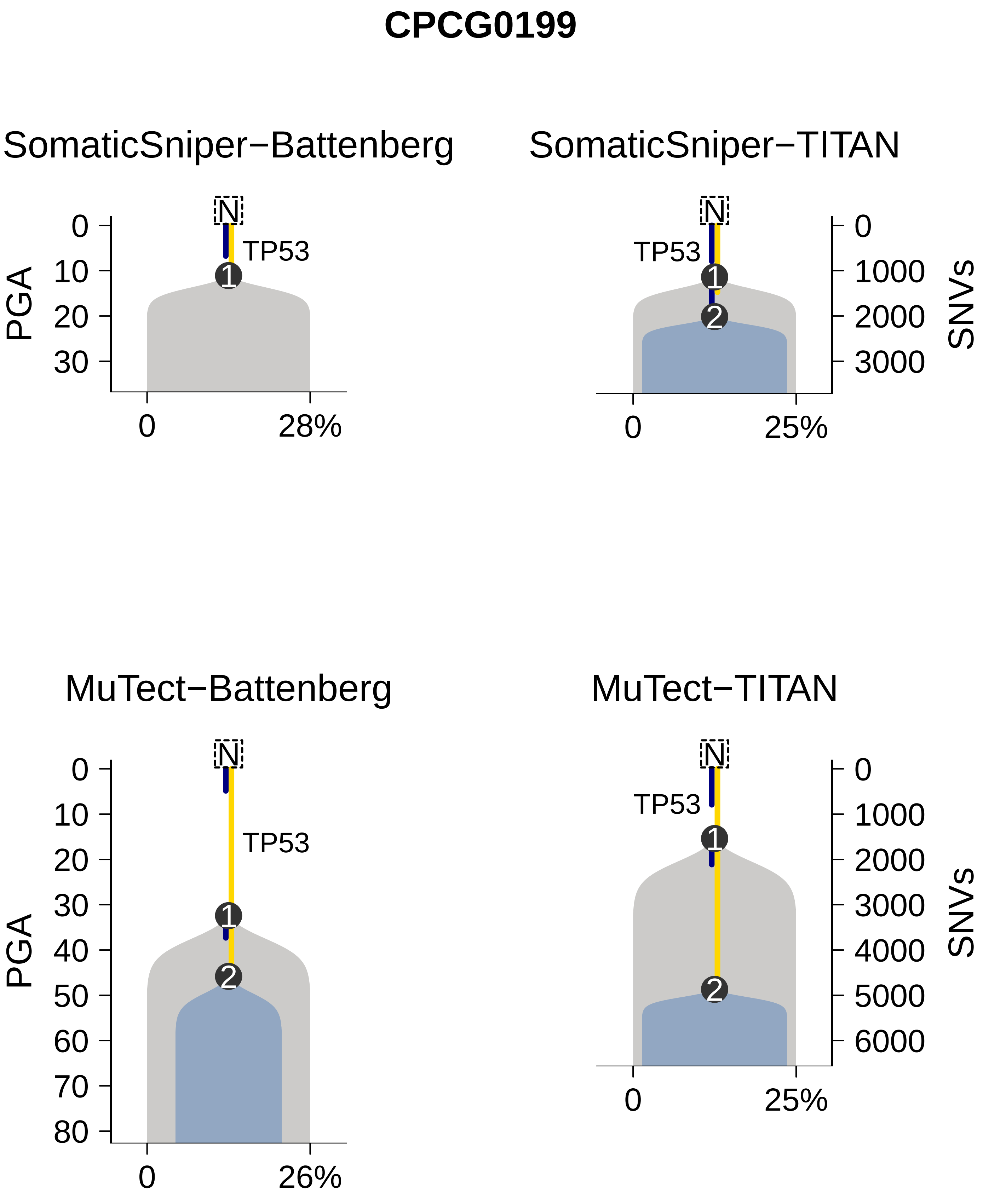
CPCG0199
SomaticSniper−Battenberg
SomaticSniper−TITAN
MuTect−Battenberg
MuTect−TITAN
N
TP53
1
2
PGA
SNVs
0
10
20
30
40
50
60
70
80
1000
2000
3000
4000
5000
6000
28%
25%
26%

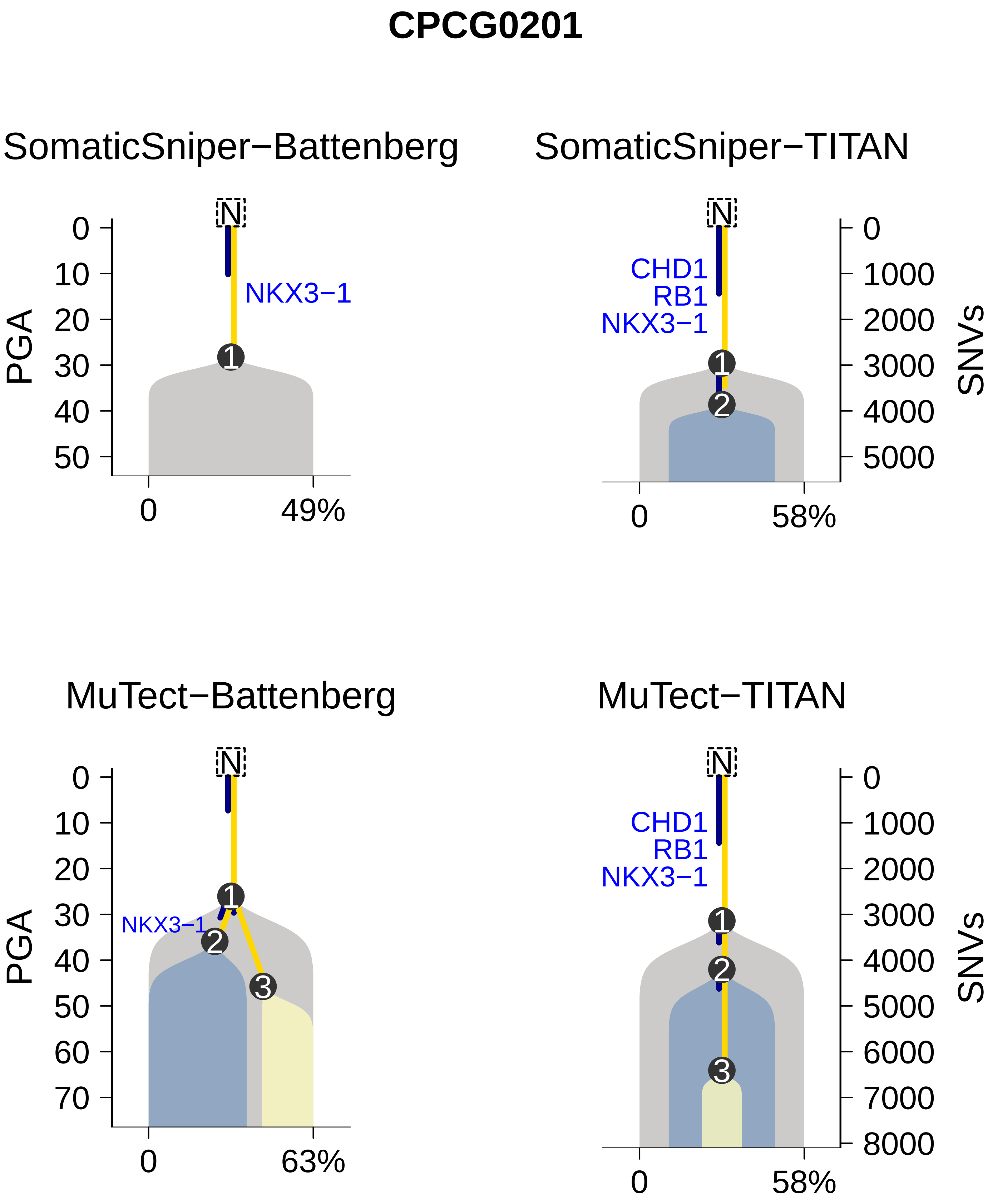
CPCG0201
SomaticSniper−Battenberg
SomaticSniper−TITAN
MuTect−Battenberg
MuTect−TITAN
N
1
2
3
NKX3−1
CHD1
RB1
PGA
SNVs
0
10
20
30
40
50
60
70
1000
2000
3000
4000
5000
6000
7000
8000
49%
58%
63%

CPCG0205

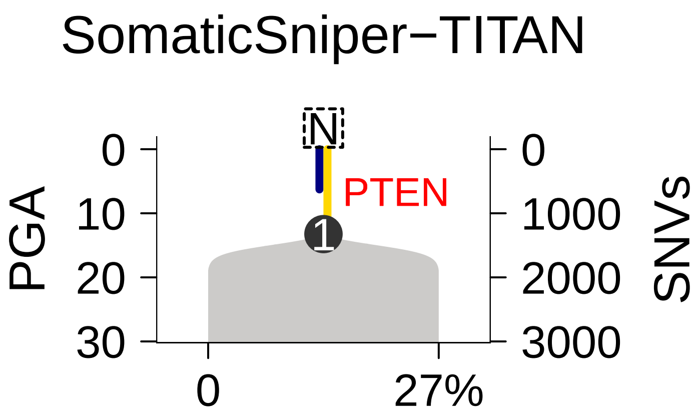
SomaticSniper−TITAN
N
PTEN
1
PGA
0
10
20
30
SNVs
0
1000
2000
3000
0
27%

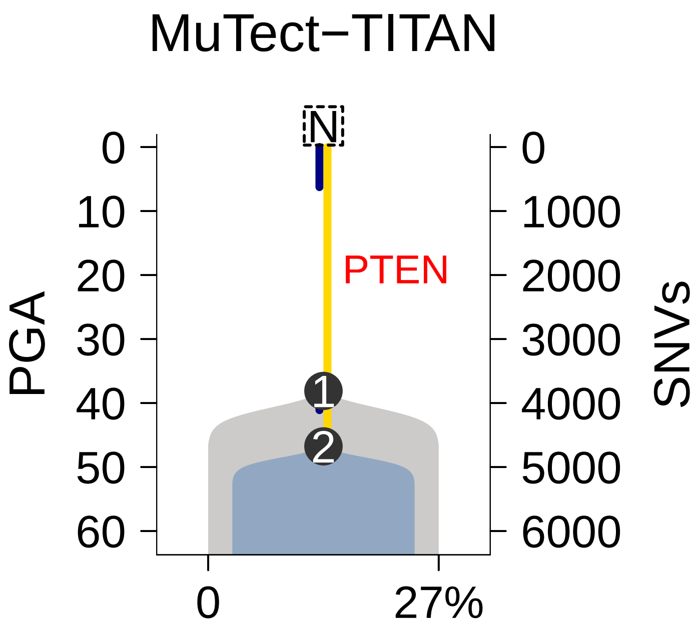
MuTect−TITAN
N
PTEN
1
2
PGA
0
10
20
30
40
50
60
SNVs
0
1000
2000
3000
4000
5000
6000
0
27%

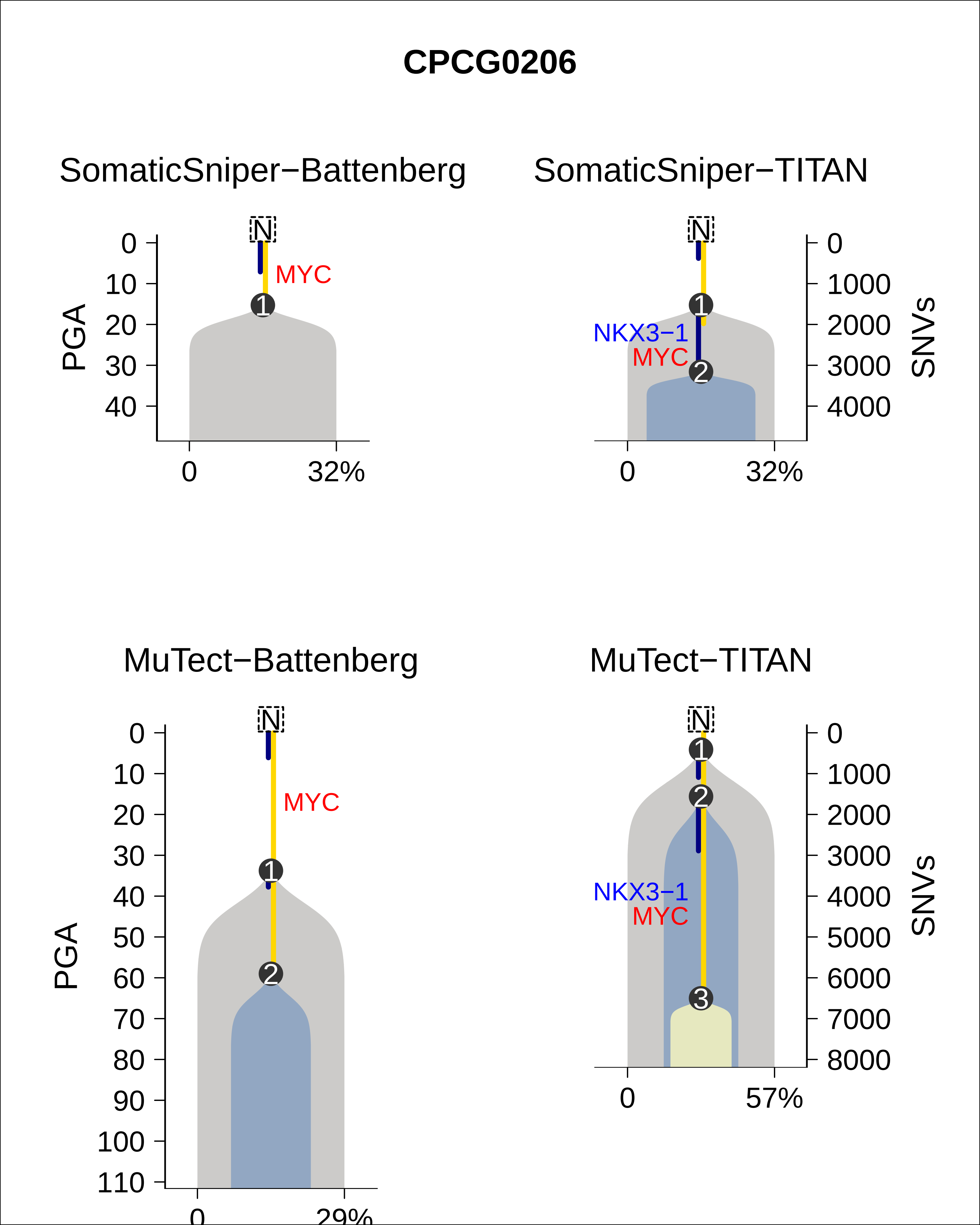
CPCG0206
SomaticSniper−Battenberg
SomaticSniper−TITAN
MuTect−Battenberg
MuTect−TITAN
N
MYC
NKX3−1
PGA
SNVs
0
32%
57%
29%

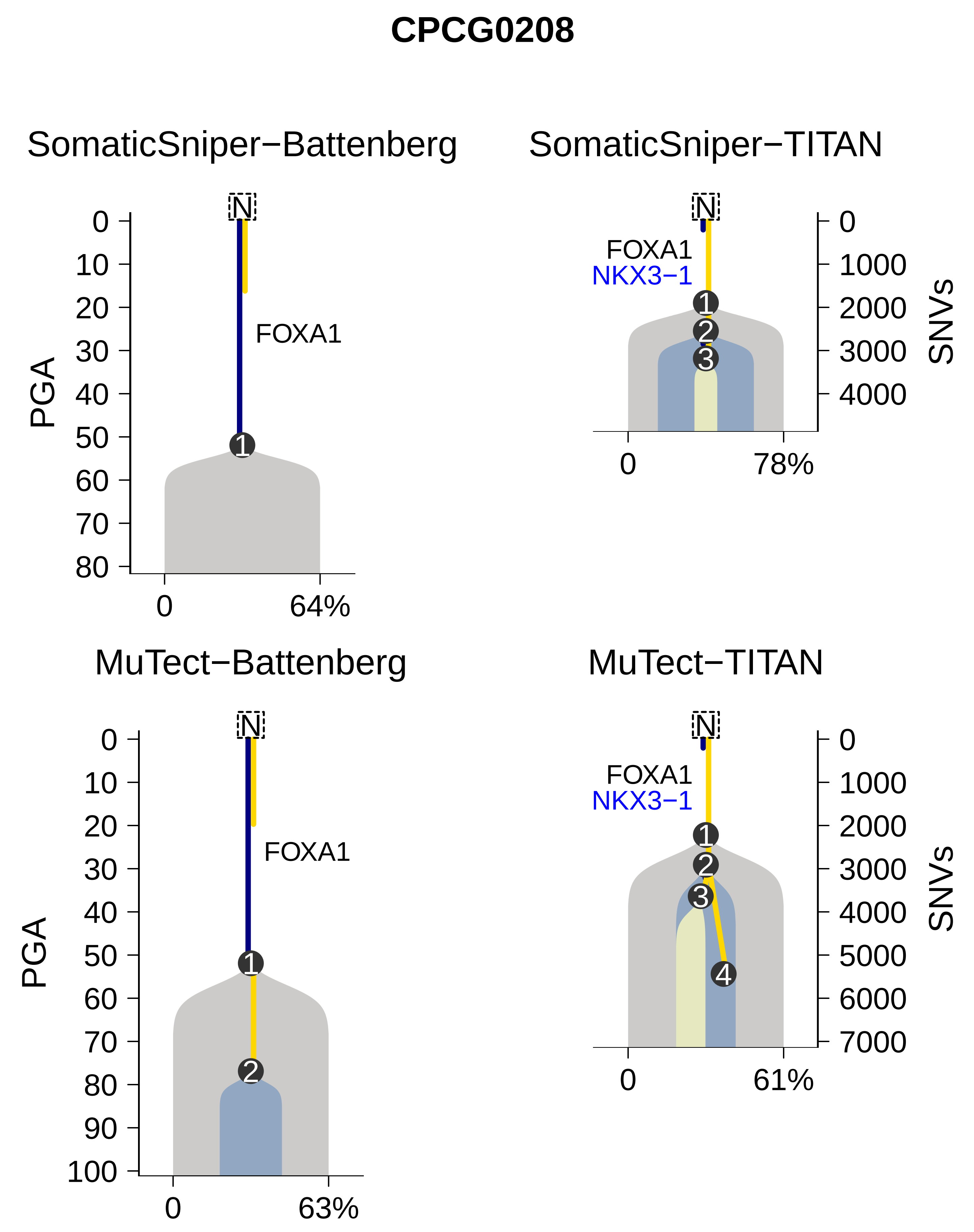
CPCG0208
SomaticSniper−Battenberg
SomaticSniper−TITAN
MuTect−Battenberg
MuTect−TITAN
PGA
SNVs
N
FOXA1
NKX3−1
1
2
3
4
0
10
20
30
40
50
60
70
80
90
100
1000
2000
3000
4000
5000
6000
7000
64%
78%
63%
61%

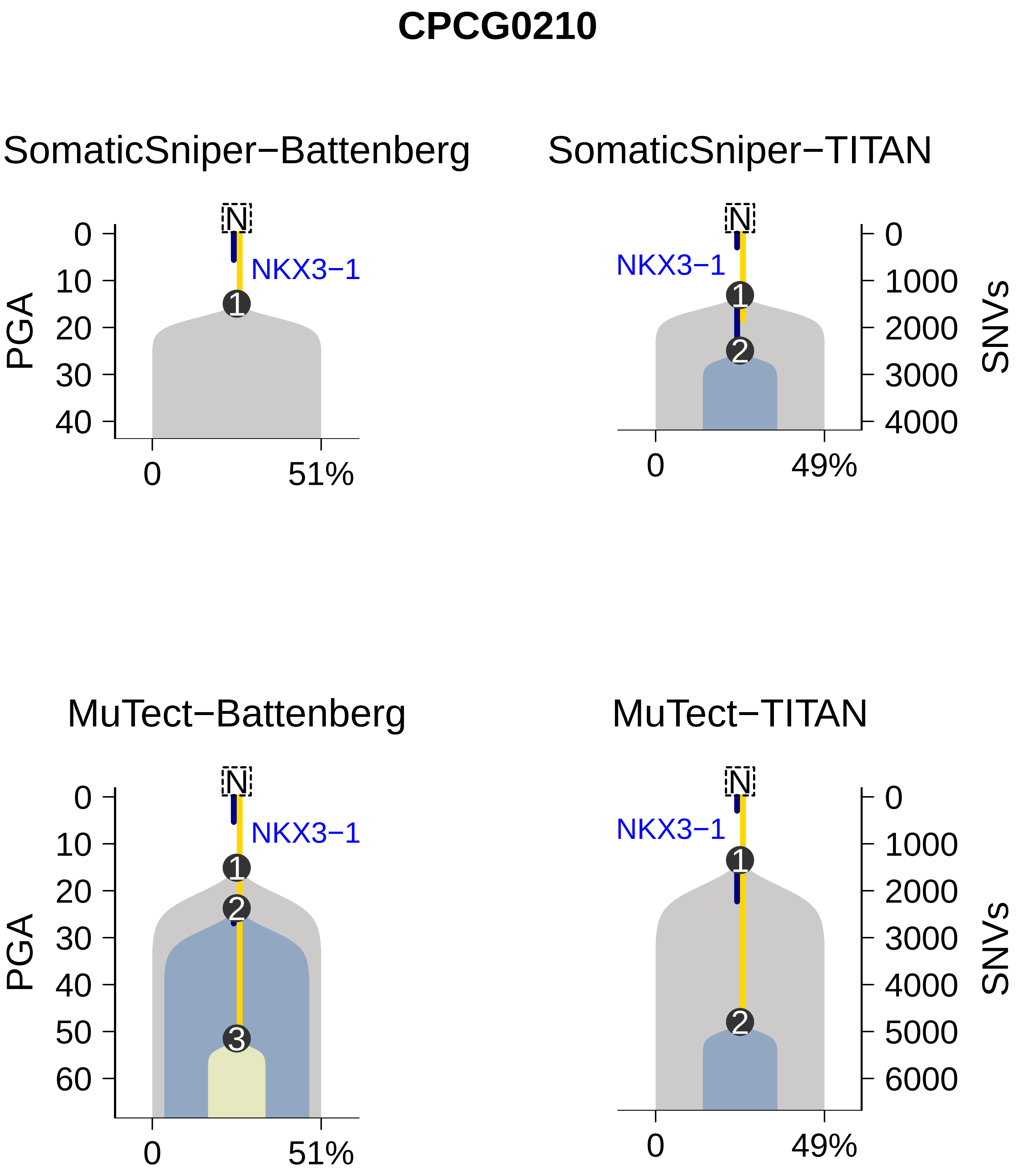
CPCG0210
SomaticSniper−Battenberg
SomaticSniper−TITAN
MuTect−Battenberg
MuTect−TITAN
N
NKX3−1
PGA
SNVs
0
10
20
30
40
50
60
1000
2000
3000
4000
5000
6000
51%
49%
1
2
3

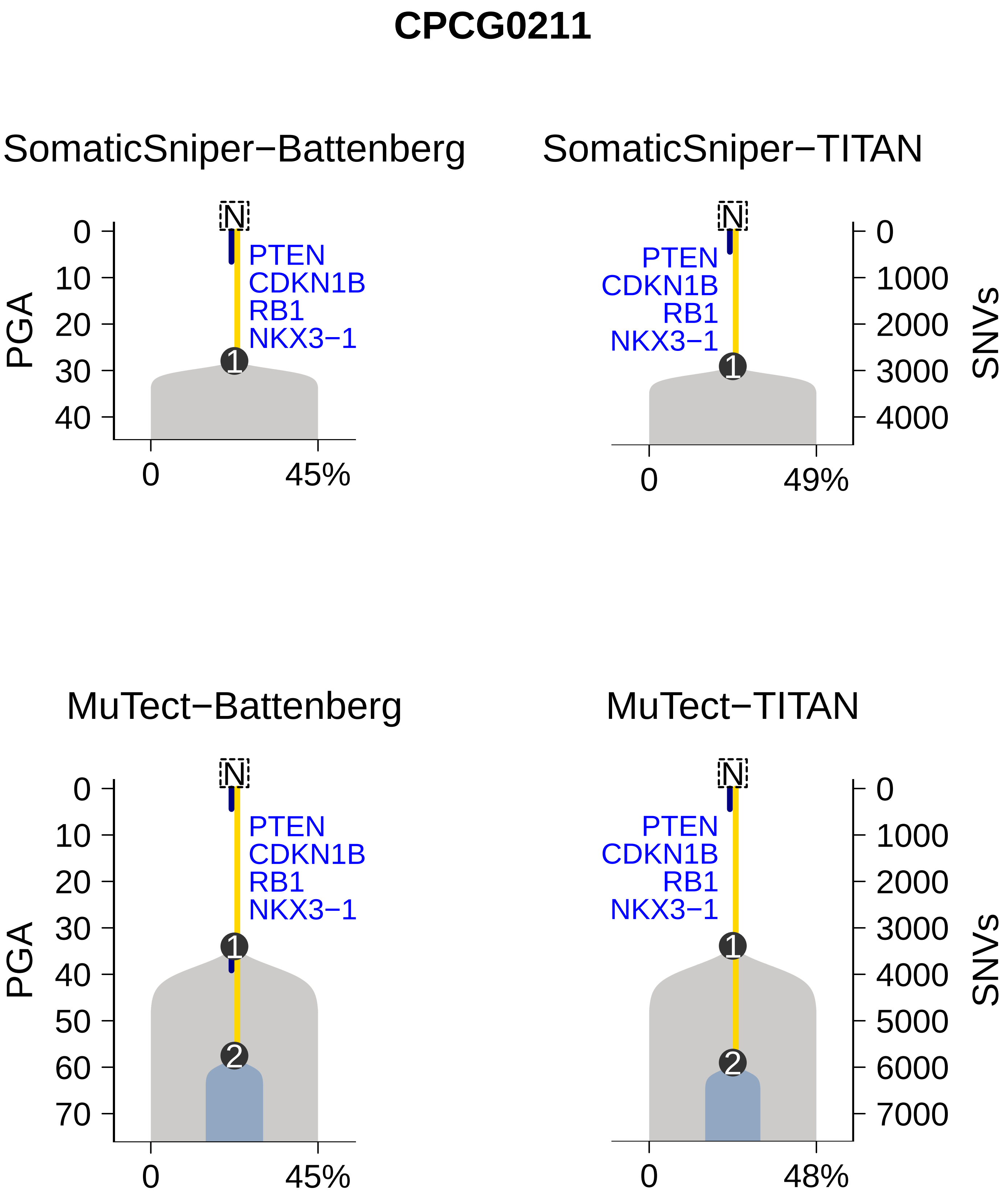
CPCG0211
SomaticSniper−Battenberg
SomaticSniper−TITAN
MuTect−Battenberg
MuTect−TITAN
N
PTEN
CDKN1B
RB1
NKX3−1
1
2
PGA
SNVs
0
10
20
30
40
50
60
70
1000
2000
3000
4000
5000
6000
7000
45%
49%
48%

CPCG0212

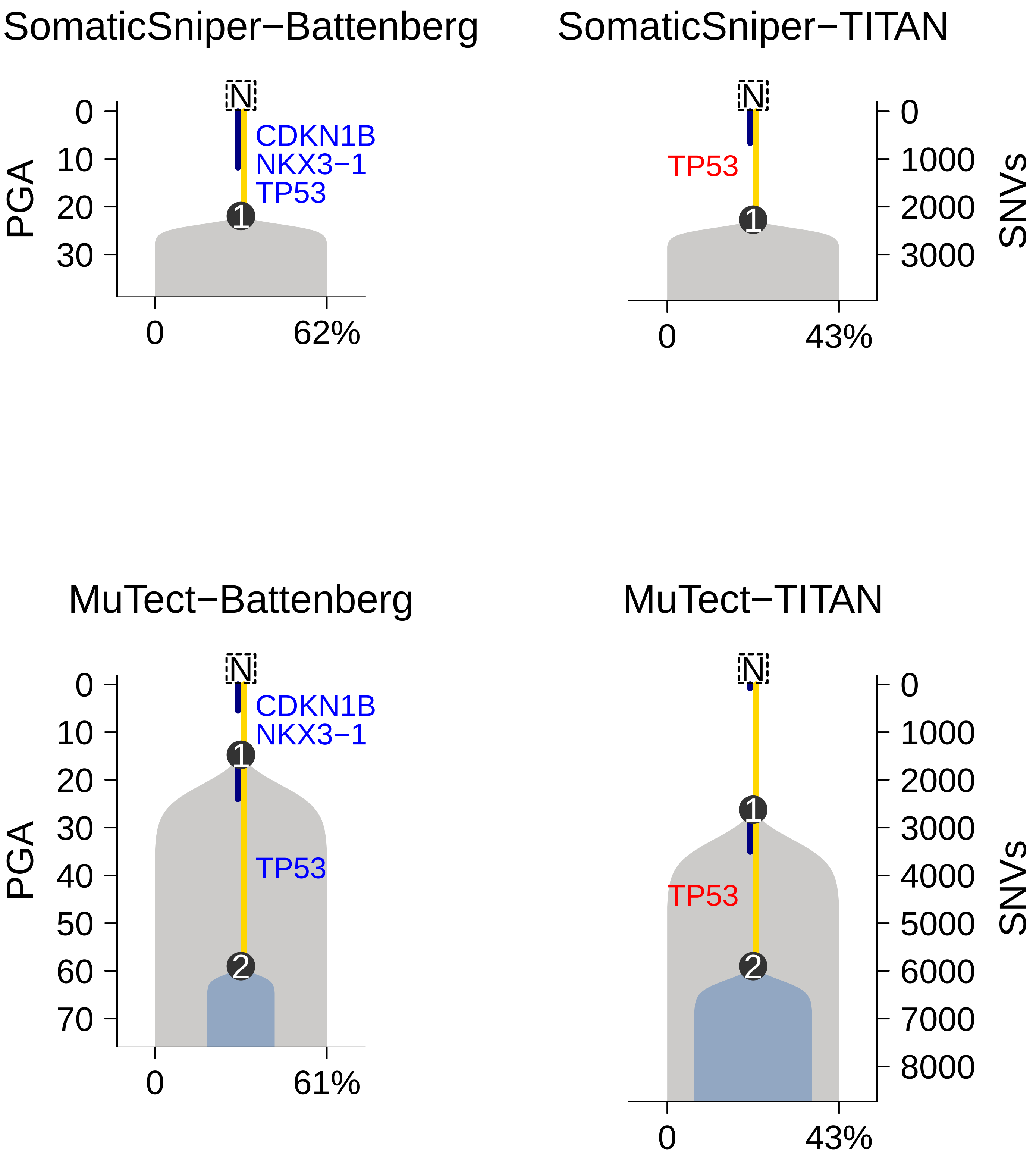
SomaticSniper−Battenberg
SomaticSniper−TITAN
MuTect−Battenberg
MuTect−TITAN
N
1
2
CDKN1B
NKX3−1
TP53
PGA
SNVs
0
10
20
30
40
50
60
70
1000
2000
3000
4000
5000
6000
7000
8000
62%
61%
43%

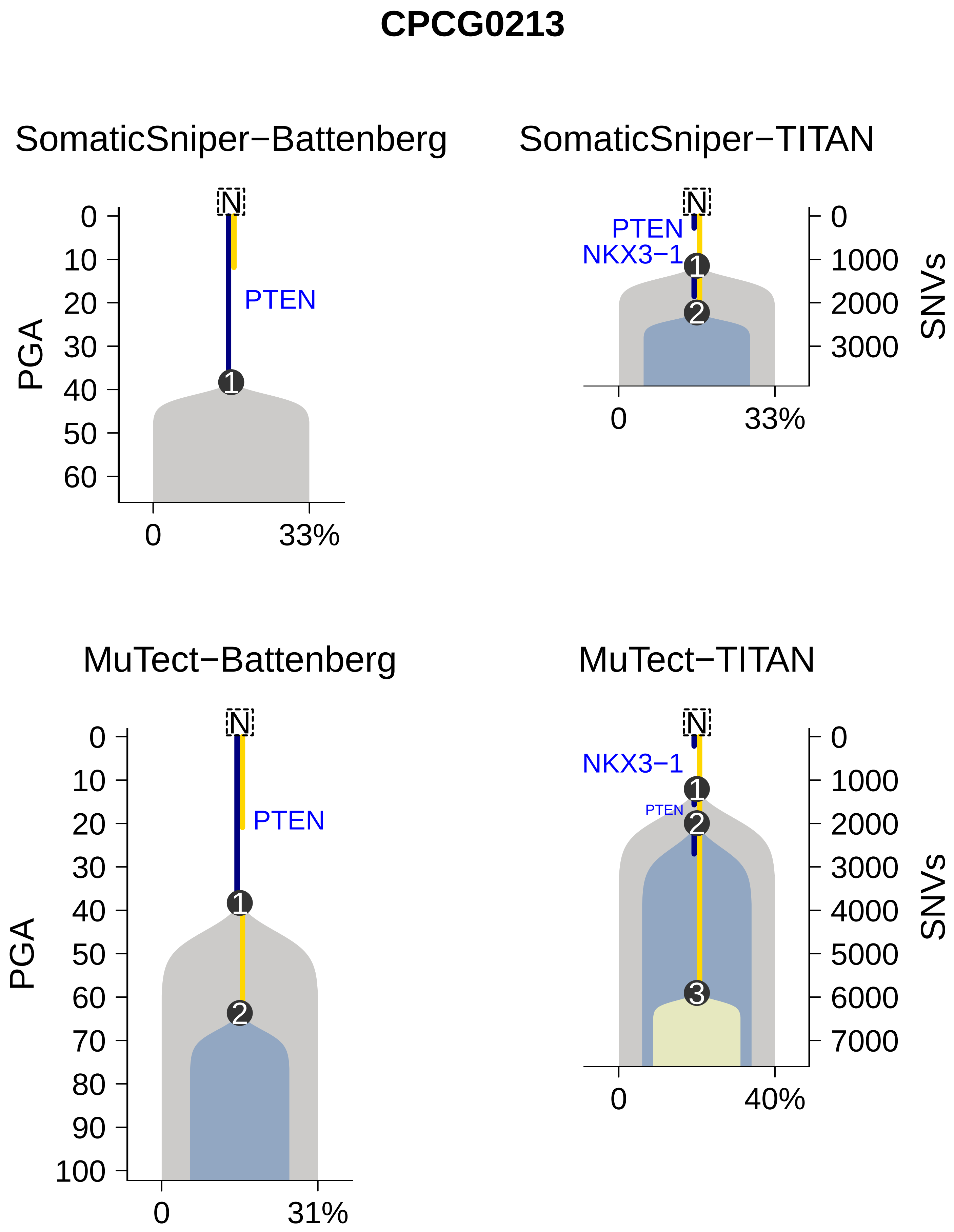

CPCG0213
SomaticSniper−Battenberg
SomaticSniper−TITAN
MuTect−Battenberg
MuTect−TITAN
N
PTEN
NKX3−1
1
2
3
PGA
SNVs
0
10
20
30
40
50
60
70
80
90
100
1000
2000
3000
4000
5000
6000
7000
33%
31%
40%

CPCG0217

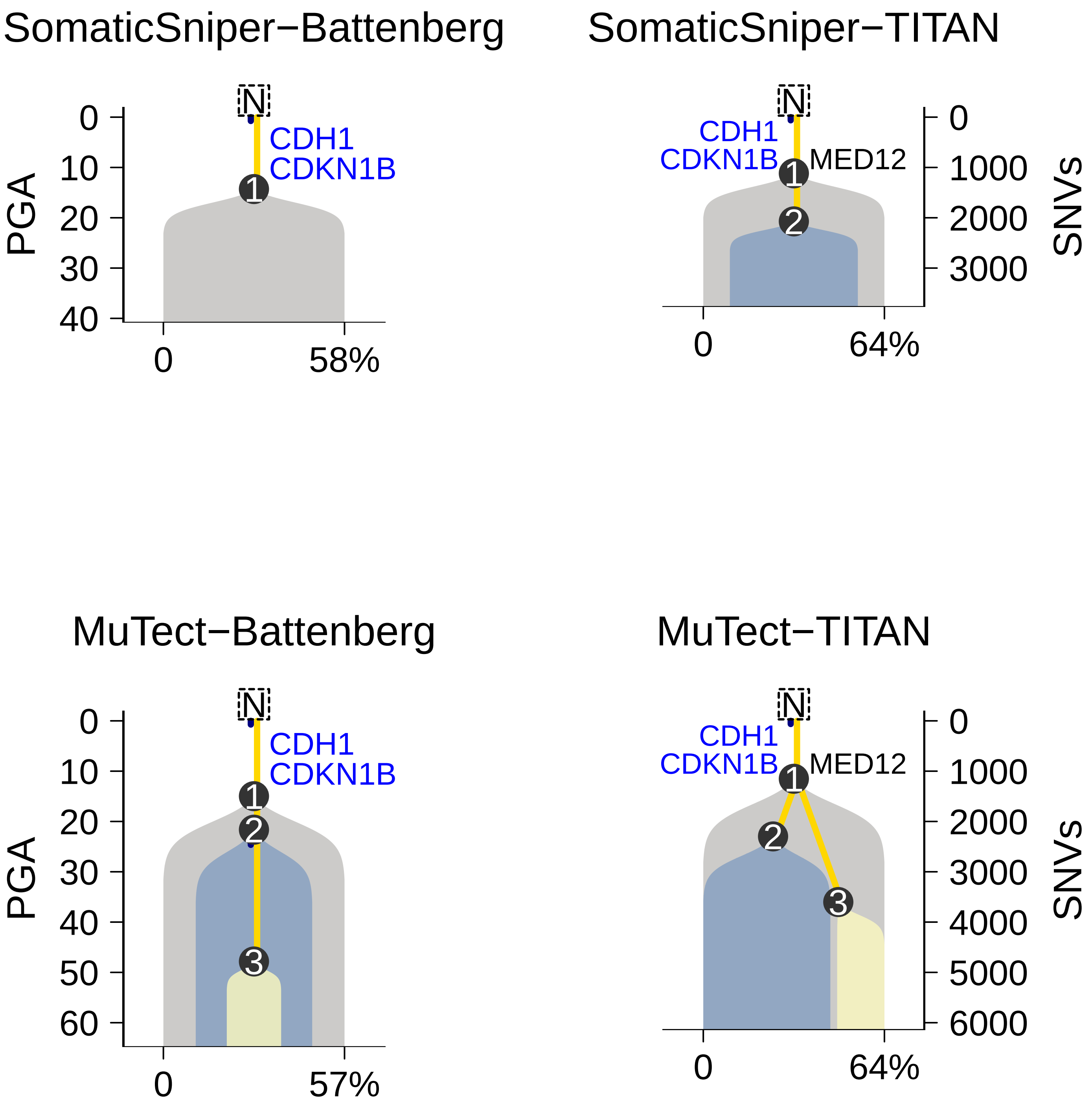

SomaticSniper−Battenberg
N
CDH1
CDKN1B
1
PGA
0
10
20
30
40
0
58%
SomaticSniper−TITAN
N
CDH1
CDKN1B
MED12
1
2
0
1000
2000
3000
SNVs
0
64%
MuTect−Battenberg
N
CDH1
CDKN1B
1
2
3
PGA
0
10
20
30
40
50
60
0
57%
MuTect−TITAN
N
CDH1
CDKN1B
MED12
1
2
3
0
1000
2000
3000
4000
5000
6000
SNVs
0
64%

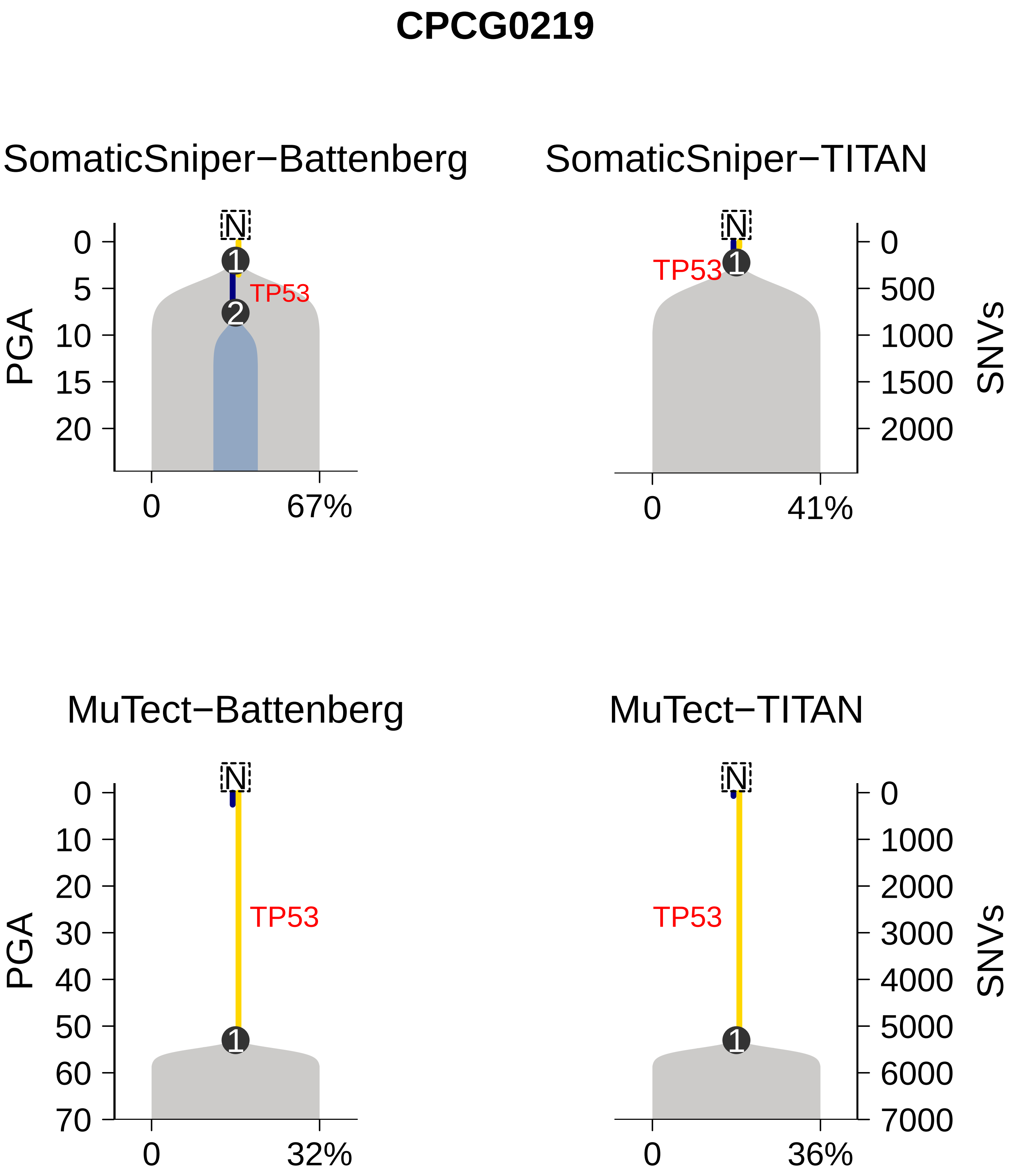
CPCG0219
SomaticSniper−Battenberg
SomaticSniper−TITAN
MuTect−Battenberg
MuTect−TITAN
N
1
2
TP53
PGA
SNVs
0
5
10
15
20
0
500
1000
1500
2000
67%
41%
30
40
50
60
70
3000
4000
5000
6000
7000
32%
36%

CPCG0232

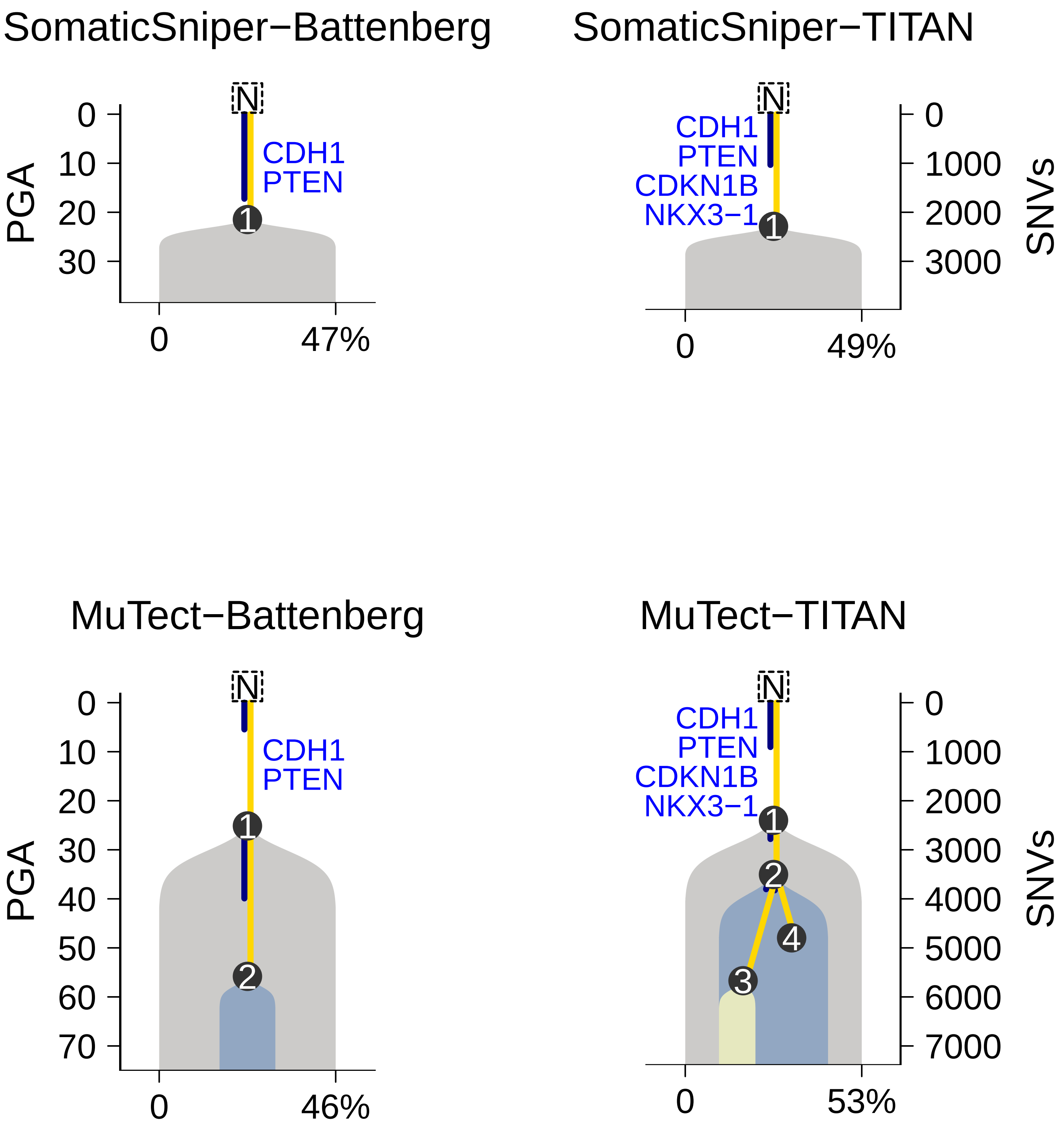
SomaticSniper−Battenberg
SomaticSniper−TITAN
MuTect−Battenberg
MuTect−TITAN
N
1
2
3
4
CDH1
PTEN
CDKN1B
NKX3−1
PGA
SNVs
0
10
20
30
40
50
60
70
1000
2000
3000
4000
5000
6000
7000
47%
49%
46%
53%

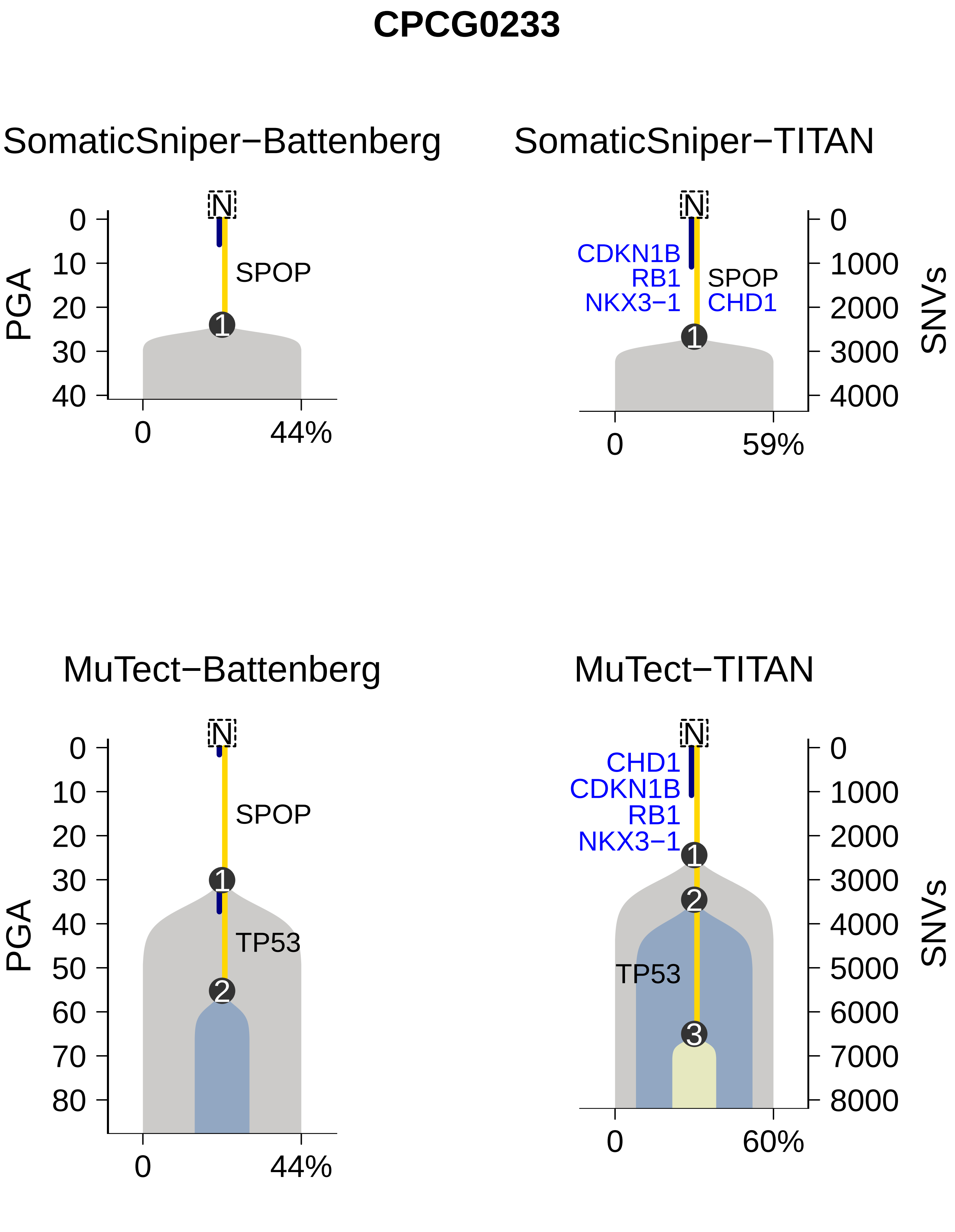
CPCG0233
SomaticSniper−Battenberg
SomaticSniper−TITAN
MuTect−Battenberg
MuTect−TITAN
N
PGA
SNVs
SPOP
CDKN1B
RB1
NKX3−1
CHD1
TP53
0
10
20
30
40
50
60
70
80
0
1000
2000
3000
4000
5000
6000
7000
8000
44%
59%
60%
1
2
3

CPCG0234

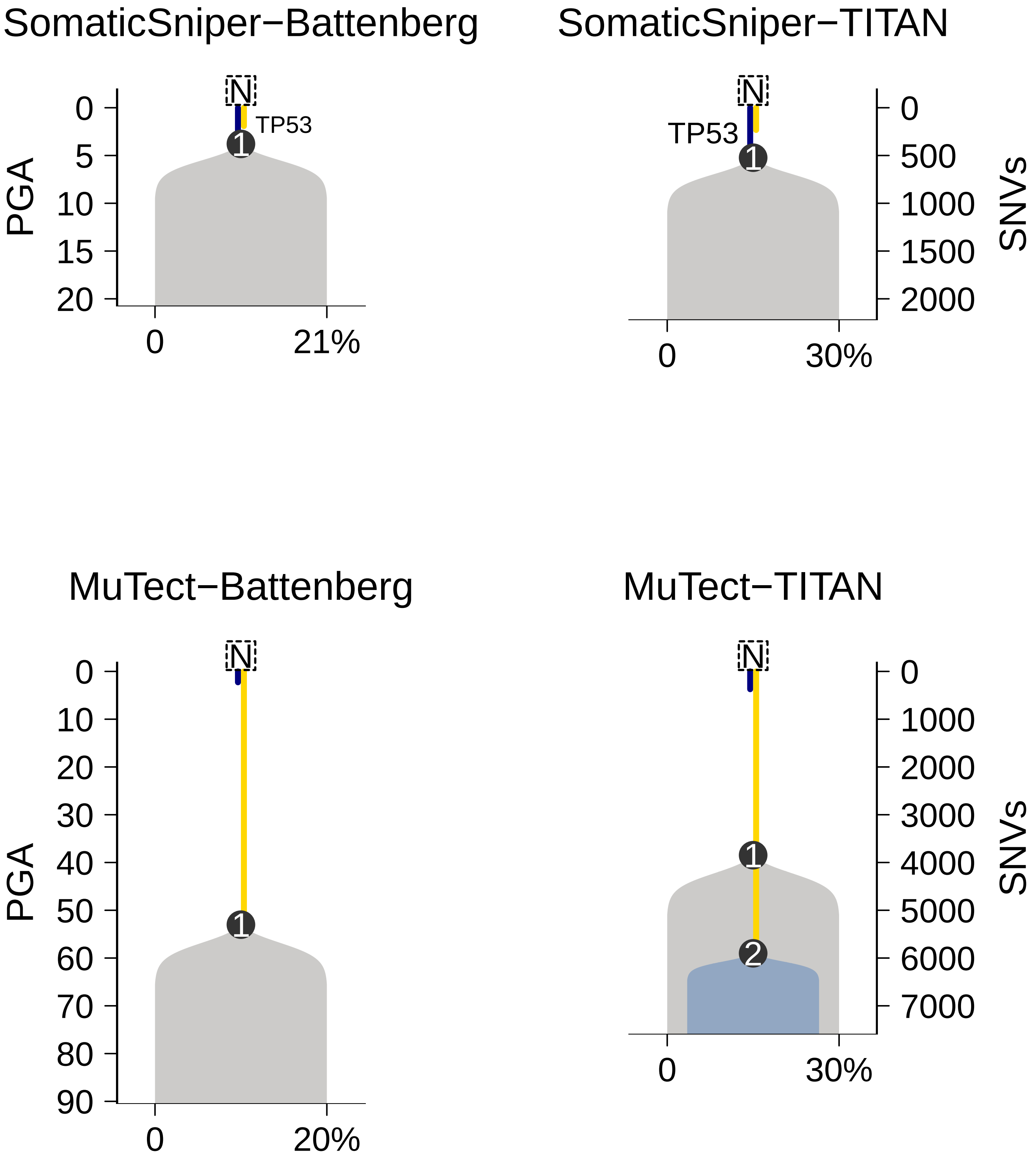

SomaticSniper−Battenberg
SomaticSniper−TITAN
MuTect−Battenberg
MuTect−TITAN
N
TP53
1
2
PGA
SNVs
0
5
10
15
20
30
40
50
60
70
80
90
500
1000
1500
2000
3000
4000
5000
6000
7000
21%
30%
20%

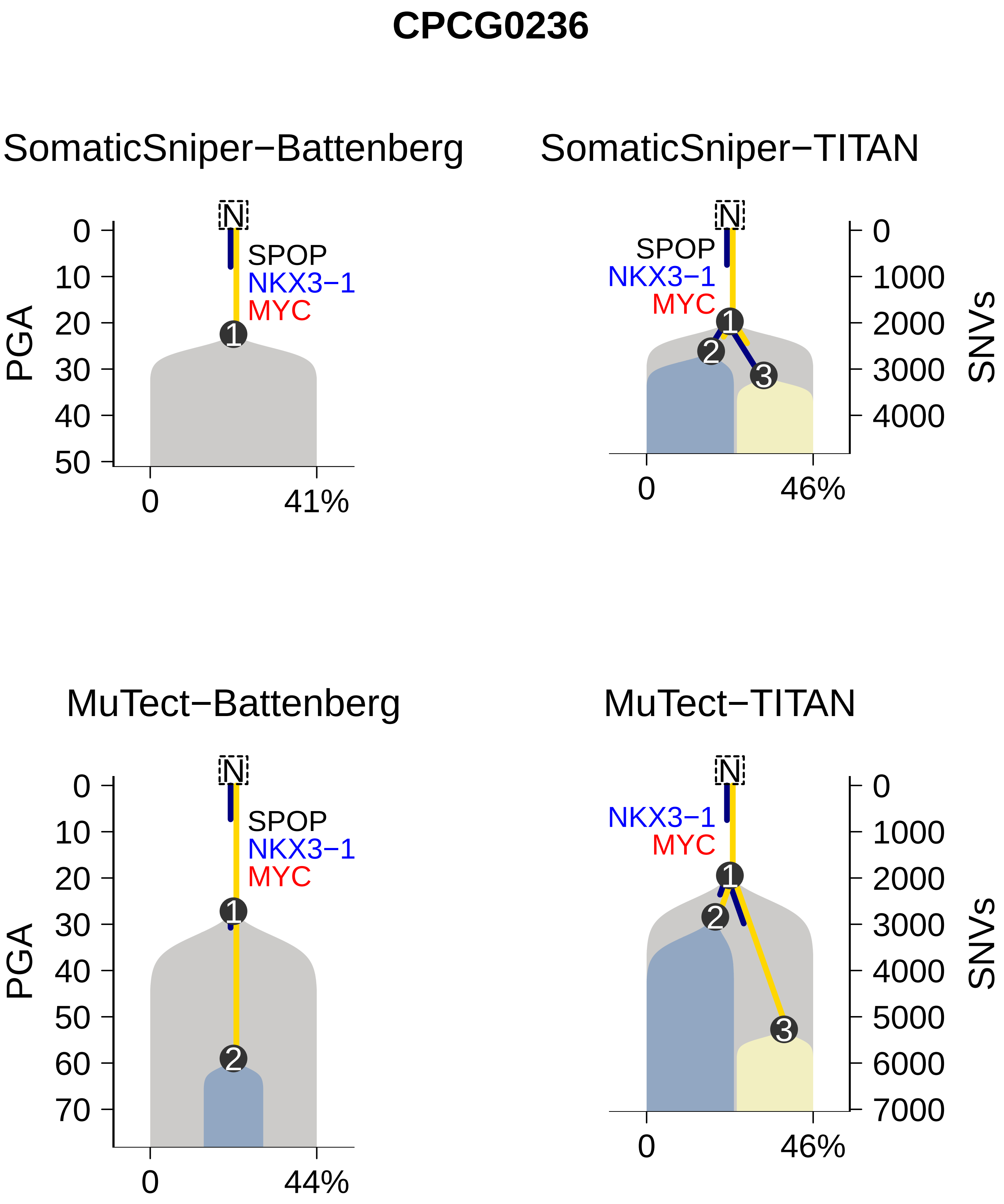

CPCG0236
SomaticSniper−Battenberg
SomaticSniper−TITAN
MuTect−Battenberg
MuTect−TITAN
N
SPOP
NKX3−1
MYC
PGA
SNVs
0
10
20
30
40
50
60
70
1000
2000
3000
4000
5000
6000
7000
1
2
3
41%
44%
46%

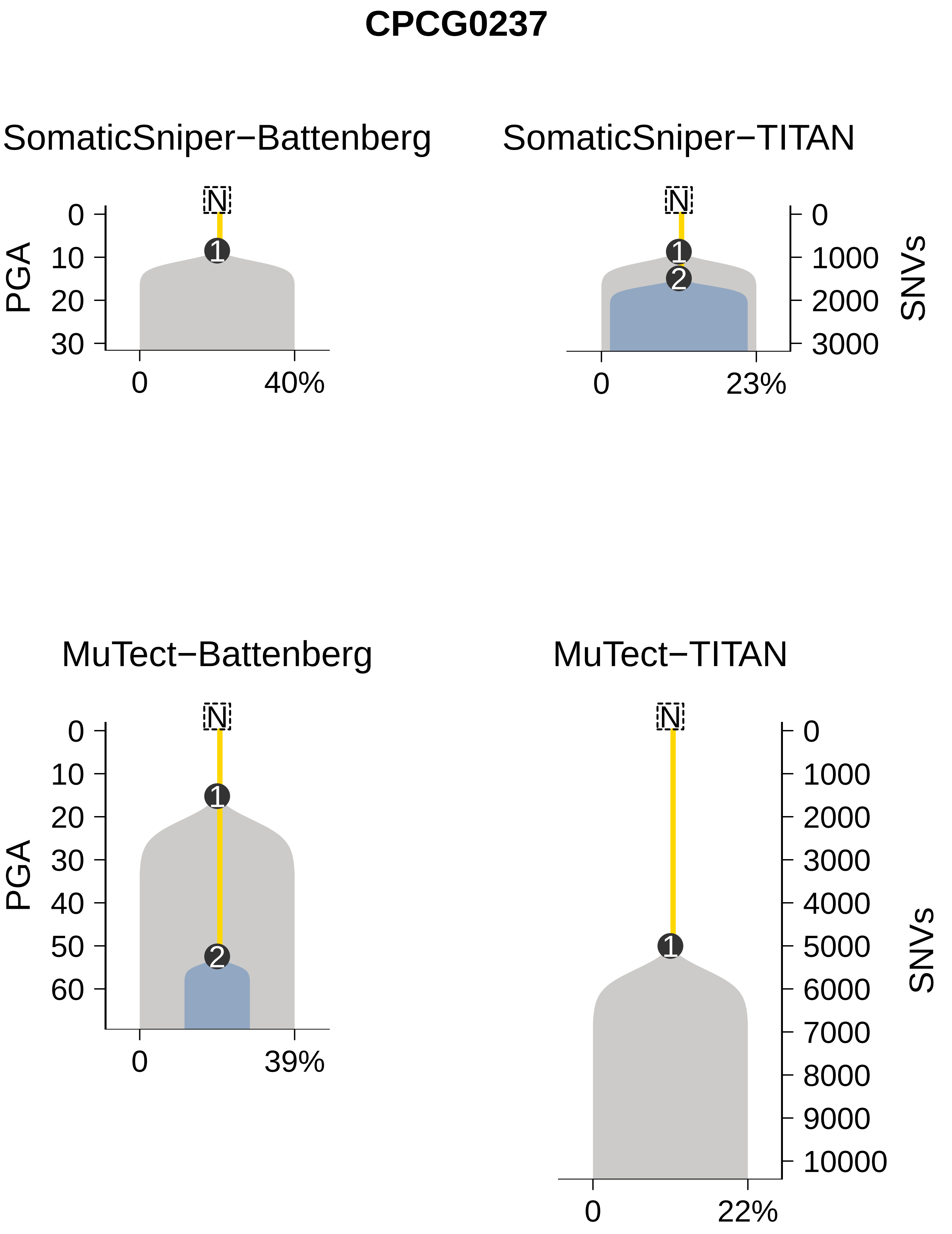
CPCG0237
SomaticSniper−Battenberg
SomaticSniper−TITAN
MuTect−Battenberg
MuTect−TITAN
PGA
SNVs
N
1
2
0
10
20
30
40
50
60
40%
23%
39%
22%
1000
2000
3000
4000
5000
6000
7000
8000
9000
10000

CPCG0238

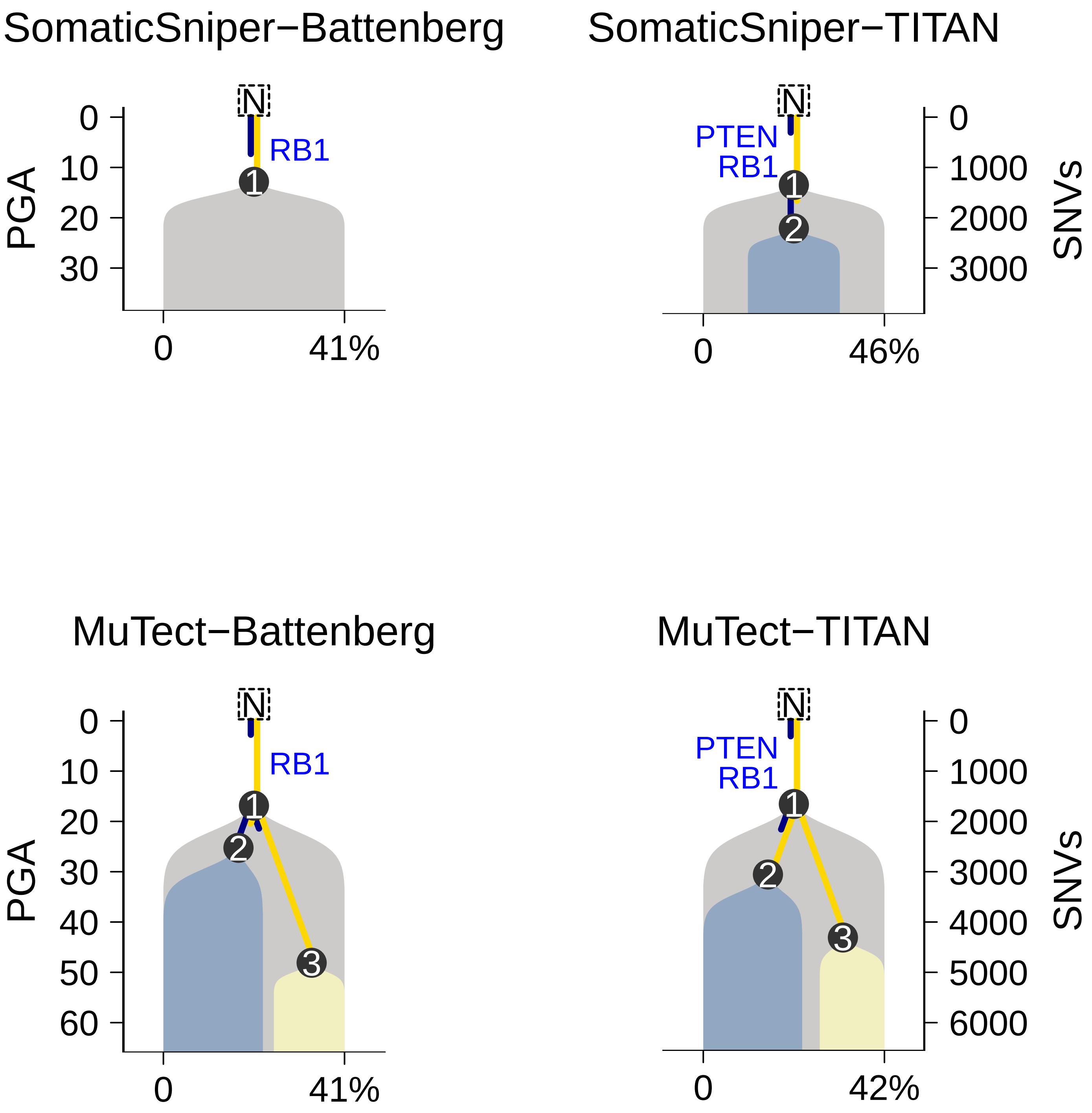

SomaticSniper−Battenberg
SomaticSniper−TITAN
N
RB1
PTEN
RB1
PGA
SNVs
0
10
20
30
0
1000
2000
3000
0
41%
0
46%
MuTect−Battenberg
MuTect−TITAN
N
RB1
PTEN
RB1
PGA
SNVs
0
10
20
30
40
50
60
0
1000
2000
3000
4000
5000
6000
0
41%
0
42%

CPCG0241

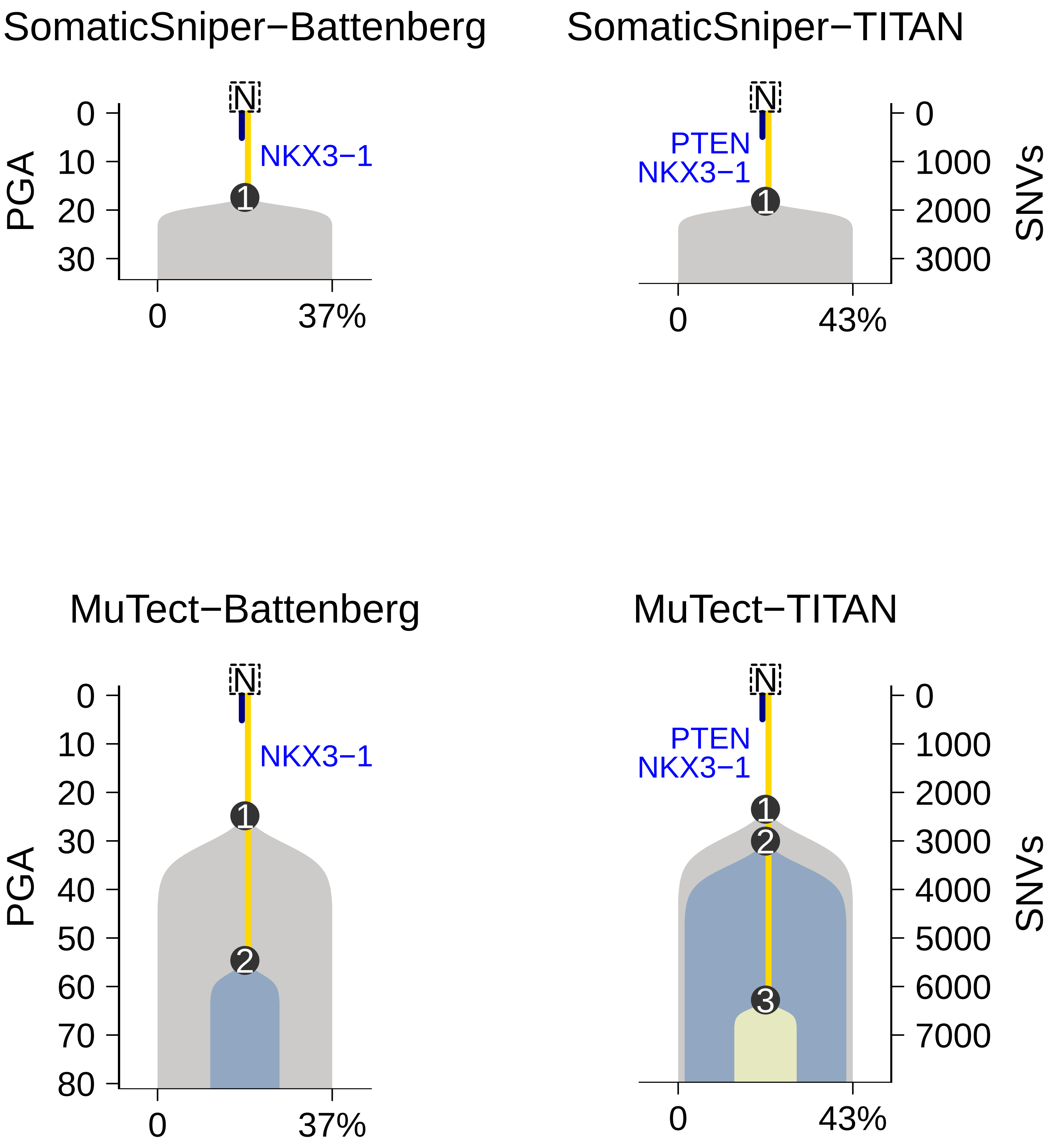

SomaticSniper−Battenberg
SomaticSniper−TITAN
MuTect−Battenberg
MuTect−TITAN
N
NKX3−1
PTEN
PGA
SNVs
0
10
20
30
40
50
60
70
80
1000
2000
3000
4000
5000
6000
7000
37%
43%
1
2
3

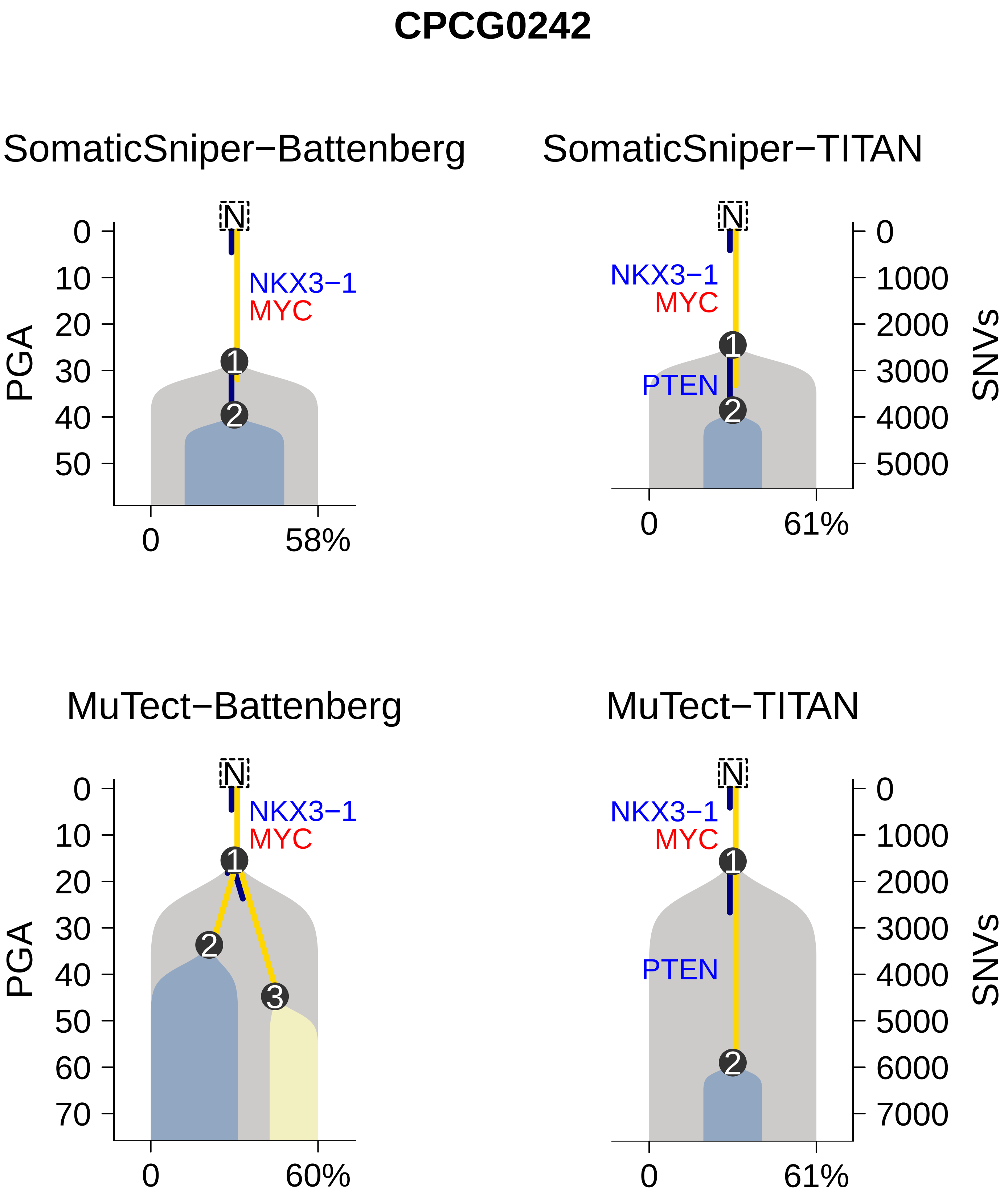
CPCG0242
SomaticSniper−Battenberg
SomaticSniper−TITAN
MuTect−Battenberg
MuTect−TITAN
N
NKX3−1
MYC
PTEN
PGA
SNVs
0
10
20
30
40
50
60
70
1000
2000
3000
4000
5000
6000
7000
58%
61%
60%
1
2
3

CPCG0243

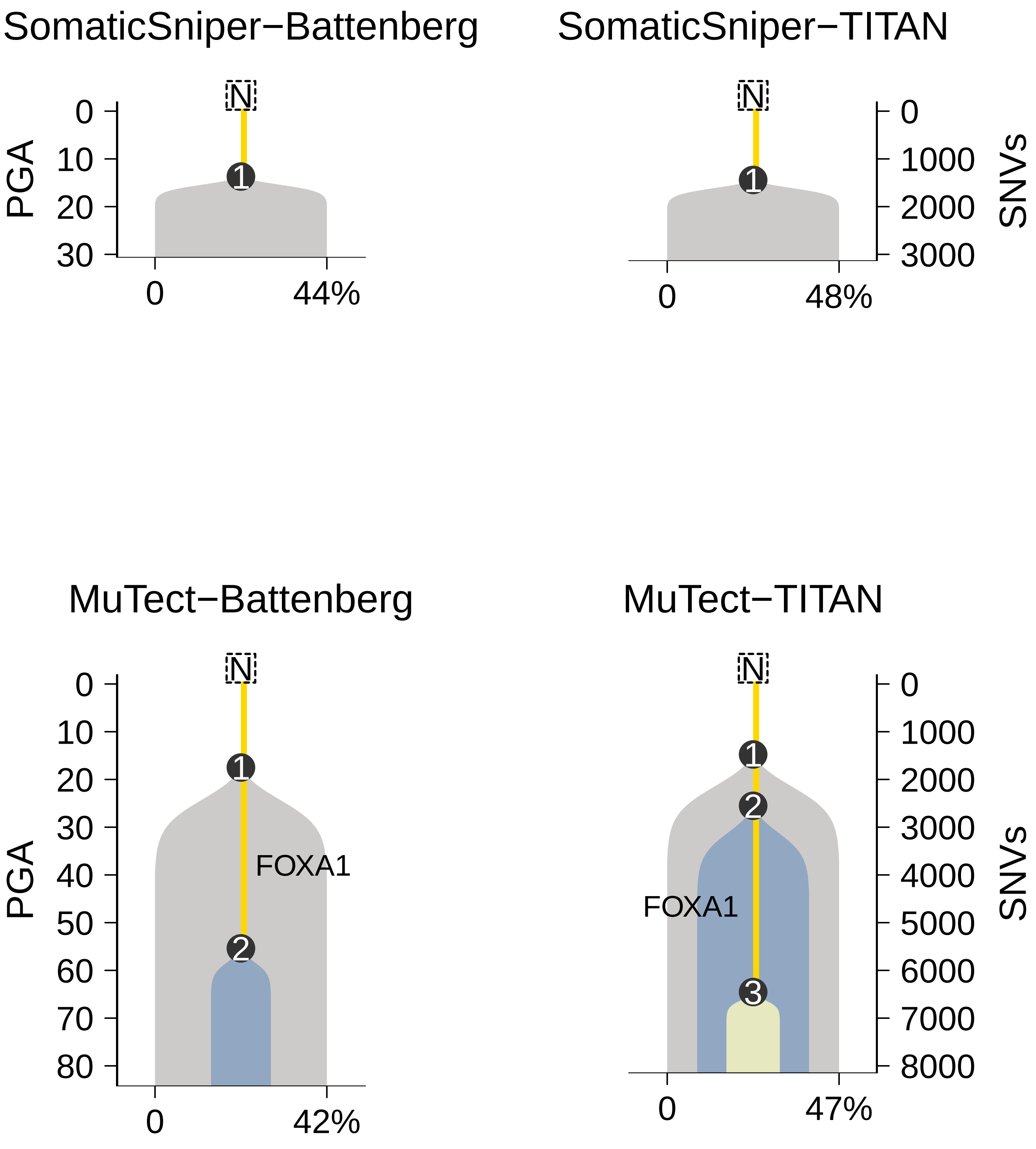

SomaticSniper−Battenberg
SomaticSniper−TITAN
N
1
PGA
SNVs
0
10
20
30
0
1000
2000
3000
0
44%
0
48%
MuTect−Battenberg
MuTect−TITAN
N
1
2
3
FOXA1
FOXA1
PGA
SNVs
0
10
20
30
40
50
60
70
80
0
1000
2000
3000
4000
5000
6000
7000
8000
0
42%
0
47%

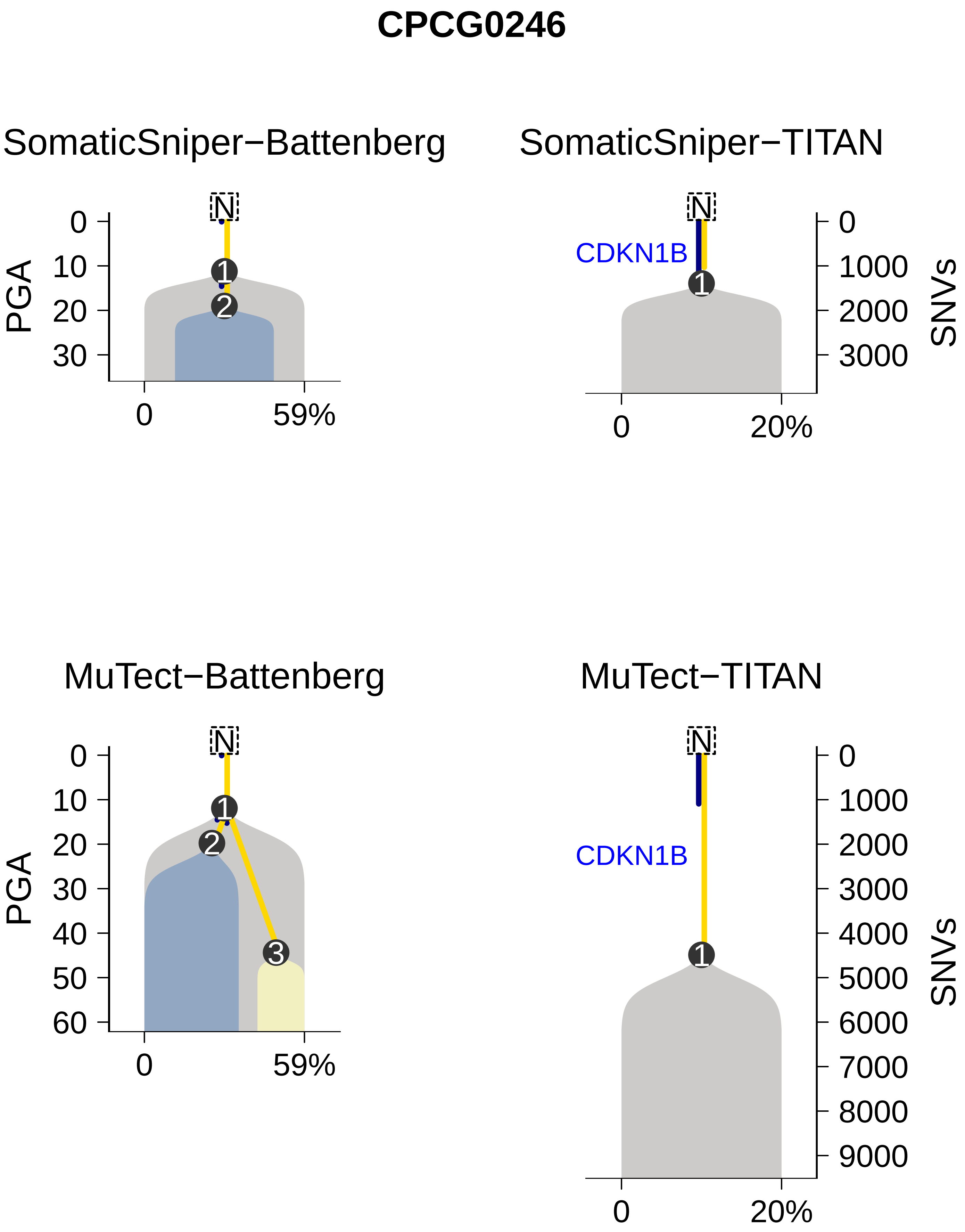
CPCG0246
SomaticSniper−Battenberg
SomaticSniper−TITAN
MuTect−Battenberg
MuTect−TITAN
N
1
2
3
CDKN1B
PGA
SNVs
0
10
20
30
40
50
60
59%
20%
1000
2000
3000
4000
5000
6000
7000
8000
9000

CPCG0248

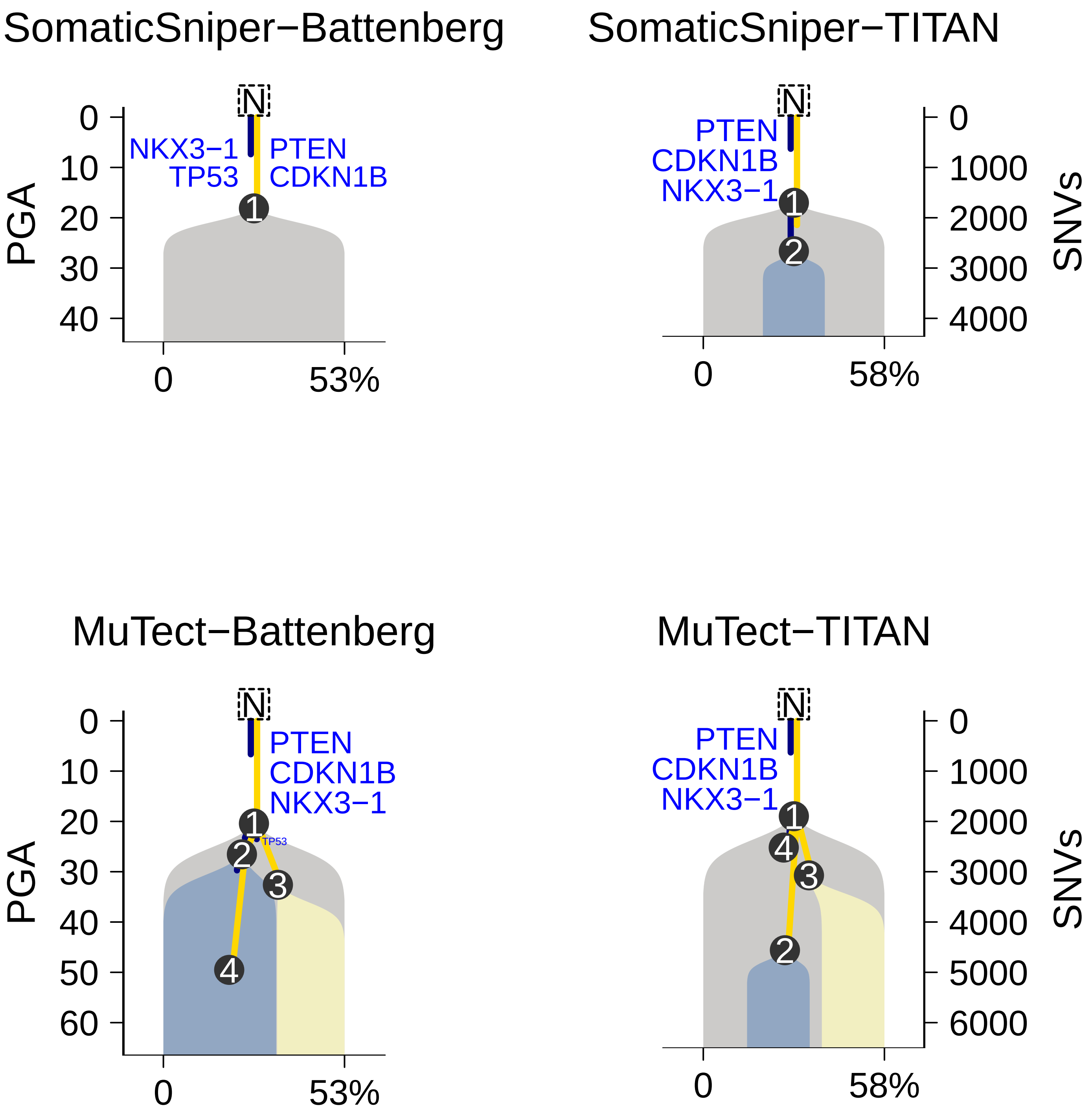
SomaticSniper−Battenberg
SomaticSniper−TITAN
MuTect−Battenberg
MuTect−TITAN
PGA
SNVs
N
NKX3−1
TP53
PTEN
CDKN1B
0
10
20
30
40
50
60
1000
2000
3000
4000
5000
6000
53%
58%

CPCG0249

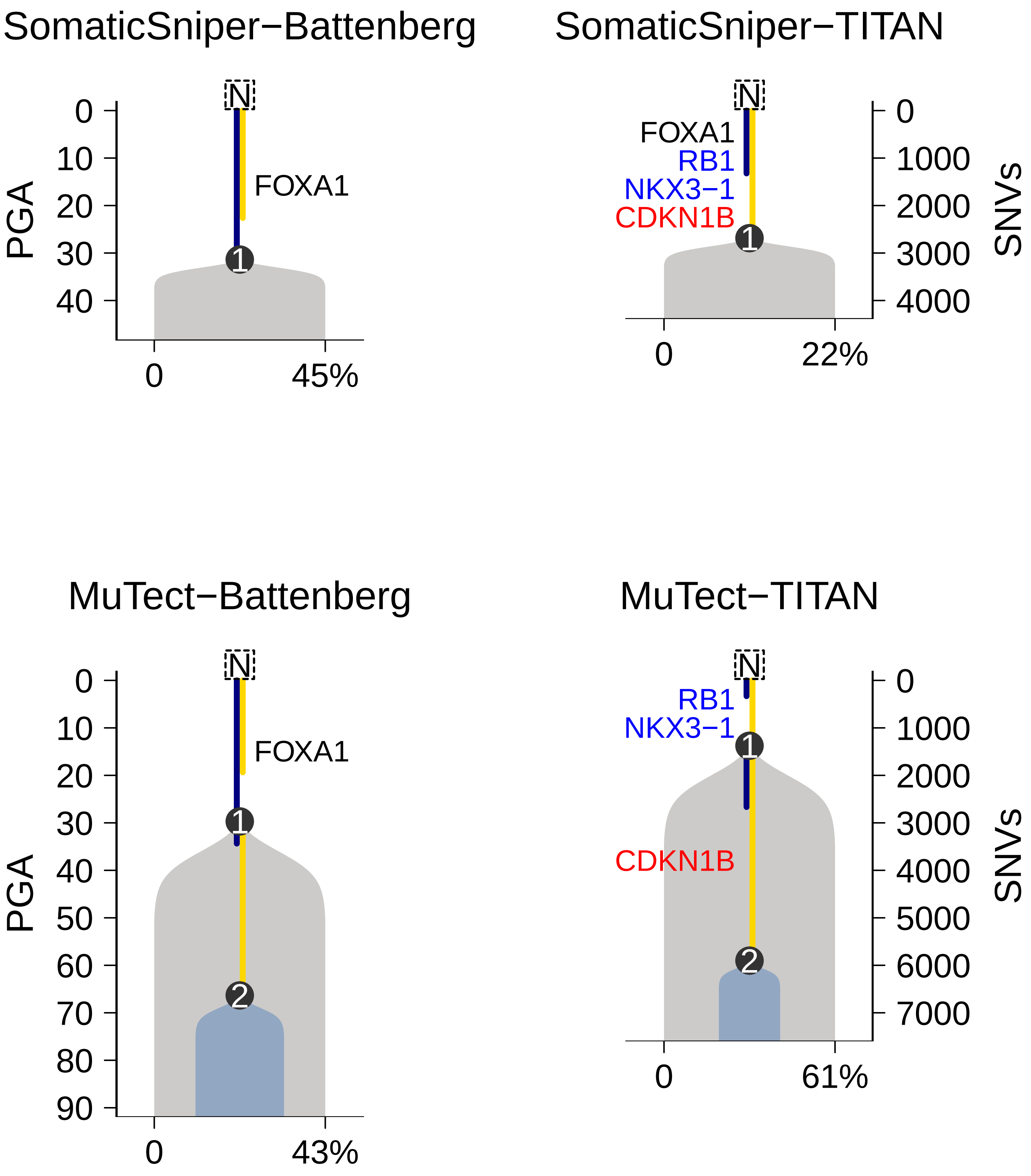

SomaticSniper−Battenberg
N
FOXA1
1
PGA
0
10
20
30
40
0
45%
SomaticSniper−TITAN
N
FOXA1
RB1
NKX3−1
CDKN1B
1
0
1000
2000
3000
4000
SNVs
0
22%
MuTect−Battenberg
N
FOXA1
1
2
PGA
0
10
20
30
40
50
60
70
80
90
0
43%
MuTect−TITAN
N
RB1
NKX3−1
1
CDKN1B
2
0
1000
2000
3000
4000
5000
6000
7000
SNVs
0
61%

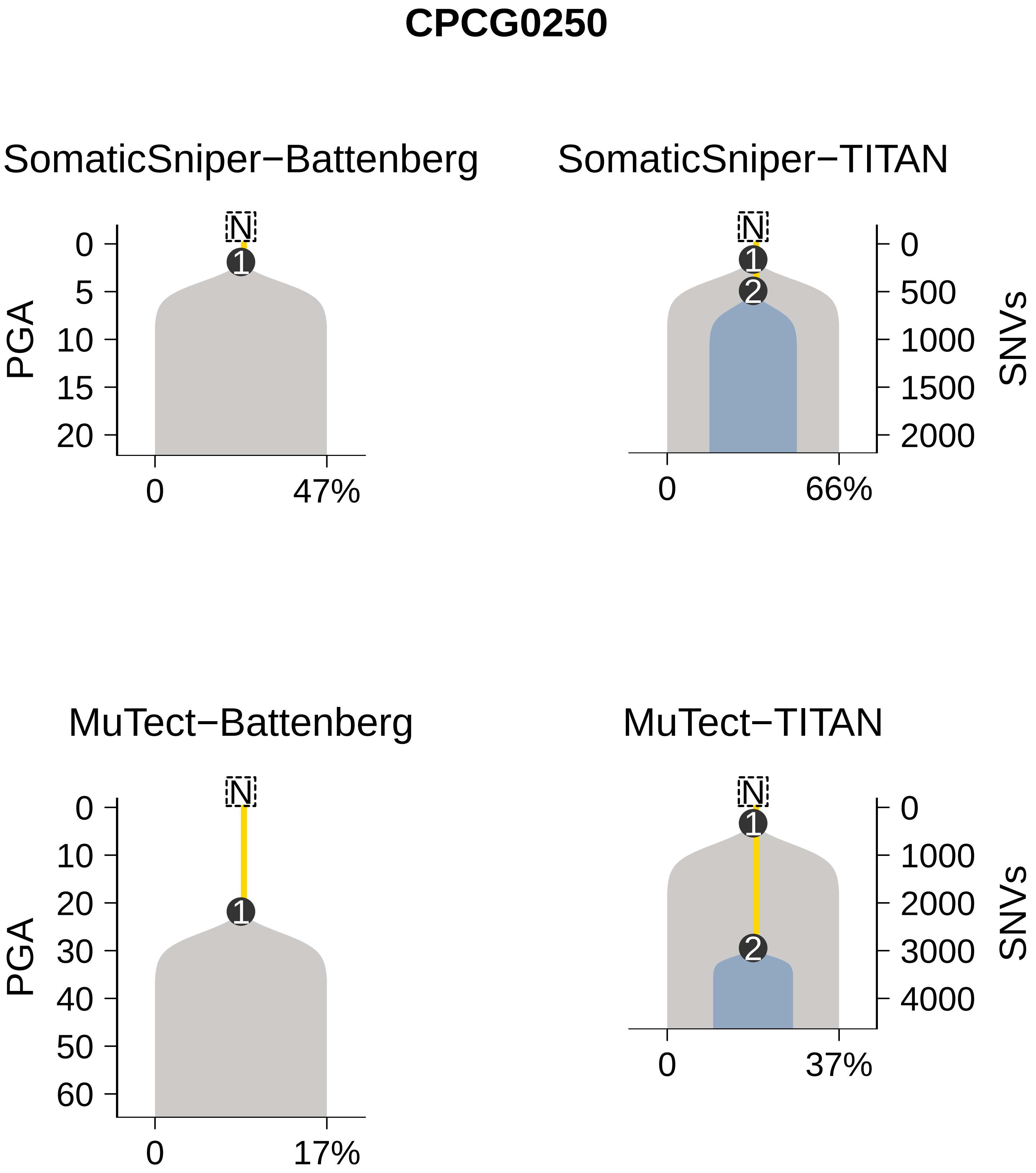
CPCG0250
SomaticSniper−Battenberg
SomaticSniper−TITAN
N
1
2
PGA
SNVs
0
5
10
15
20
500
1000
1500
2000
47%
66%
MuTect−Battenberg
MuTect−TITAN
30
40
50
60
3000
4000
17%
37%

CPCG0251

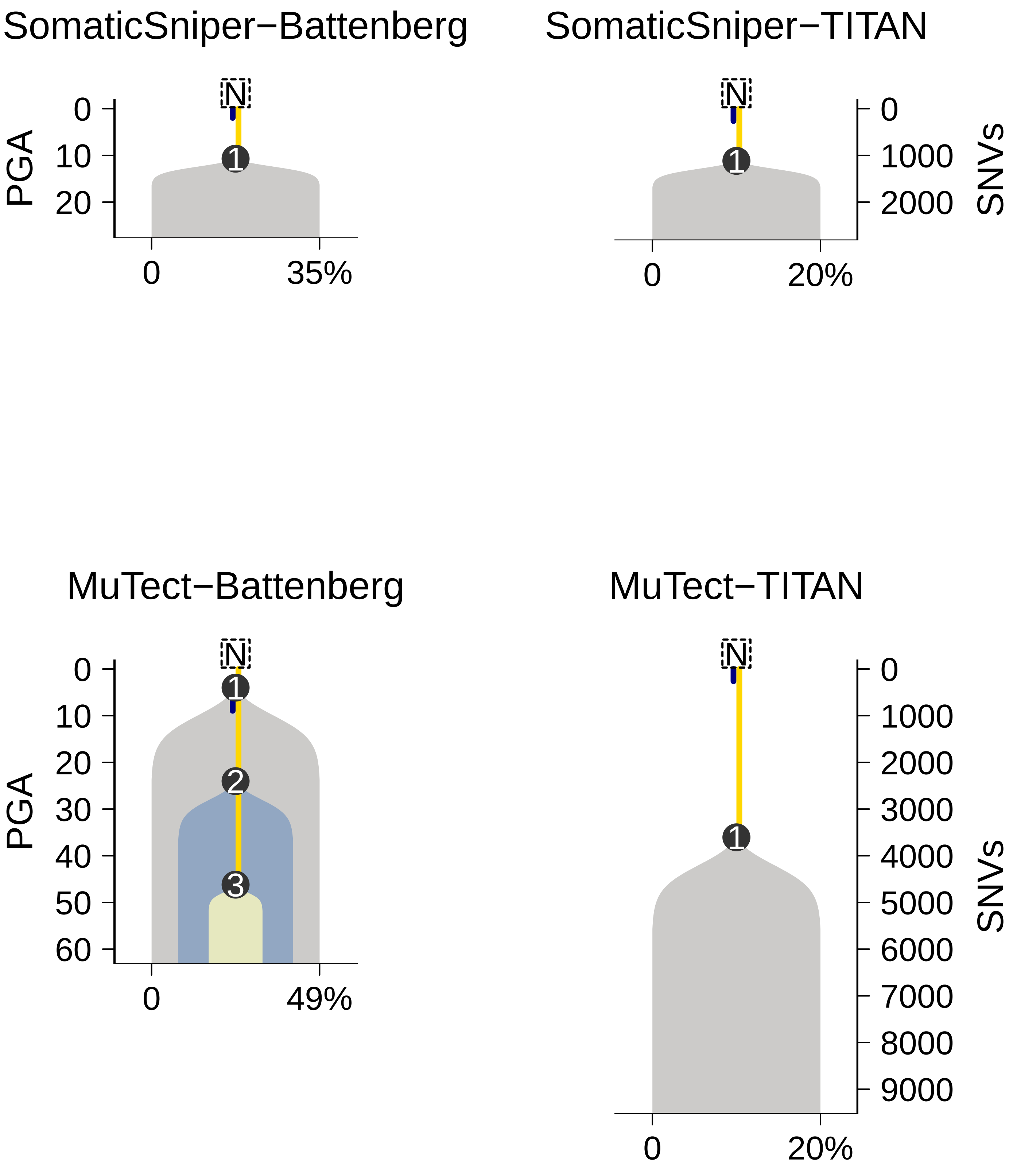

SomaticSniper−Battenberg
SomaticSniper−TITAN
MuTect−Battenberg
MuTect−TITAN
N
1
2
3
PGA
SNVs
0
10
20
30
40
50
60
1000
2000
3000
4000
5000
6000
7000
8000
9000
35%
20%
49%

CPCG0255

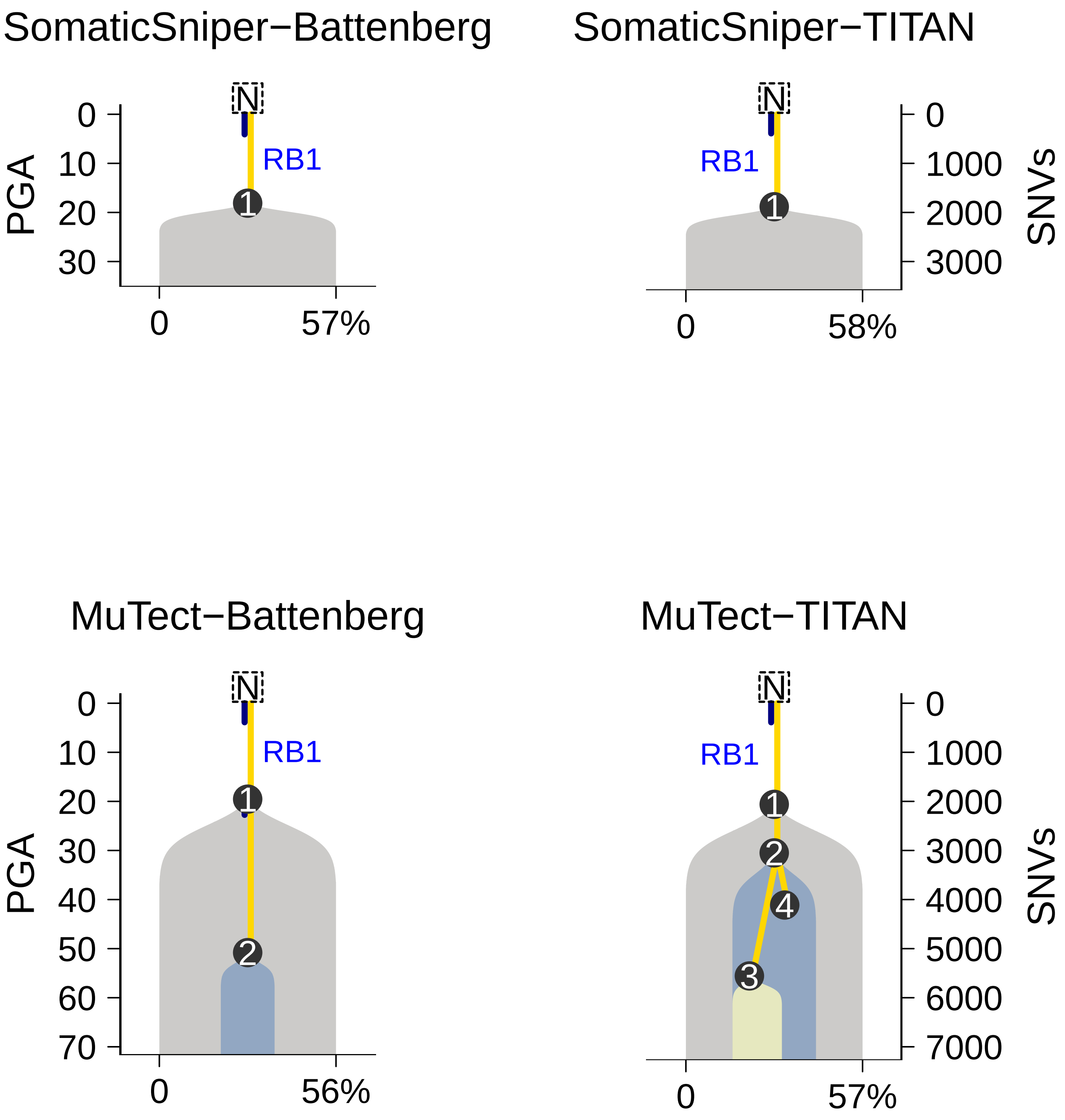
SomaticSniper−Battenberg
SomaticSniper−TITAN
MuTect−Battenberg
MuTect−TITAN
N
RB1
PGA
SNVs
0
10
20
30
40
50
60
70
1000
2000
3000
4000
5000
6000
7000
57%
58%
56%
57%
1
2
3
4

CPCG0256

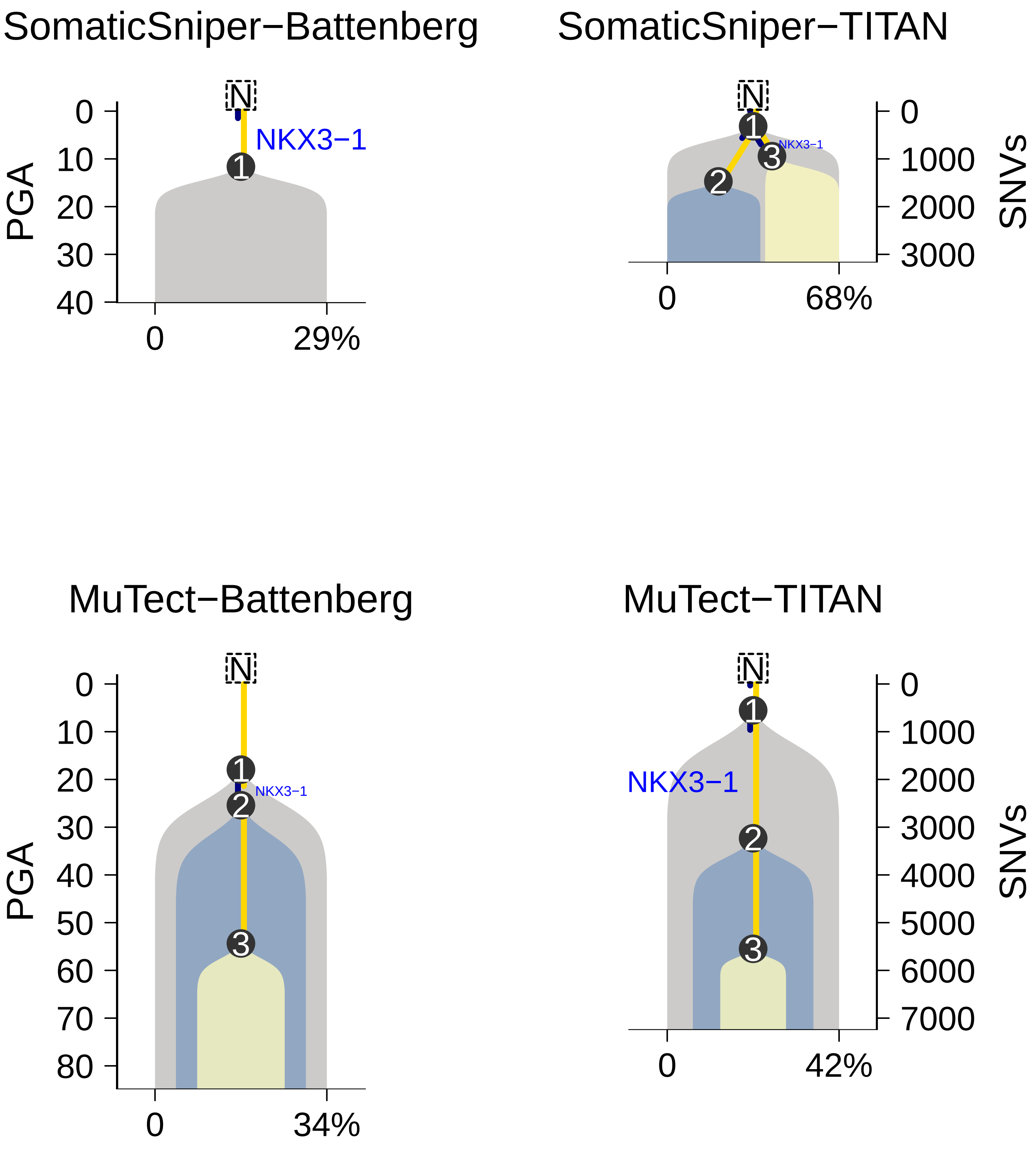

SomaticSniper−Battenberg
SomaticSniper−TITAN
MuTect−Battenberg
MuTect−TITAN
N
1
2
3
NKX3−1
PGA
SNVs
0
10
20
30
40
50
60
70
80
1000
2000
3000
4000
5000
6000
7000
29%
68%
34%
42%

CPCG0258

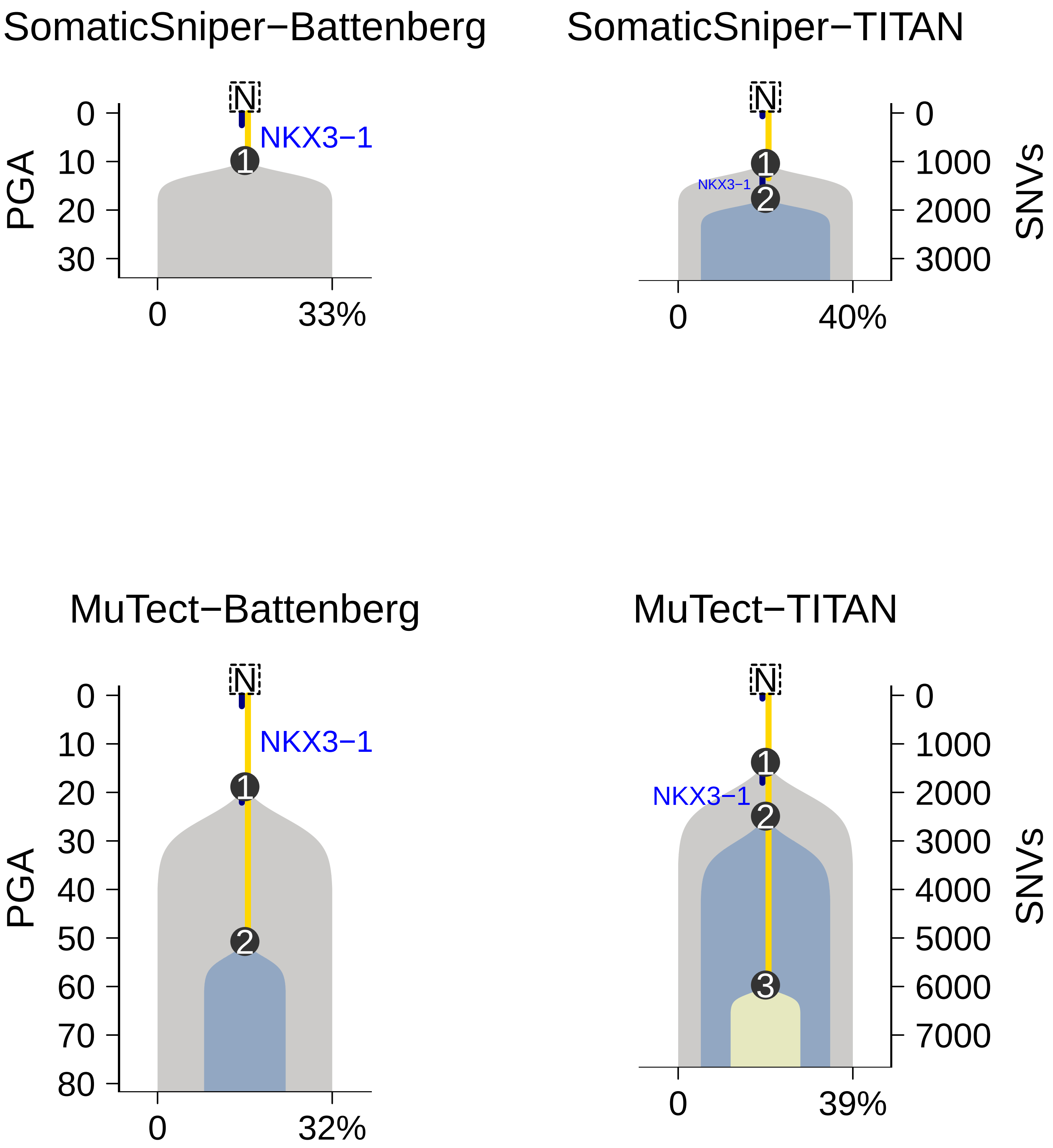
SomaticSniper−Battenberg
SomaticSniper−TITAN
MuTect−Battenberg
MuTect−TITAN
N
NKX3−1
PGA
SNVs
0
10
20
30
40
50
60
70
80
1000
2000
3000
4000
5000
6000
7000
33%
40%
32%
39%
1
2
3

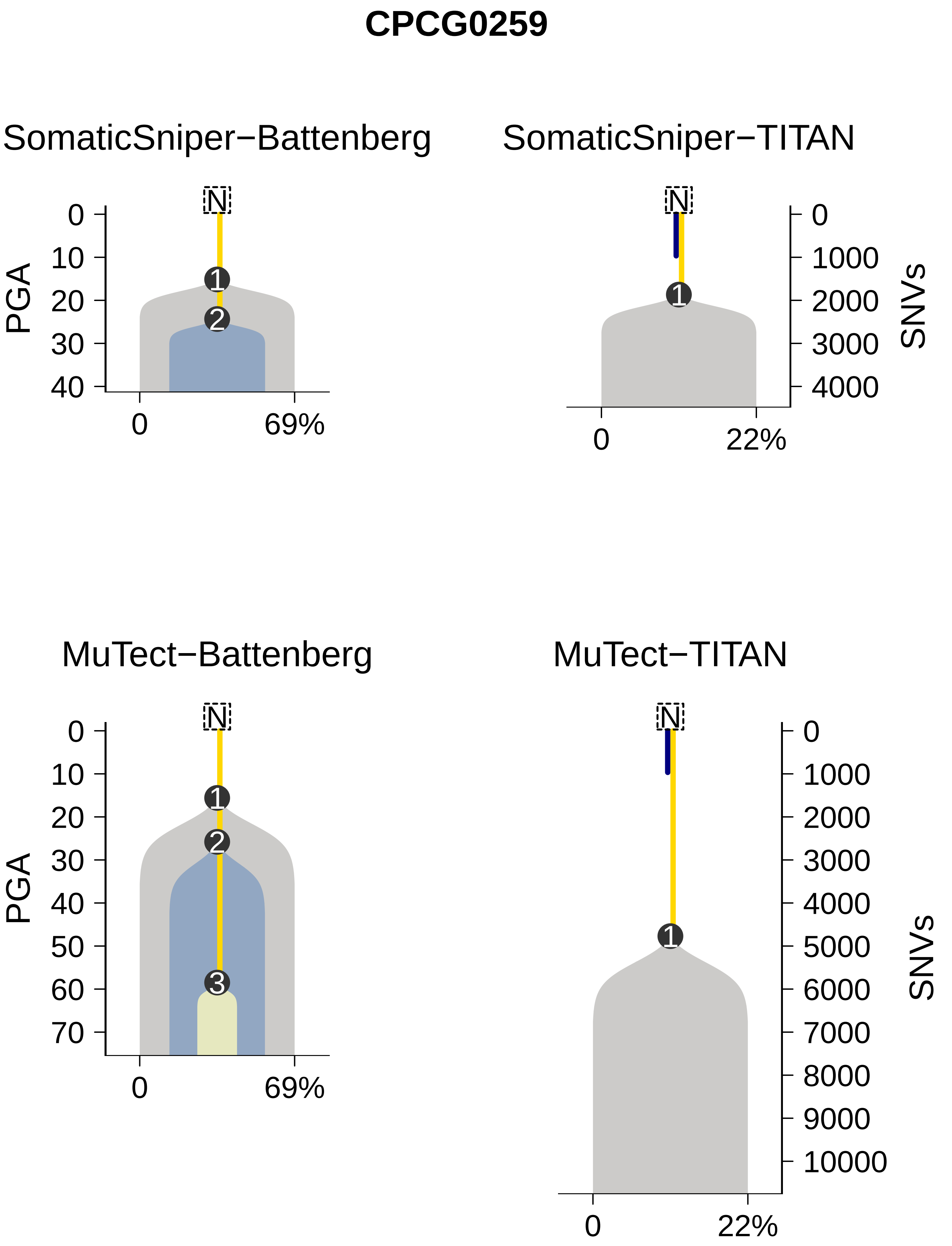
CPCG0259
SomaticSniper−Battenberg
SomaticSniper−TITAN
MuTect−Battenberg
MuTect−TITAN
N
1
2
3
PGA
SNVs
0
10
20
30
40
50
60
70
0
1000
2000
3000
4000
5000
6000
7000
8000
9000
10000
69%
22%

CPCG0260

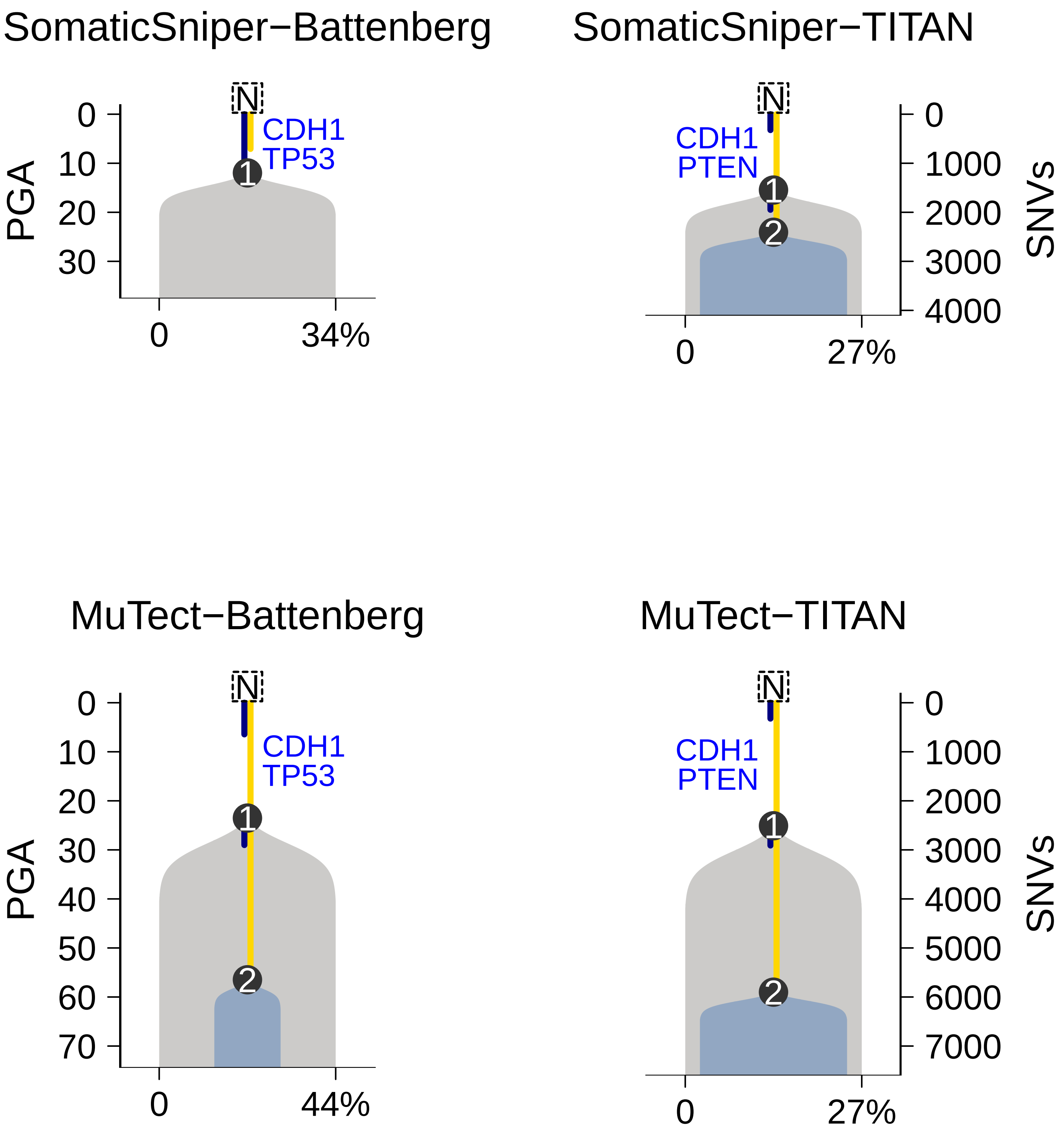
SomaticSniper−Battenberg
SomaticSniper−TITAN
MuTect−Battenberg
MuTect−TITAN
N
1
2
CDH1
TP53
CDH1
PTEN
PGA
SNVs
0
10
20
30
40
50
60
70
1000
2000
3000
4000
5000
6000
7000
34%
27%
44%

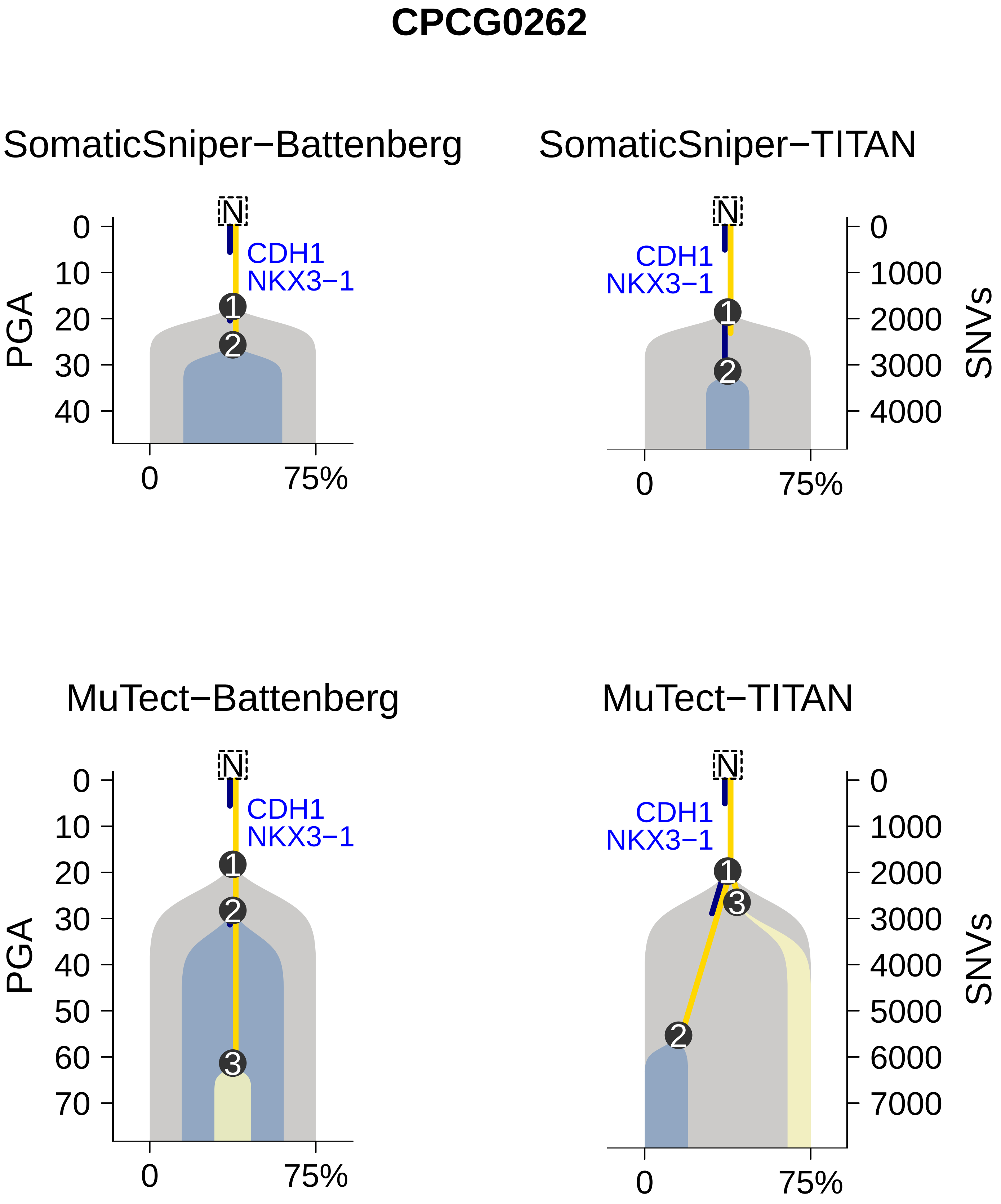
CPCG0262
SomaticSniper−Battenberg
SomaticSniper−TITAN
MuTect−Battenberg
MuTect−TITAN
N
CDH1
NKX3−1
1
2
3
PGA
0
10
20
30
40
50
60
70
SNVs
0
1000
2000
3000
4000
5000
6000
7000
0
75%

CPCG0263

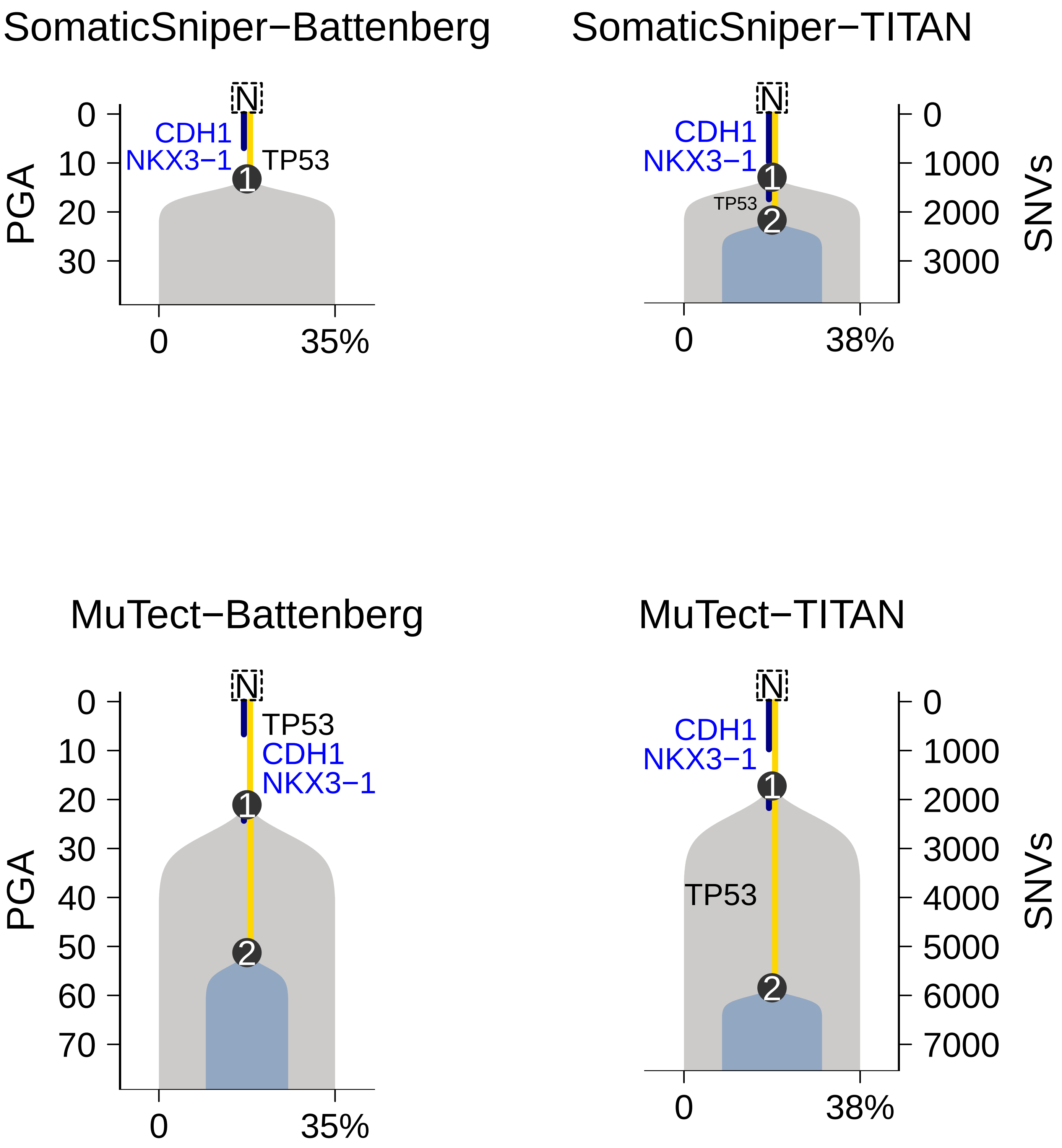

SomaticSniper−Battenberg
SomaticSniper−TITAN
MuTect−Battenberg
MuTect−TITAN
N
CDH1
NKX3−1
TP53
1
2
PGA
SNVs
0
10
20
30
40
50
60
70
1000
2000
3000
4000
5000
6000
7000
35%
38%

CPCG0265

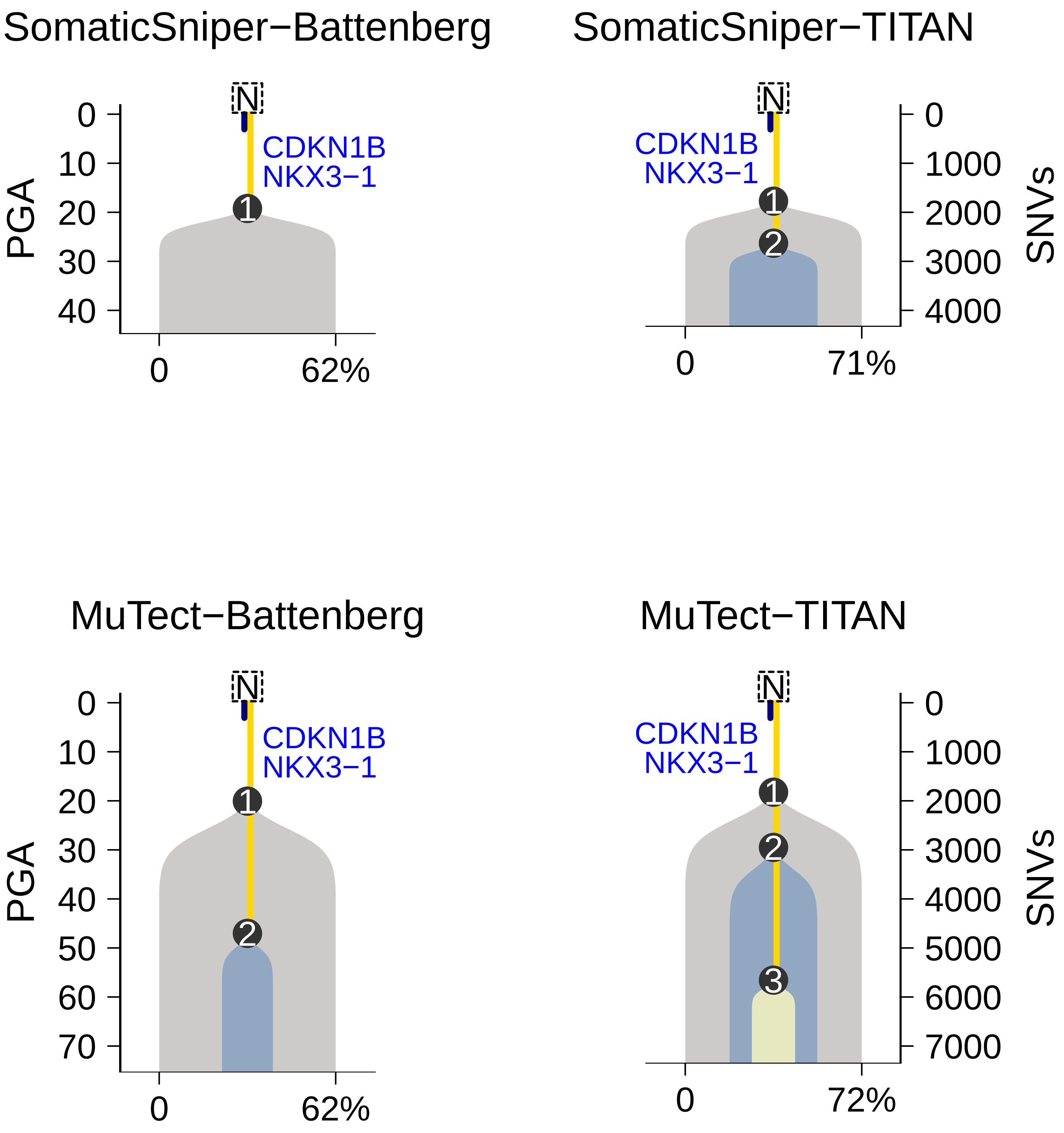
SomaticSniper−Battenberg
SomaticSniper−TITAN
MuTect−Battenberg
MuTect−TITAN
N
CDKN1B
NKX3−1
PGA
SNVs
0
10
20
30
40
50
60
70
0
1000
2000
3000
4000
5000
6000
7000
0
62%
71%
72%
1
2
3

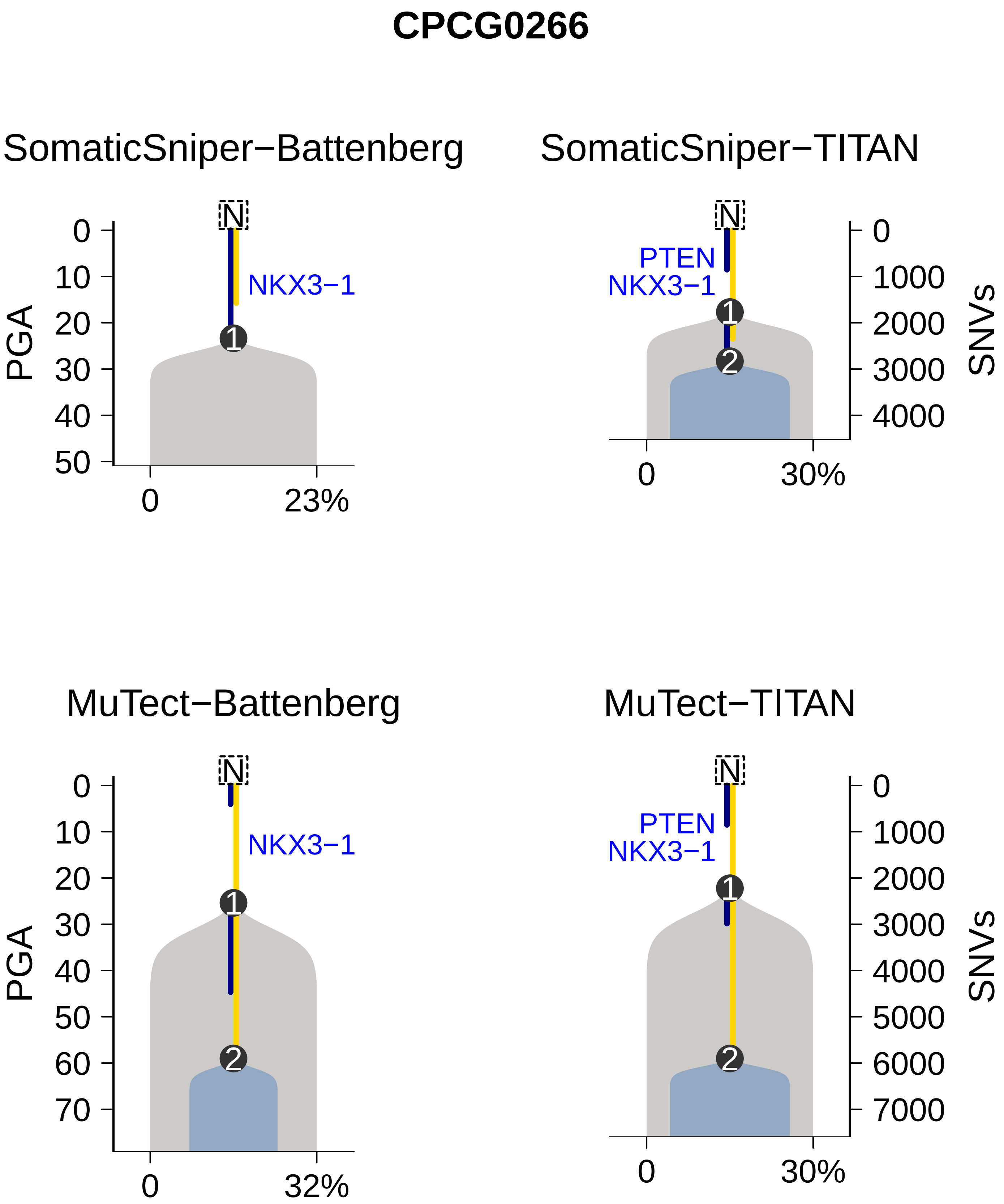
CPCG0266
SomaticSniper−Battenberg
SomaticSniper−TITAN
MuTect−Battenberg
MuTect−TITAN
N
1
2
NKX3−1
PTEN
PGA
SNVs
0
10
20
30
40
50
60
70
1000
2000
3000
4000
5000
6000
7000
23%
30%
32%

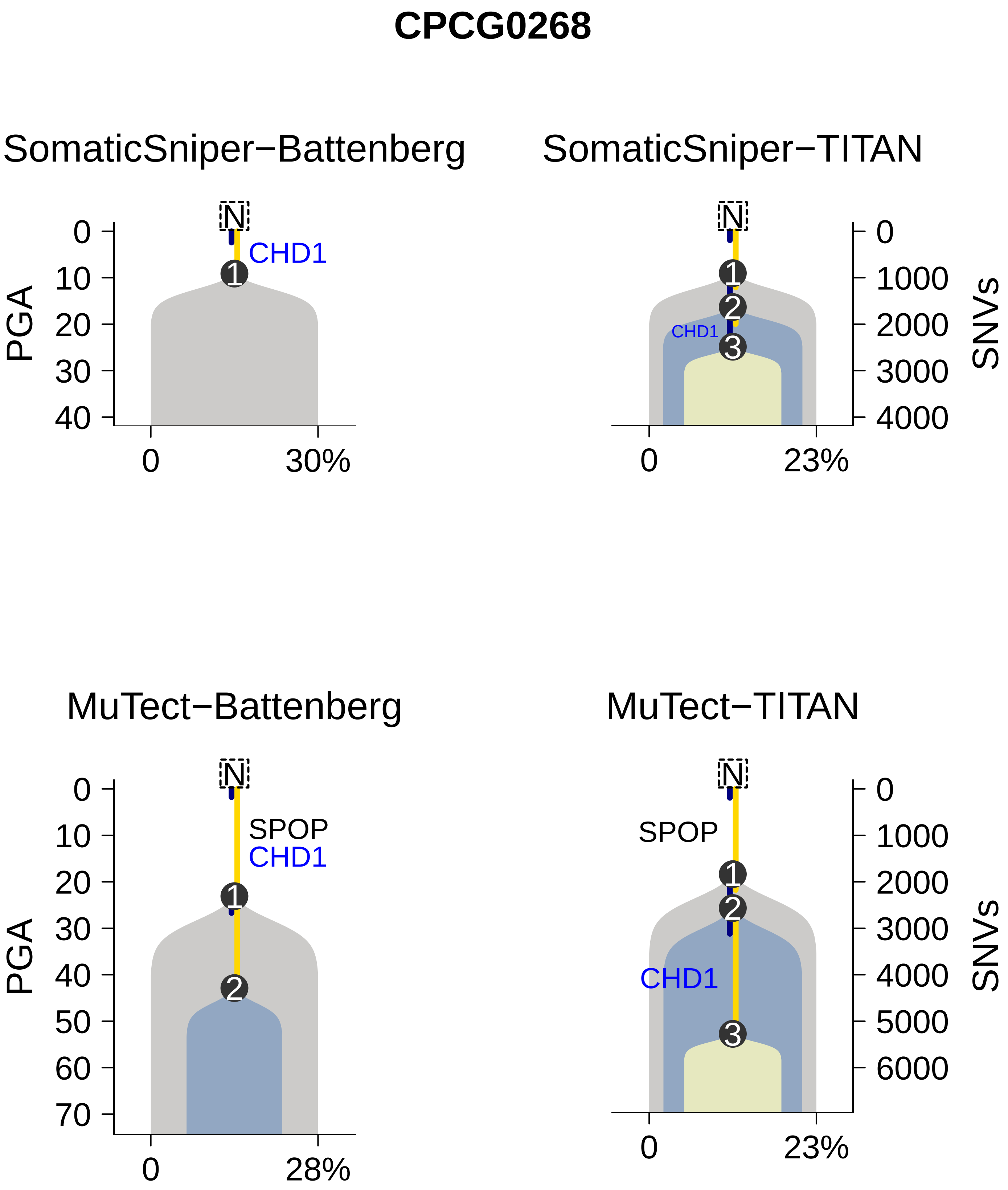
CPCG0268
SomaticSniper−Battenberg
SomaticSniper−TITAN
MuTect−Battenberg
MuTect−TITAN
N
1
2
3
CHD1
SPOP
PGA
SNVs
0
10
20
30
40
50
60
70
1000
2000
3000
4000
5000
6000
30%
23%
28%

CPCG0269

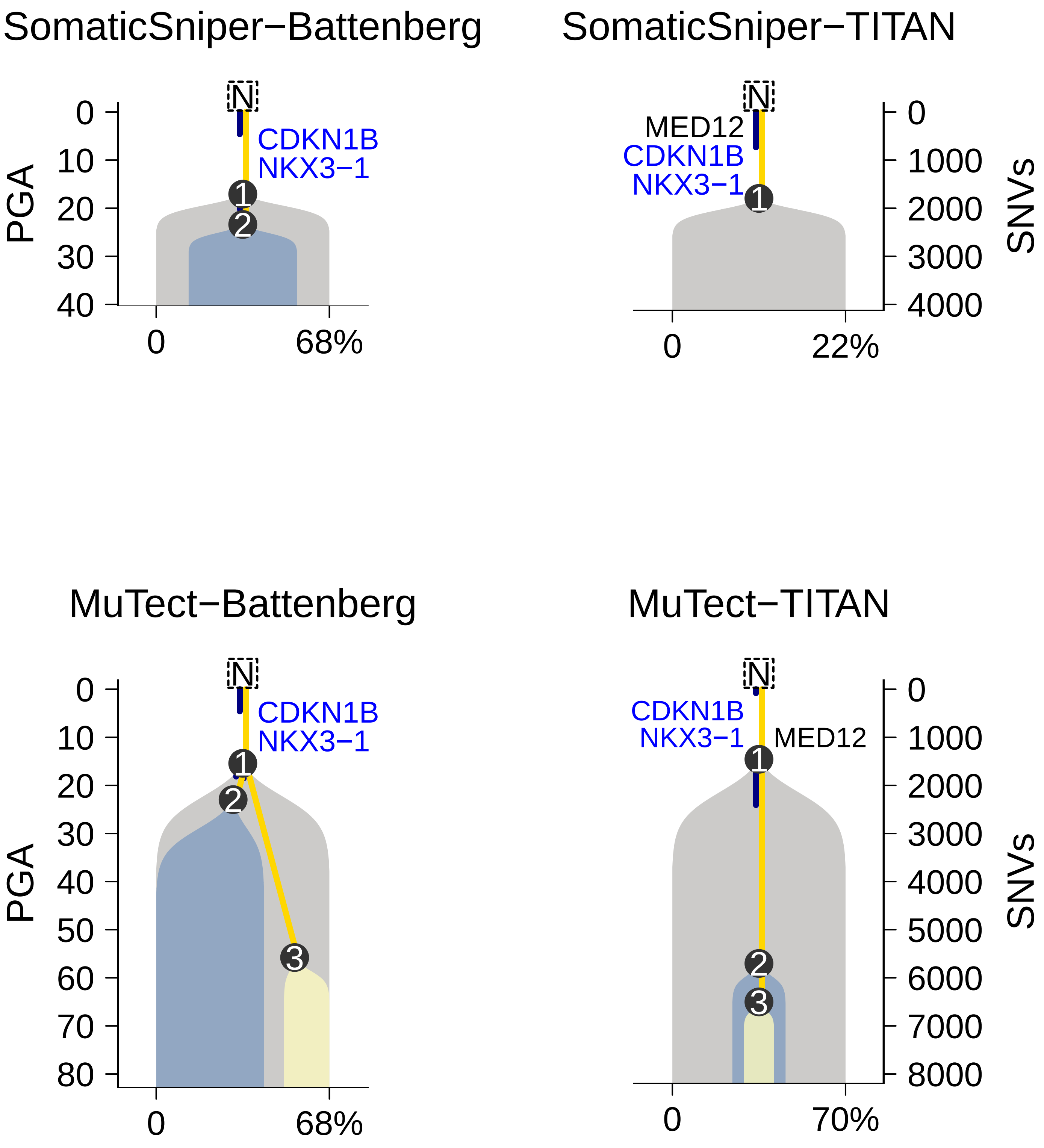

SomaticSniper−Battenberg
SomaticSniper−TITAN
N
CDKN1B
NKX3−1
MED12
PGA
SNVs
0
10
20
30
40
68%
22%
1000
2000
3000
4000
1
2
MuTect−Battenberg
MuTect−TITAN
50
60
70
80
70%
5000
6000
7000
8000
3

CPCG0274

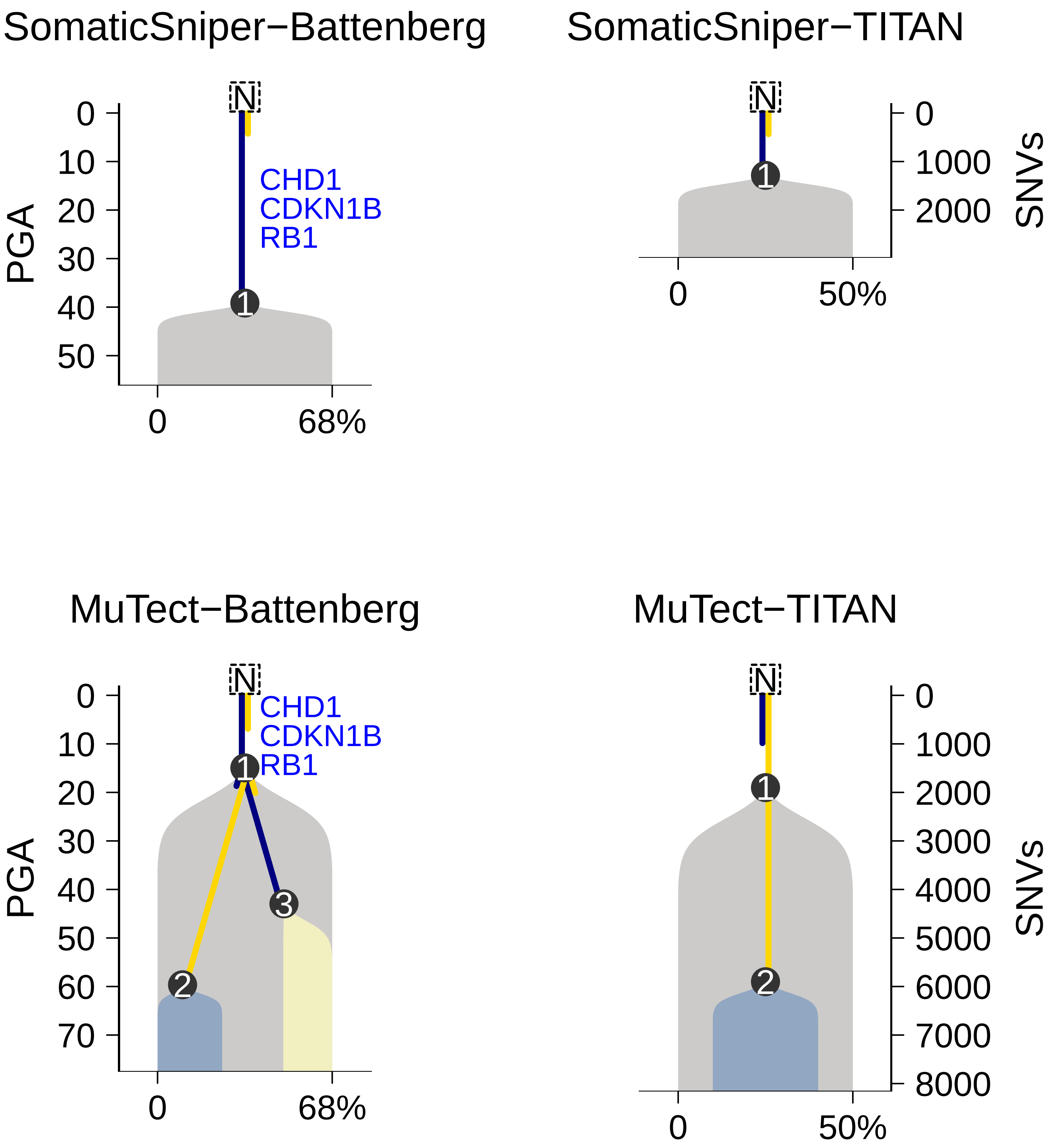
SomaticSniper−Battenberg
SomaticSniper−TITAN
MuTect−Battenberg
MuTect−TITAN
N
PGA
SNVs
CHD1
CDKN1B
RB1
0
10
20
30
40
50
60
70
0
1000
2000
3000
4000
5000
6000
7000
8000
0
68%
50%
1
2
3

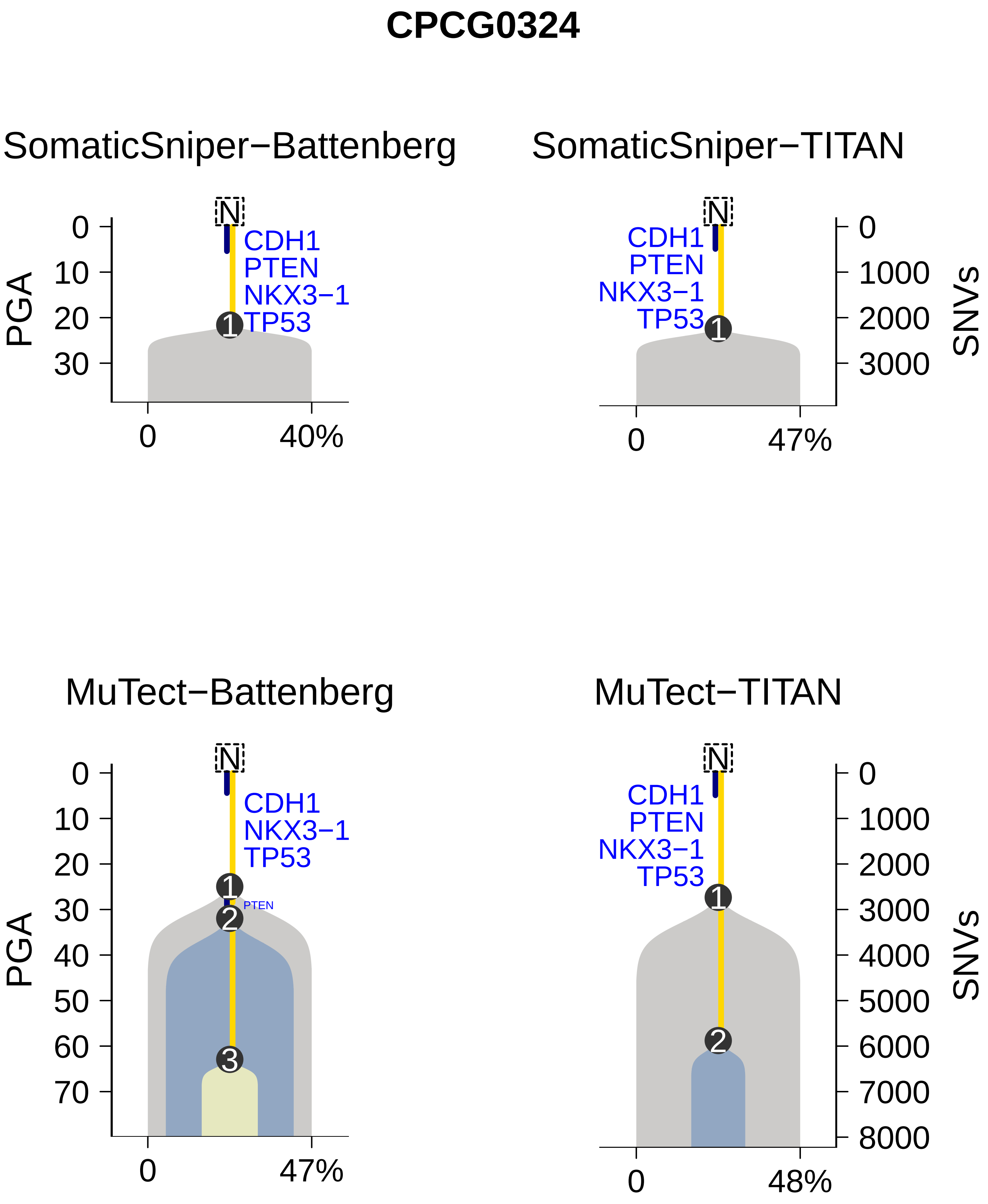
CPCG0324
SomaticSniper−Battenberg
SomaticSniper−TITAN
MuTect−Battenberg
MuTect−TITAN
N
CDH1
PTEN
NKX3−1
TP53
PTEN
PGA
SNVs
0
10
20
30
40
50
60
70
1000
2000
3000
4000
5000
6000
7000
8000
40%
47%
48%
1
2
3

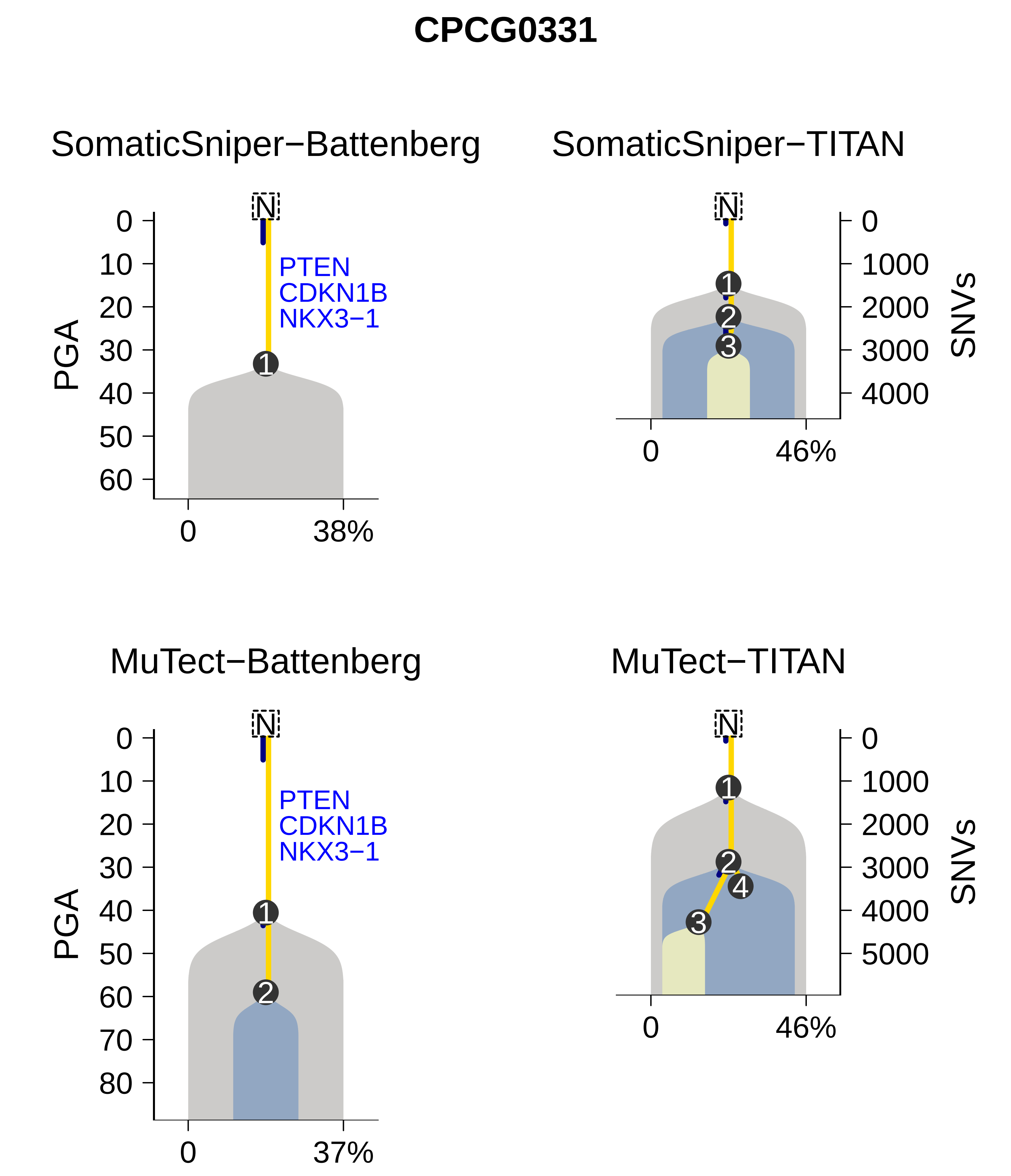
CPCG0331
SomaticSniper−Battenberg
N
PTEN
CDKN1B
NKX3−1
1
PGA
0
10
20
30
40
50
60
0
38%
SomaticSniper−TITAN
N
1
2
3
0
1000
2000
3000
4000
SNVs
0
46%
MuTect−Battenberg
N
PTEN
CDKN1B
NKX3−1
1
2
PGA
0
10
20
30
40
50
60
70
80
0
37%
MuTect−TITAN
N
1
2
3
4
0
1000
2000
3000
4000
5000
SNVs
0
46%

CPCG0334

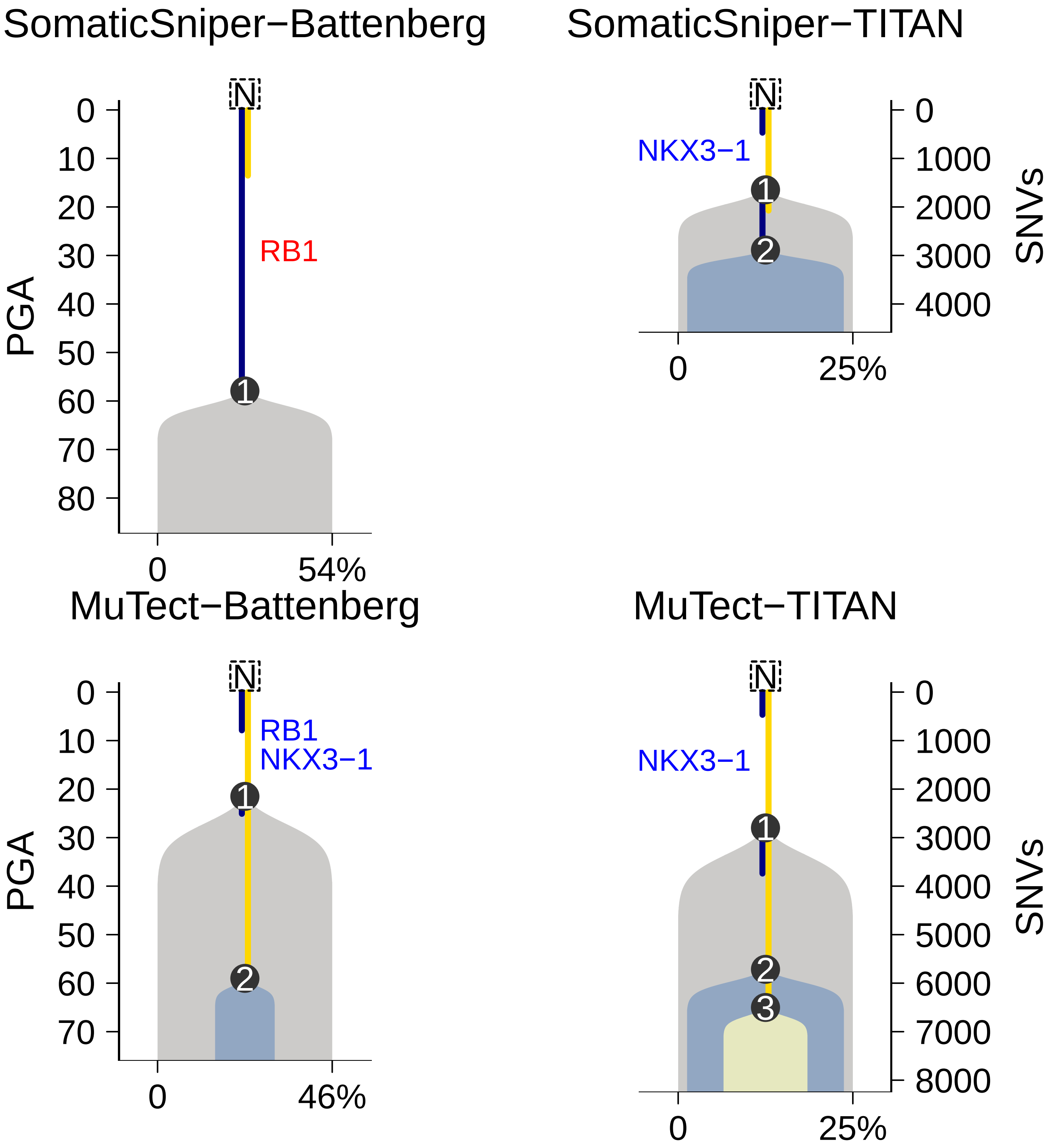
SomaticSniper−Battenberg
SomaticSniper−TITAN
MuTect−Battenberg
MuTect−TITAN
PGA
SNVs
N
1
2
3
RB1
NKX3−1
0
10
20
30
40
50
60
70
80
54%
46%
25%
1000
2000
3000
4000
5000
6000
7000
8000

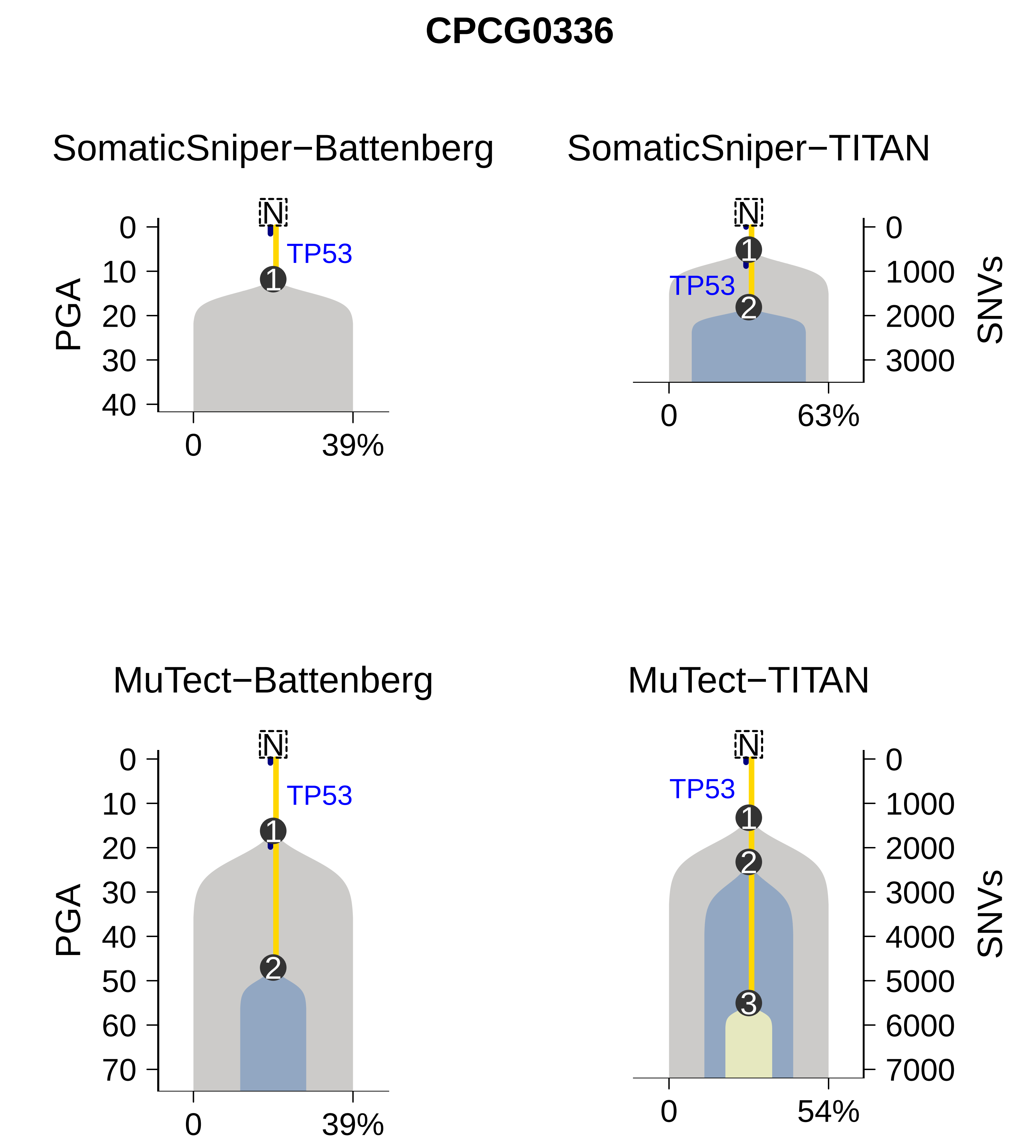
CPCG0336
SomaticSniper−Battenberg
SomaticSniper−TITAN
MuTect−Battenberg
MuTect−TITAN
N
TP53
1
2
3
PGA
SNVs
0
10
20
30
40
50
60
70
1000
2000
3000
4000
5000
6000
7000
39%
63%
54%

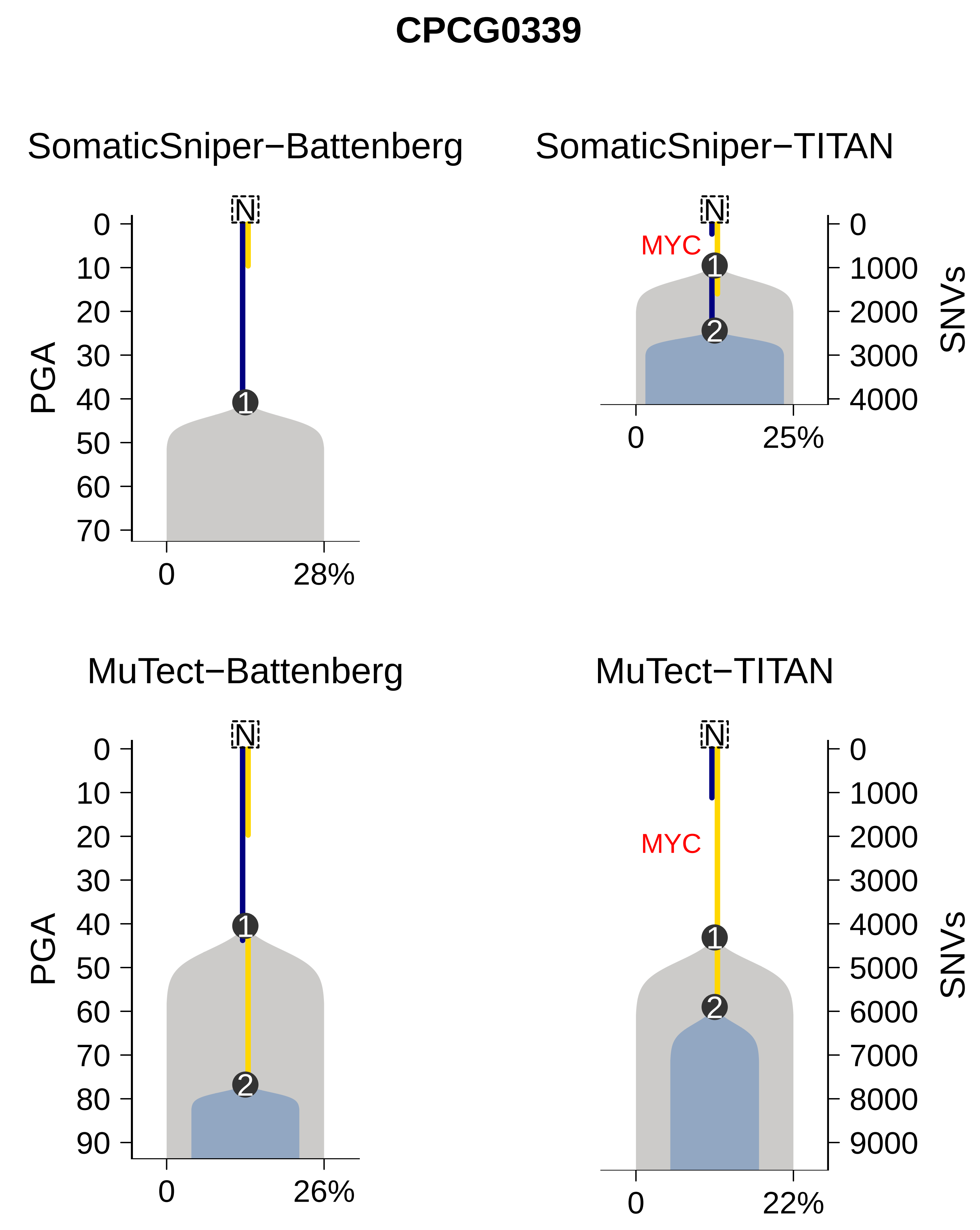
CPCG0339
SomaticSniper−Battenberg
SomaticSniper−TITAN
MuTect−Battenberg
MuTect−TITAN
PGA
SNVs
N
1
2
MYC
0
28%
25%
26%
22%
0
10
20
30
40
50
60
70
80
90
0
1000
2000
3000
4000
5000
6000
7000
8000
9000

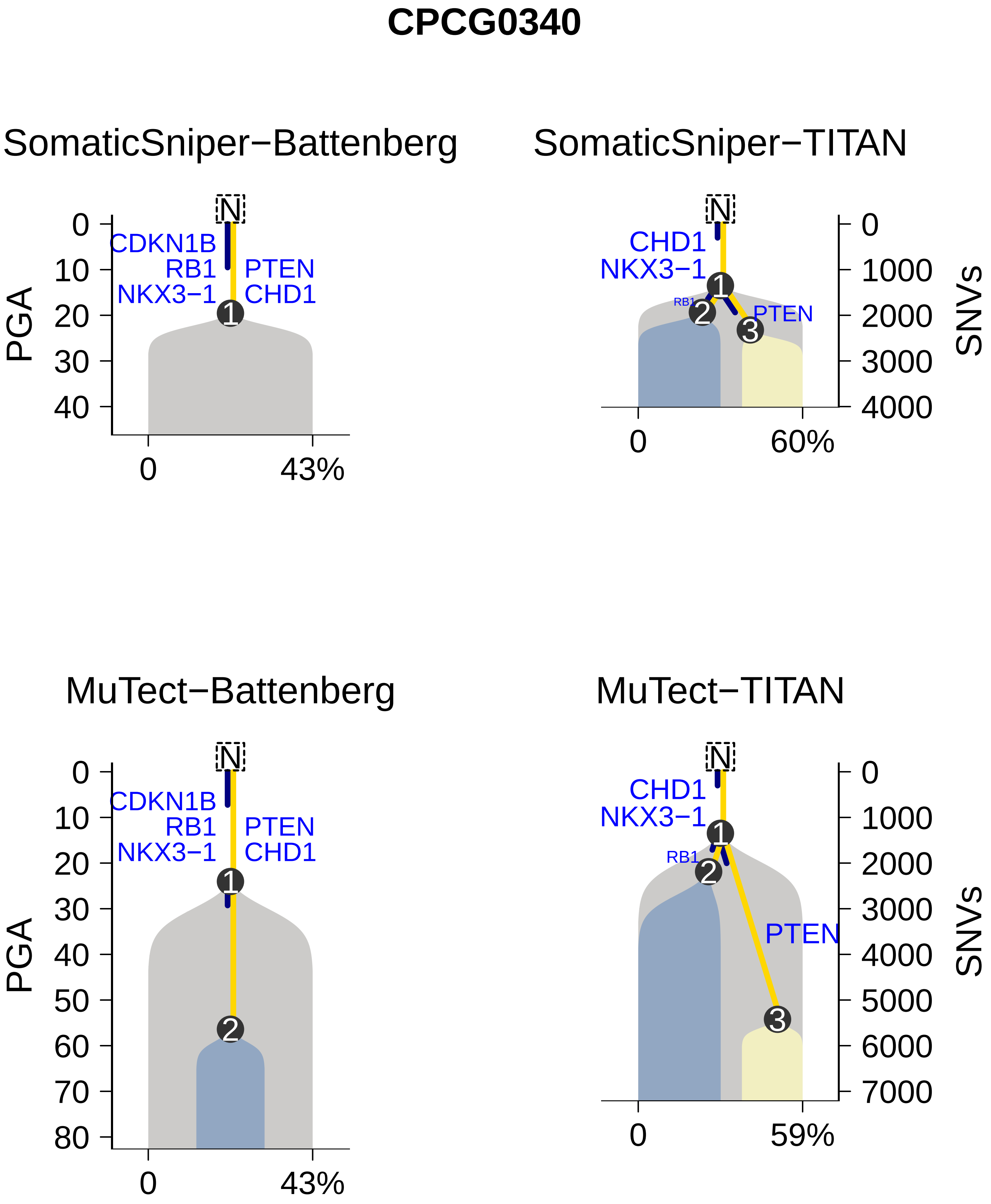
CPCG0340
SomaticSniper−Battenberg
SomaticSniper−TITAN
MuTect−Battenberg
MuTect−TITAN
N
CDKN1B
RB1
NKX3−1
PTEN
CHD1
PGA
SNVs
0
43%
60%
59%

CPCG0341

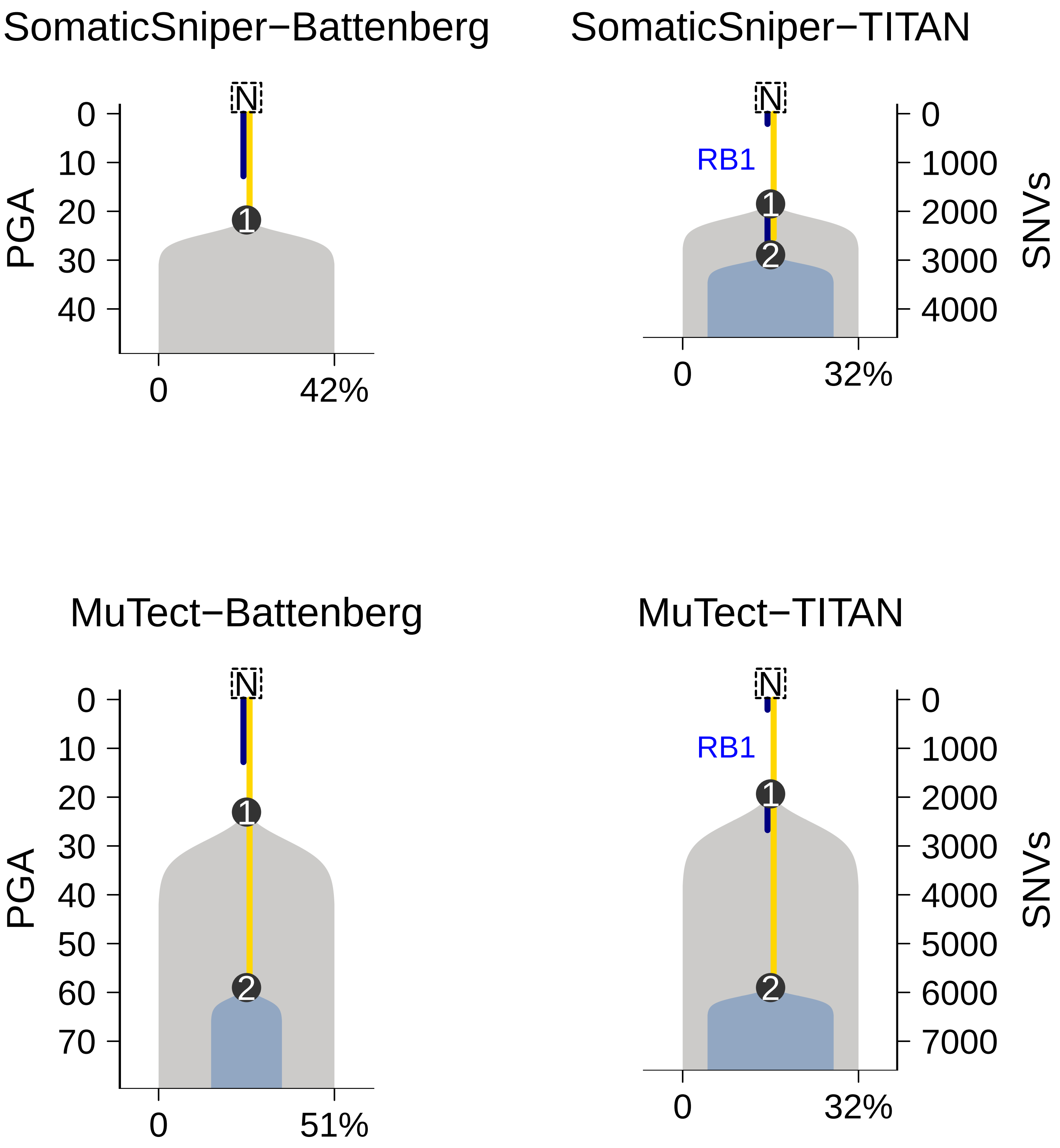

SomaticSniper−Battenberg
SomaticSniper−TITAN
MuTect−Battenberg
MuTect−TITAN
N
1
2
RB1
PGA
SNVs
0
10
20
30
40
50
60
70
1000
2000
3000
4000
5000
6000
7000
42%
32%
51%

CPCG0342

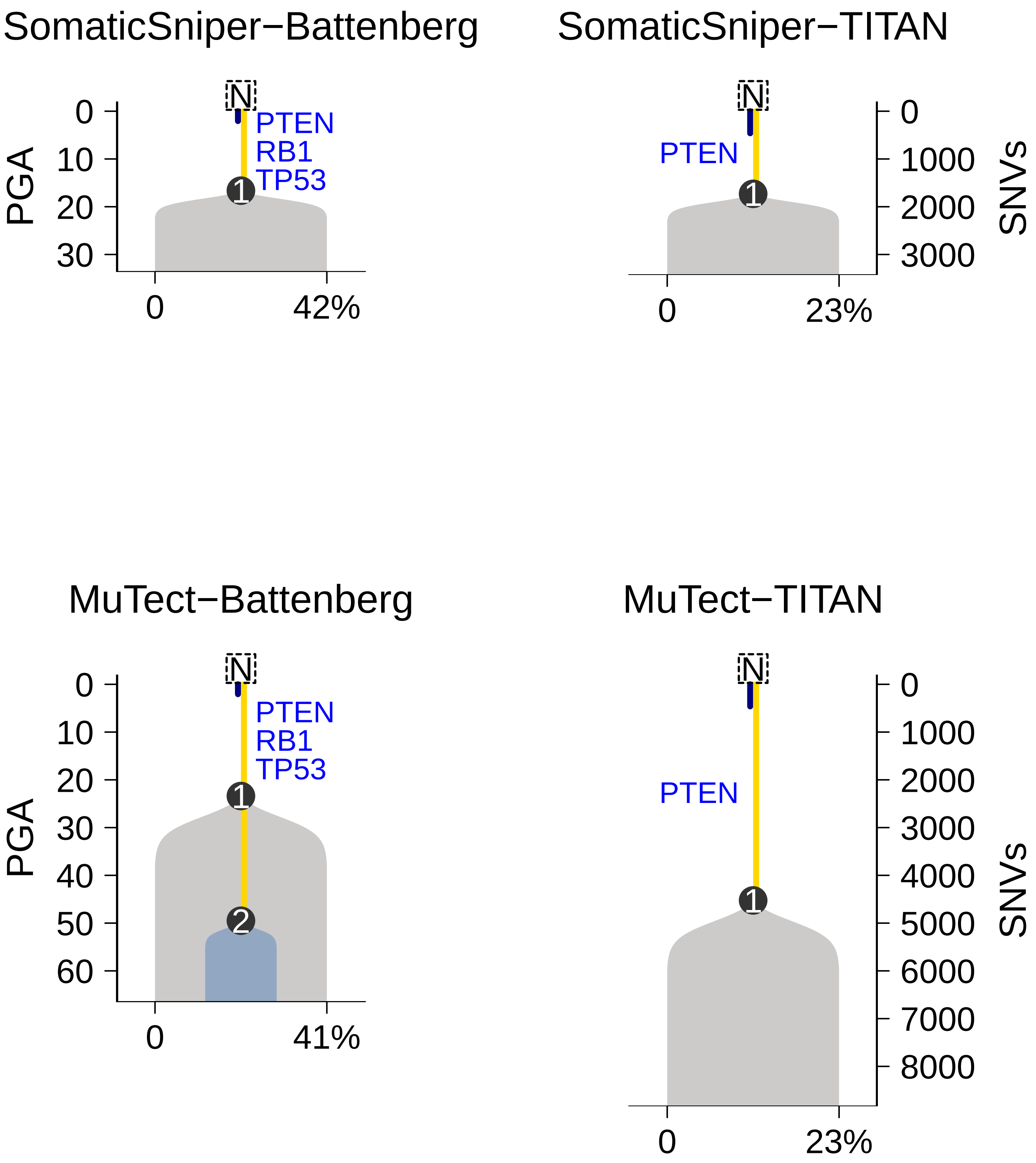
SomaticSniper−Battenberg
SomaticSniper−TITAN
MuTect−Battenberg
MuTect−TITAN
N
PTEN
RB1
TP53
1
2
PGA
SNVs
0
10
20
30
40
50
60
1000
2000
3000
4000
5000
6000
7000
8000
42%
41%
23%

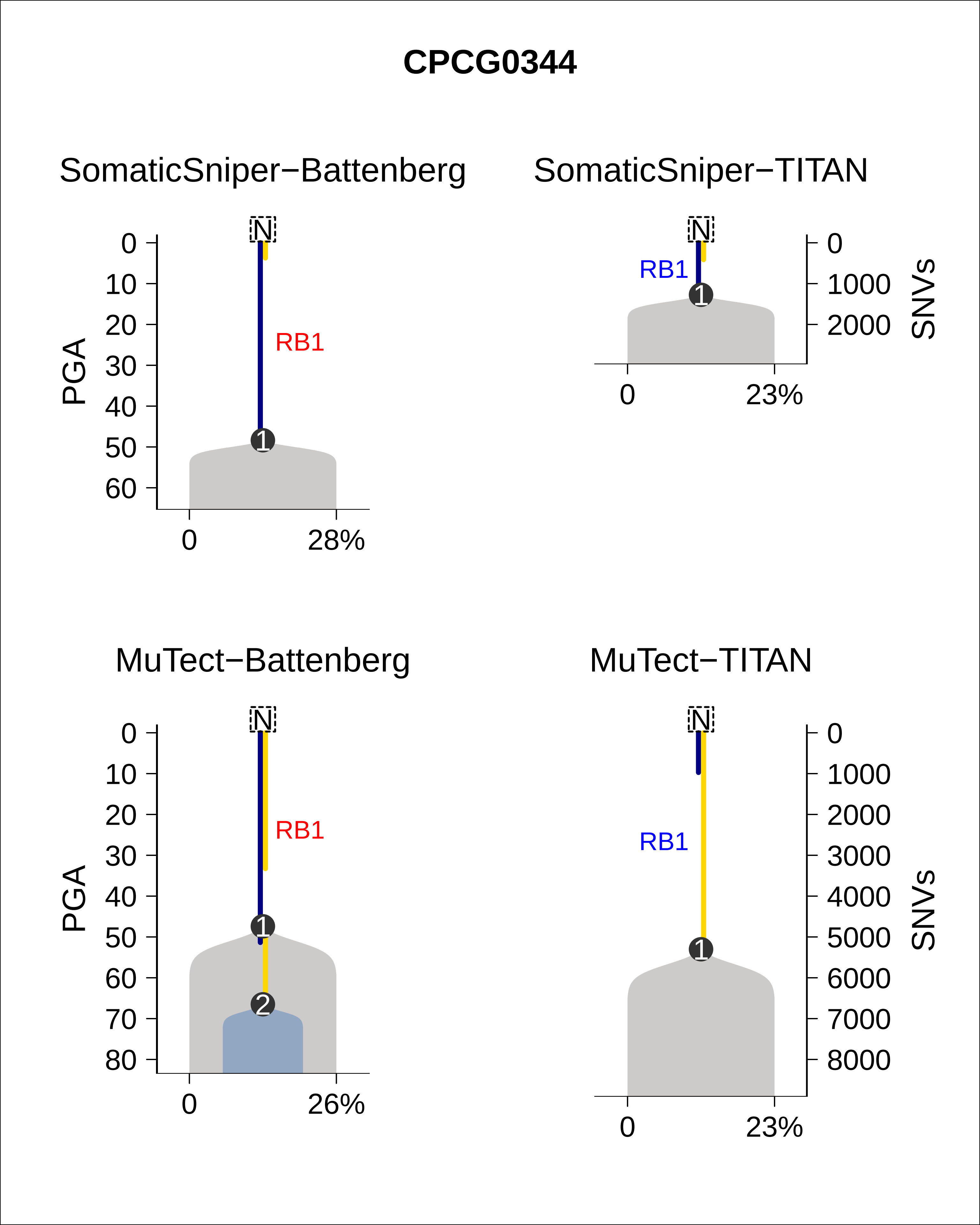
CPCG0344
SomaticSniper−Battenberg
SomaticSniper−TITAN
MuTect−Battenberg
MuTect−TITAN
N
1
2
RB1
PGA
SNVs
0
10
20
30
40
50
60
70
80
1000
2000
3000
4000
5000
6000
7000
8000
28%
26%
23%

CPCG0345

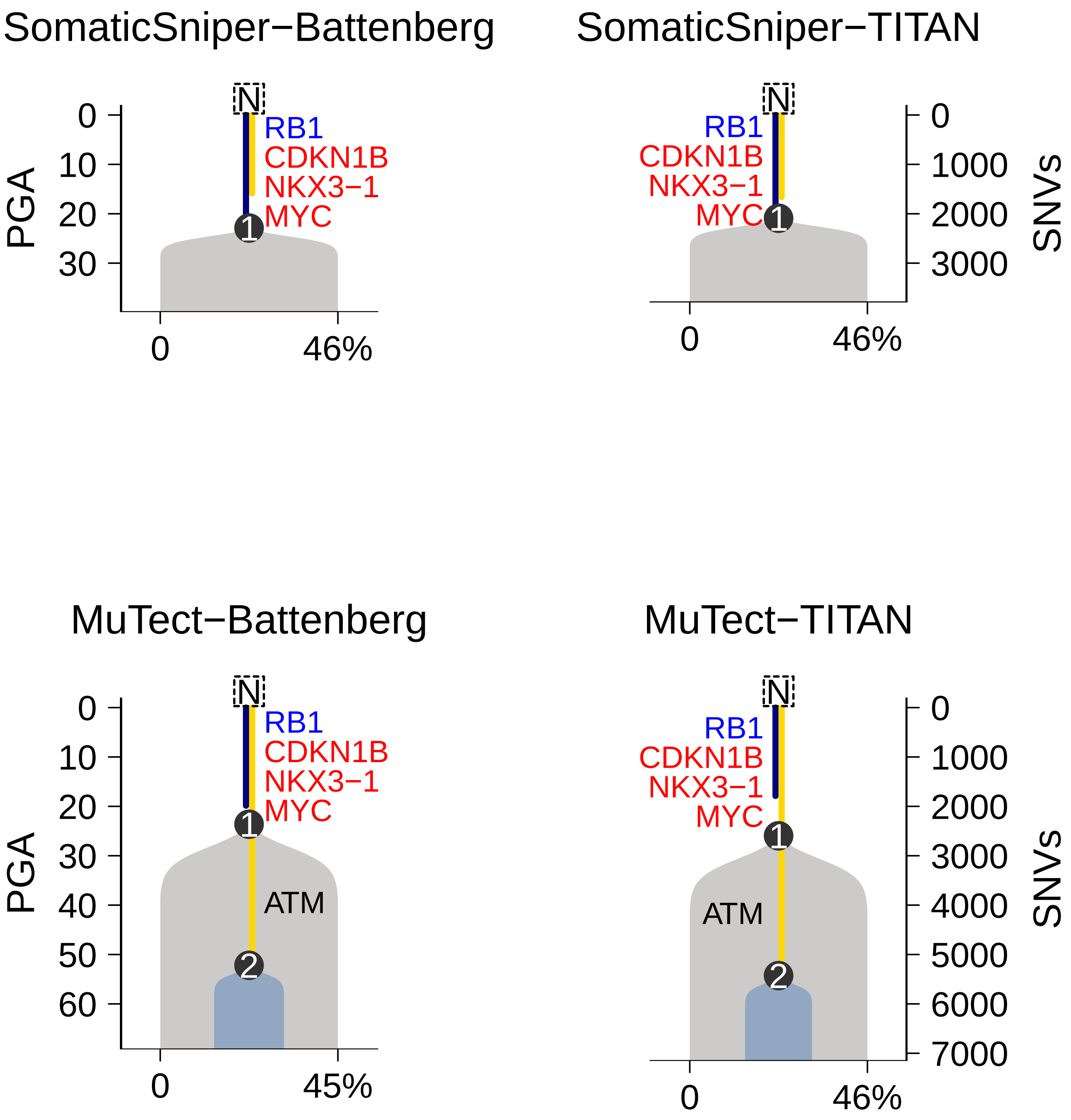
SomaticSniper−Battenberg
SomaticSniper−TITAN
MuTect−Battenberg
MuTect−TITAN
N
RB1
CDKN1B
NKX3−1
MYC
ATM
1
2
PGA
SNVs
0
10
20
30
40
50
60
1000
2000
3000
4000
5000
6000
7000
46%
45%

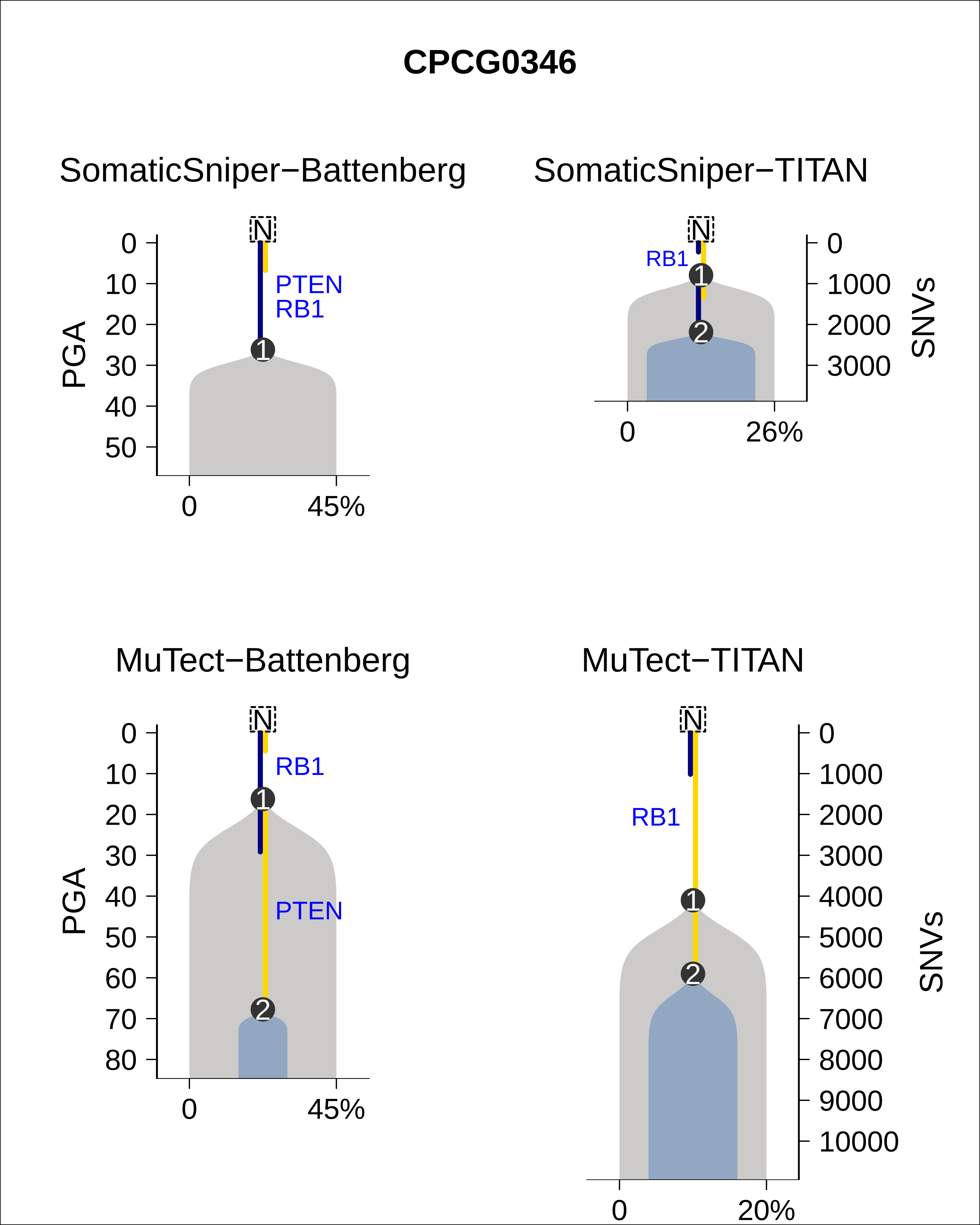
CPCG0346
SomaticSniper−Battenberg
SomaticSniper−TITAN
MuTect−Battenberg
MuTect−TITAN
PGA
SNVs
N
1
2
PTEN
RB1
0
10
20
30
40
50
60
70
80
1000
2000
3000
4000
5000
6000
7000
8000
9000
10000
45%
26%
20%

CPCG0348

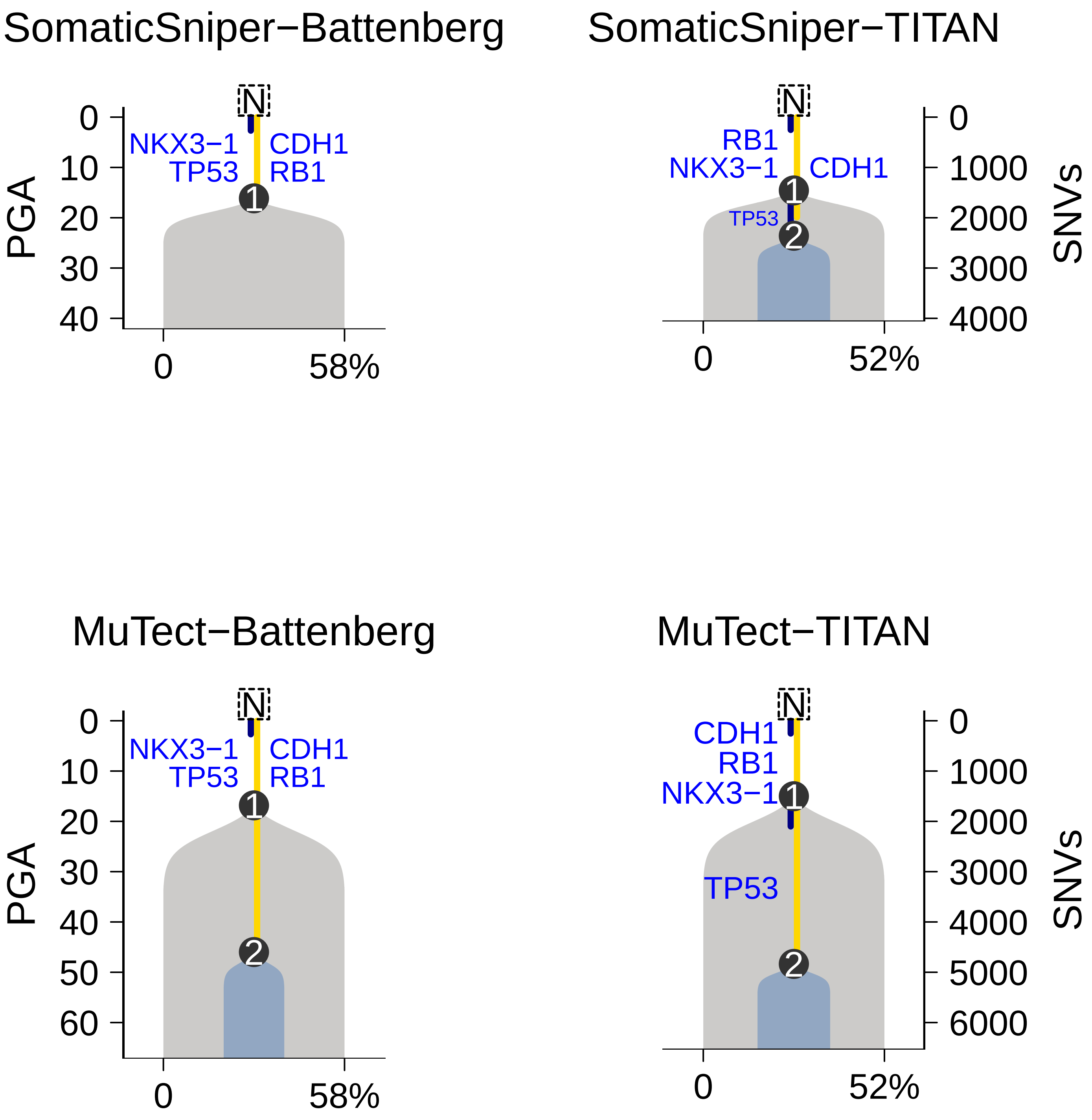

SomaticSniper−Battenberg
SomaticSniper−TITAN
MuTect−Battenberg
MuTect−TITAN
N
NKX3−1
TP53
CDH1
RB1
1
2
PGA
SNVs
0
10
20
30
40
50
60
1000
2000
3000
4000
5000
6000
58%
52%

CPCG0350

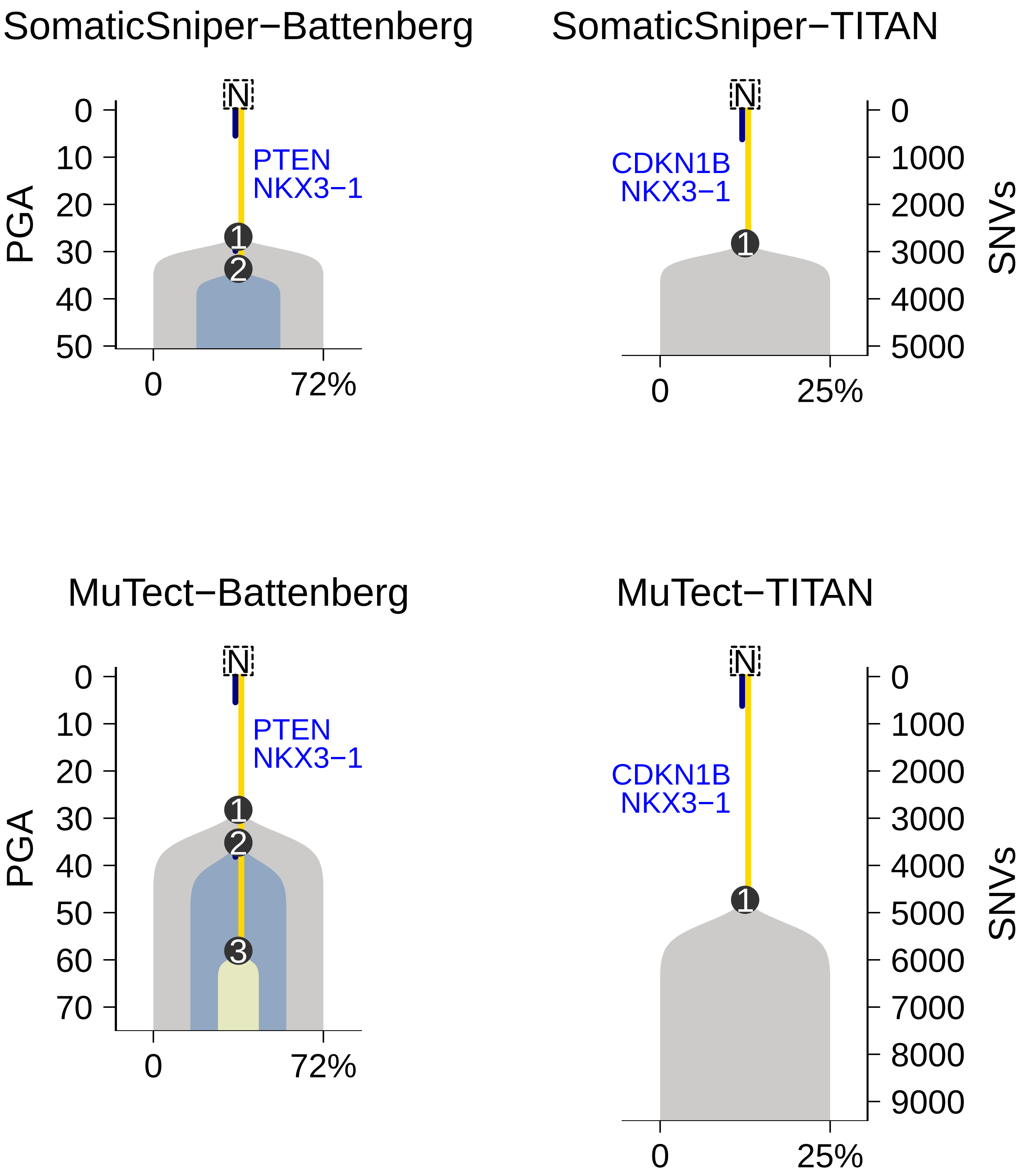
SomaticSniper−Battenberg
SomaticSniper−TITAN
N
PTEN
NKX3−1
CDKN1B
NKX3−1
PGA
SNVs
0
10
20
30
40
50
0
1000
2000
3000
4000
5000
1
2
0
72%
0
25%
MuTect−Battenberg
MuTect−TITAN
PTEN
NKX3−1
CDKN1B
NKX3−1
PGA
SNVs
0
10
20
30
40
50
60
70
0
1000
2000
3000
4000
5000
6000
7000
8000
9000
1
2
3
0
72%
0
25%

CPCG0352

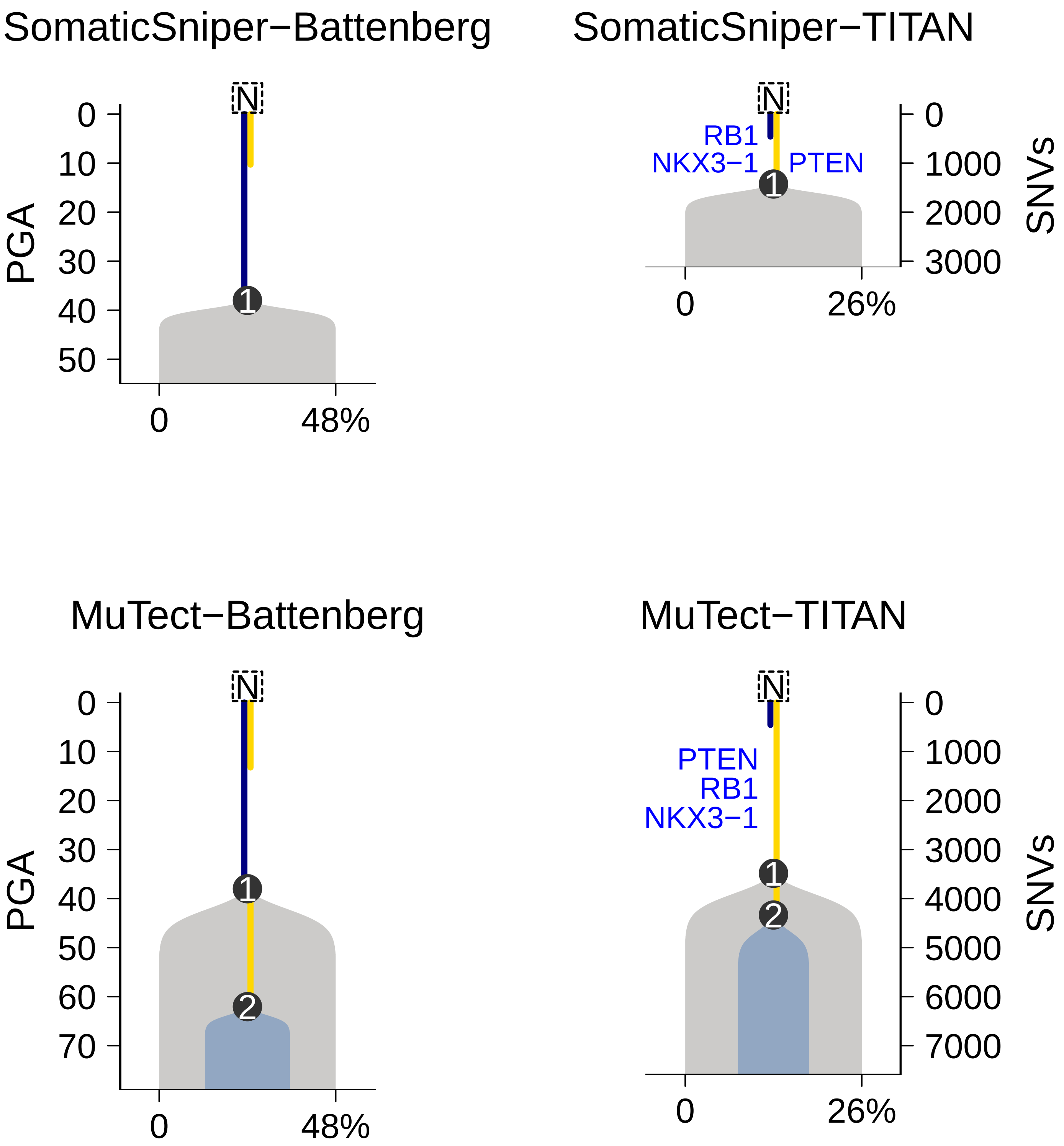

SomaticSniper−Battenberg
SomaticSniper−TITAN
MuTect−Battenberg
MuTect−TITAN
N
1
2
PGA
SNVs
0
10
20
30
40
50
60
70
48%
26%
1000
2000
3000
4000
5000
6000
7000
RB1
NKX3−1
PTEN

CPCG0353

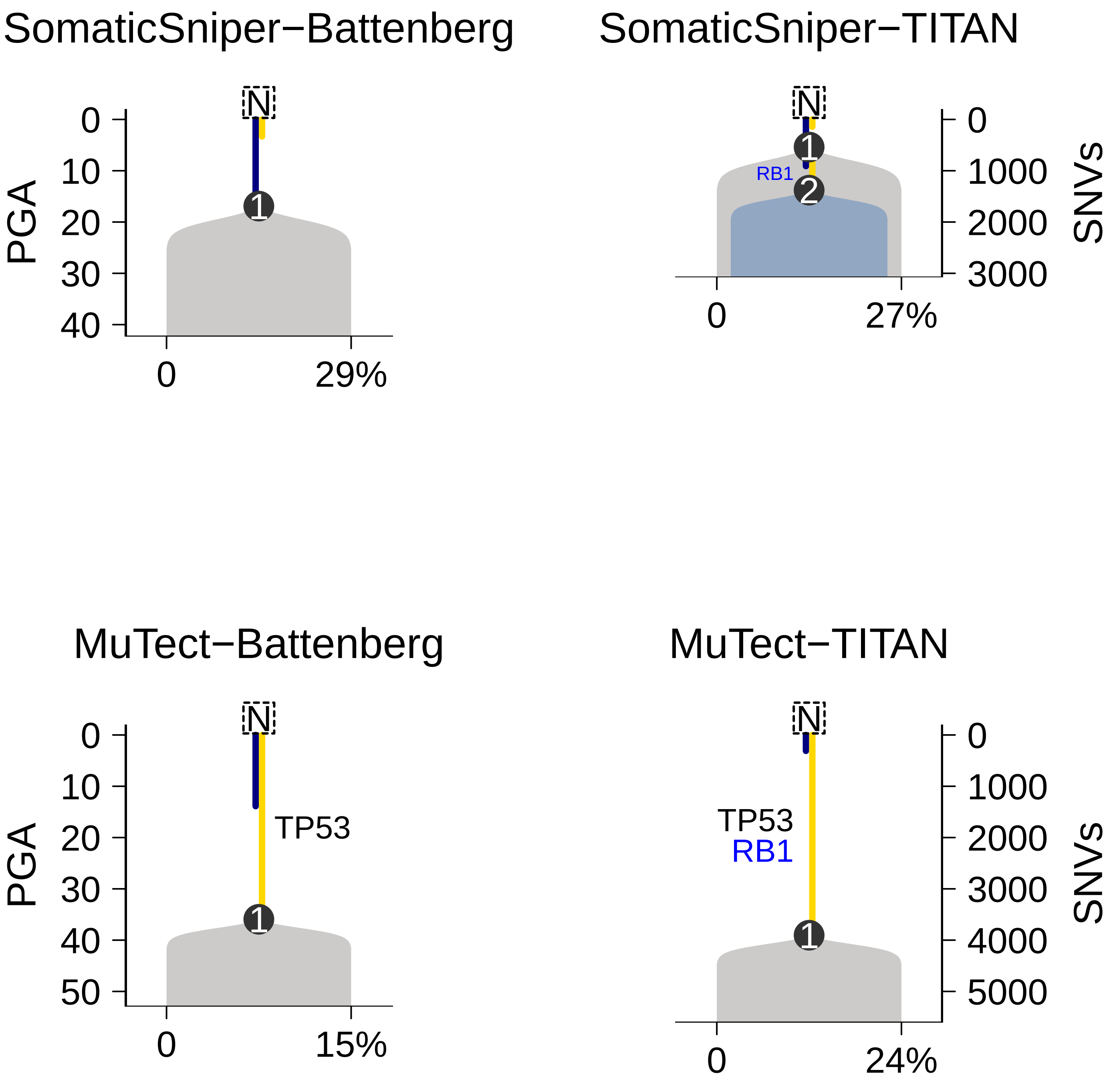

SomaticSniper−Battenberg
SomaticSniper−TITAN
MuTect−Battenberg
MuTect−TITAN
N
1
2
RB1
TP53
PGA
SNVs
0
10
20
30
40
50
29%
27%
15%
24%
1000
2000
3000
4000
5000

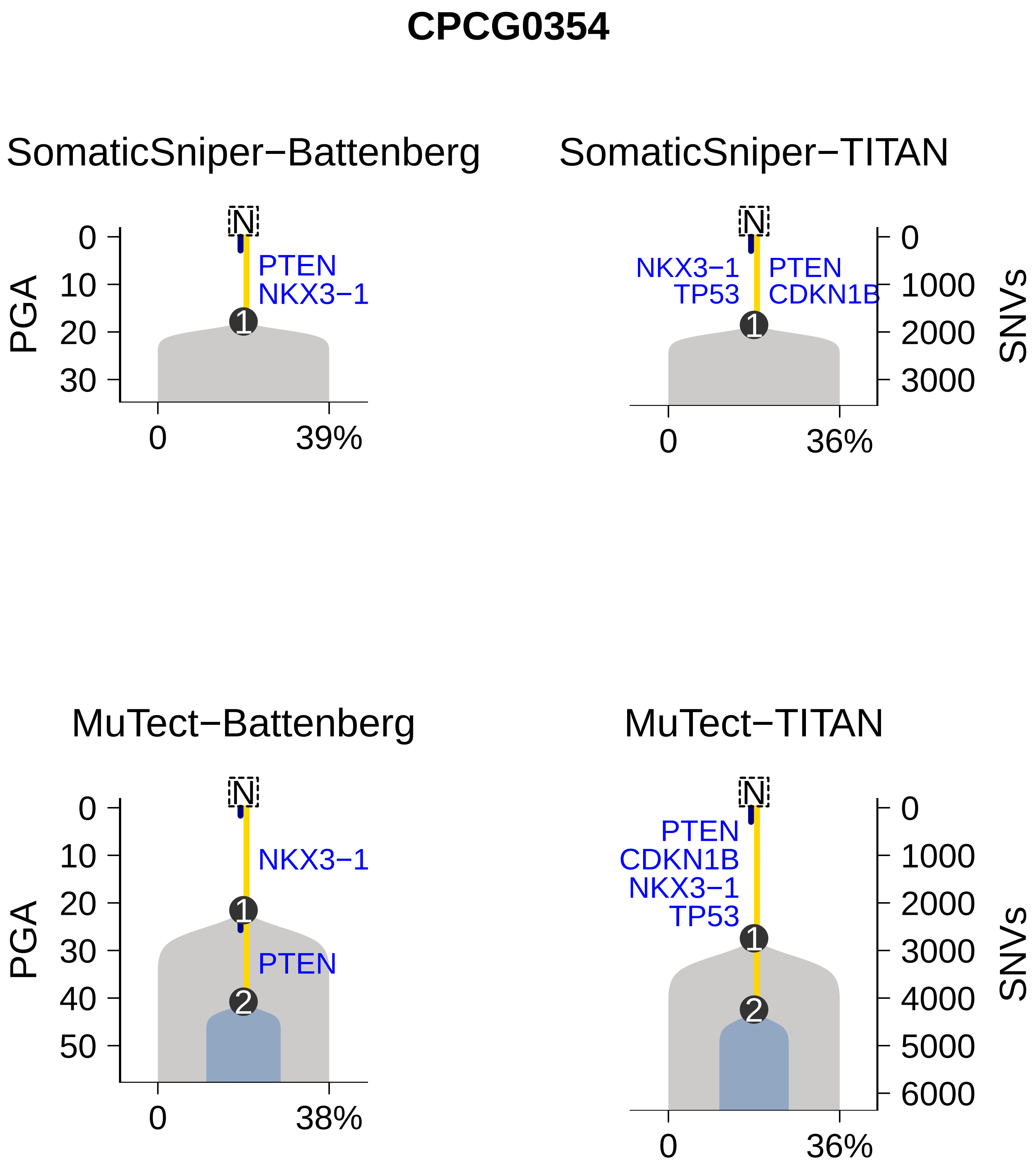
CPCG0354
SomaticSniper−Battenberg
SomaticSniper−TITAN
MuTect−Battenberg
MuTect−TITAN
PGA
SNVs
N
1
2
PTEN
NKX3−1
TP53
CDKN1B
0
10
20
30
40
50
1000
2000
3000
4000
5000
6000
39%
36%
38%

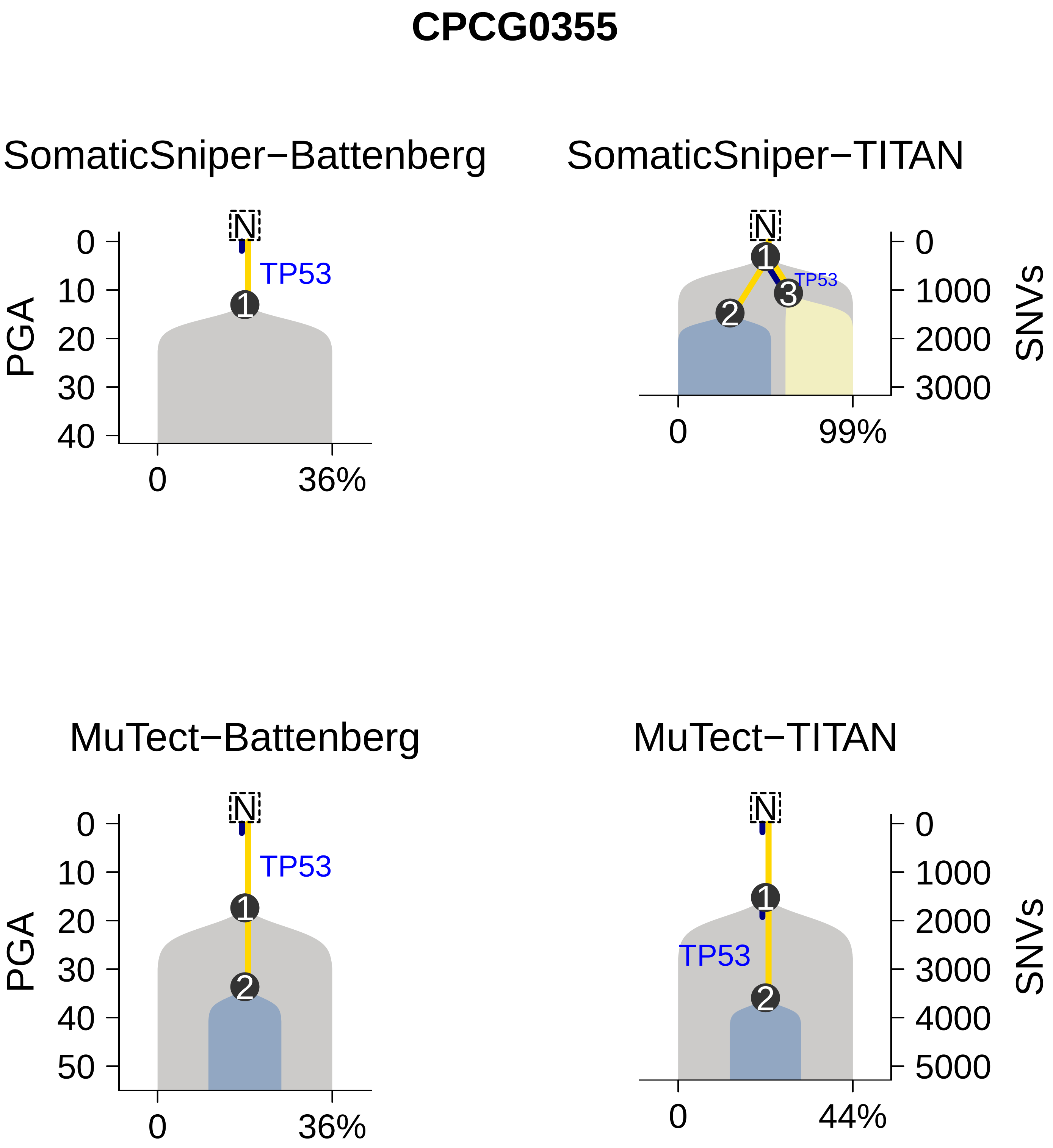
CPCG0355
SomaticSniper−Battenberg
SomaticSniper−TITAN
MuTect−Battenberg
MuTect−TITAN
N
1
2
3
TP53
PGA
SNVs
0
10
20
30
40
50
0
1000
2000
3000
4000
5000
36%
99%
44%

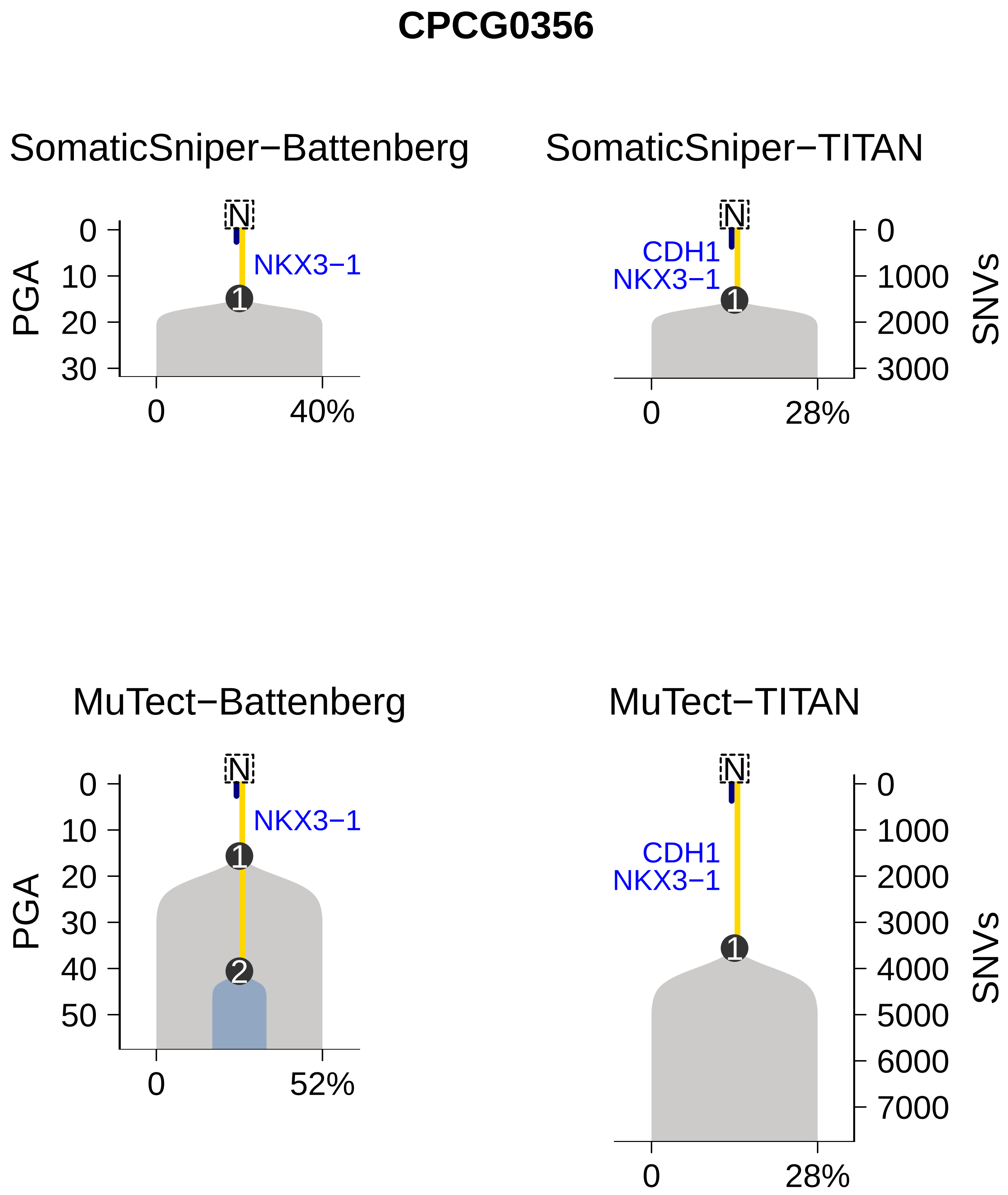
CPCG0356
SomaticSniper−Battenberg
SomaticSniper−TITAN
MuTect−Battenberg
MuTect−TITAN
N
NKX3−1
CDH1
1
2
PGA
SNVs
0
10
20
30
40
50
1000
2000
3000
4000
5000
6000
7000
40%
28%
52%

CPCG0357

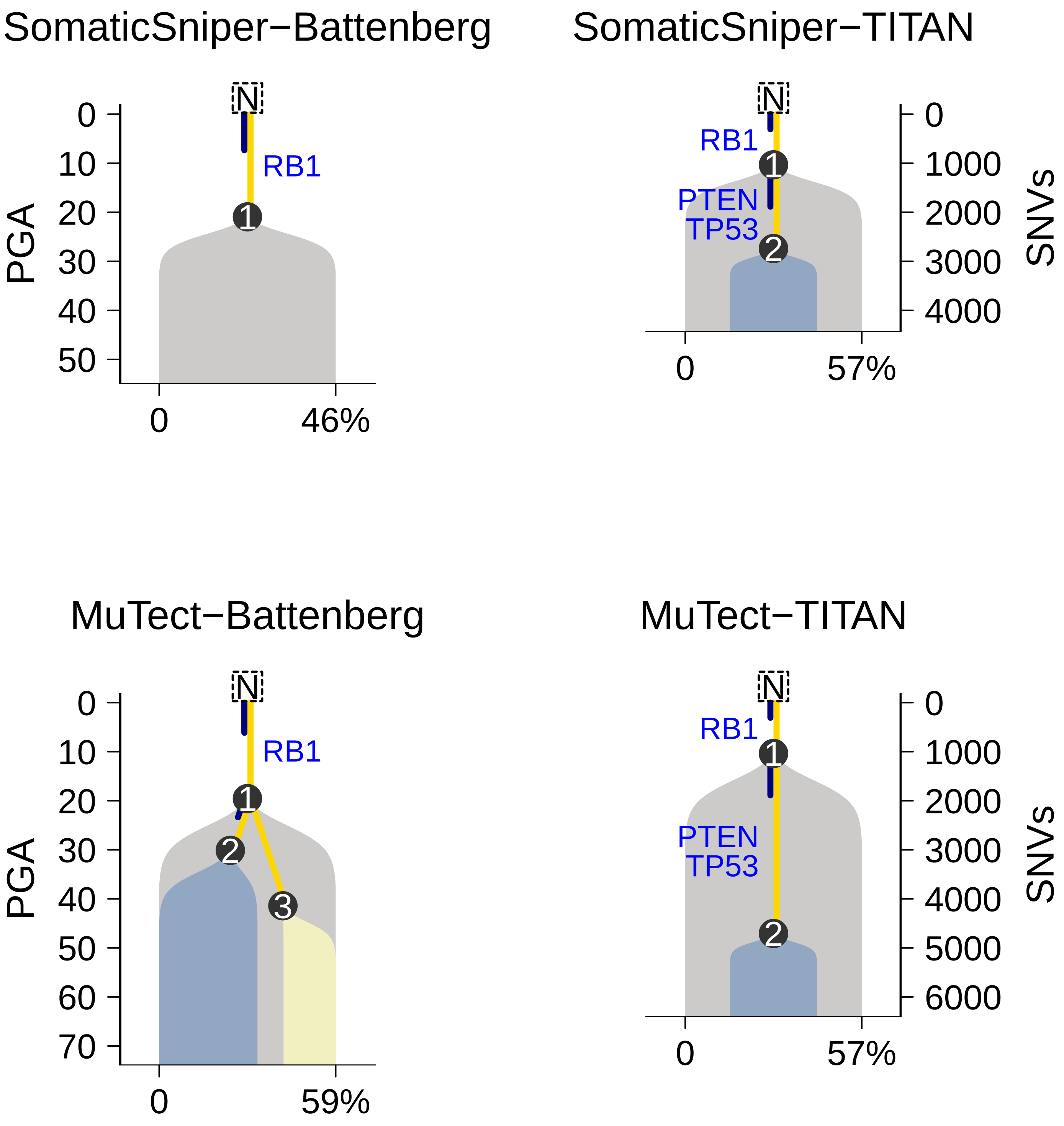
SomaticSniper−Battenberg
N
RB1
1
PGA
0
10
20
30
40
50
0
46%
SomaticSniper−TITAN
N
RB1
1
PTEN
TP53
2
0
1000
2000
3000
4000
SNVs
0
57%
MuTect−Battenberg
N
RB1
1
2
3
PGA
0
10
20
30
40
50
60
70
0
59%
MuTect−TITAN
N
RB1
1
PTEN
TP53
2
0
1000
2000
3000
4000
5000
6000
SNVs
0
57%

CPCG0358

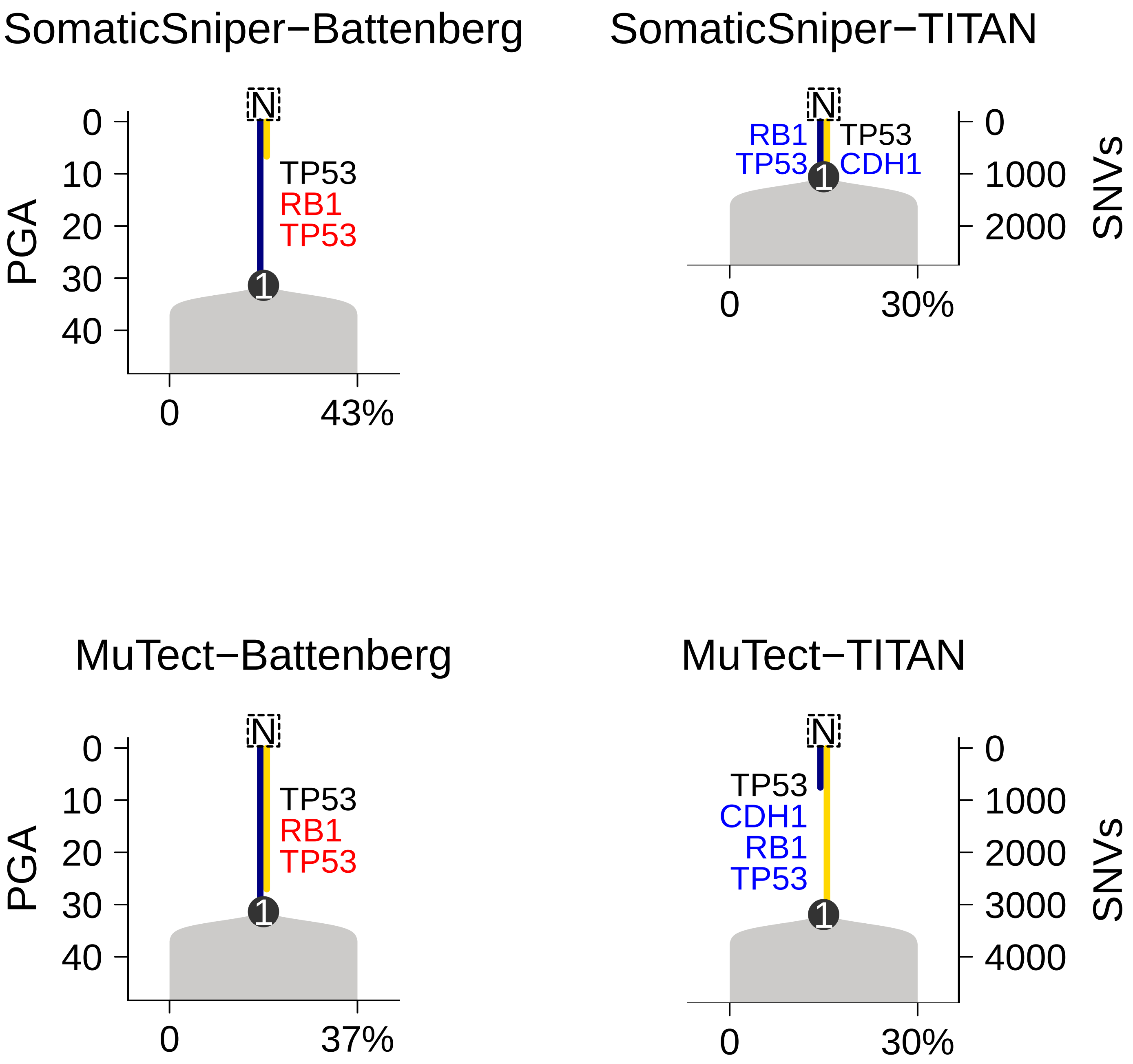
SomaticSniper−Battenberg
N
TP53
RB1
TP53
1
PGA
0
10
20
30
40
0
43%
SomaticSniper−TITAN
N
RB1
TP53
TP53
CDH1
1
0
1000
2000
SNVs
0
30%
MuTect−Battenberg
N
TP53
RB1
TP53
1
PGA
0
10
20
30
40
0
37%
MuTect−TITAN
N
TP53
CDH1
RB1
TP53
1
0
1000
2000
3000
4000
SNVs
0
30%

CPCG0360

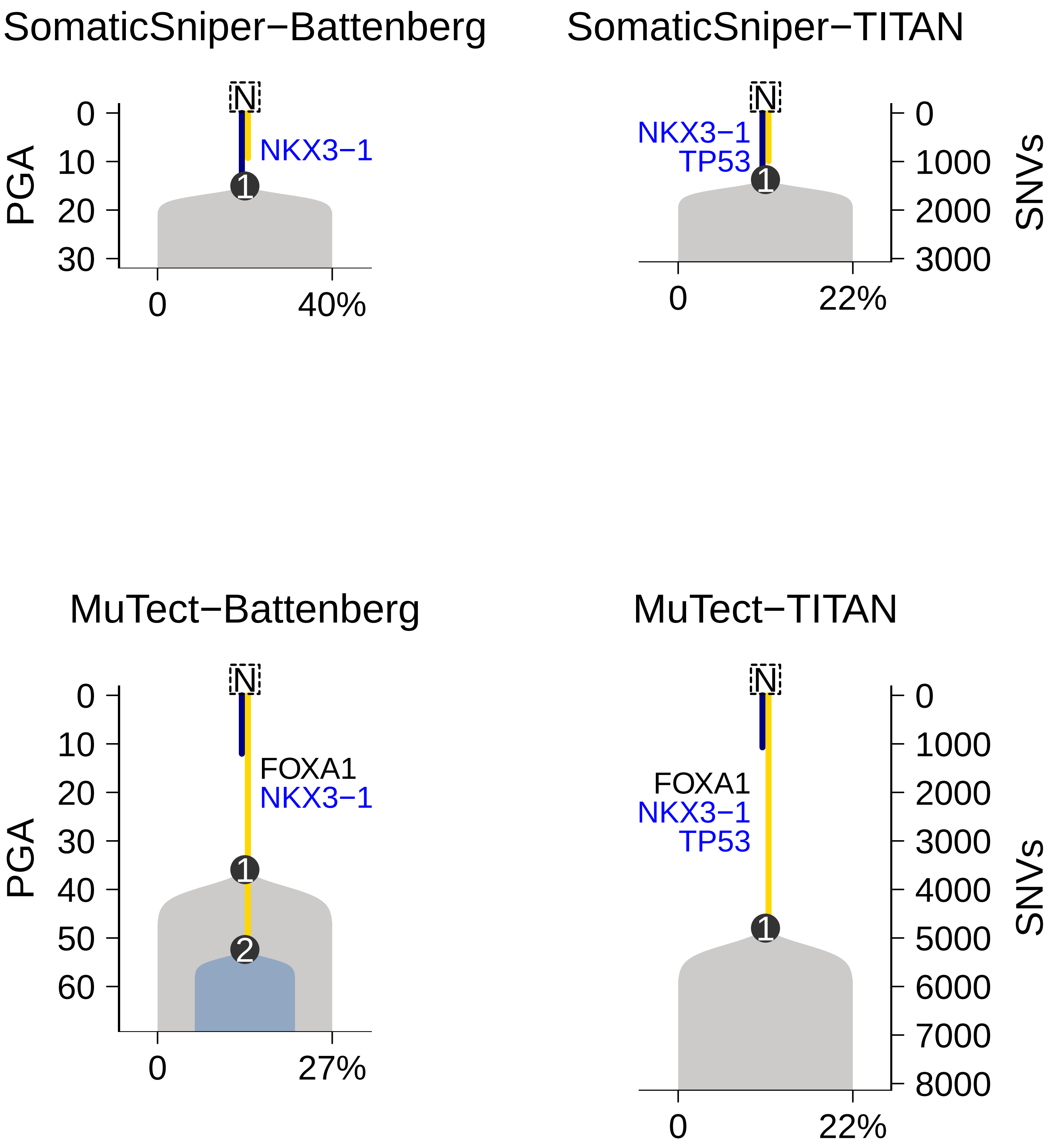
SomaticSniper−Battenberg
N
NKX3−1
1
PGA
0
10
20
30
0
40%
SomaticSniper−TITAN
N
NKX3−1
TP53
1
0
1000
2000
3000
SNVs
0
22%
MuTect−Battenberg
N
FOXA1
NKX3−1
1
2
PGA
0
10
20
30
40
50
60
0
27%
MuTect−TITAN
N
FOXA1
NKX3−1
TP53
1
0
1000
2000
3000
4000
5000
6000
7000
8000
SNVs
0
22%

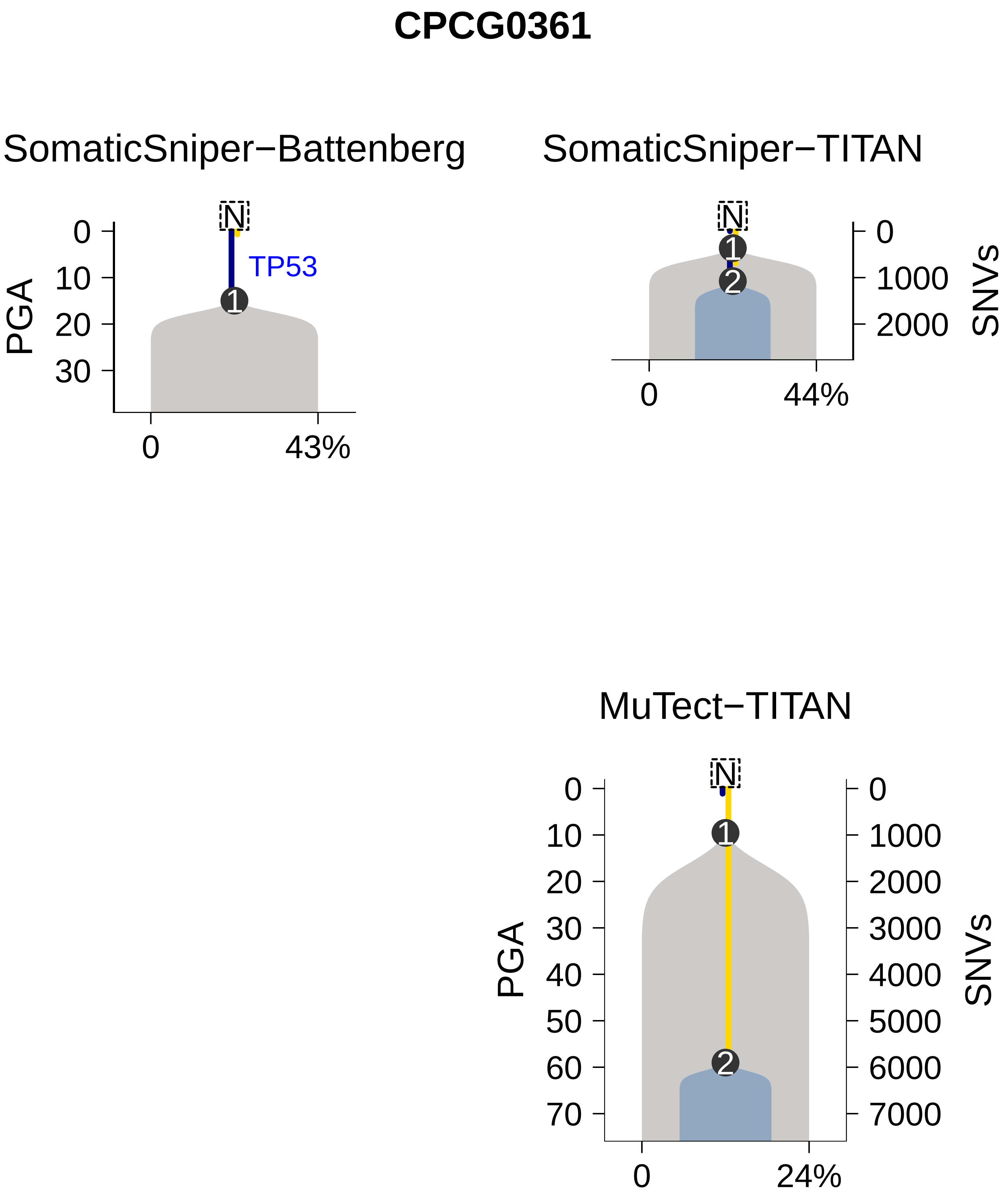
CPCG0361
SomaticSniper−Battenberg
N
TP53
1
PGA
0
10
20
30
0
43%
SomaticSniper−TITAN
N
1
2
0
1000
2000
SNVs
0
44%
MuTect−TITAN
N
1
2
PGA
0
10
20
30
40
50
60
70
0
1000
2000
3000
4000
5000
6000
7000
SNVs
0
24%

CPCG0362

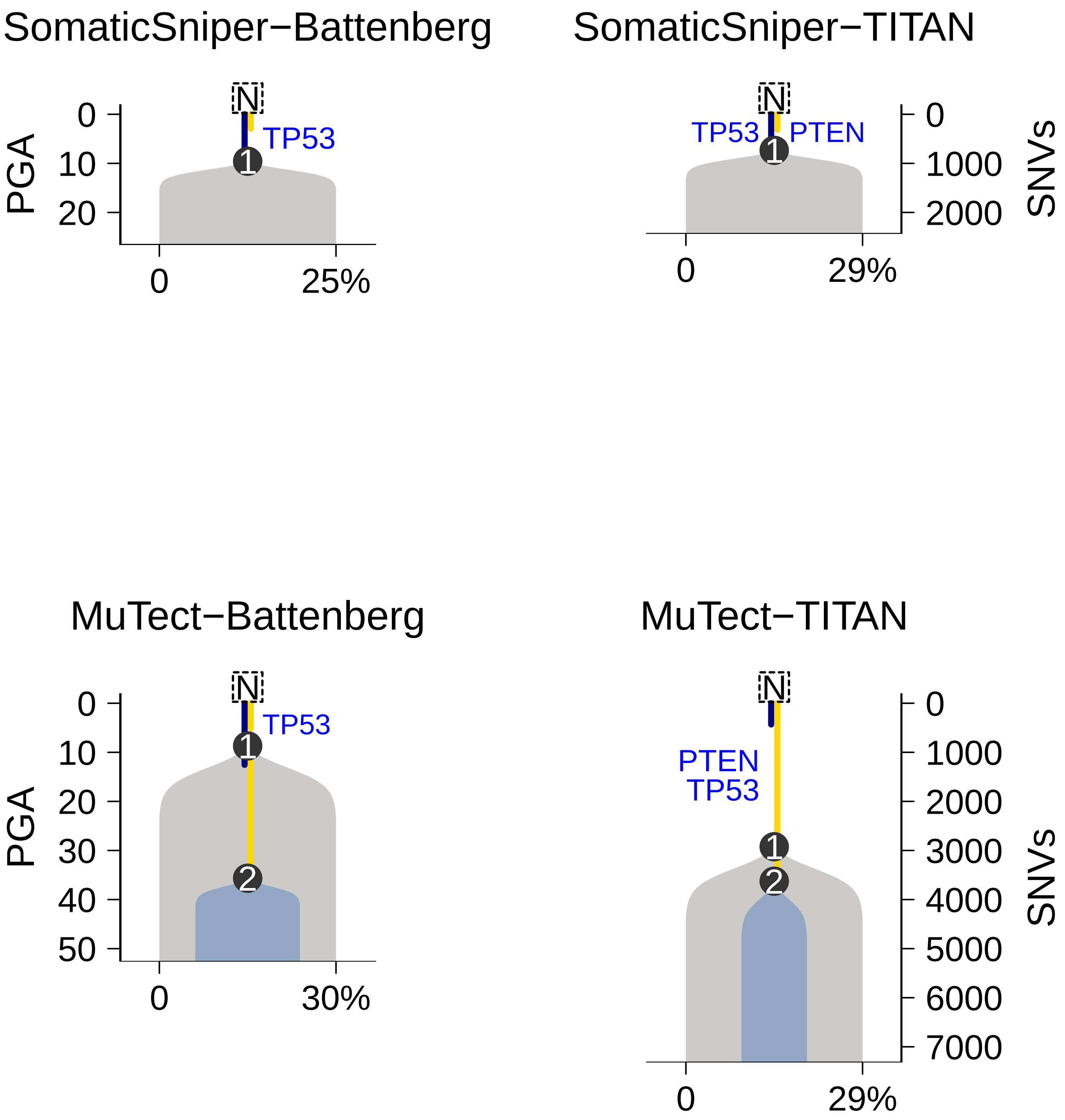

SomaticSniper−Battenberg
SomaticSniper−TITAN
MuTect−Battenberg
MuTect−TITAN
N
TP53
PTEN
1
2
PGA
SNVs
0
10
20
30
40
50
1000
2000
3000
4000
5000
6000
7000
25%
29%
30%

CPCG0363

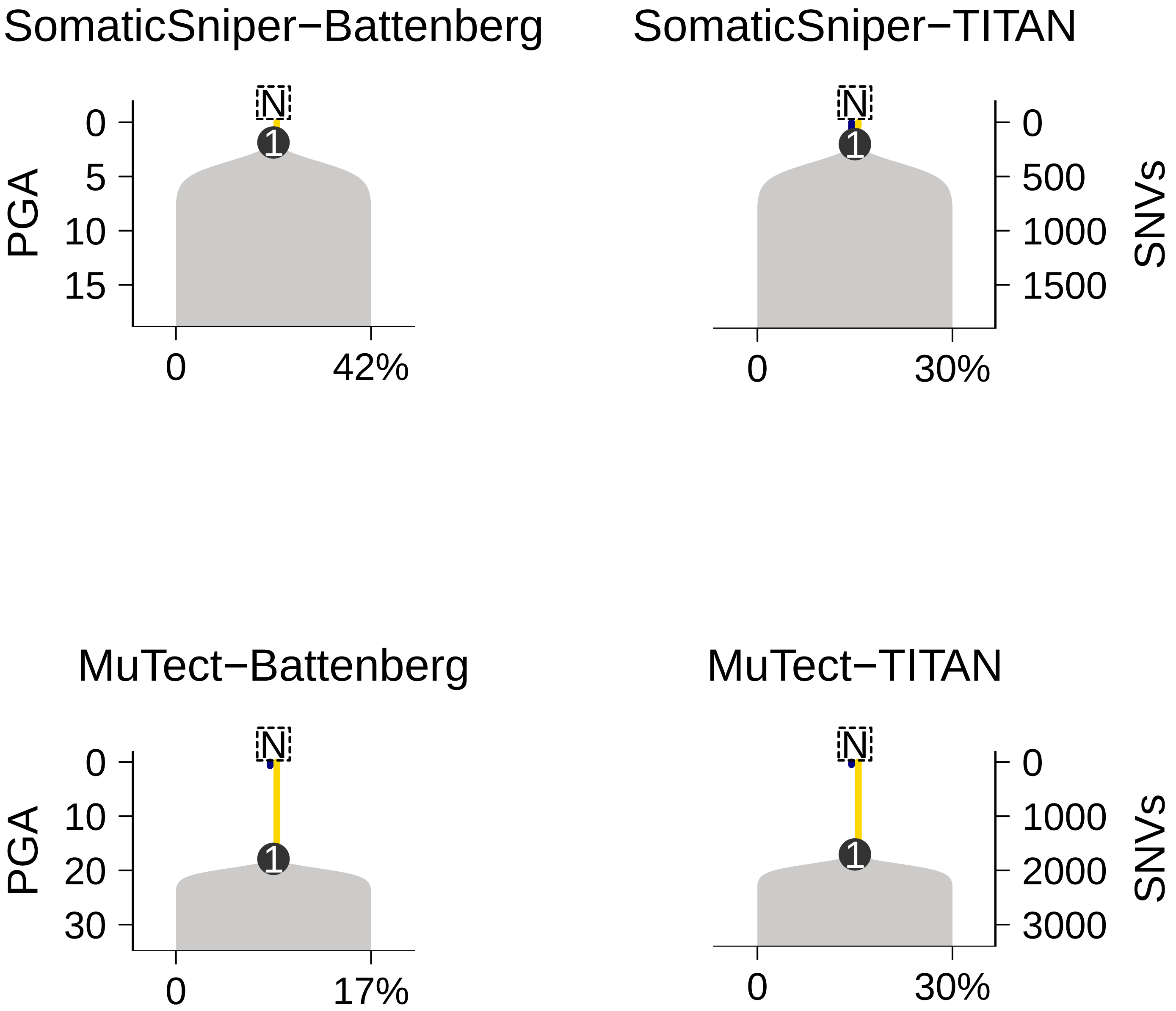

SomaticSniper−Battenberg
SomaticSniper−TITAN
N
1
PGA
0
5
10
15
0
42%
0
500
1000
1500
SNVs
0
30%
MuTect−Battenberg
MuTect−TITAN
PGA
0
10
20
30
0
17%
0
1000
2000
3000
SNVs
0
30%

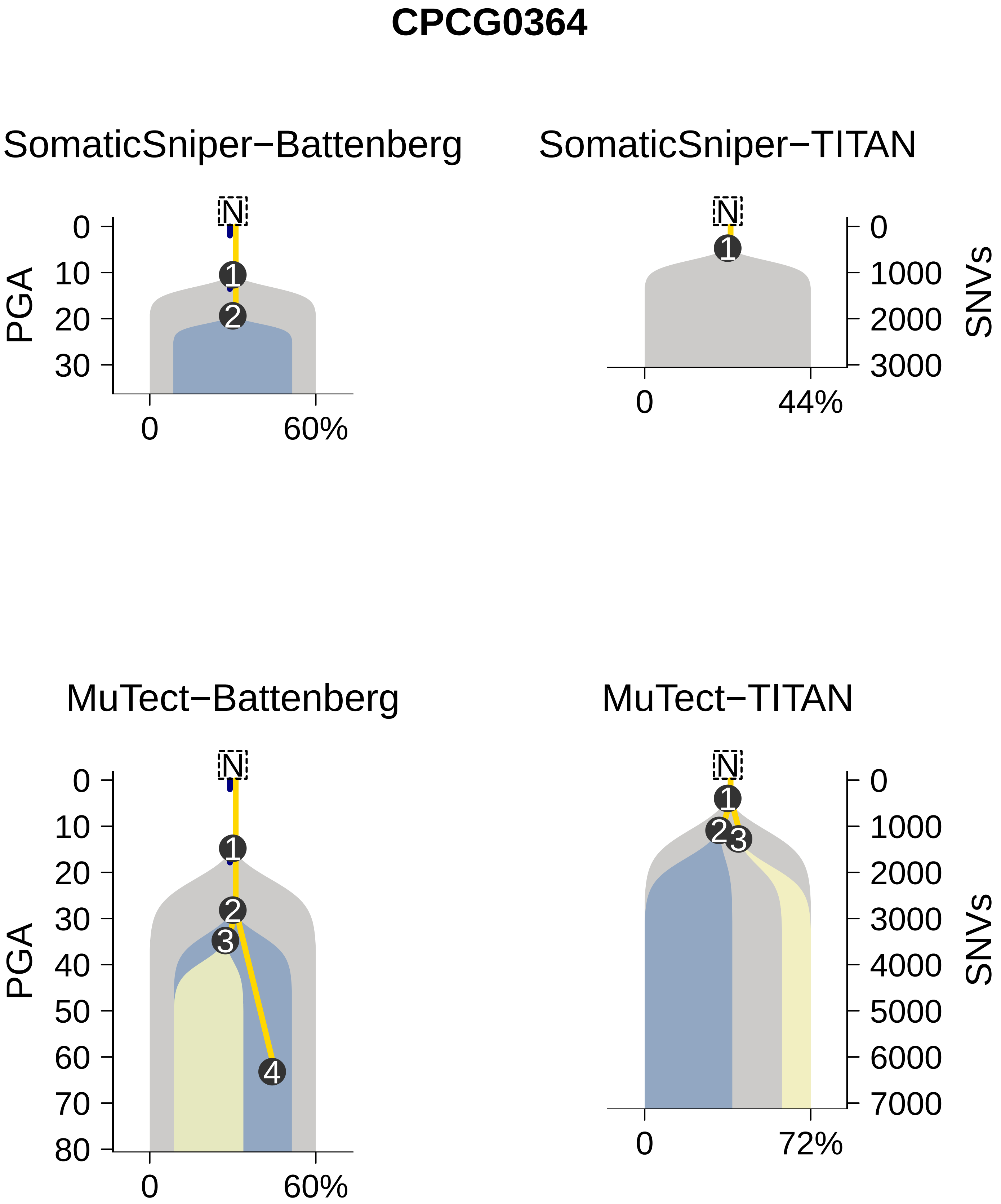
CPCG0364
SomaticSniper−Battenberg
SomaticSniper−TITAN
MuTect−Battenberg
MuTect−TITAN
N
1
2
3
4
PGA
SNVs
0
10
20
30
40
50
60
70
80
1000
2000
3000
4000
5000
6000
7000
60%
44%
72%

CPCG0365

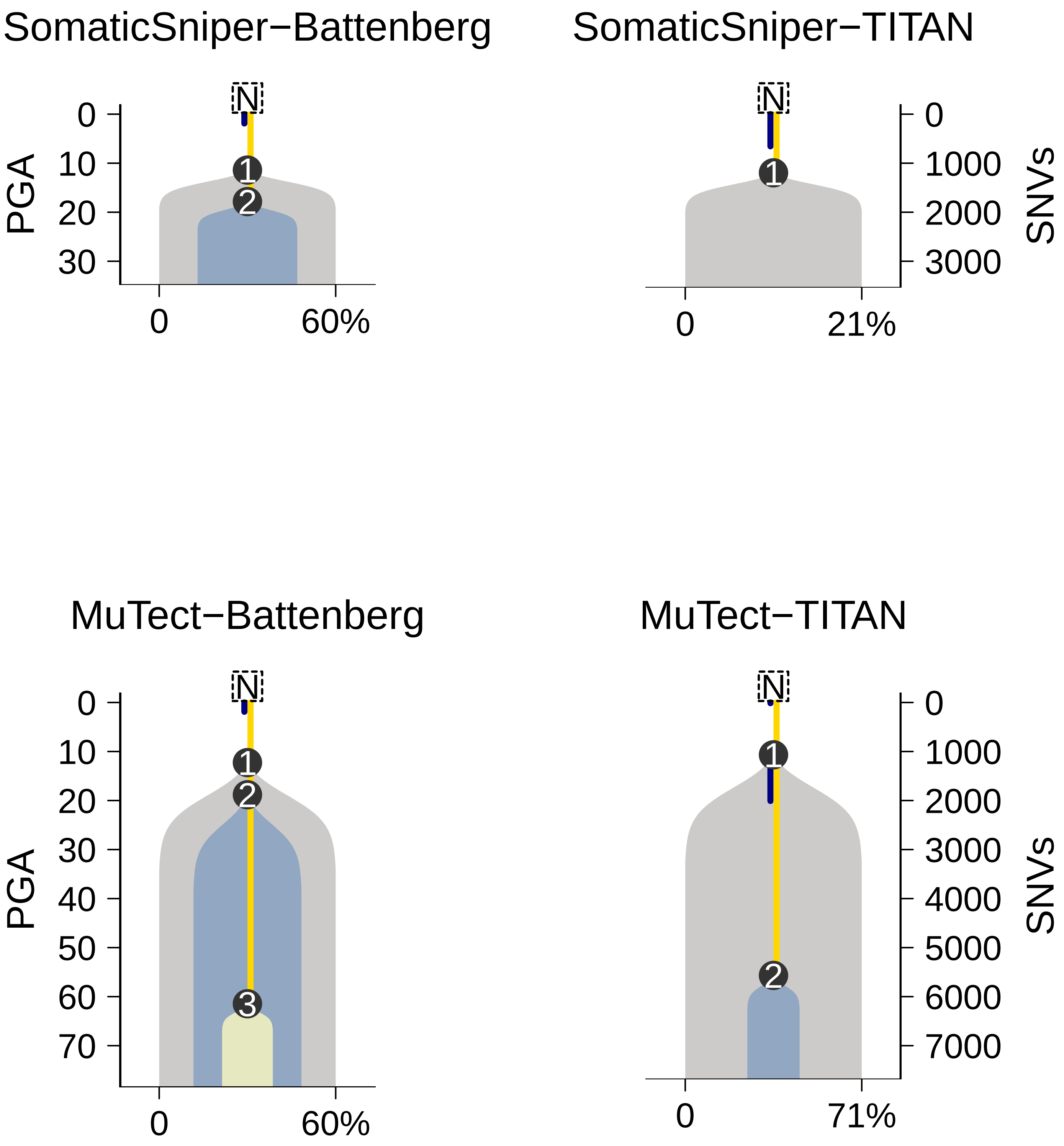

SomaticSniper−Battenberg
SomaticSniper−TITAN
MuTect−Battenberg
MuTect−TITAN
N
1
2
3
PGA
SNVs
0
10
20
30
40
50
60
70
1000
2000
3000
4000
5000
6000
7000
60%
21%
71%

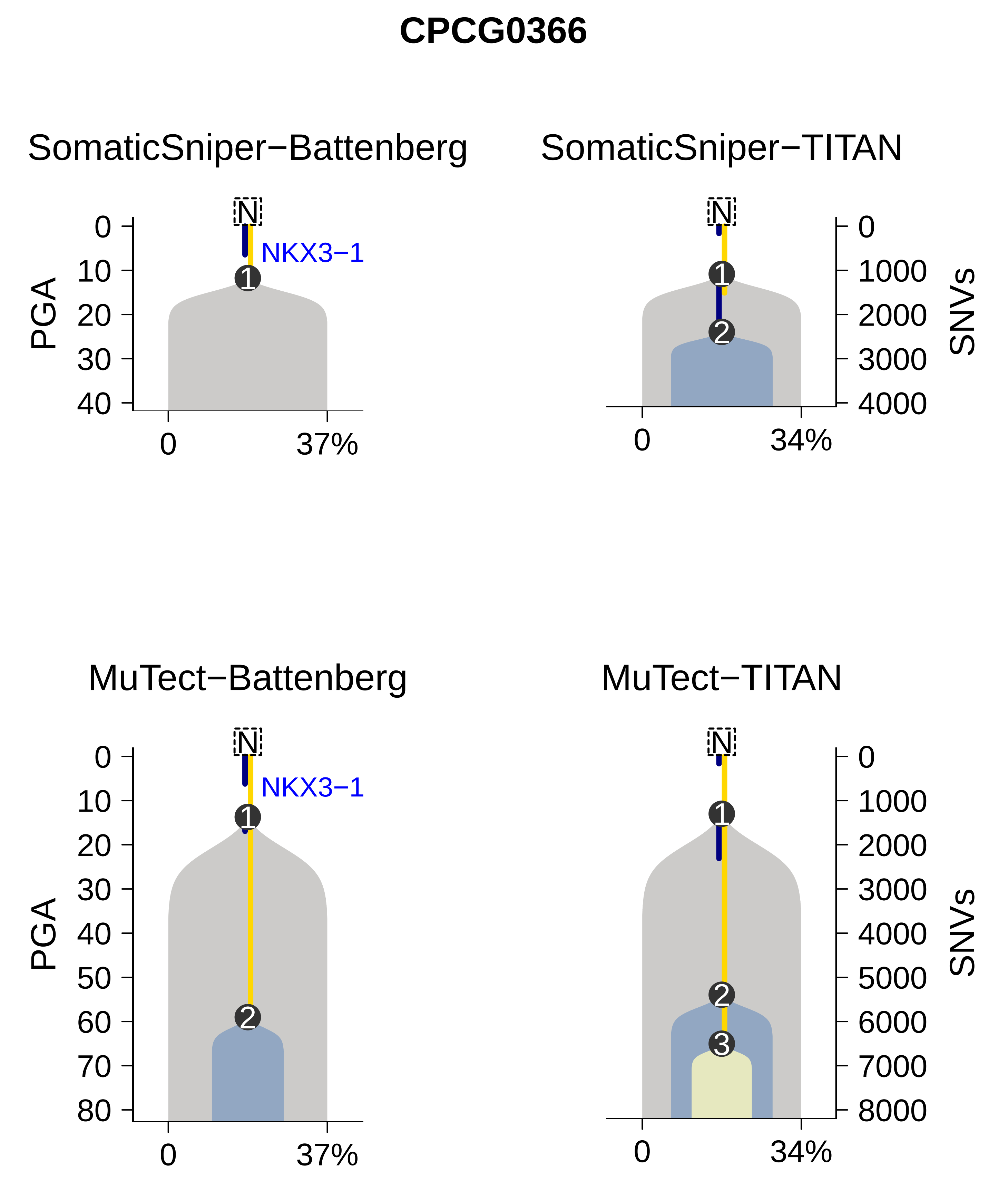
CPCG0366
SomaticSniper−Battenberg
SomaticSniper−TITAN
N
NKX3−1
1
2
PGA
SNVs
0
10
20
30
40
0
1000
2000
3000
4000
0
37%
0
34%
MuTect−Battenberg
MuTect−TITAN
NKX3−1
3
50
60
70
80
5000
6000
7000
8000

CPCG0368

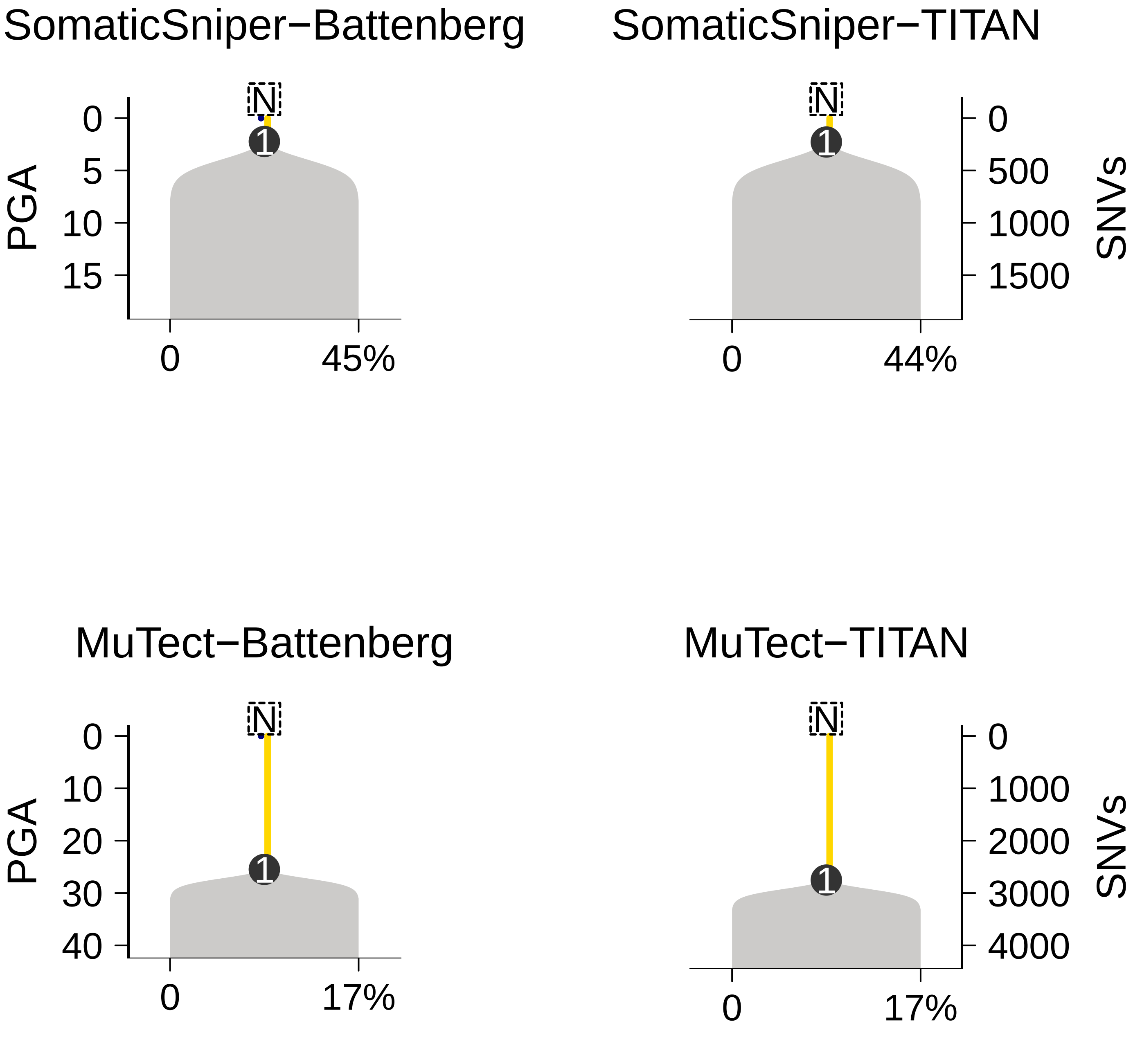

SomaticSniper−Battenberg
SomaticSniper−TITAN
N
1
PGA
0
5
10
15
0
45%
0
44%
0
500
1000
1500
SNVs
MuTect−Battenberg
MuTect−TITAN
N
1
PGA
0
10
20
30
40
0
17%
0
17%
0
1000
2000
3000
4000
SNVs

CPCG0369

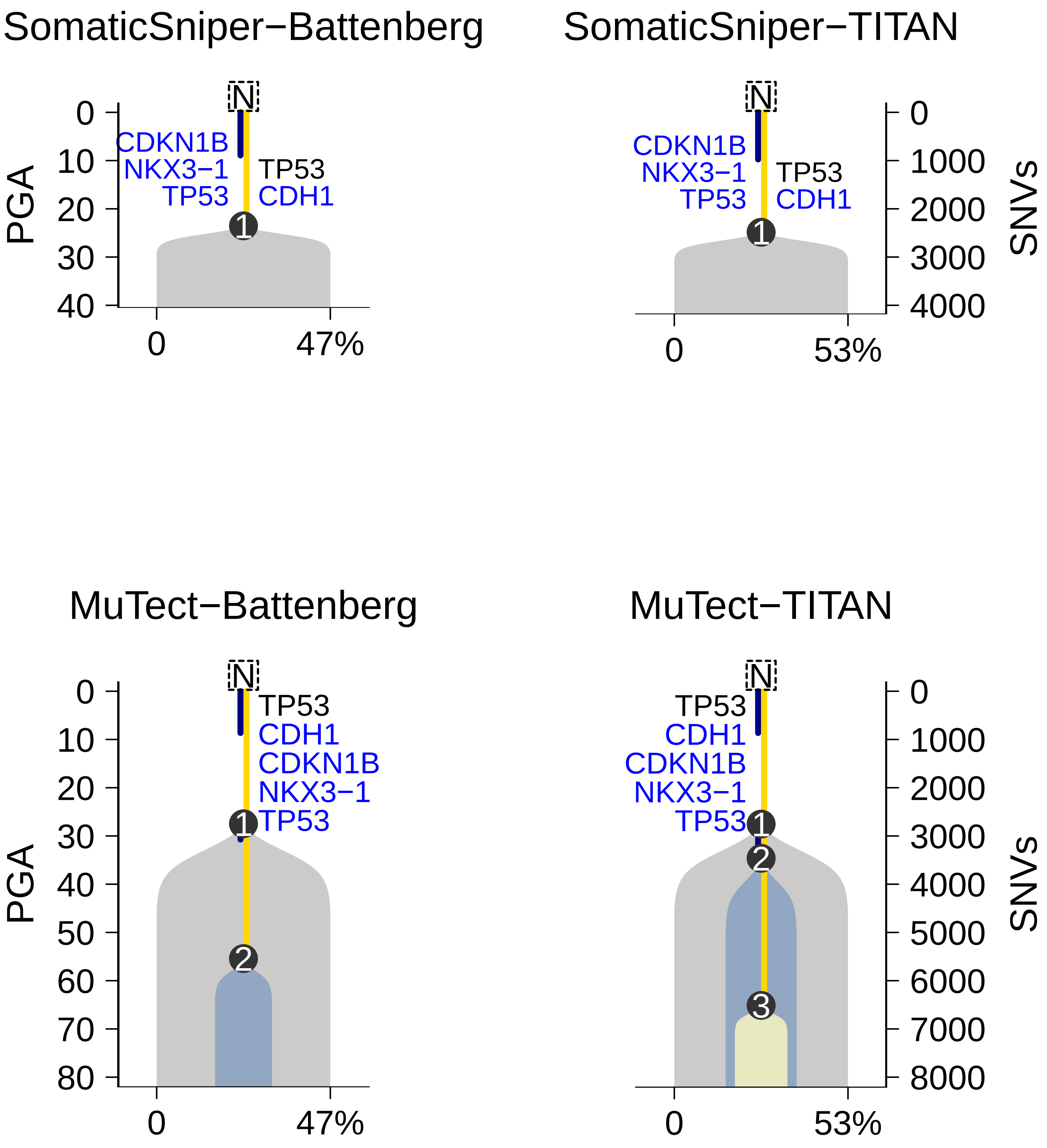
SomaticSniper−Battenberg
SomaticSniper−TITAN
MuTect−Battenberg
MuTect−TITAN
N
CDKN1B
NKX3−1
TP53
TP53
CDH1
PGA
SNVs
0
10
20
30
40
50
60
70
80
1000
2000
3000
4000
5000
6000
7000
8000
47%
53%
1
2
3

CPCG0371

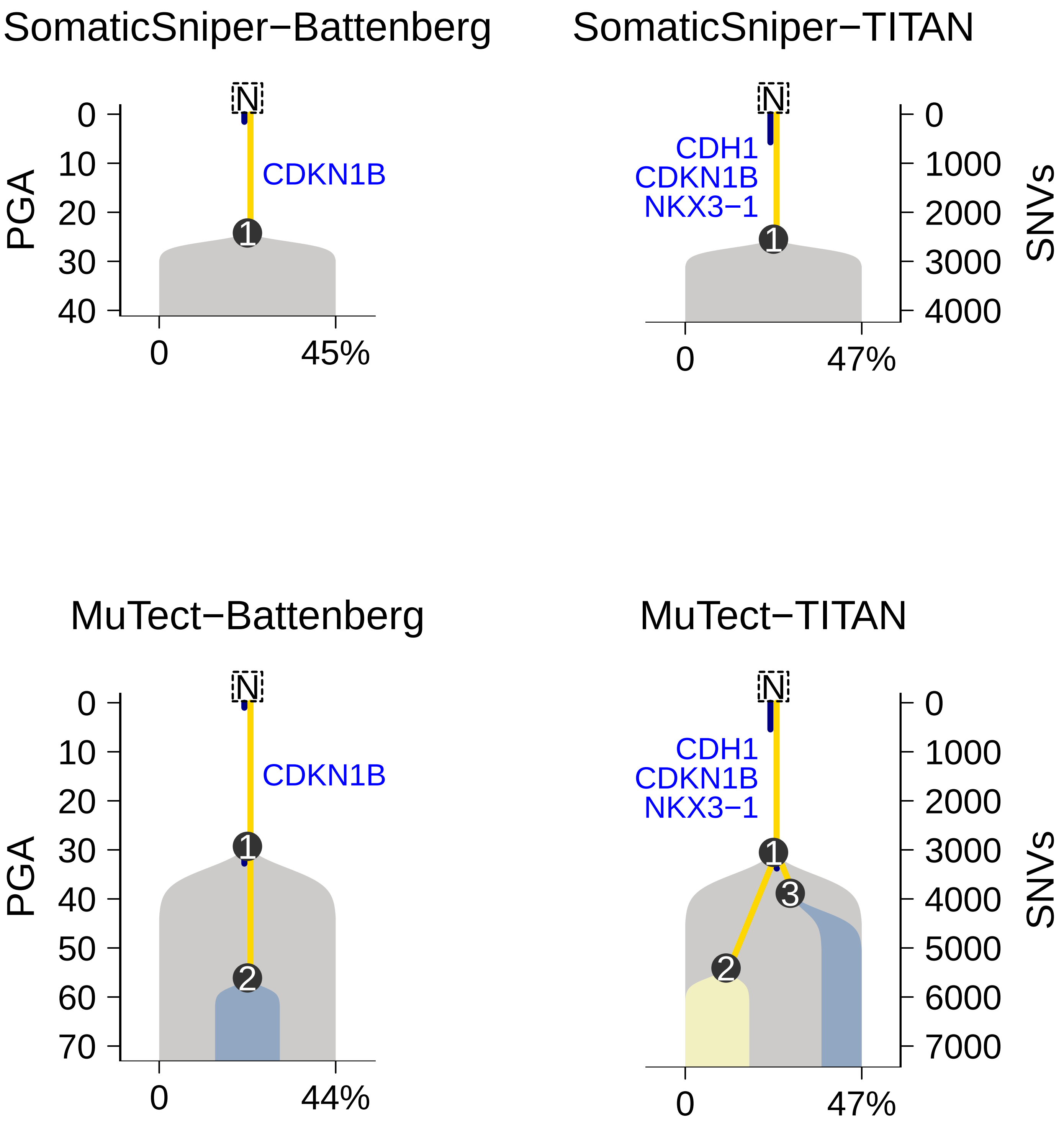

SomaticSniper−Battenberg
N
CDKN1B
1
PGA
0
10
20
30
40
0
45%
SomaticSniper−TITAN
N
CDH1
CDKN1B
NKX3−1
1
0
1000
2000
3000
4000
SNVs
0
47%
MuTect−Battenberg
N
CDKN1B
1
2
PGA
0
10
20
30
40
50
60
70
0
44%
MuTect−TITAN
N
CDH1
CDKN1B
NKX3−1
1
2
3
0
1000
2000
3000
4000
5000
6000
7000
SNVs
0
47%

CPCG0372

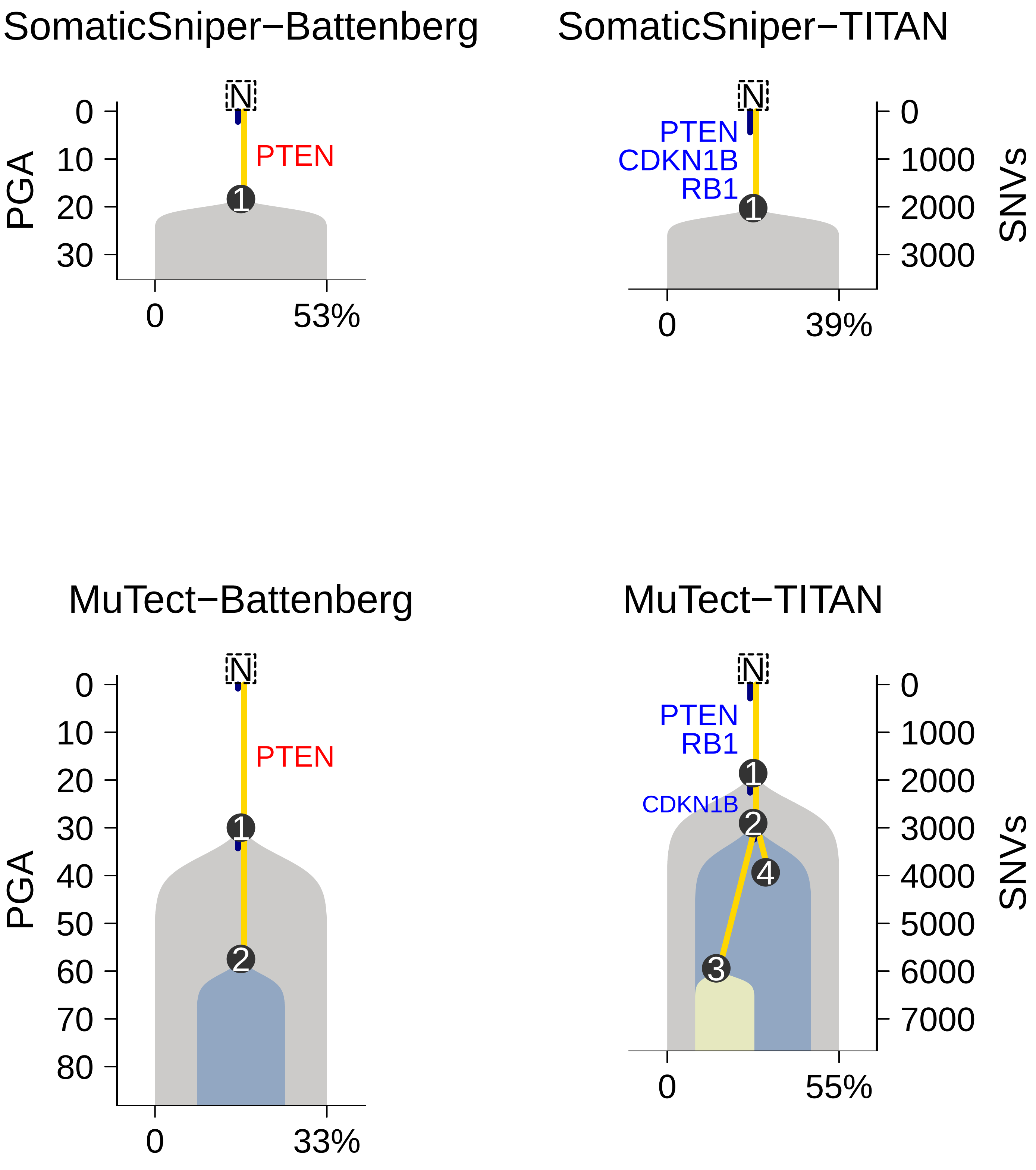

SomaticSniper−Battenberg
N
PTEN
1
PGA
0
10
20
30
0
53%
SomaticSniper−TITAN
N
PTEN
CDKN1B
RB1
1
0
1000
2000
3000
SNVs
0
39%
MuTect−Battenberg
N
PTEN
1
2
PGA
0
10
20
30
40
50
60
70
80
0
33%
MuTect−TITAN
N
PTEN
RB1
CDKN1B
1
2
3
4
0
1000
2000
3000
4000
5000
6000
7000
SNVs
0
55%

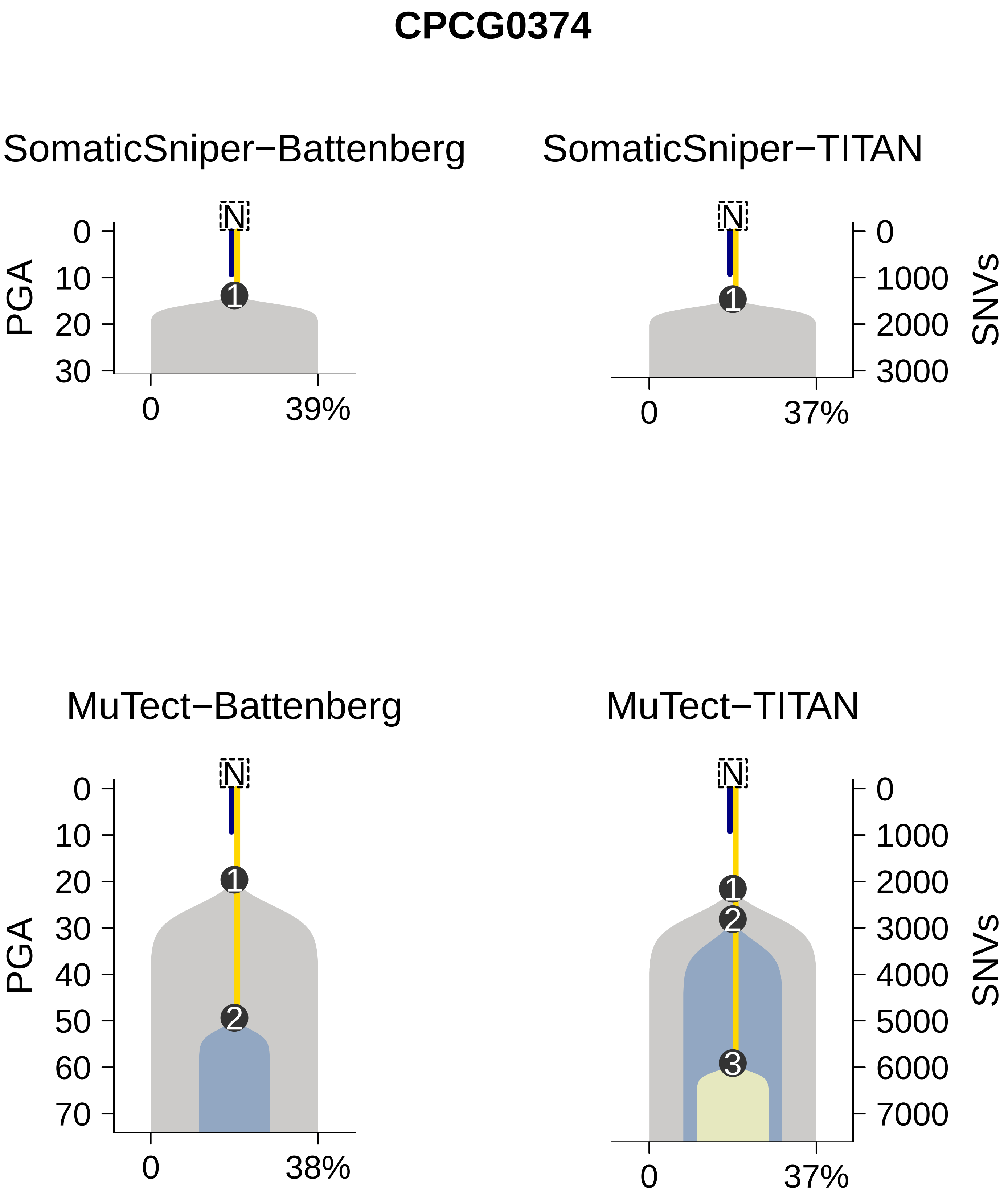
CPCG0374
SomaticSniper−Battenberg
SomaticSniper−TITAN
MuTect−Battenberg
MuTect−TITAN
N
1
2
3
PGA
SNVs
0
10
20
30
40
50
60
70
1000
2000
3000
4000
5000
6000
7000
39%
37%
38%

CPCG0375

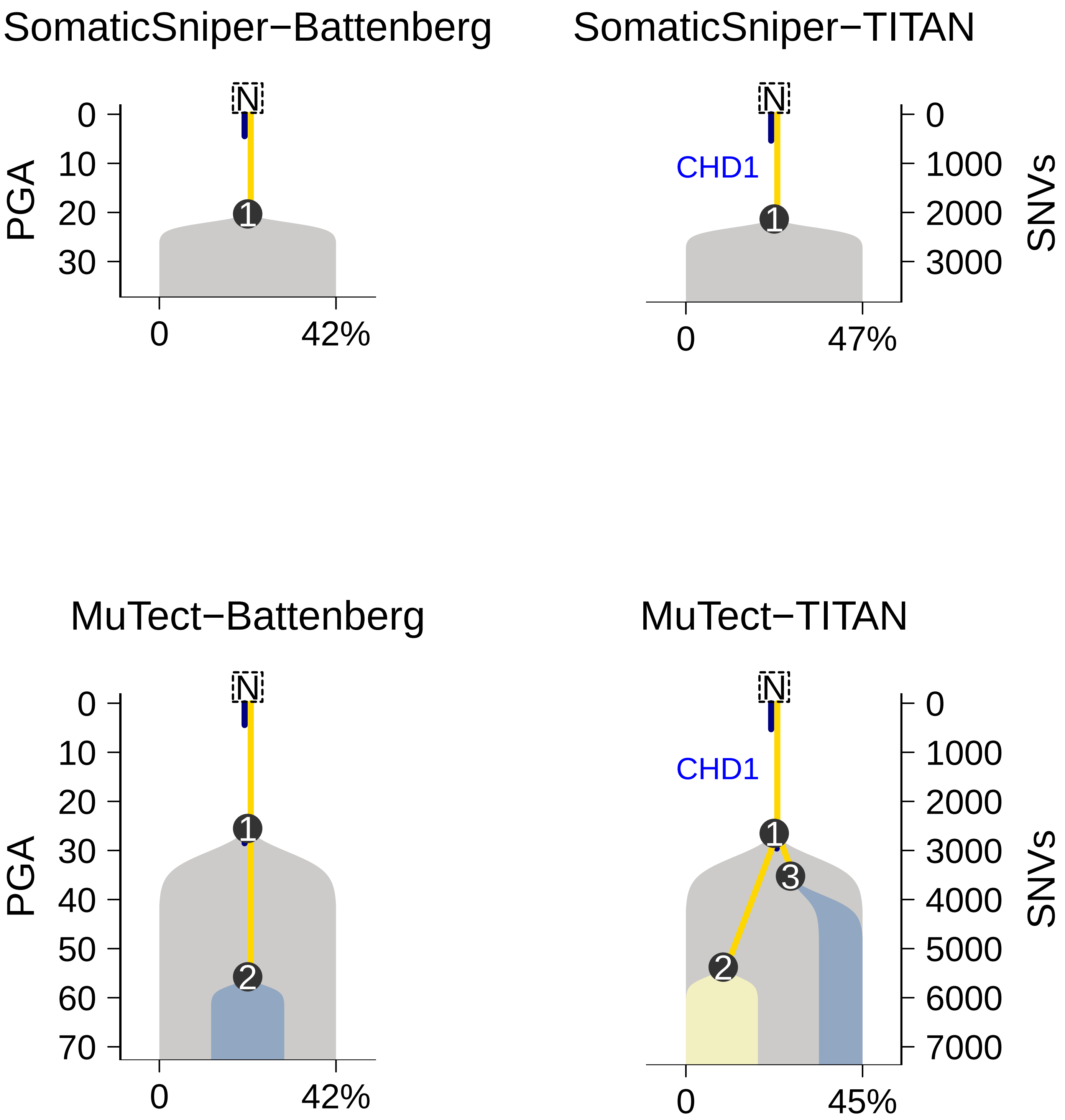

SomaticSniper−Battenberg
SomaticSniper−TITAN
MuTect−Battenberg
MuTect−TITAN
N
CHD1
PGA
SNVs
0
10
20
30
40
50
60
70
1000
2000
3000
4000
5000
6000
7000
42%
47%
45%
1
2
3

CPCG0378

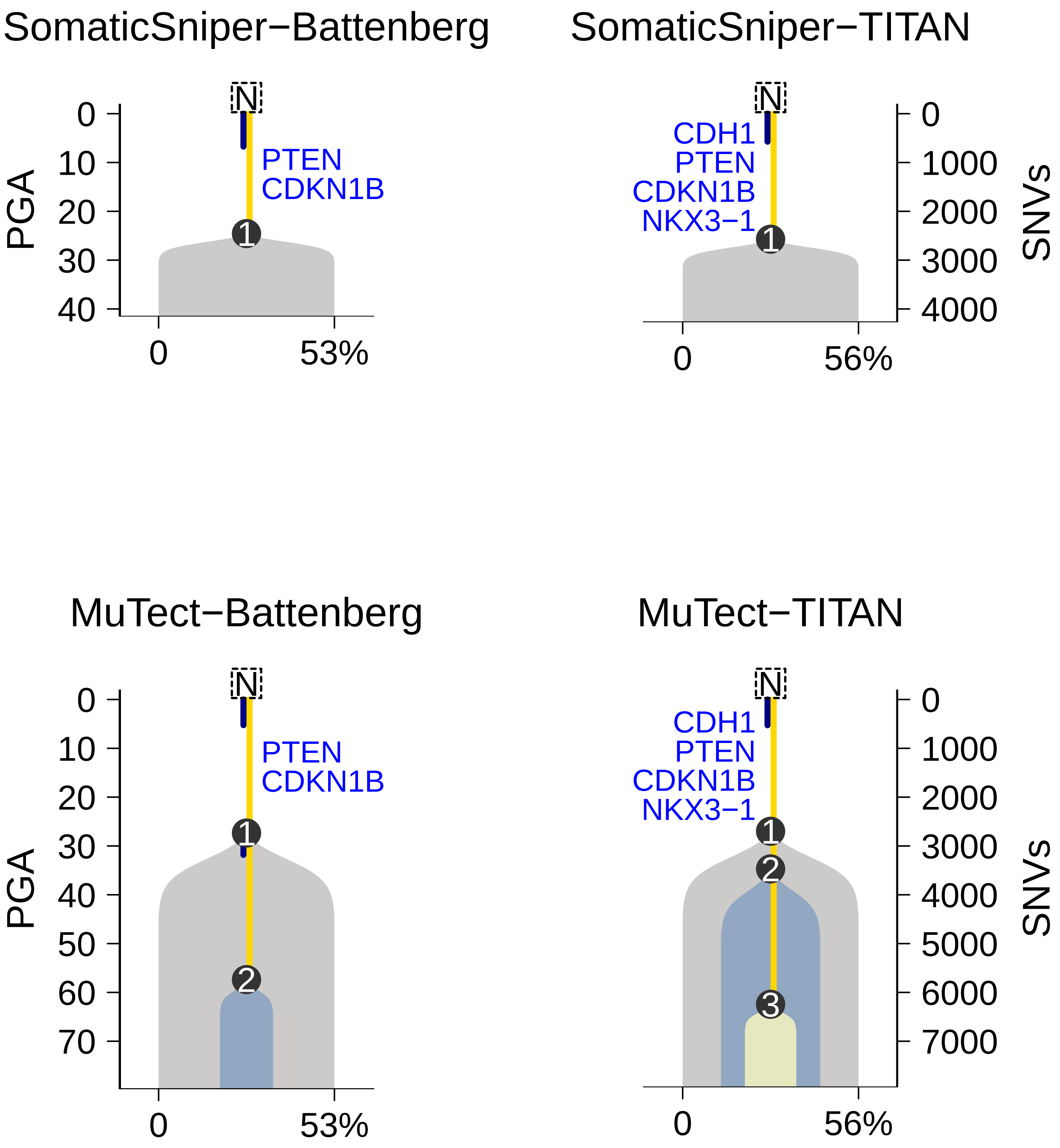

SomaticSniper−Battenberg
SomaticSniper−TITAN
MuTect−Battenberg
MuTect−TITAN
N
PTEN
CDKN1B
CDH1
NKX3−1
PGA
SNVs
0
10
20
30
40
50
60
70
1000
2000
3000
4000
5000
6000
7000
53%
56%
1
2
3

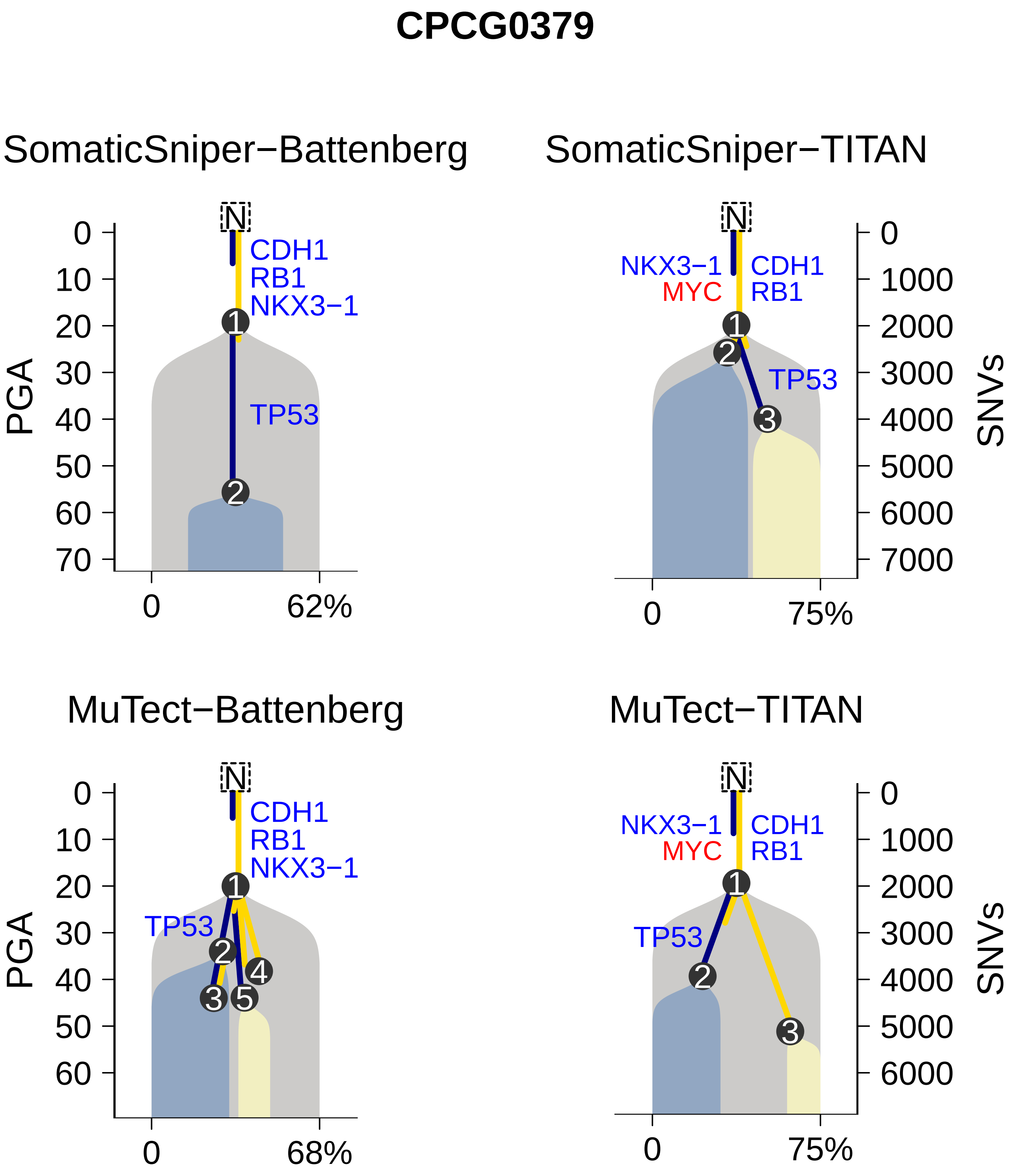
CPCG0379
SomaticSniper−Battenberg
SomaticSniper−TITAN
MuTect−Battenberg
MuTect−TITAN
N
CDH1
RB1
NKX3−1
MYC
TP53
PGA
SNVs
0
10
20
30
40
50
60
70
1000
2000
3000
4000
5000
6000
7000
62%
75%
68%

CPCG0380

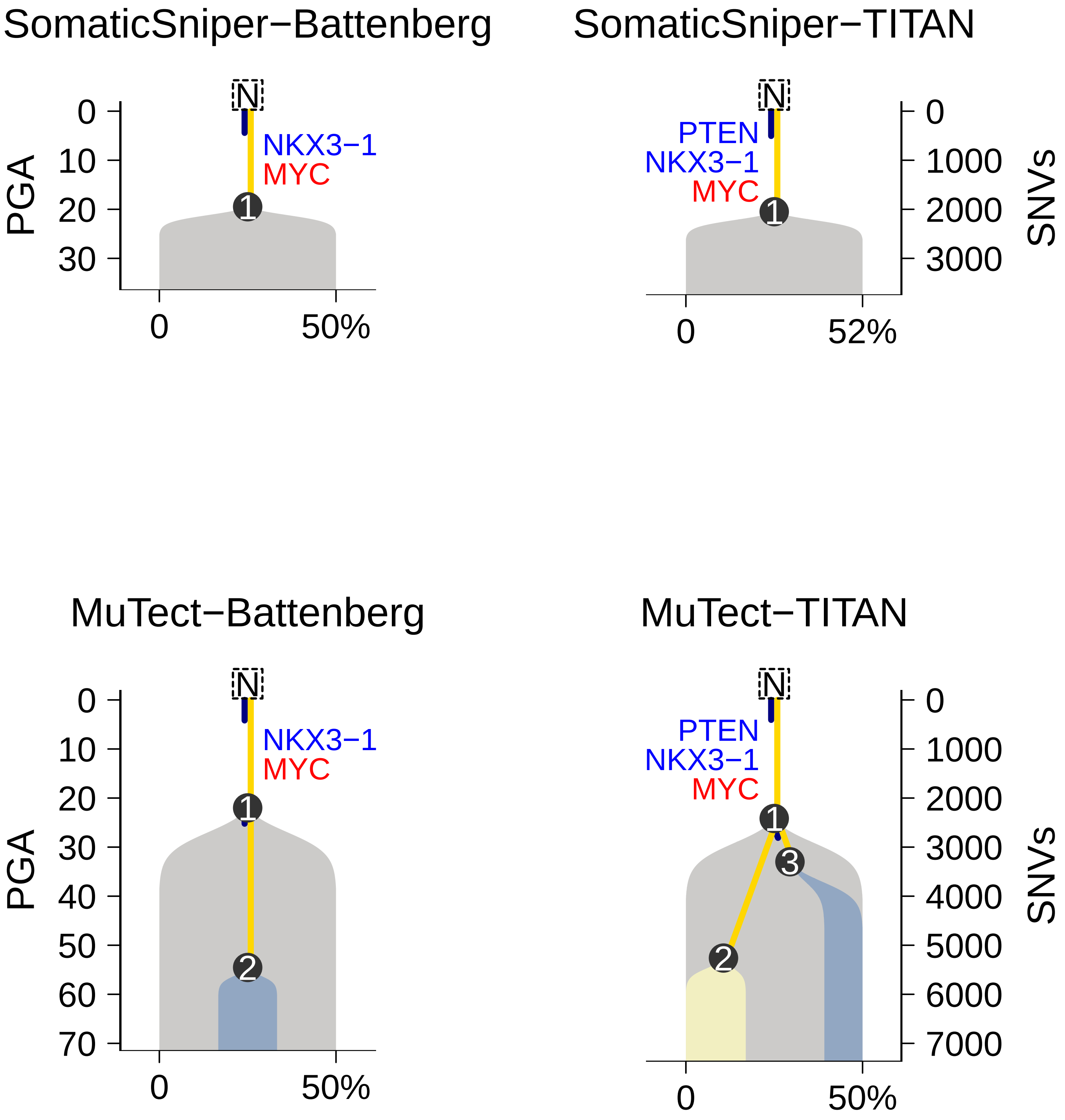
SomaticSniper−Battenberg
N
NKX3−1
MYC
1
PGA
0
10
20
30
0
50%
SomaticSniper−TITAN
N
PTEN
NKX3−1
MYC
1
SNVs
0
1000
2000
3000
0
52%
MuTect−Battenberg
N
NKX3−1
MYC
1
2
PGA
0
10
20
30
40
50
60
70
0
50%
MuTect−TITAN
N
PTEN
NKX3−1
MYC
1
2
3
SNVs
0
1000
2000
3000
4000
5000
6000
7000
0
50%

CPCG0381

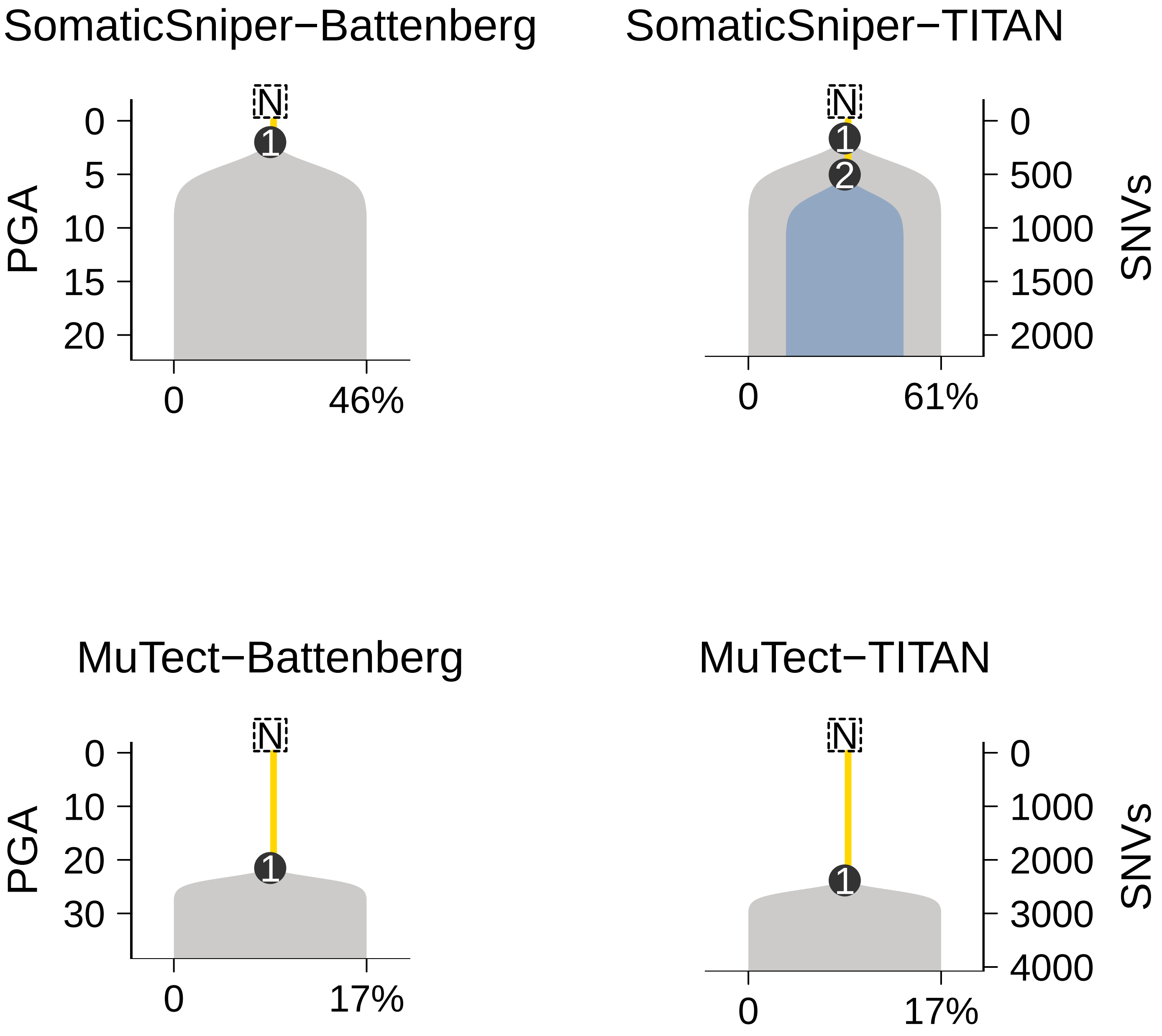
SomaticSniper−Battenberg
SomaticSniper−TITAN
MuTect−Battenberg
MuTect−TITAN
N
1
2
PGA
SNVs
0
5
10
15
20
30
500
1000
1500
2000
3000
4000
46%
61%
17%

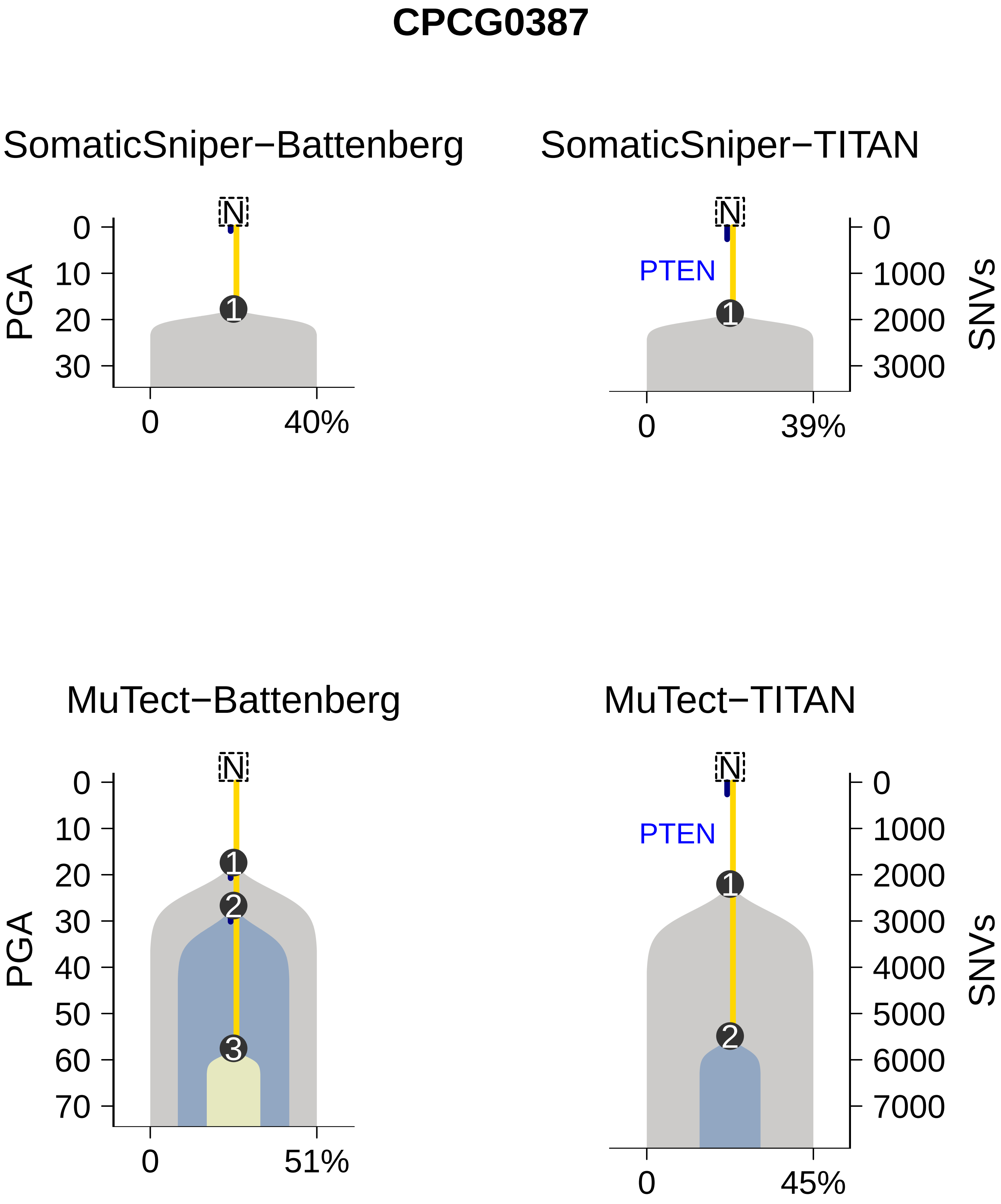
CPCG0387
SomaticSniper−Battenberg
SomaticSniper−TITAN
MuTect−Battenberg
MuTect−TITAN
N
PTEN
PGA
SNVs
0
10
20
30
40
50
60
70
1000
2000
3000
4000
5000
6000
7000
40%
39%
51%
45%
1
2
3

CPCG0388

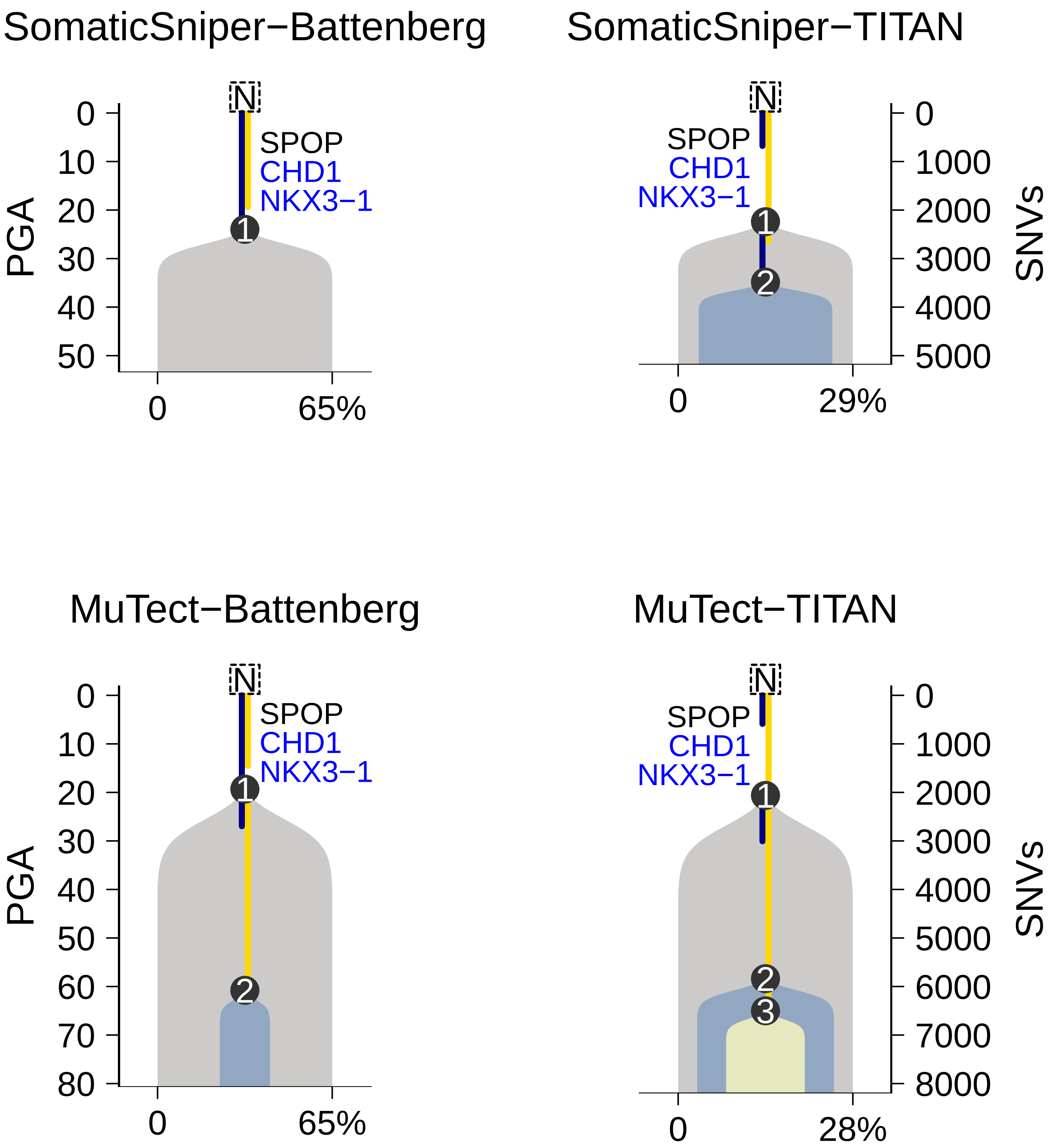

SomaticSniper−Battenberg
N
SPOP
CHD1
NKX3−1
1
PGA
0
10
20
30
40
50
0
65%
SomaticSniper−TITAN
N
SPOP
CHD1
NKX3−1
1
2
0
1000
2000
3000
4000
5000
SNVs
0
29%
MuTect−Battenberg
N
SPOP
CHD1
NKX3−1
1
2
PGA
0
10
20
30
40
50
60
70
80
0
65%
MuTect−TITAN
N
SPOP
CHD1
NKX3−1
1
2
3
0
1000
2000
3000
4000
5000
6000
7000
8000
SNVs
0
28%

CPCG0391

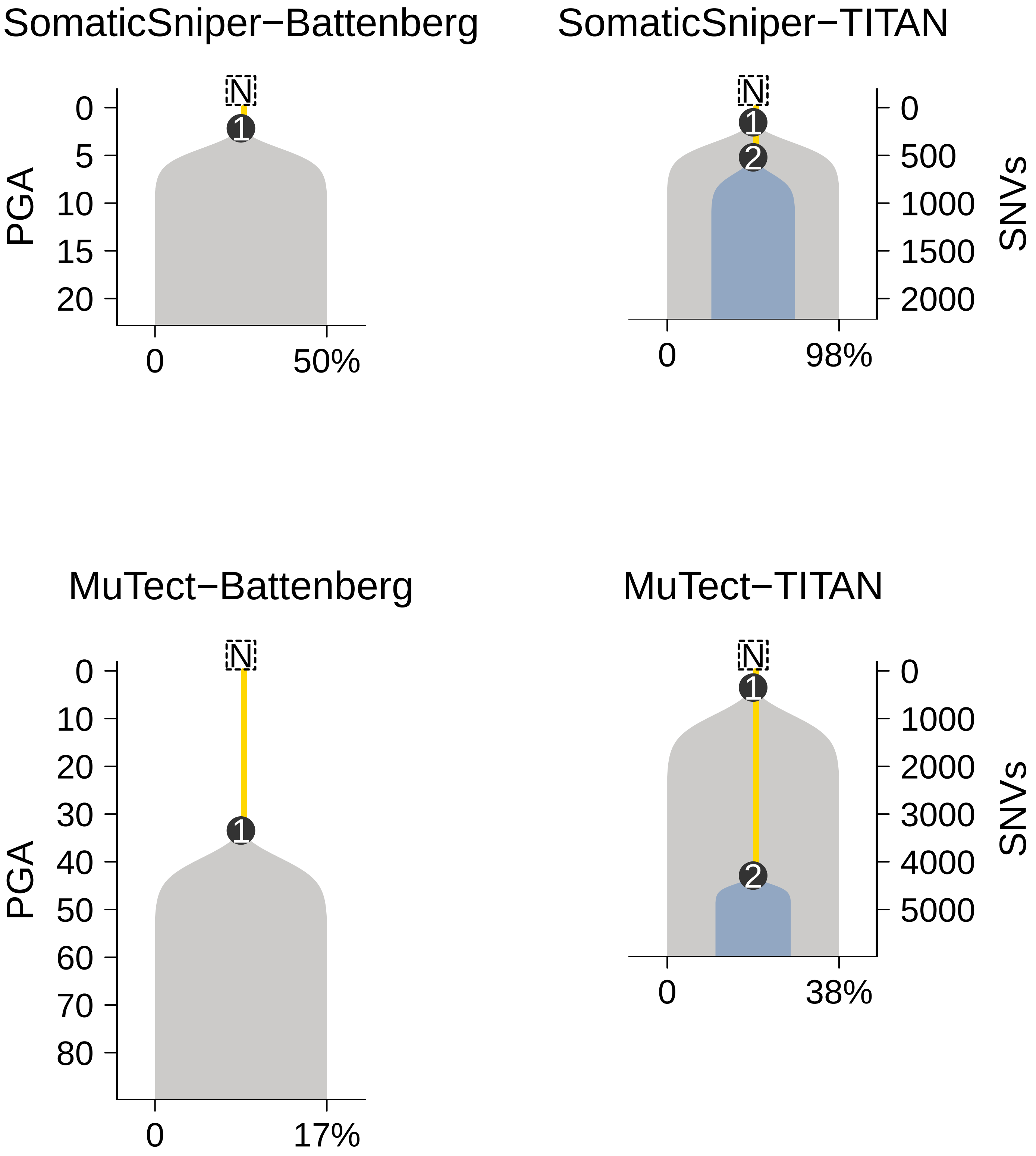

SomaticSniper−Battenberg
SomaticSniper−TITAN
N
1
2
PGA
SNVs
0
5
10
15
20
0
500
1000
1500
2000
0
50%
98%
MuTect−Battenberg
MuTect−TITAN
0
10
20
30
40
50
60
70
80
1000
2000
3000
4000
5000
17%
38%

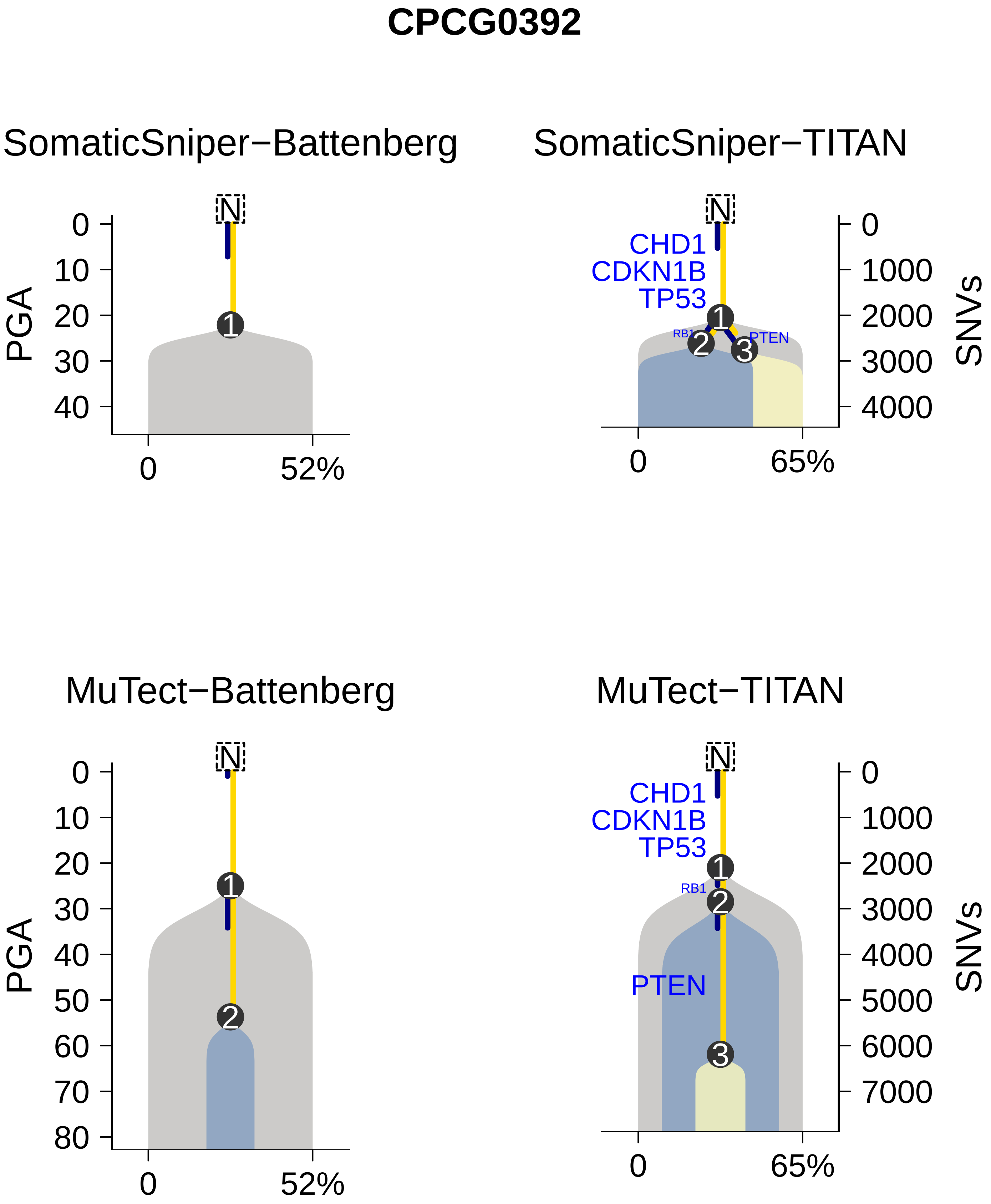
CPCG0392
SomaticSniper−Battenberg
SomaticSniper−TITAN
MuTect−Battenberg
MuTect−TITAN
N
1
2
3
PGA
SNVs
0
10
20
30
40
50
60
70
80
1000
2000
3000
4000
5000
6000
7000
52%
65%
CHD1
CDKN1B
TP53
RB1
PTEN

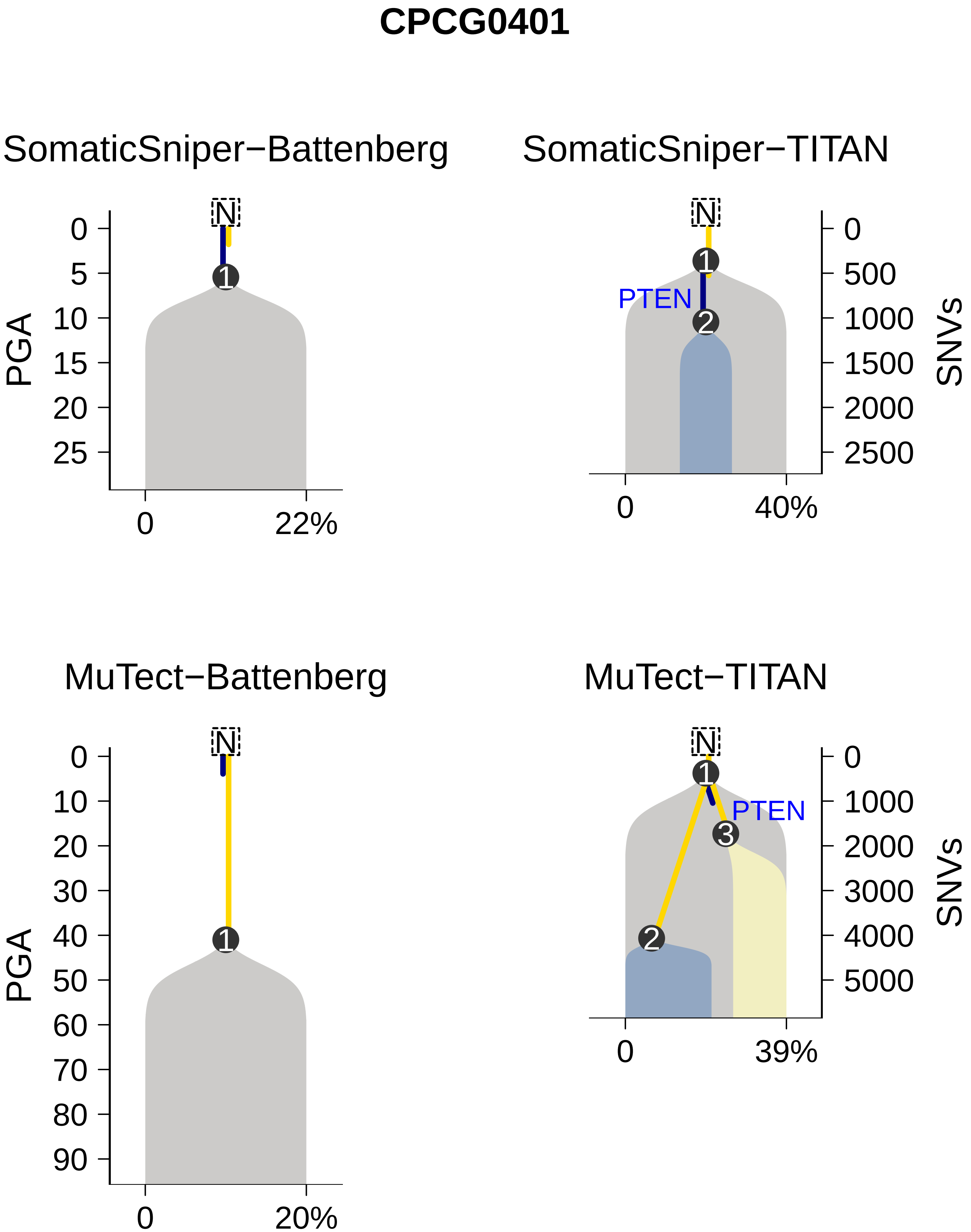
CPCG0401
SomaticSniper−Battenberg
SomaticSniper−TITAN
MuTect−Battenberg
MuTect−TITAN
N
1
2
3
PTEN
PGA
SNVs
0
5
10
15
20
25
30
40
50
60
70
80
90
500
1000
1500
2000
2500
3000
4000
5000
22%
40%
20%
39%

CPCG0404

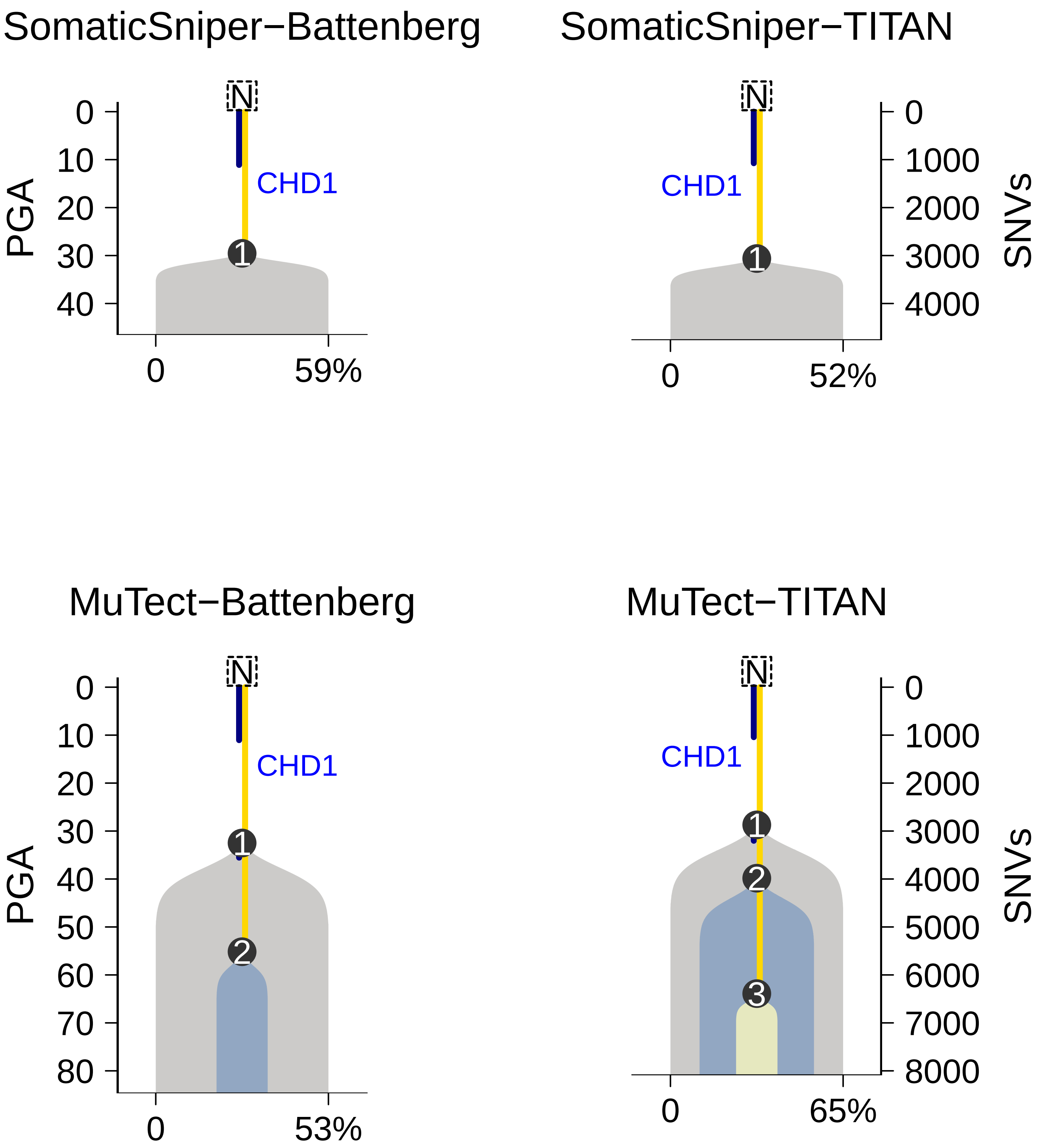

SomaticSniper−Battenberg
SomaticSniper−TITAN
N
PGA
0
10
20
30
40
CHD1
1
0
59%
0
1000
2000
3000
4000
SNVs
52%
MuTect−Battenberg
MuTect−TITAN
50
60
70
80
2
3
5000
6000
7000
8000
53%
65%

CPCG0407

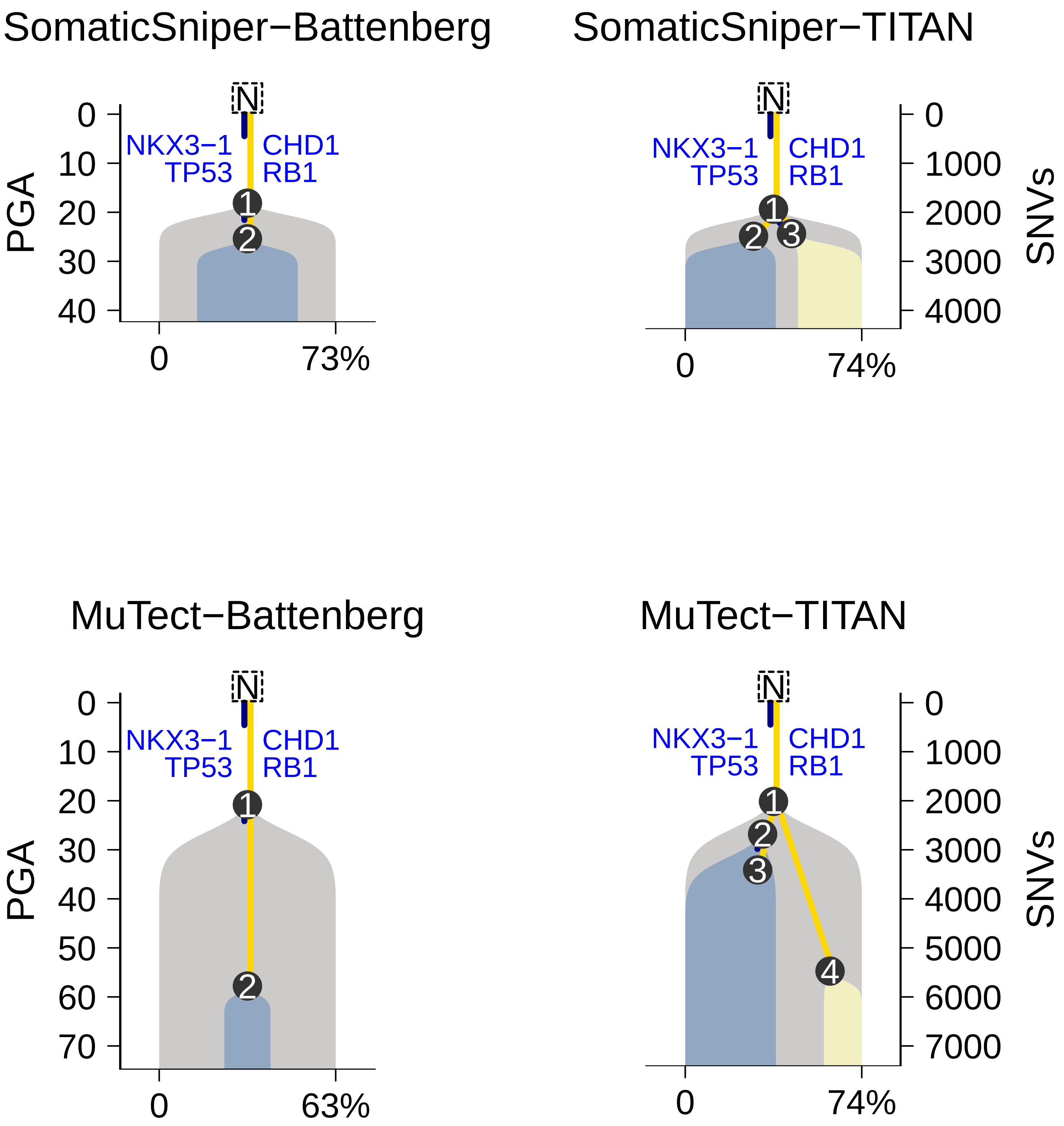

SomaticSniper−Battenberg
SomaticSniper−TITAN
MuTect−Battenberg
MuTect−TITAN
N
NKX3−1
TP53
CHD1
RB1
PGA
SNVs
0
10
20
30
40
50
60
70
1000
2000
3000
4000
5000
6000
7000
73%
74%
63%
1
2
3
4

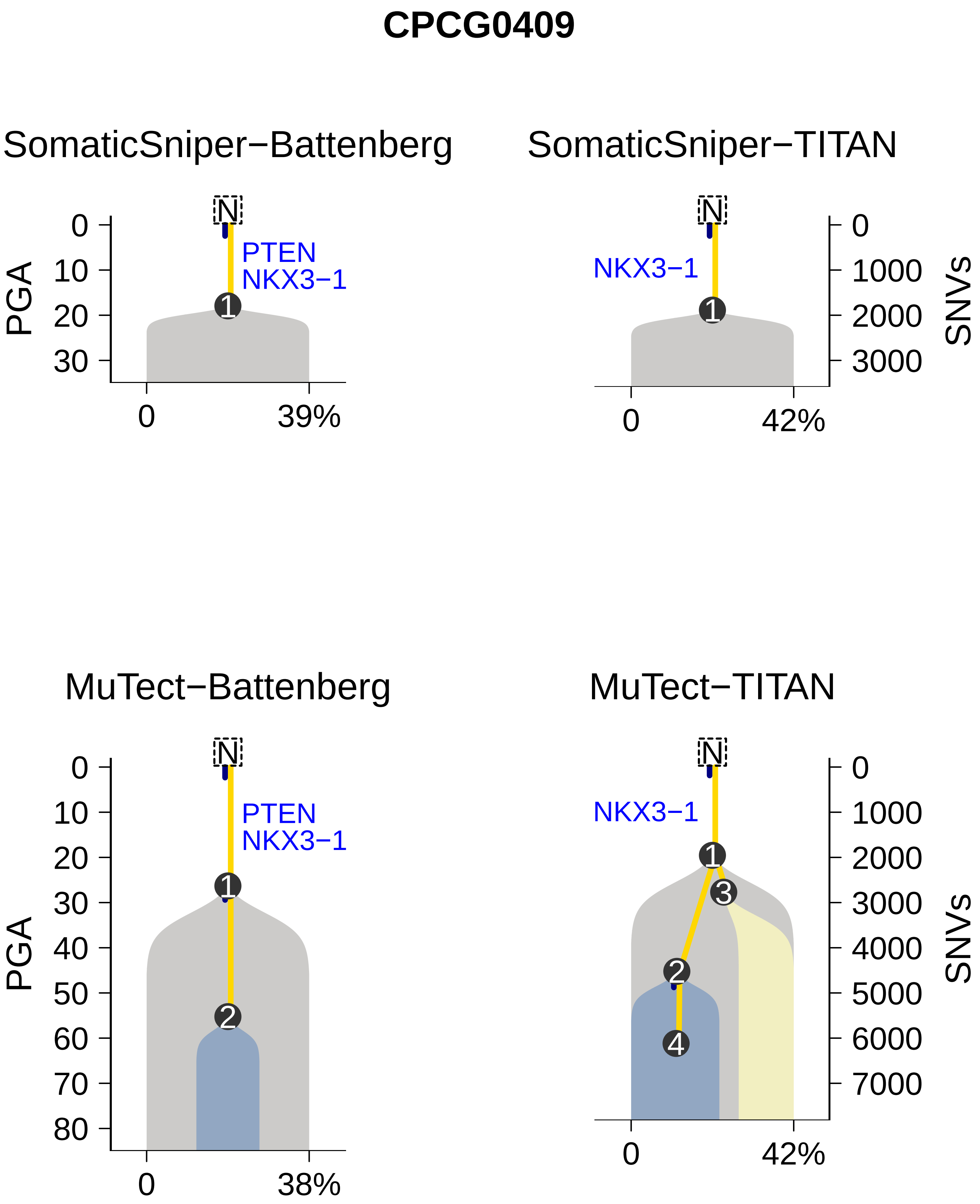

CPCG0409
SomaticSniper−Battenberg
SomaticSniper−TITAN
MuTect−Battenberg
MuTect−TITAN
N
PTEN
NKX3−1
PGA
SNVs
0
10
20
30
40
50
60
70
80
0
1000
2000
3000
4000
5000
6000
7000
39%
42%
38%
1
2
3
4

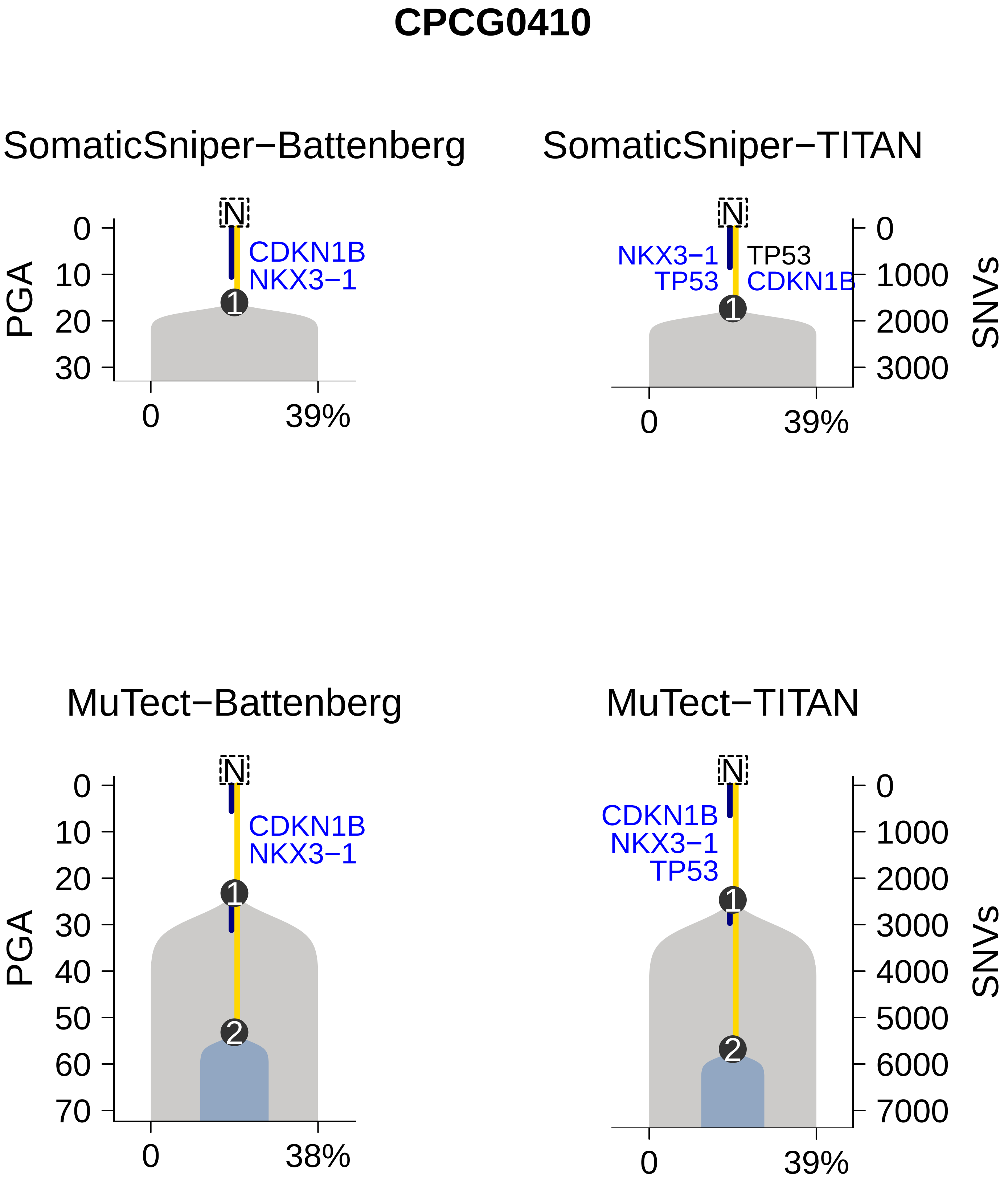

CPCG0410
SomaticSniper−Battenberg
N
CDKN1B
NKX3−1
1
PGA
0
10
20
30
0
39%
SomaticSniper−TITAN
N
NKX3−1
TP53
TP53
CDKN1B
1
0
1000
2000
3000
SNVs
0
39%
MuTect−Battenberg
N
CDKN1B
NKX3−1
1
2
PGA
0
10
20
30
40
50
60
70
0
38%
MuTect−TITAN
N
CDKN1B
NKX3−1
TP53
1
2
0
1000
2000
3000
4000
5000
6000
7000
SNVs
0
39%

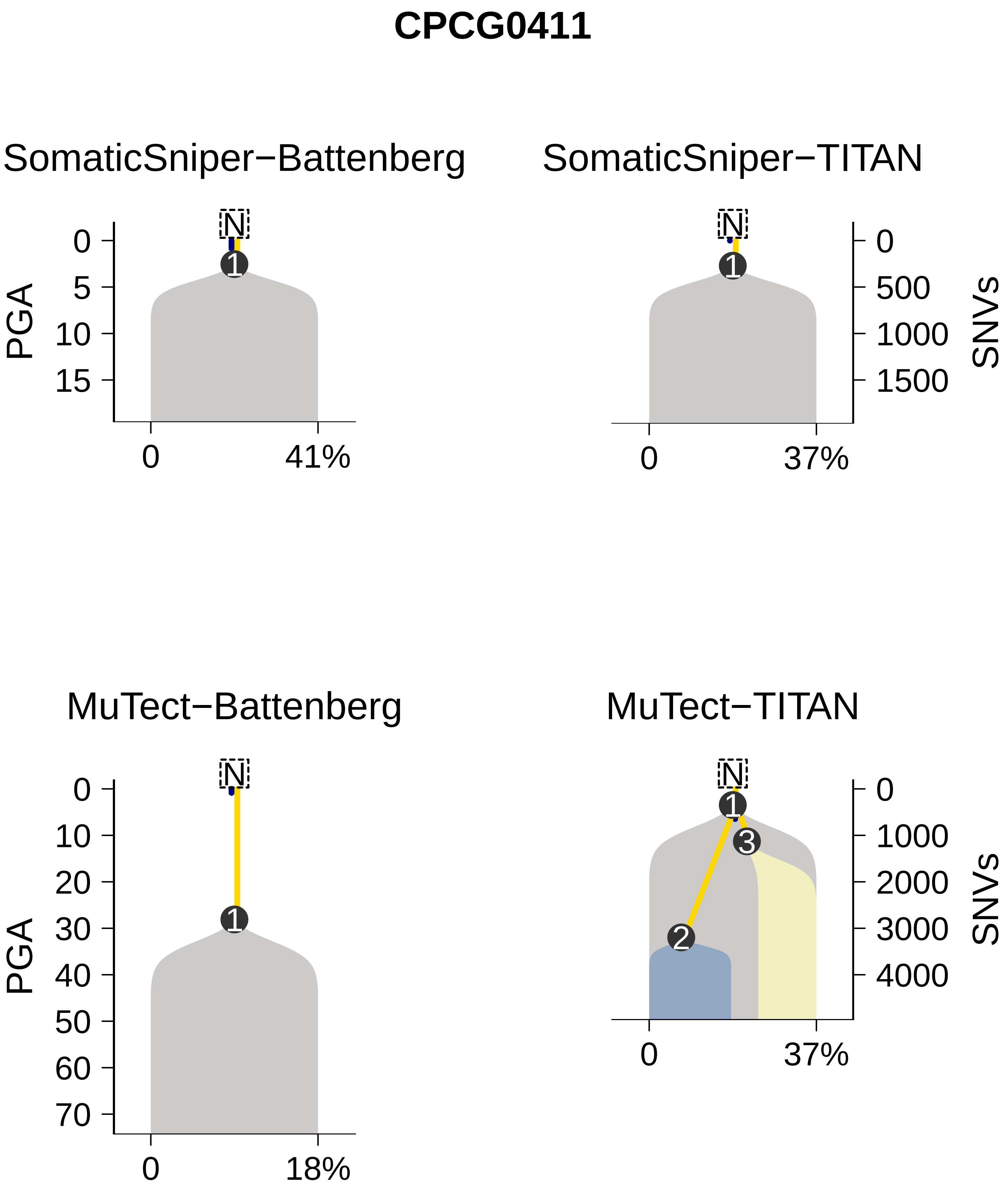
CPCG0411
SomaticSniper−Battenberg
SomaticSniper−TITAN
MuTect−Battenberg
MuTect−TITAN
N
1
2
3
PGA
SNVs
0
5
10
15
20
30
40
50
60
70
500
1000
1500
2000
3000
4000
41%
37%
18%

CPCG0412

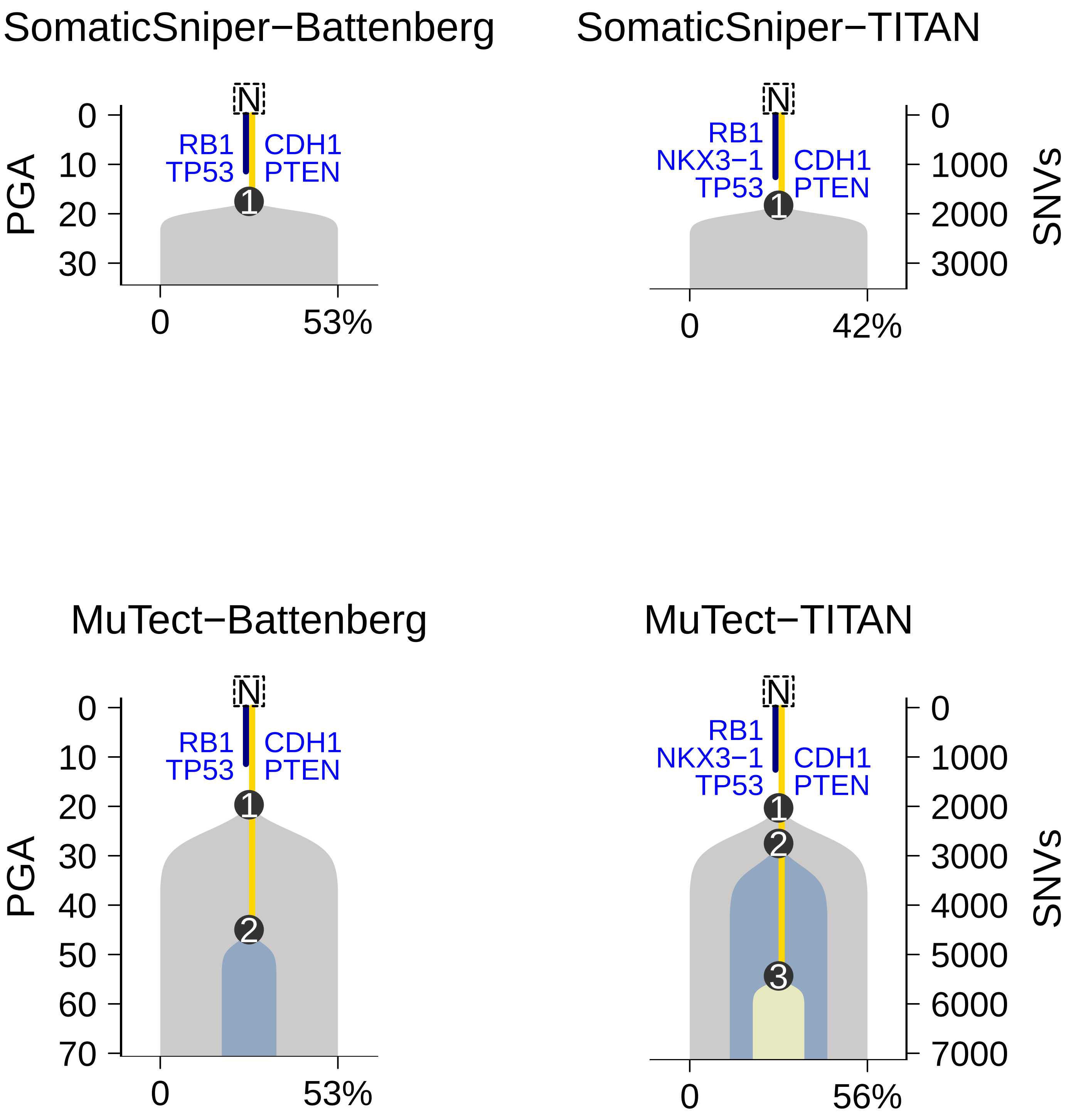

SomaticSniper−Battenberg
SomaticSniper−TITAN
N
RB1
TP53
CDH1
PTEN
NKX3−1
1
2
3
PGA
SNVs
0
10
20
30
40
50
60
70
1000
2000
3000
4000
5000
6000
7000
53%
42%
56%
MuTect−Battenberg
MuTect−TITAN

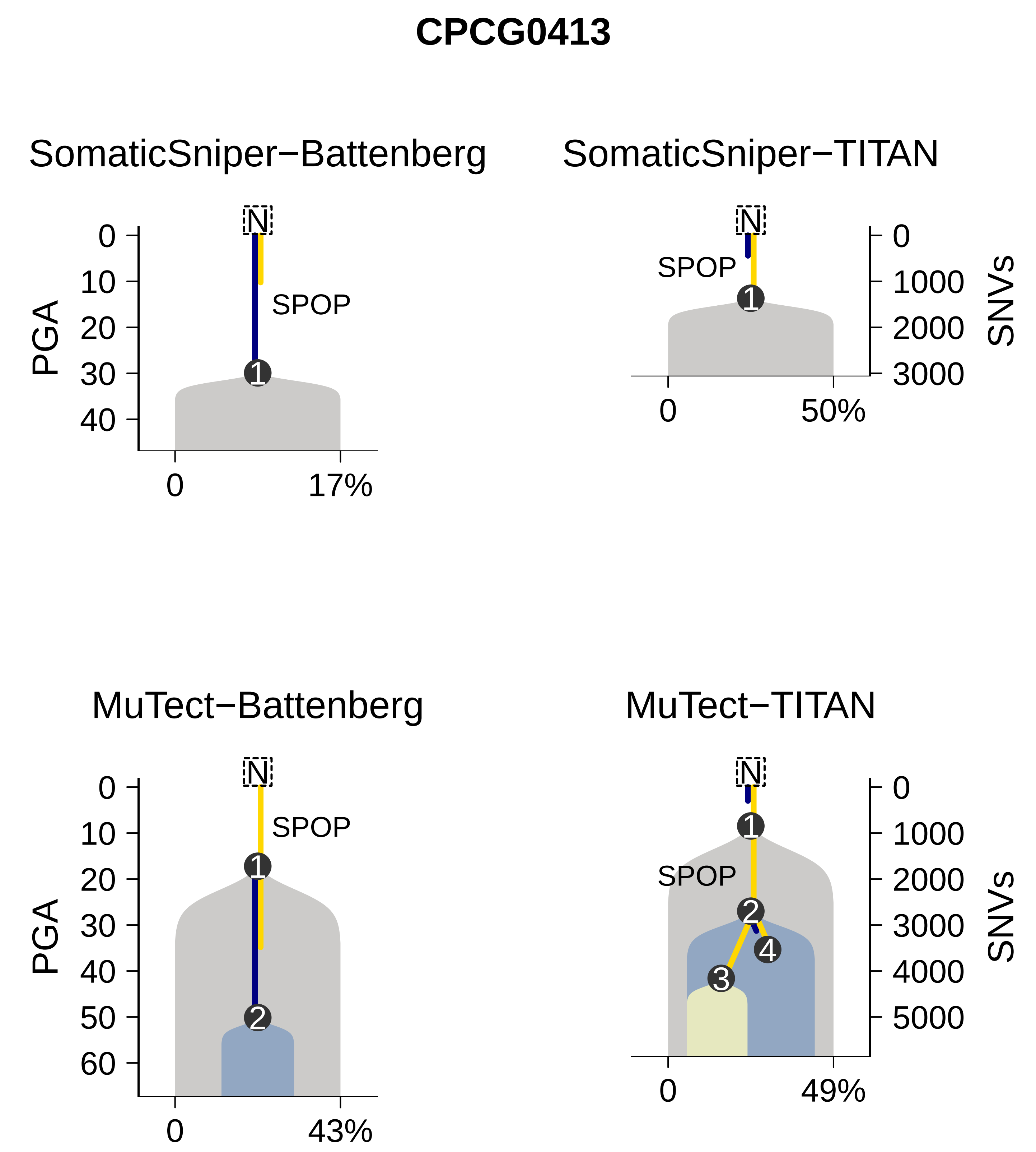
CPCG0413
SomaticSniper−Battenberg
N
SPOP
1
PGA
0
10
20
30
40
0
17%
SomaticSniper−TITAN
N
SPOP
1
0
1000
2000
3000
SNVs
0
50%
MuTect−Battenberg
N
SPOP
1
2
PGA
0
10
20
30
40
50
60
0
43%
MuTect−TITAN
N
1
SPOP
2
3
4
0
1000
2000
3000
4000
5000
SNVs
0
49%

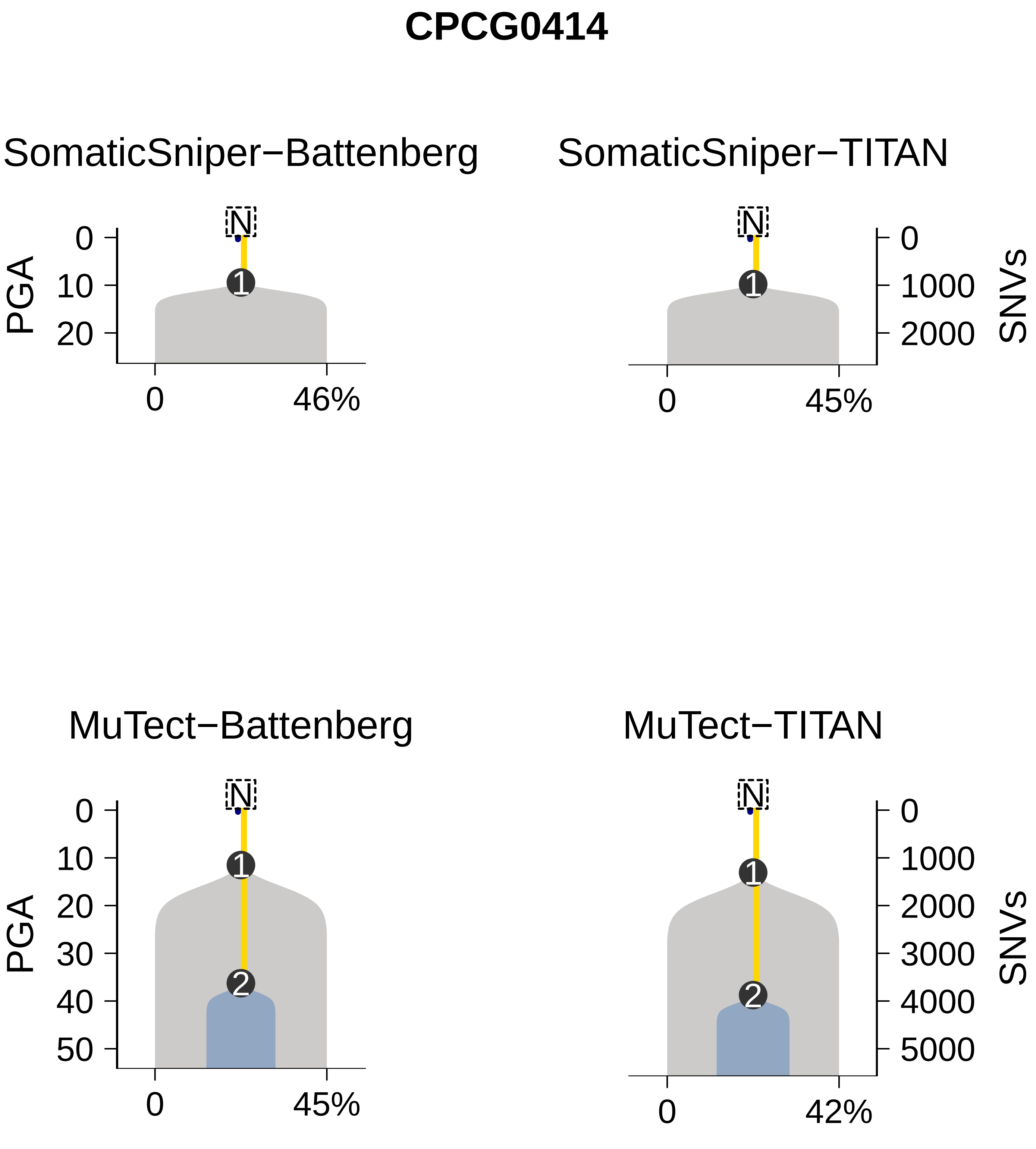
CPCG0414
SomaticSniper−Battenberg
SomaticSniper−TITAN
N
1
PGA
0
10
20
0
46%
0
1000
2000
SNVs
0
45%
MuTect−Battenberg
MuTect−TITAN
2
30
40
50
45%
3000
4000
5000
42%

CPCG0424

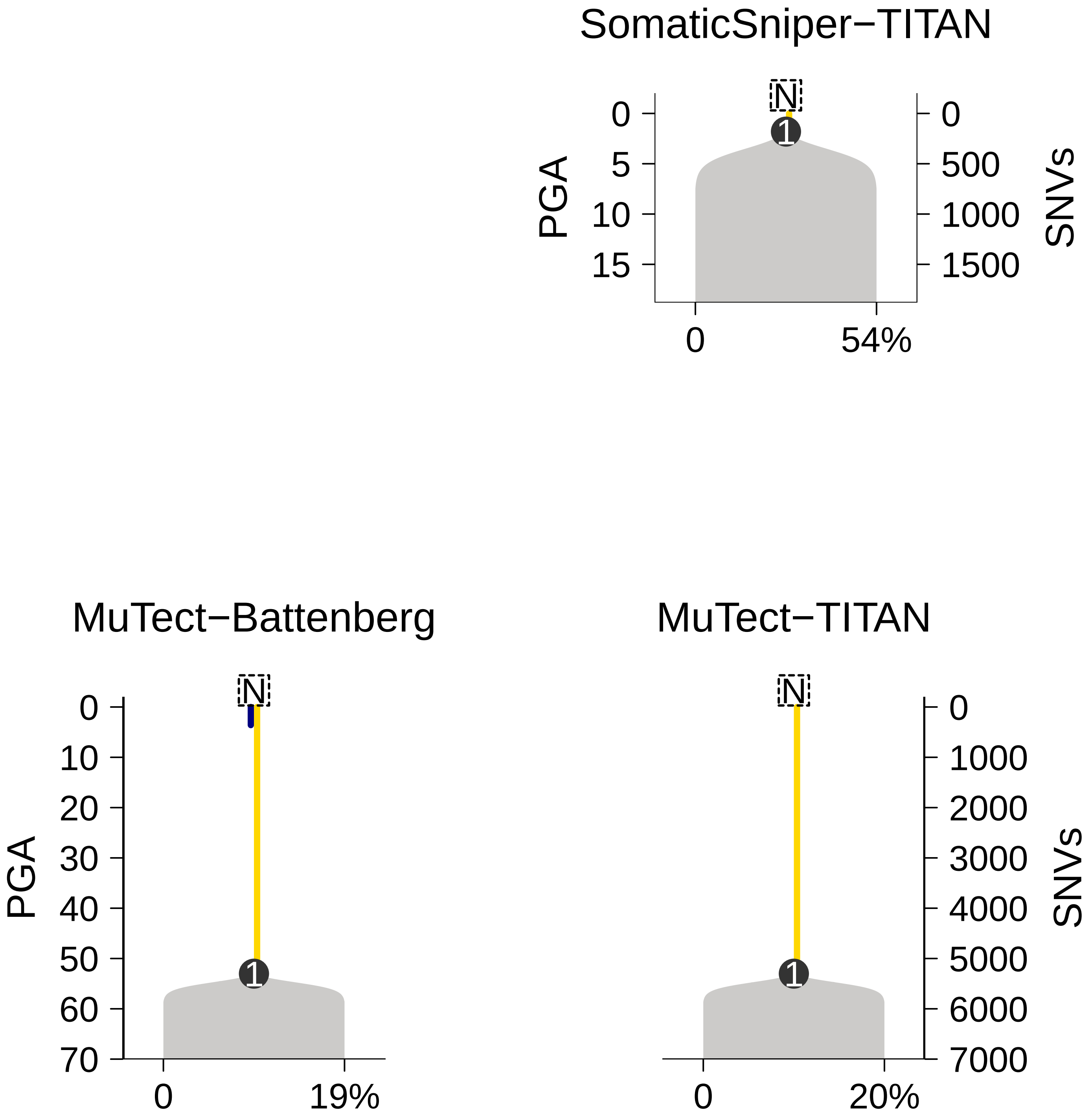

SomaticSniper−TITAN
N
1
PGA
0
5
10
15
0
500
1000
1500
SNVs
0
54%
MuTect−Battenberg
N
1
PGA
0
10
20
30
40
50
60
70
0
19%
MuTect−TITAN
N
1
0
1000
2000
3000
4000
5000
6000
7000
SNVs
0
20%

CPCG0432

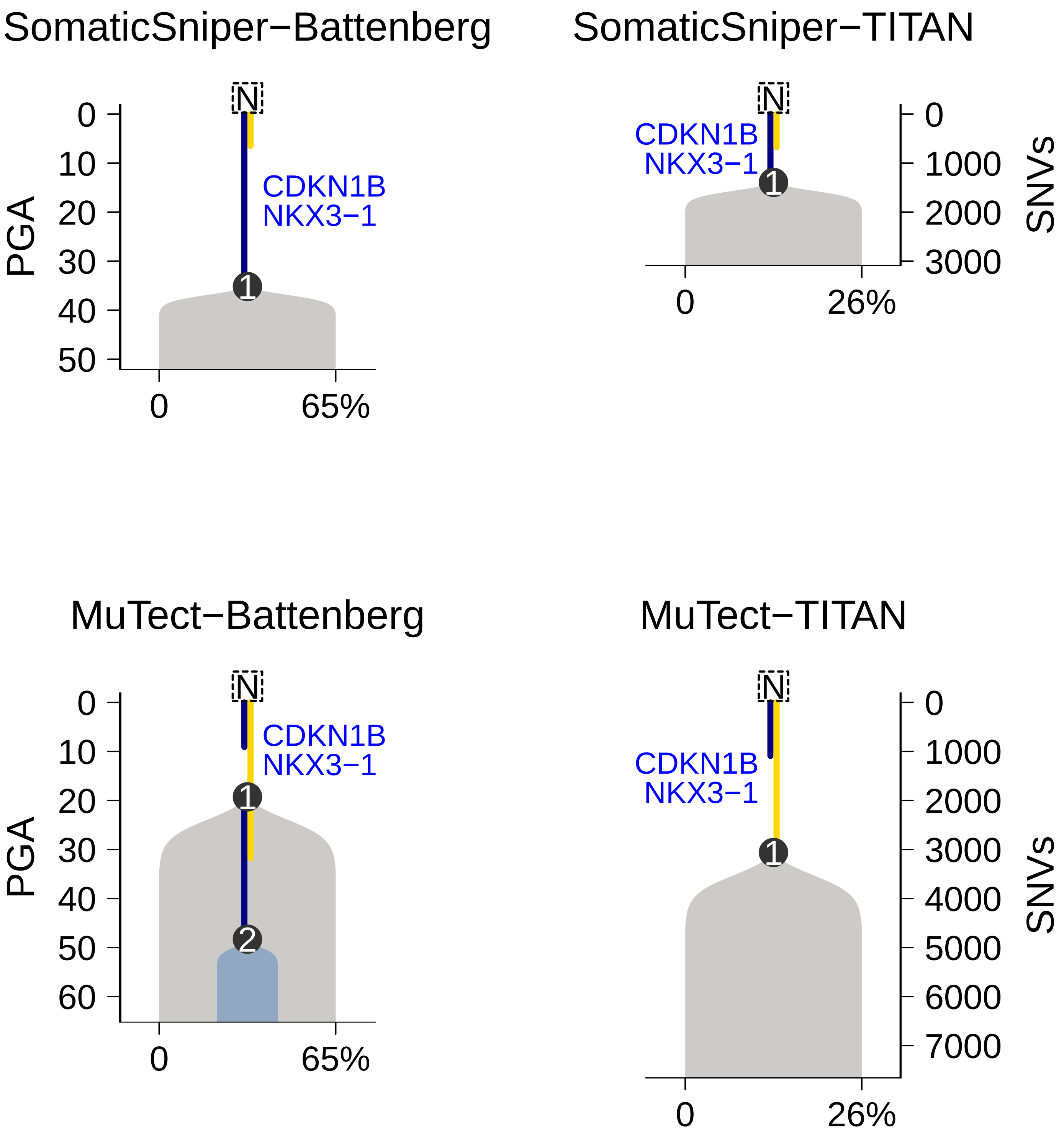

SomaticSniper−Battenberg
N
CDKN1B
NKX3−1
1
PGA
0
10
20
30
40
50
0
65%
SomaticSniper−TITAN
N
CDKN1B
NKX3−1
1
0
1000
2000
3000
SNVs
0
26%
MuTect−Battenberg
N
CDKN1B
NKX3−1
1
2
PGA
0
10
20
30
40
50
60
0
65%
MuTect−TITAN
N
CDKN1B
NKX3−1
1
0
1000
2000
3000
4000
5000
6000
7000
SNVs
0
26%

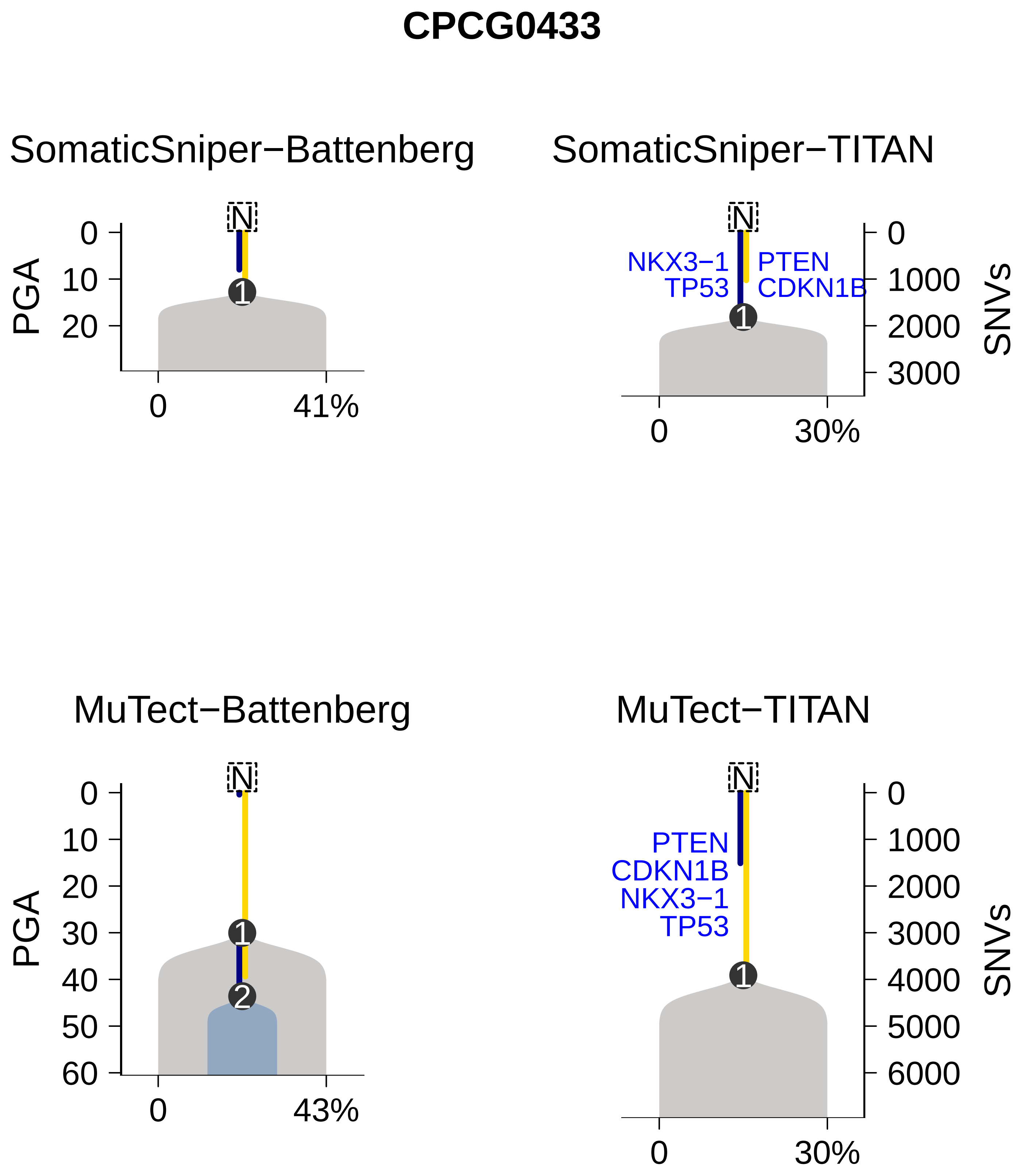
CPCG0433
SomaticSniper−Battenberg
SomaticSniper−TITAN
MuTect−Battenberg
MuTect−TITAN
N
1
2
PGA
SNVs
0
10
20
30
40
50
60
1000
2000
3000
4000
5000
6000
41%
43%
30%
NKX3−1
TP53
PTEN
CDKN1B

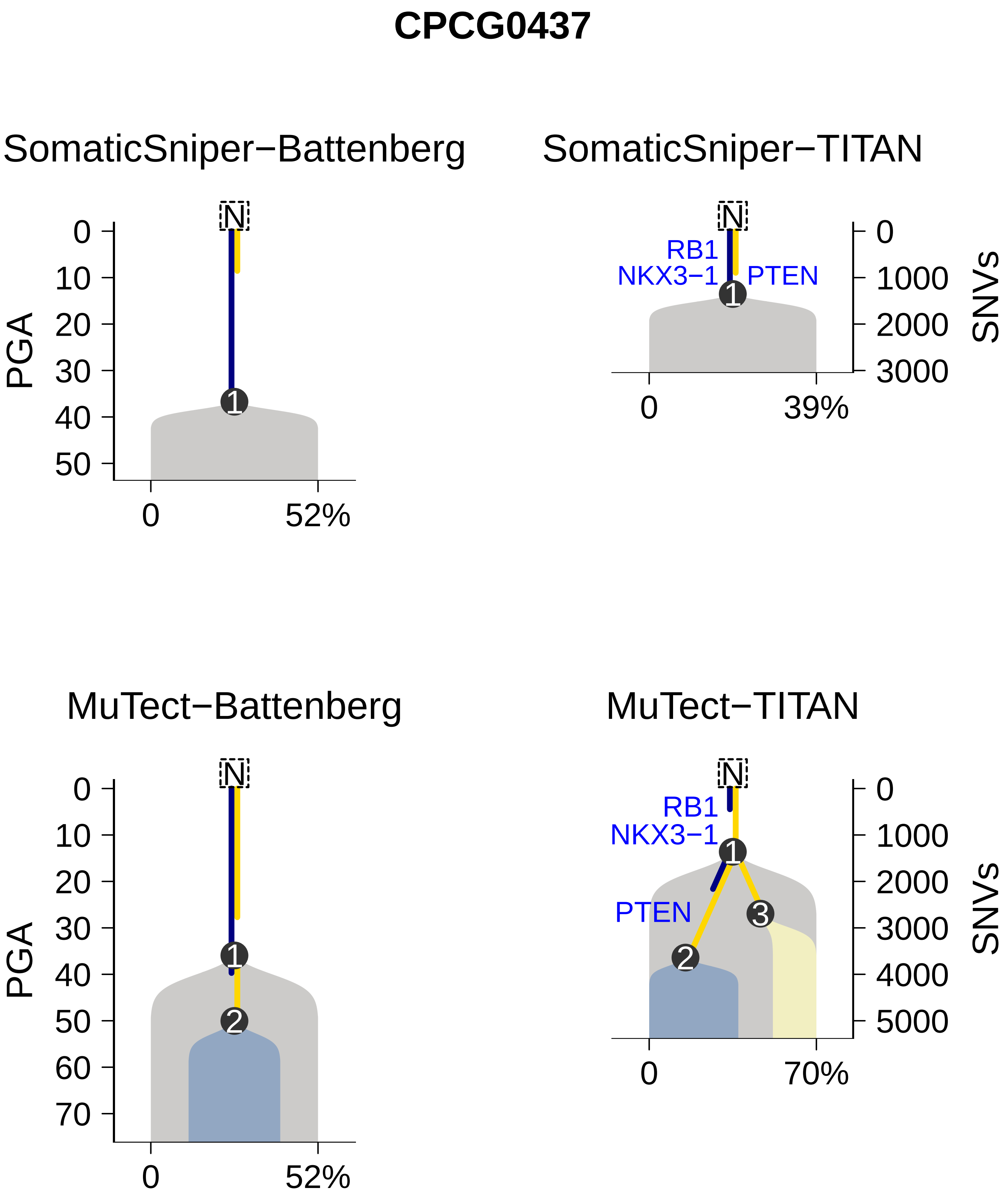
CPCG0437
SomaticSniper−Battenberg
SomaticSniper−TITAN
MuTect−Battenberg
MuTect−TITAN
N
1
2
3
RB1
NKX3−1
PTEN
PGA
SNVs
0
10
20
30
40
50
60
70
1000
2000
3000
4000
5000
52%
39%
70%

CPCG0450

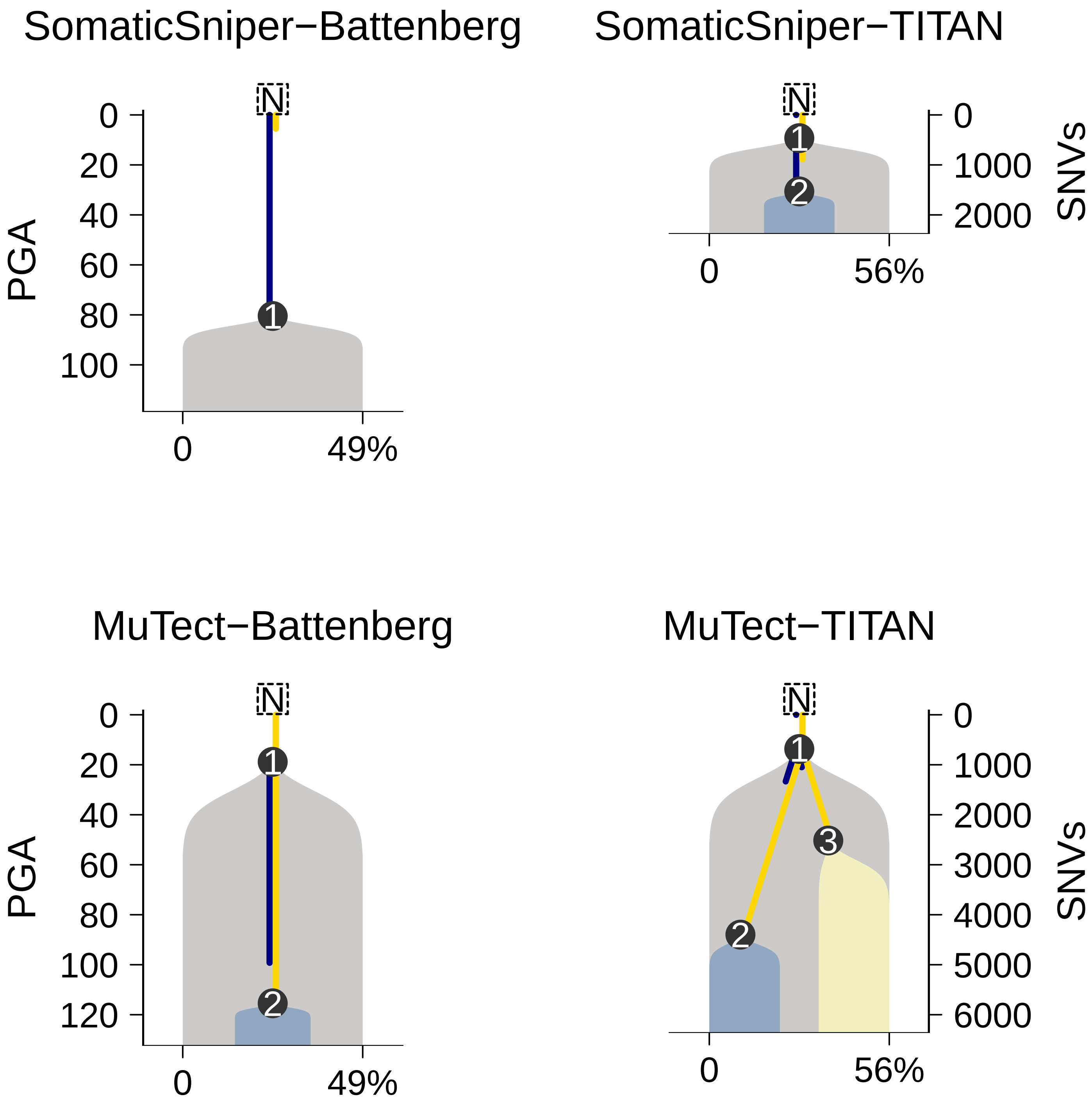

SomaticSniper−Battenberg
SomaticSniper−TITAN
MuTect−Battenberg
MuTect−TITAN
PGA
SNVs
N
0
20
40
60
80
100
120
49%
56%
1000
2000
3000
4000
5000
6000
1
2
3

CPCG0451

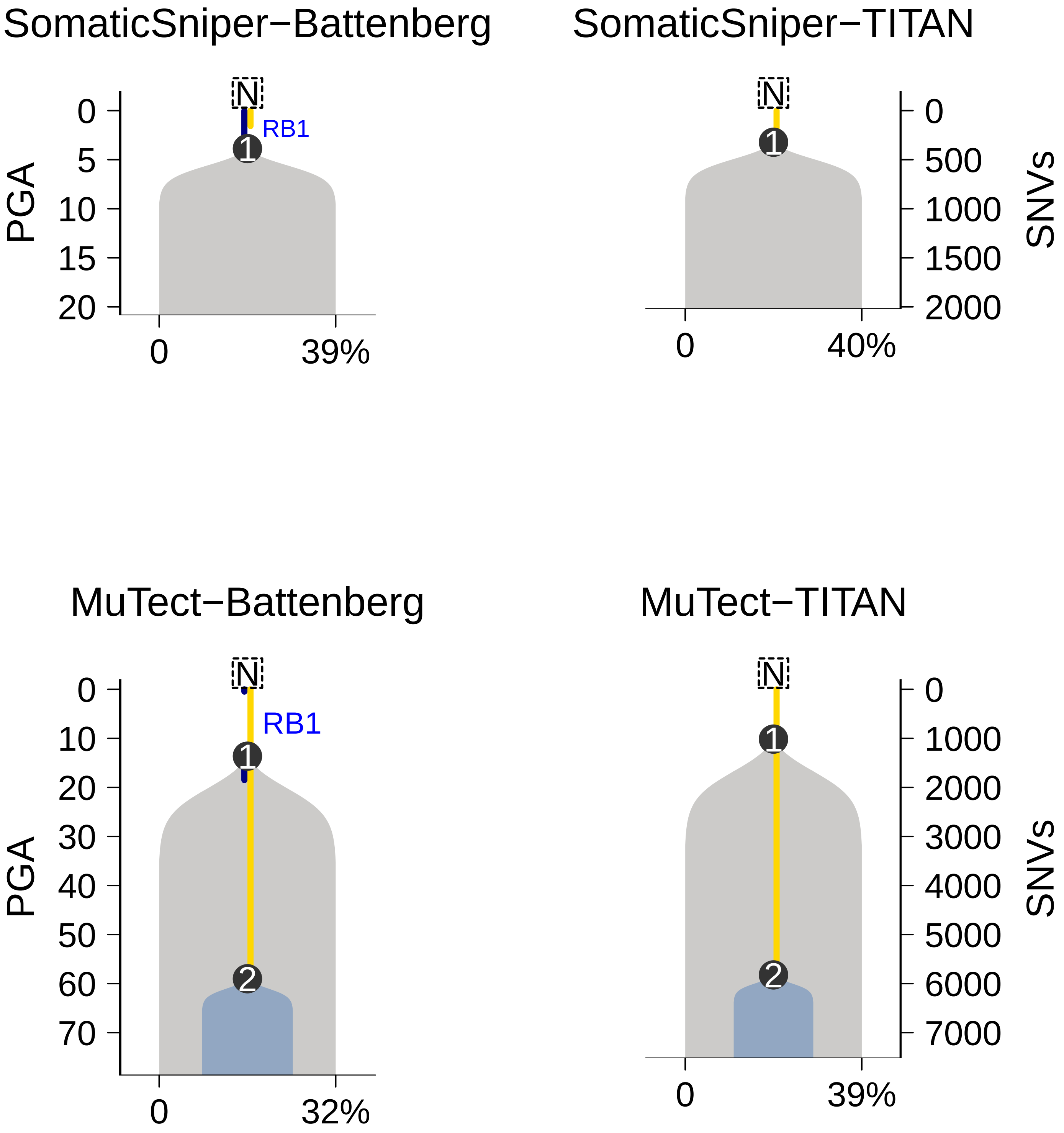
SomaticSniper−Battenberg
SomaticSniper−TITAN
MuTect−Battenberg
MuTect−TITAN
N
RB1
1
2
PGA
SNVs
0
5
10
15
20
30
40
50
60
70
500
1000
1500
2000
3000
4000
5000
6000
7000
39%
40%
32%

CPCG0452

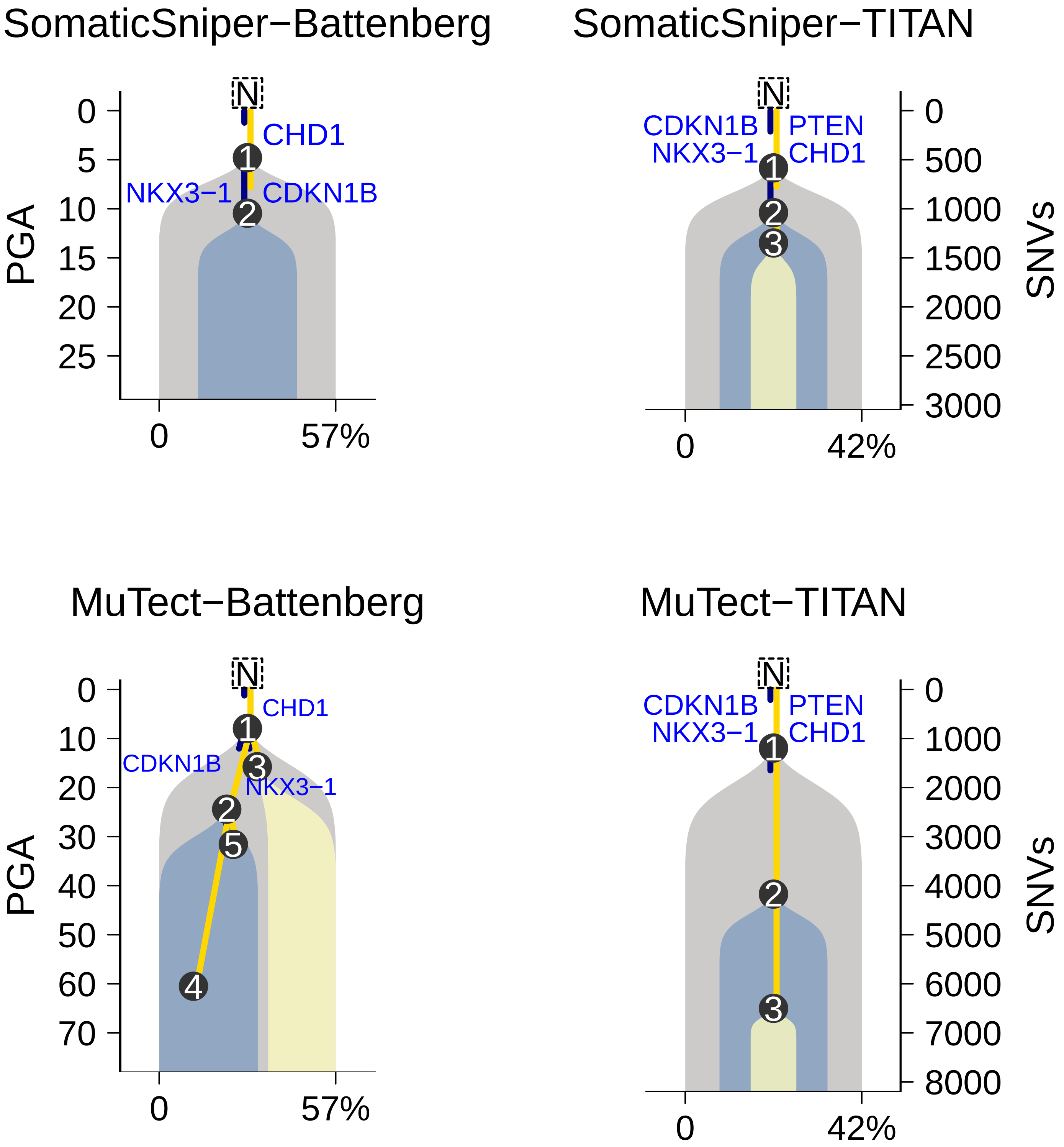
SomaticSniper−Battenberg
SomaticSniper−TITAN
MuTect−Battenberg
MuTect−TITAN
N
CHD1
NKX3−1
CDKN1B
PTEN
PGA
SNVs
0
57%
42%

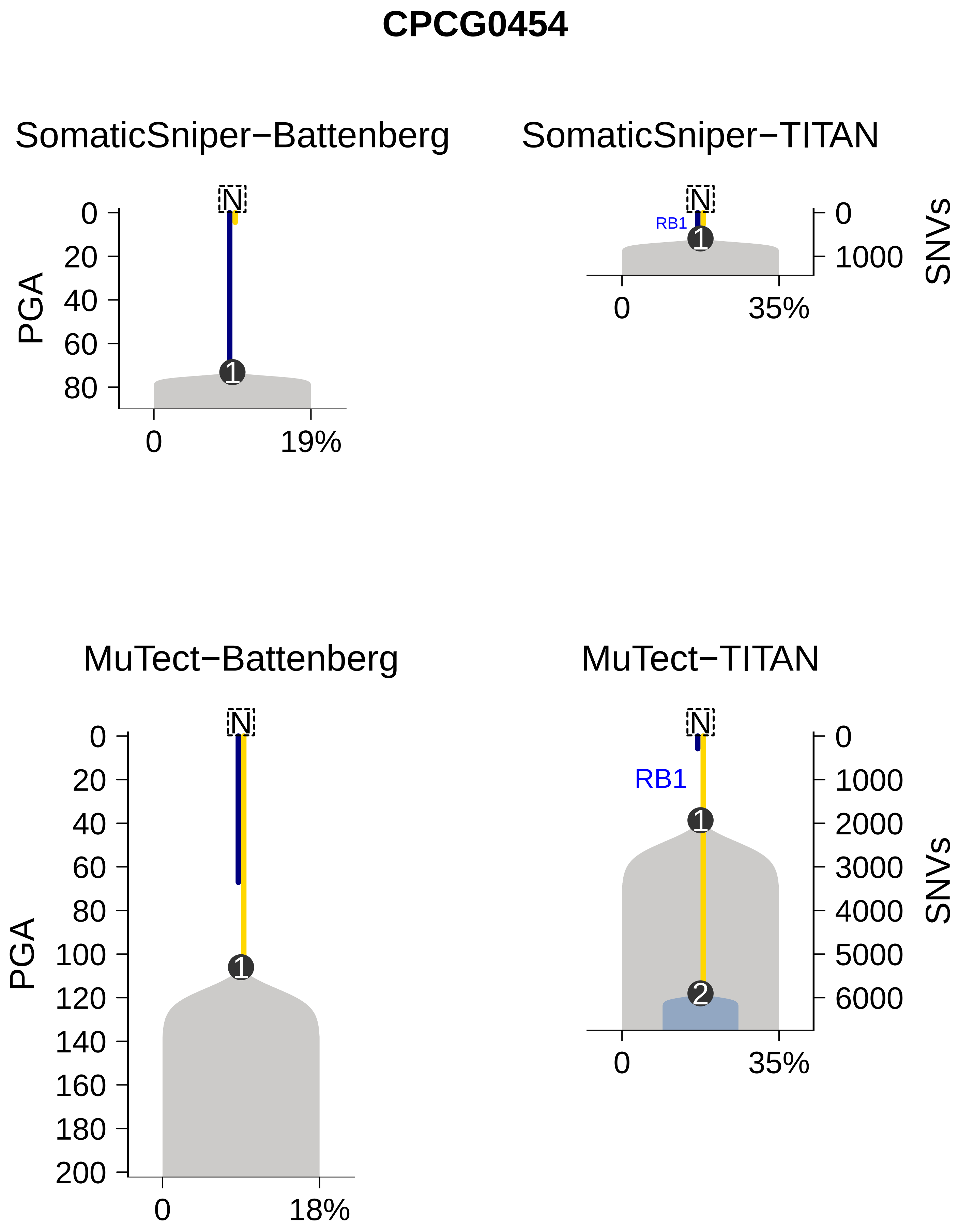

CPCG0454
SomaticSniper−Battenberg
SomaticSniper−TITAN
MuTect−Battenberg
MuTect−TITAN
N
1
2
RB1
PGA
SNVs
0
20
40
60
80
100
120
140
160
180
200
1000
2000
3000
4000
5000
6000
19%
35%
18%

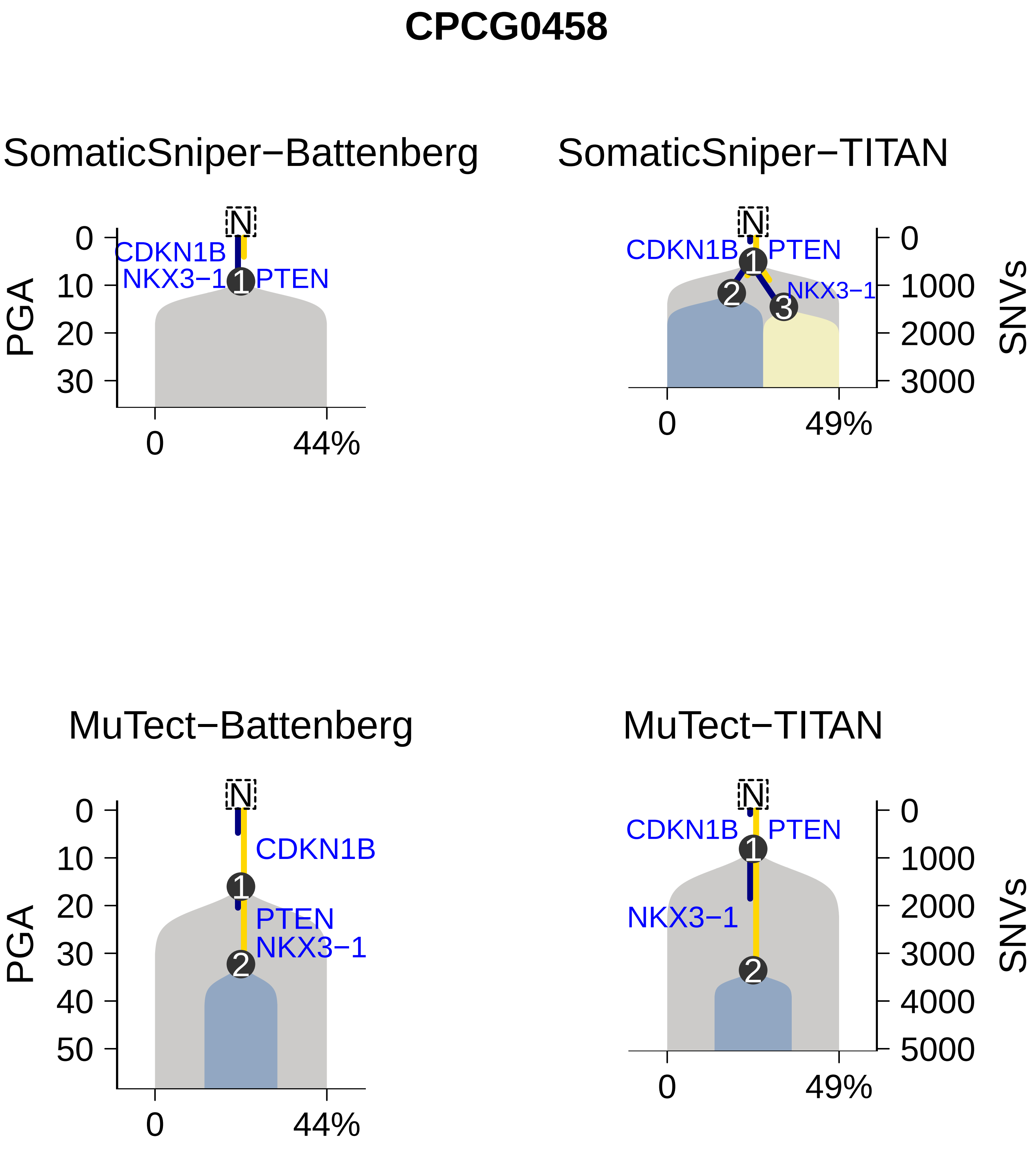
CPCG0458
SomaticSniper−Battenberg
N
CDKN1B
NKX3−1
1
PTEN
PGA
0
10
20
30
0
44%
SomaticSniper−TITAN
N
CDKN1B
PTEN
1
2
3
NKX3−1
0
1000
2000
3000
SNVs
0
49%
MuTect−Battenberg
N
CDKN1B
1
PTEN
NKX3−1
2
PGA
0
10
20
30
40
50
0
44%
MuTect−TITAN
N
CDKN1B
PTEN
1
NKX3−1
2
0
1000
2000
3000
4000
5000
SNVs
0
49%

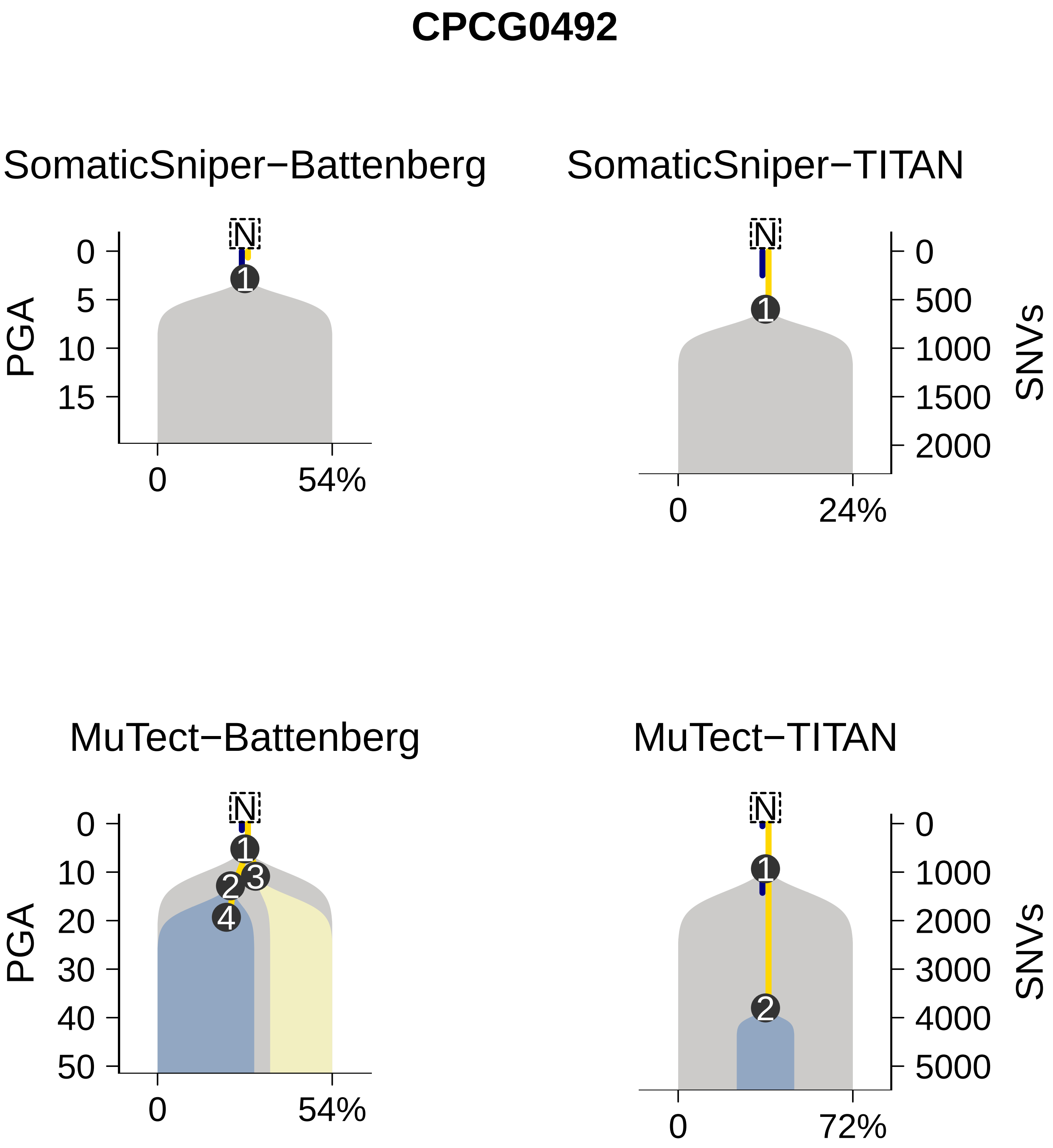
CPCG0492
SomaticSniper−Battenberg
SomaticSniper−TITAN
MuTect−Battenberg
MuTect−TITAN
N
1
2
3
4
PGA
SNVs
0
5
10
15
20
30
40
50
500
1000
1500
2000
3000
4000
5000
54%
24%
72%

CPCG0520

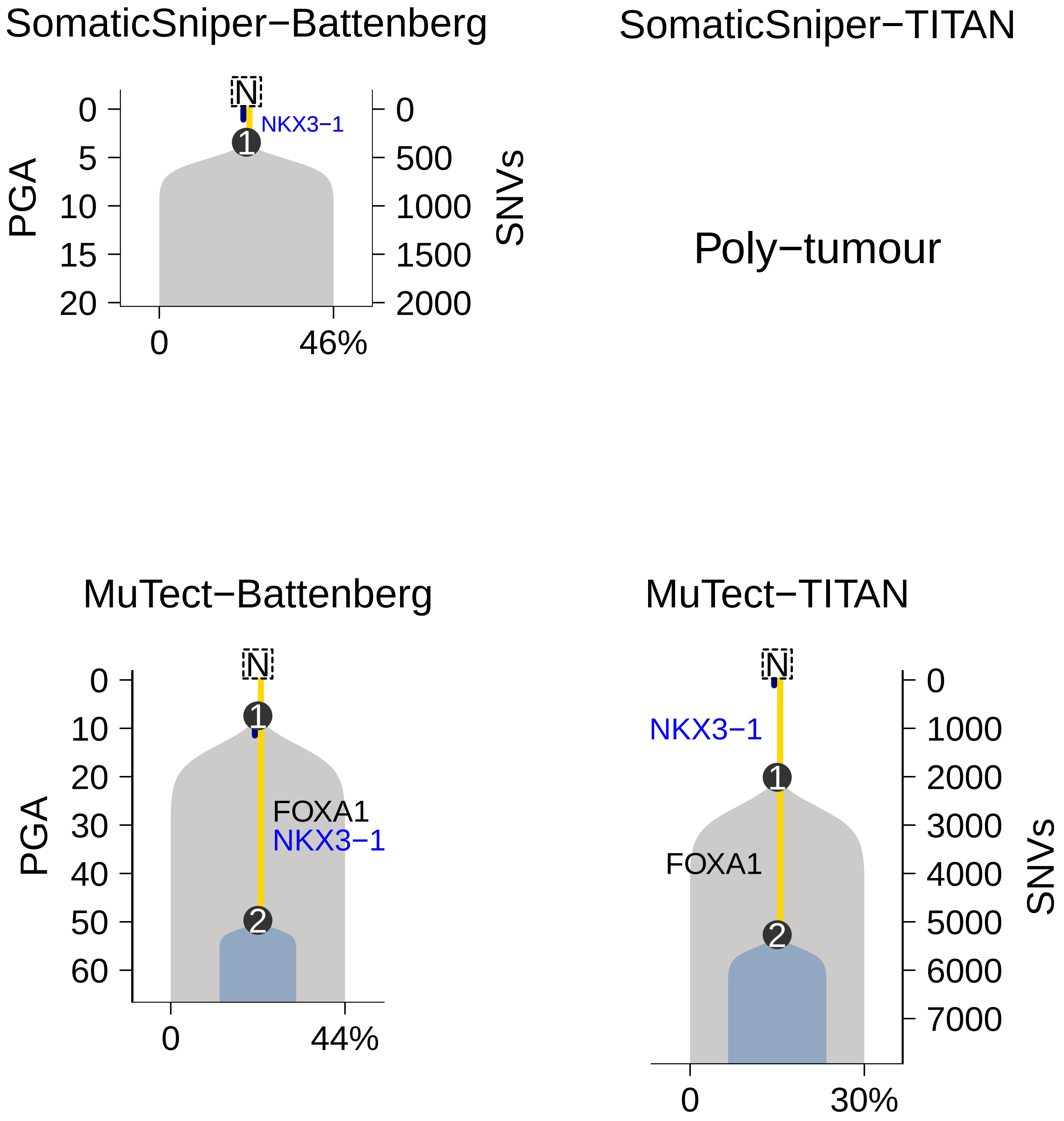
SomaticSniper−Battenberg
N
NKX3−1
1
PGA
0
5
10
15
20
SNVs
0
500
1000
1500
2000
0
46%
SomaticSniper−TITAN
Poly−tumour
MuTect−Battenberg
N
1
FOXA1
NKX3−1
2
PGA
0
10
20
30
40
50
60
0
44%
MuTect−TITAN
N
NKX3−1
1
FOXA1
2
SNVs
0
1000
2000
3000
4000
5000
6000
7000
0
30%

CPCG0525

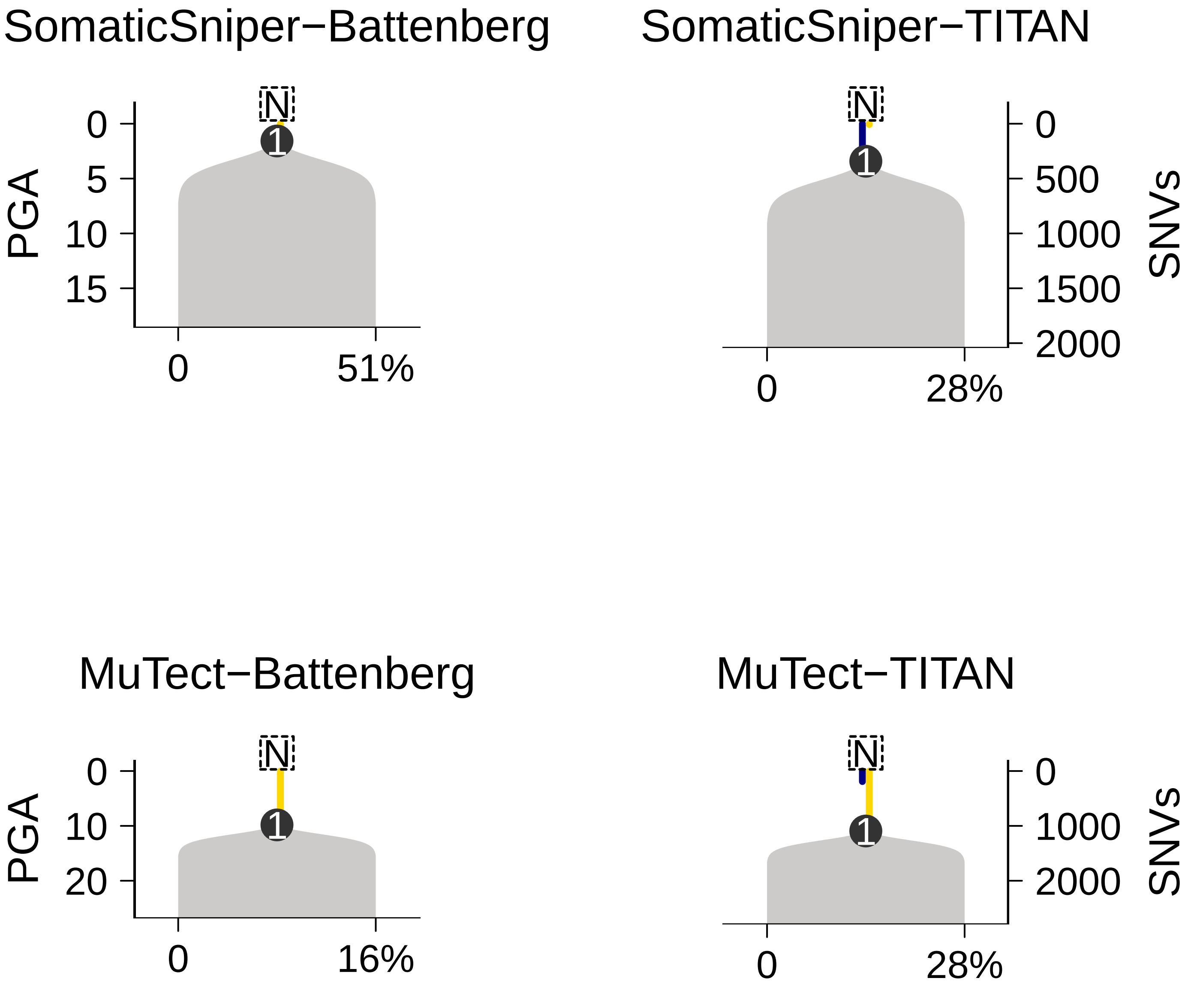

SomaticSniper−Battenberg
SomaticSniper−TITAN
N
1
PGA
0
5
10
15
0
51%
0
500
1000
1500
2000
SNVs
0
28%
MuTect−Battenberg
MuTect−TITAN
0
10
20
0
16%
0
1000
2000
0
28%

CPCG0528

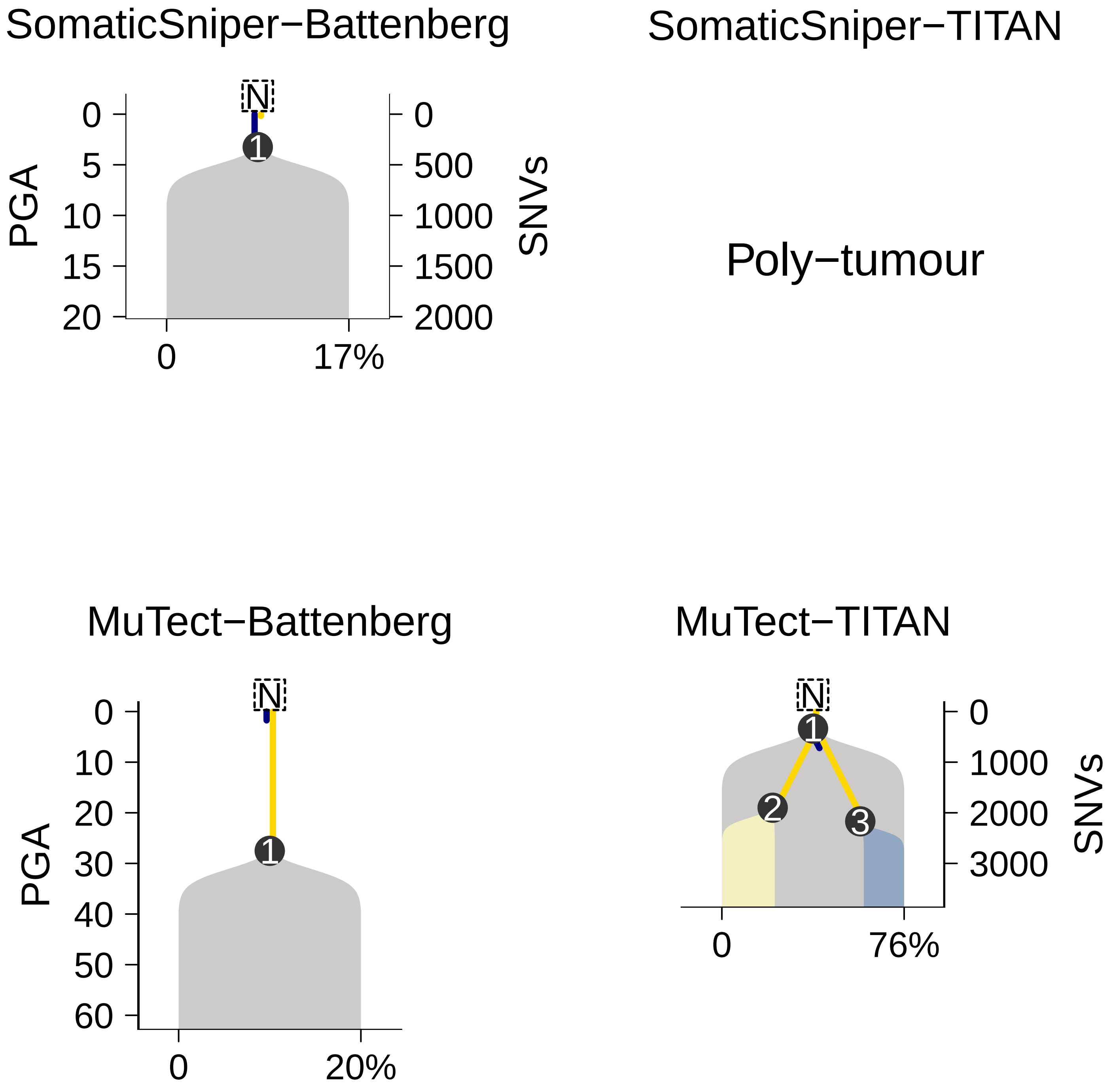
SomaticSniper−Battenberg
SomaticSniper−TITAN
N
1
PGA
0
5
10
15
20
SNVs
0
500
1000
1500
2000
0
17%
Poly−tumour
MuTect−Battenberg
MuTect−TITAN
N
1
PGA
0
10
20
30
40
50
60
0
20%
N
1
2
3
0
1000
2000
3000
SNVs
0
76%

CPCG0529

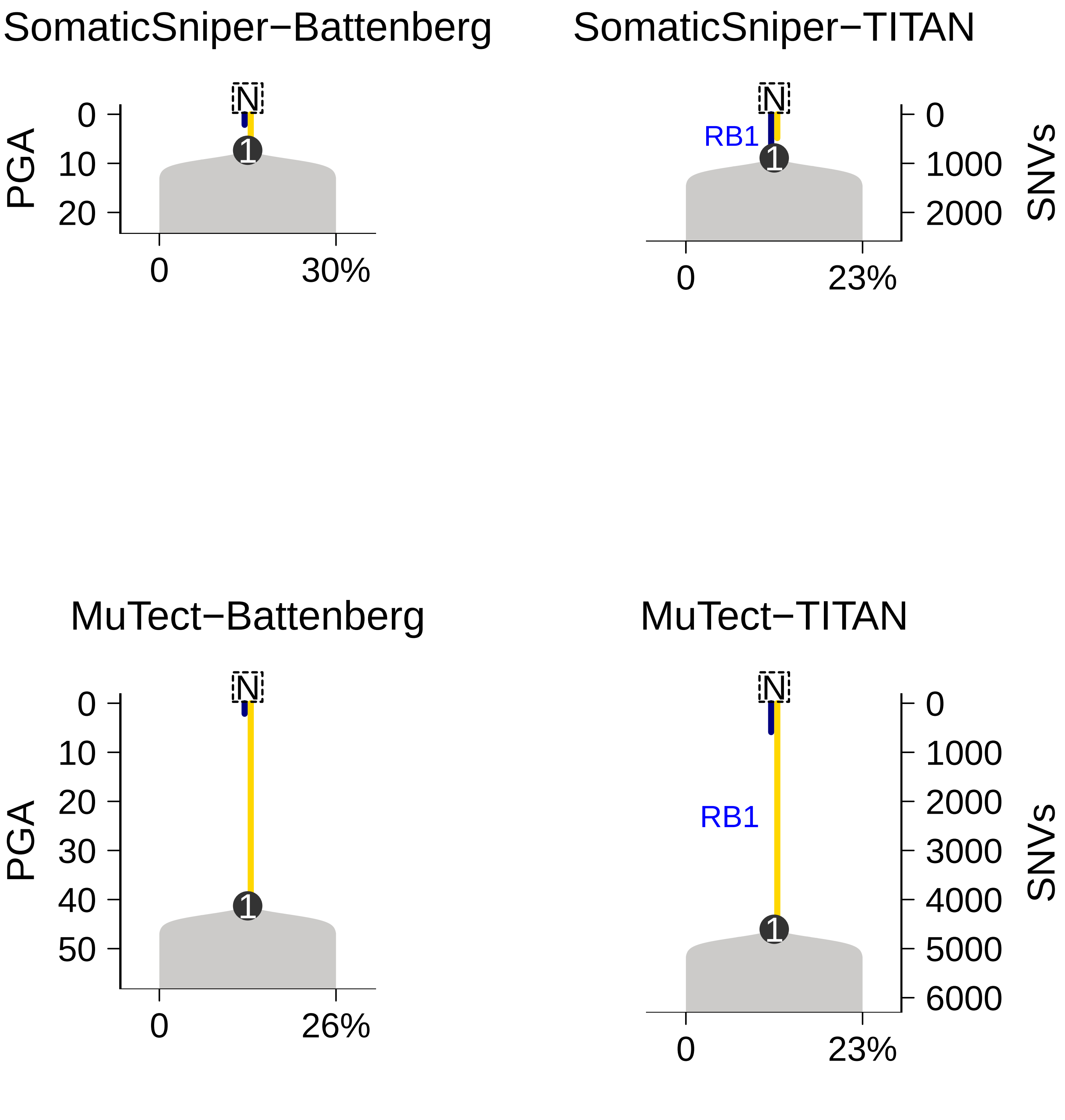

SomaticSniper−Battenberg
N
1
PGA
0
10
20
0
30%
SomaticSniper−TITAN
N
RB1
1
0
1000
2000
SNVs
0
23%
MuTect−Battenberg
N
1
PGA
0
10
20
30
40
50
0
26%
MuTect−TITAN
N
RB1
1
0
1000
2000
3000
4000
5000
6000
SNVs
0
23%

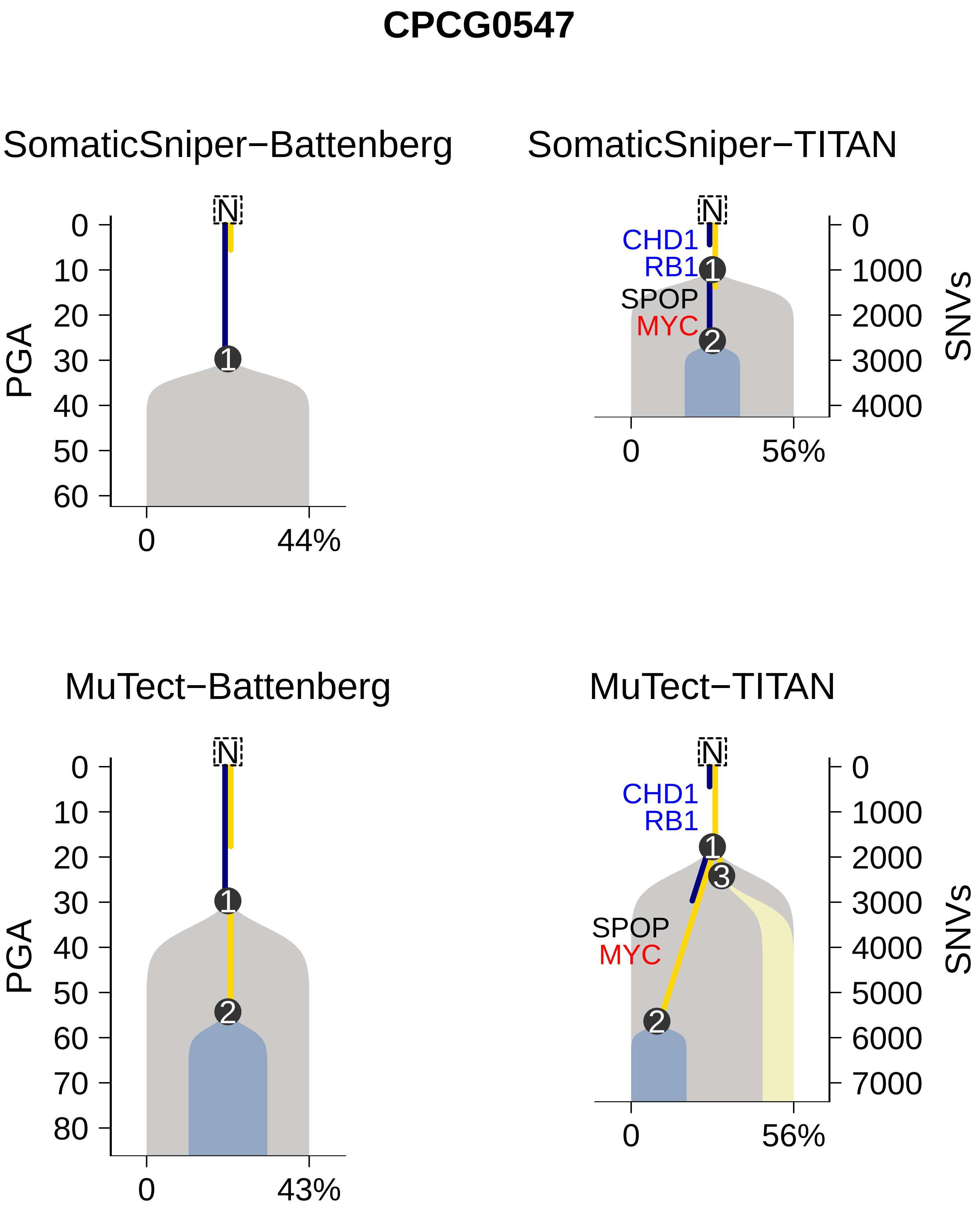
CPCG0547
SomaticSniper−Battenberg
SomaticSniper−TITAN
MuTect−Battenberg
MuTect−TITAN
N
1
2
3
CHD1
RB1
SPOP
MYC
PGA
SNVs
0
10
20
30
40
50
60
70
80
1000
2000
3000
4000
5000
6000
7000
44%
56%
43%

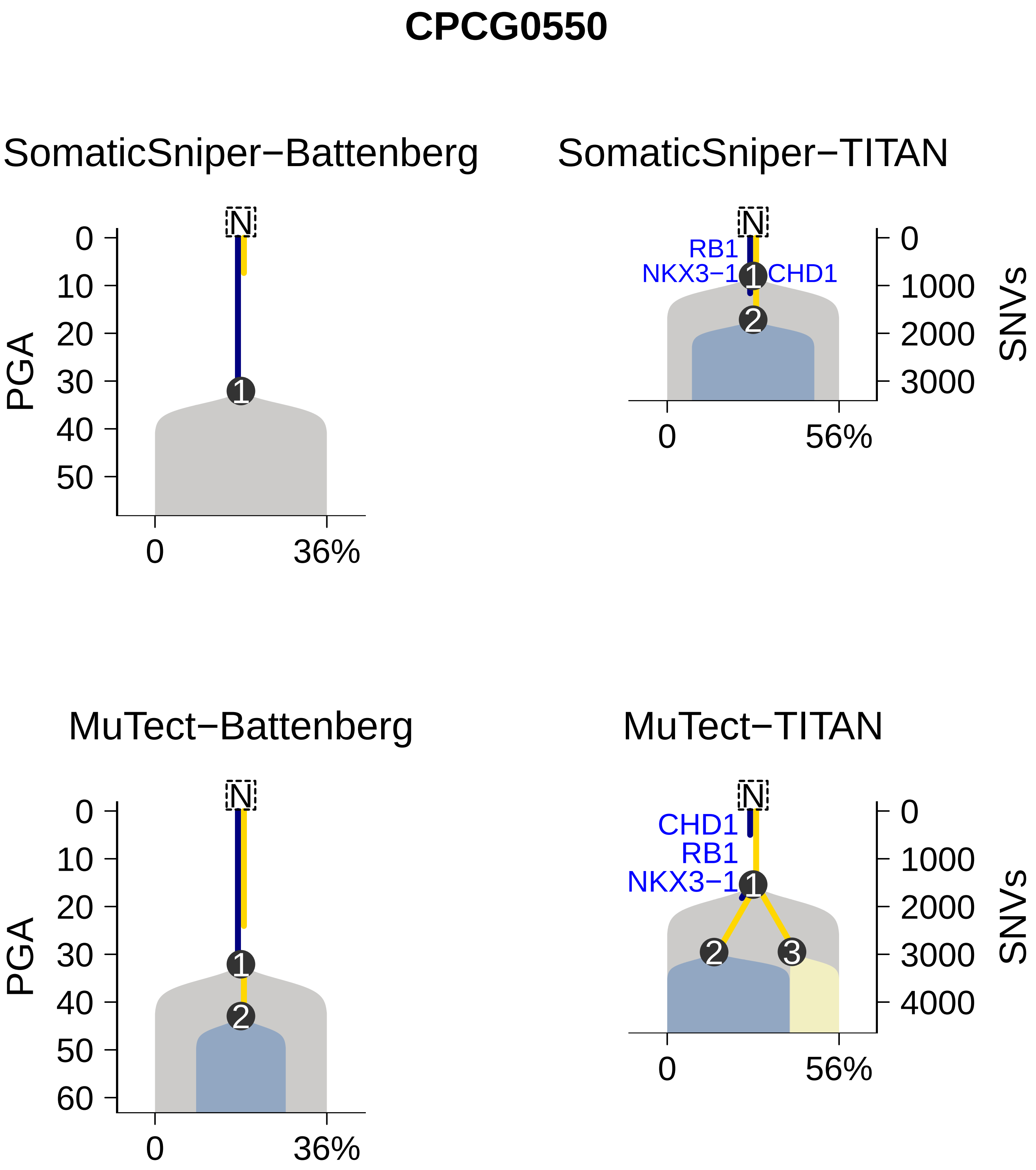
CPCG0550
SomaticSniper−Battenberg
SomaticSniper−TITAN
MuTect−Battenberg
MuTect−TITAN
N
1
2
3
PGA
SNVs
0
10
20
30
40
50
60
1000
2000
3000
4000
36%
56%
RB1
NKX3−1
CHD1

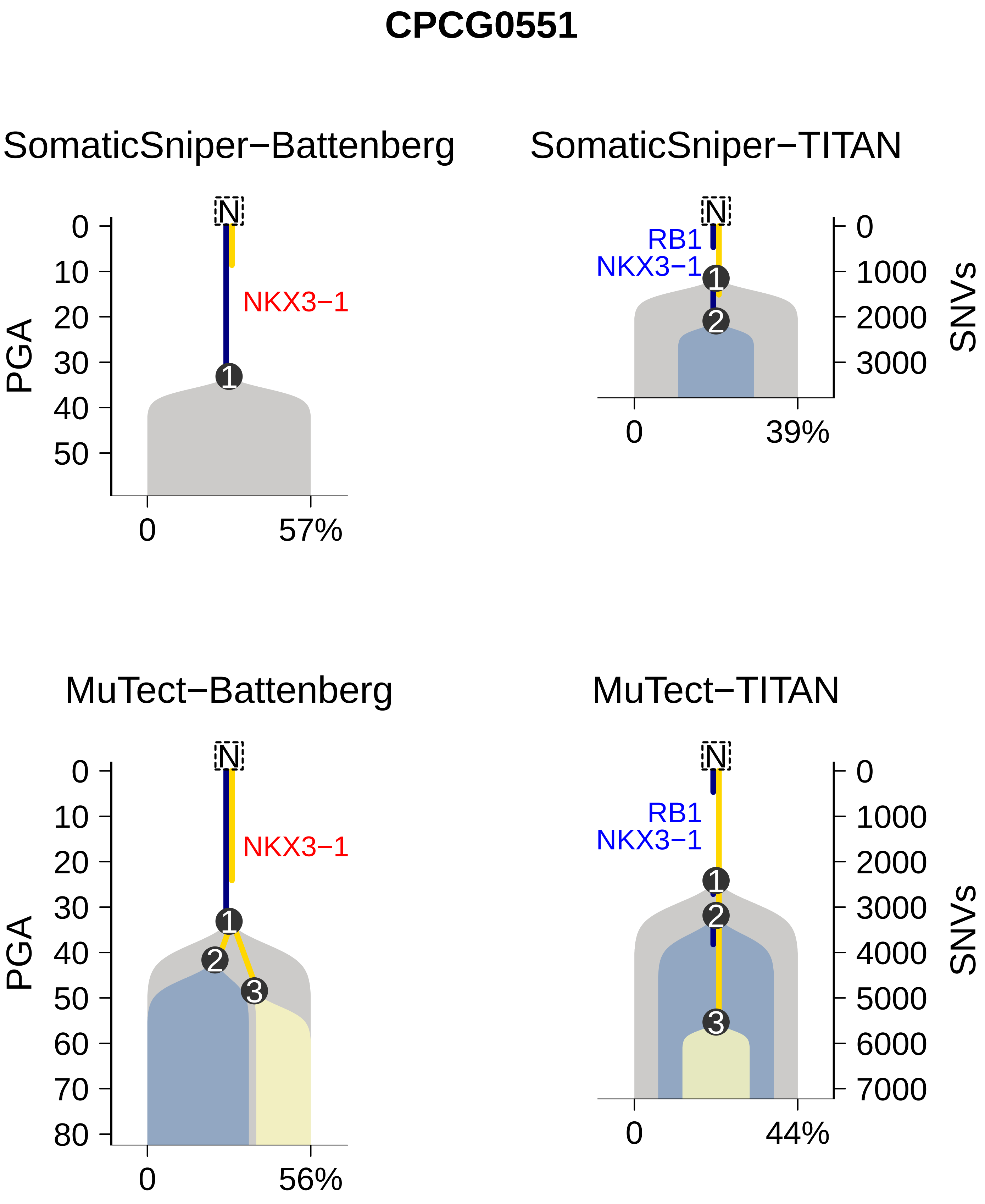
CPCG0551
SomaticSniper−Battenberg
N
NKX3−1
1
PGA
0
10
20
30
40
50
0
57%
SomaticSniper−TITAN
N
RB1
NKX3−1
1
2
0
1000
2000
3000
SNVs
0
39%
MuTect−Battenberg
N
NKX3−1
1
2
3
PGA
0
10
20
30
40
50
60
70
80
0
56%
MuTect−TITAN
N
RB1
NKX3−1
1
2
3
0
1000
2000
3000
4000
5000
6000
7000
SNVs
0
44%

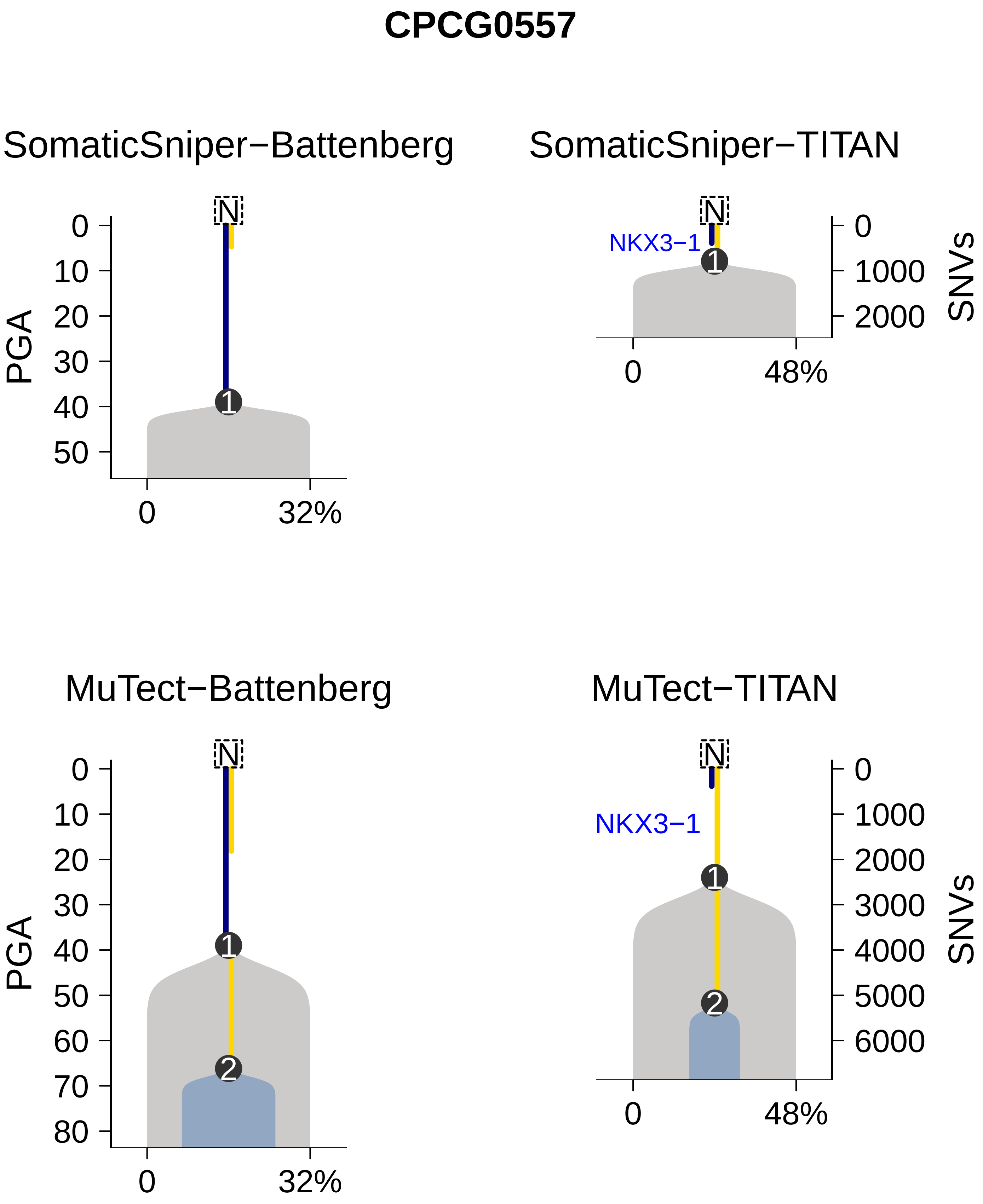

CPCG0557
SomaticSniper−Battenberg
SomaticSniper−TITAN
MuTect−Battenberg
MuTect−TITAN
N
1
2
NKX3−1
PGA
SNVs
0
10
20
30
40
50
60
70
80
1000
2000
3000
4000
5000
6000
32%
48%

CPCG0559

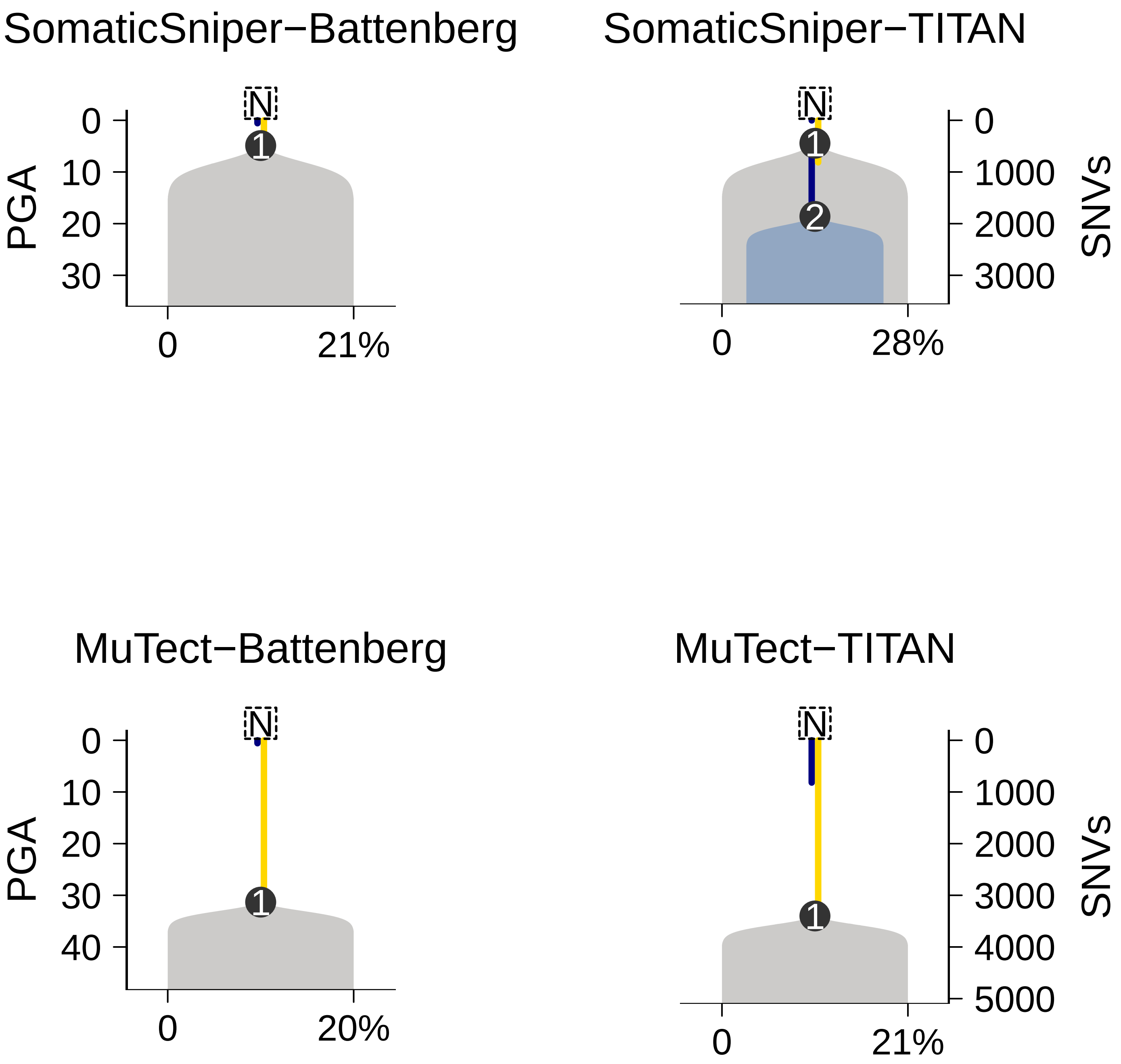
SomaticSniper−Battenberg
SomaticSniper−TITAN
MuTect−Battenberg
MuTect−TITAN
N
1
2
PGA
SNVs
0
10
20
30
40
1000
2000
3000
4000
5000
21%
28%
20%

CPCG0560

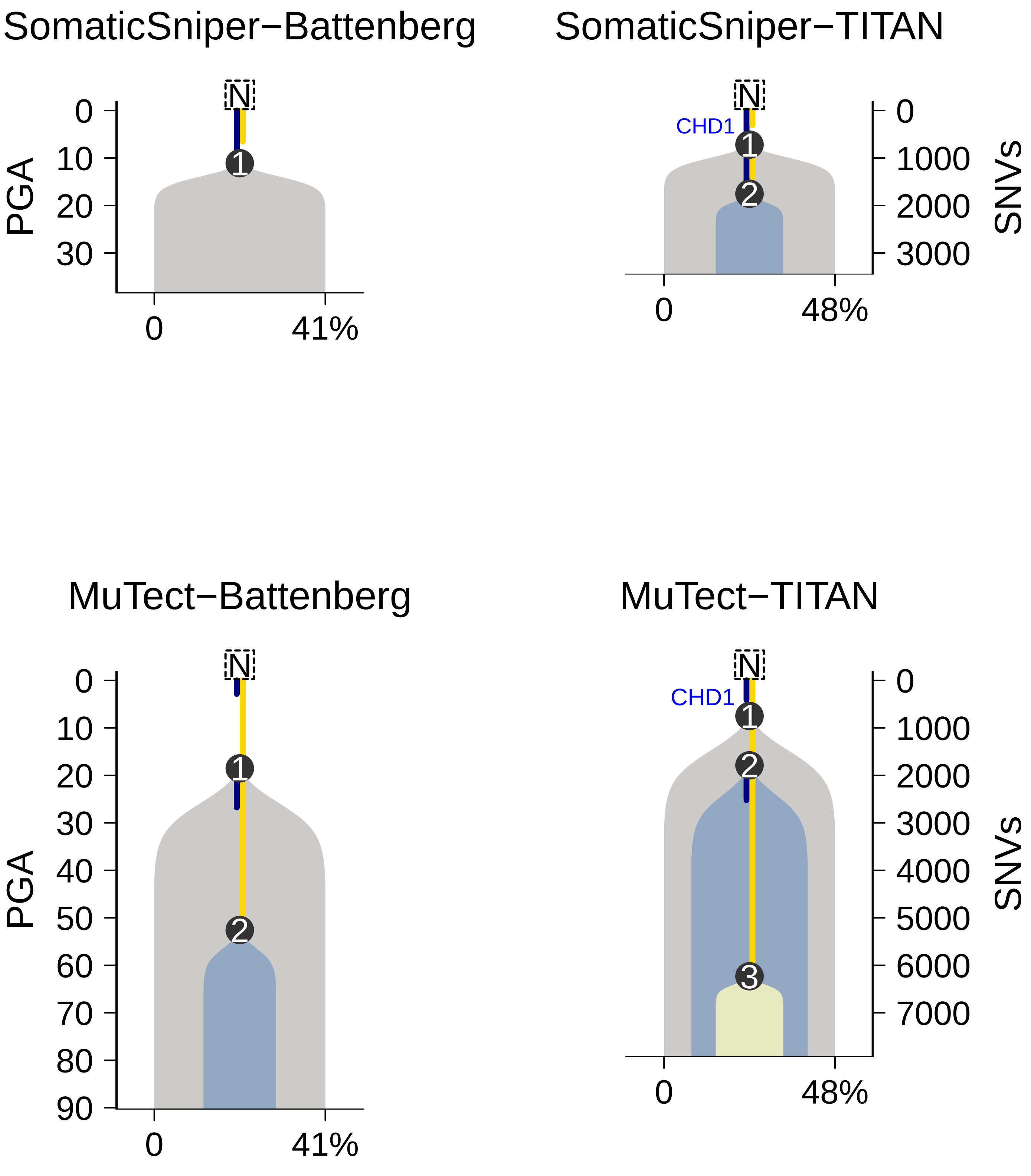

SomaticSniper−Battenberg
SomaticSniper−TITAN
N
1
2
CHD1
PGA
0
10
20
30
41%
48%
1000
2000
3000
SNVs
MuTect−Battenberg
MuTect−TITAN
3
40
50
60
70
80
90
4000
5000
6000
7000

CPCG0561

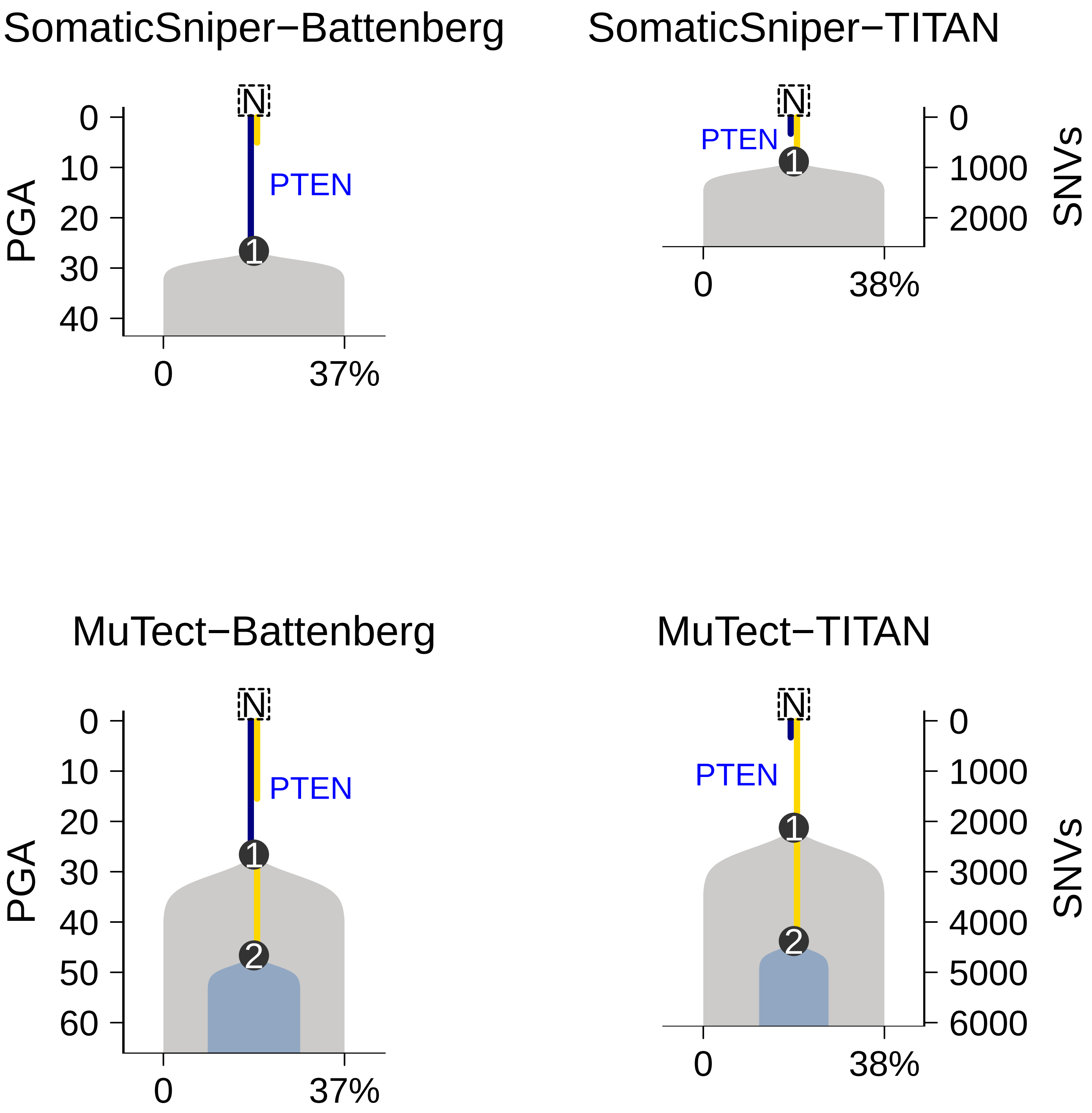
SomaticSniper−Battenberg
SomaticSniper−TITAN
MuTect−Battenberg
MuTect−TITAN
N
1
2
PTEN
PGA
SNVs
0
10
20
30
40
50
60
1000
2000
3000
4000
5000
6000
37%
38%

CPCG0562

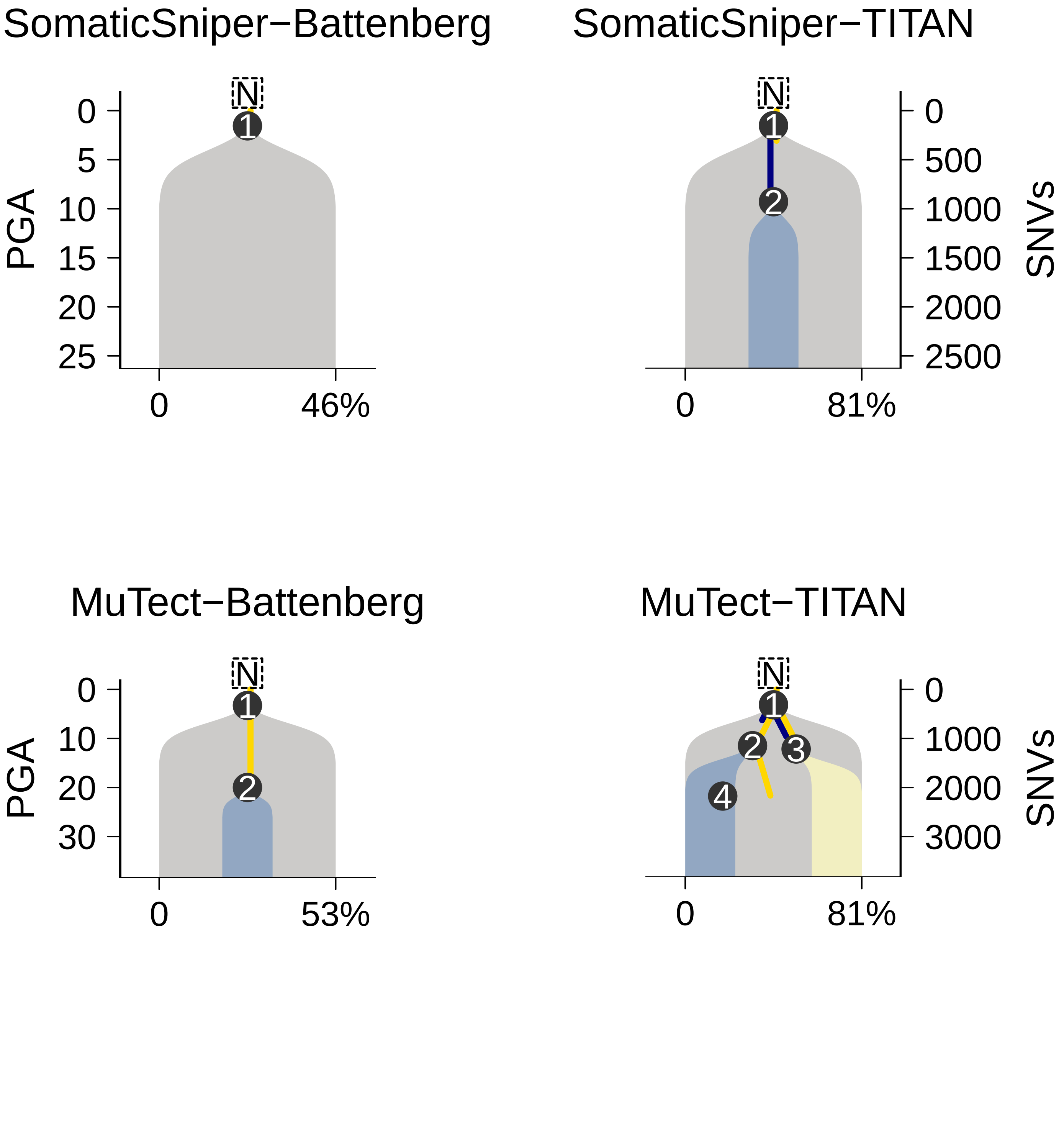

SomaticSniper−Battenberg
SomaticSniper−TITAN
MuTect−Battenberg
MuTect−TITAN
N
1
2
3
4
PGA
SNVs
0
5
10
15
20
25
30
500
1000
1500
2000
2500
3000
46%
81%
53%

CPCG0565

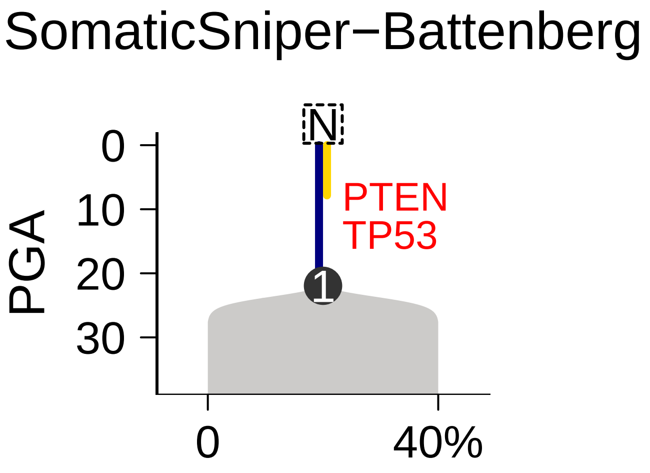

SomaticSniper−Battenberg
N
PTEN
TP53
1
PGA
0
10
20
30
0
40%

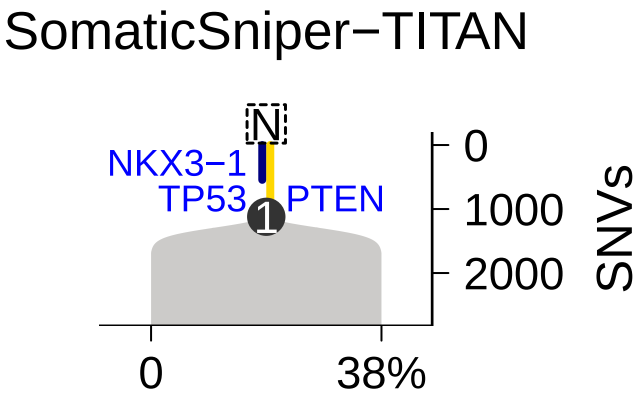

SomaticSniper−TITAN
N
NKX3−1
TP53
PTEN
1
0
1000
2000
SNVs
0
38%

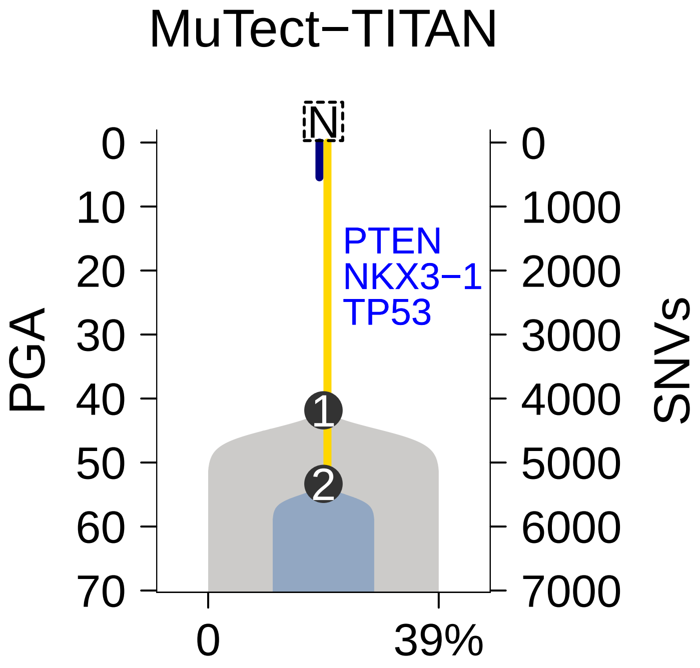

MuTect−TITAN
N
PTEN
NKX3−1
TP53
1
2
PGA
0
10
20
30
40
50
60
70
0
1000
2000
3000
4000
5000
6000
7000
SNVs
0
39%

CPCG0566

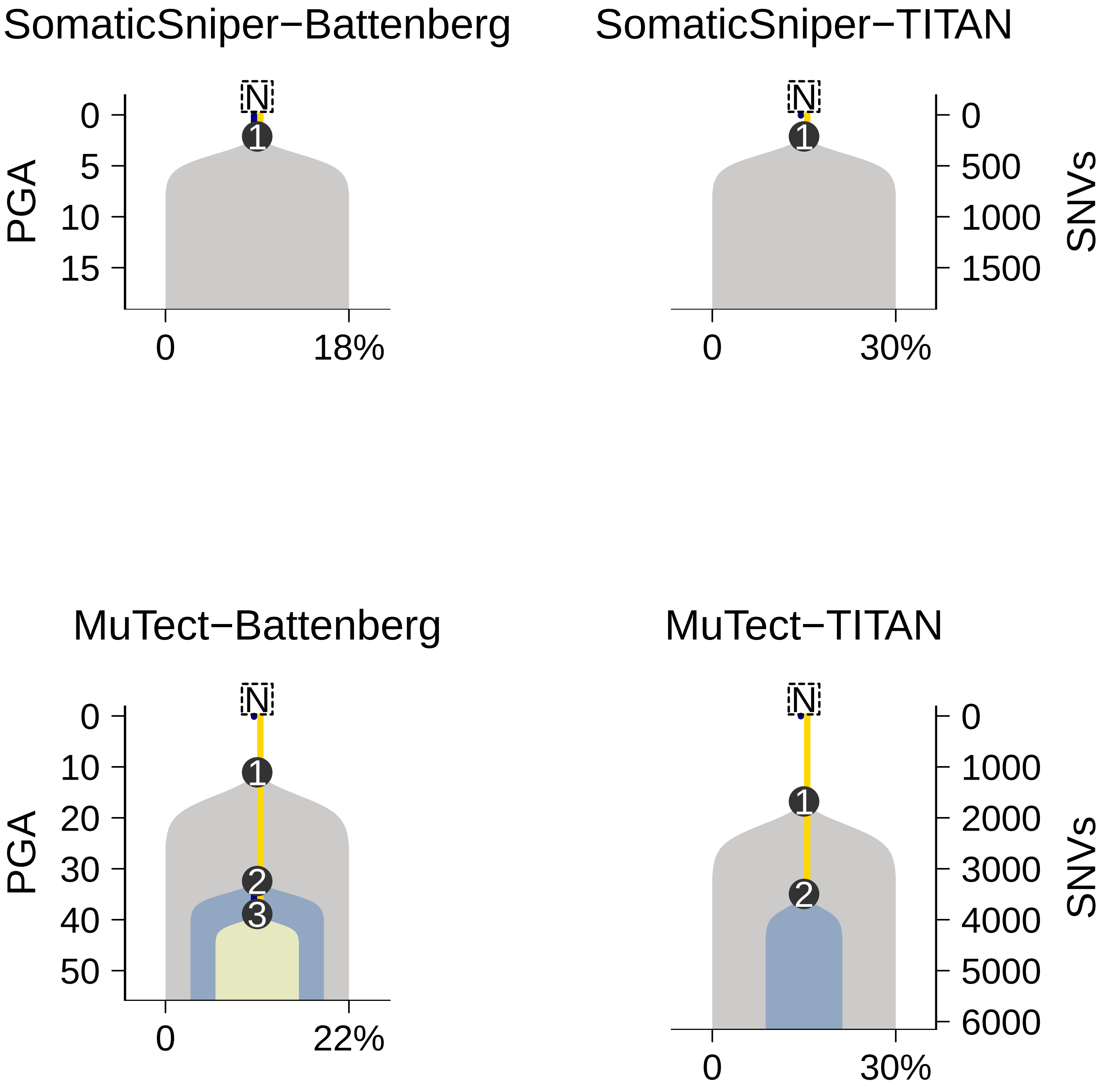

SomaticSniper−Battenberg
SomaticSniper−TITAN
MuTect−Battenberg
MuTect−TITAN
N
1
2
3
PGA
SNVs
0
5
10
15
20
30
40
50
500
1000
1500
2000
3000
4000
5000
6000
18%
22%
30%

CPCG0567

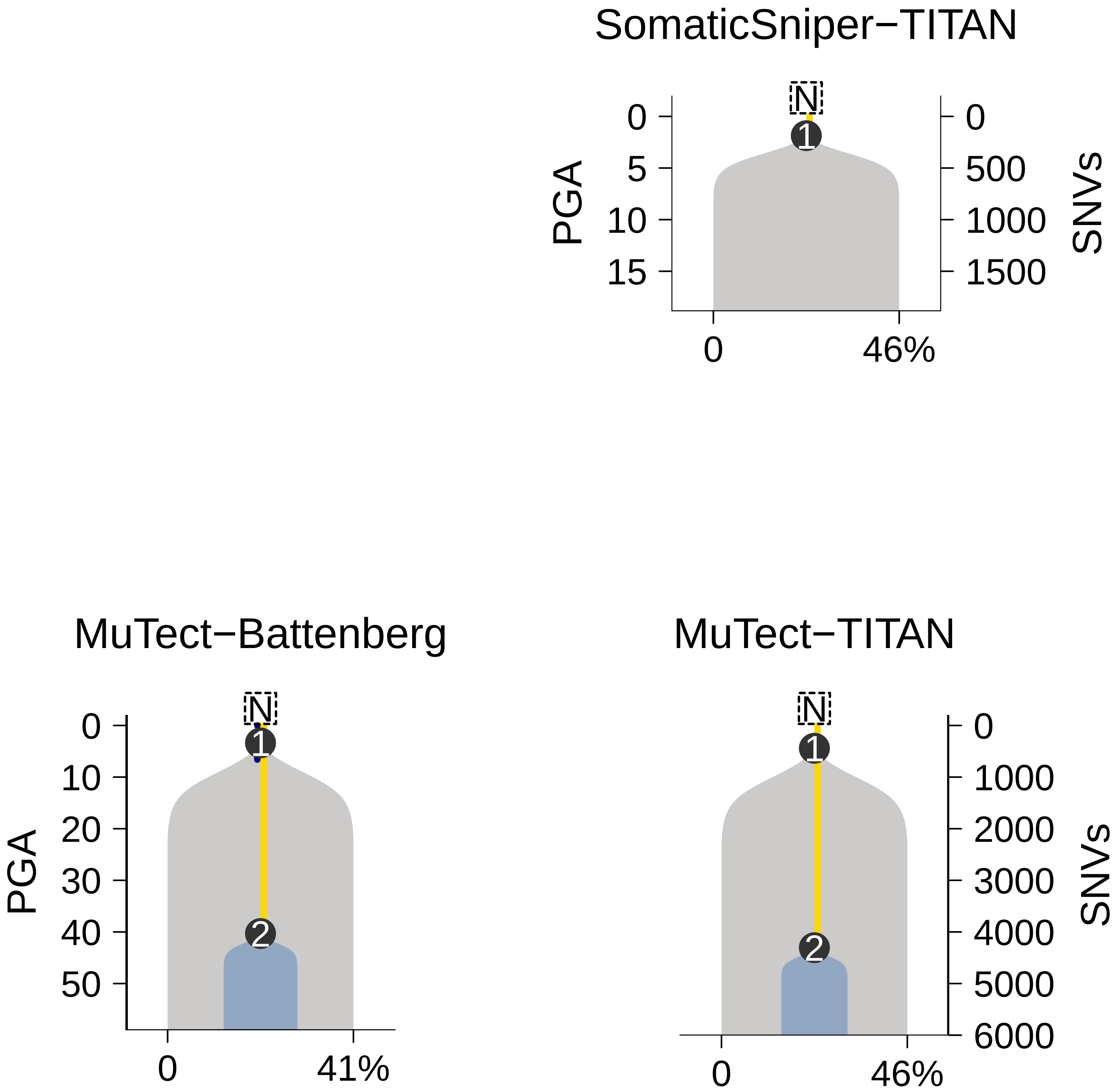

SomaticSniper−TITAN
N
1
PGA
0
5
10
15
0
500
1000
1500
SNVs
0
46%
MuTect−Battenberg
N
1
2
PGA
0
10
20
30
40
50
0
41%
MuTect−TITAN
N
1
2
0
1000
2000
3000
4000
5000
6000
SNVs
0
46%

CPCG0570

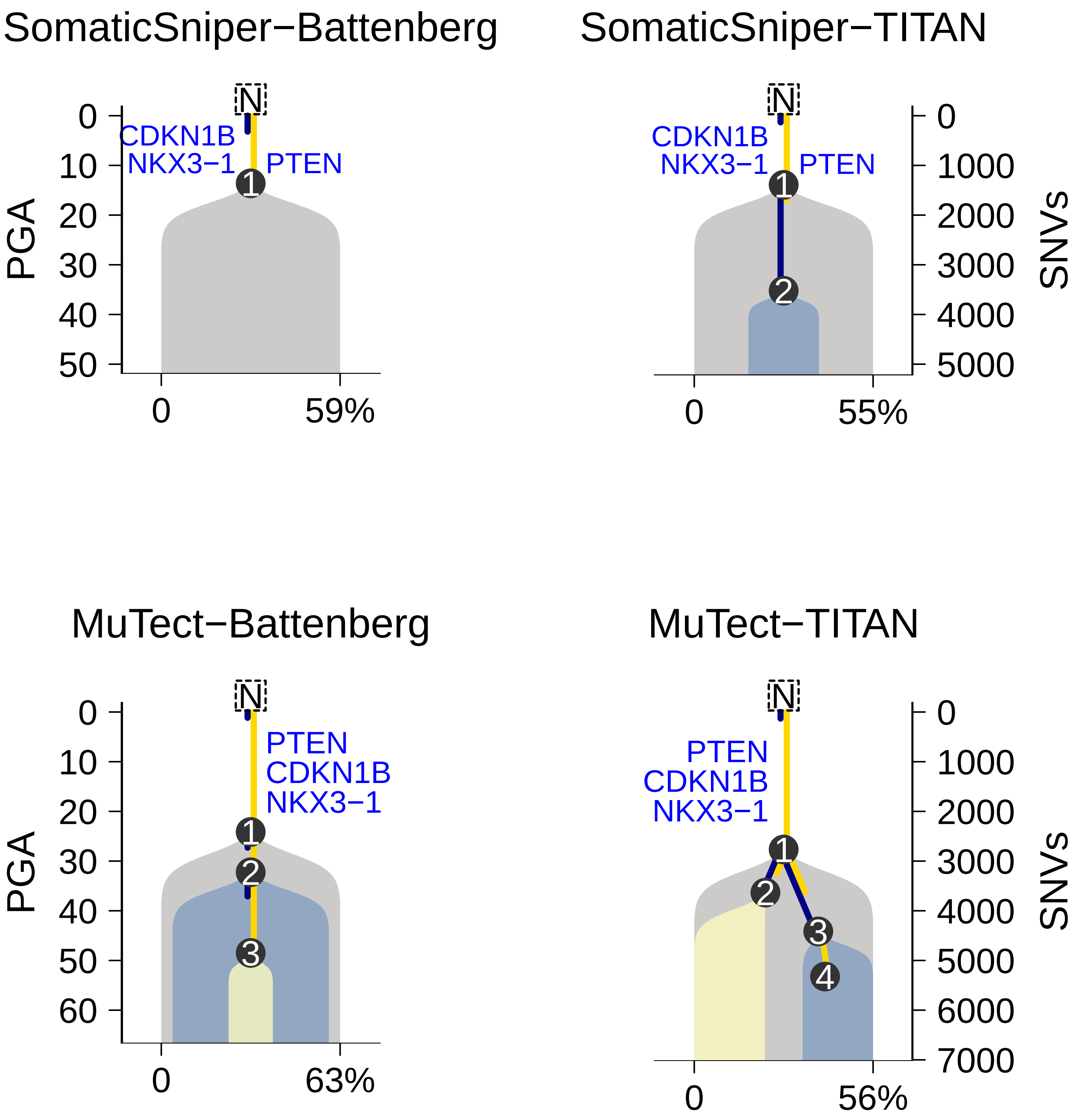
SomaticSniper−Battenberg
SomaticSniper−TITAN
MuTect−Battenberg
MuTect−TITAN
N
CDKN1B
NKX3−1
PTEN
PGA
SNVs
0
10
20
30
40
50
60
1000
2000
3000
4000
5000
6000
7000
59%
55%
63%
56%
1
2
3
4

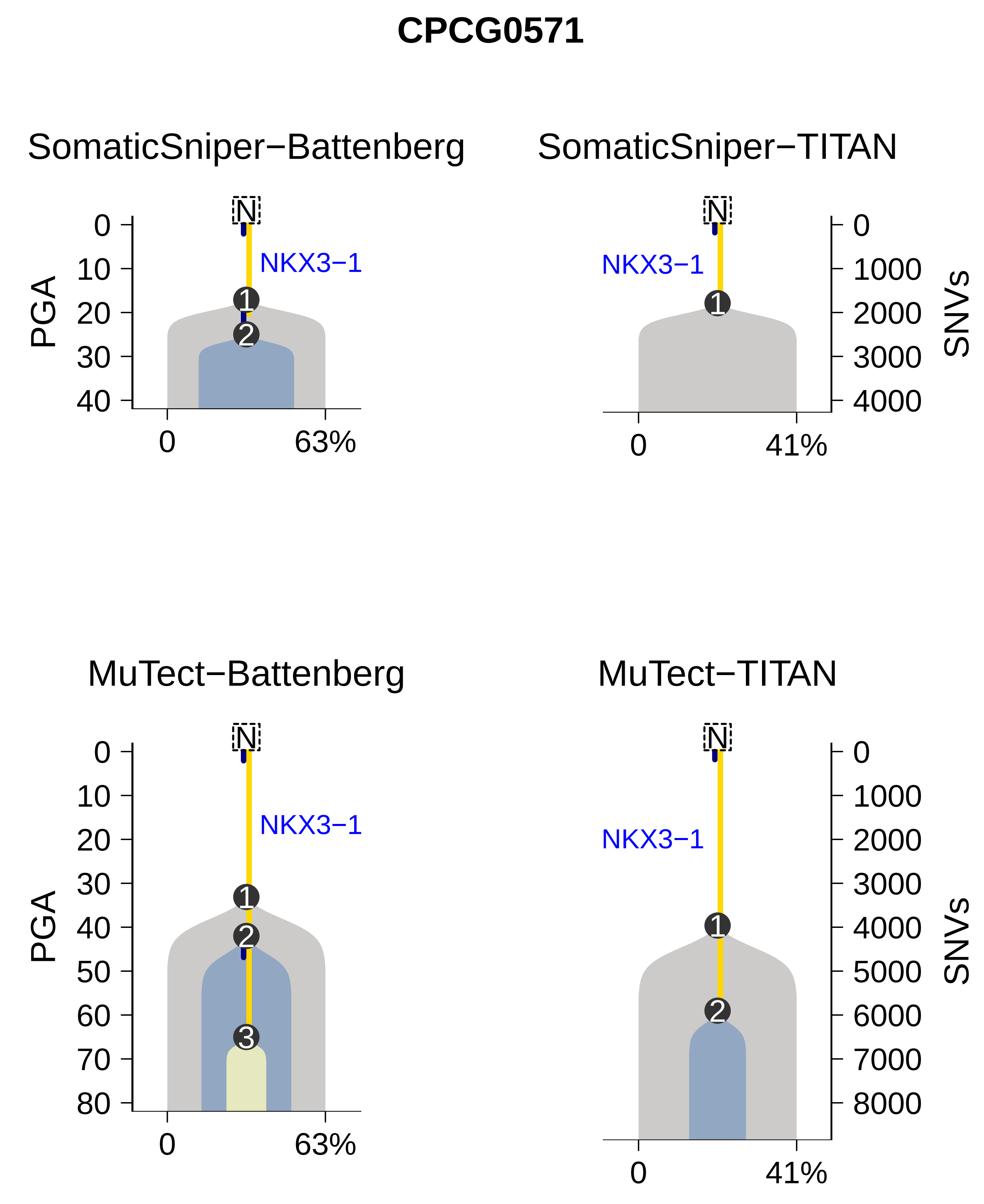

CPCG0571
SomaticSniper−Battenberg
SomaticSniper−TITAN
MuTect−Battenberg
MuTect−TITAN
N
NKX3−1
1
2
3
PGA
SNVs
0
10
20
30
40
50
60
70
80
1000
2000
3000
4000
5000
6000
7000
8000
63%
41%

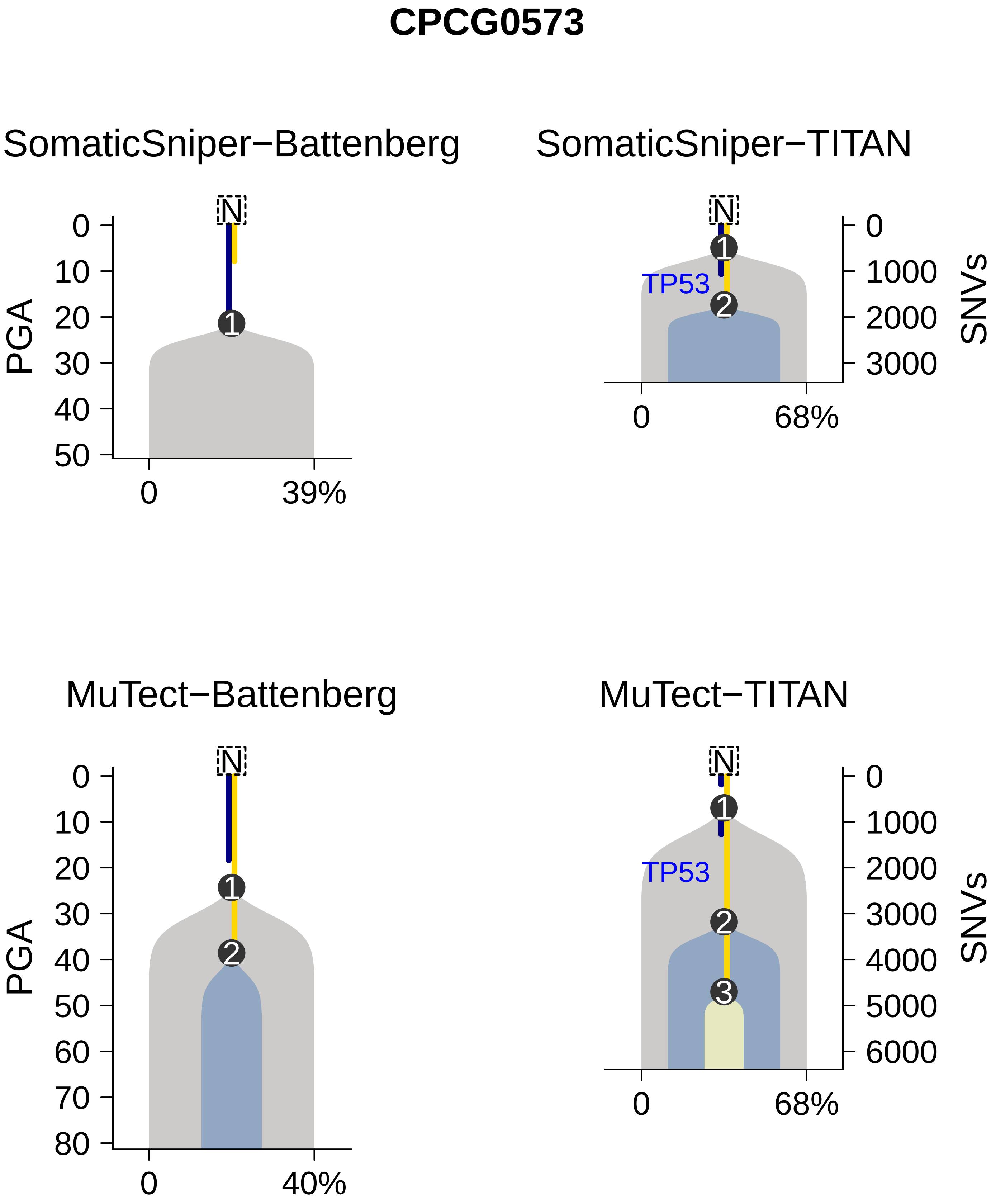
CPCG0573
SomaticSniper−Battenberg
SomaticSniper−TITAN
MuTect−Battenberg
MuTect−TITAN
N
TP53
PGA
SNVs
0
39%
40%
68%

CPCG0575

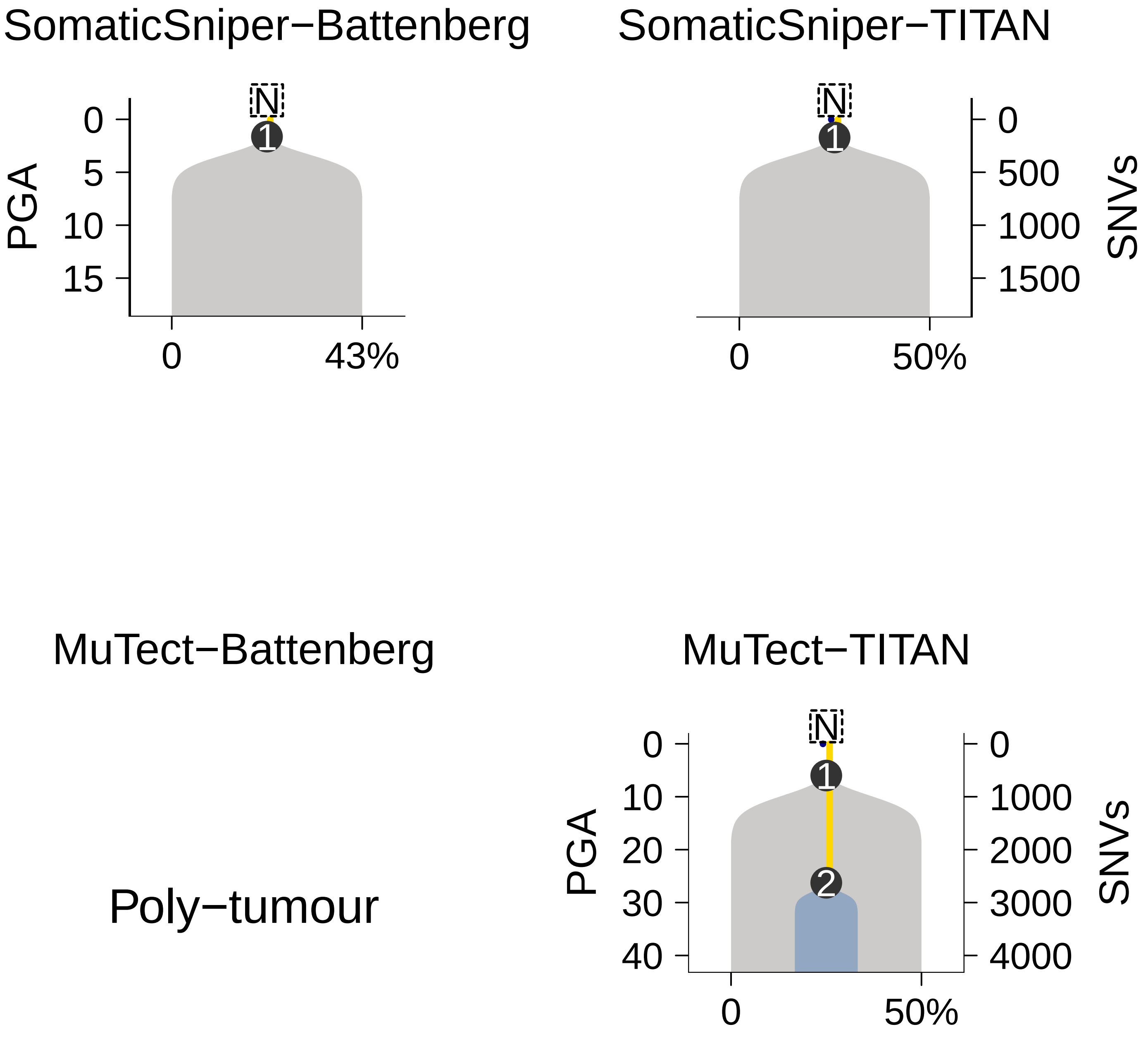

SomaticSniper−Battenberg
SomaticSniper−TITAN
N
1
PGA
0
5
10
15
0
43%
0
500
1000
1500
SNVs
50%
MuTect−Battenberg
MuTect−TITAN
Poly−tumour
2
20
30
40
2000
3000
4000

CPCG0576

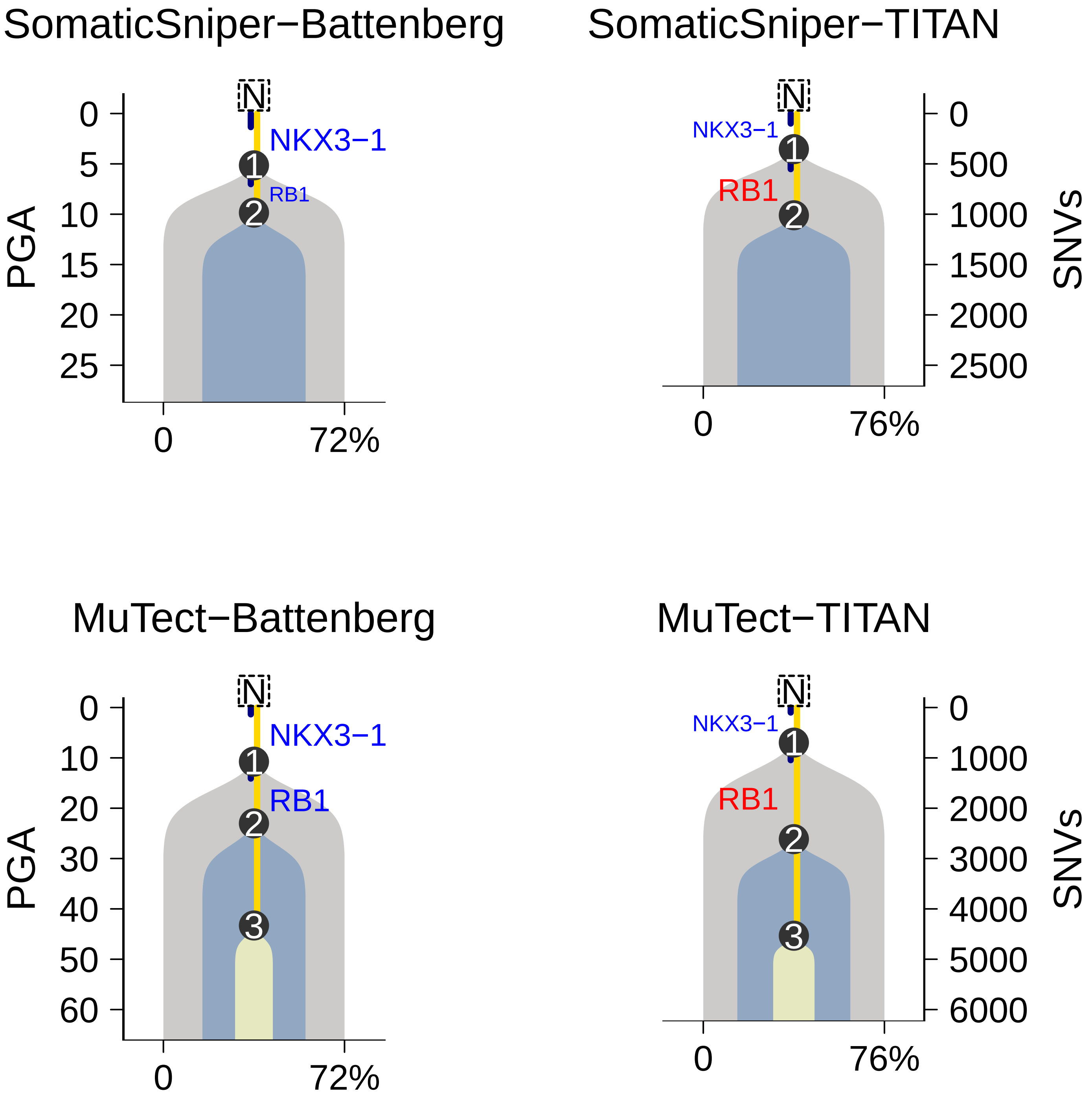

SomaticSniper−Battenberg
SomaticSniper−TITAN
MuTect−Battenberg
MuTect−TITAN
N
NKX3−1
RB1
1
2
3
PGA
SNVs
0
5
10
15
20
25
30
40
50
60
500
1000
1500
2000
2500
3000
4000
5000
6000
72%
76%

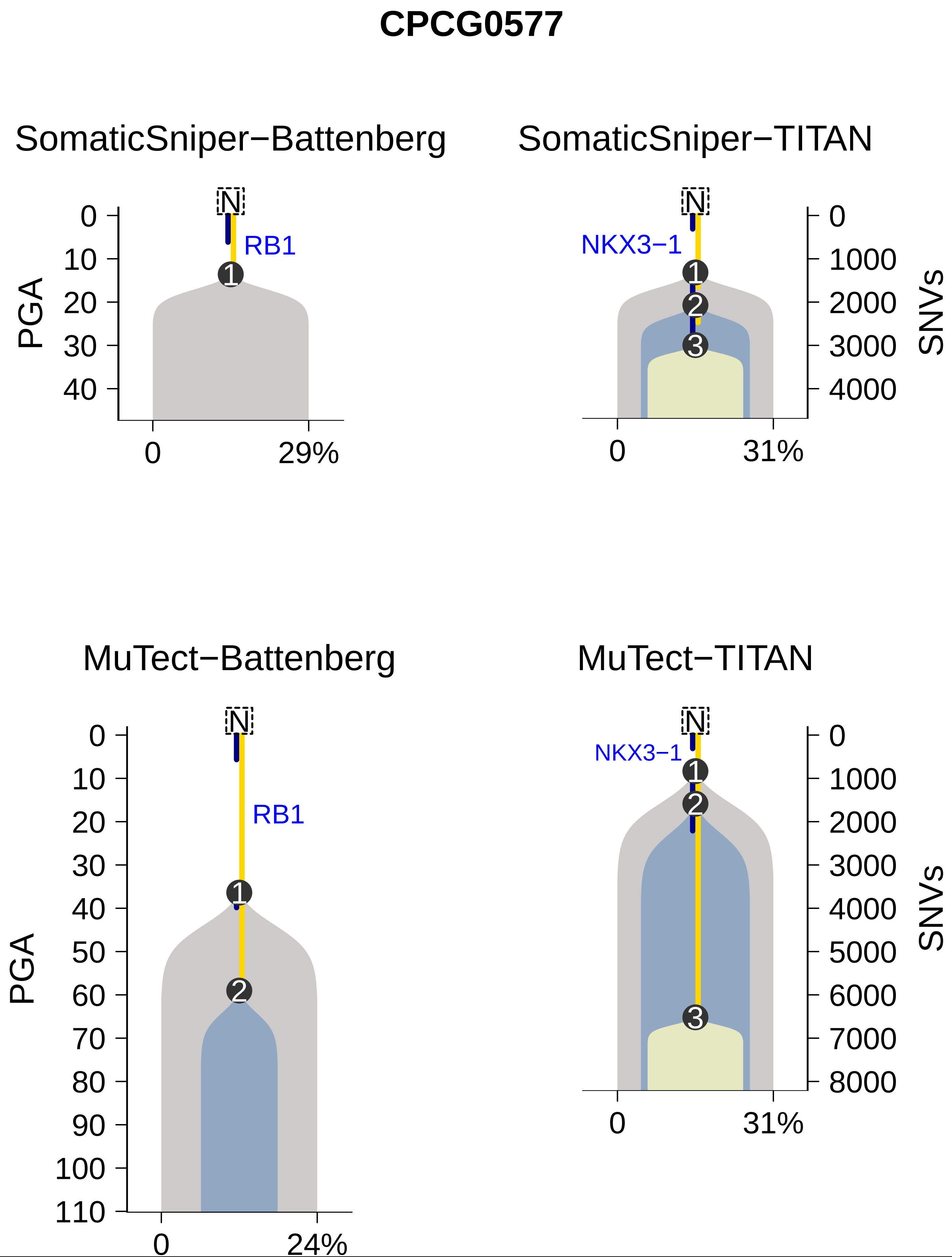

CPCG0577
SomaticSniper−Battenberg
SomaticSniper−TITAN
MuTect−Battenberg
MuTect−TITAN
N
RB1
NKX3−1
PGA
SNVs
1
2
3
0
10
20
30
40
50
60
70
80
90
100
110
1000
2000
3000
4000
5000
6000
7000
8000
29%
31%
24%

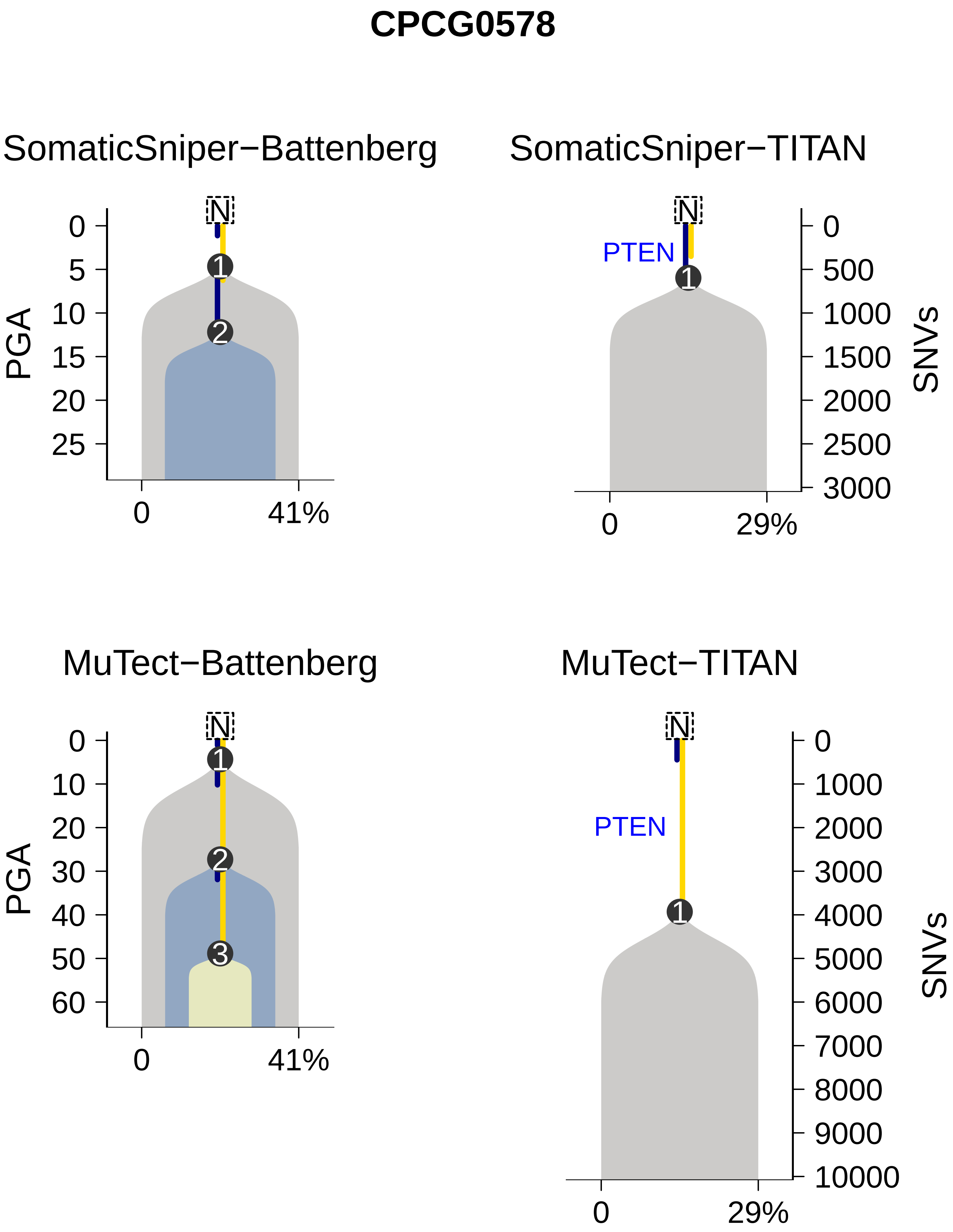
CPCG0578
SomaticSniper−Battenberg
SomaticSniper−TITAN
MuTect−Battenberg
MuTect−TITAN
PGA
SNVs
N
PTEN
0
41%
29%

CPCG0580

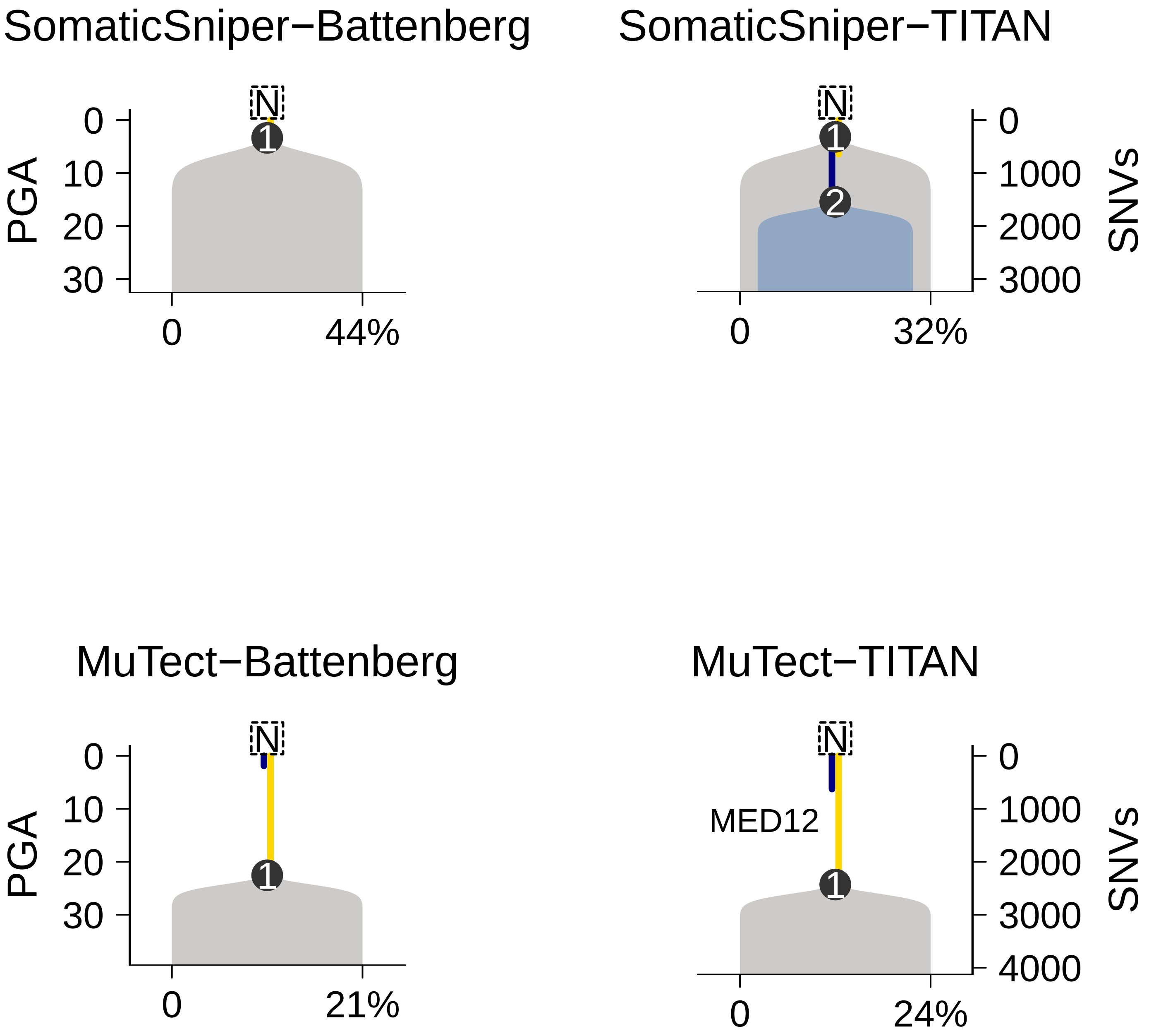

SomaticSniper−Battenberg
SomaticSniper−TITAN
MuTect−Battenberg
MuTect−TITAN
N
1
2
PGA
SNVs
0
10
20
30
1000
2000
3000
4000
44%
32%
21%
24%
MED12

CPCG0581

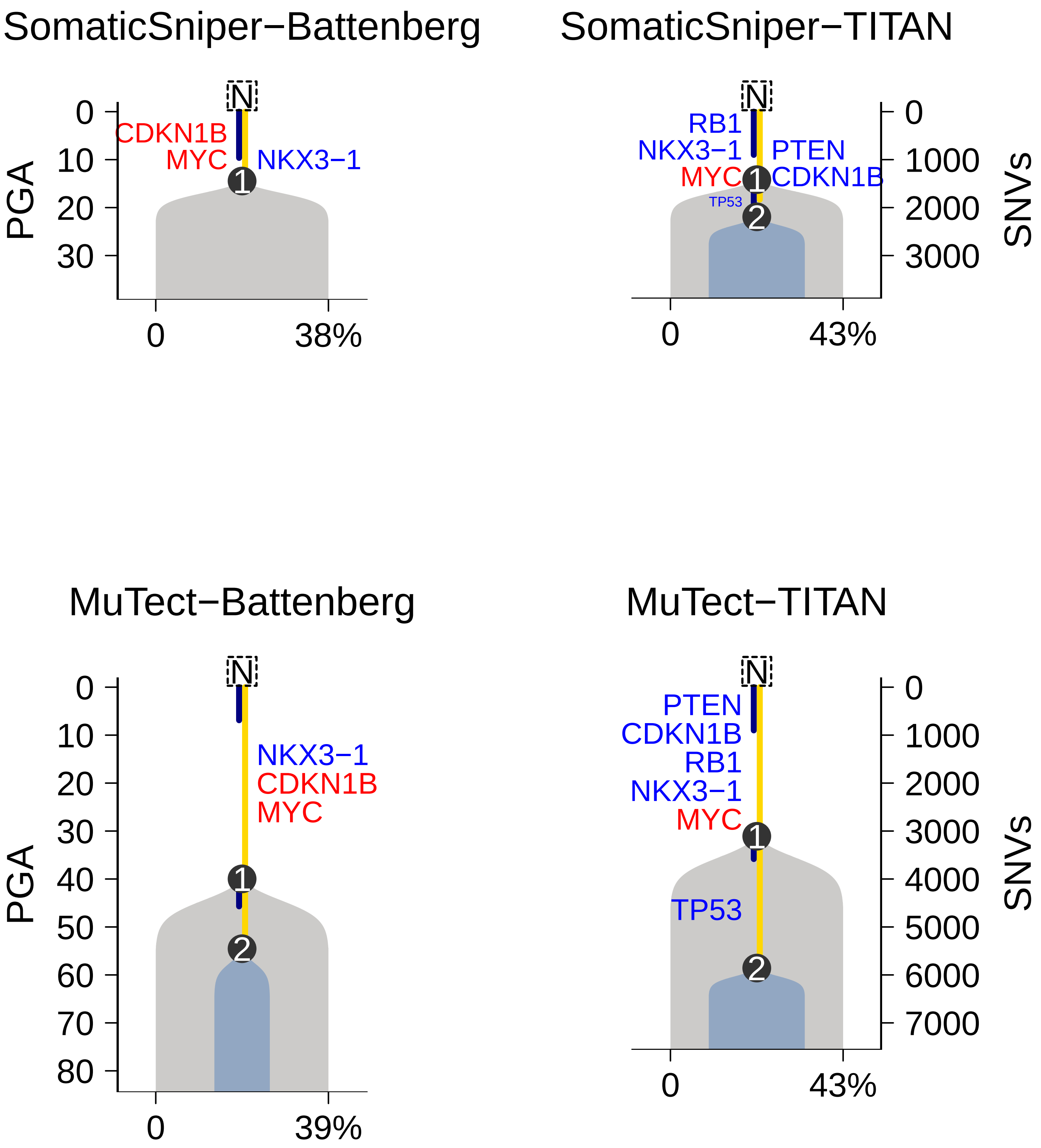

SomaticSniper−Battenberg
N
CDKN1B
MYC
NKX3−1
1
PGA
0
10
20
30
0
38%
SomaticSniper−TITAN
N
RB1
NKX3−1
PTEN
MYC
CDKN1B
TP53
1
2
0
1000
2000
3000
SNVs
0
43%
MuTect−Battenberg
N
NKX3−1
CDKN1B
MYC
1
2
PGA
0
10
20
30
40
50
60
70
80
0
39%
MuTect−TITAN
N
PTEN
CDKN1B
RB1
NKX3−1
MYC
TP53
1
2
0
1000
2000
3000
4000
5000
6000
7000
SNVs
0
43%

CPCG0582

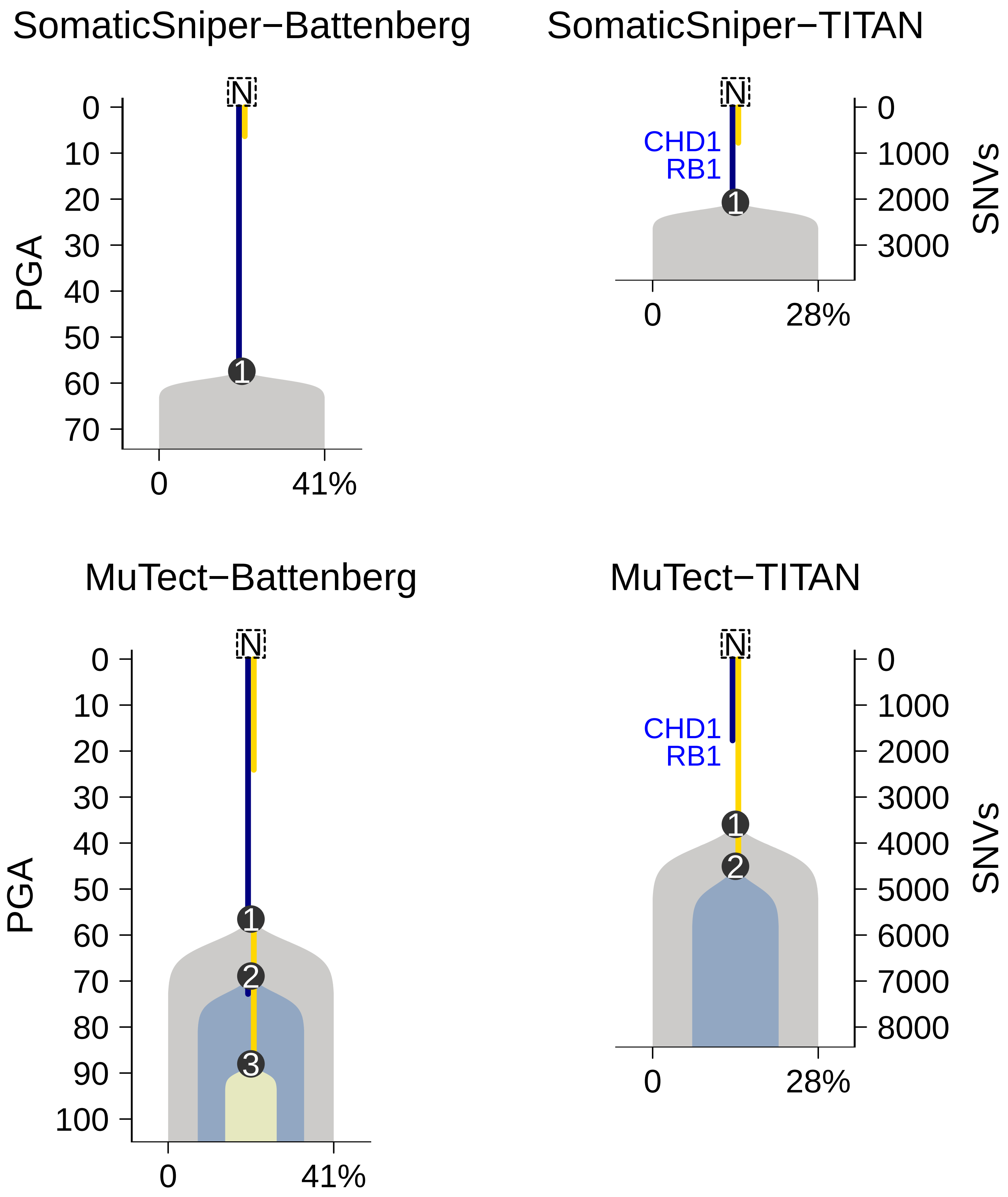
SomaticSniper−Battenberg
SomaticSniper−TITAN
MuTect−Battenberg
MuTect−TITAN
N
1
2
3
CHD1
RB1
PGA
SNVs
0
10
20
30
40
50
60
70
80
90
100
1000
2000
3000
4000
5000
6000
7000
8000
41%
28%

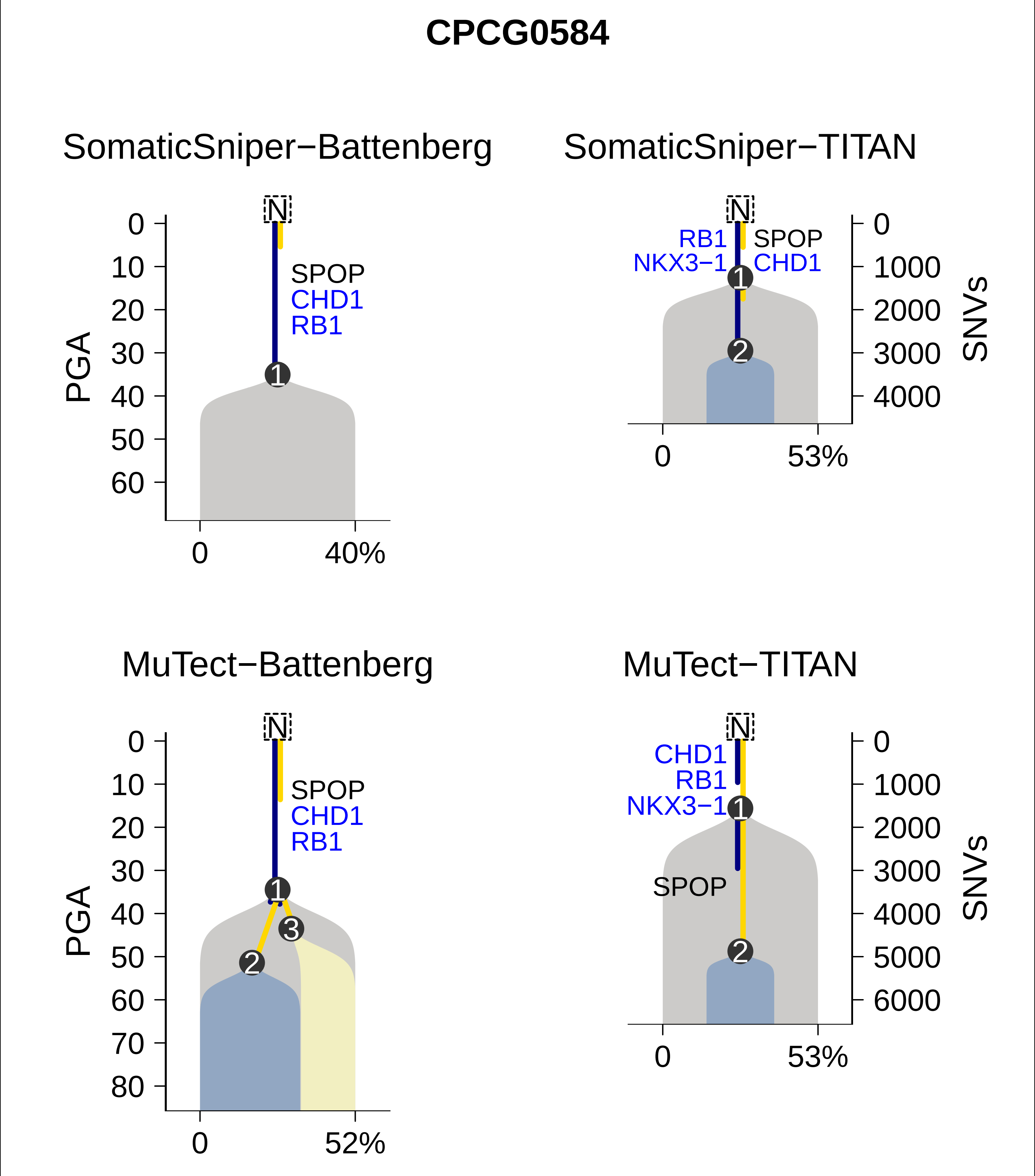
CPCG0584
SomaticSniper−Battenberg
N
SPOP
CHD1
RB1
1
PGA
0
10
20
30
40
50
60
0
40%
SomaticSniper−TITAN
N
RB1
NKX3−1
SPOP
CHD1
1
2
0
1000
2000
3000
4000
SNVs
0
53%
MuTect−Battenberg
N
SPOP
CHD1
RB1
1
2
3
PGA
0
10
20
30
40
50
60
70
80
0
52%
MuTect−TITAN
N
CHD1
RB1
NKX3−1
1
SPOP
2
0
1000
2000
3000
4000
5000
6000
SNVs
0
53%

CPCG0587

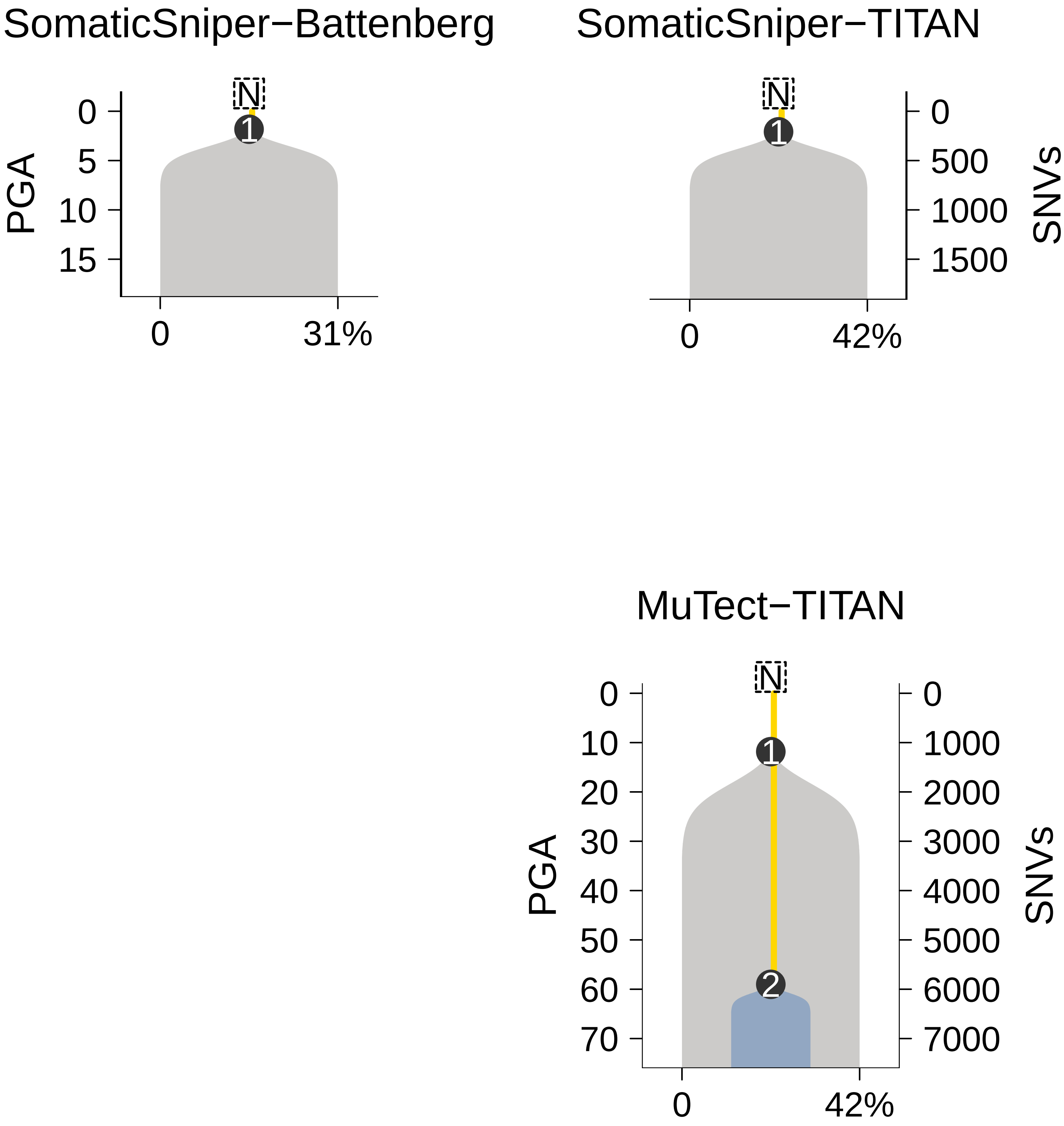
SomaticSniper−Battenberg
SomaticSniper−TITAN
MuTect−TITAN
N
1
2
PGA
SNVs
0
5
10
15
31%
42%
500
1000
1500
20
30
40
50
60
70
2000
3000
4000
5000
6000
7000

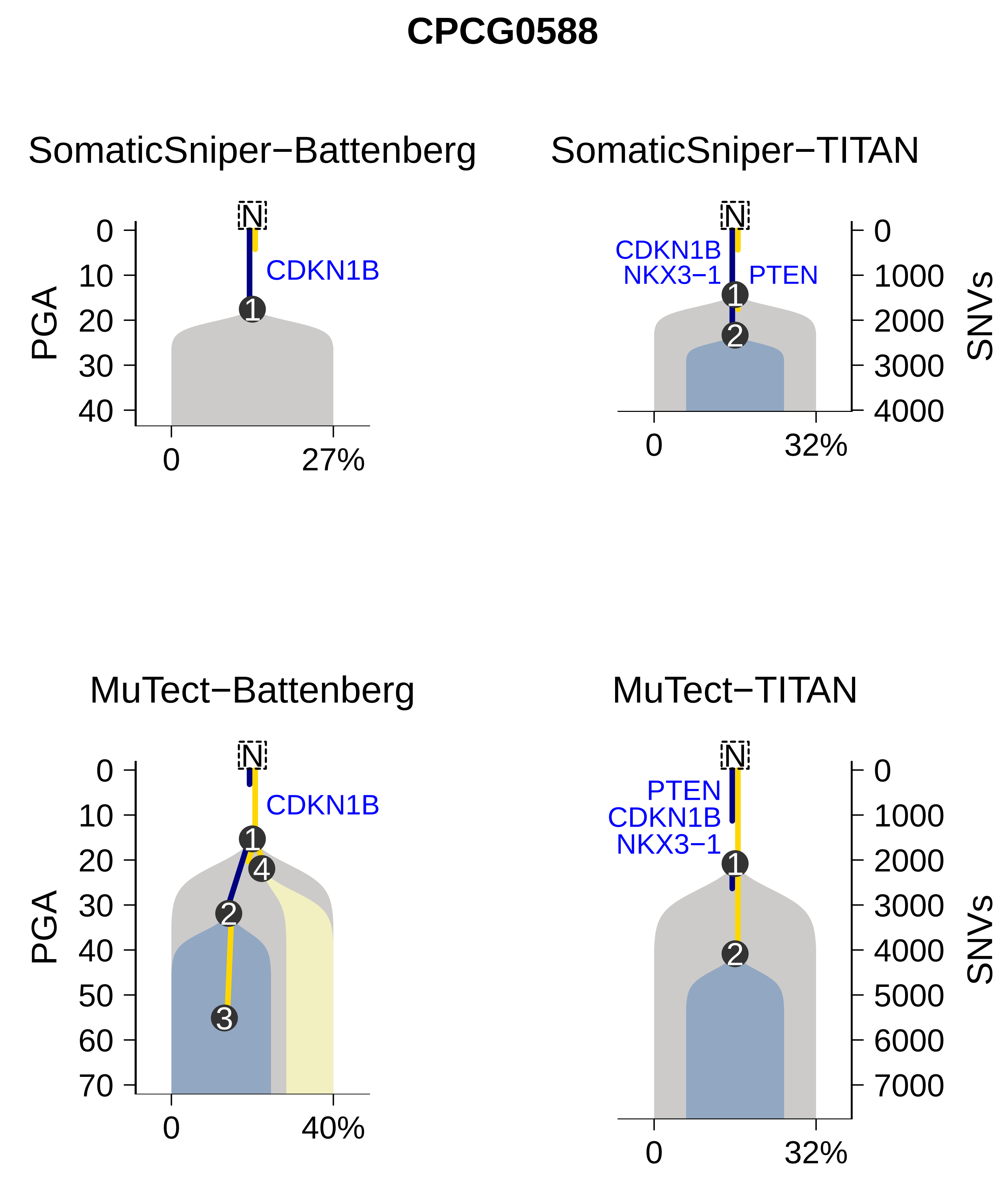

CPCG0588
SomaticSniper−Battenberg
SomaticSniper−TITAN
MuTect−Battenberg
MuTect−TITAN
N
CDKN1B
NKX3−1
PTEN
PGA
SNVs
0
10
20
30
40
50
60
70
27%
32%
40%
1000
2000
3000
4000
5000
6000
7000
1
2
3
4

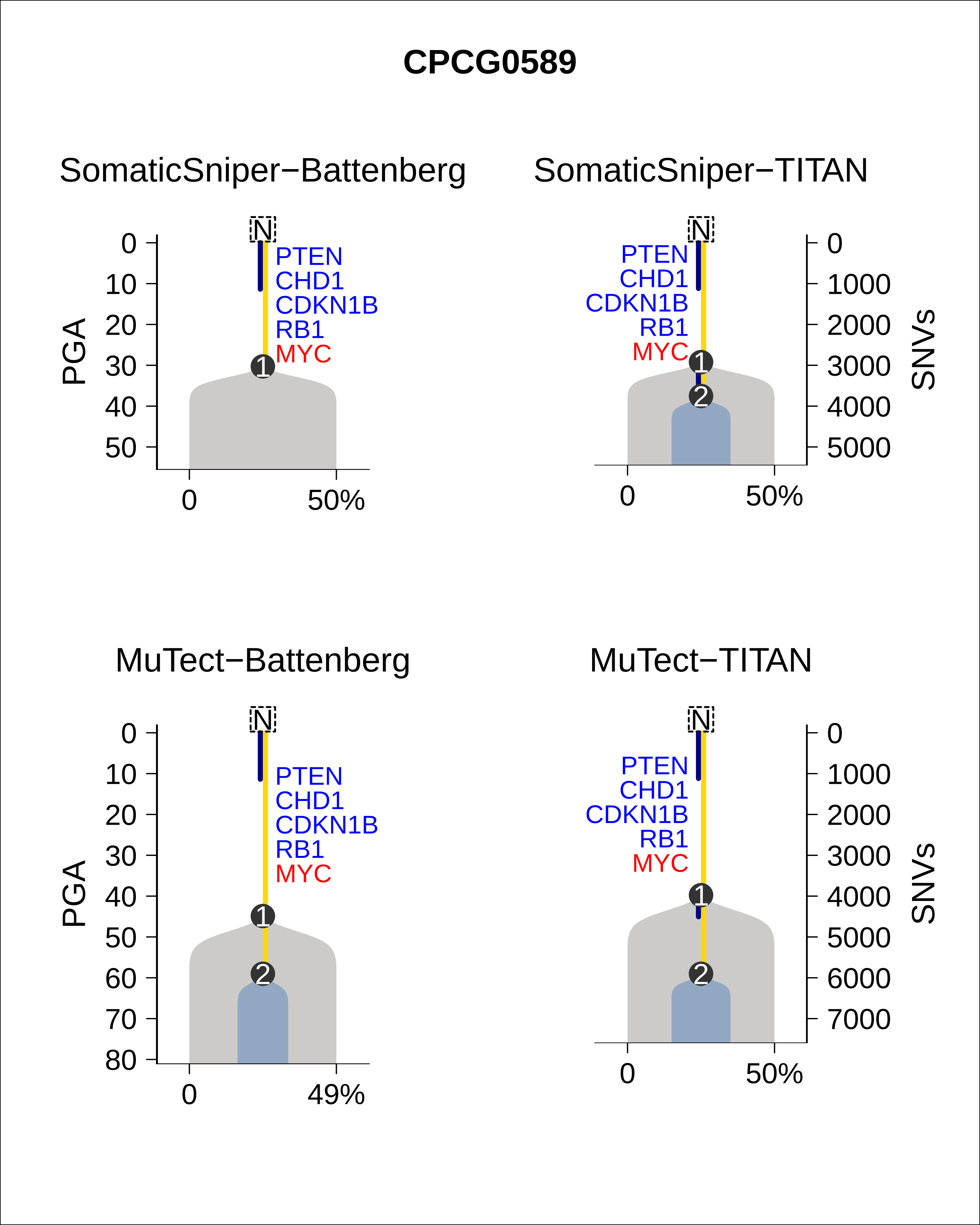
CPCG0589
SomaticSniper−Battenberg
SomaticSniper−TITAN
MuTect−Battenberg
MuTect−TITAN
PGA
SNVs
N
PTEN
CHD1
CDKN1B
RB1
MYC
1
2
0
10
20
30
40
50
60
70
80
1000
2000
3000
4000
5000
6000
7000
50%
49%

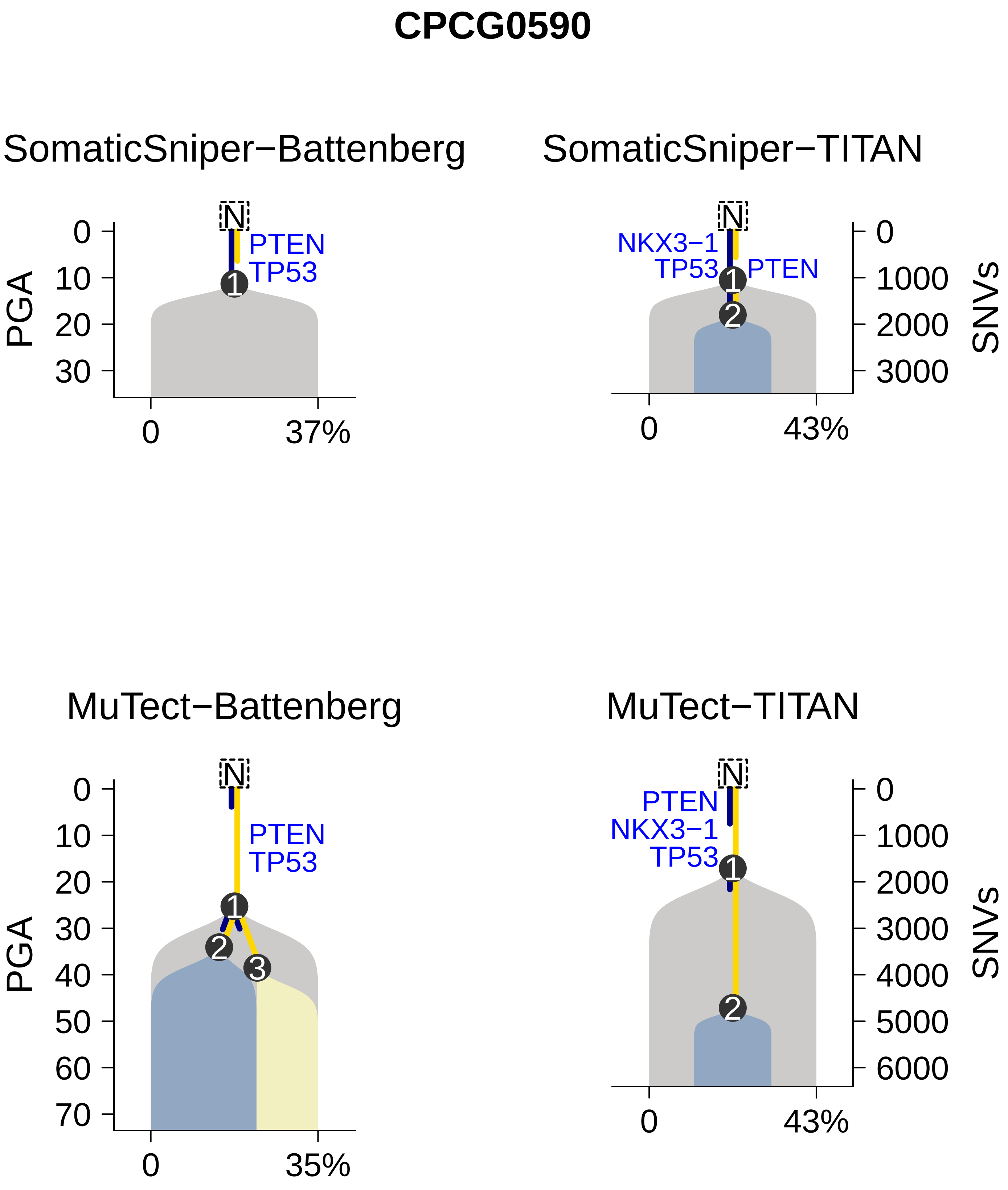

CPCG0590
SomaticSniper−Battenberg
SomaticSniper−TITAN
MuTect−Battenberg
MuTect−TITAN
N
PTEN
TP53
NKX3−1
1
2
3
PGA
SNVs
0
10
20
30
40
50
60
70
1000
2000
3000
4000
5000
6000
37%
43%
35%

CPCG0591

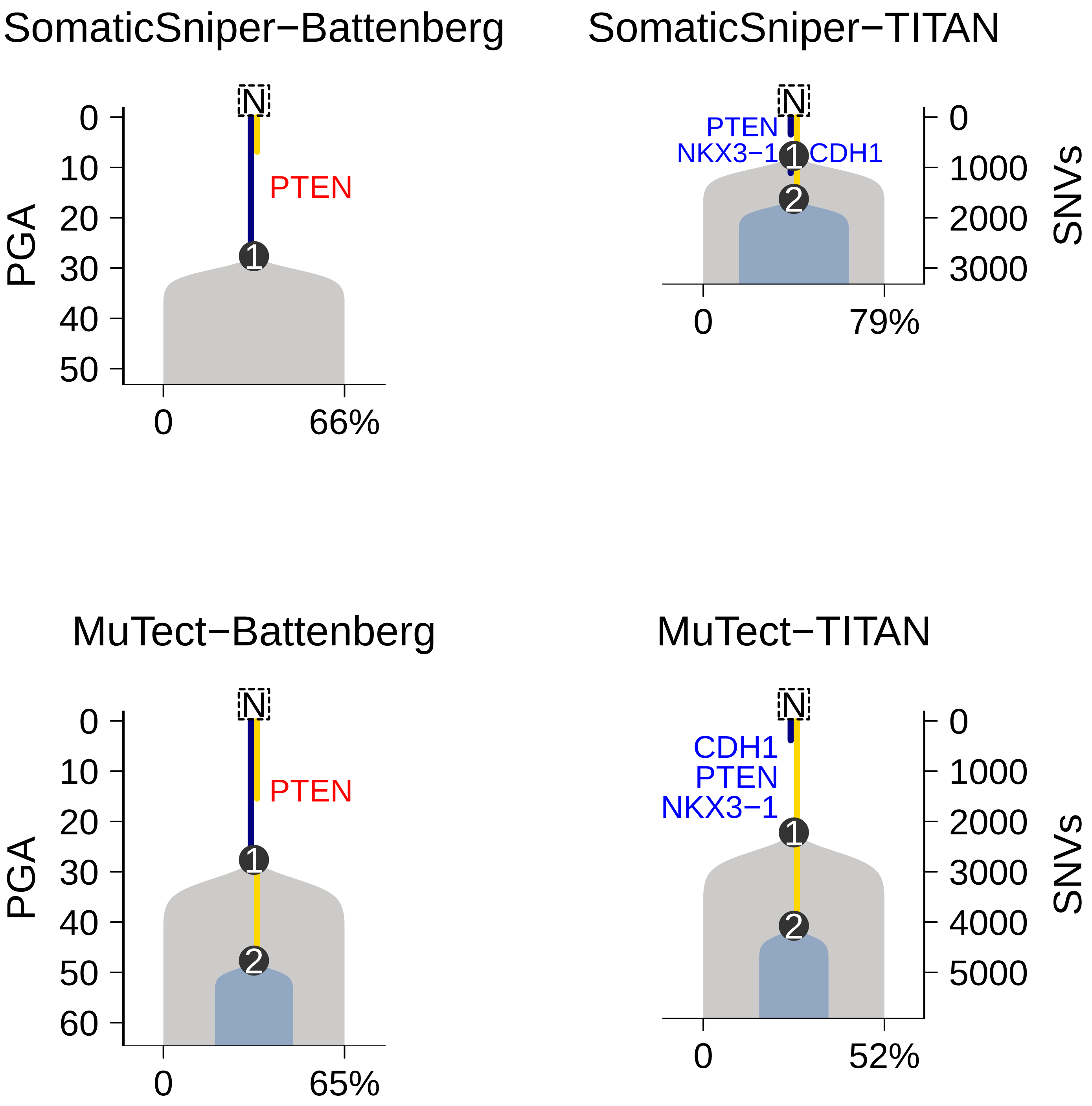
SomaticSniper−Battenberg
SomaticSniper−TITAN
MuTect−Battenberg
MuTect−TITAN
N
1
2
PTEN
NKX3−1
CDH1
PGA
SNVs
0
10
20
30
40
50
60
1000
2000
3000
4000
5000
66%
79%
65%
52%

CPCG0592

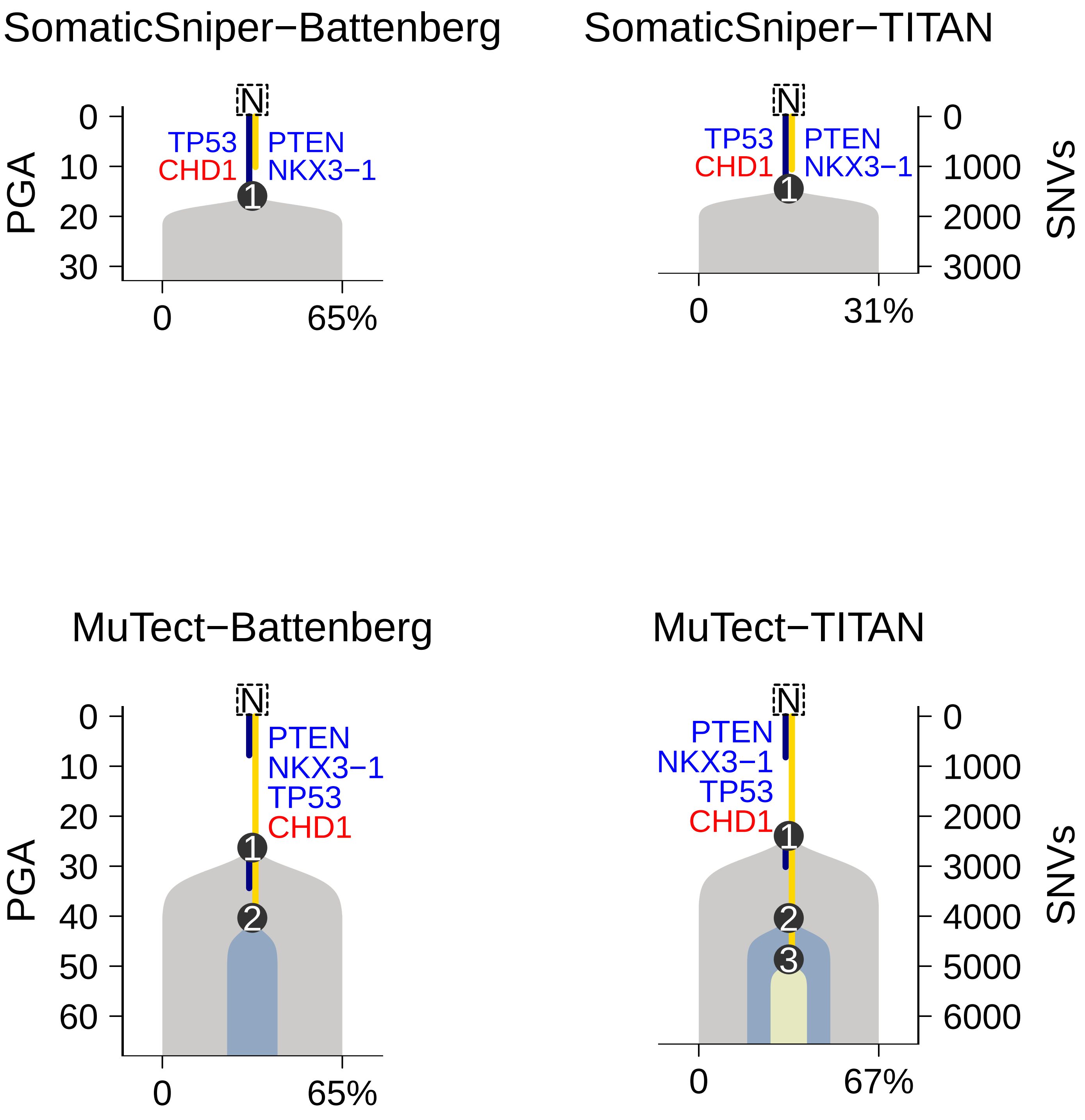

SomaticSniper−Battenberg
SomaticSniper−TITAN
MuTect−Battenberg
MuTect−TITAN
N
TP53
CHD1
PTEN
NKX3−1
1
2
3
PGA
SNVs
0
10
20
30
40
50
60
1000
2000
3000
4000
5000
6000
65%
31%
67%

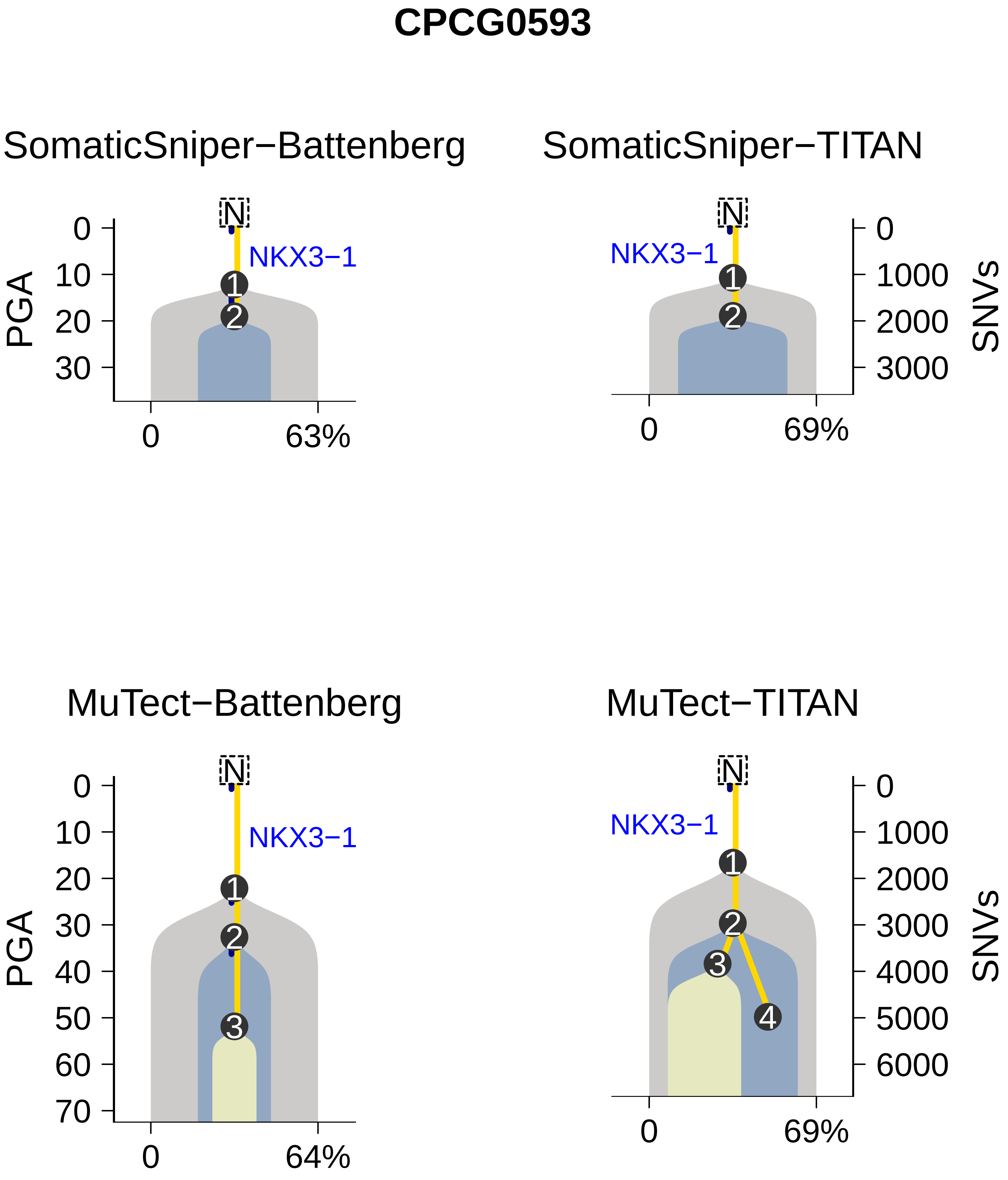
CPCG0593
SomaticSniper−Battenberg
SomaticSniper−TITAN
MuTect−Battenberg
MuTect−TITAN
N
NKX3−1
PGA
SNVs
0
10
20
30
40
50
60
70
1000
2000
3000
4000
5000
6000
63%
69%
64%
1
2
3
4

CPCG0595

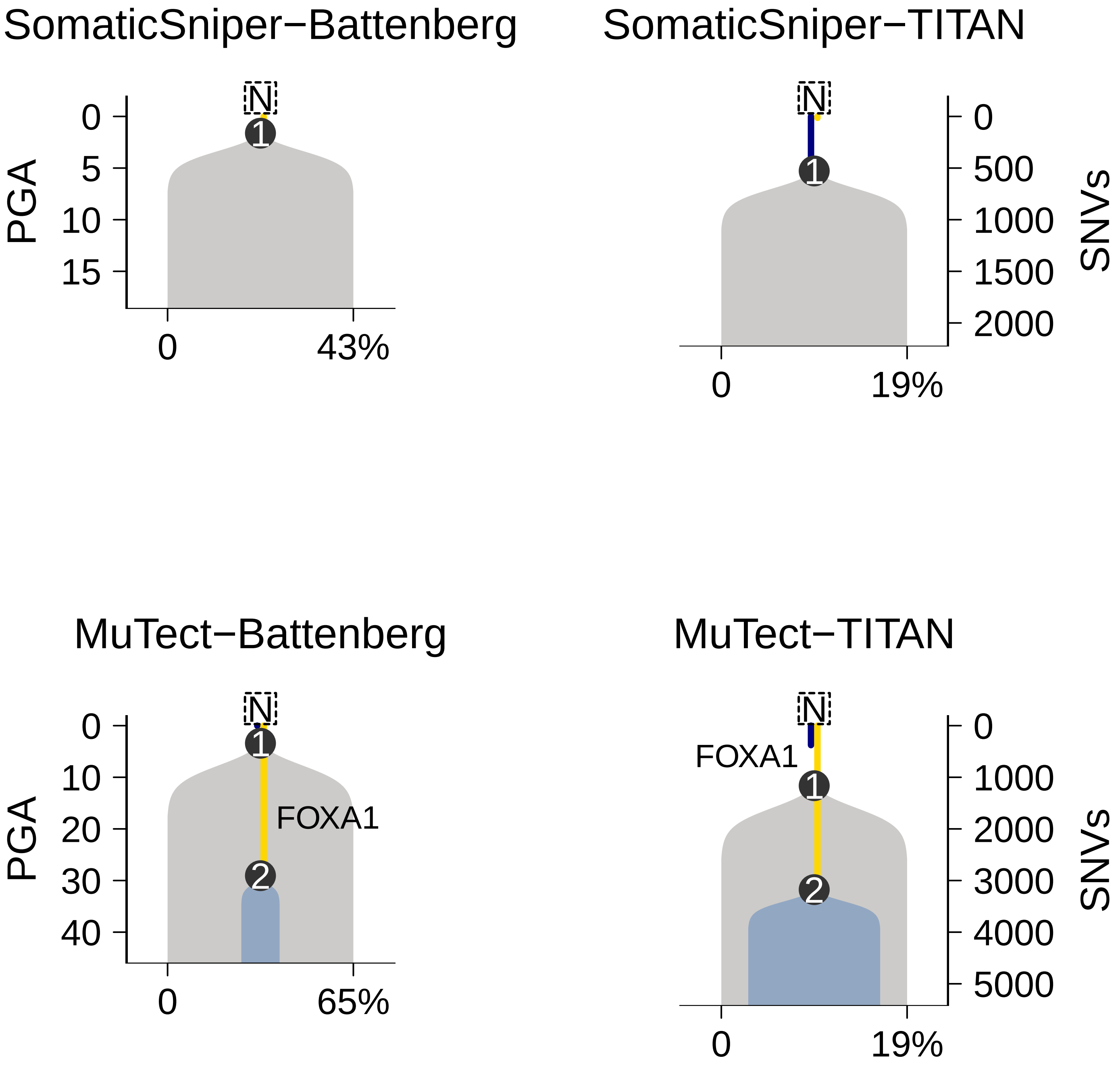
SomaticSniper−Battenberg
SomaticSniper−TITAN
N
1
PGA
0
5
10
15
0
43%
0
500
1000
1500
2000
SNVs
0
19%
MuTect−Battenberg
MuTect−TITAN
2
FOXA1
20
30
40
65%
3000
4000
5000

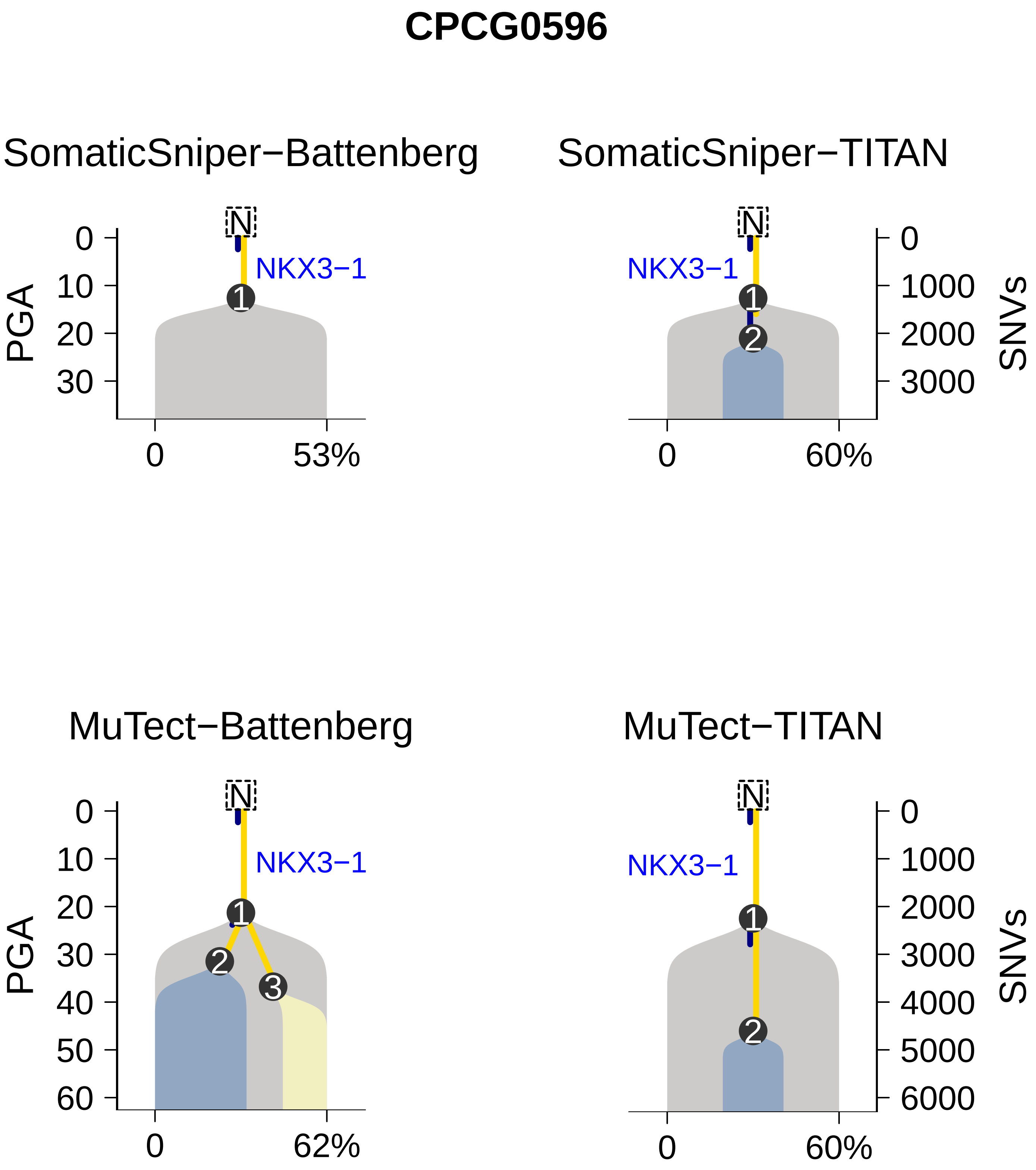
CPCG0596
SomaticSniper−Battenberg
SomaticSniper−TITAN
MuTect−Battenberg
MuTect−TITAN
N
NKX3−1
PGA
SNVs
0
10
20
30
40
50
60
1000
2000
3000
4000
5000
6000
53%
60%
62%
1
2
3

CPCG0597

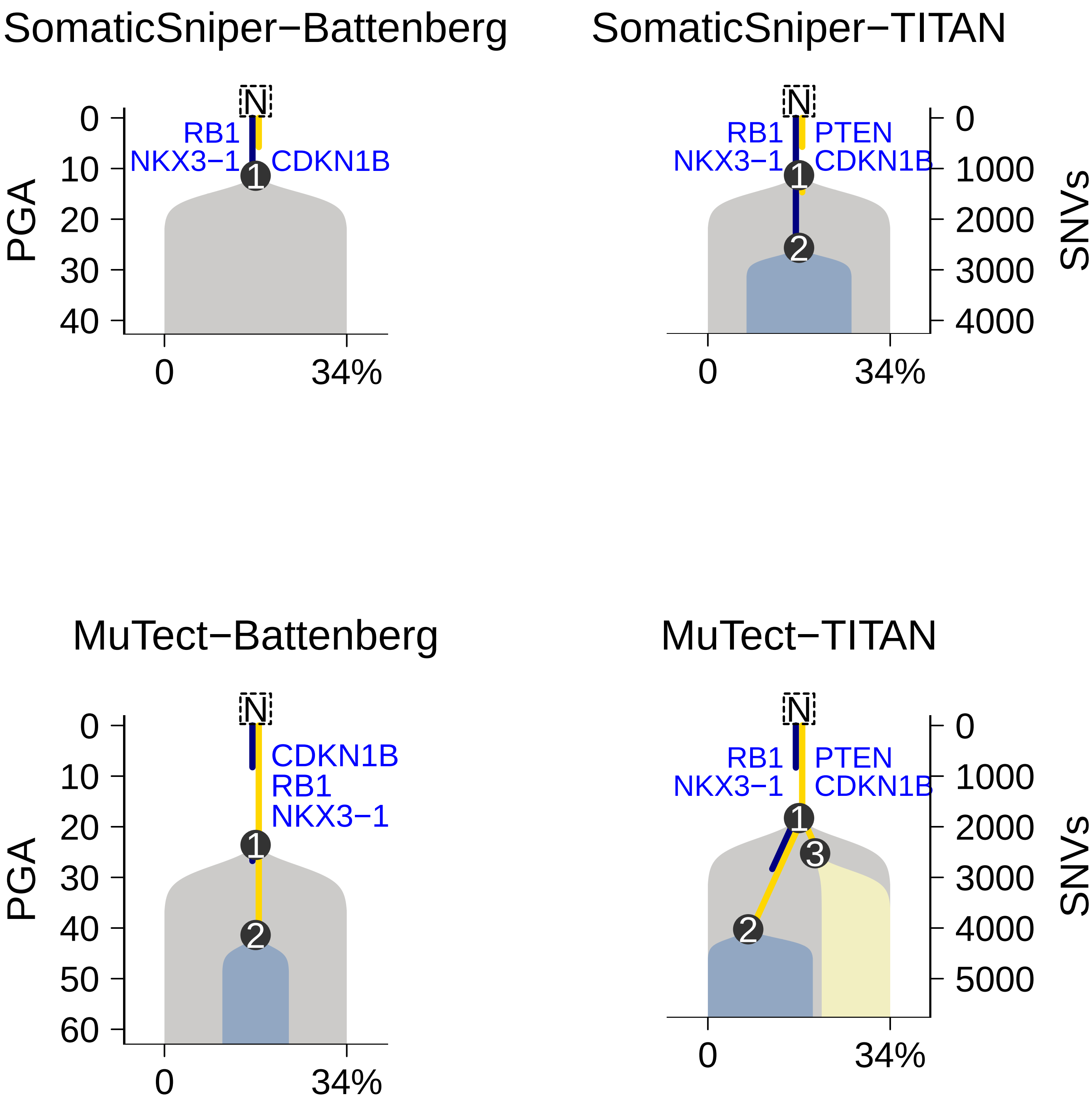
SomaticSniper−Battenberg
SomaticSniper−TITAN
MuTect−Battenberg
MuTect−TITAN
N
RB1
NKX3−1
CDKN1B
PTEN
PGA
SNVs
0
34%
0
10
20
30
40
50
60
1000
2000
3000
4000
5000
1
2
3

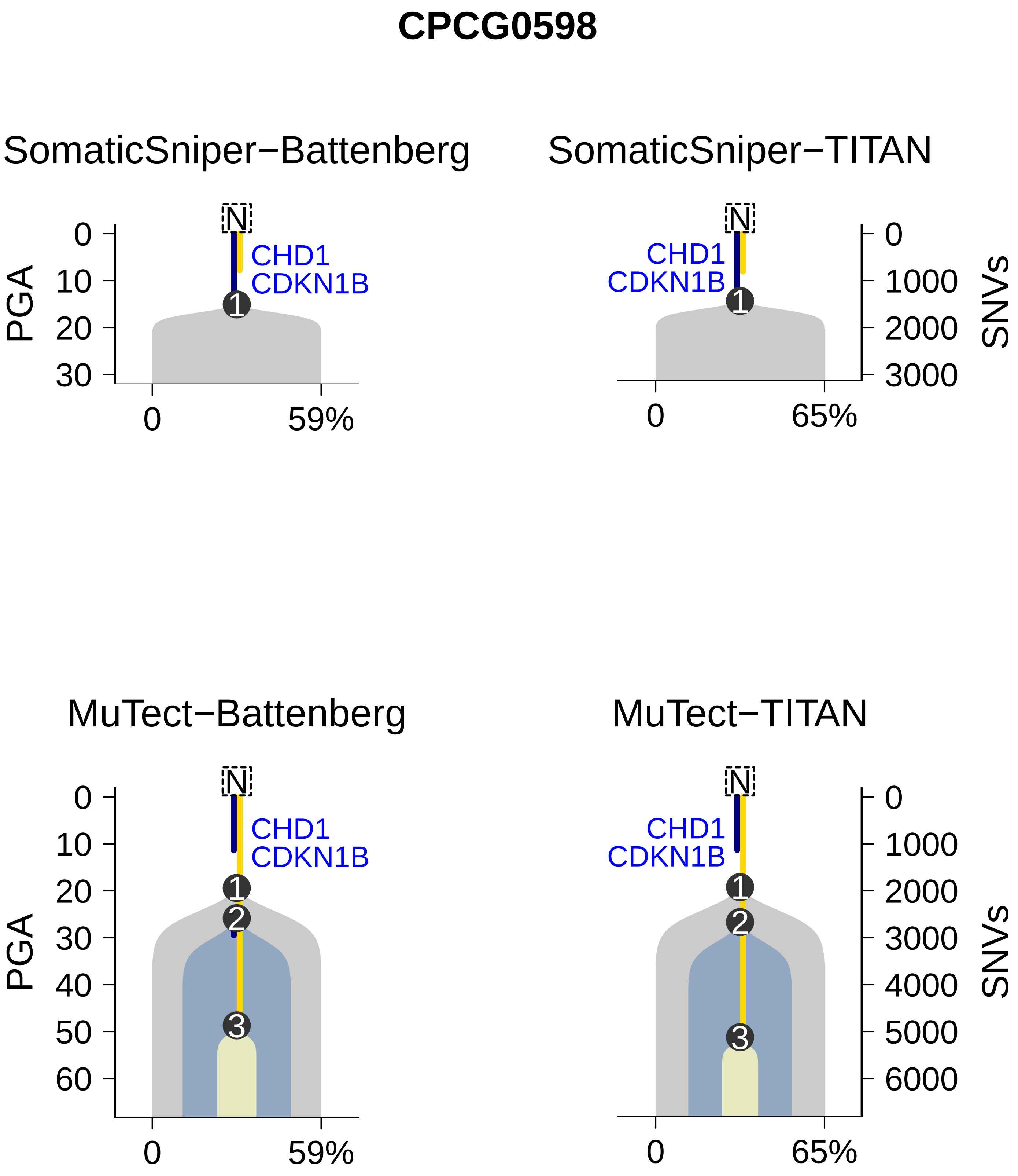
CPCG0598
SomaticSniper−Battenberg
SomaticSniper−TITAN
MuTect−Battenberg
MuTect−TITAN
N
CHD1
CDKN1B
1
2
3
PGA
SNVs
0
10
20
30
40
50
60
1000
2000
3000
4000
5000
6000
59%
65%

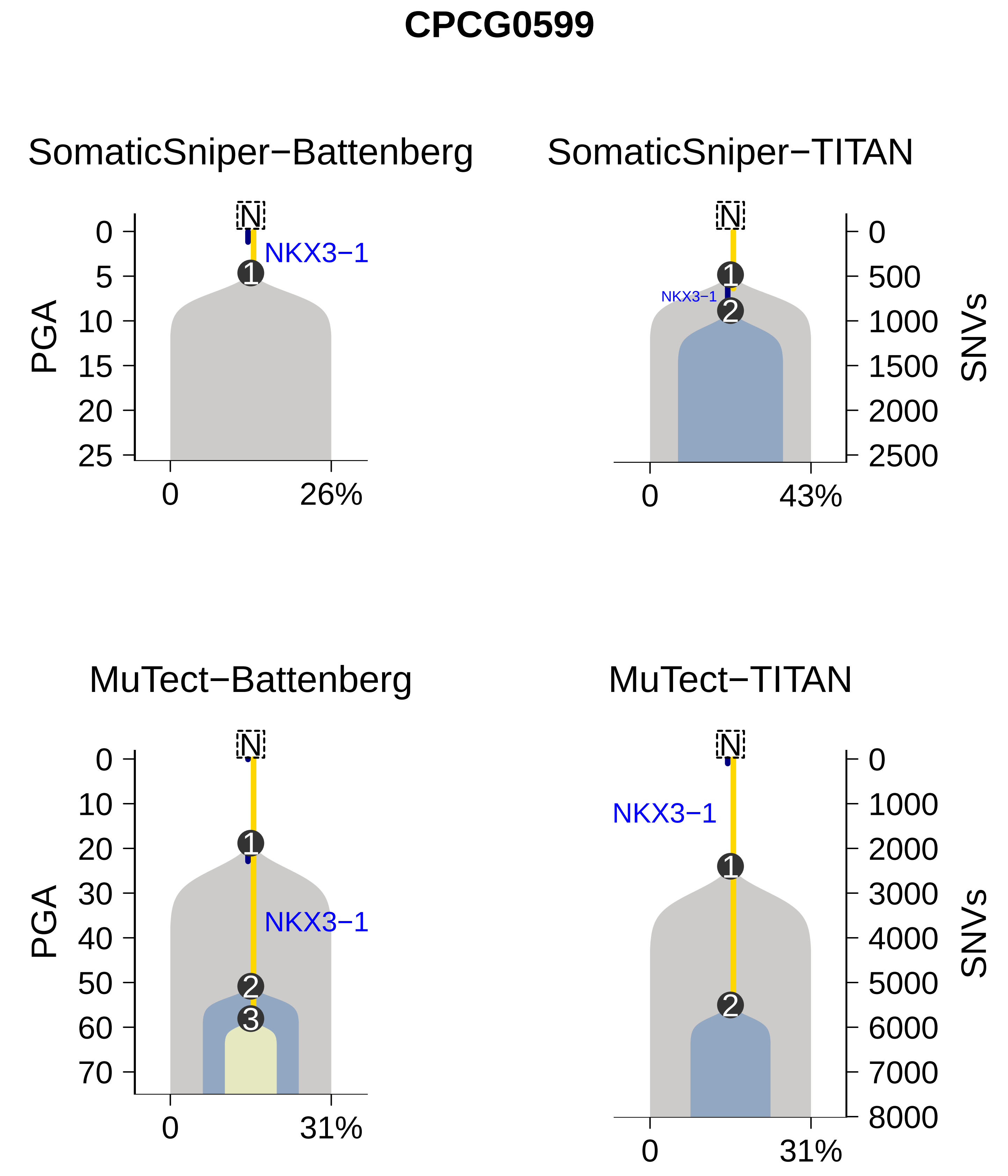
CPCG0599
SomaticSniper−Battenberg
SomaticSniper−TITAN
MuTect−Battenberg
MuTect−TITAN
N
NKX3−1
1
2
3
PGA
SNVs
0
5
10
15
20
25
26%
43%
31%
500
1000
1500
2000
2500
30
40
50
60
70
3000
4000
5000
6000
7000
8000

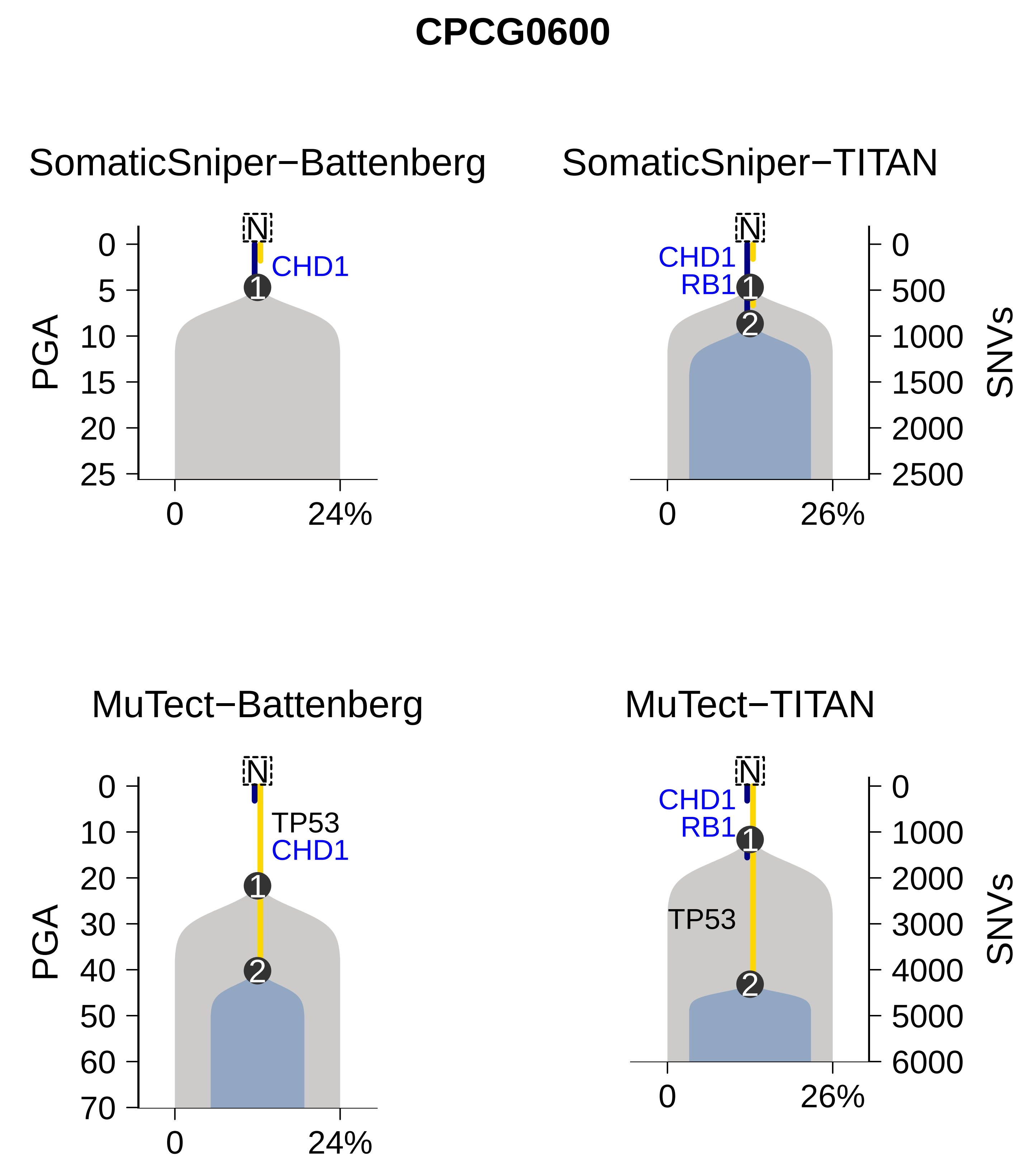
CPCG0600
SomaticSniper−Battenberg
SomaticSniper−TITAN
MuTect−Battenberg
MuTect−TITAN
N
CHD1
RB1
TP53
PGA
SNVs
0
5
10
15
20
25
30
40
50
60
70
500
1000
1500
2000
2500
3000
4000
5000
6000
24%
26%

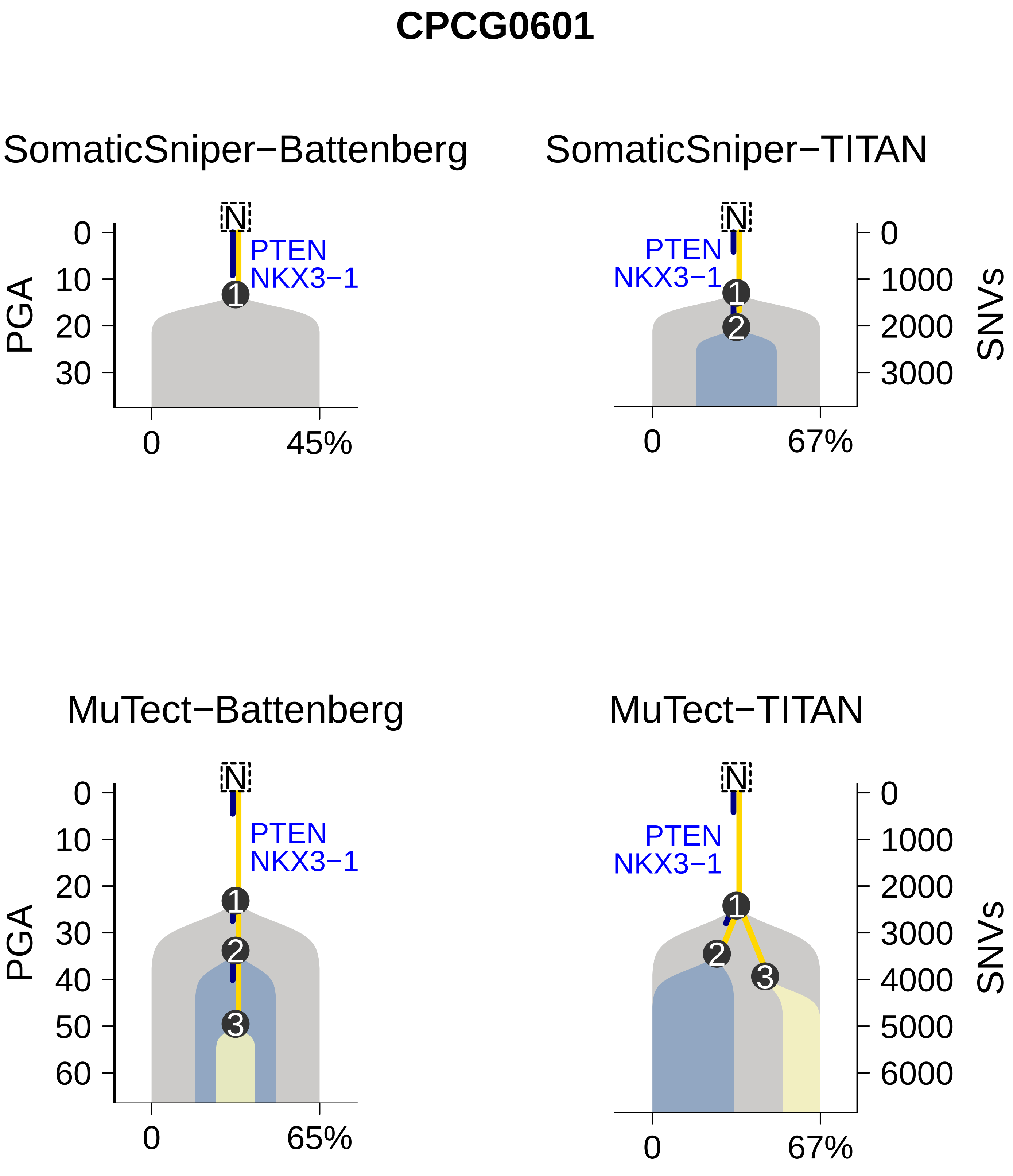
CPCG0601
SomaticSniper−Battenberg
SomaticSniper−TITAN
MuTect−Battenberg
MuTect−TITAN
N
1
2
3
PTEN
NKX3−1
PGA
SNVs
0
10
20
30
40
50
60
1000
2000
3000
4000
5000
6000
45%
65%
67%

CPCG0604

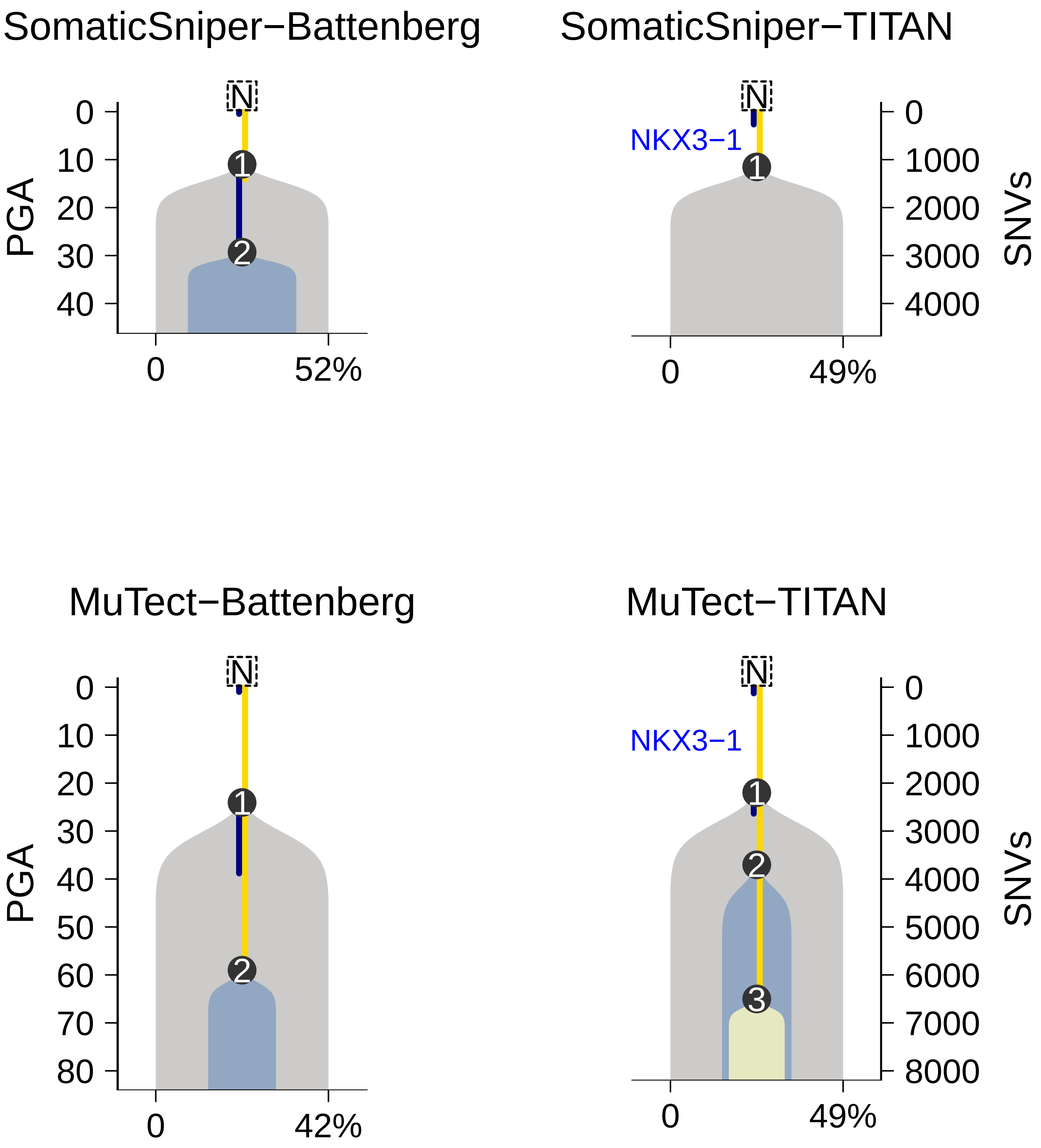

SomaticSniper−Battenberg
SomaticSniper−TITAN
MuTect−Battenberg
MuTect−TITAN
N
1
2
3
NKX3−1
PGA
SNVs
0
10
20
30
40
50
60
70
80
1000
2000
3000
4000
5000
6000
7000
8000
52%
49%
42%

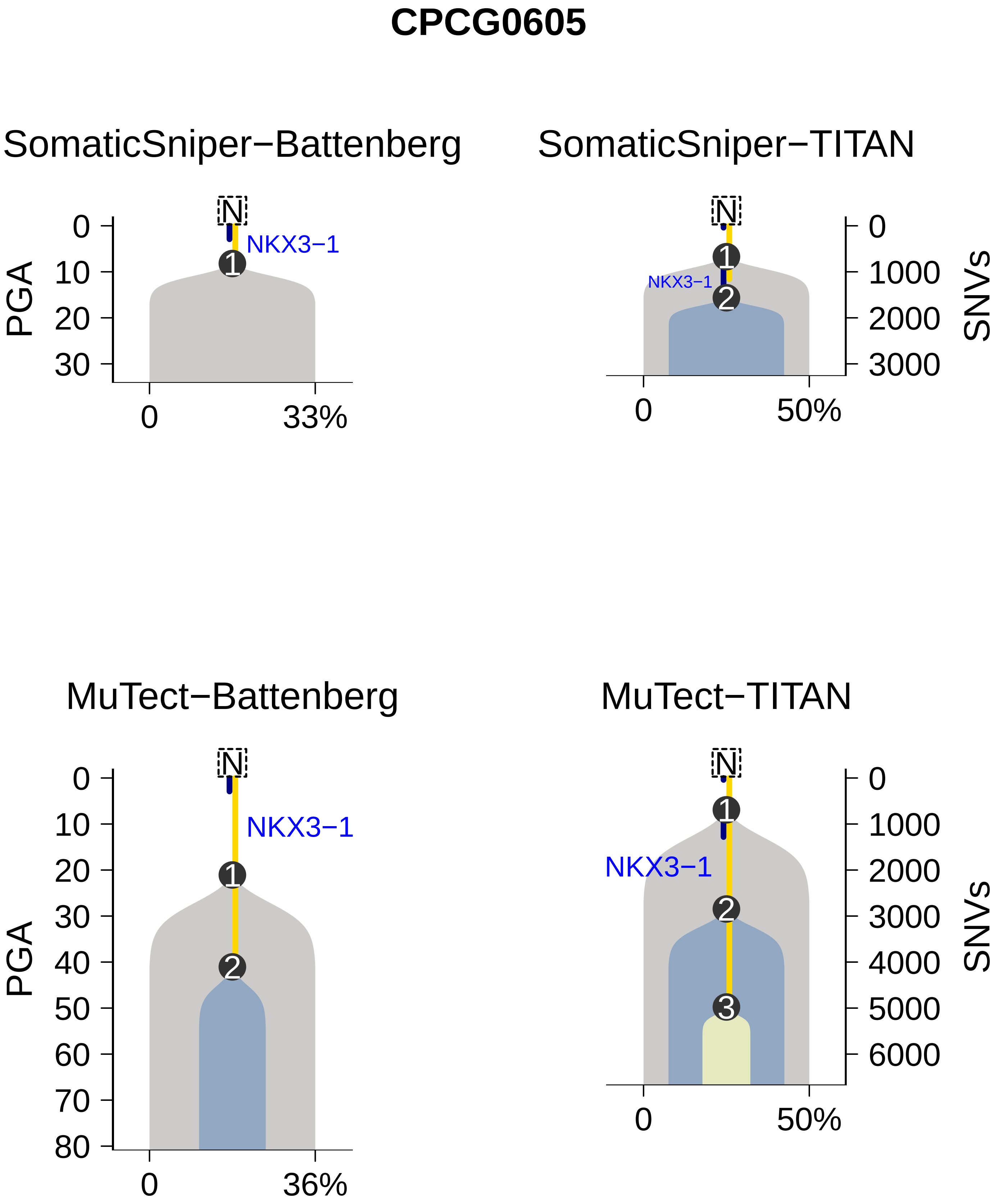

CPCG0605
SomaticSniper−Battenberg
SomaticSniper−TITAN
MuTect−Battenberg
MuTect−TITAN
N
1
2
3
NKX3−1
PGA
SNVs
0
10
20
30
40
50
60
70
80
1000
2000
3000
4000
5000
6000
33%
36%
50%

CPCG0606

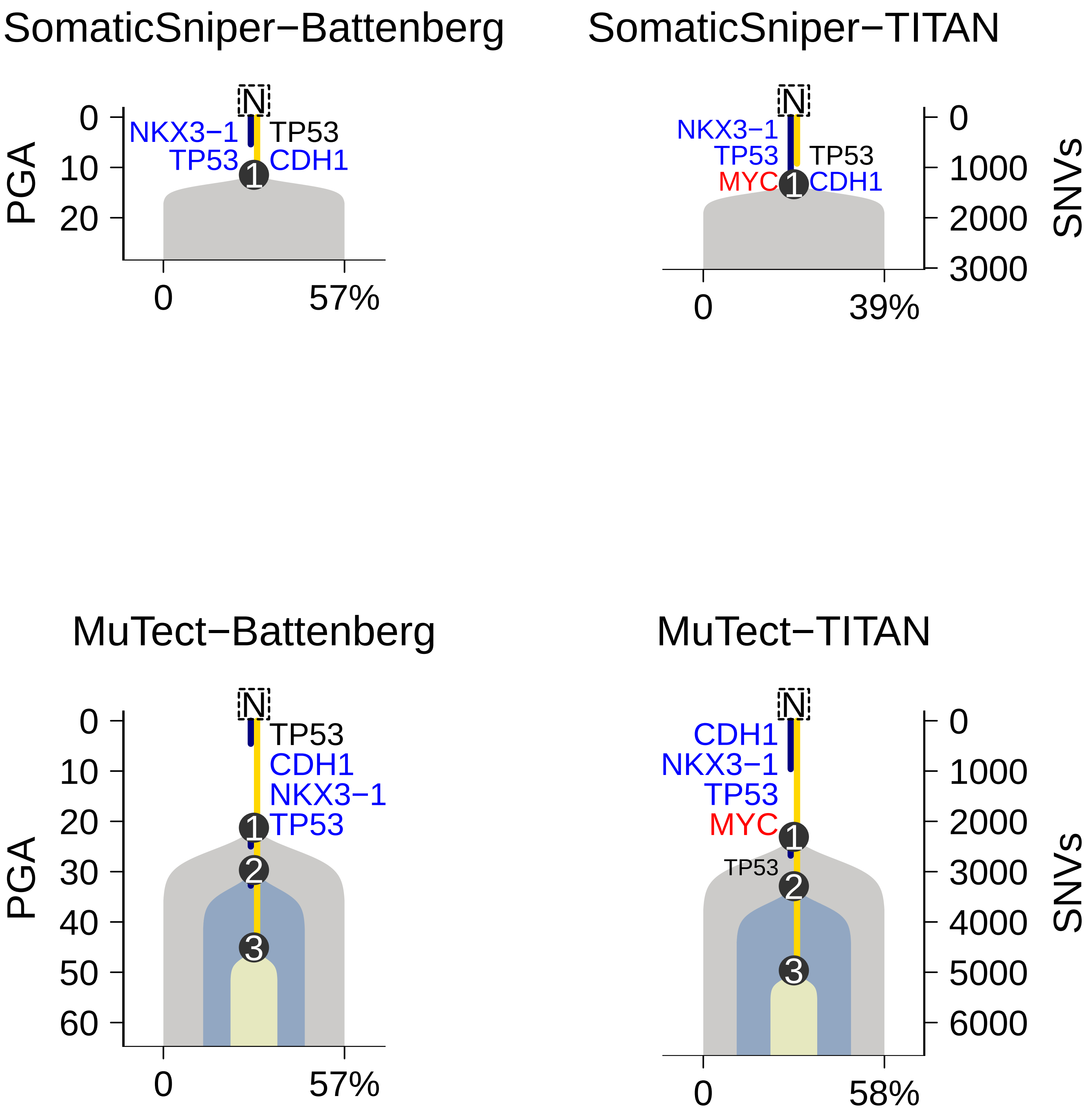

SomaticSniper−Battenberg
SomaticSniper−TITAN
MuTect−Battenberg
MuTect−TITAN
N
NKX3−1
TP53
CDH1
MYC
PGA
SNVs
0
10
20
30
40
50
60
57%
39%
58%
1000
2000
3000
4000
5000
6000
1
2
3

TCGA_5503_T1_wgs

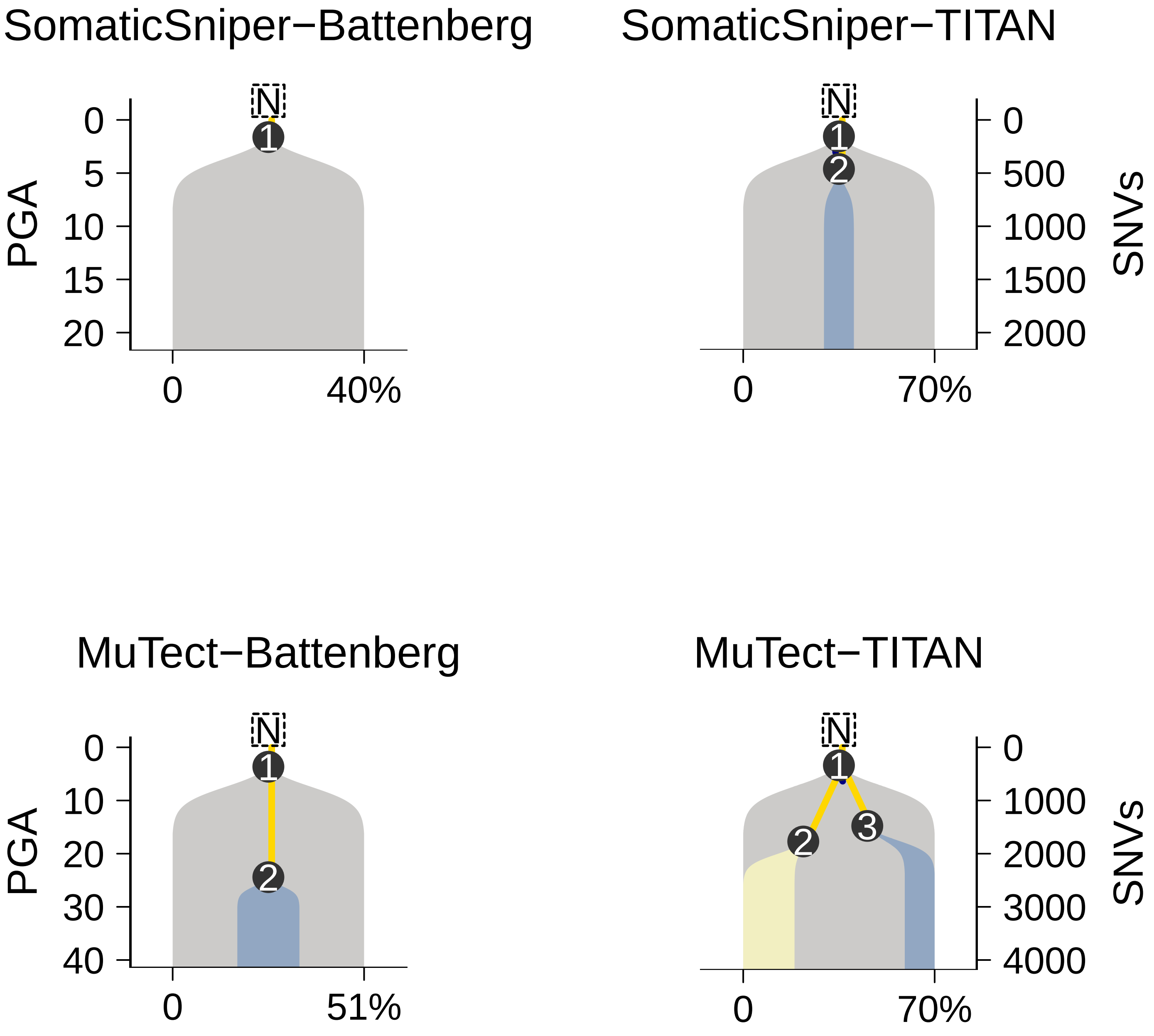

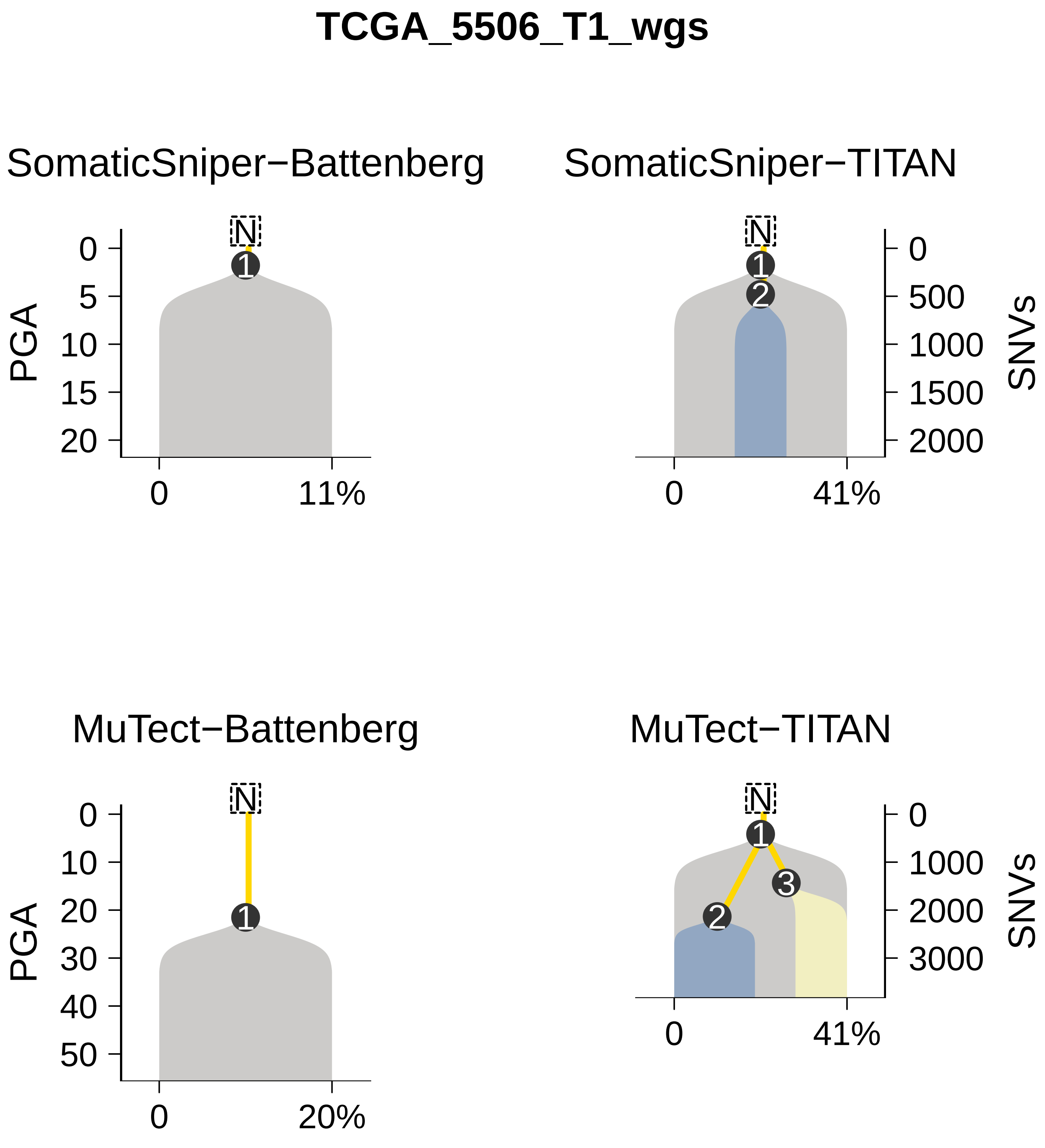
TCGA_5506_T1_wgs
SomaticSniper−Battenberg
SomaticSniper−TITAN
MuTect−Battenberg
MuTect−TITAN
N
1
2
3
PGA
SNVs
0
5
10
15
20
30
40
50
500
1000
1500
2000
3000
11%
41%
20%

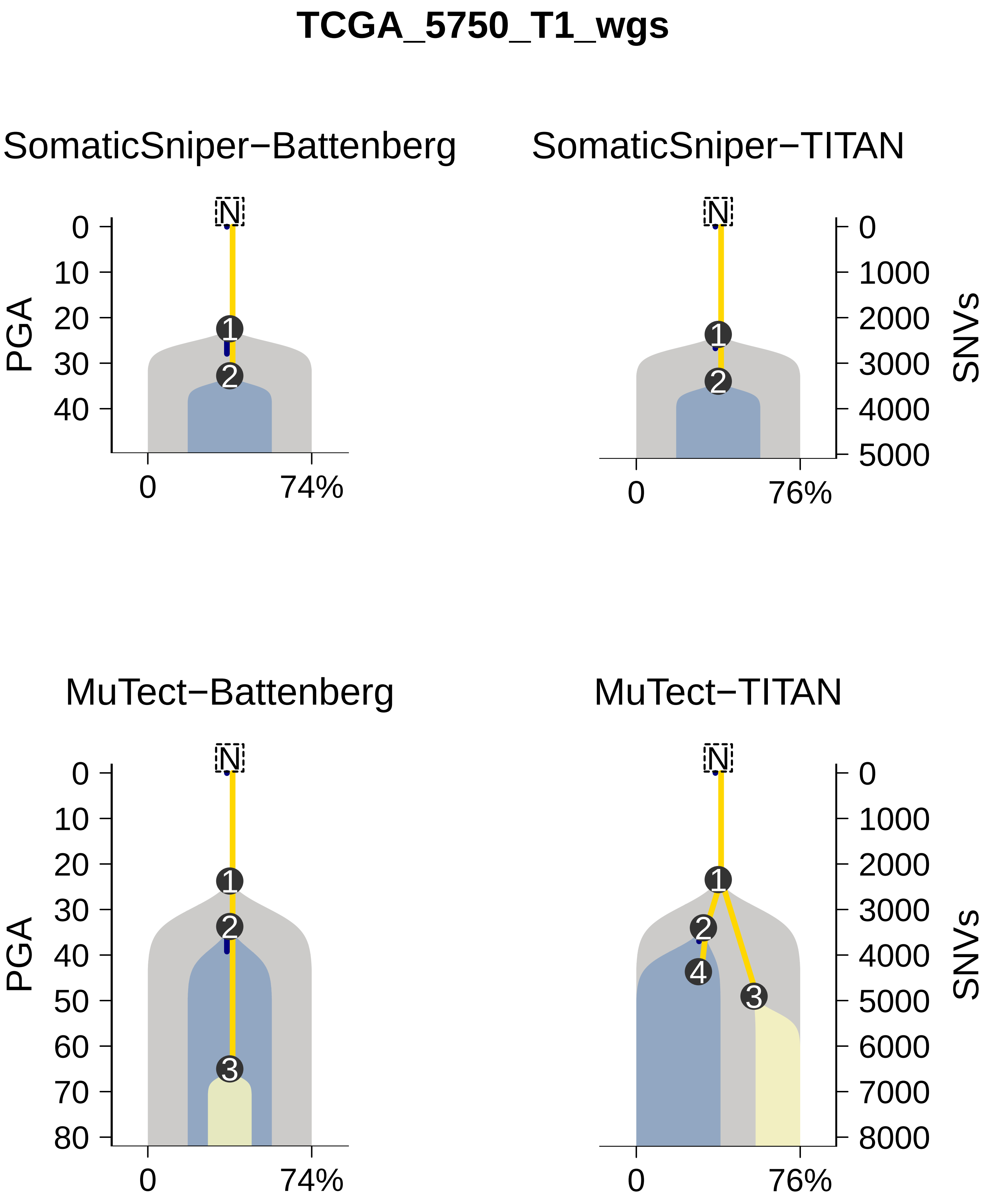
TCGA_5750_T1_wgs
SomaticSniper−Battenberg
SomaticSniper−TITAN
MuTect−Battenberg
MuTect−TITAN
PGA
SNVs
N
1
2
3
4
0
10
20
30
40
50
60
70
80
1000
2000
3000
4000
5000
6000
7000
8000
74%
76%

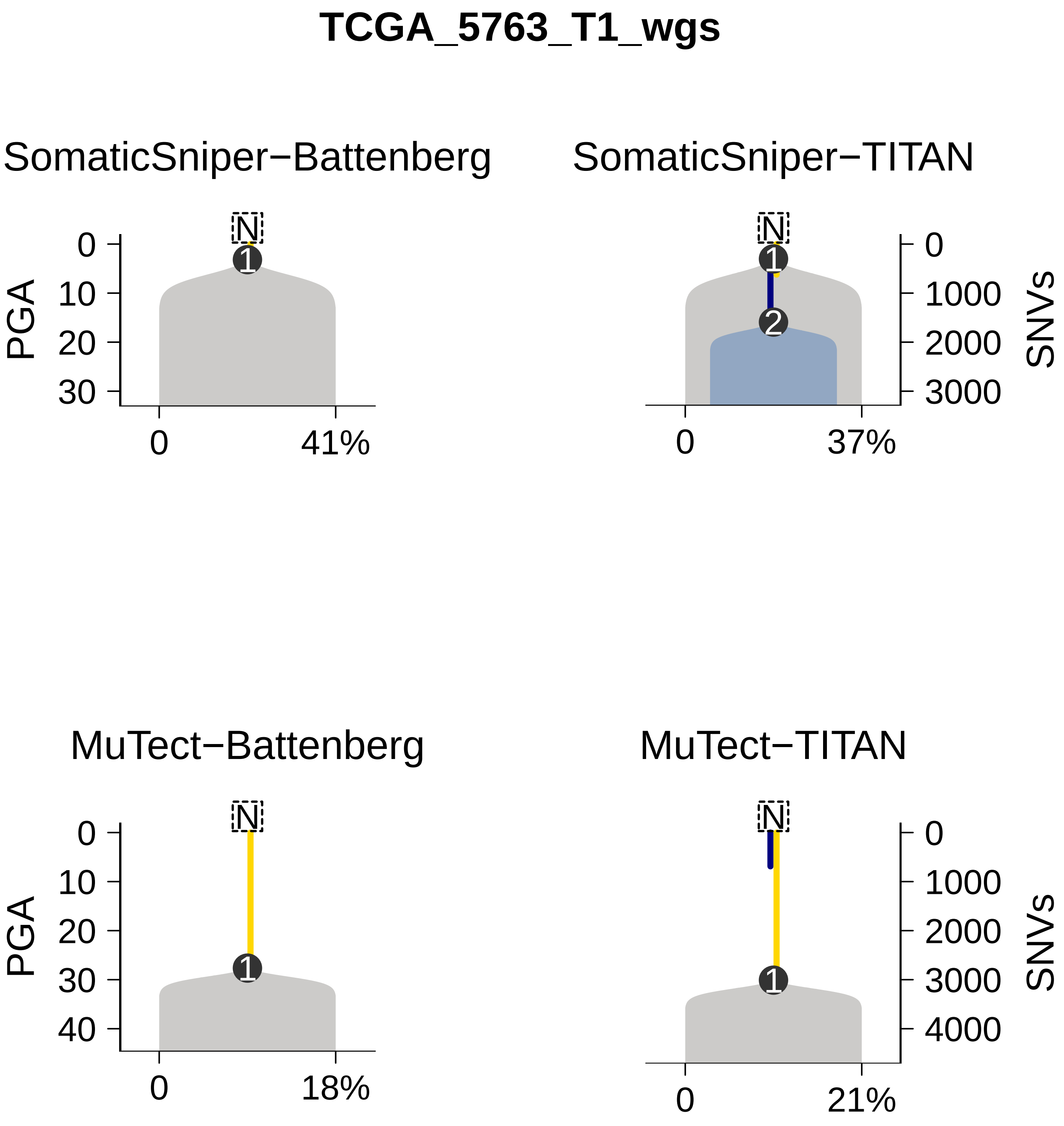
TCGA_5763_T1_wgs
SomaticSniper−Battenberg
SomaticSniper−TITAN
MuTect−Battenberg
MuTect−TITAN
N
1
2
PGA
SNVs
0
10
20
30
40
1000
2000
3000
4000
41%
37%
18%
21%

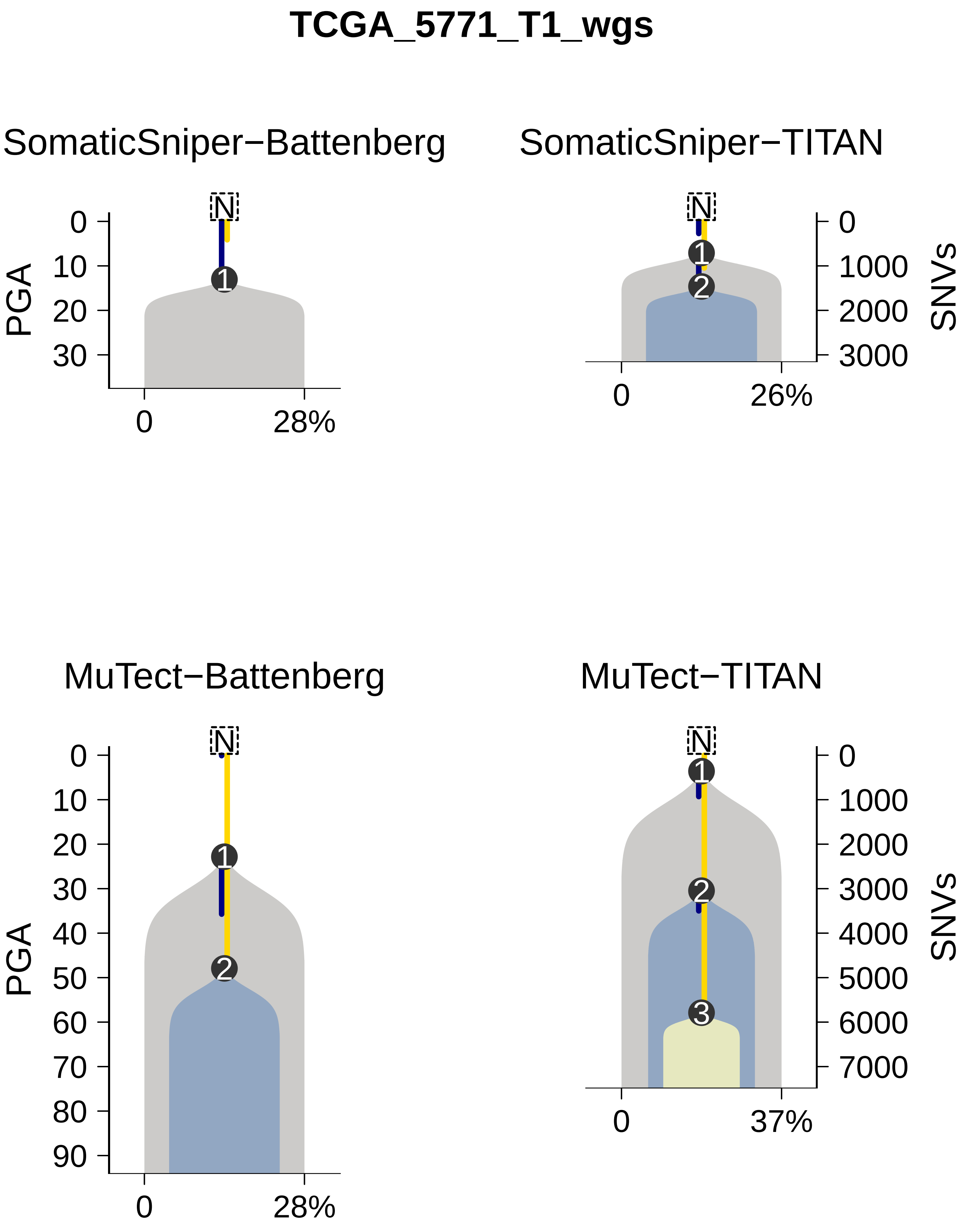
TCGA_5771_T1_wgs
SomaticSniper−Battenberg
SomaticSniper−TITAN
MuTect−Battenberg
MuTect−TITAN
N
1
2
3
PGA
SNVs
0
10
20
30
40
50
60
70
80
90
1000
2000
3000
4000
5000
6000
7000
28%
26%
37%

TCGA_5788_T1_wgs

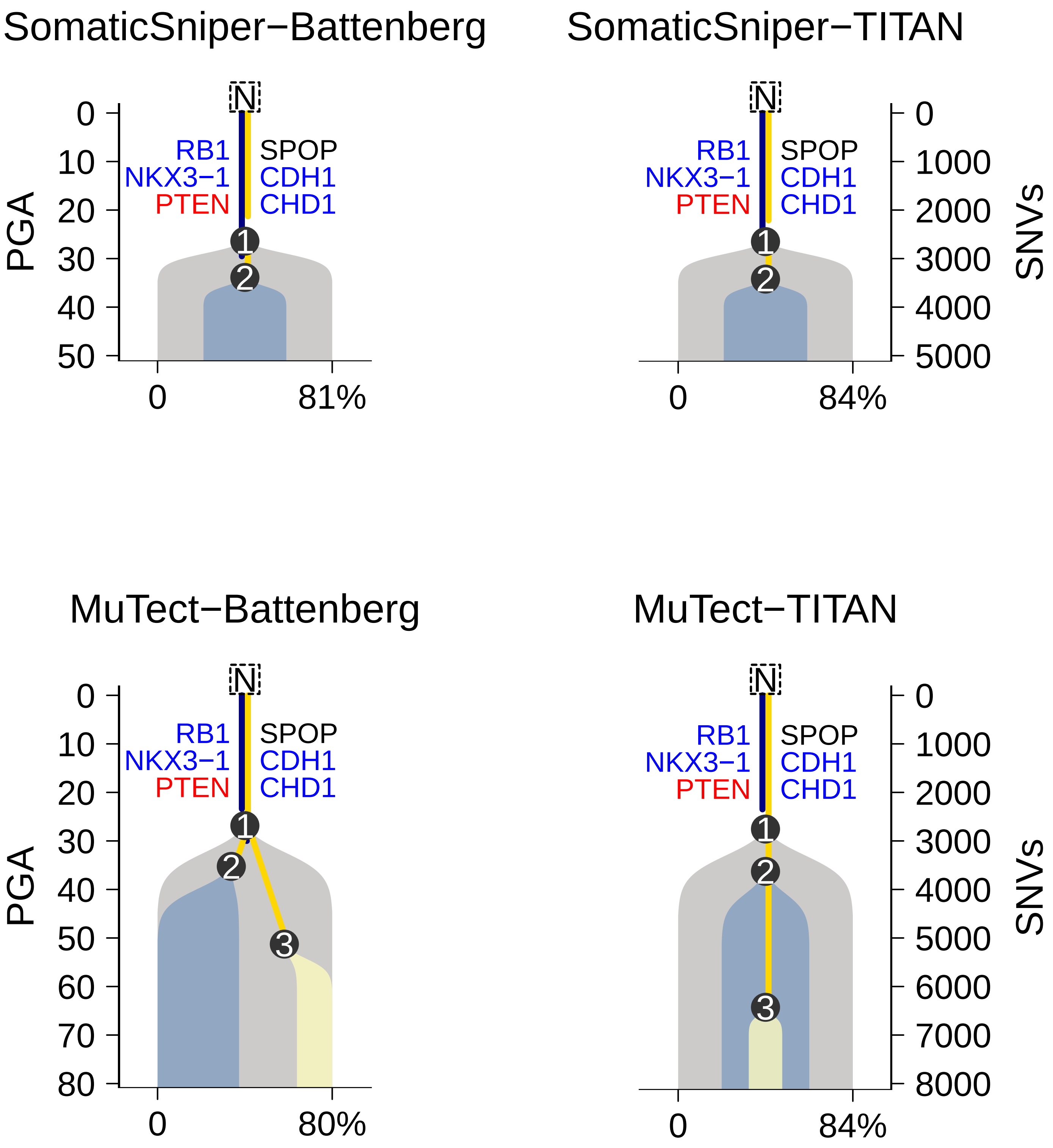

TCGA_5789_T1_wgs

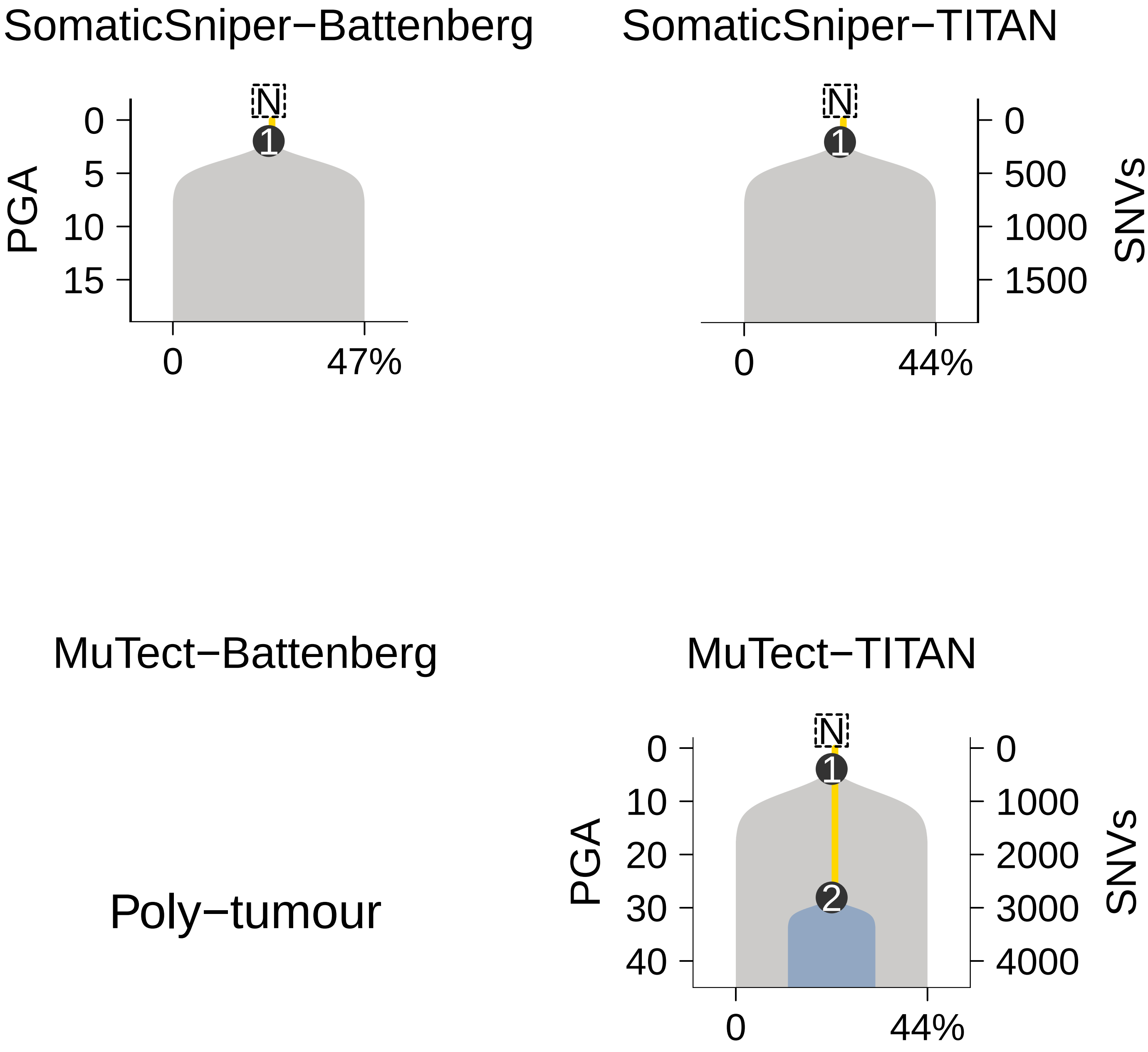

TCGA_6336_T1_wgs

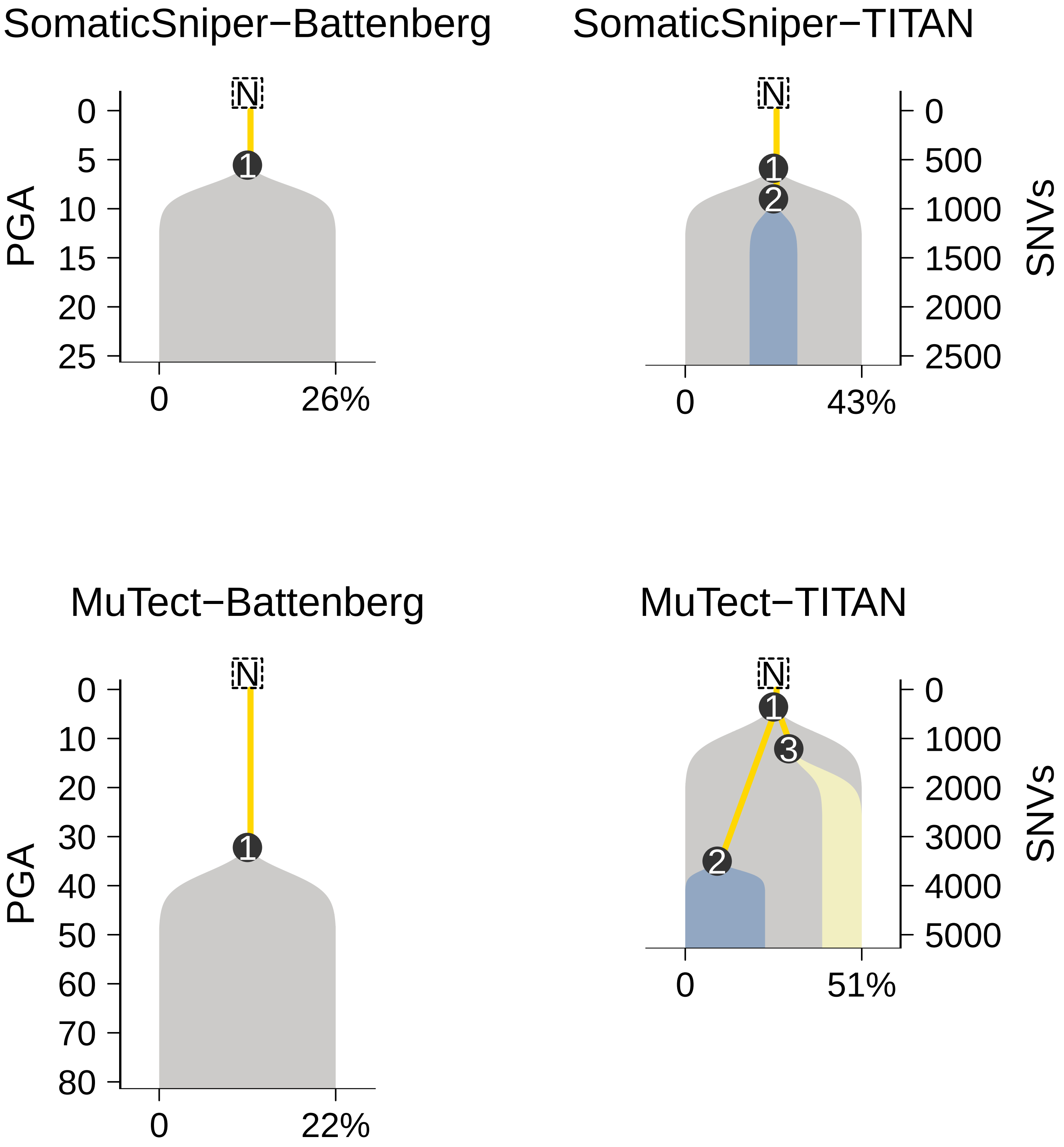

TCGA_6365_T1_wgs

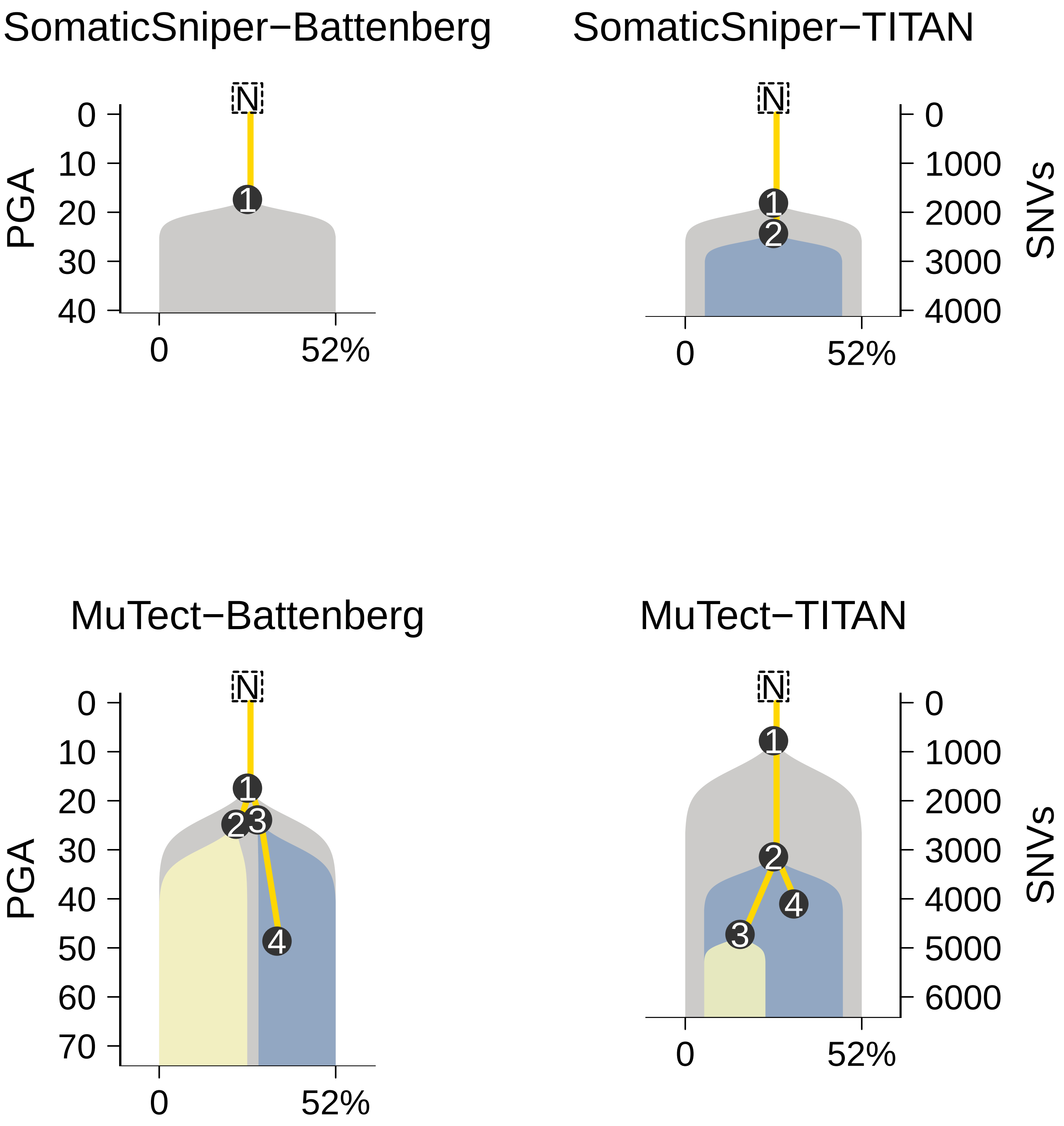

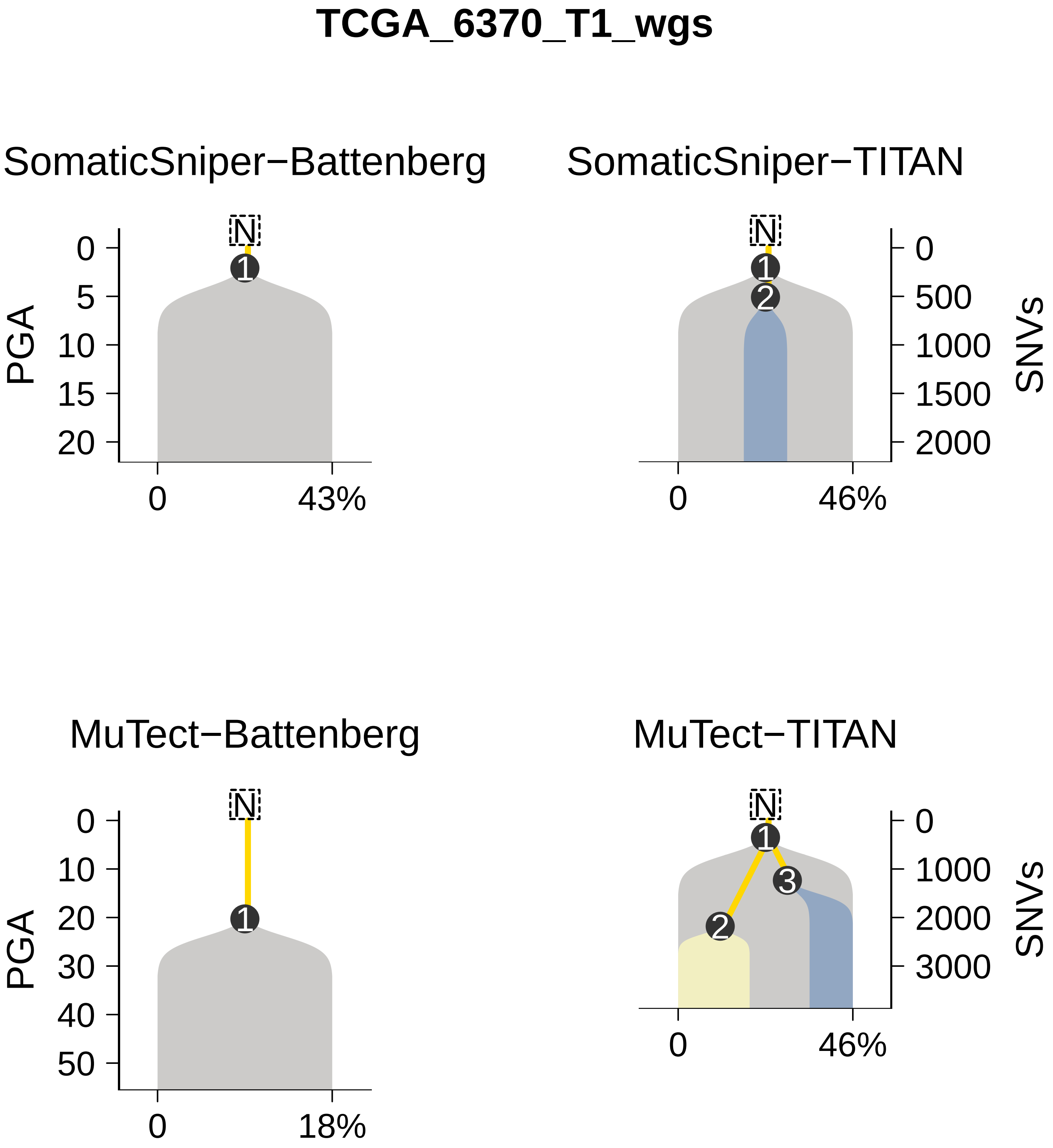
TCGA_6370_T1_wgs
SomaticSniper−Battenberg
SomaticSniper−TITAN
MuTect−Battenberg
MuTect−TITAN
PGA
SNVs
N
1
2
3
0
5
10
15
20
30
40
50
500
1000
1500
2000
3000
43%
46%
18%

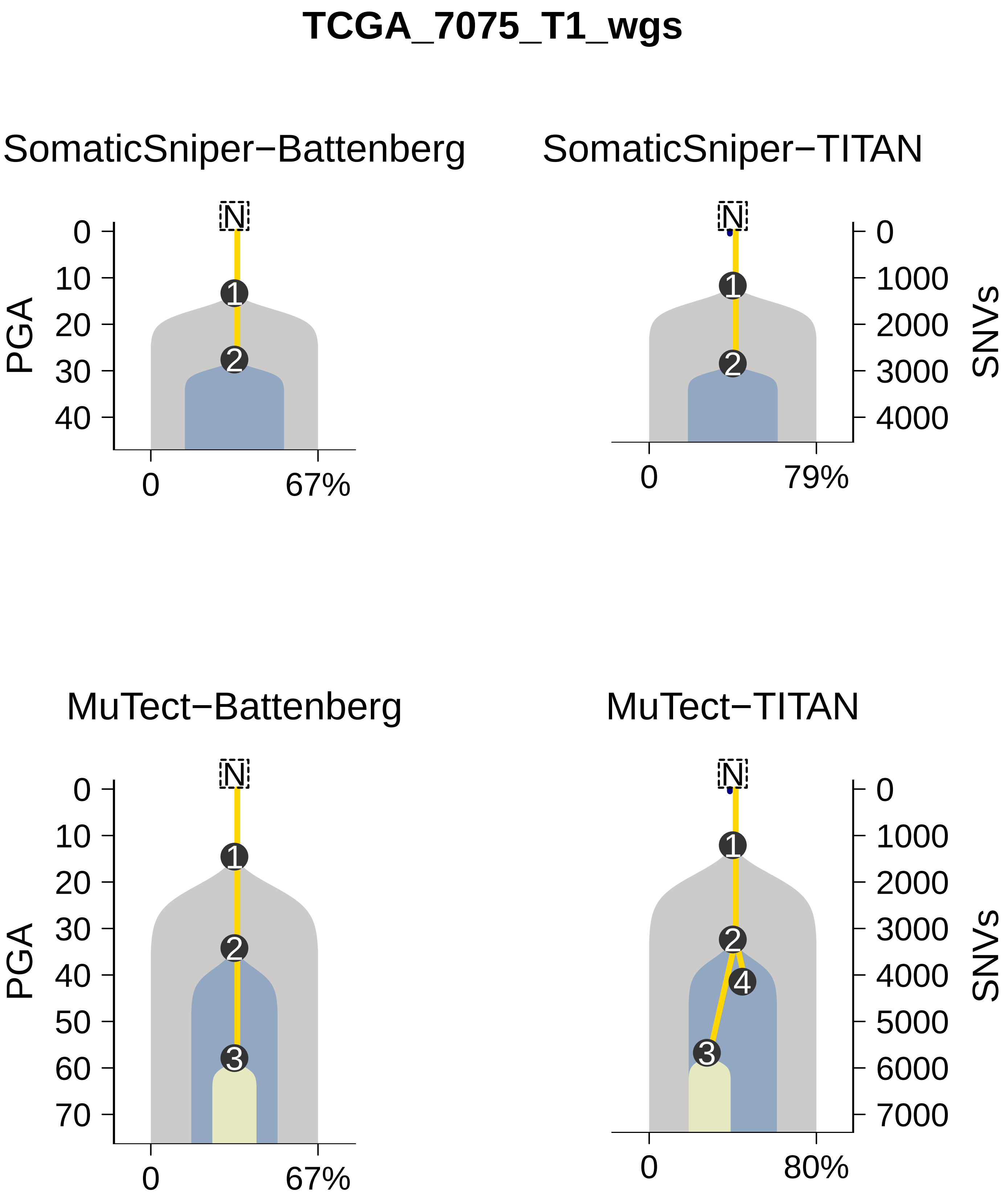
TCGA_7075_T1_wgs
SomaticSniper−Battenberg
SomaticSniper−TITAN
MuTect−Battenberg
MuTect−TITAN
PGA
SNVs
0
10
20
30
40
50
60
70
1000
2000
3000
4000
5000
6000
7000
67%
79%
80%
N
1
2
3
4

TCGA_7079_T1_wgs

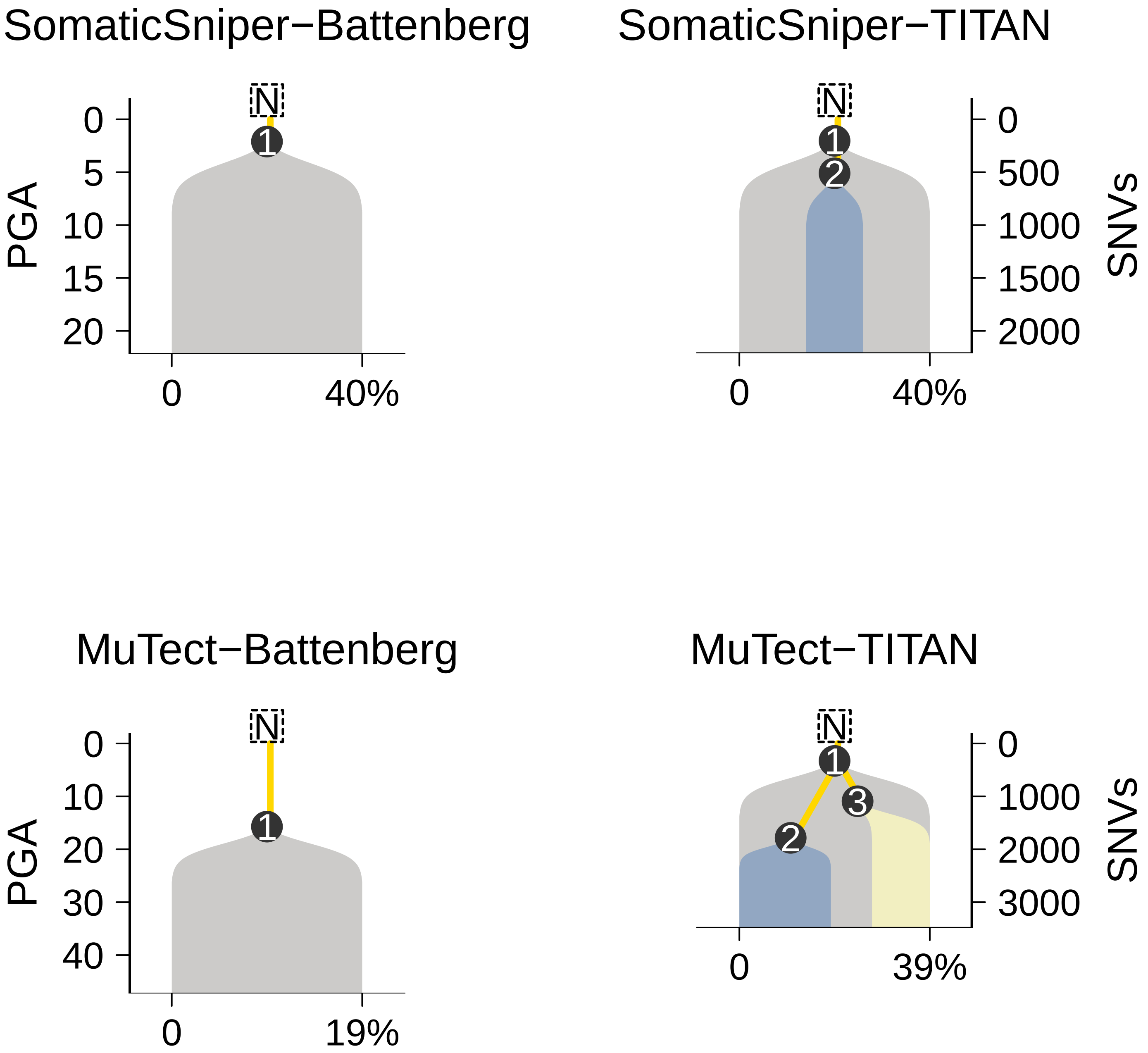

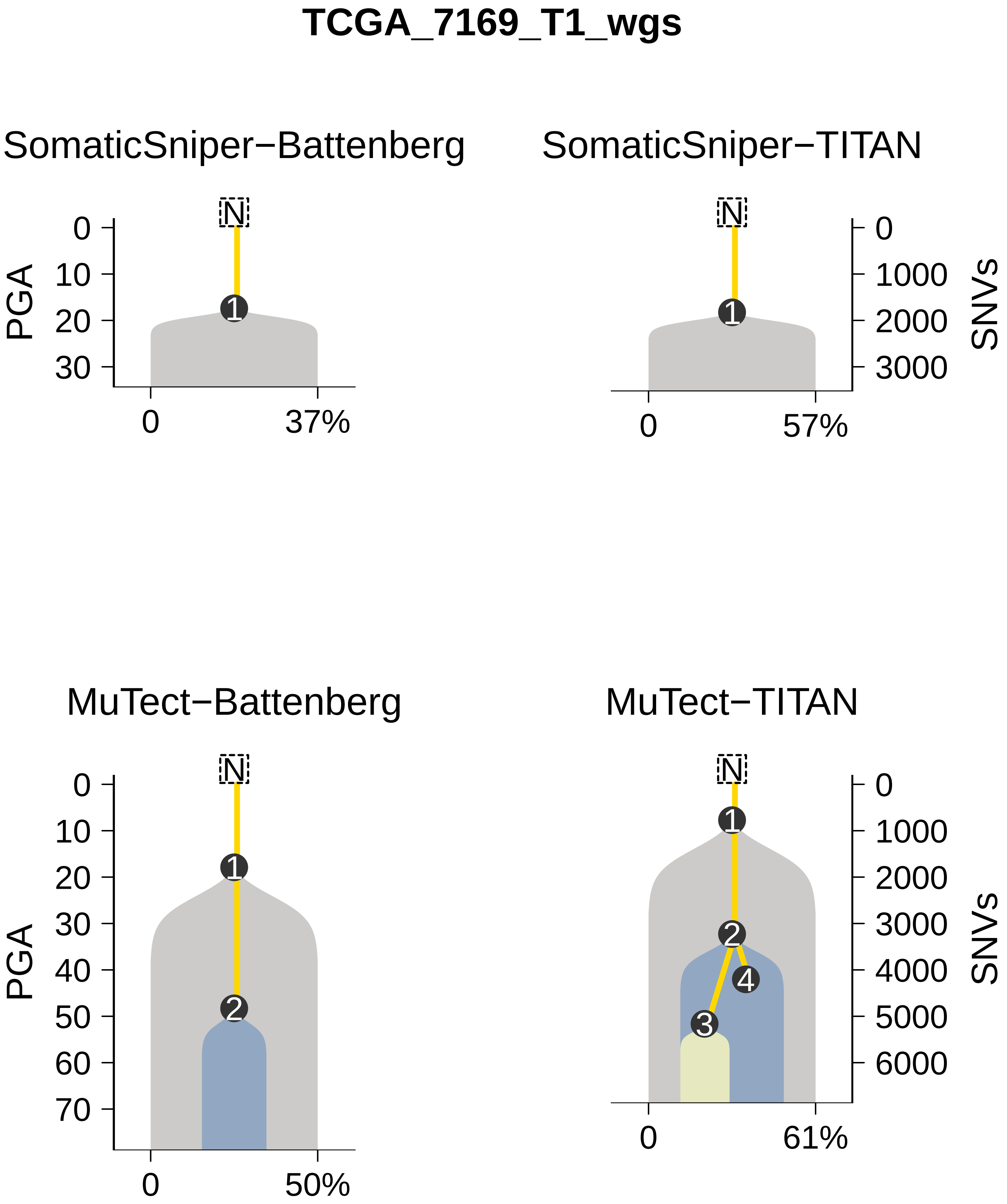
TCGA_7169_T1_wgs
SomaticSniper−Battenberg
SomaticSniper−TITAN
MuTect−Battenberg
MuTect−TITAN
N
PGA
SNVs
0
10
20
30
40
50
60
70
1000
2000
3000
4000
5000
6000
37%
57%
50%
61%
1
2
3
4

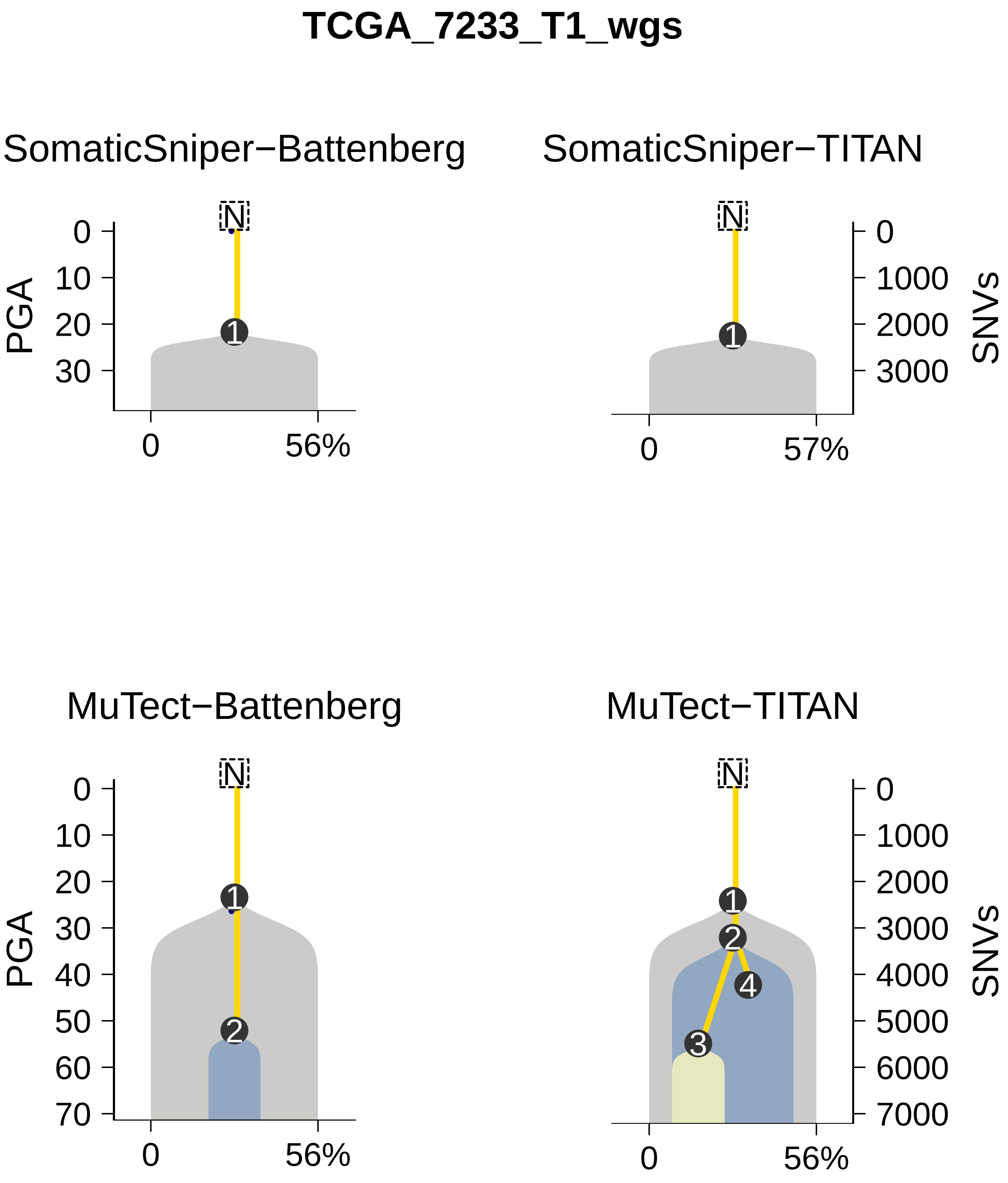
TCGA_7233_T1_wgs
SomaticSniper−Battenberg
SomaticSniper−TITAN
MuTect−Battenberg
MuTect−TITAN
PGA
SNVs
N
1
2
3
4
0
10
20
30
40
50
60
70
1000
2000
3000
4000
5000
6000
7000
56%
57%

TCGA_7522_T1_wgs

SomaticSniper−Battenberg

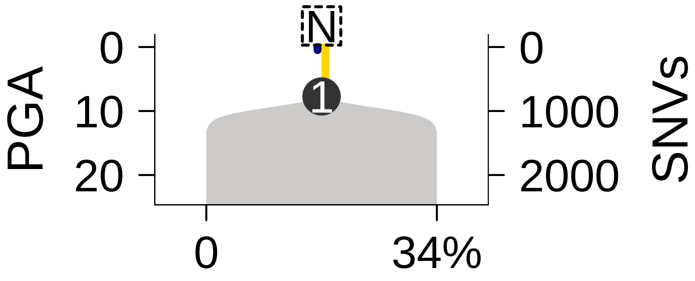

SomaticSniper−TITAN

Poly−tumour

MuTect−Battenberg

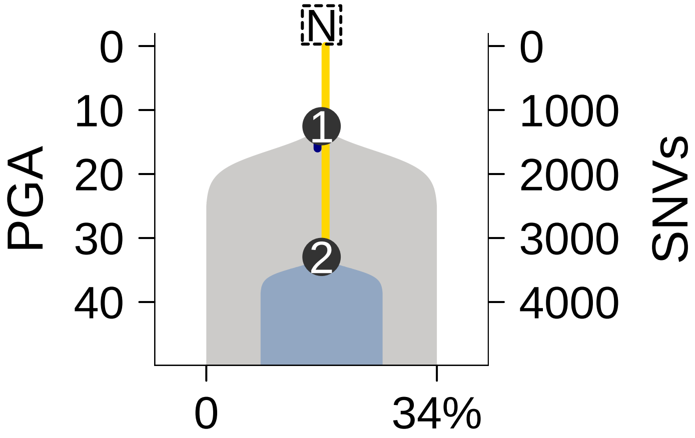

MuTect−TITAN

Poly−tumour

TCGA_7737_T1_wgs

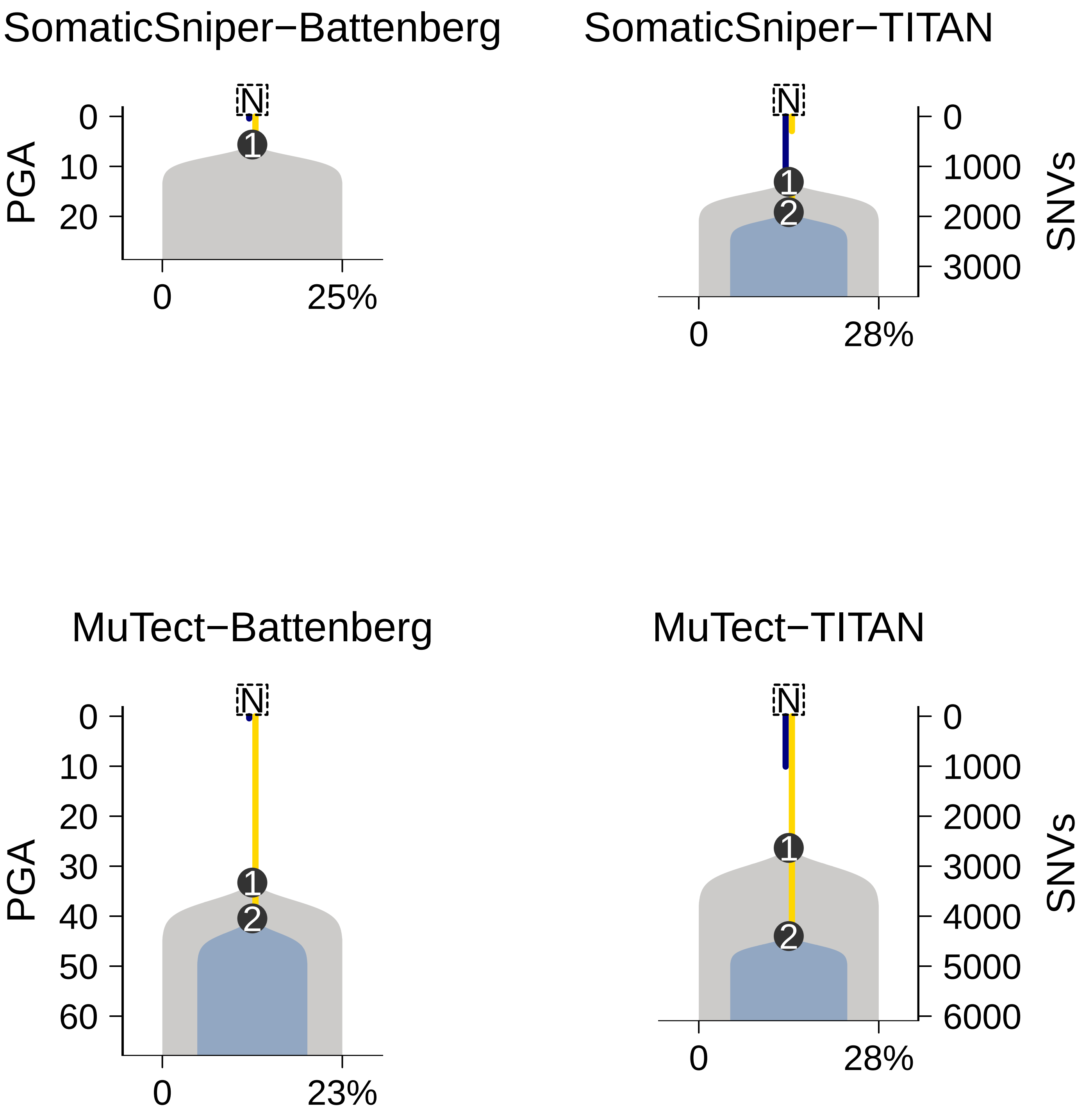

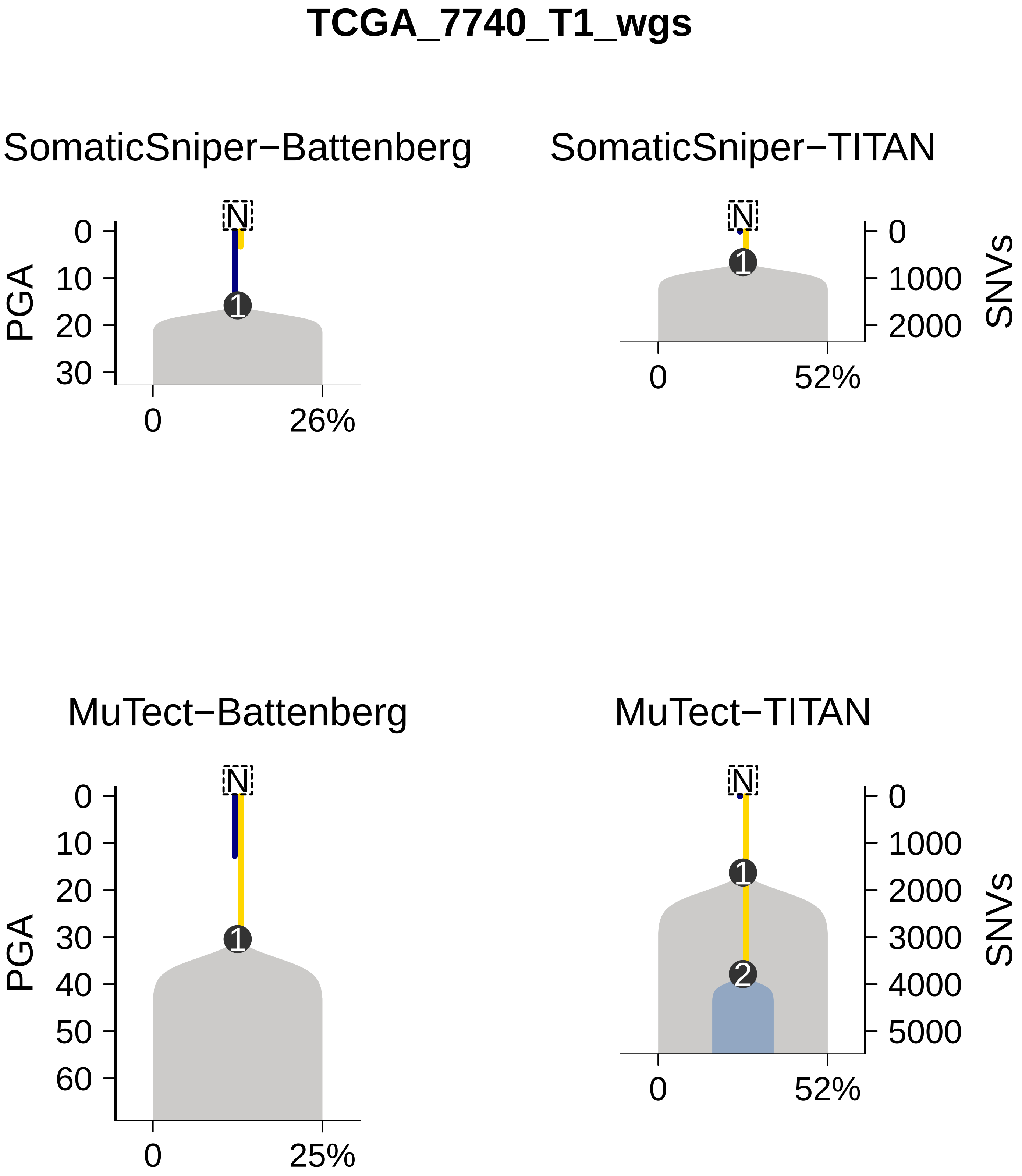

TCGA_7740_T1_wgs
SomaticSniper−Battenberg
SomaticSniper−TITAN
MuTect−Battenberg
MuTect−TITAN
N
1
2
PGA
SNVs
0
10
20
30
40
50
60
1000
2000
3000
4000
5000
26%
52%
25%

TCGA_7744_T1_wgs

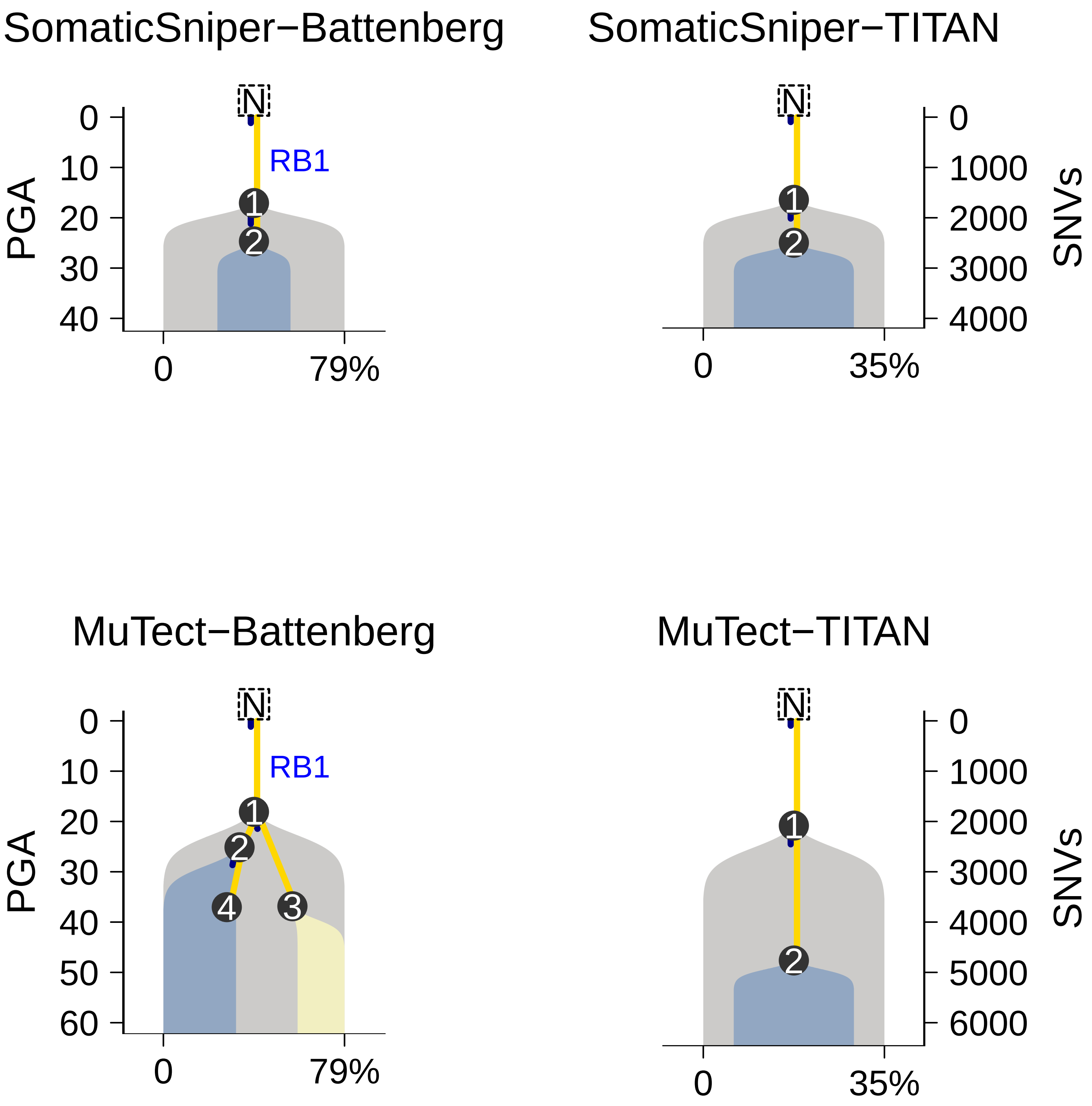

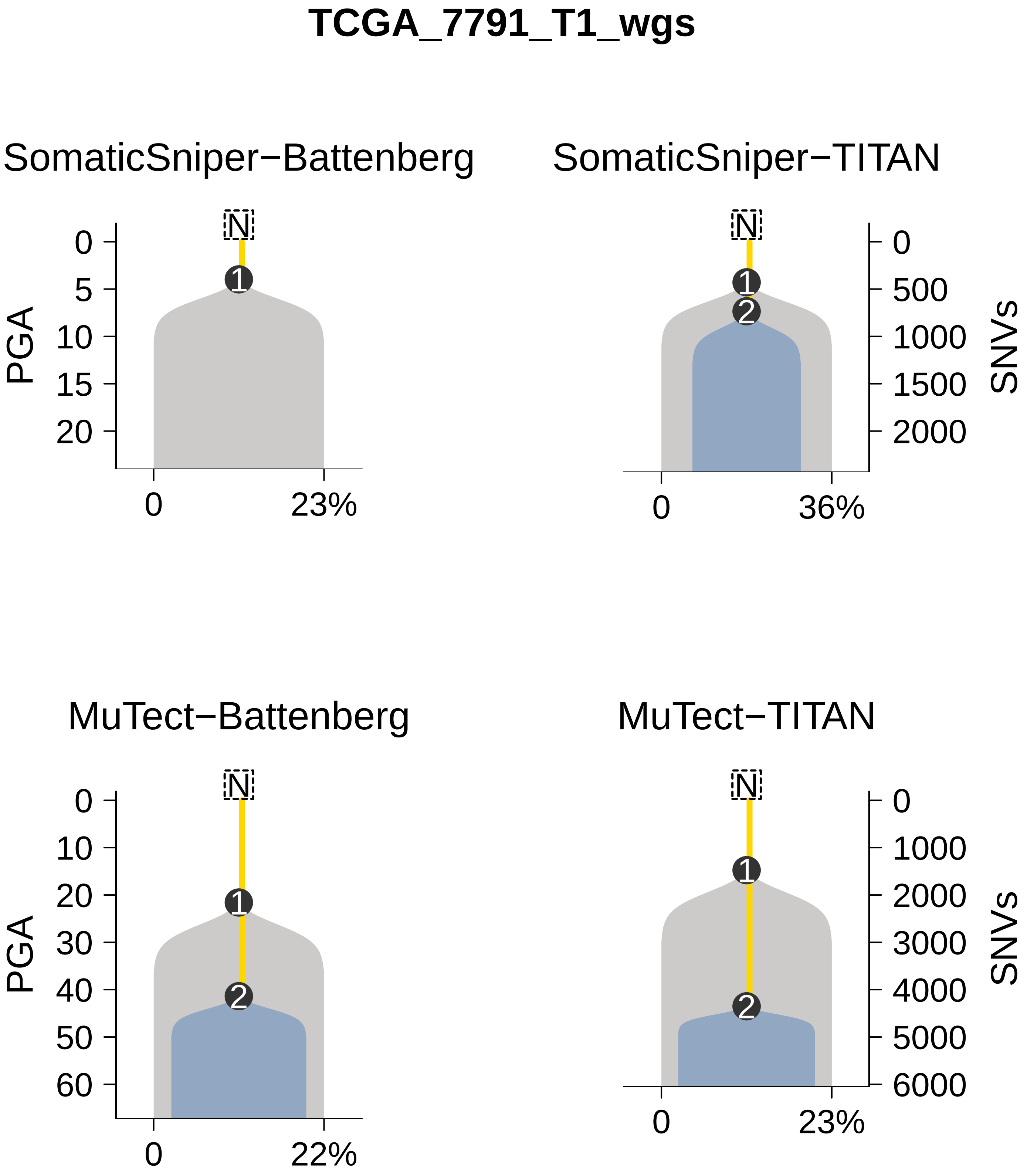
TCGA_7791_T1_wgs
SomaticSniper−Battenberg
SomaticSniper−TITAN
MuTect−Battenberg
MuTect−TITAN
PGA
SNVs
N
1
2
0
5
10
15
20
500
1000
1500
2000
23%
36%
22%
30
40
50
60
3000
4000
5000
6000

TCGA_8258_T1_wgs

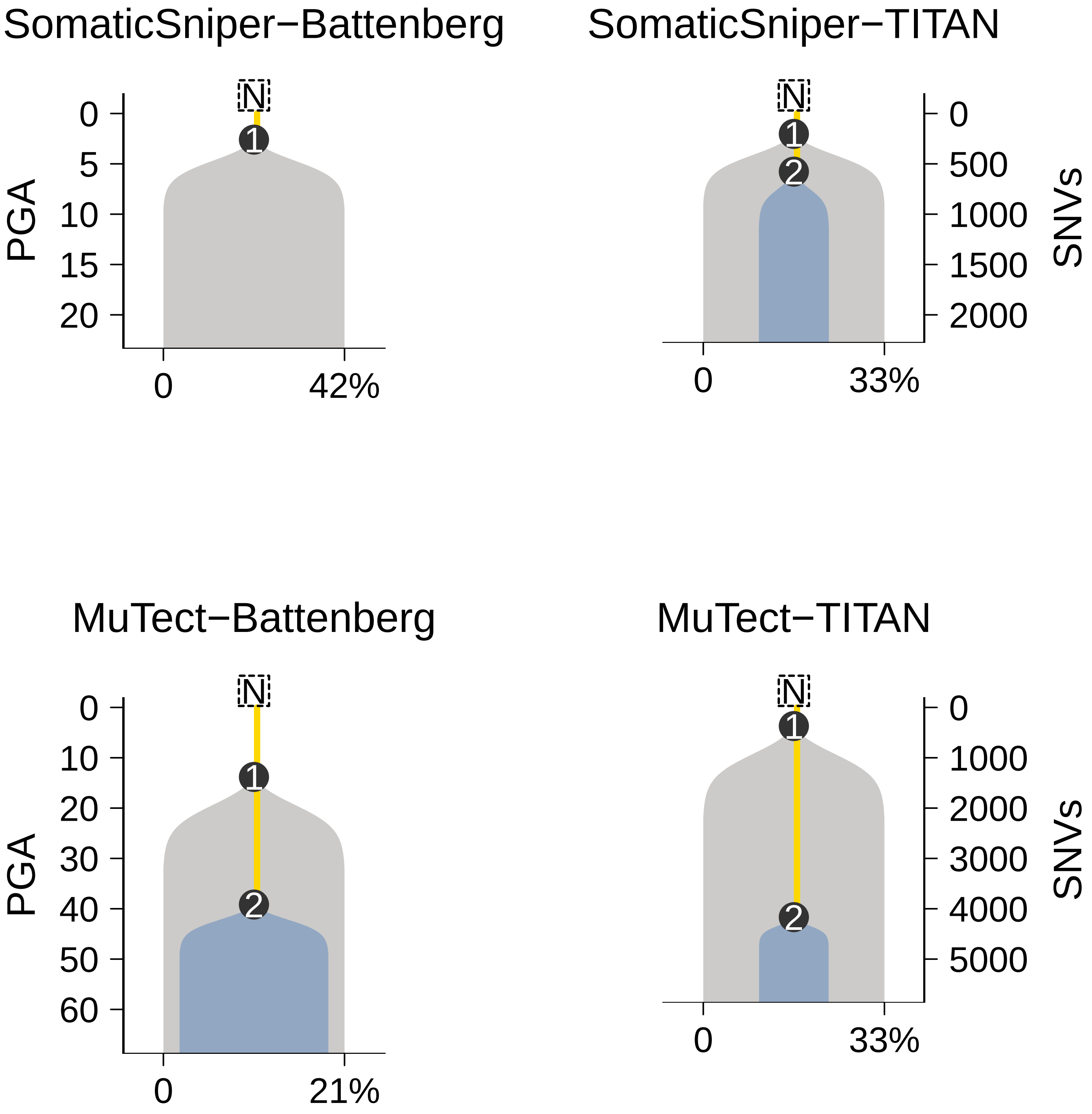

Weischenfeldt_010_T_wgs

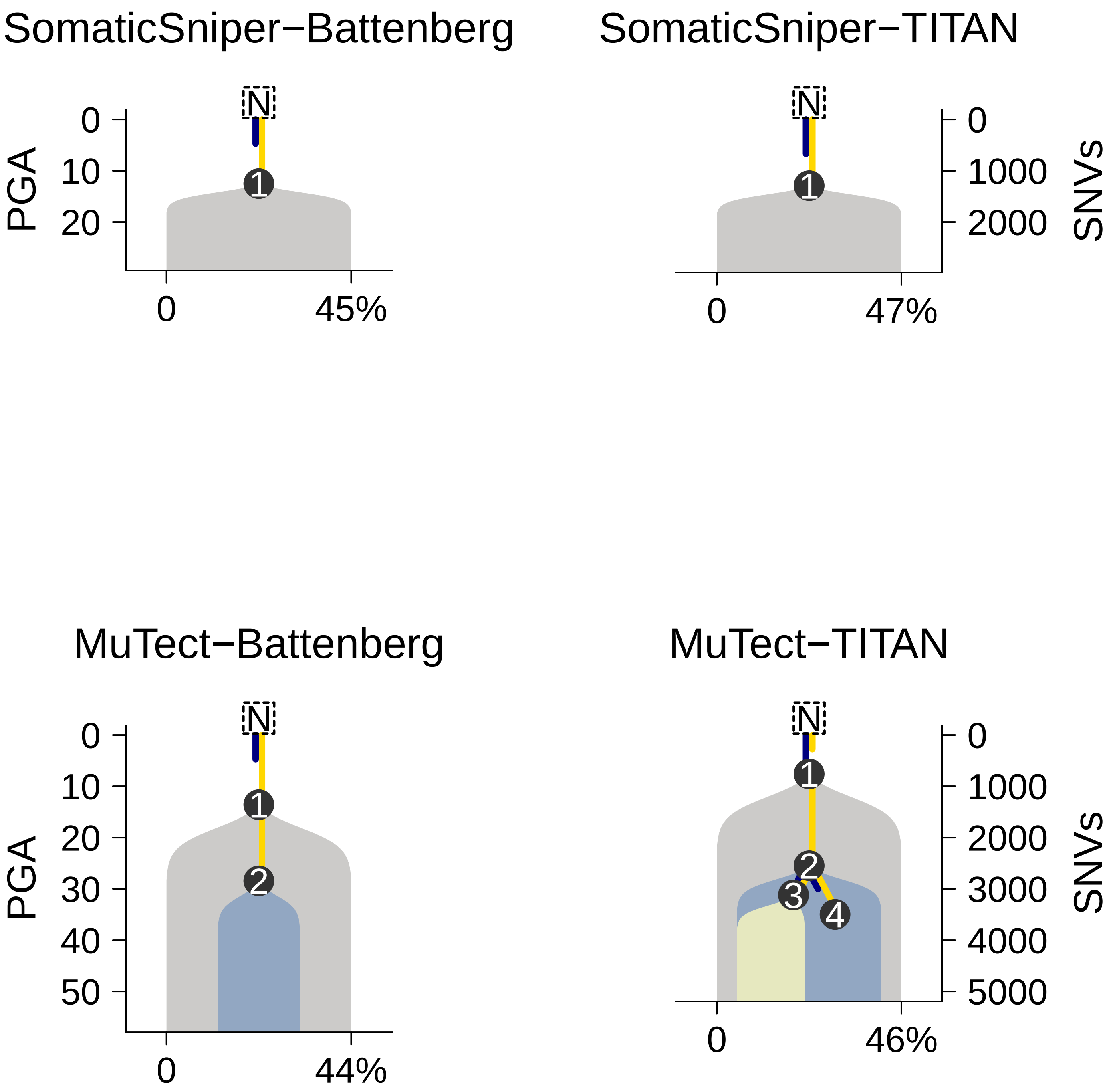

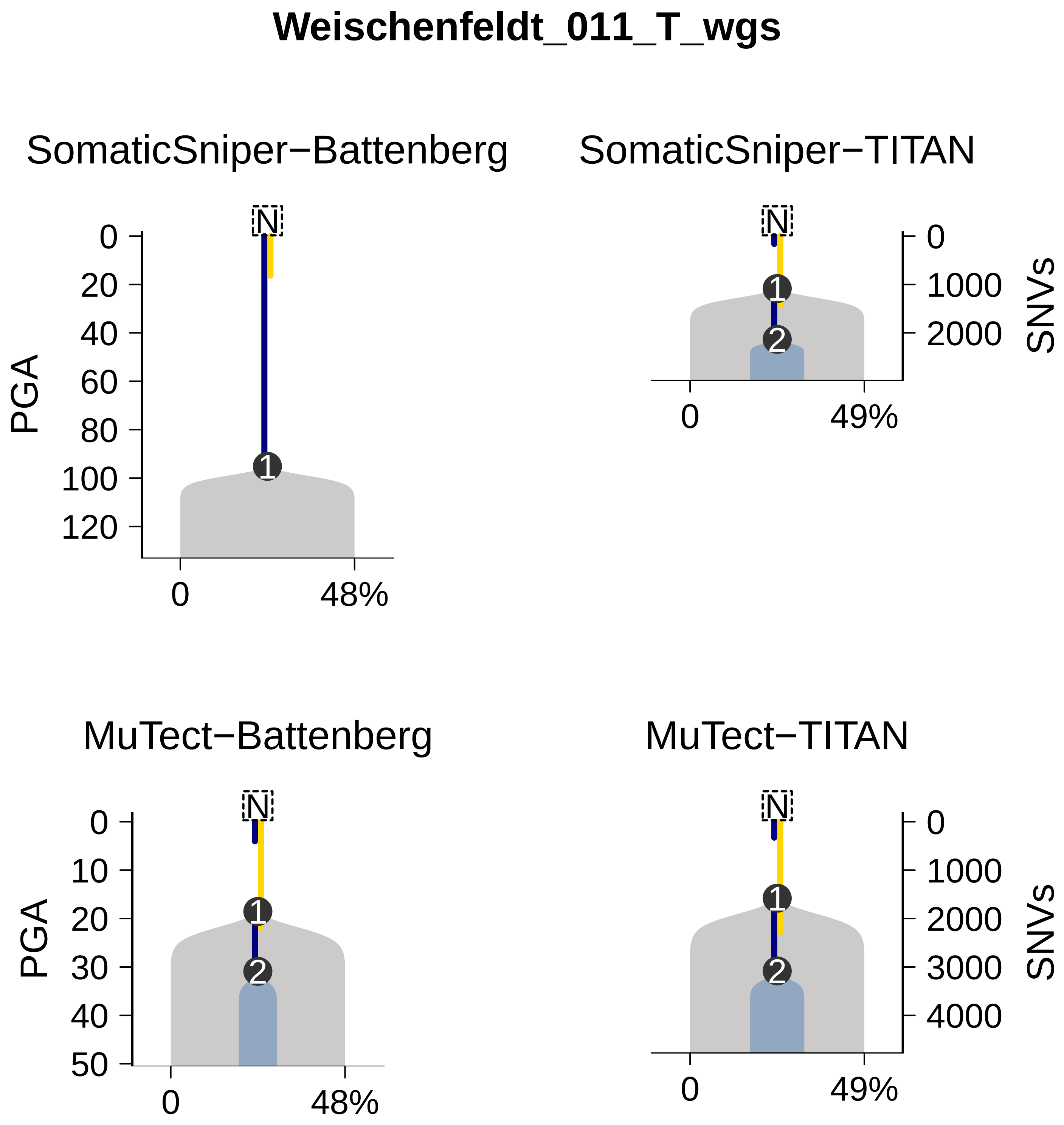
Weischenfeldt_011_T_wgs
SomaticSniper−Battenberg
SomaticSniper−TITAN
MuTect−Battenberg
MuTect−TITAN
N
1
2
PGA
SNVs
0
20
40
60
80
100
120
10
30
50
1000
2000
3000
4000
48%
49%

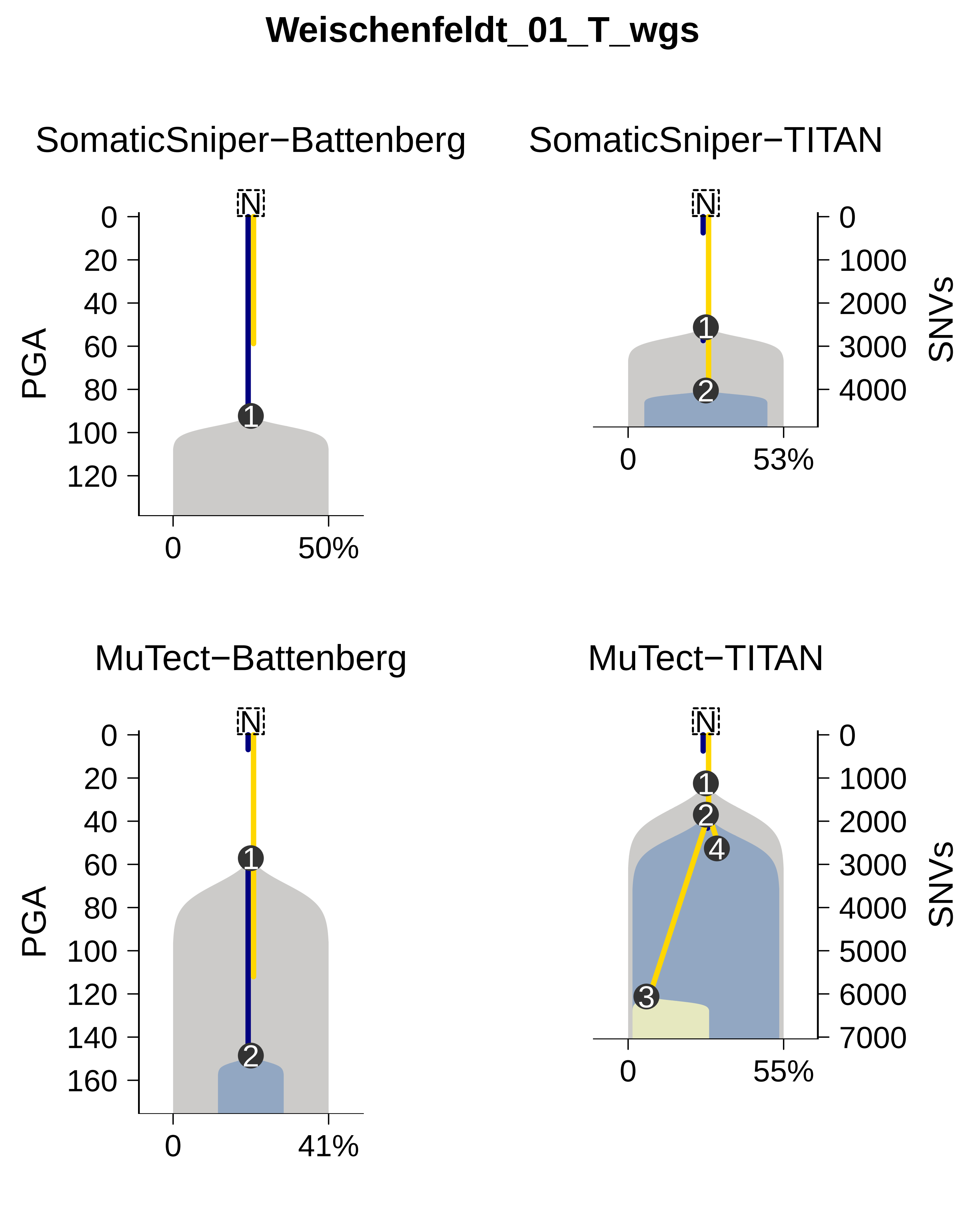
Weischenfeldt_01_T_wgs
SomaticSniper−Battenberg
SomaticSniper−TITAN
MuTect−Battenberg
MuTect−TITAN
PGA
SNVs
N
0
20
40
60
80
100
120
140
160
0
1000
2000
3000
4000
5000
6000
7000
0
50%
53%
41%
55%
1
2
3
4

Weischenfeldt_02_T_wgs

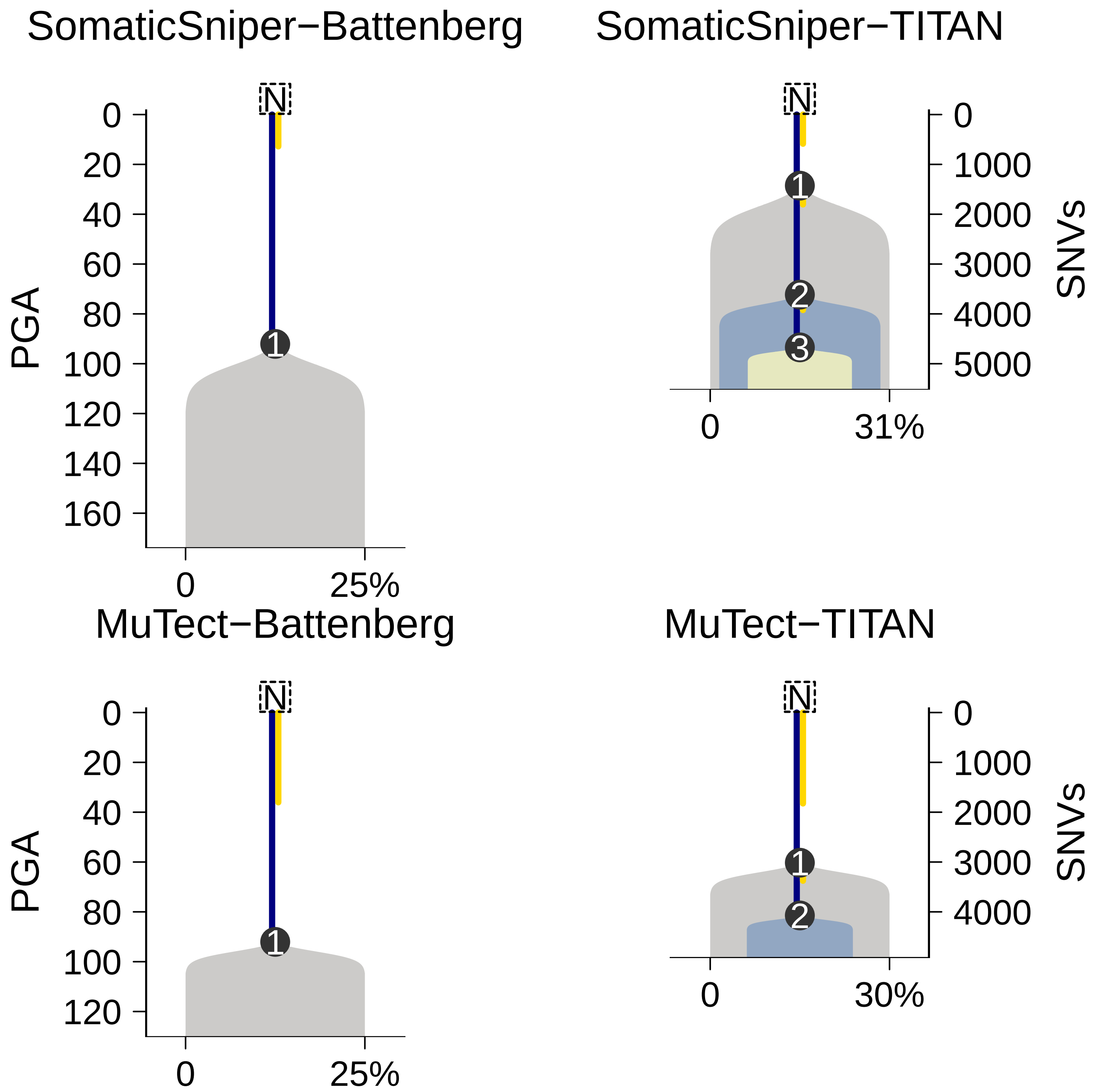

Weischenfeldt_03_T_wgs

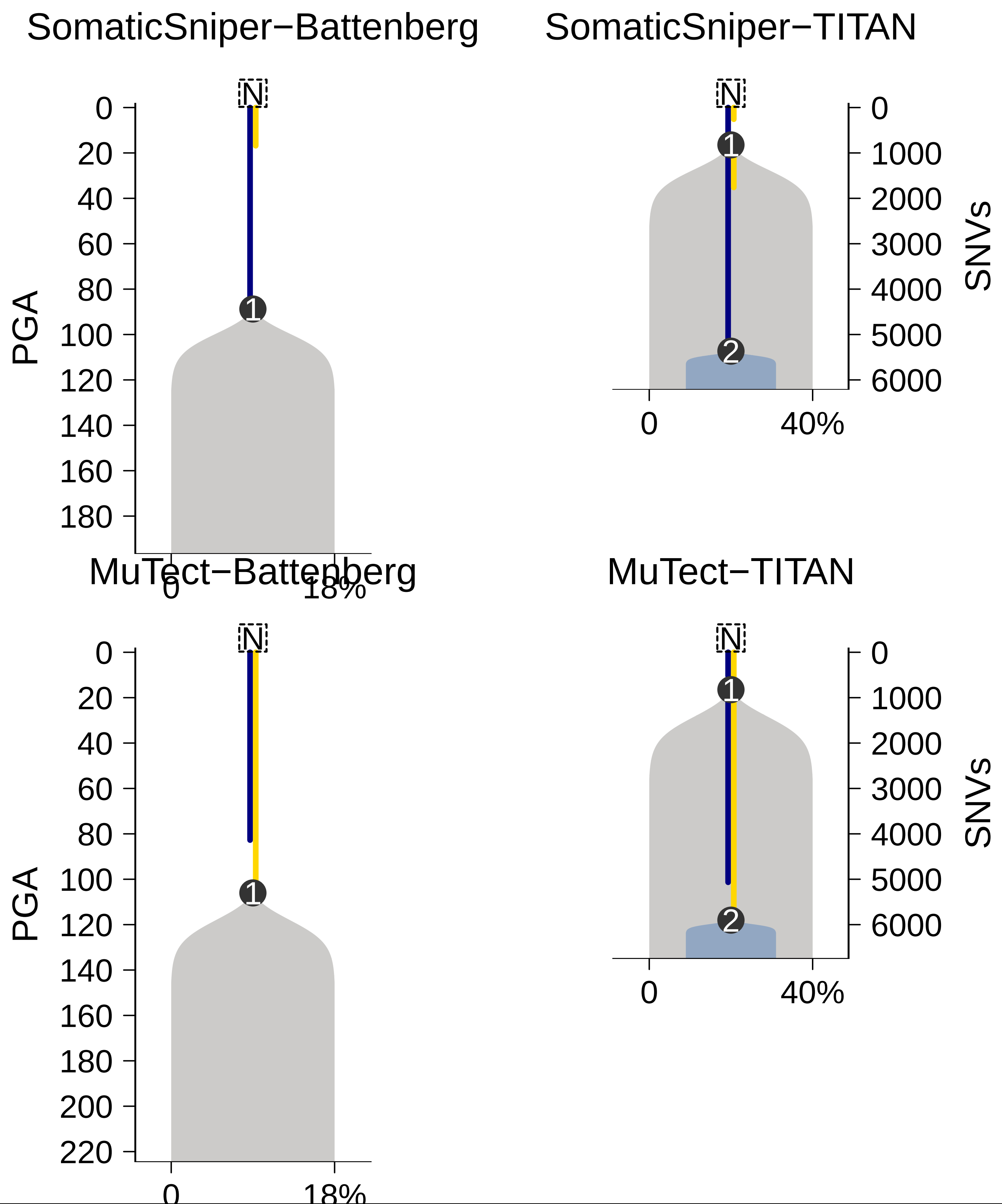

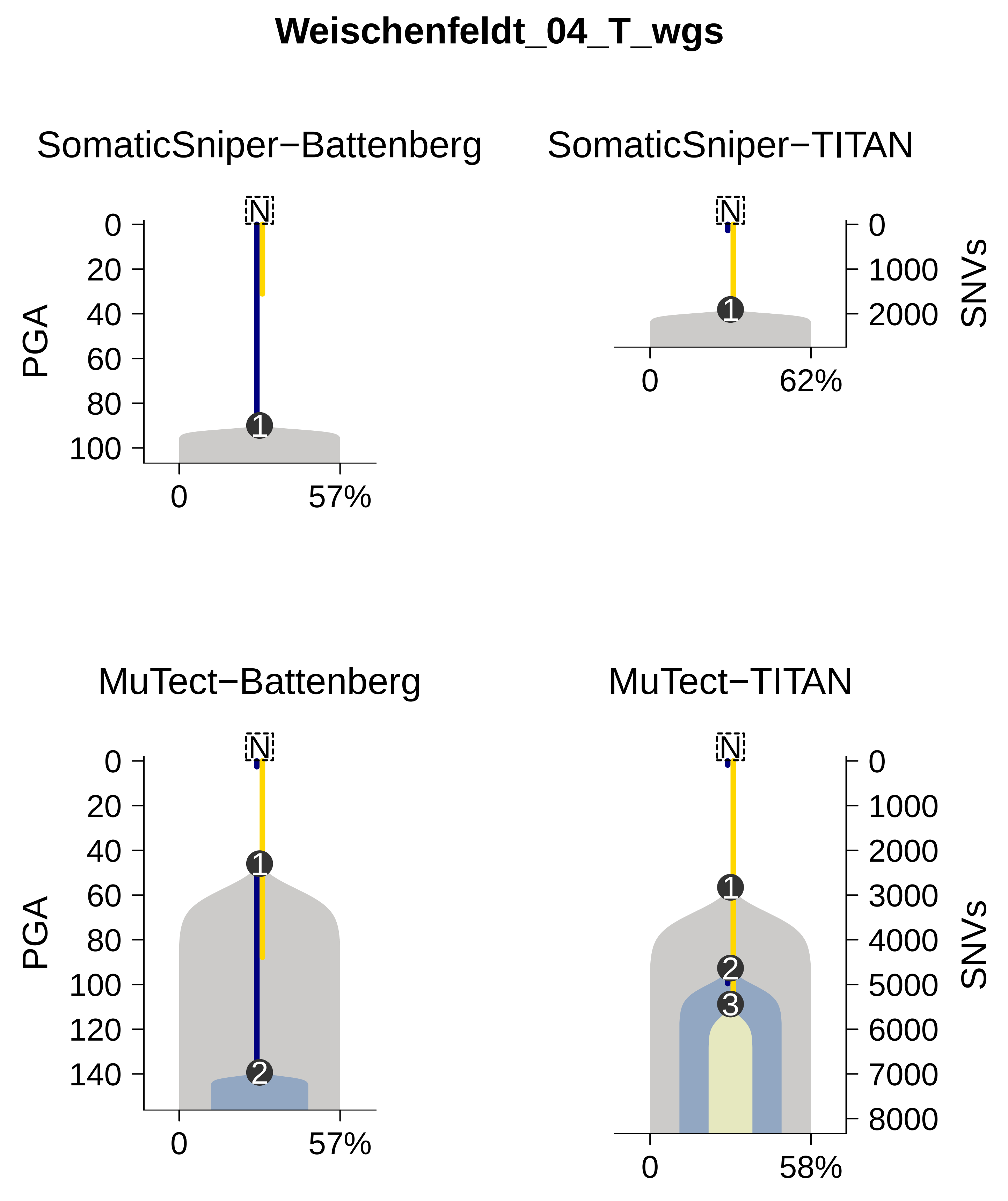

Weischenfeldt_04_T_wgs
SomaticSniper−Battenberg
SomaticSniper−TITAN
MuTect−Battenberg
MuTect−TITAN
PGA
SNVs
0
20
40
60
80
100
120
140
1000
2000
3000
4000
5000
6000
7000
8000
57%
62%
58%
N
1
2
3

Weischenfeldt_05_T_wgs

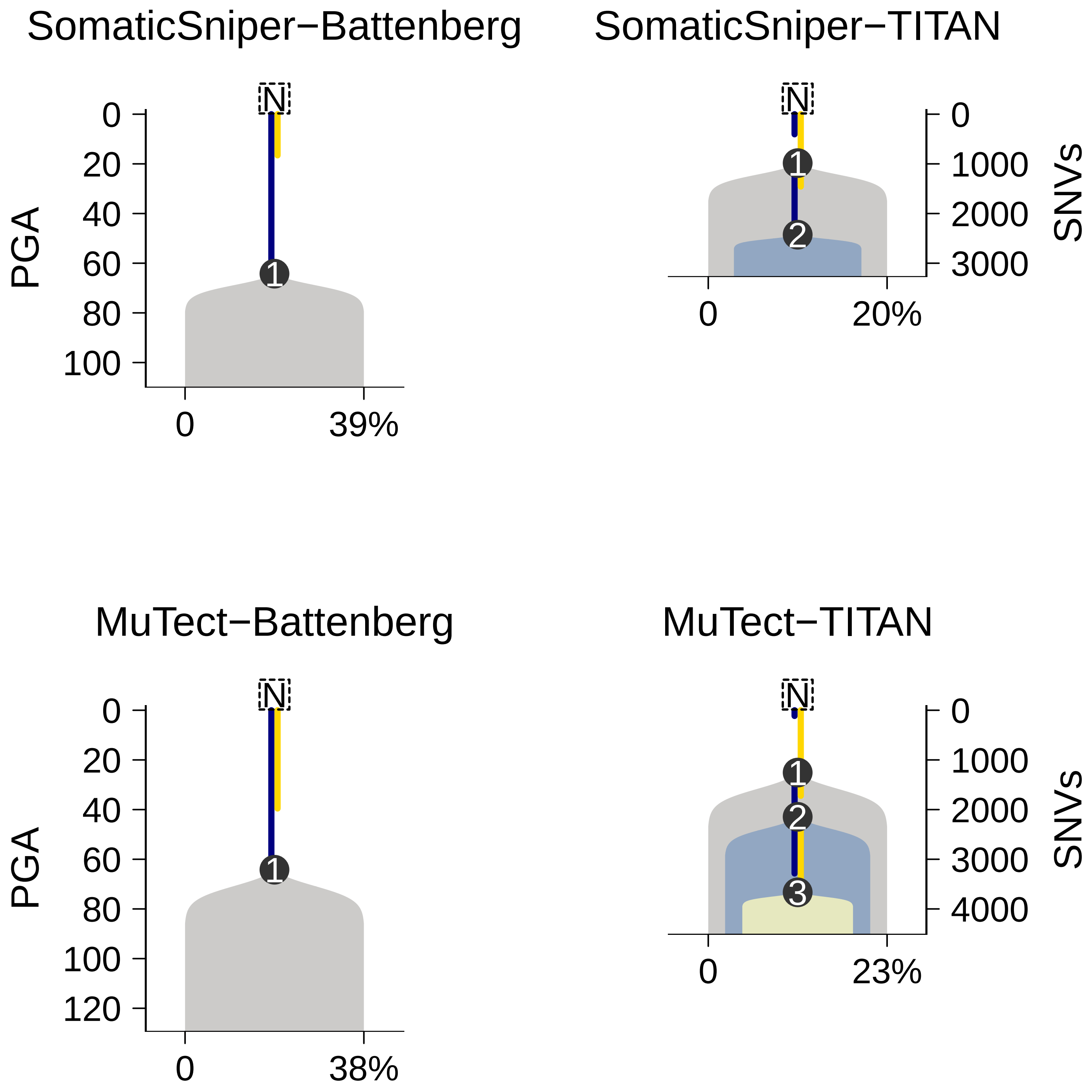

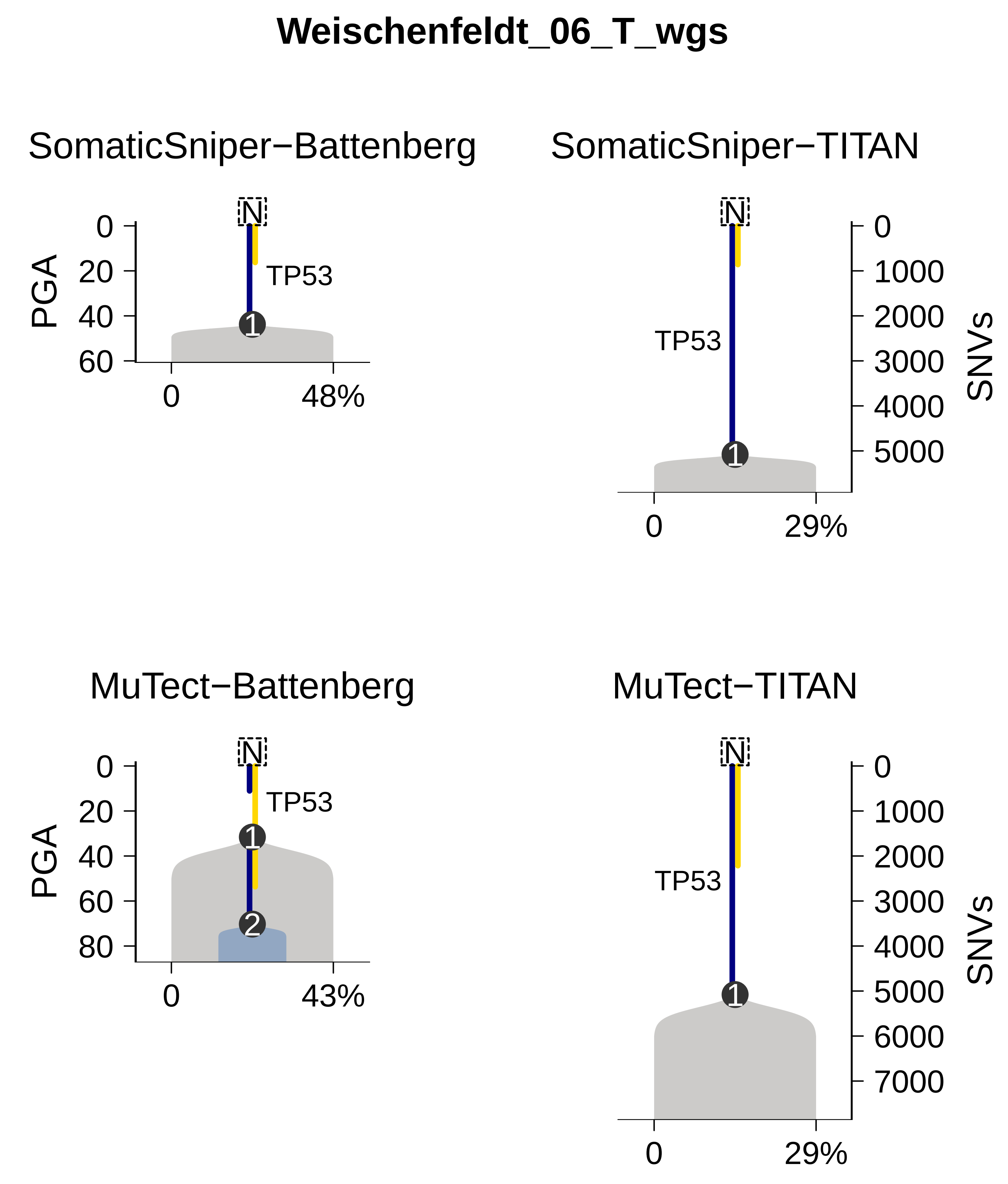
Weischenfeldt_06_T_wgs
SomaticSniper−Battenberg
SomaticSniper−TITAN
MuTect−Battenberg
MuTect−TITAN
N
TP53
PGA
SNVs
0
20
40
60
80
0
1000
2000
3000
4000
5000
6000
7000
1
2
0
48%
29%
43%

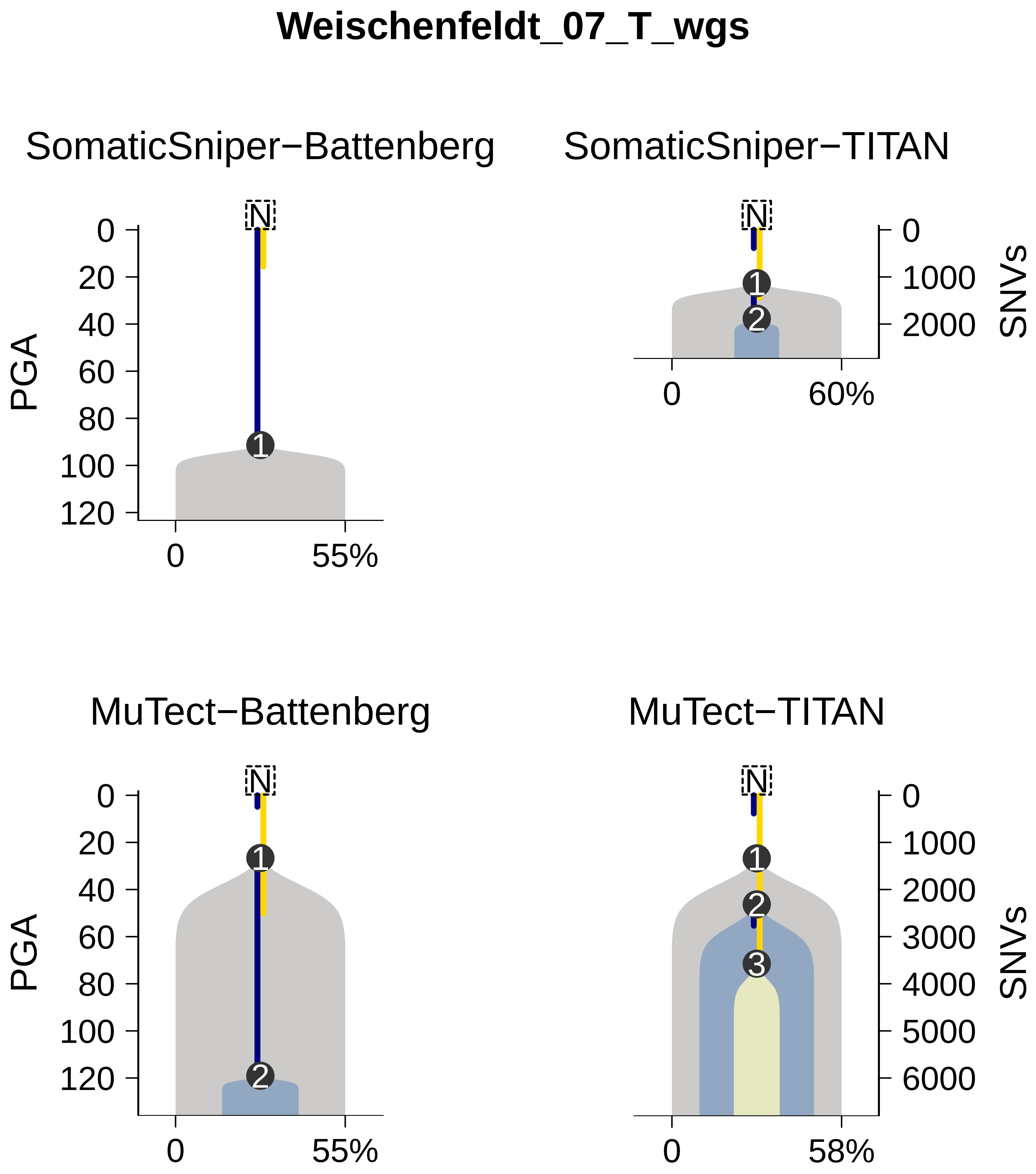
Weischenfeldt_07_T_wgs
SomaticSniper−Battenberg
SomaticSniper−TITAN
MuTect−Battenberg
MuTect−TITAN
PGA
SNVs
N
0
20
40
60
80
100
120
0
1000
2000
3000
4000
5000
6000
55%
60%
58%

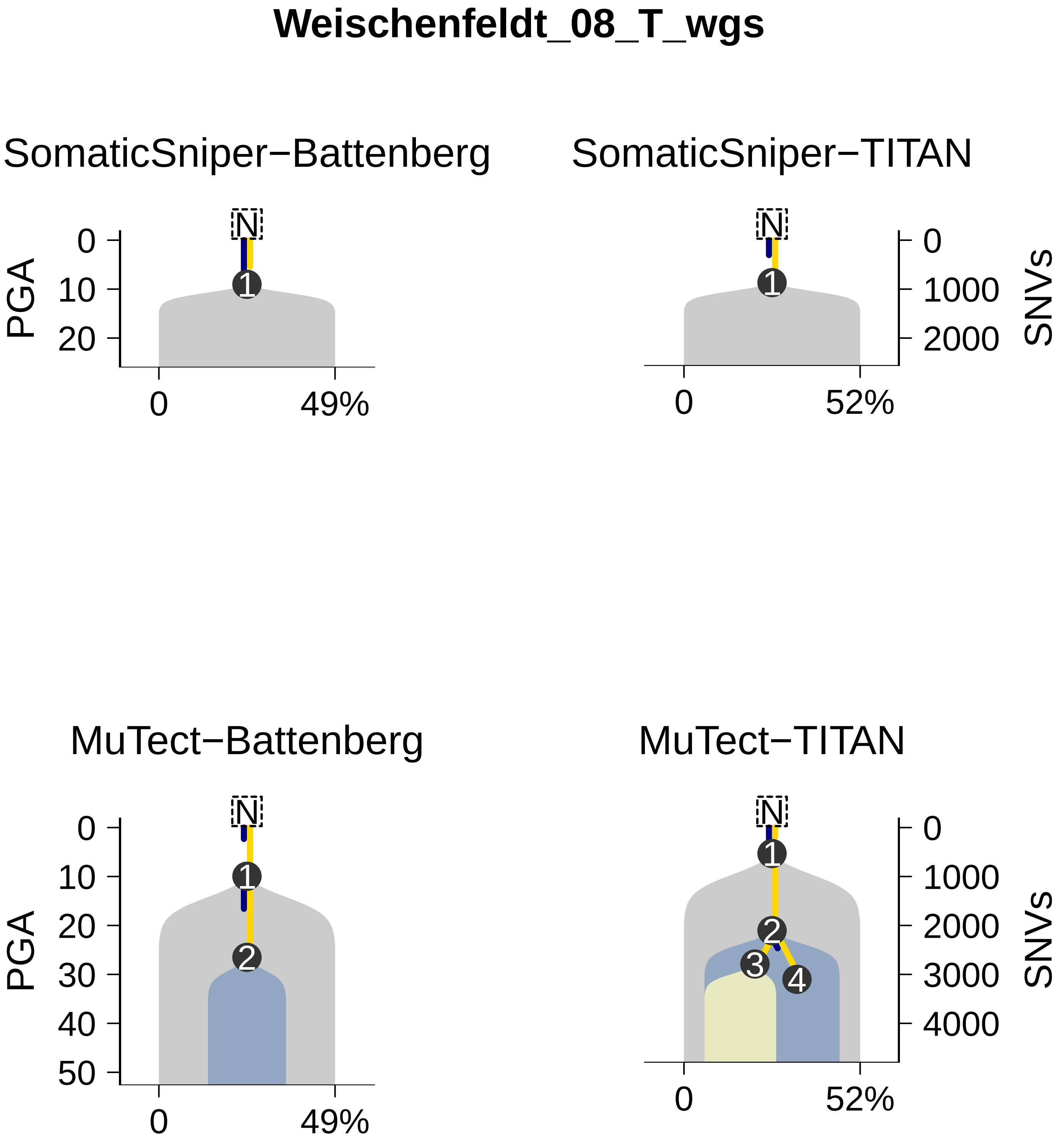
Weischenfeldt_08_T_wgs
SomaticSniper−Battenberg
SomaticSniper−TITAN
MuTect−Battenberg
MuTect−TITAN
N
1
2
3
4
PGA
SNVs
0
10
20
30
40
50
1000
2000
3000
4000
49%
52%

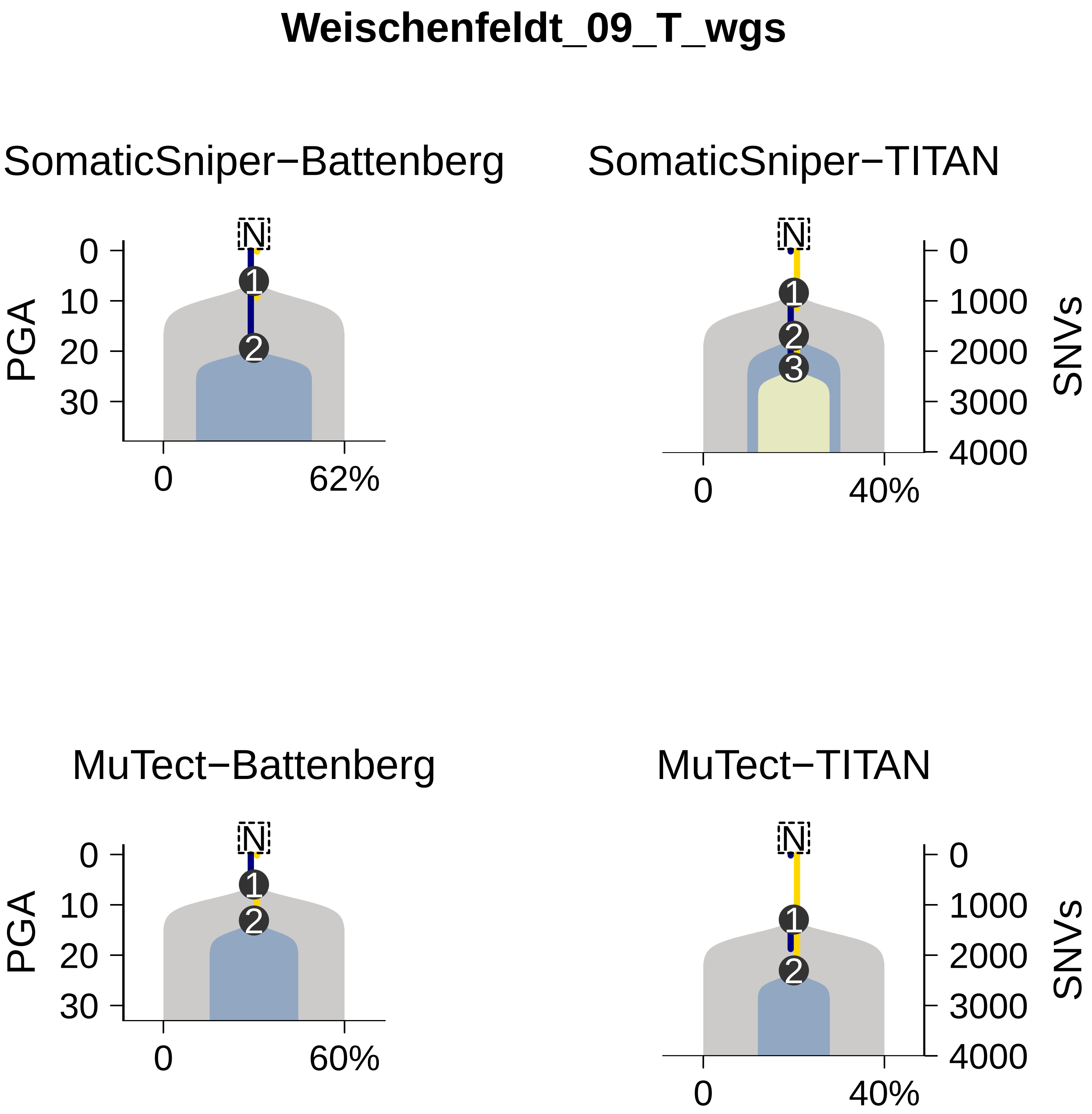
Weischenfeldt_09_T_wgs
SomaticSniper−Battenberg
SomaticSniper−TITAN
MuTect−Battenberg
MuTect−TITAN
N
1
2
3
PGA
SNVs
0
10
20
30
1000
2000
3000
4000
62%
60%
40%

CPCG0100−B1F3

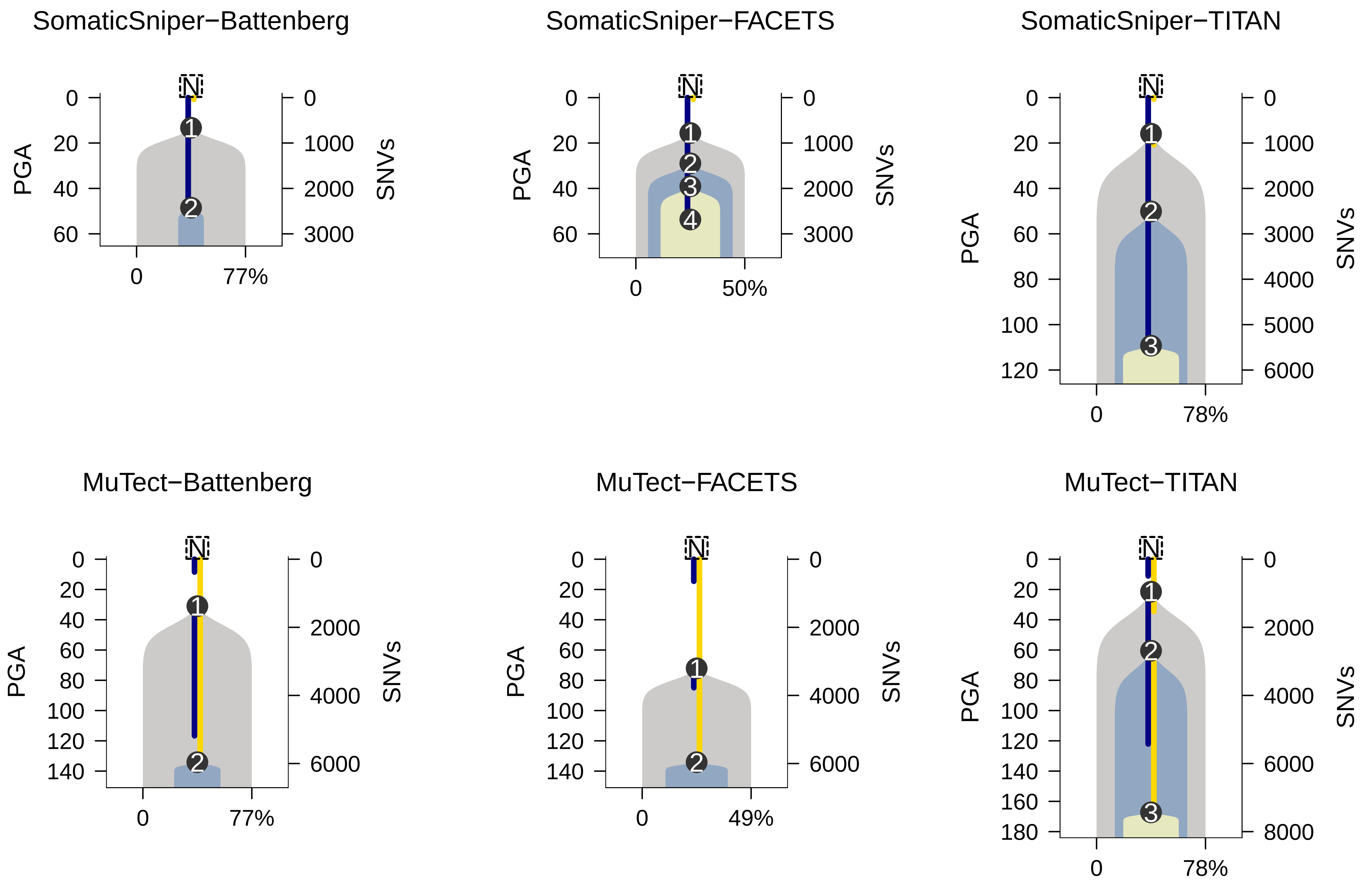

CPCG0100−B1F5

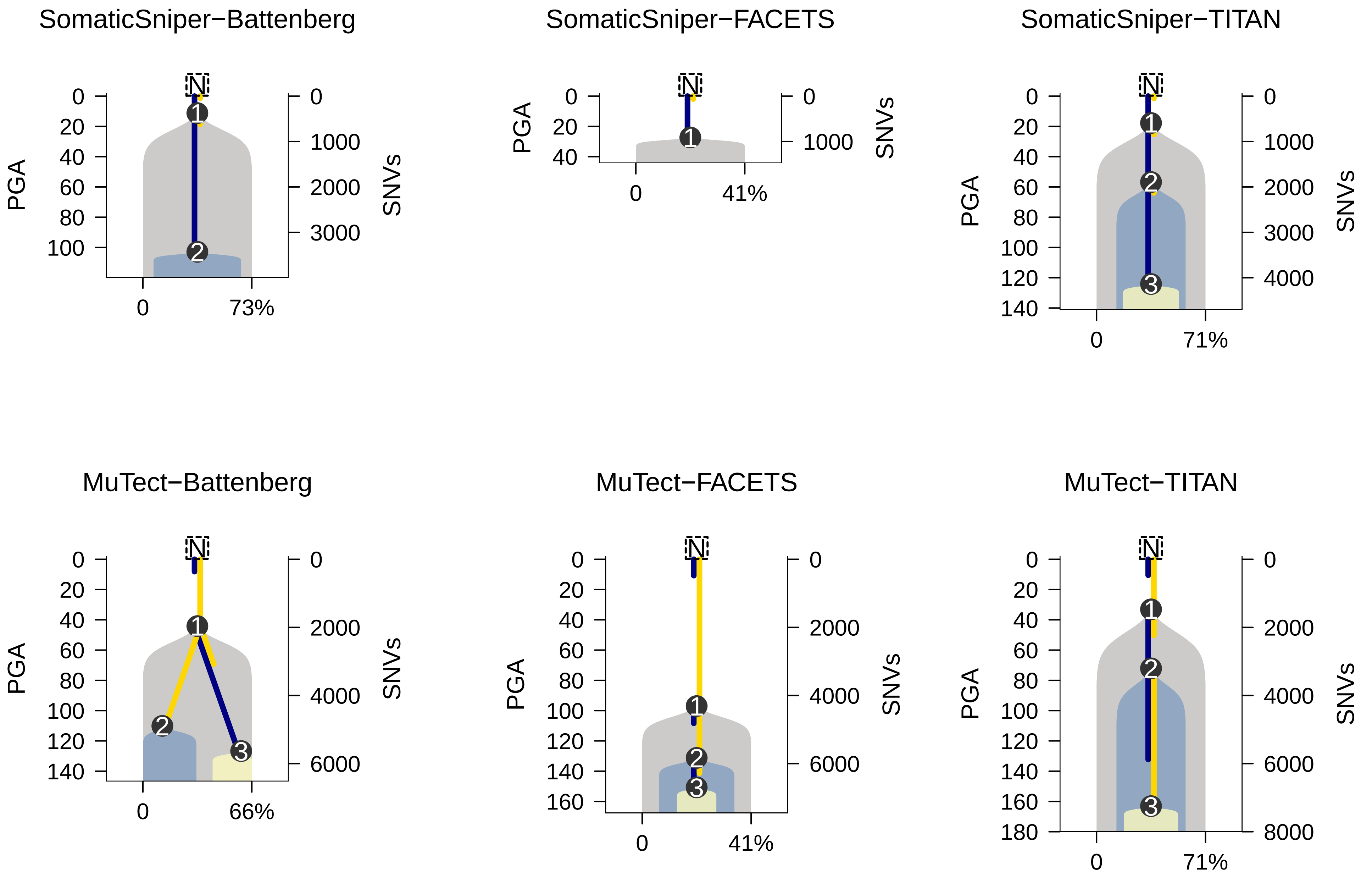

CPCG0100−B1F6

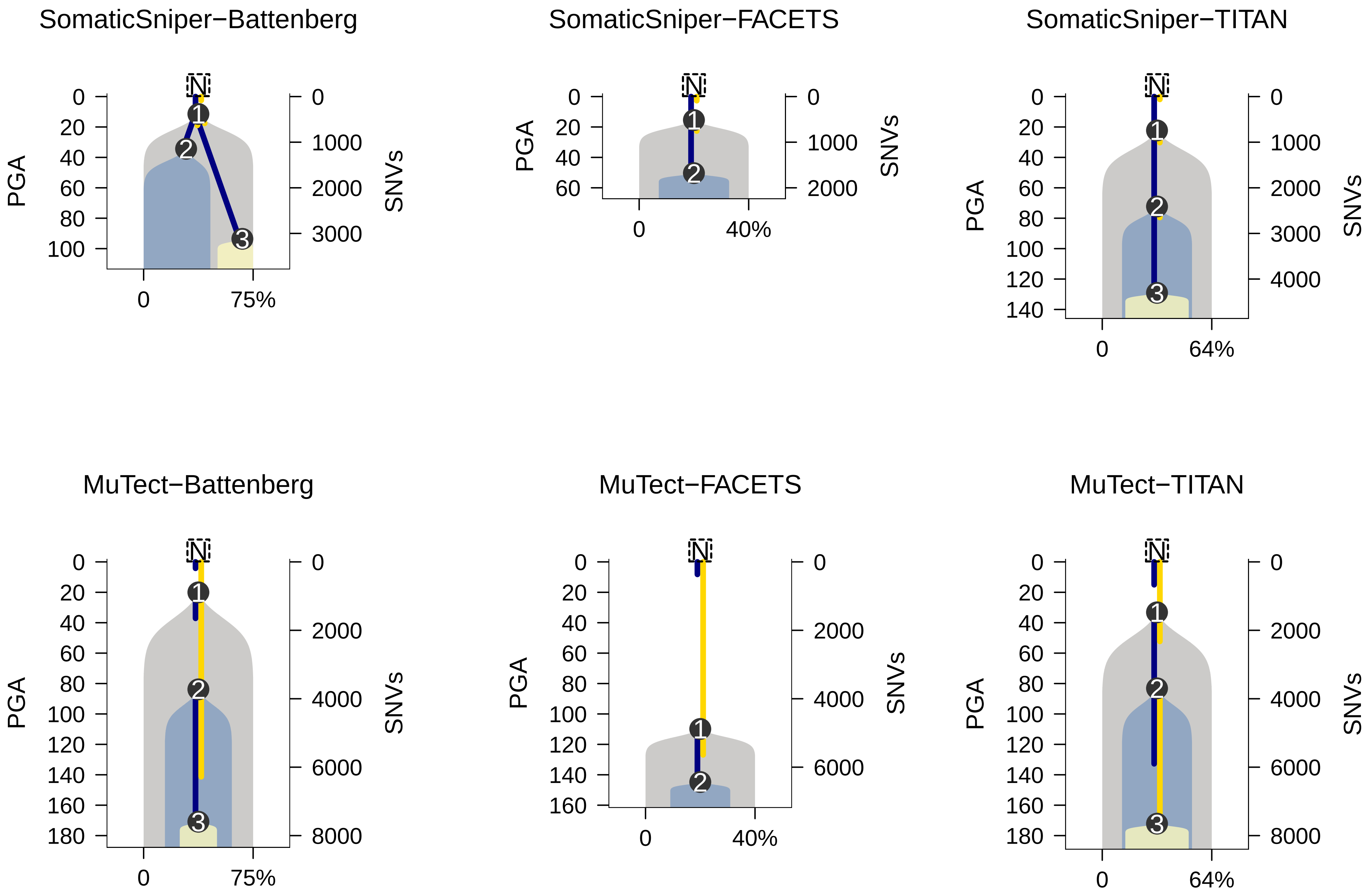

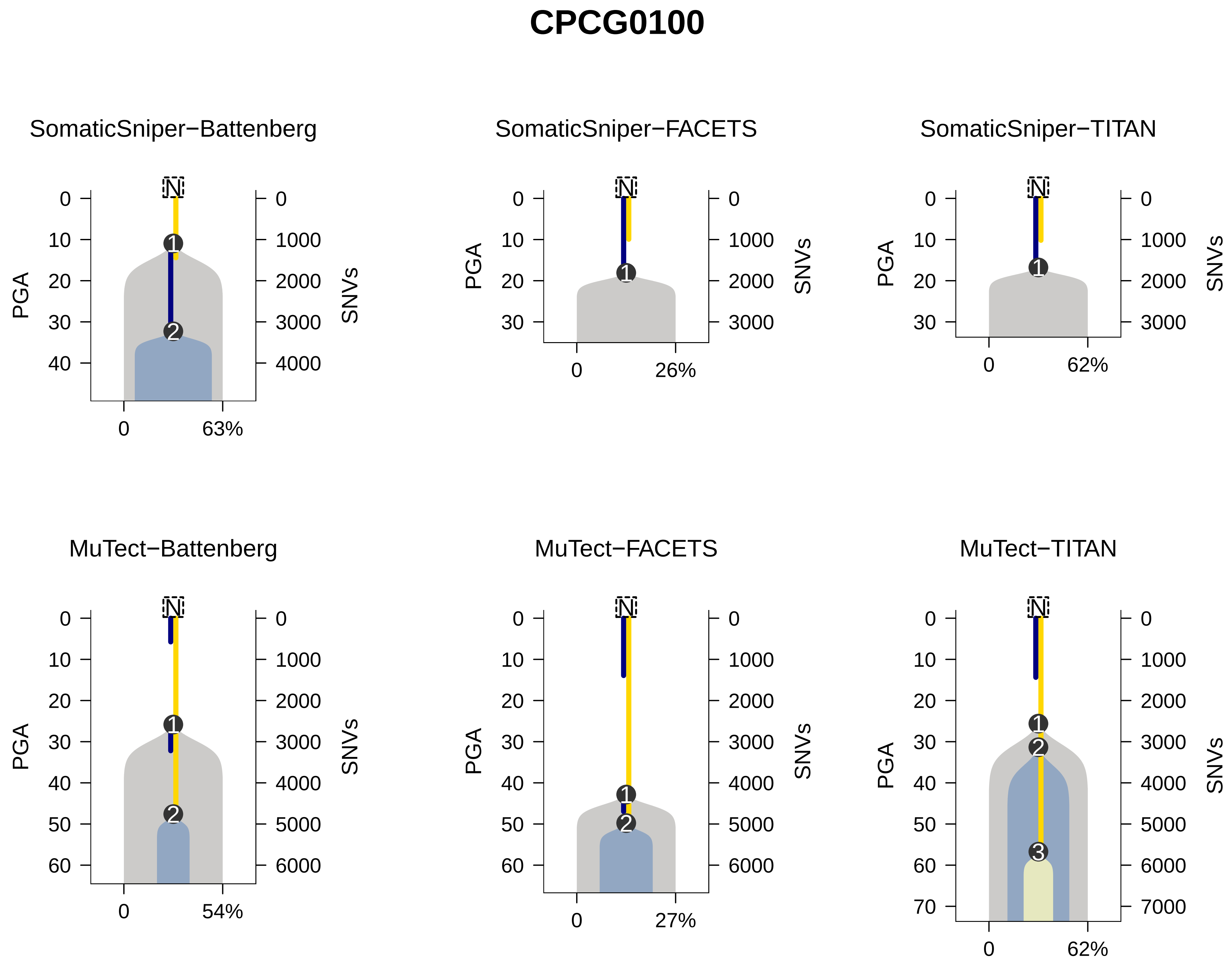

CPCG0100
SomaticSniper−Battenberg
SomaticSniper−FACETS
SomaticSniper−TITAN
MuTect−Battenberg
MuTect−FACETS
MuTect−TITAN
PGA
SNVs
N
0
63%
26%
62%
54%
27%
62%

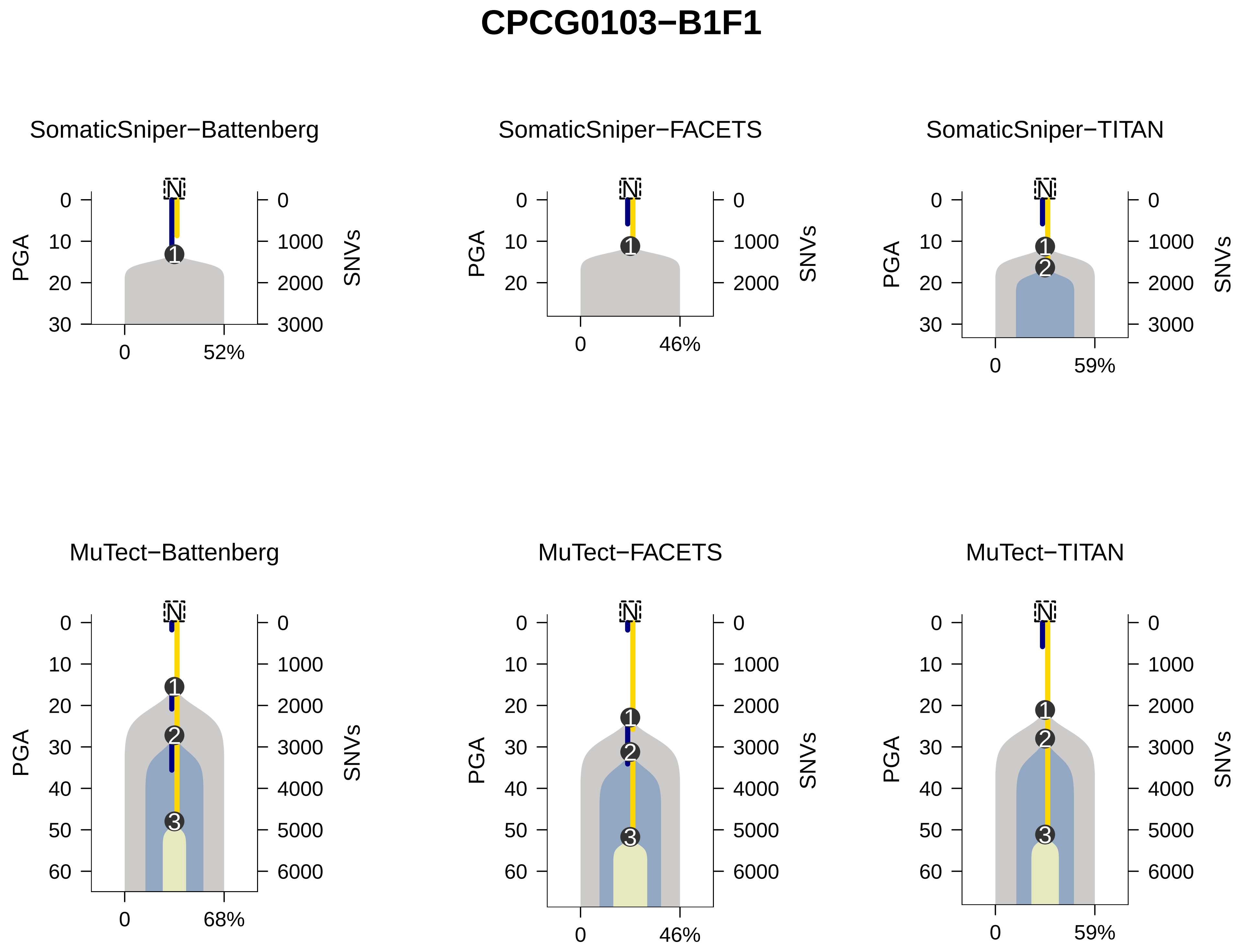
CPCG0103−B1F1
SomaticSniper−Battenberg
SomaticSniper−FACETS
SomaticSniper−TITAN
MuTect−Battenberg
MuTect−FACETS
MuTect−TITAN
PGA
SNVs
N
1
2
3
0
10
20
30
40
50
60
1000
2000
3000
4000
5000
6000
52%
46%
59%
68%

CPCG0103−B1F2

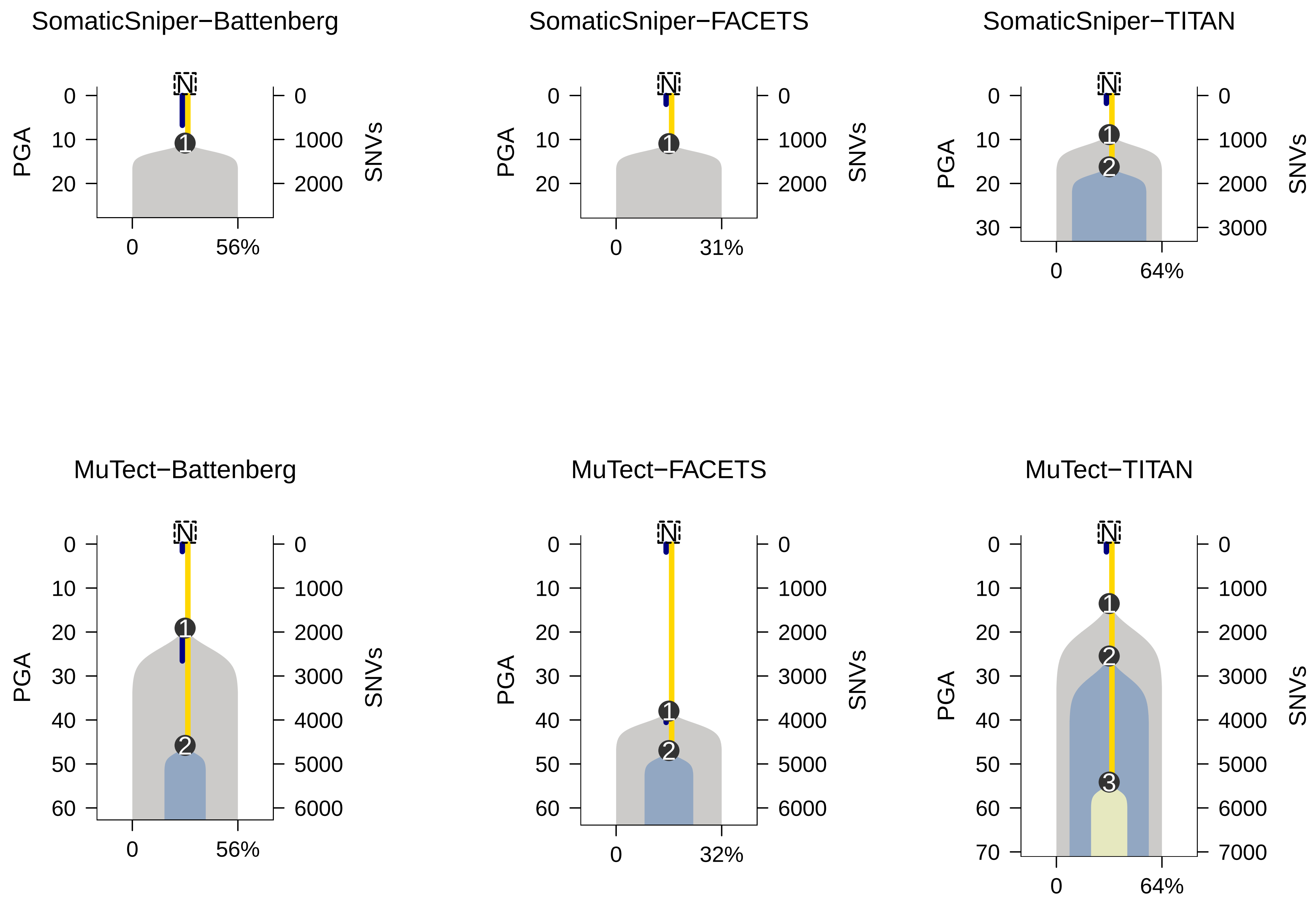

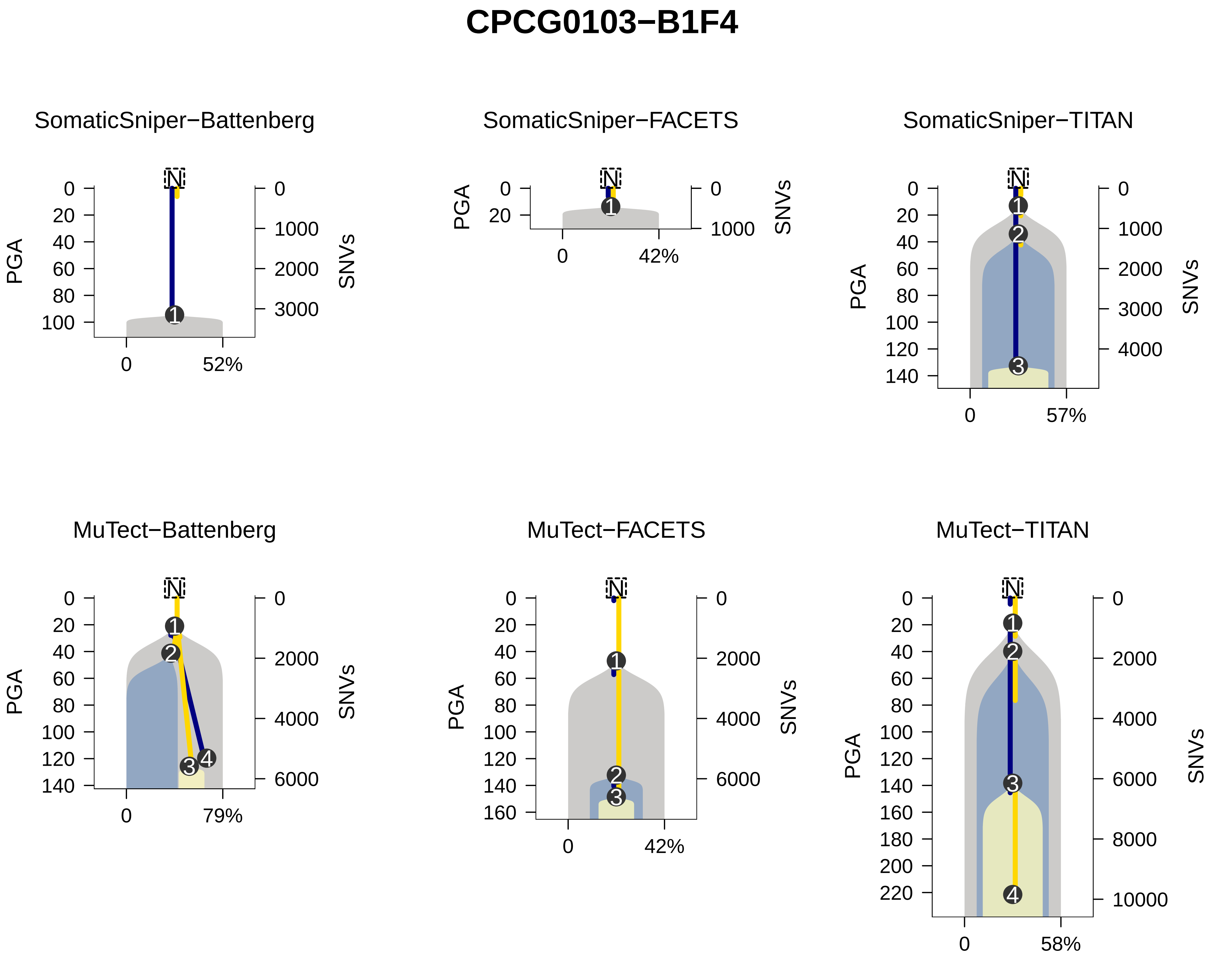

CPCG0103−B1F4
SomaticSniper−Battenberg
SomaticSniper−FACETS
SomaticSniper−TITAN
MuTect−Battenberg
MuTect−FACETS
MuTect−TITAN
PGA
SNVs
52%
42%
57%
79%
58%

CPCG0260−B1F2

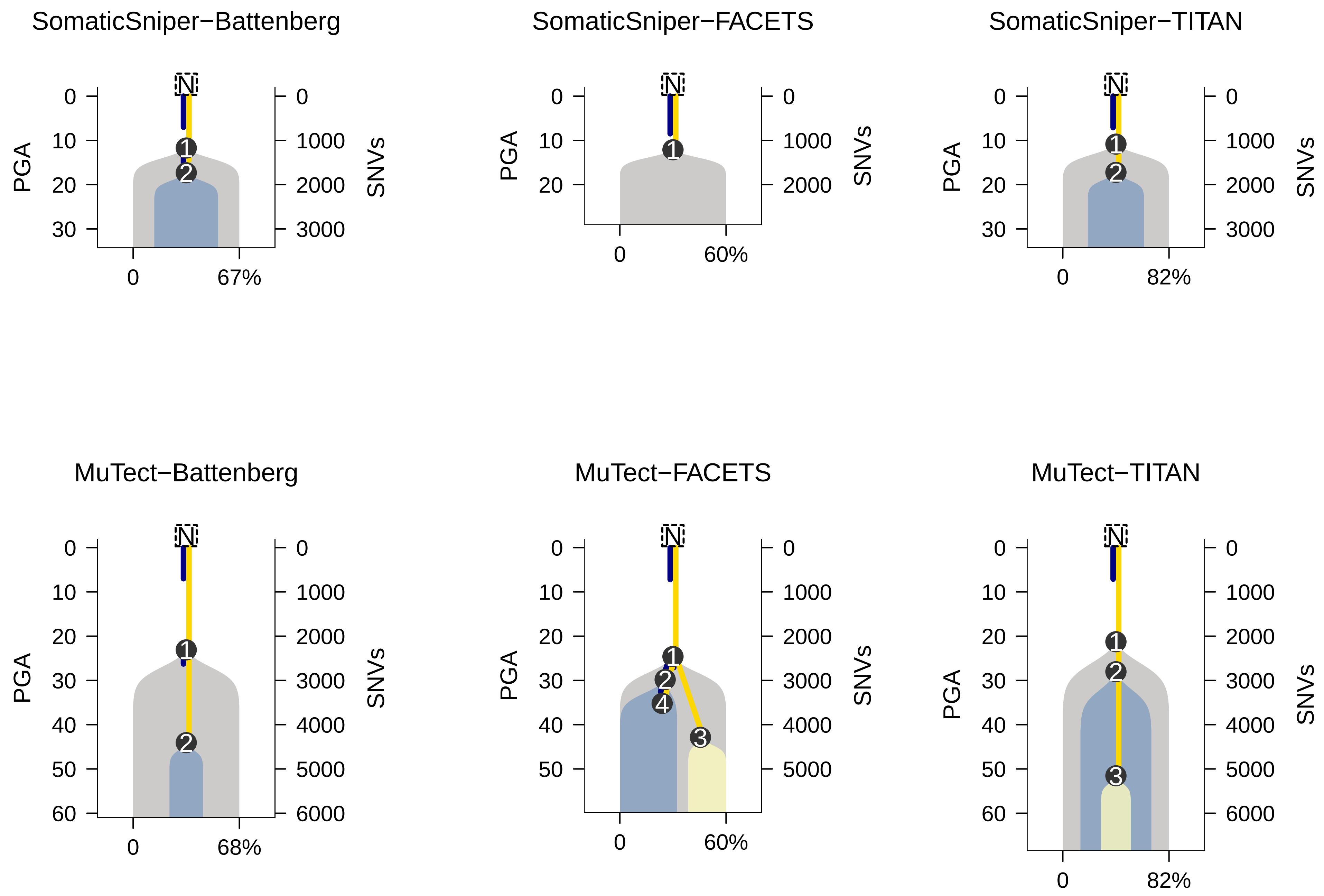

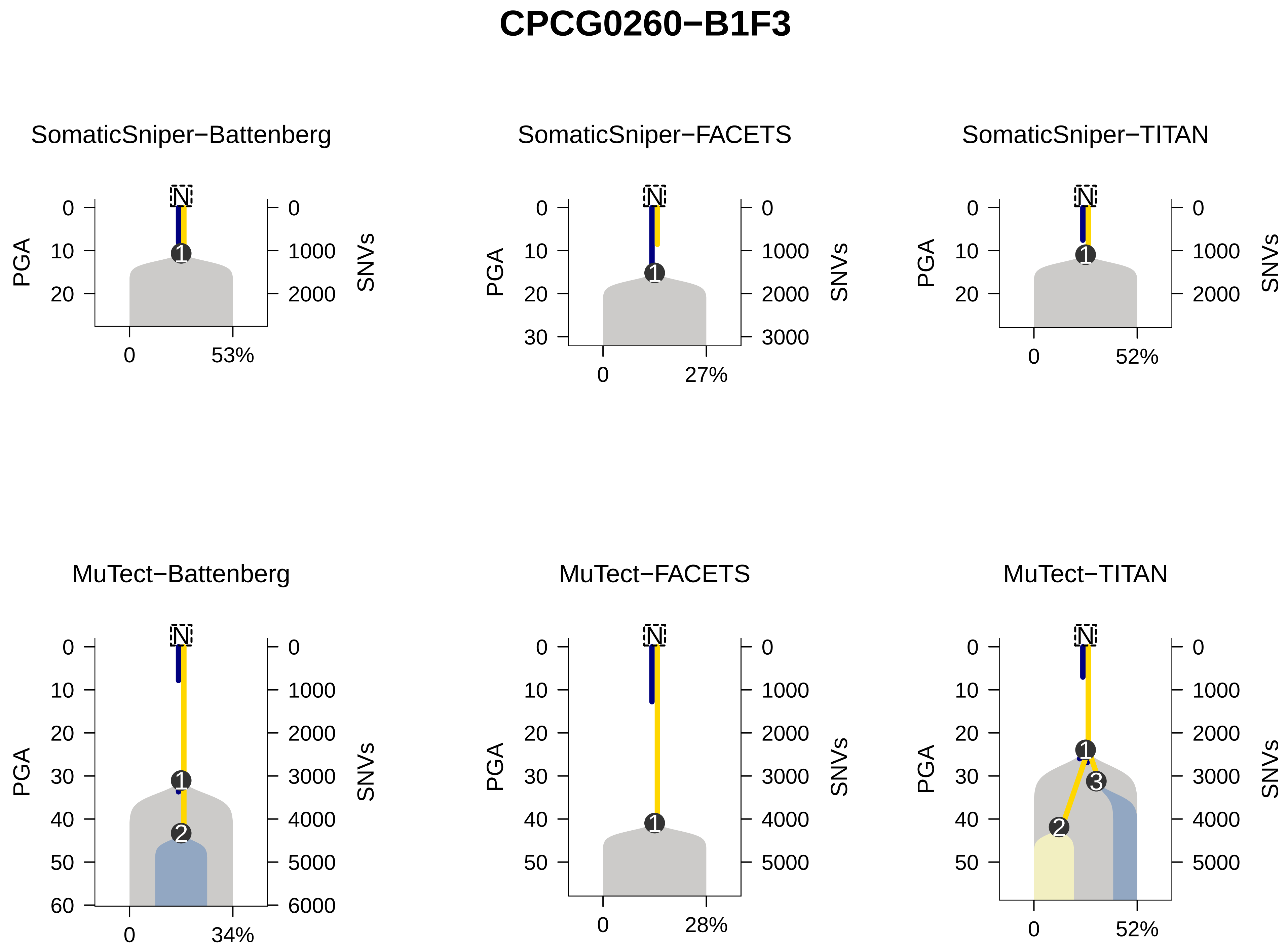
CPCG0260−B1F3
SomaticSniper−Battenberg
SomaticSniper−FACETS
SomaticSniper−TITAN
MuTect−Battenberg
MuTect−FACETS
MuTect−TITAN
PGA
SNVs
N
0
53%
27%
52%
34%
28%
52%

CPCG0260−B1F4

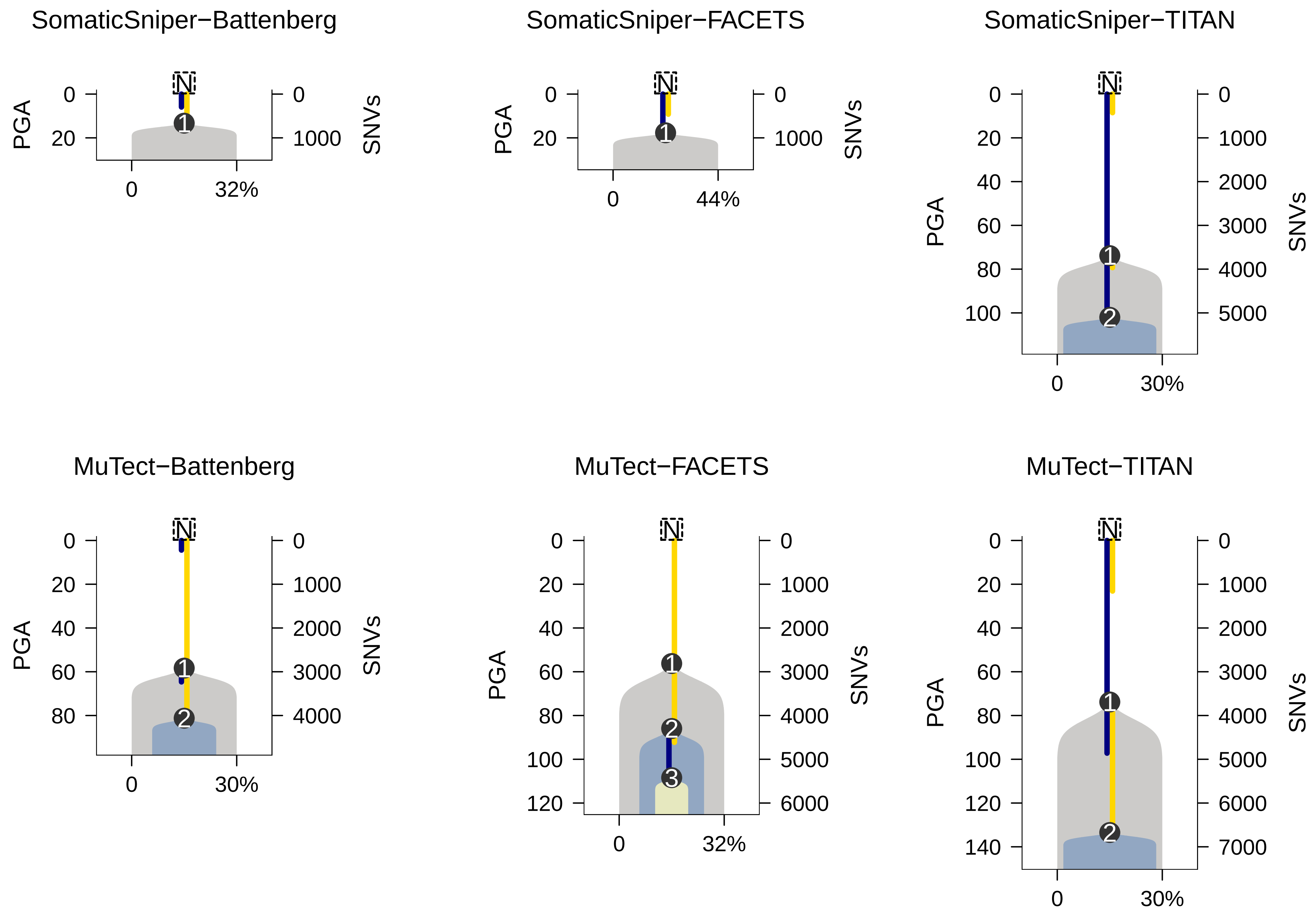

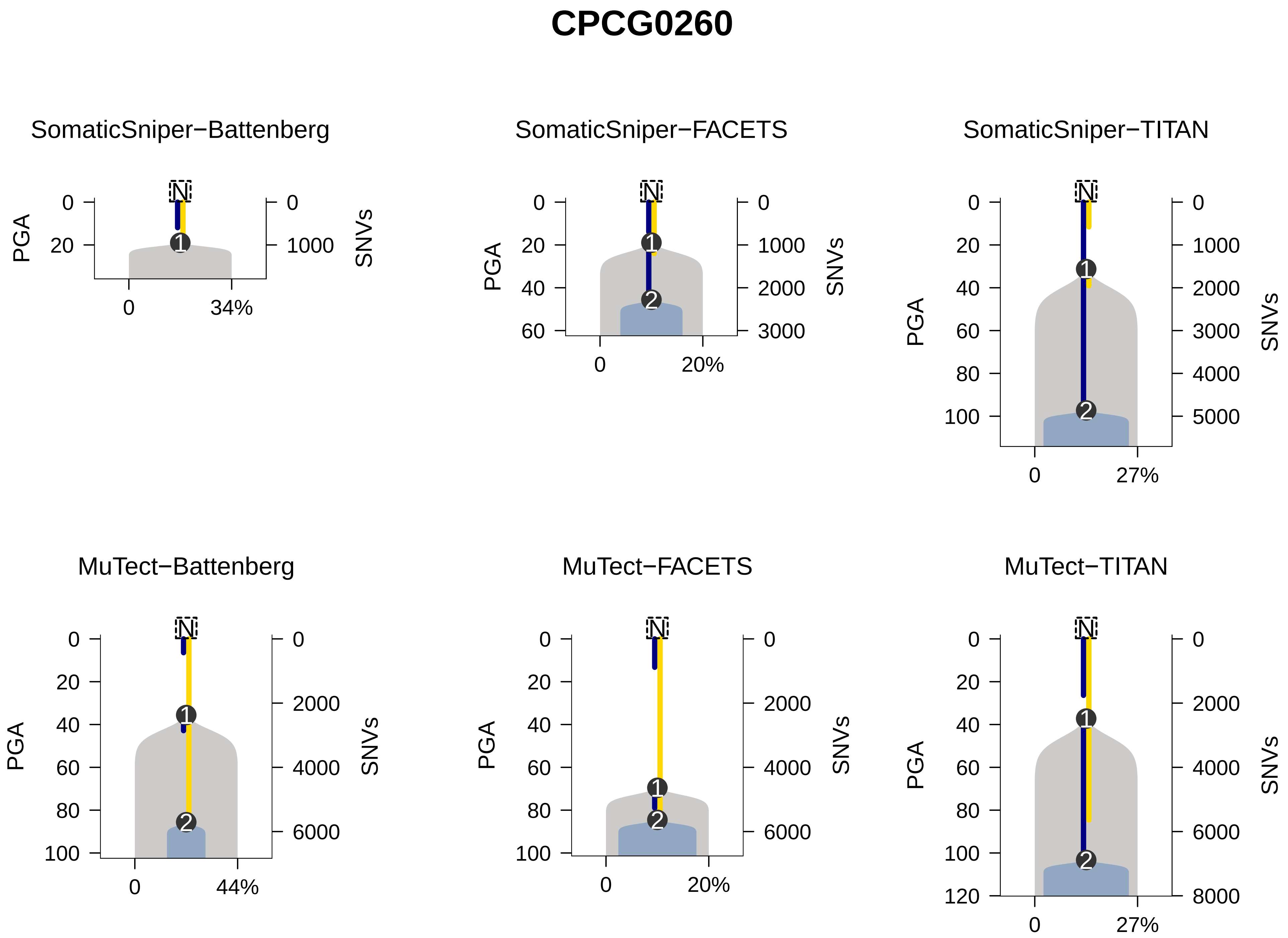
CPCG0260
SomaticSniper−Battenberg
SomaticSniper−FACETS
SomaticSniper−TITAN
MuTect−Battenberg
MuTect−FACETS
MuTect−TITAN
PGA
SNVs
N
1
2
0
20
40
60
80
100
120
1000
2000
3000
4000
5000
6000
8000
34%
20%
27%
44%

CPCG0334−B1F2

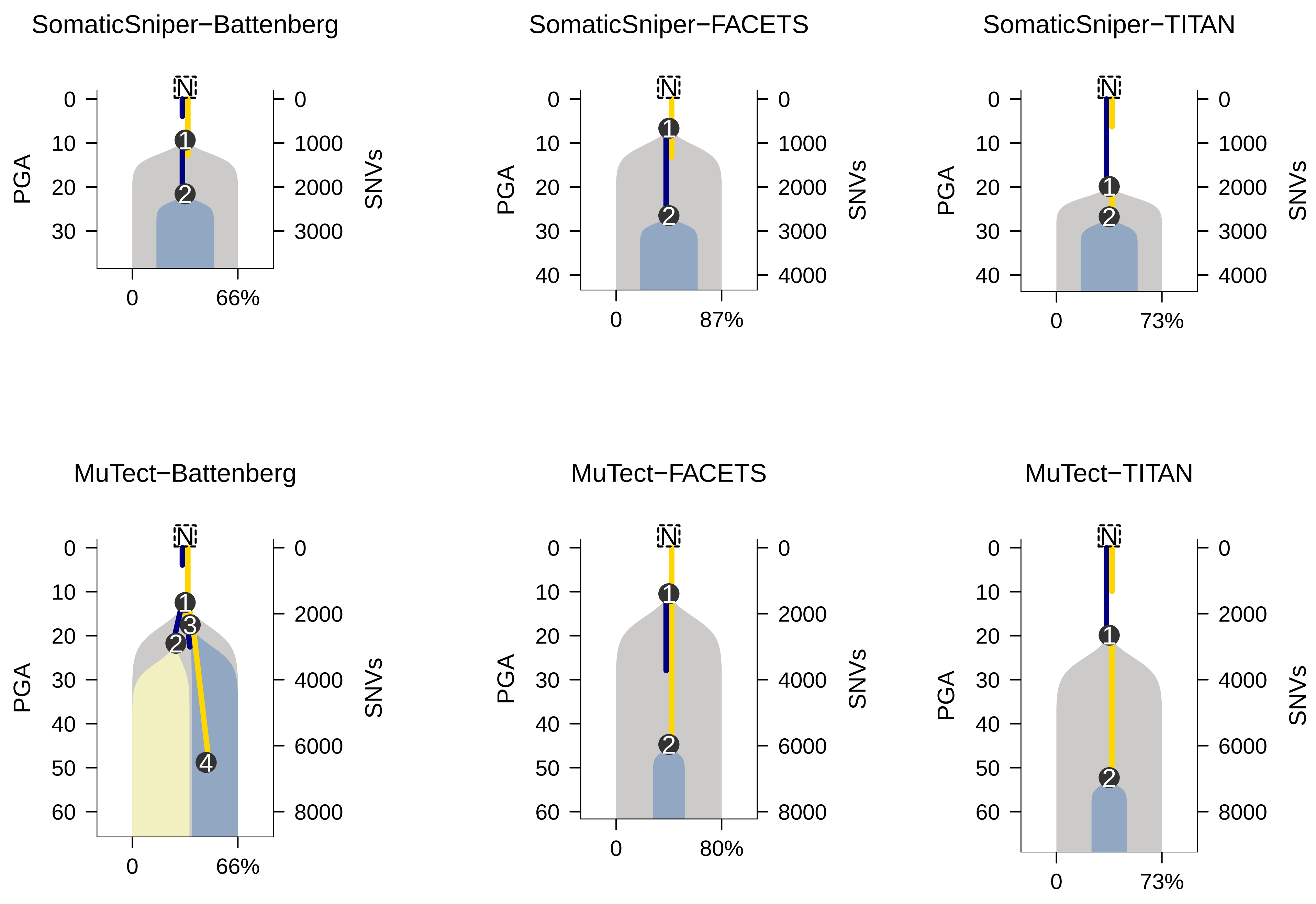

CPCG0334−B1F3

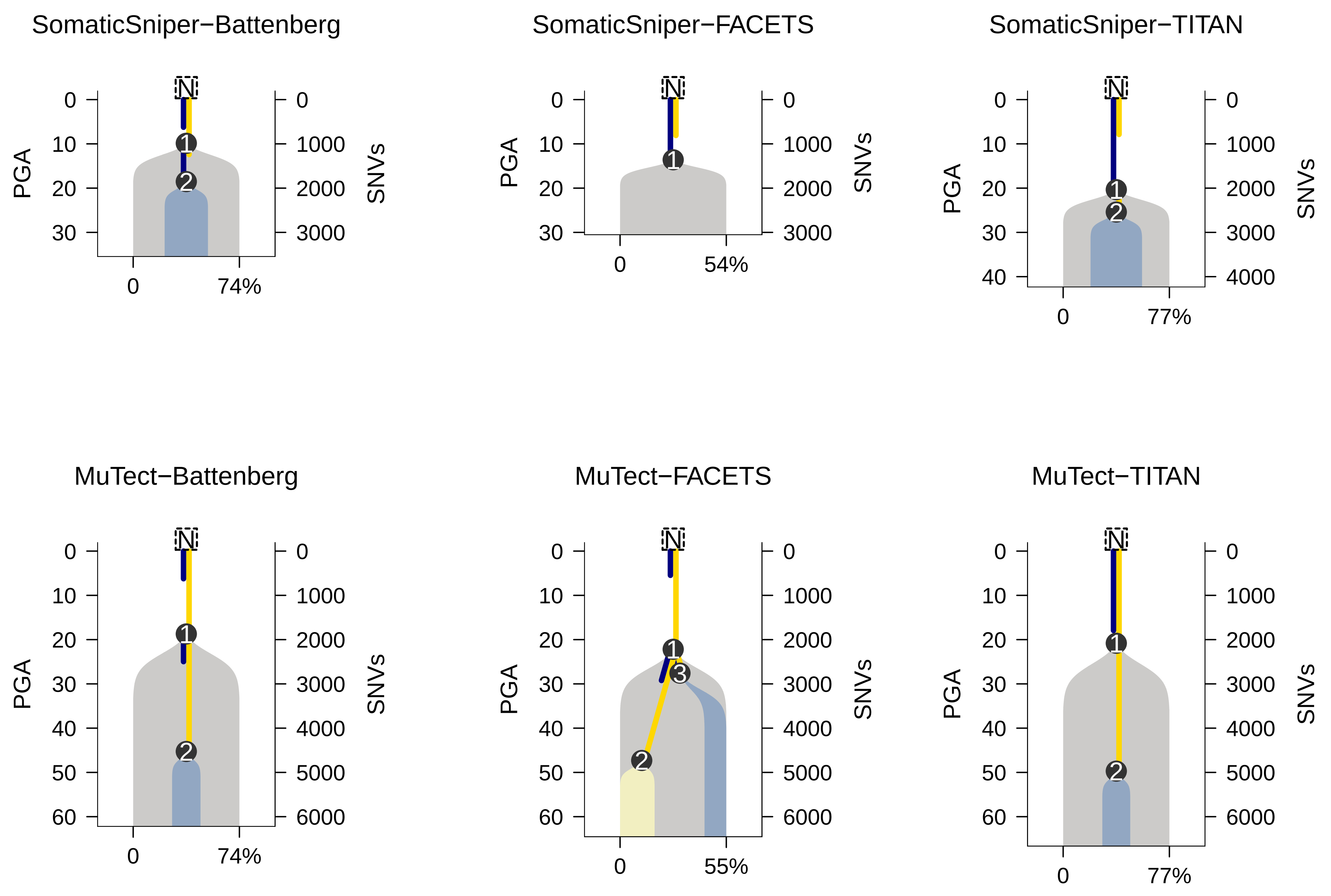

CPCG0334

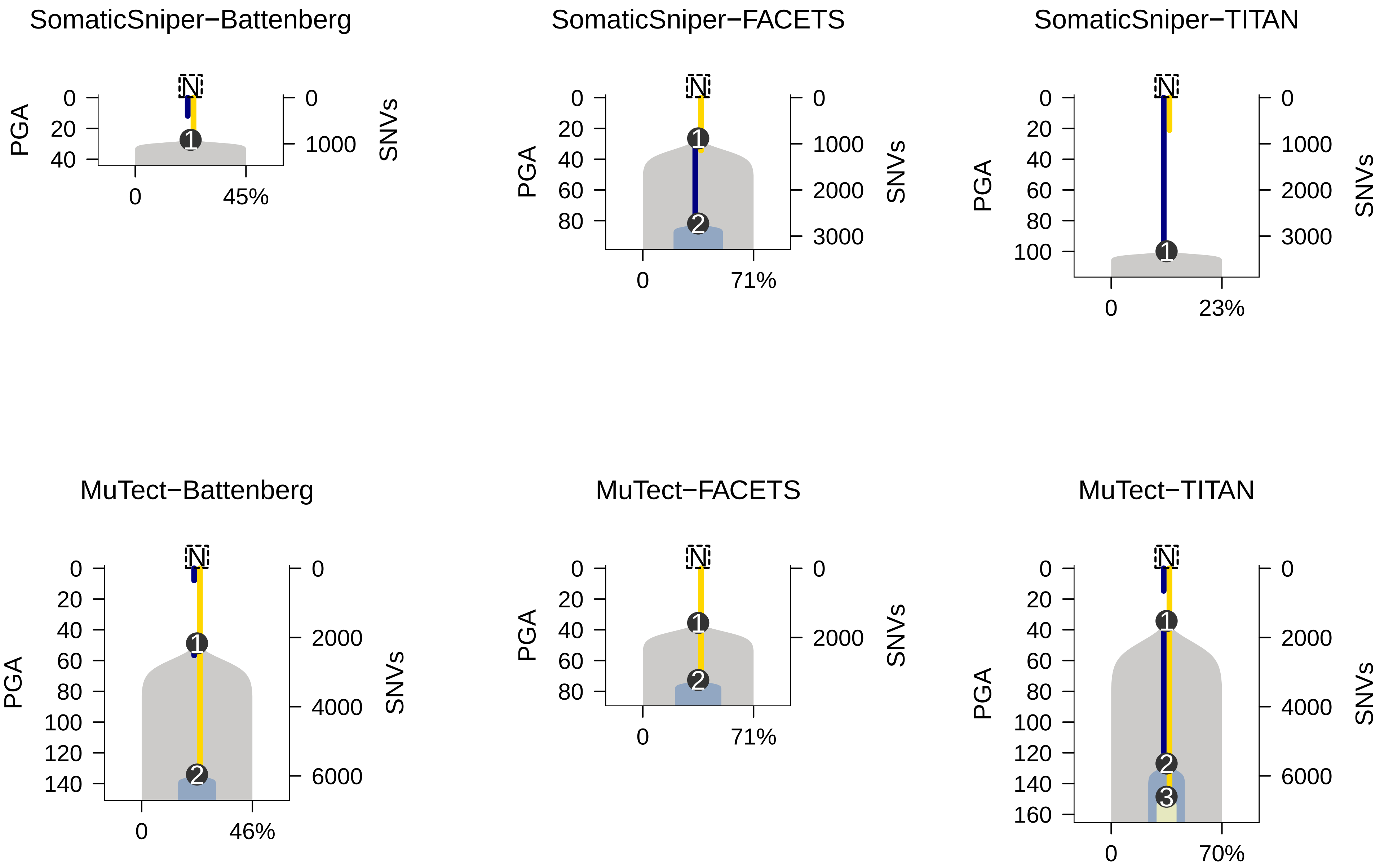
SomaticSniper−Battenberg
SomaticSniper−FACETS
SomaticSniper−TITAN
MuTect−Battenberg
MuTect−FACETS
MuTect−TITAN
PGA
SNVs
N
1
2
3
0
20
40
60
80
100
120
140
160
1000
2000
3000
4000
6000
45%
71%
23%
46%
70%

CPCG0361−B1F1

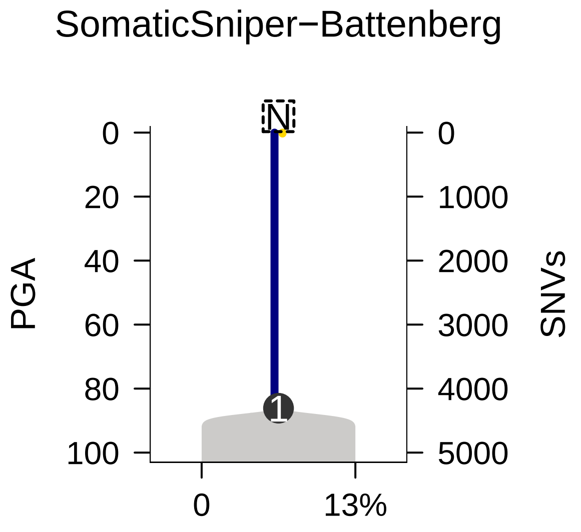

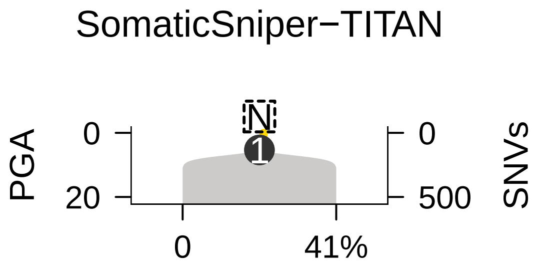

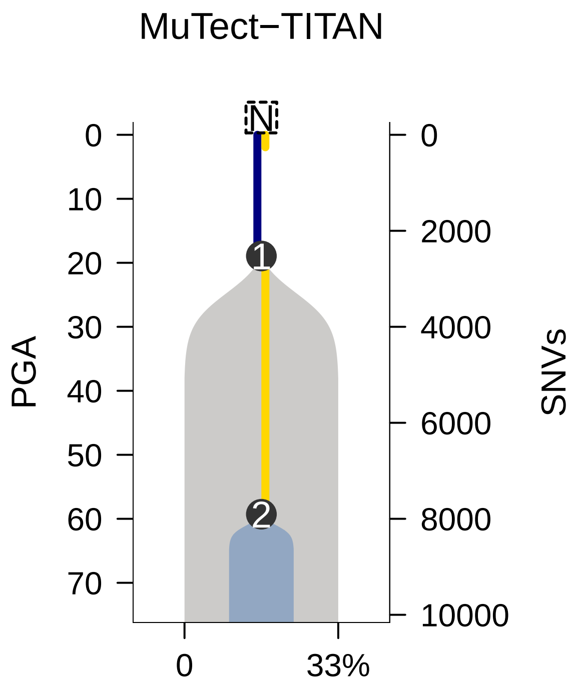

CPCG0361−B1F2

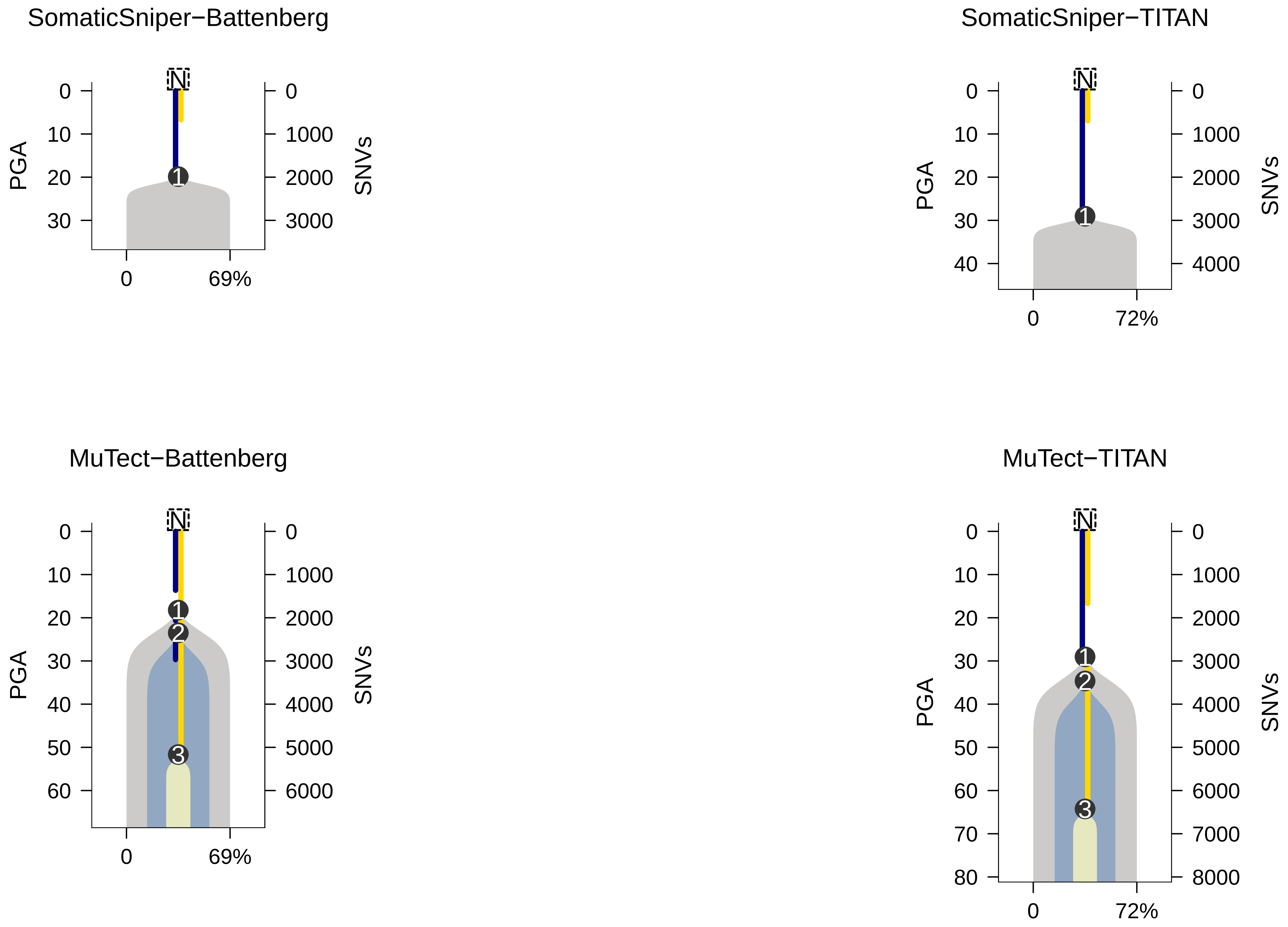

CPCG0361−B1F3

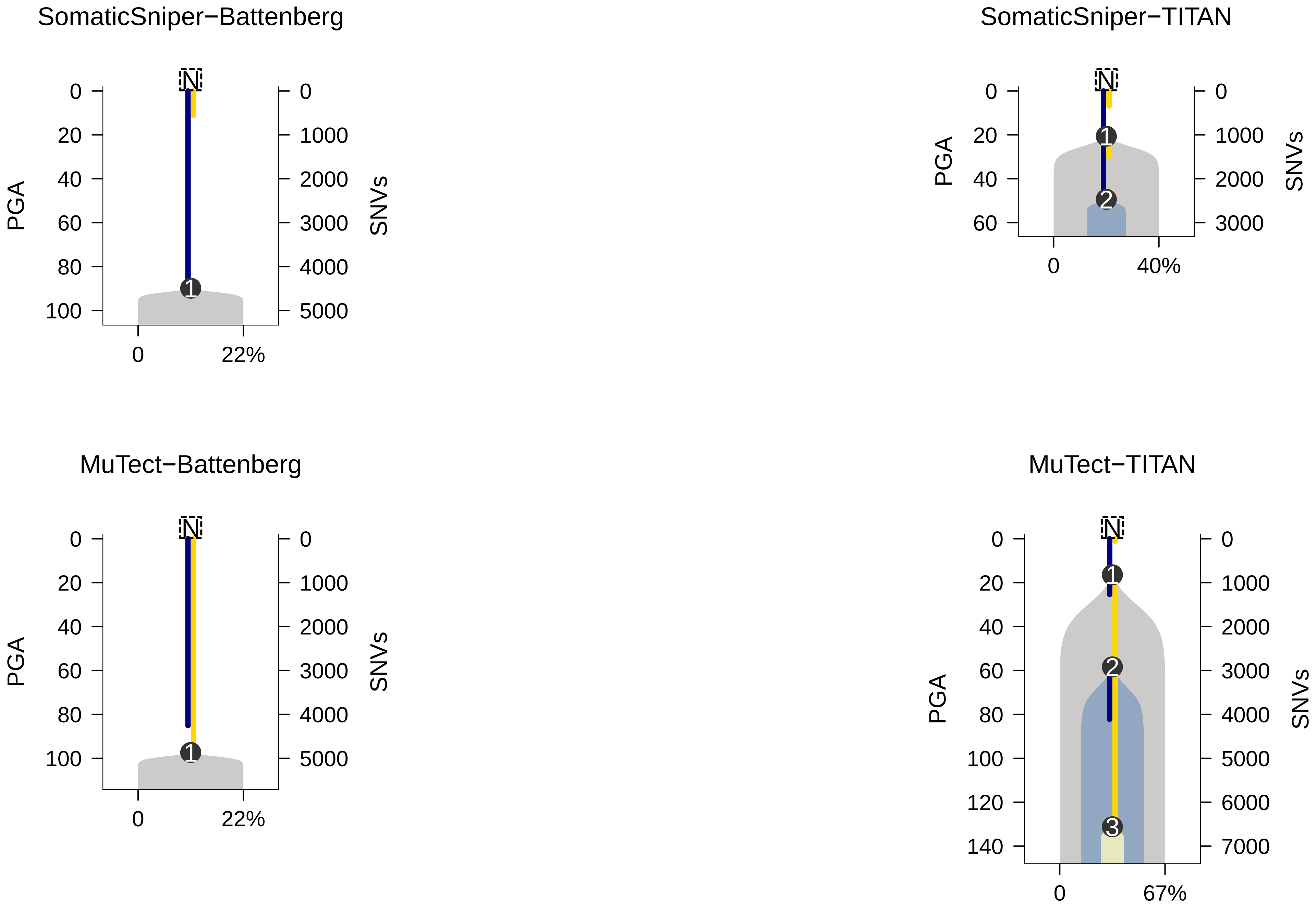

CPCG0361−B1F4

SomaticSniper−Battenberg

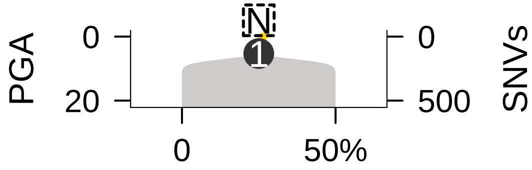

SomaticSniper−TITAN

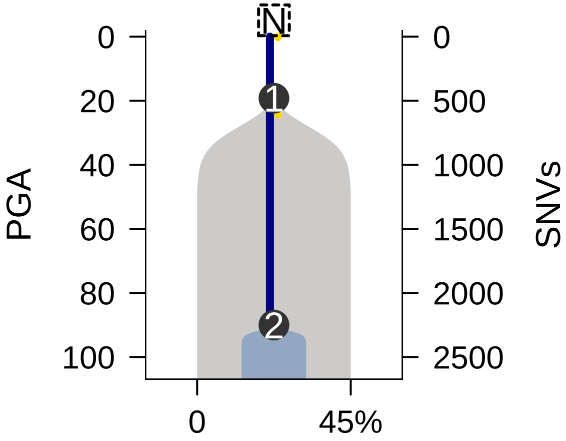

MuTect−Battenberg

Poly−tumour

MuTect−TITAN

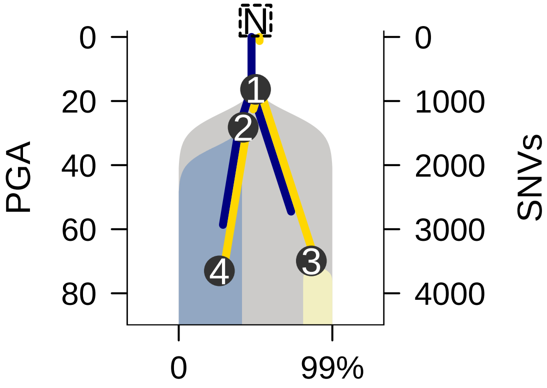

CPCG0364−B1F1

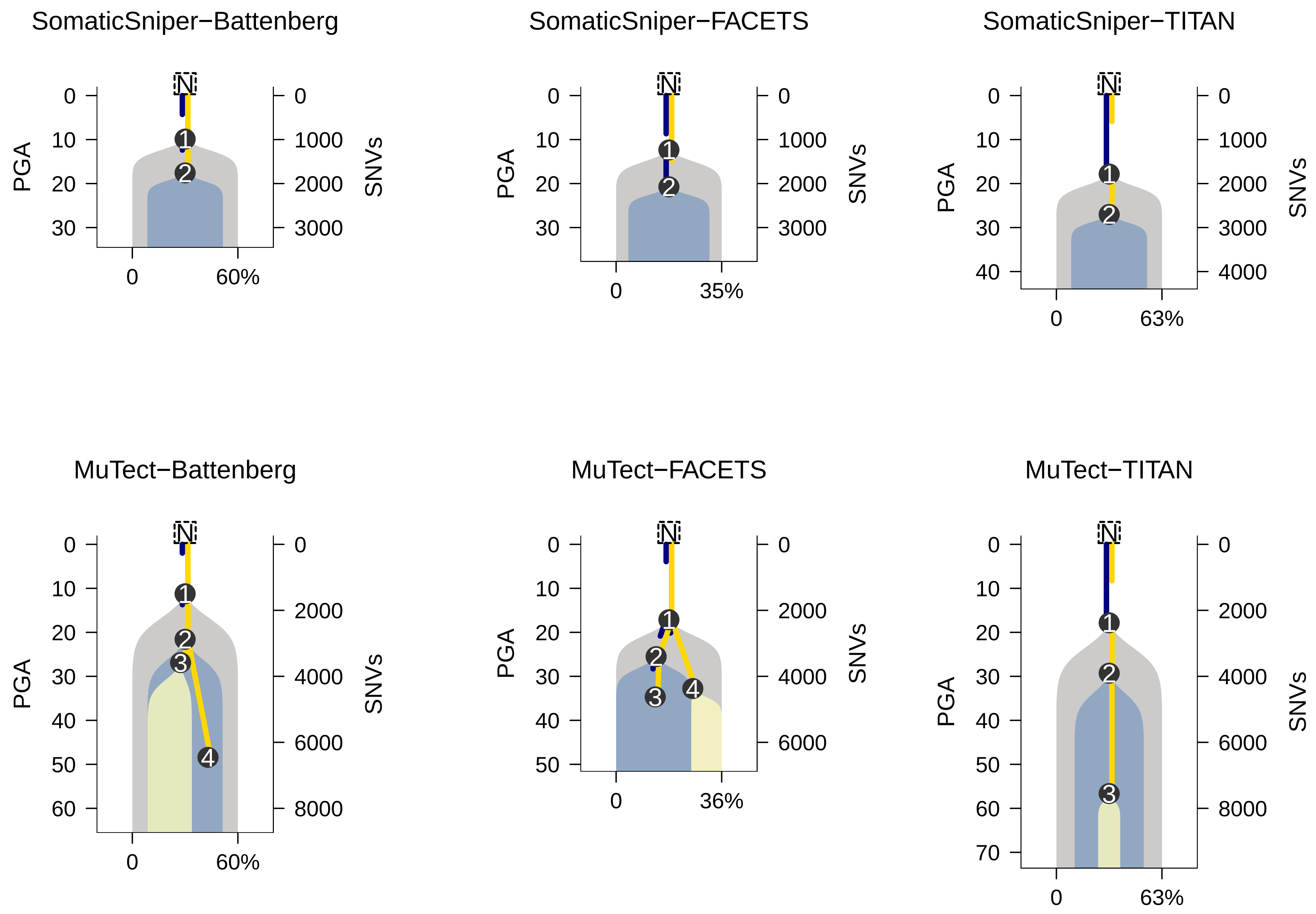

CPCG0364−B1F2

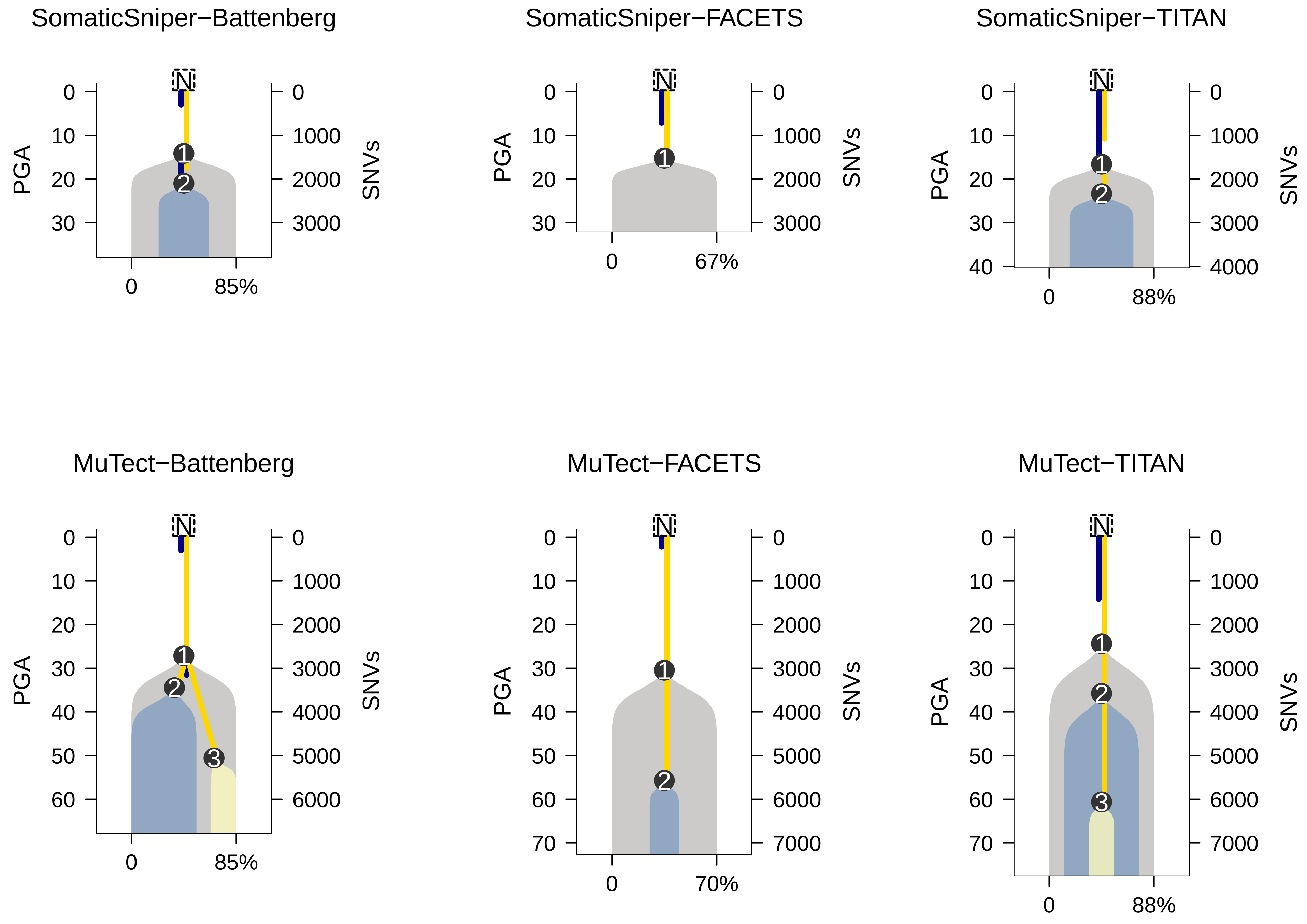

CPCG0364−B1F3

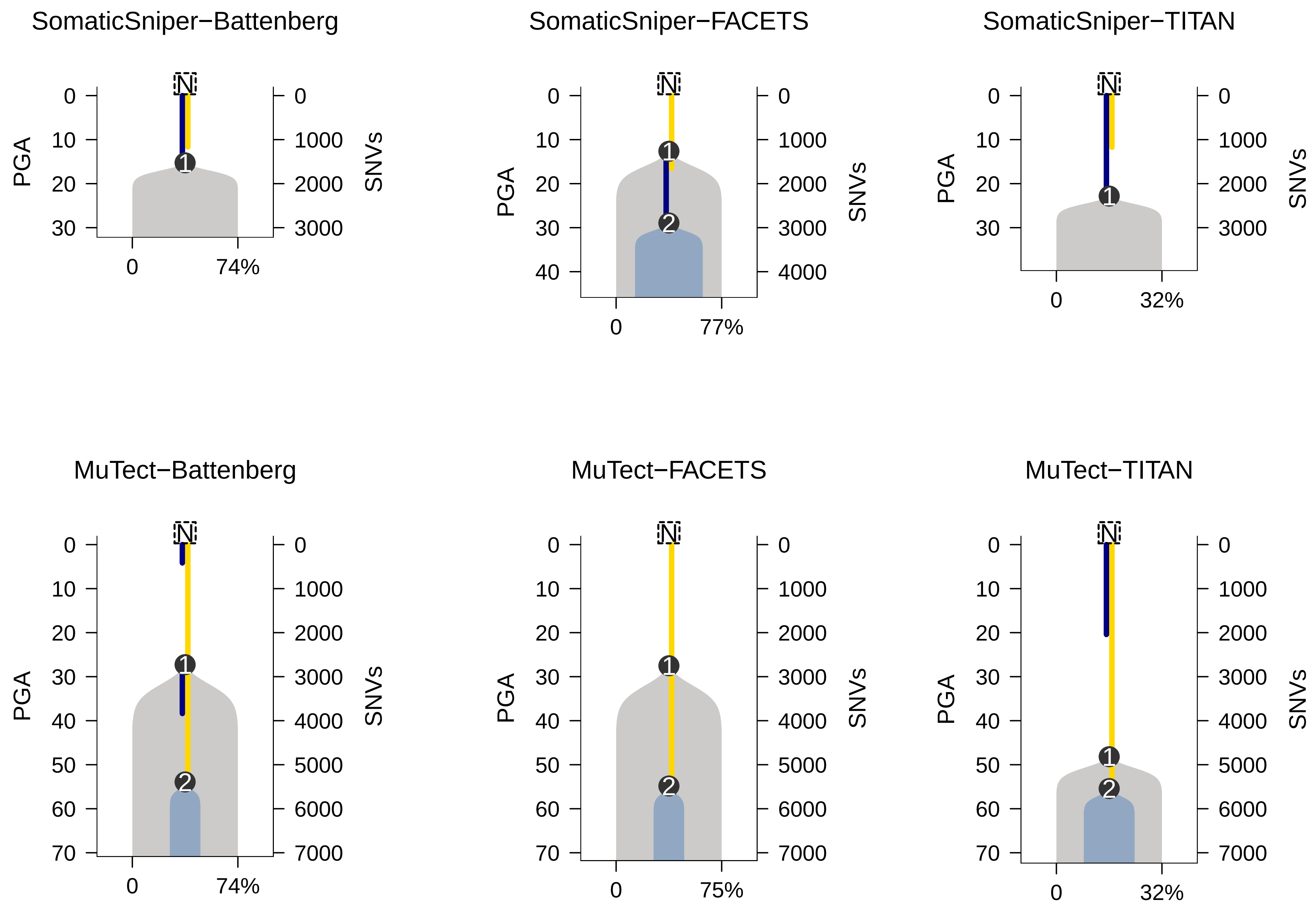

CPCG0364−B1F4

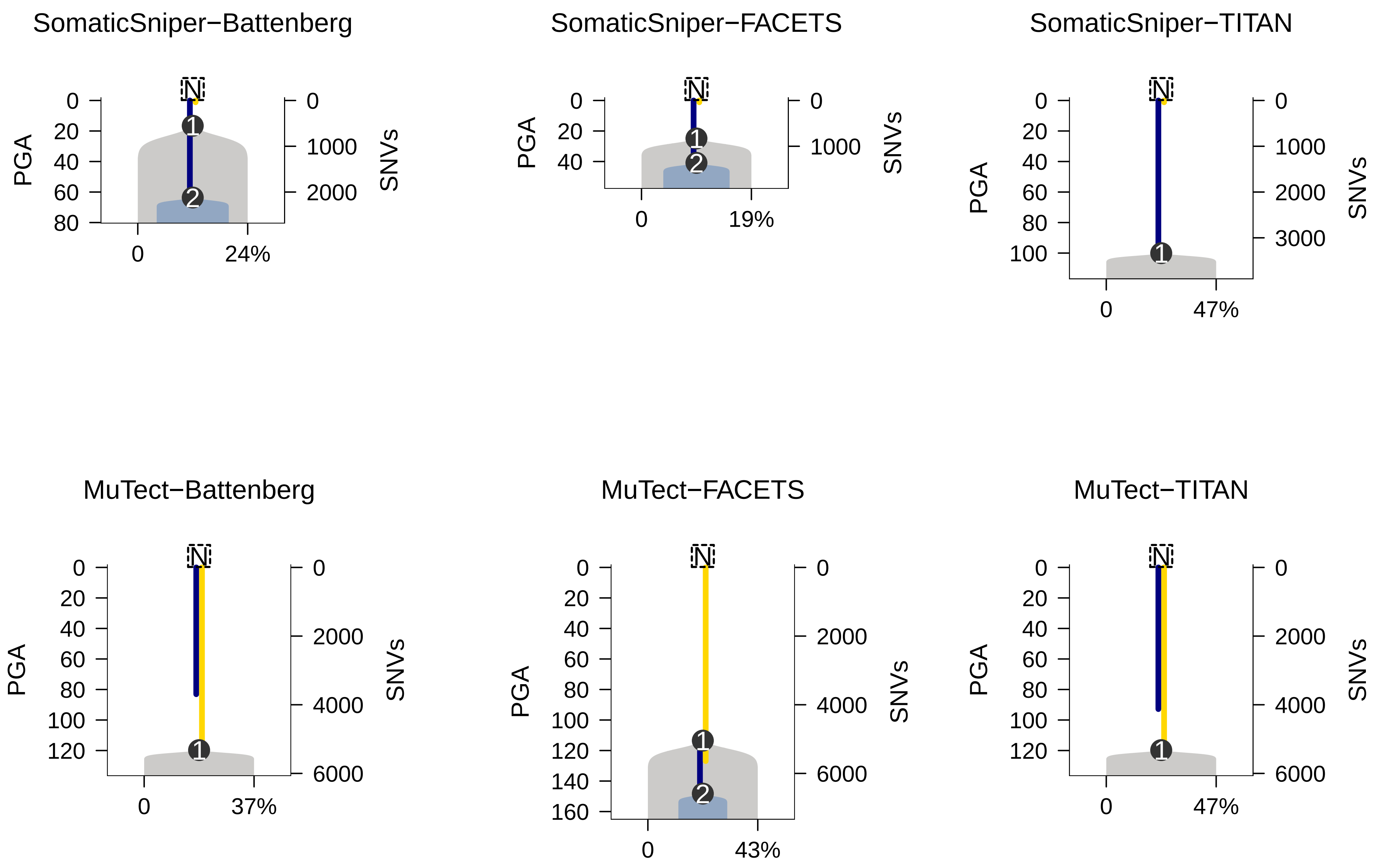

CPCG9001−B1P1

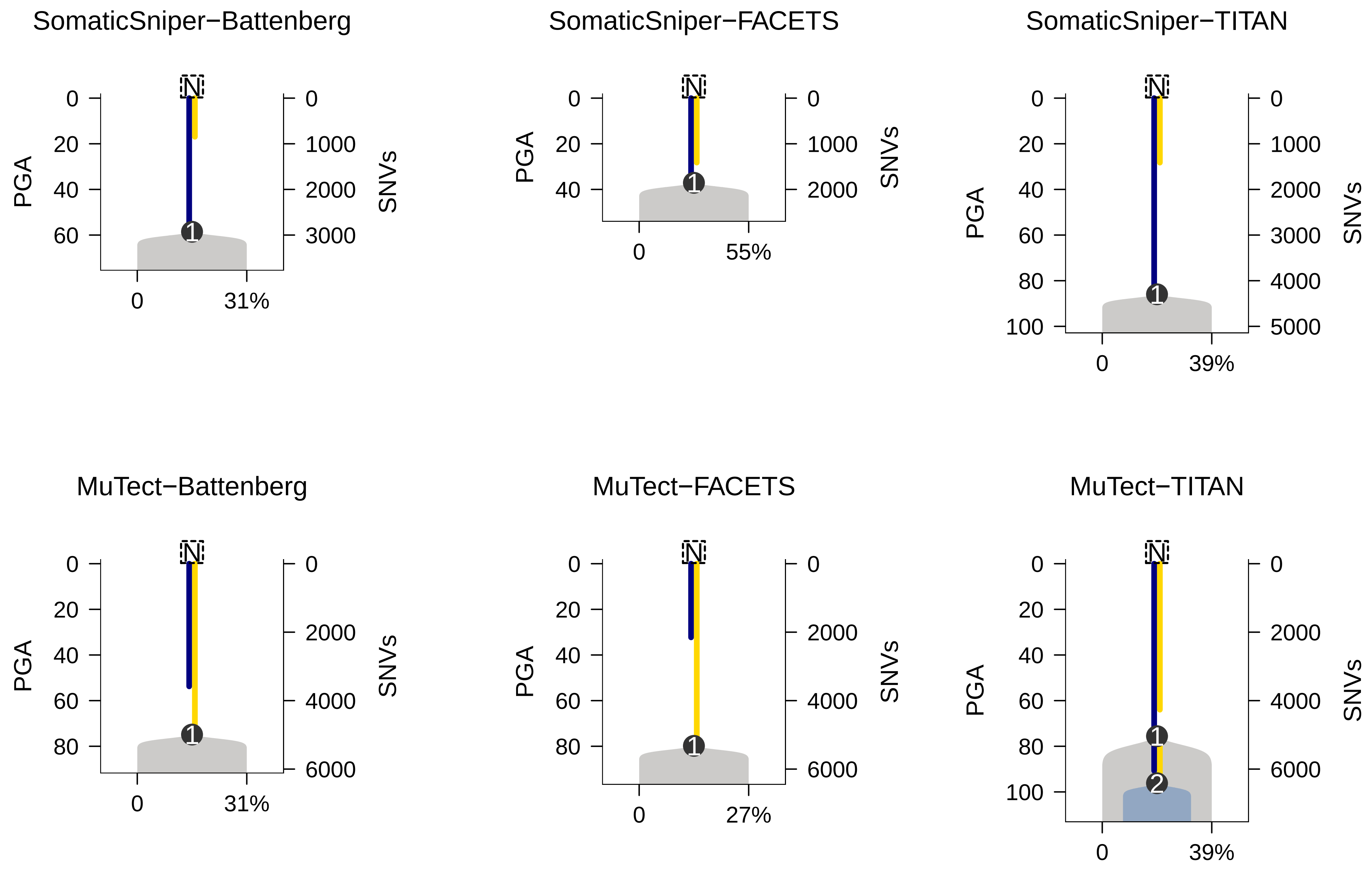

CPCG9001−B1P2

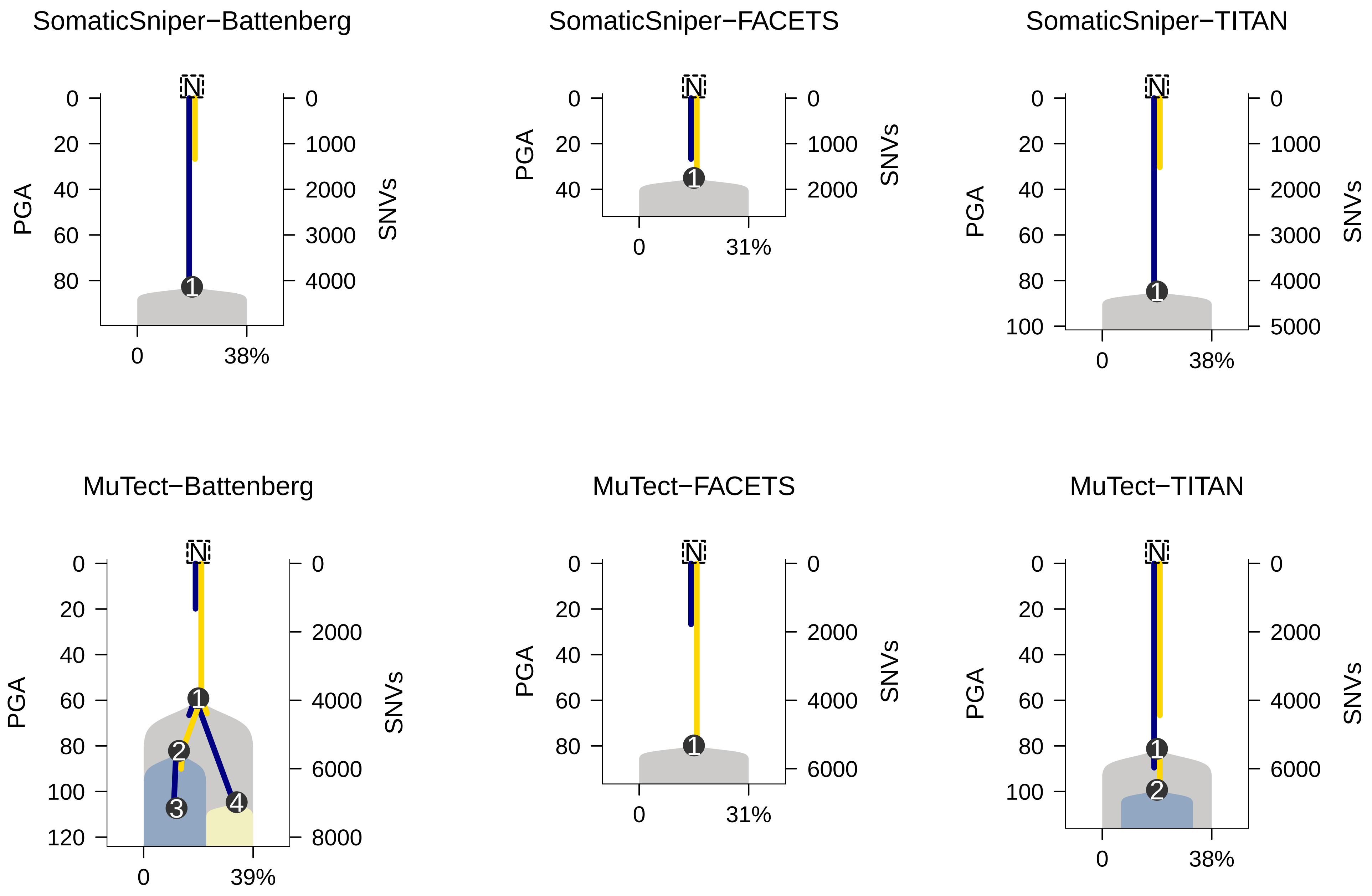

CPCG9002−B1P1

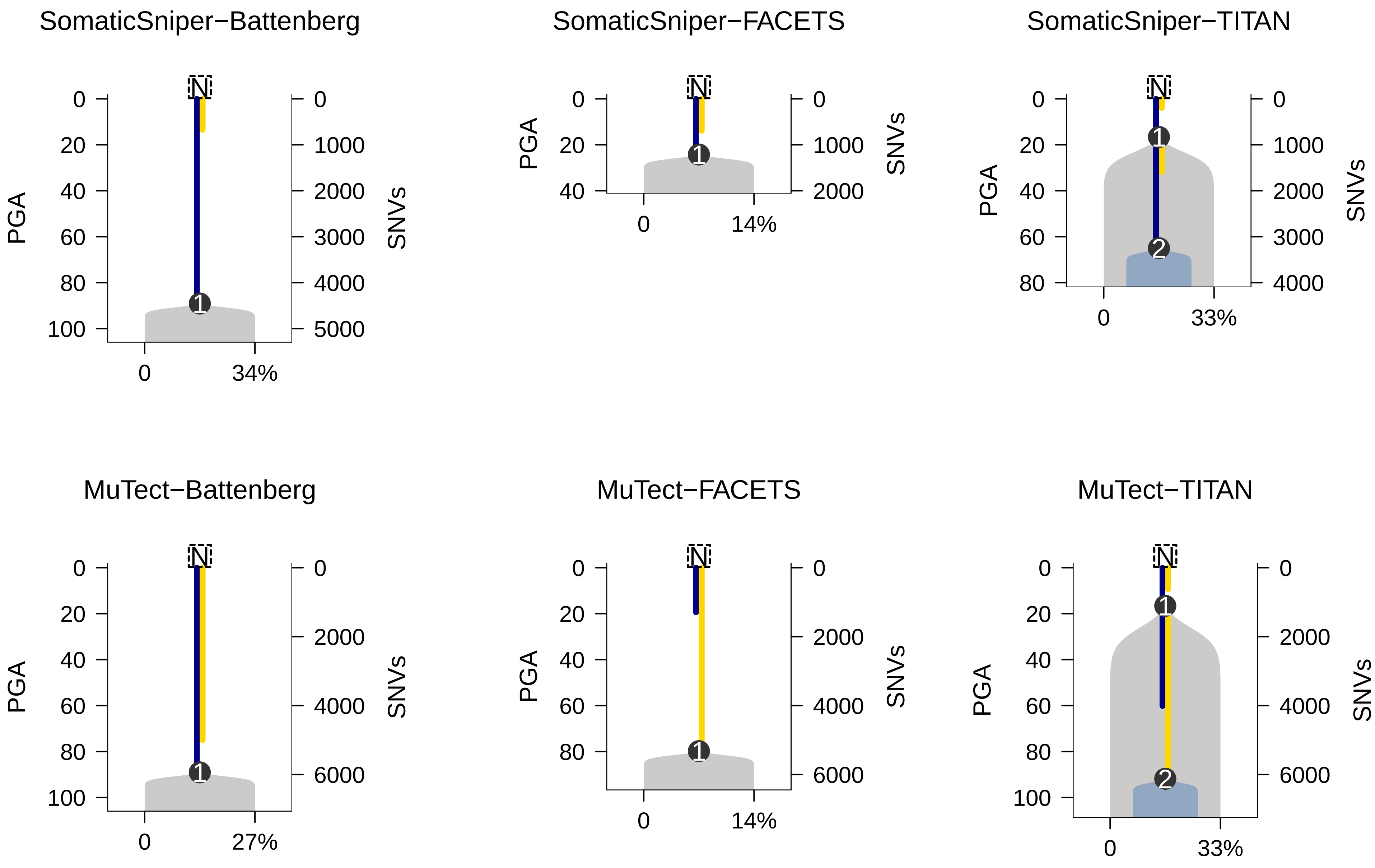

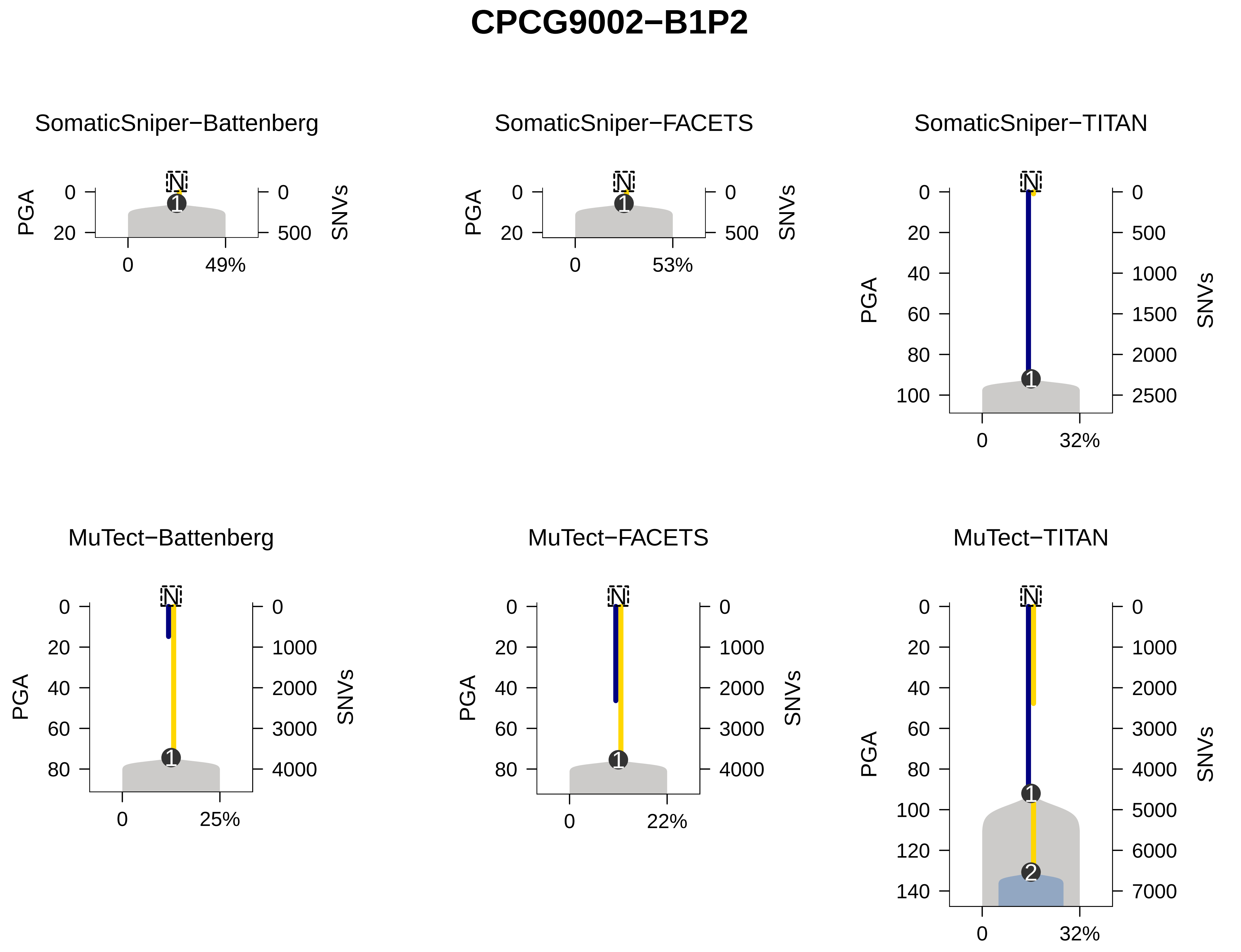
CPCG9002−B1P2
SomaticSniper−Battenberg
SomaticSniper−FACETS
SomaticSniper−TITAN
MuTect−Battenberg
MuTect−FACETS
MuTect−TITAN
PGA
SNVs
N
1
2
0
49%
53%
32%
25%
22%

CPCG9003−B1P1

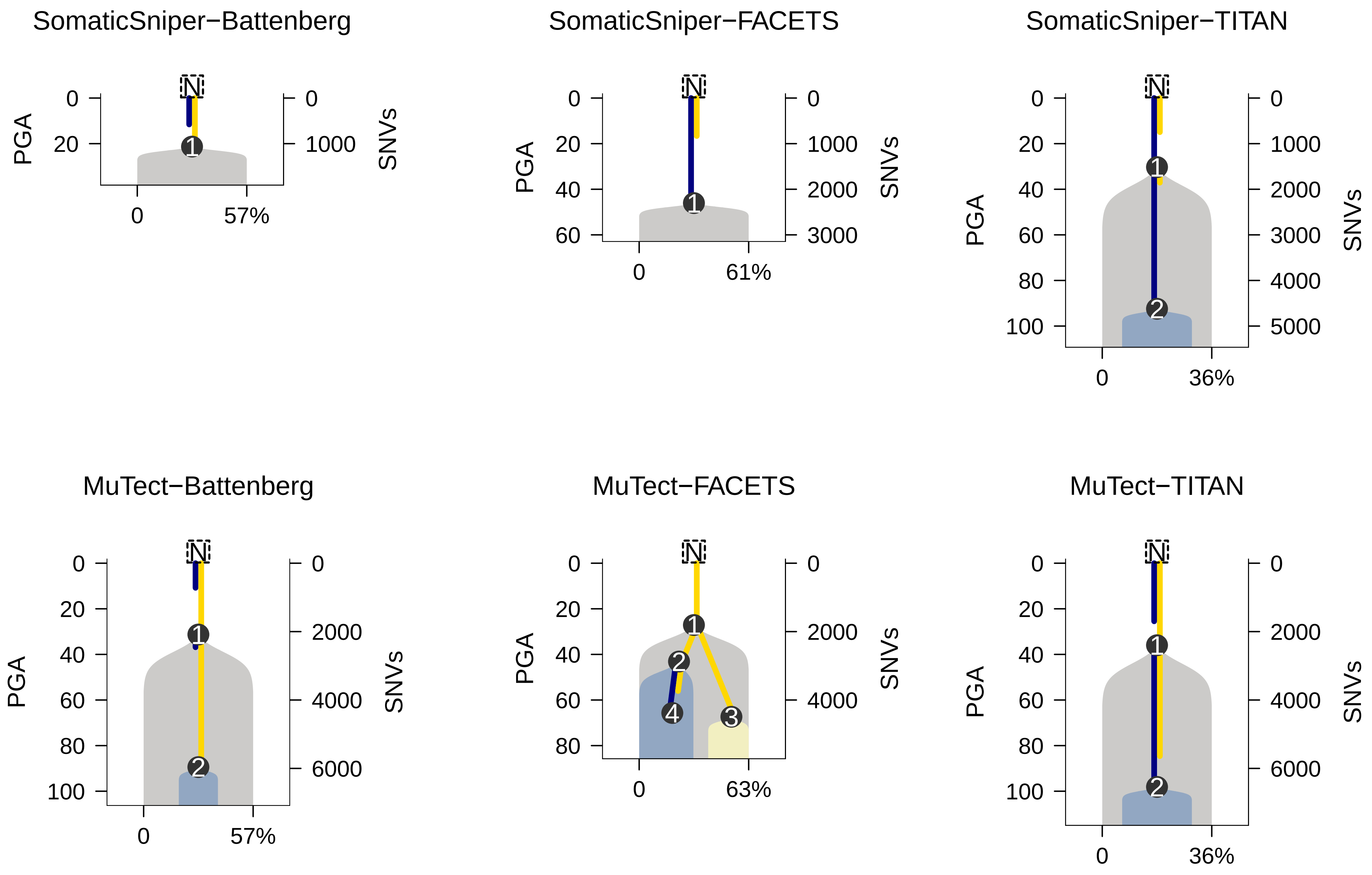

CPCG9003−B1P2

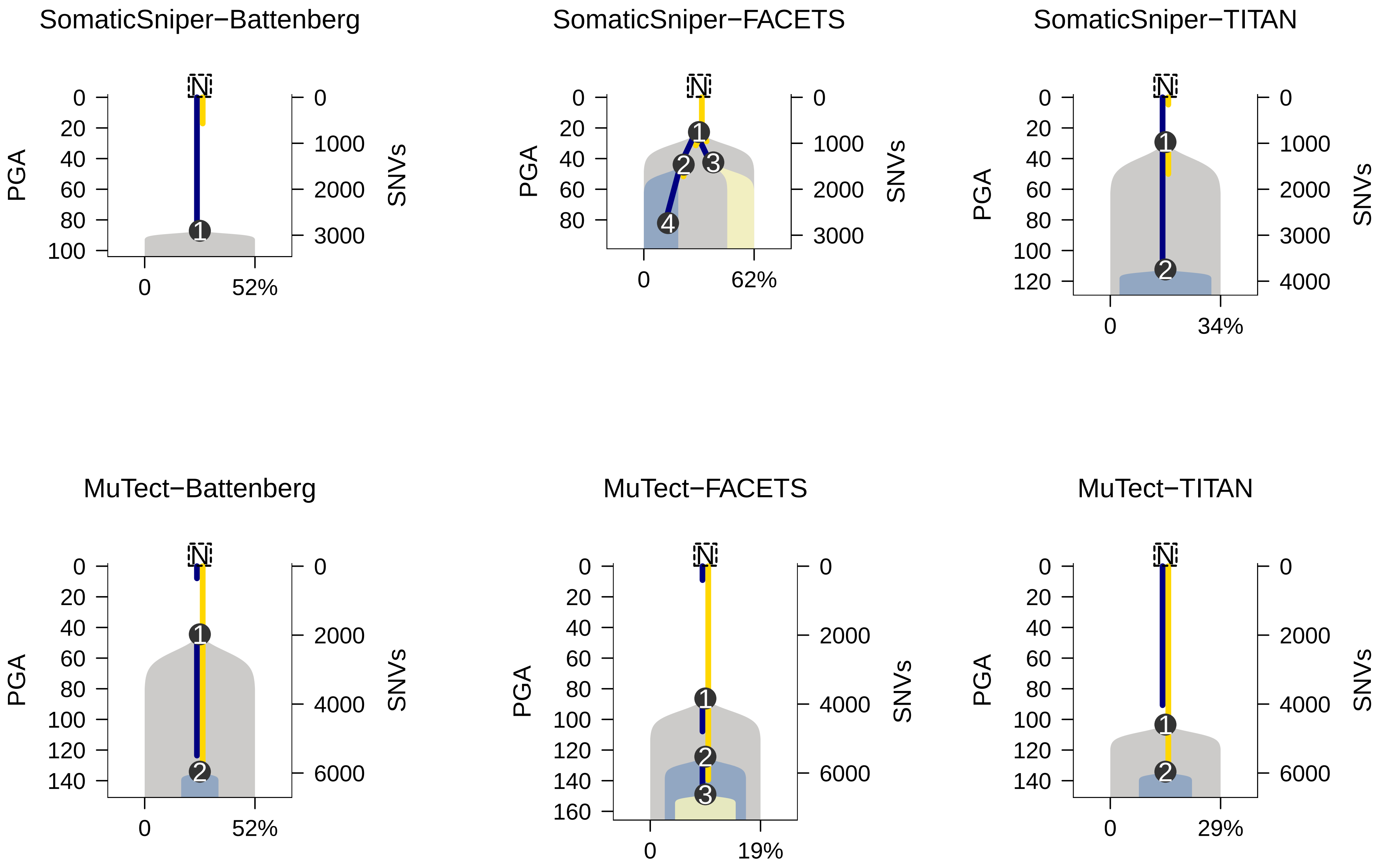

CPCG9005−B1P1

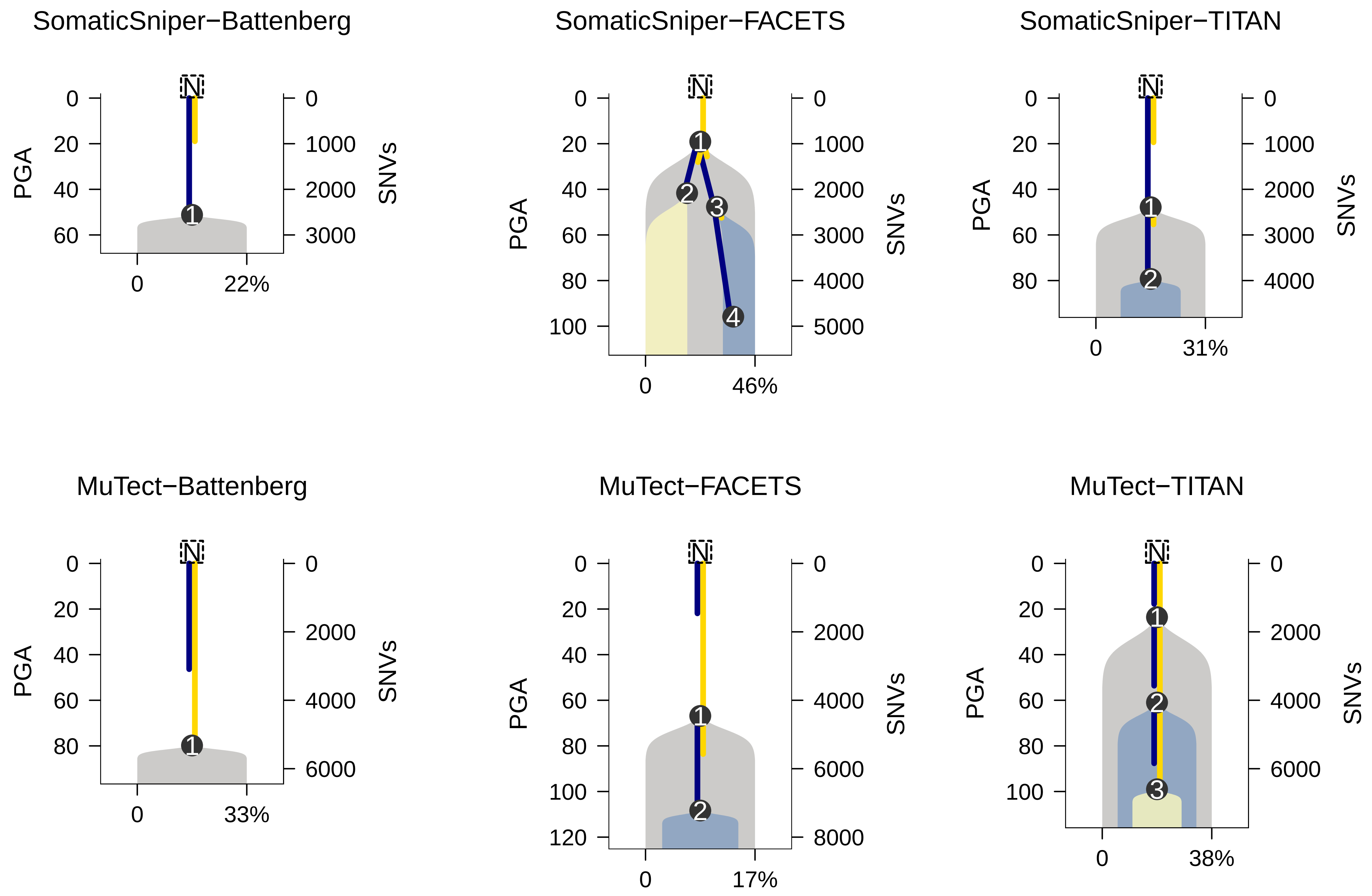

CPCG9005−B1P2

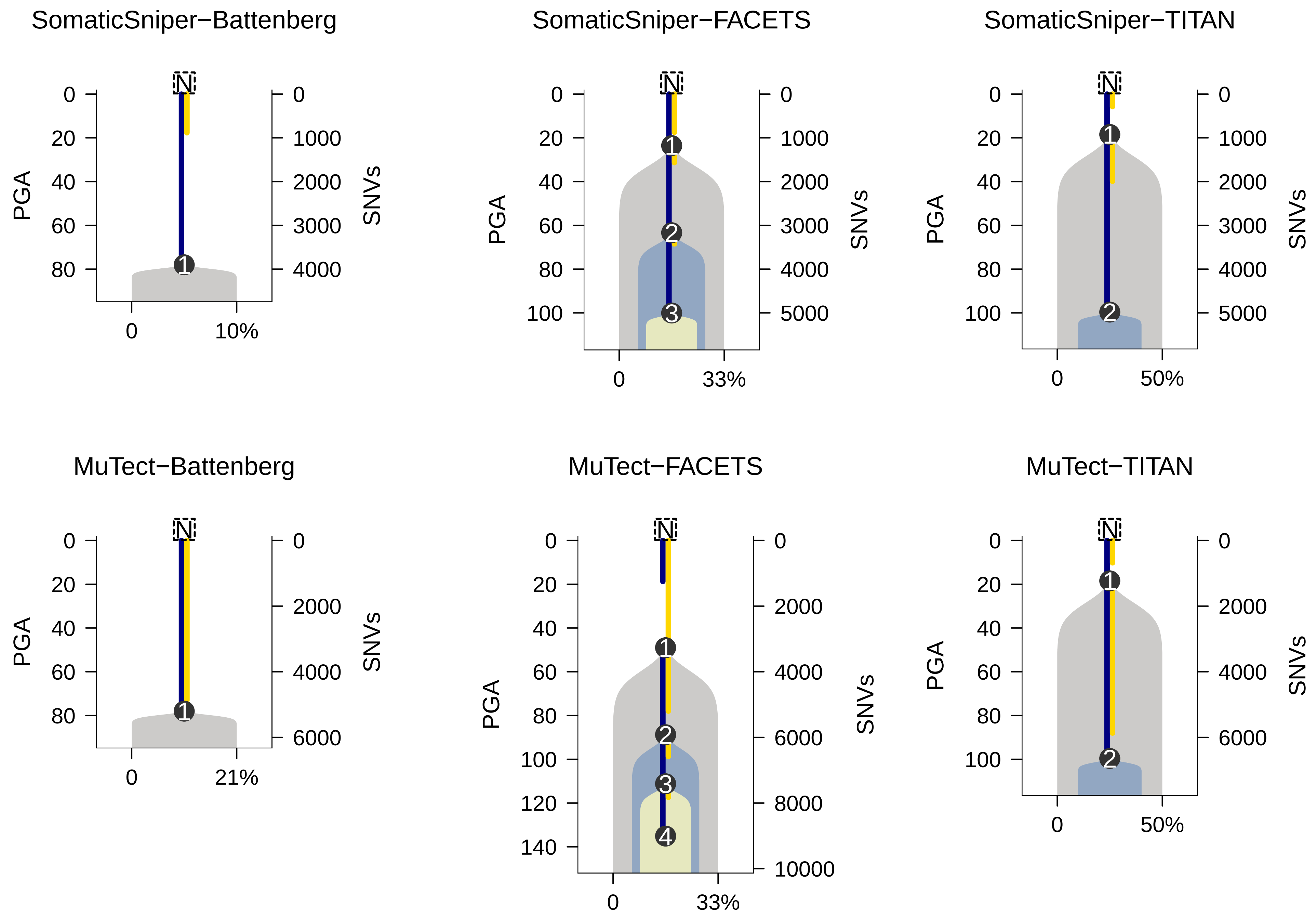

CPCG0100.MR MuTect−Battenberg

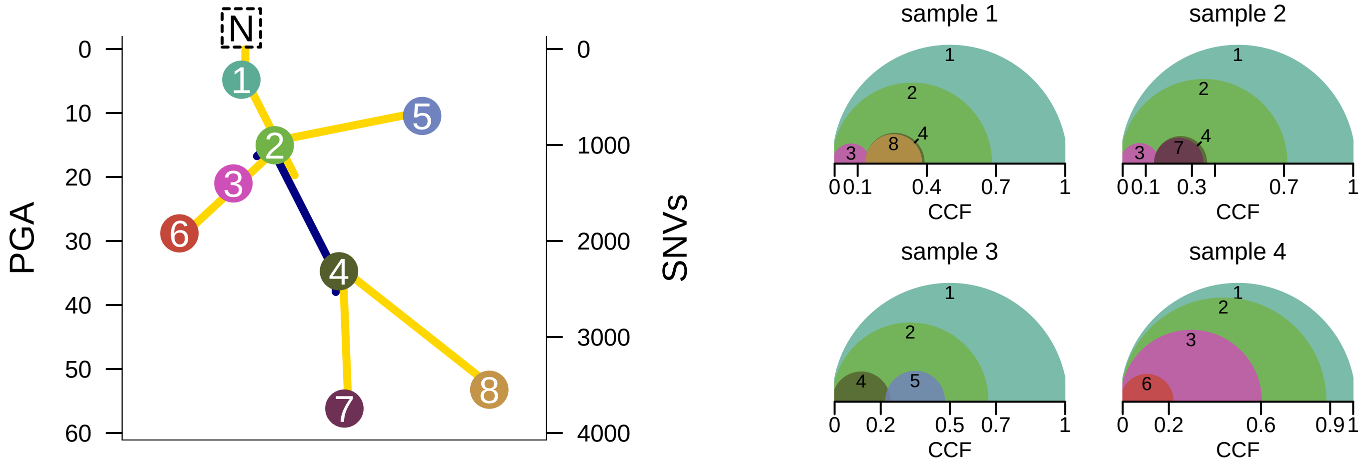

CPCG0100.MR MuTect−TITAN

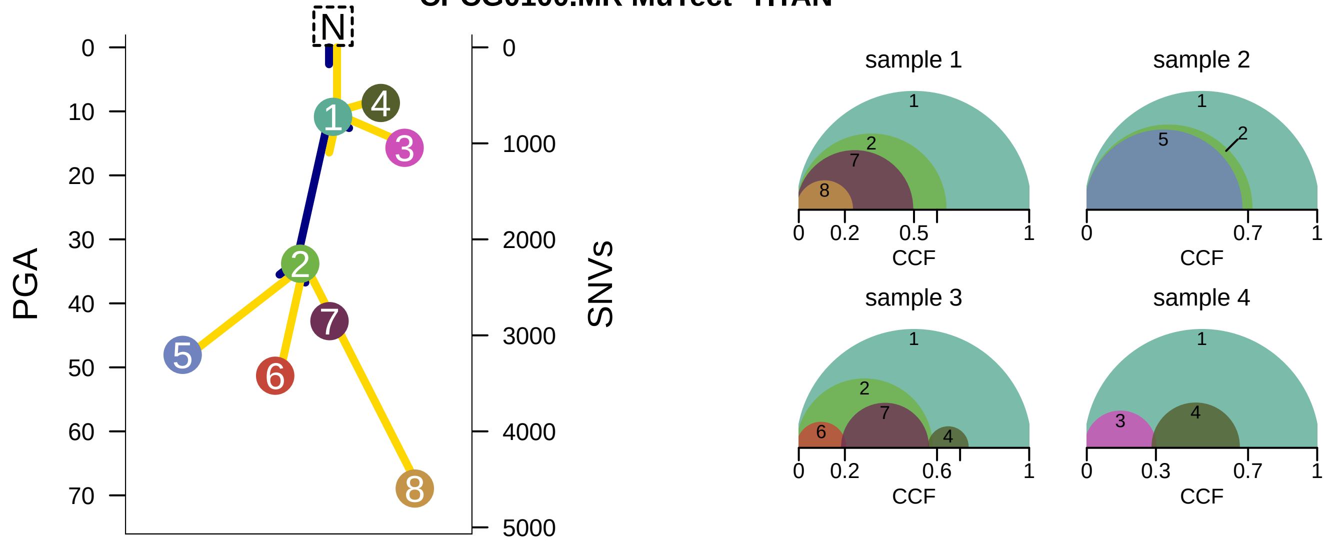

CPCG0100.MR MuTect−FACETS

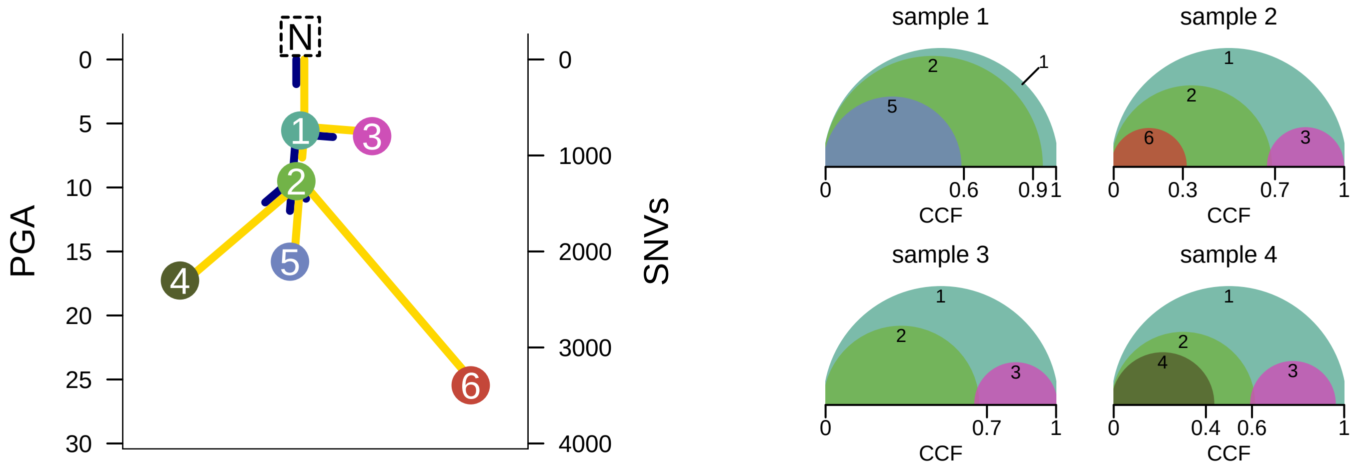

CPCG0100.MR SomaticSniper−Battenberg

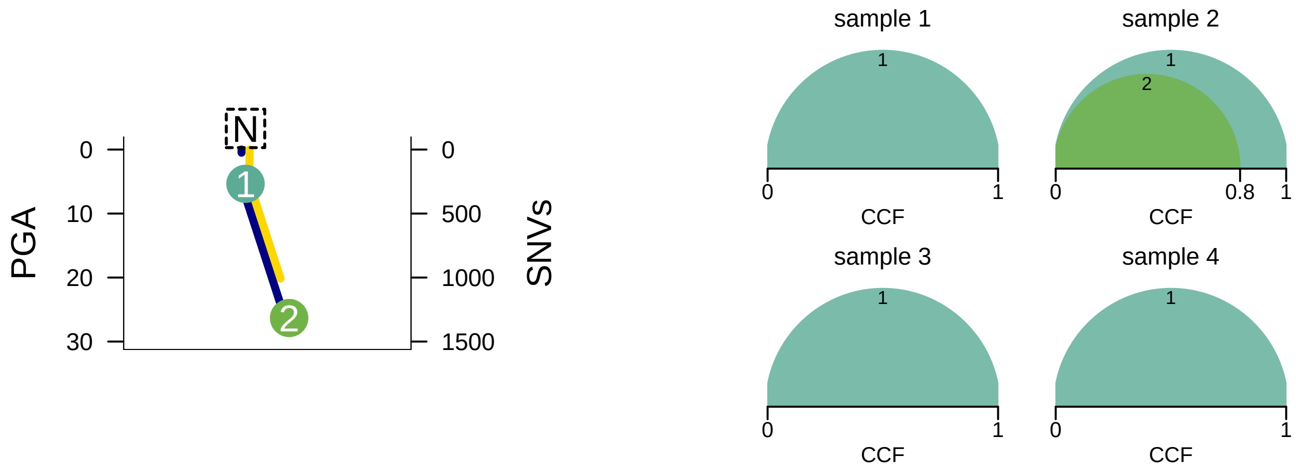

CPCG0100.MR SomaticSniper−TITAN

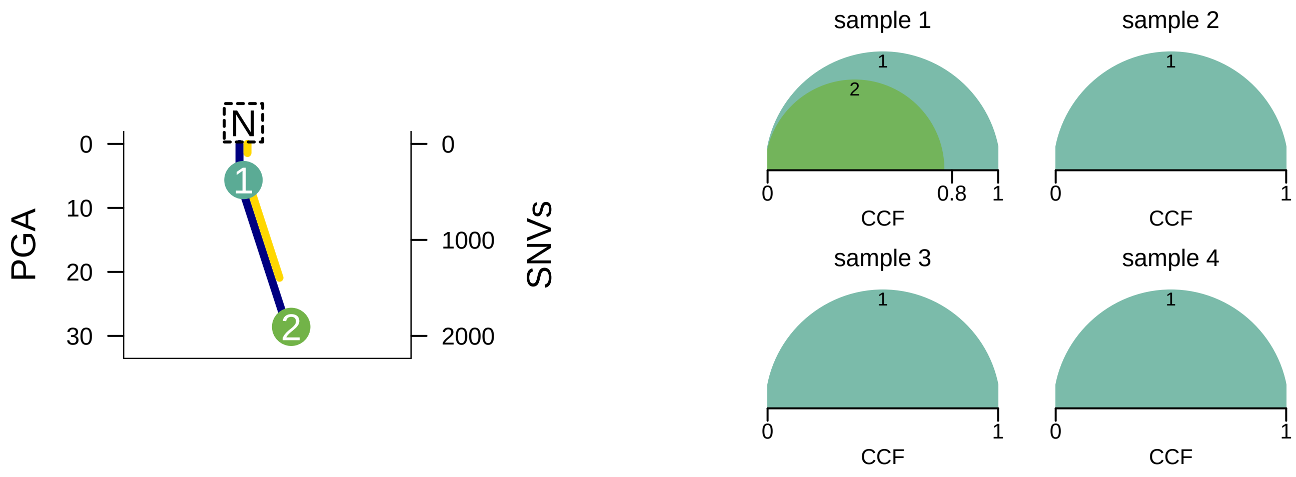

CPCG0100.MR SomaticSniper−FACETS

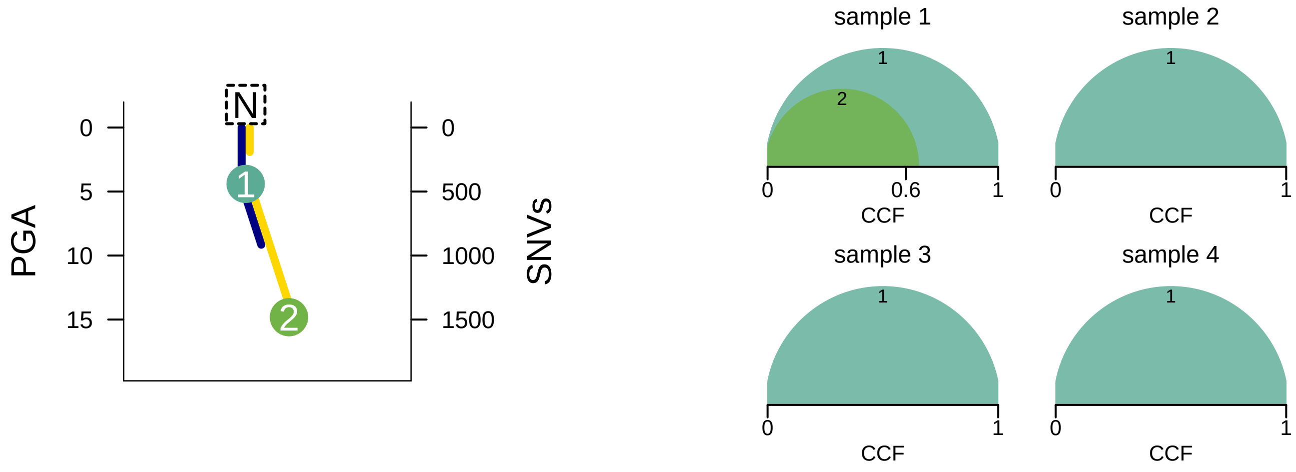

CPCG0103.MR MuTect−Battenberg

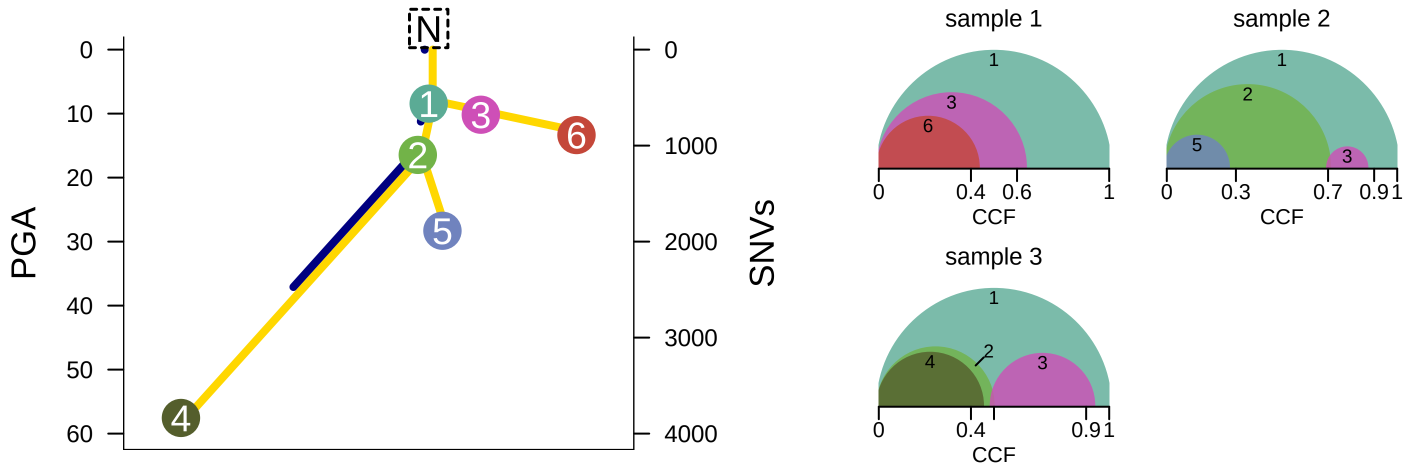

CPCG0103.MR MuTect−TITAN

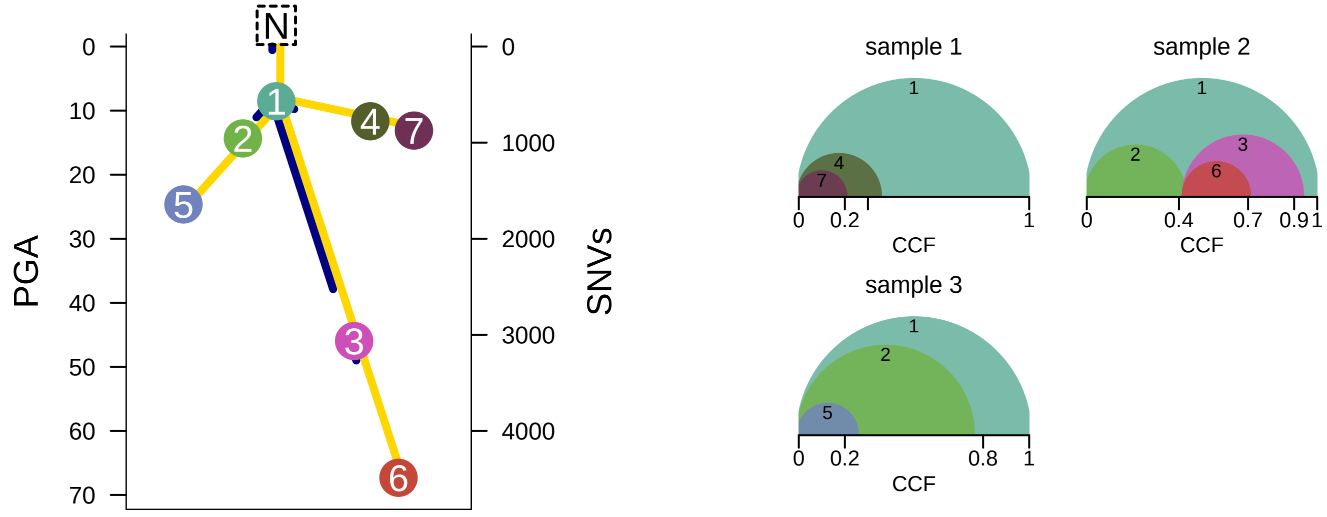

CPCG0103.MR MuTect−FACETS

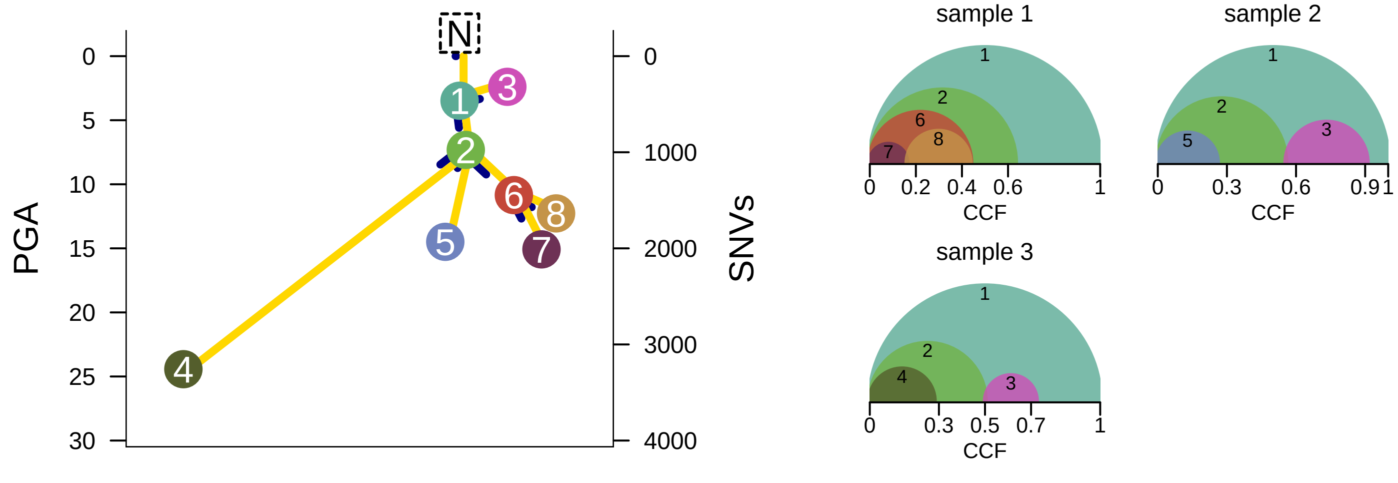

CPCG0103.MR SomaticSniper−Battenberg

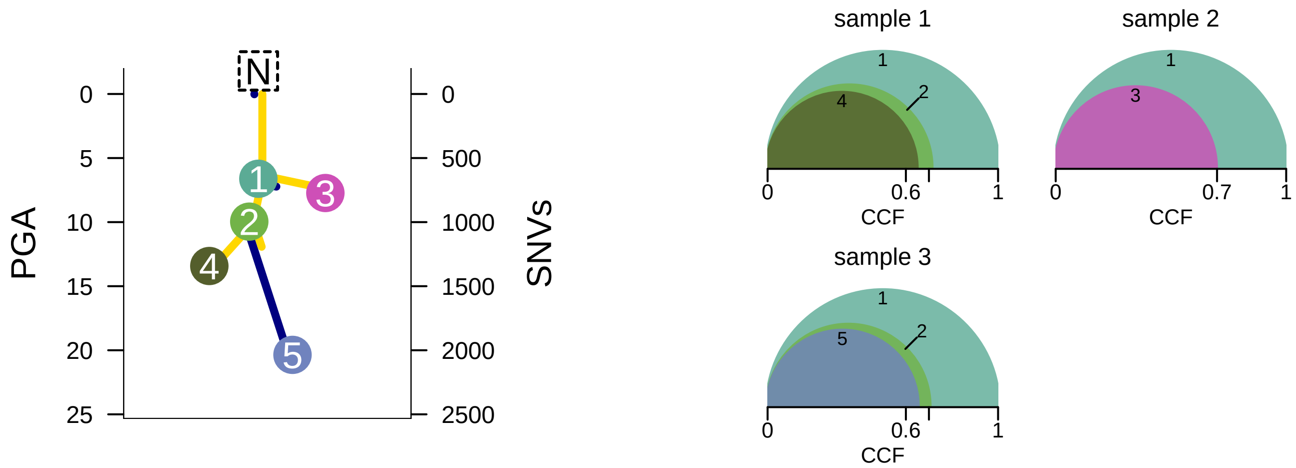

CPCG0103.MR SomaticSniper−TITAN

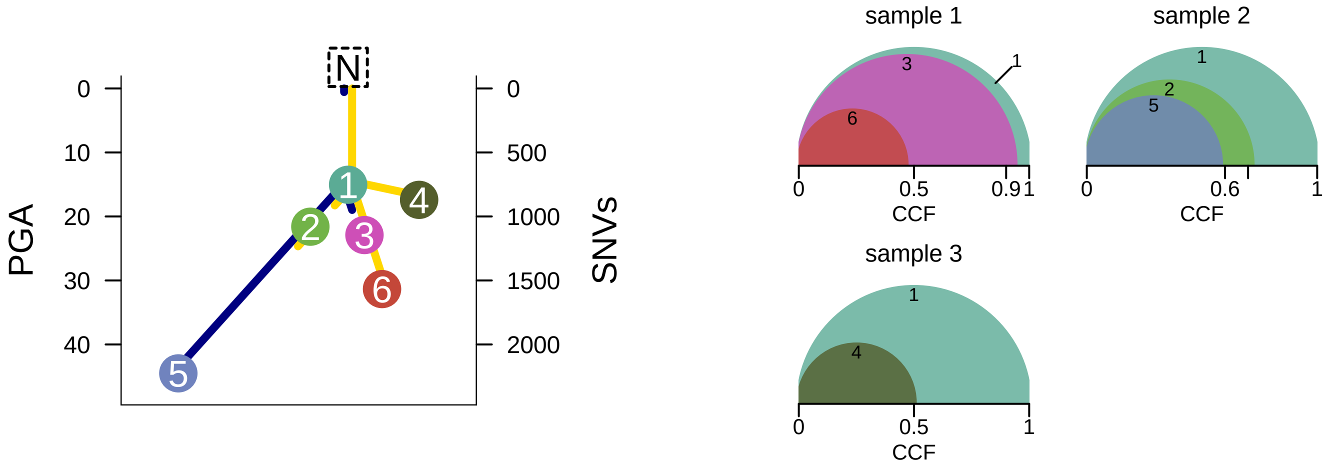

CPCG0103.MR SomaticSniper−FACETS

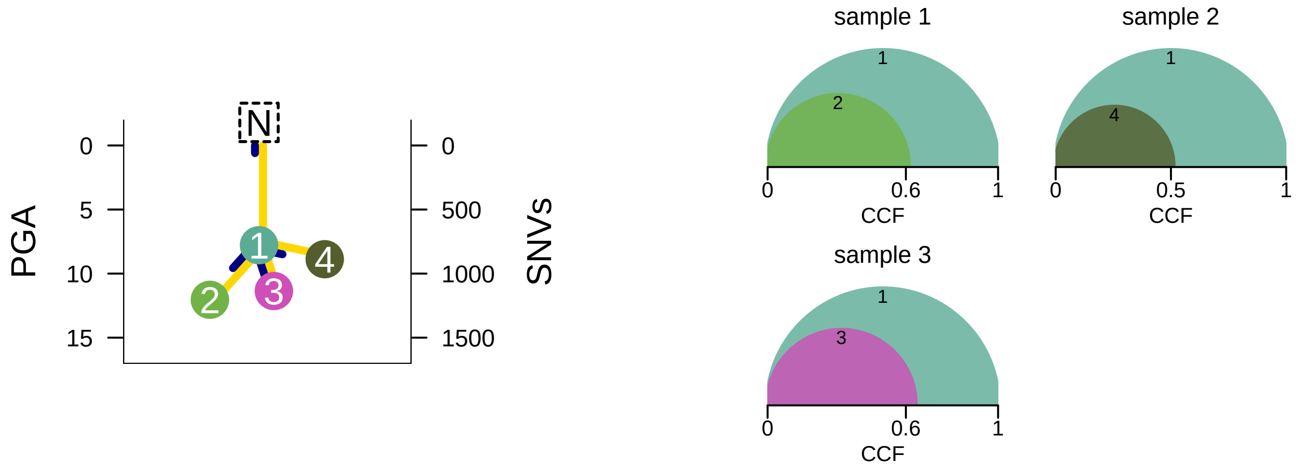

CPCG0260.MR MuTect−Battenberg

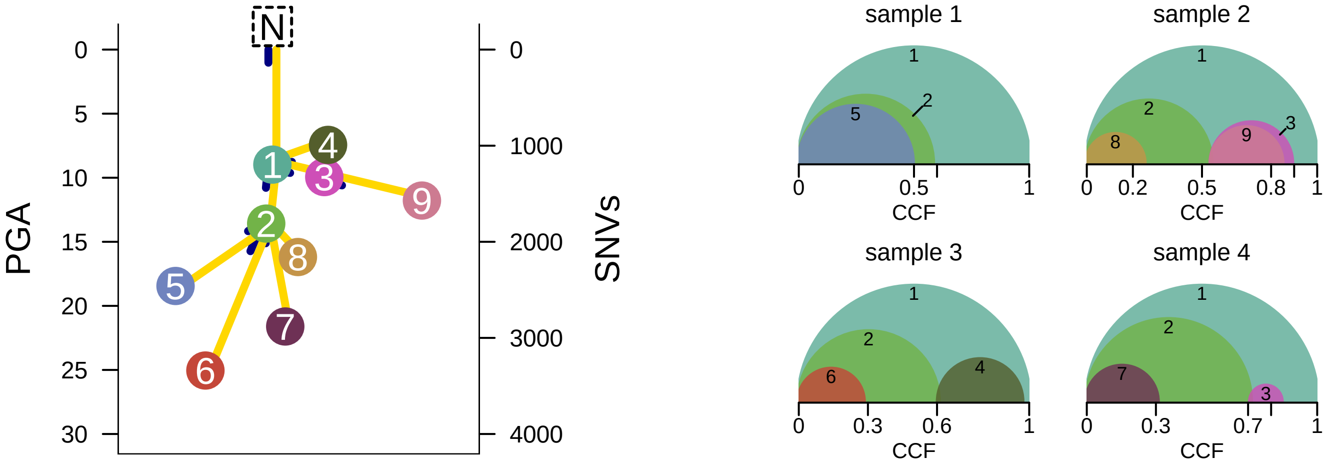

CPCG0260.MR MuTect−TITAN

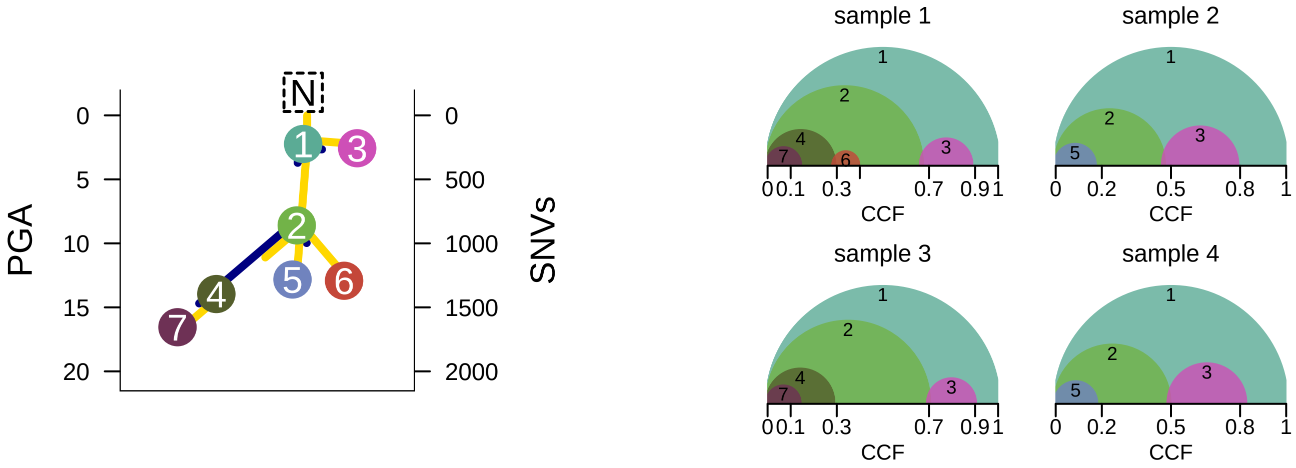

CPCG0260.MR MuTect−FACETS

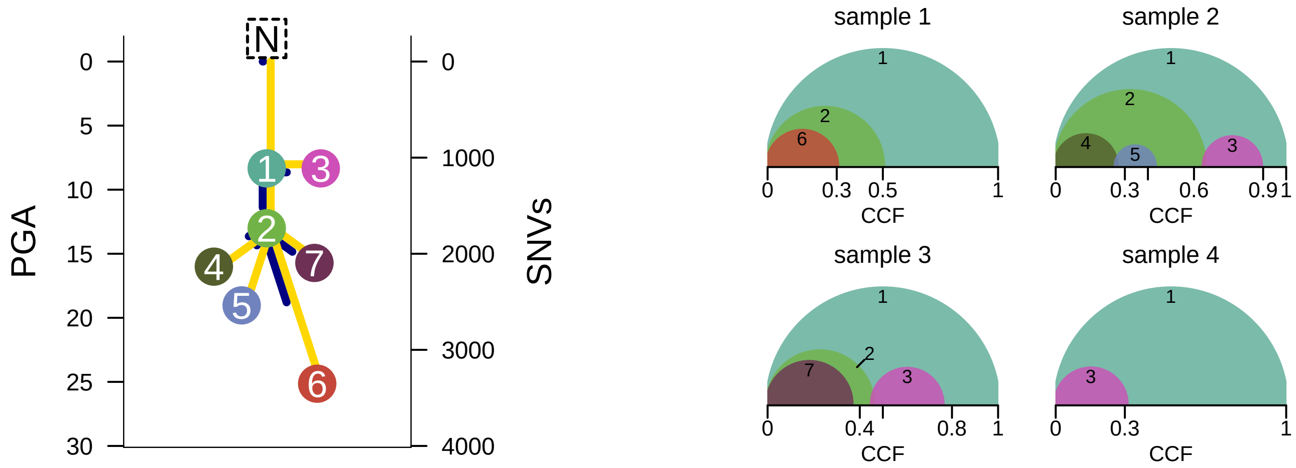

CPCG0260.MR SomaticSniper−Battenberg

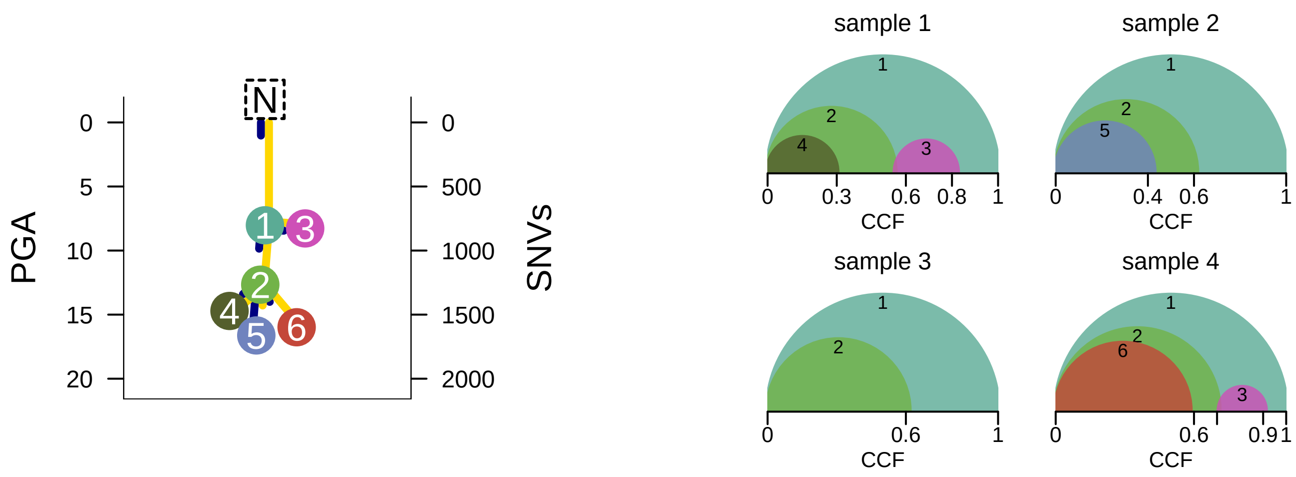

CPCG0260.MR SomaticSniper−TITAN

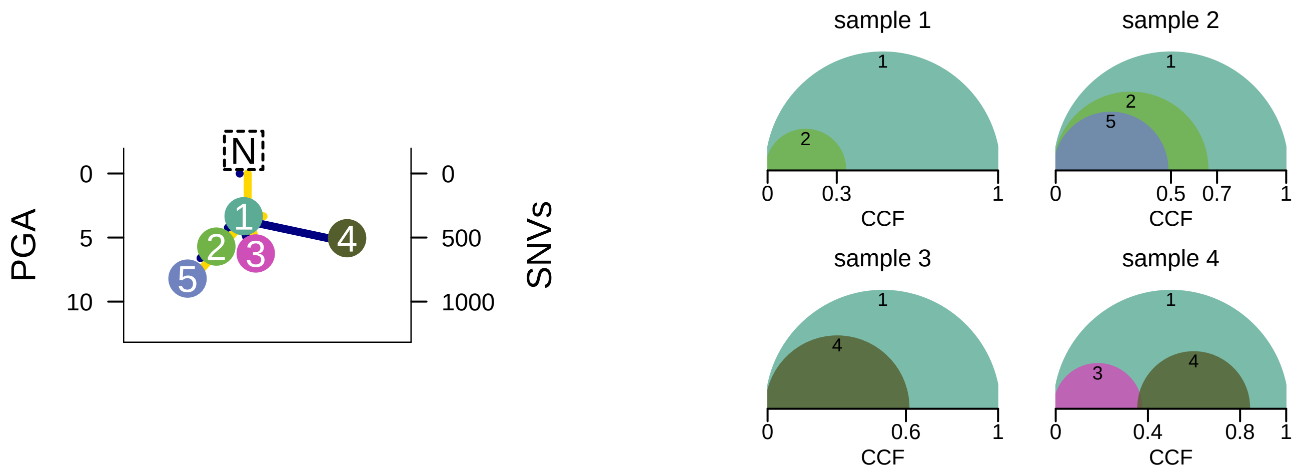

CPCG0260.MR SomaticSniper−FACETS

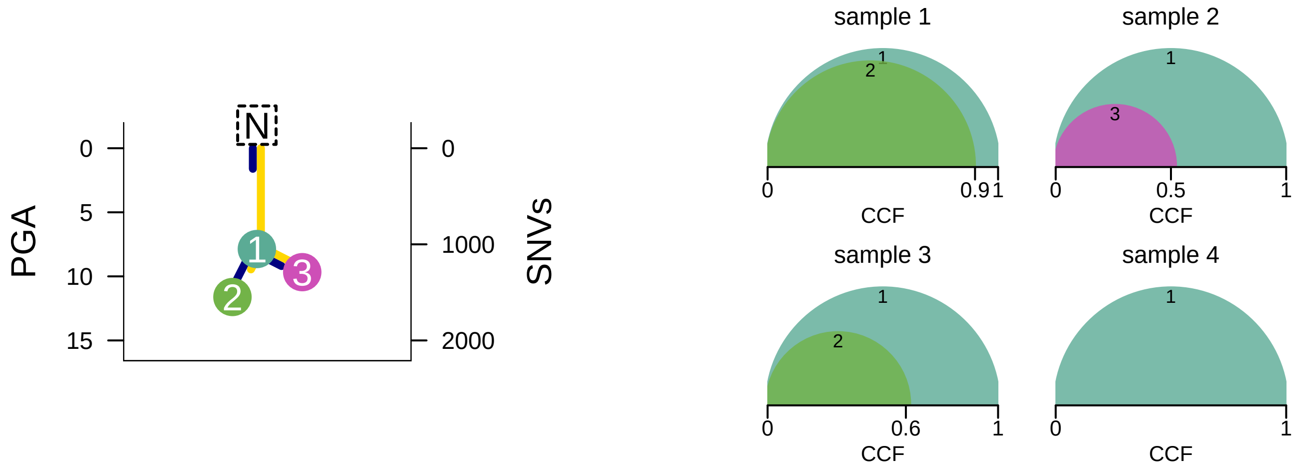

CPCG0334.MR MuTect−Battenberg

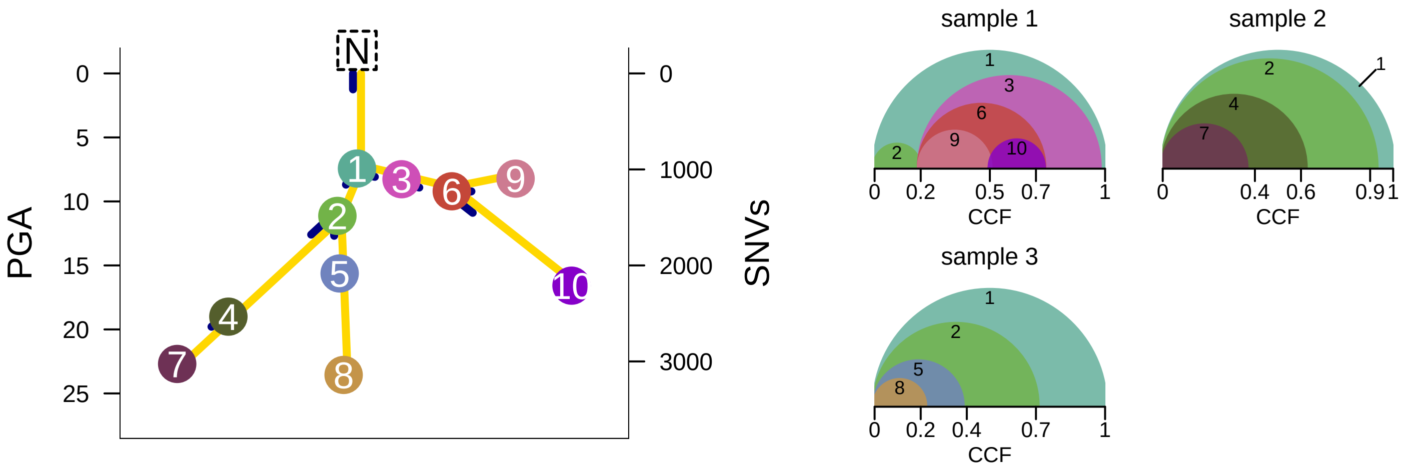

CPCG0334.MR MuTect−TITAN

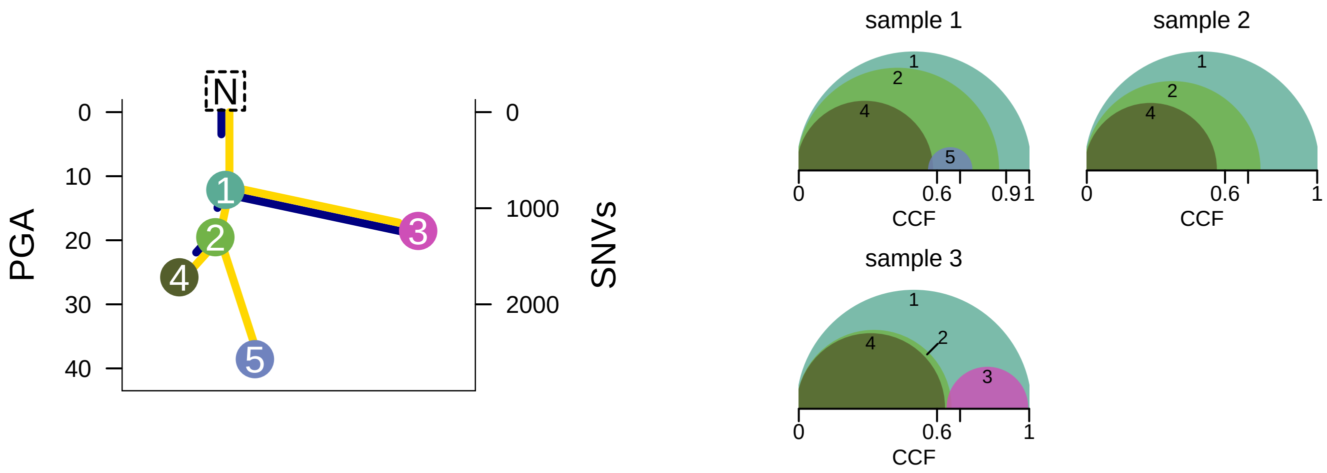

CPCG0334.MR MuTect−FACETS

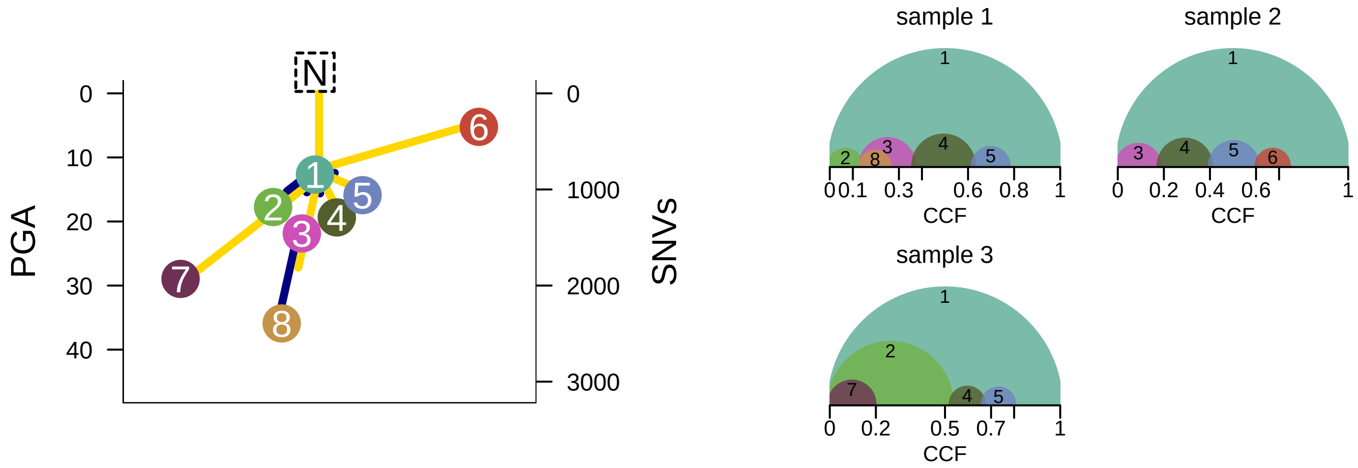

CPCG0334.MR SomaticSniper−Battenberg

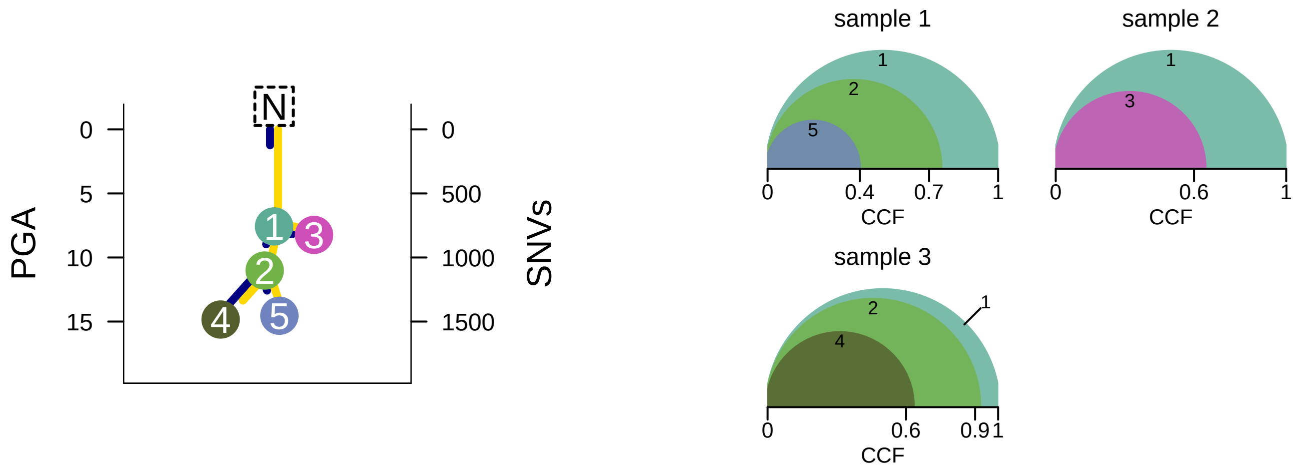

CPCG0334.MR SomaticSniper−TITAN

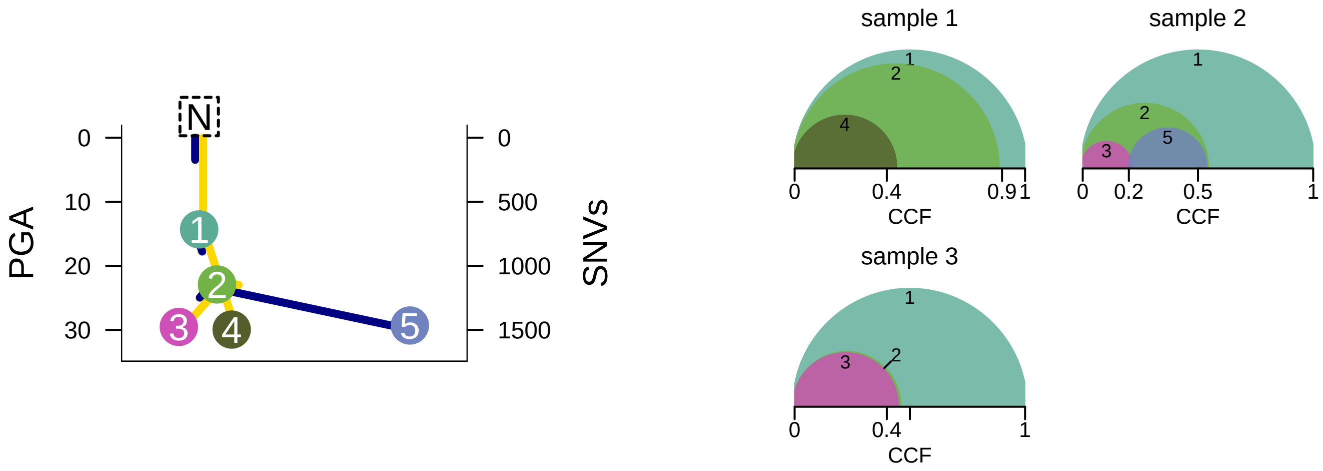

CPCG0334.MR SomaticSniper−FACETS

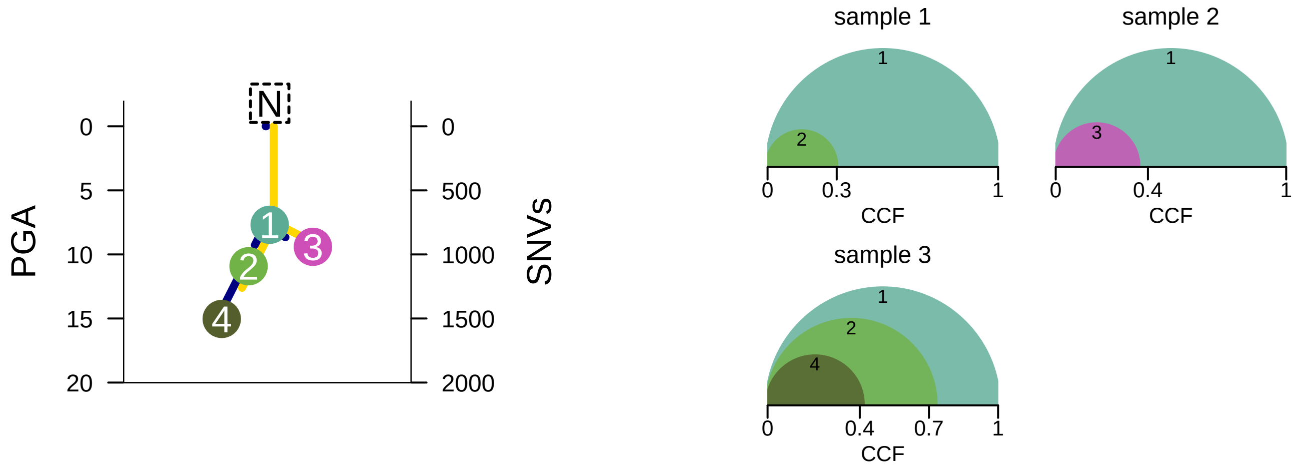

CPCG0361.MR MuTect−Battenberg

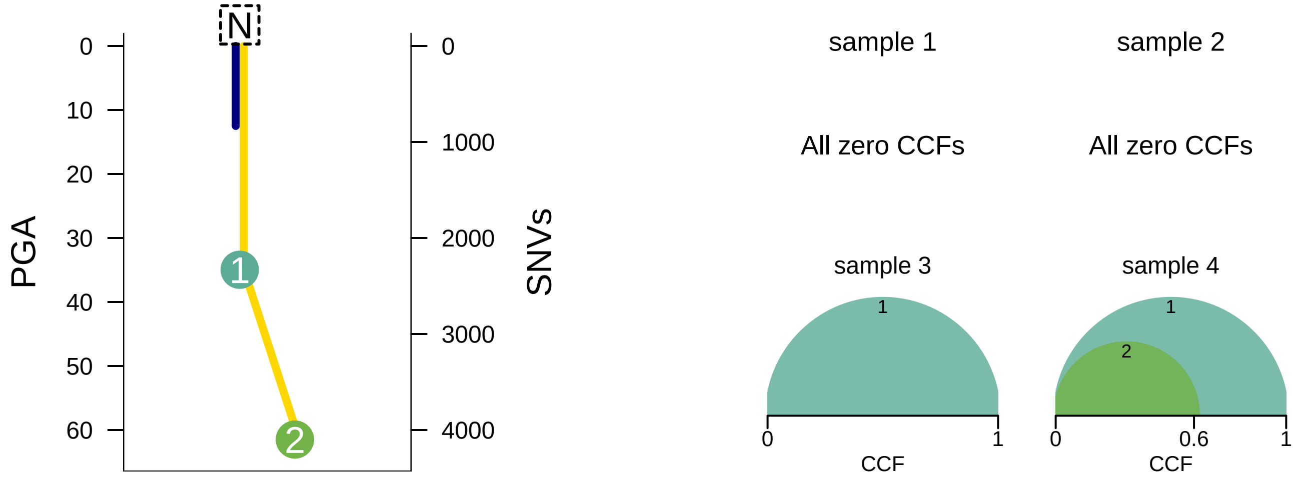

CPCG0361.MR MuTect−TITAN

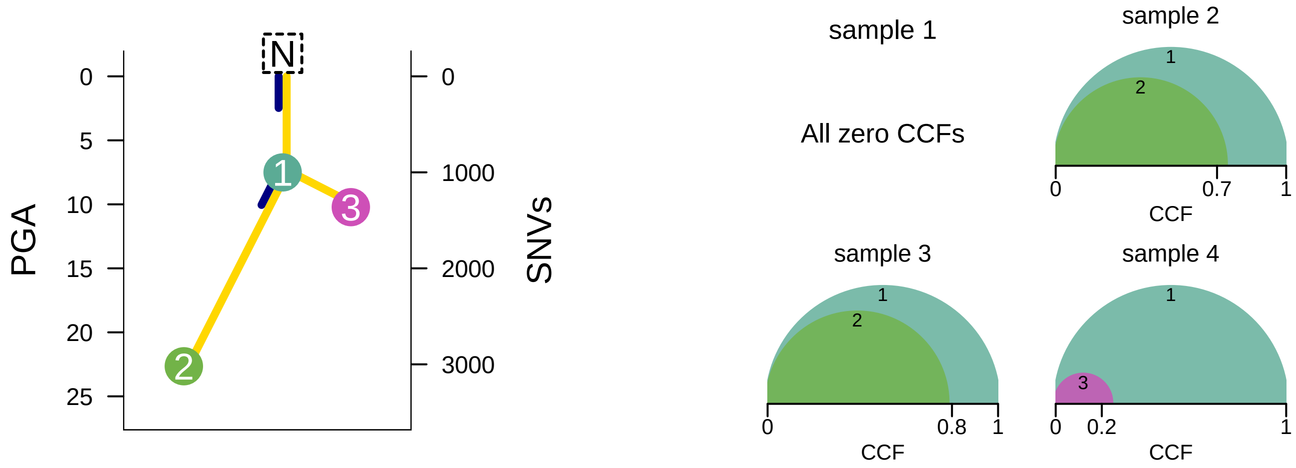

CPCG0361.MR SomaticSniper−Battenberg

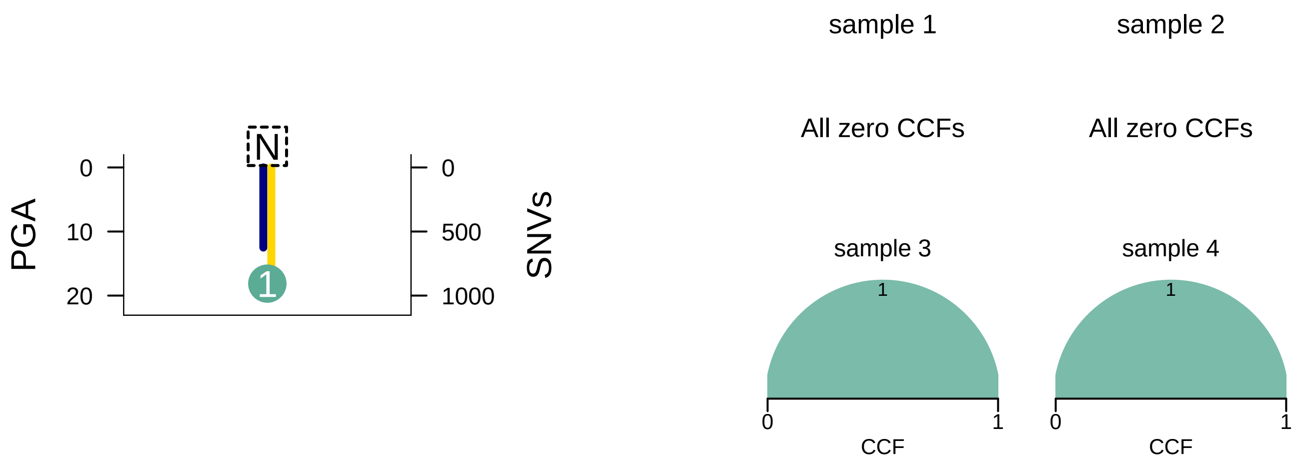

CPCG0361.MR SomaticSniper−TITAN

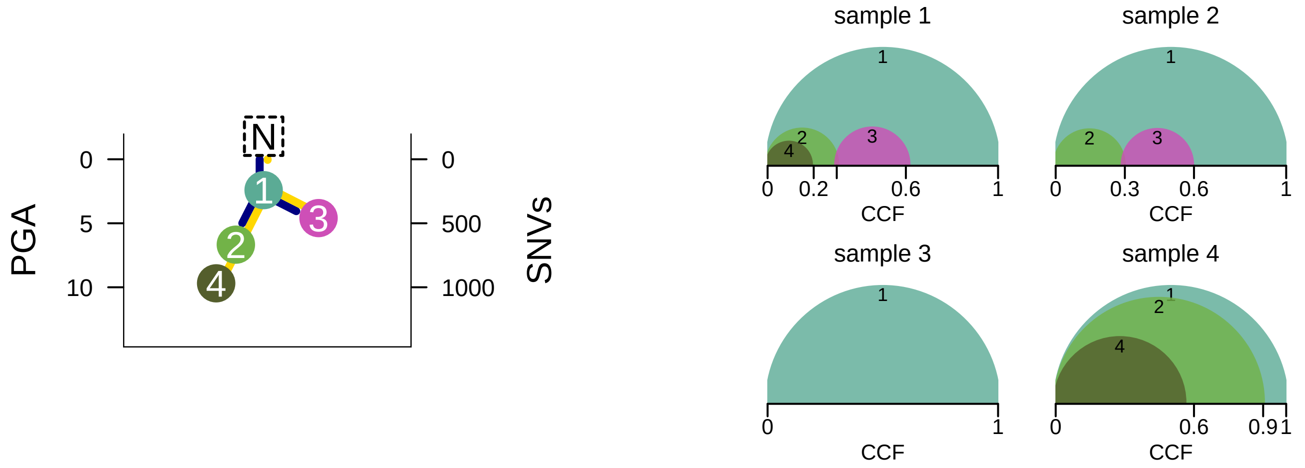

CPCG0364.MR MuTect−Battenberg

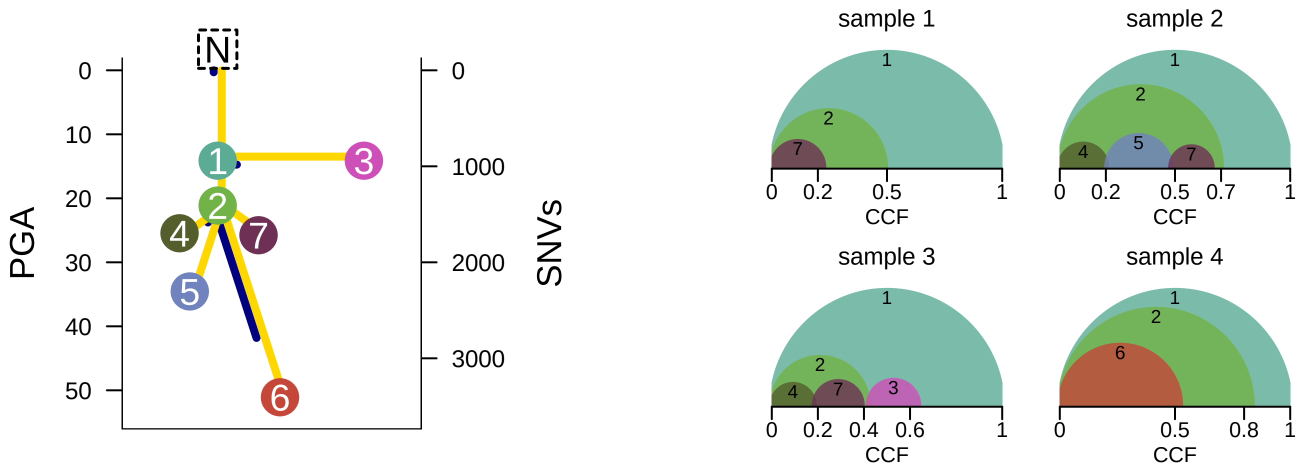

CPCG0364.MR MuTect−TITAN

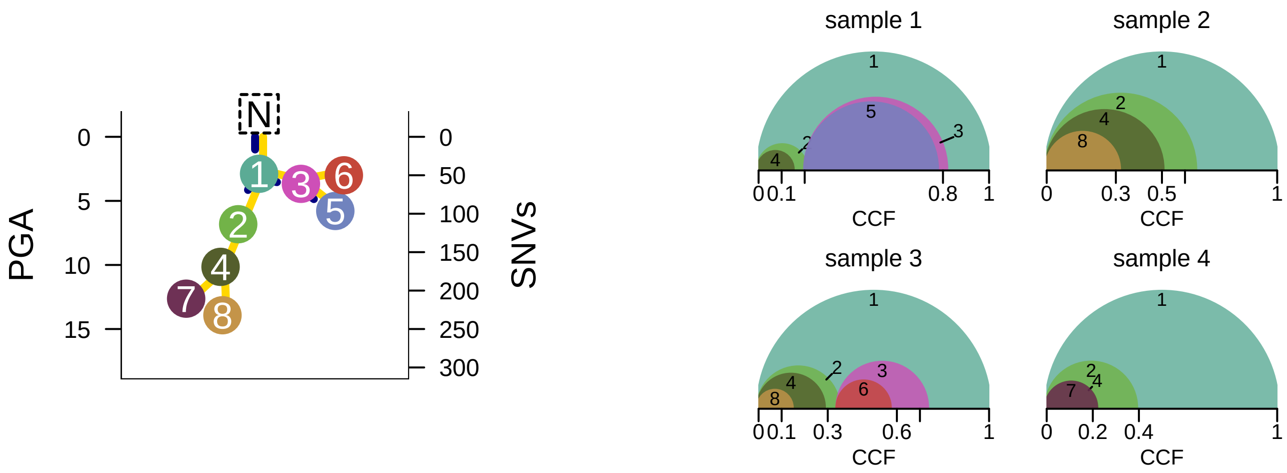

CPCG0364.MR MuTect−FACETS

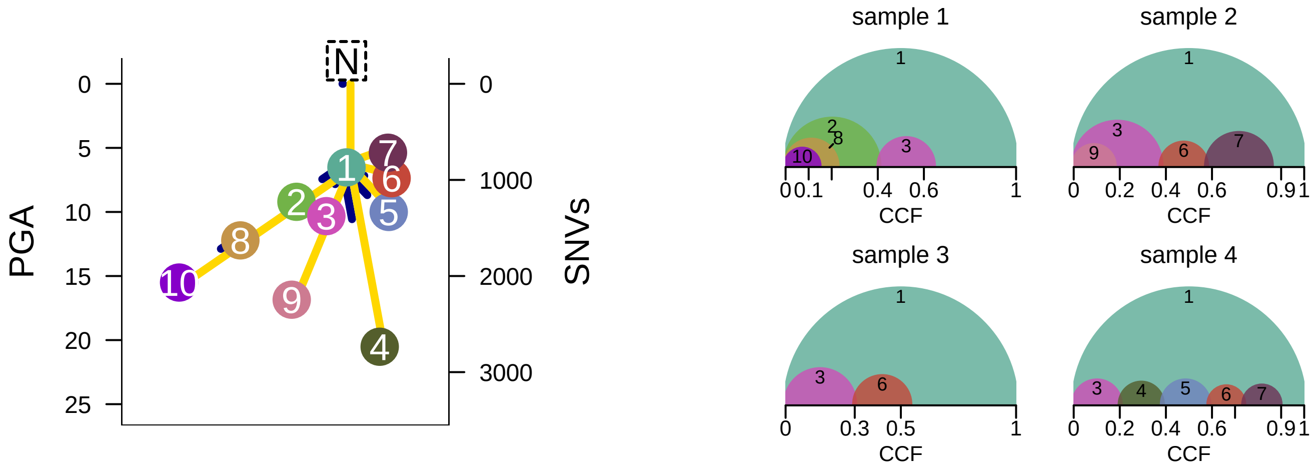

CPCG0364.MR SomaticSniper−Battenberg

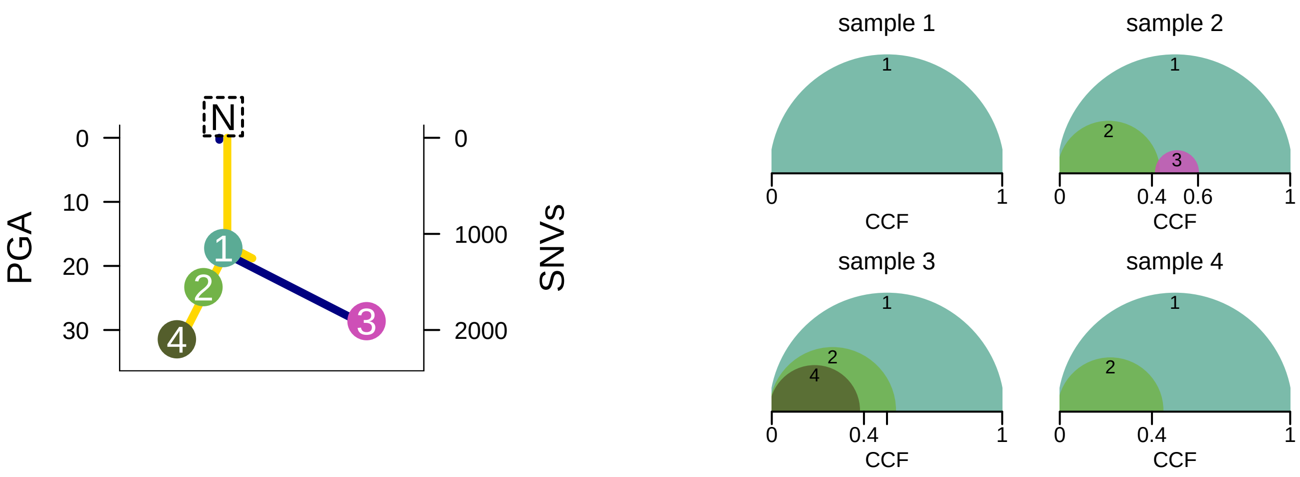

CPCG0364.MR SomaticSniper−TITAN

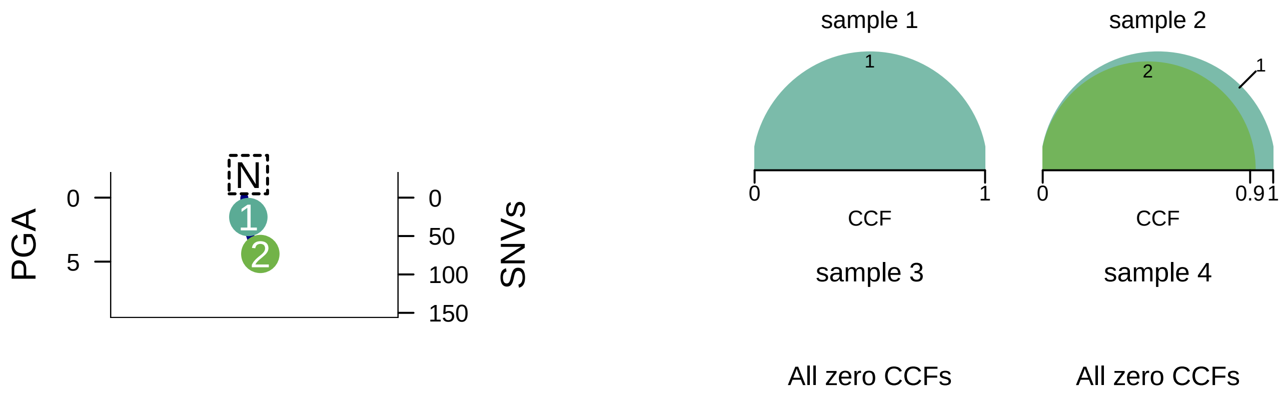

CPCG0364.MR SomaticSniper−FACETS

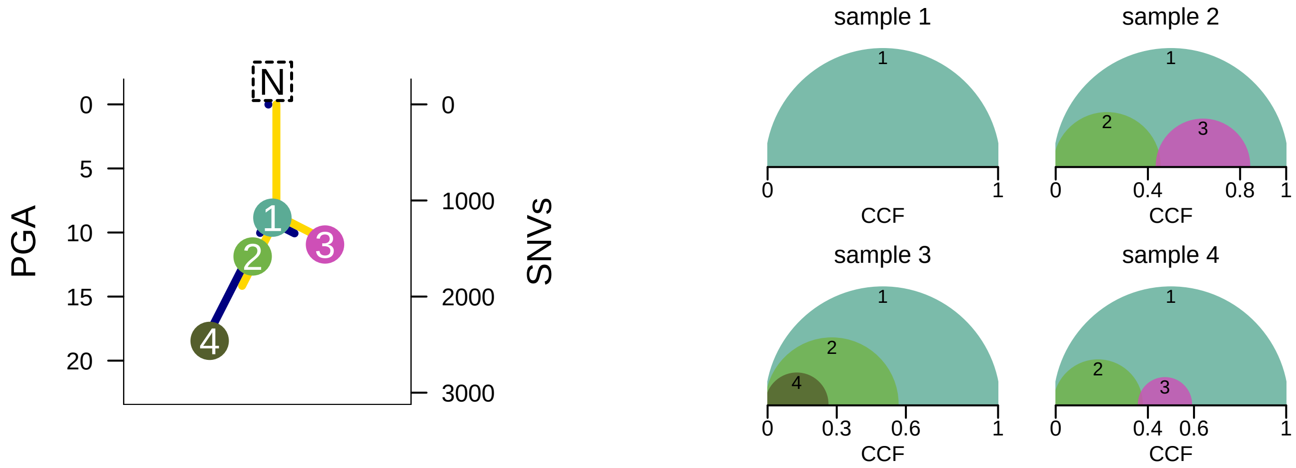

CPCG9001.MR MuTect−Battenberg

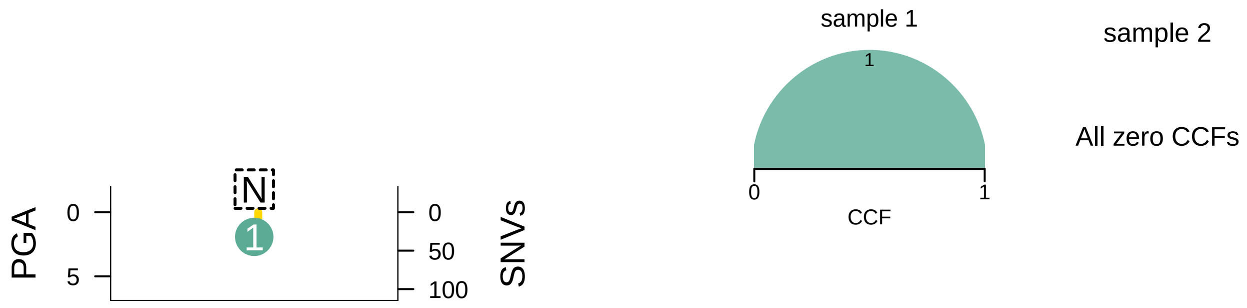

CPCG9001.MR MuTect−TITAN

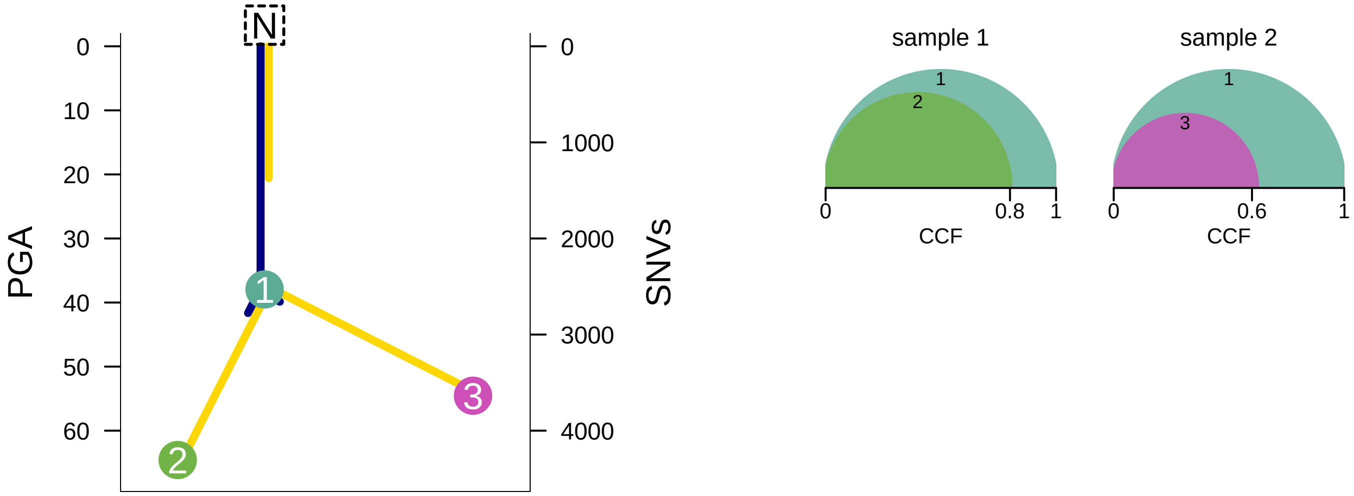

CPCG9001.MR MuTect−FACETS

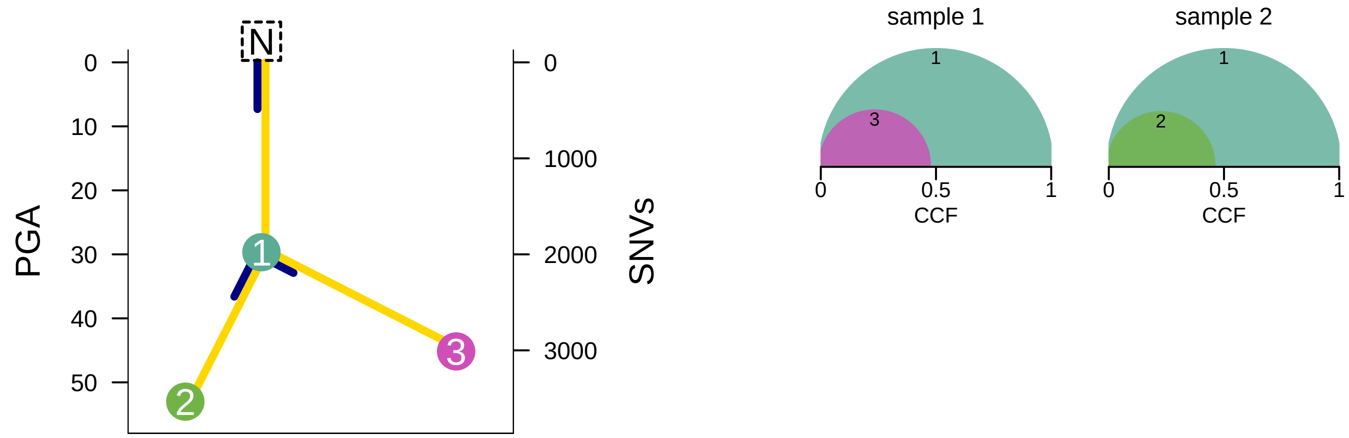

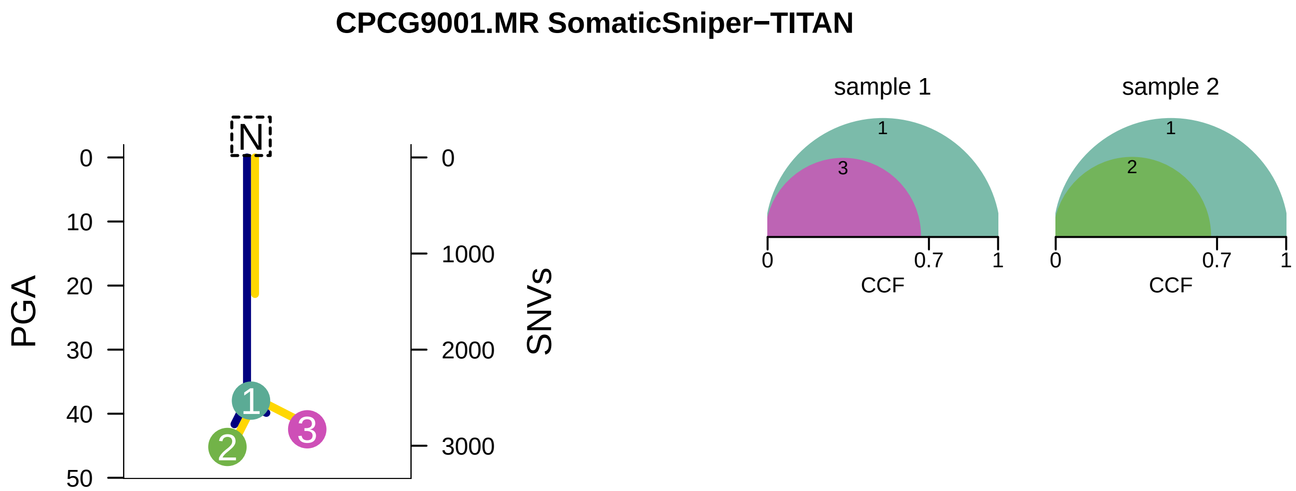
CPCG9001.MR SomaticSniper−TITAN
N
1
2
3
PGA
0
10
20
30
40
50
SNVs
0
1000
2000
3000
sample 1
1
3
0
0.7
1
CCF
sample 2
1
2
0
0.7
1
CCF

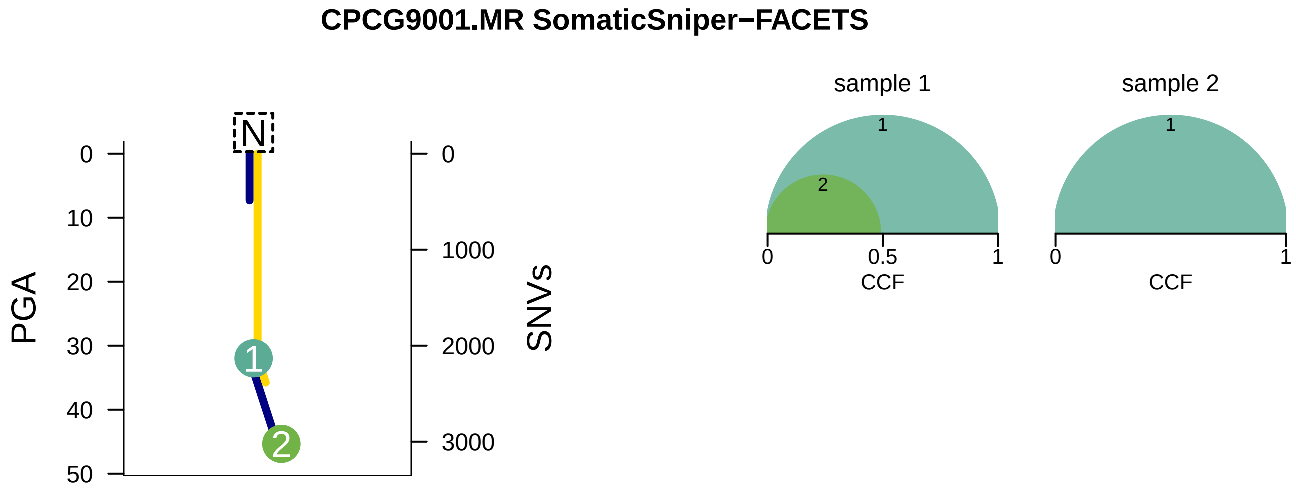
CPCG9001.MR SomaticSniper−FACETS
N
1
2
PGA
0
10
20
30
40
50
SNVs
0
1000
2000
3000
sample 1
1
2
0
0.5
1
CCF
sample 2
1
0
1
CCF

CPCG9002.MR MuTect−Battenberg

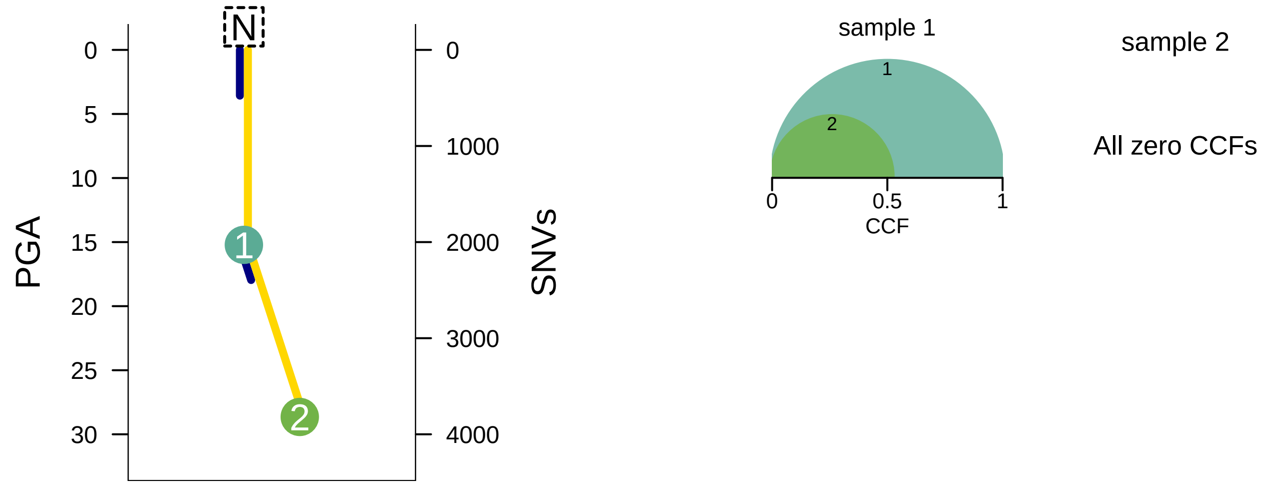

CPCG9002.MR MuTect−TITAN

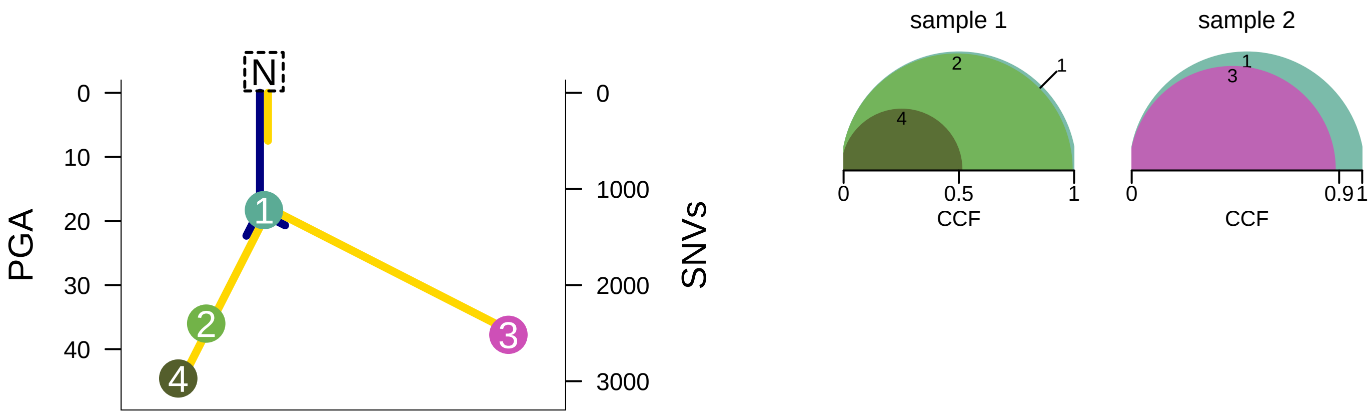

CPCG9002.MR MuTect−FACETS

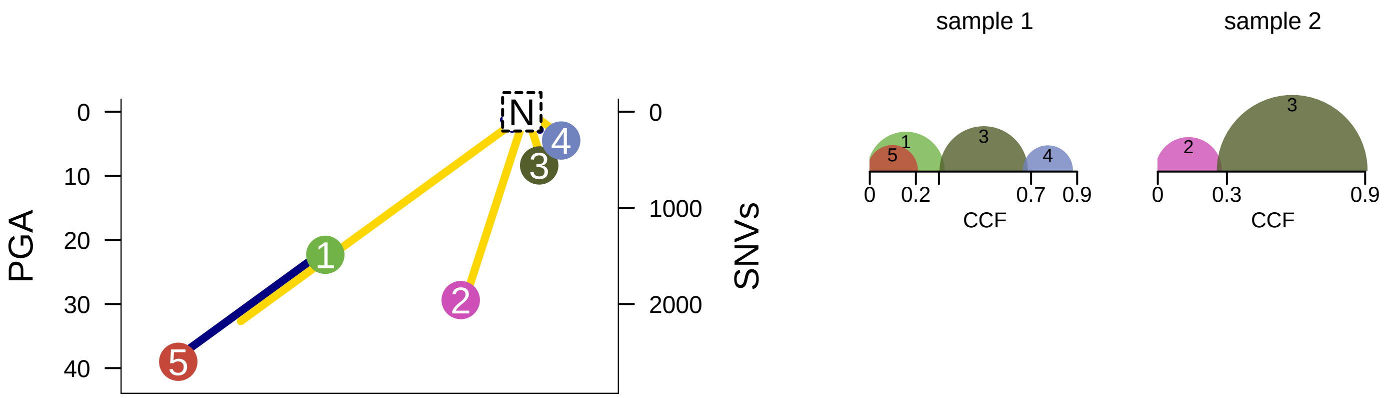

CPCG9002.MR SomaticSniper−Battenberg

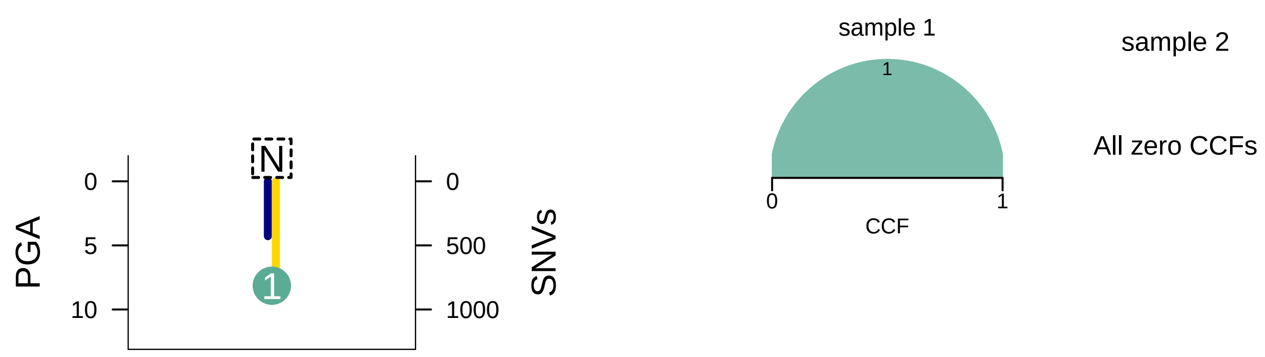

CPCG9002.MR SomaticSniper−TITAN

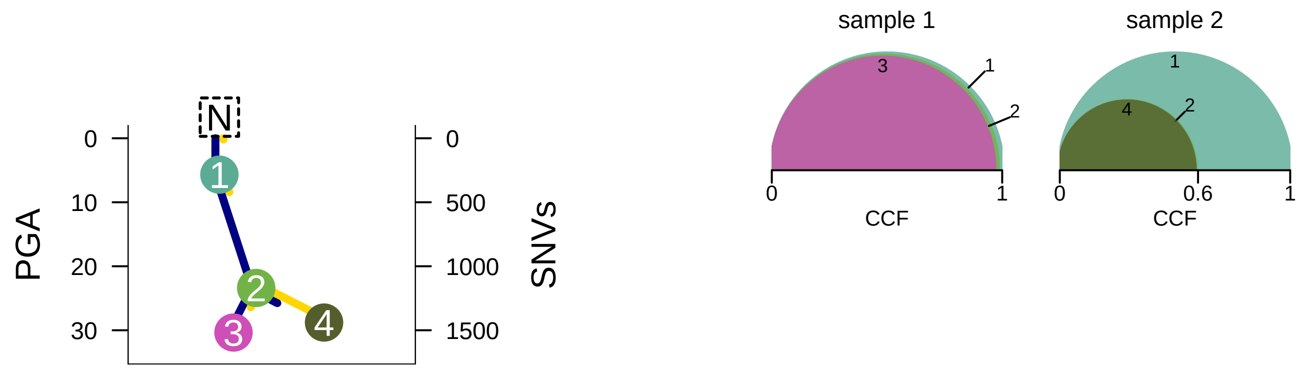

CPCG9002.MR SomaticSniper−FACETS

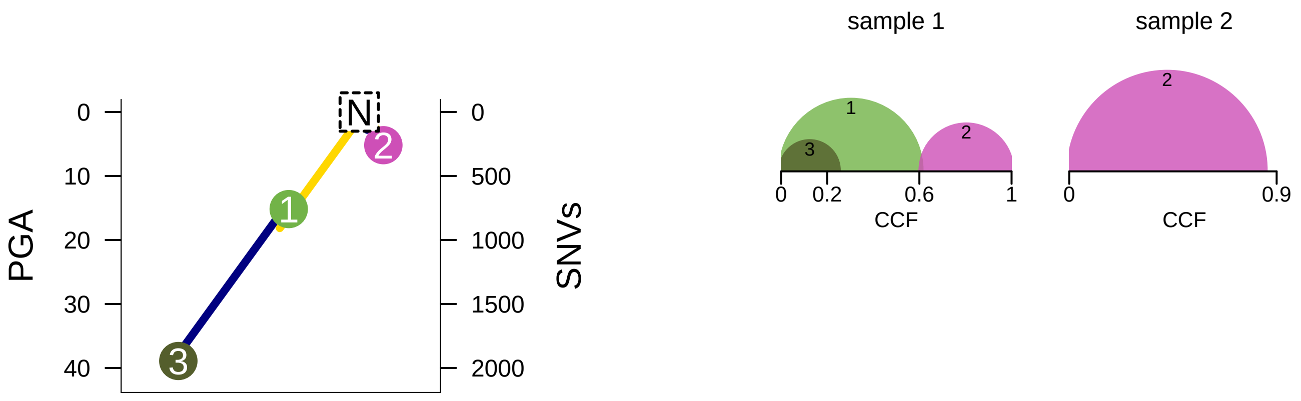

CPCG9003.MR MuTect−Battenberg

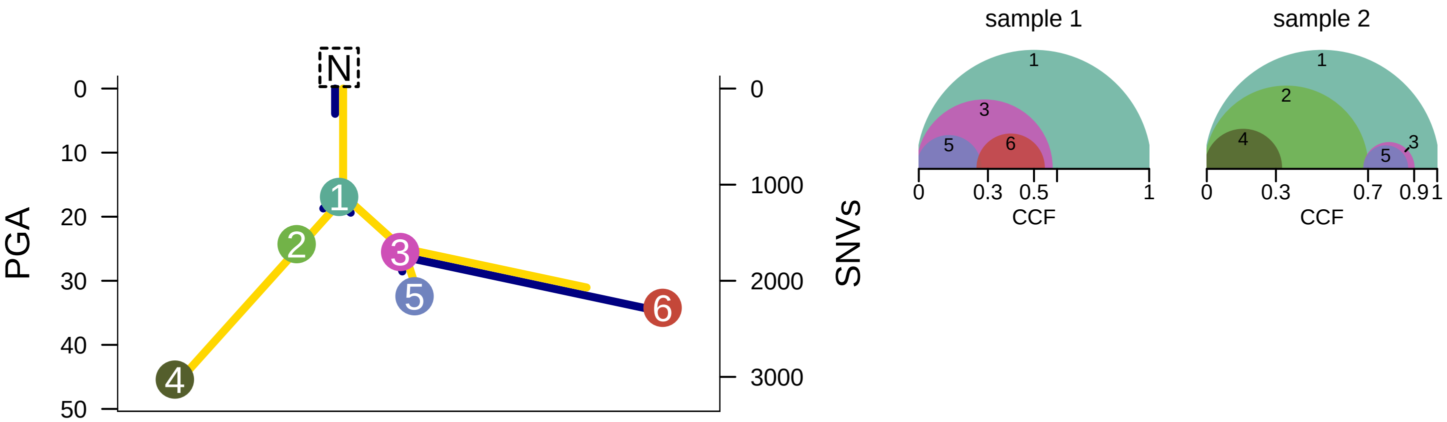

CPCG9003.MR MuTect−TITAN

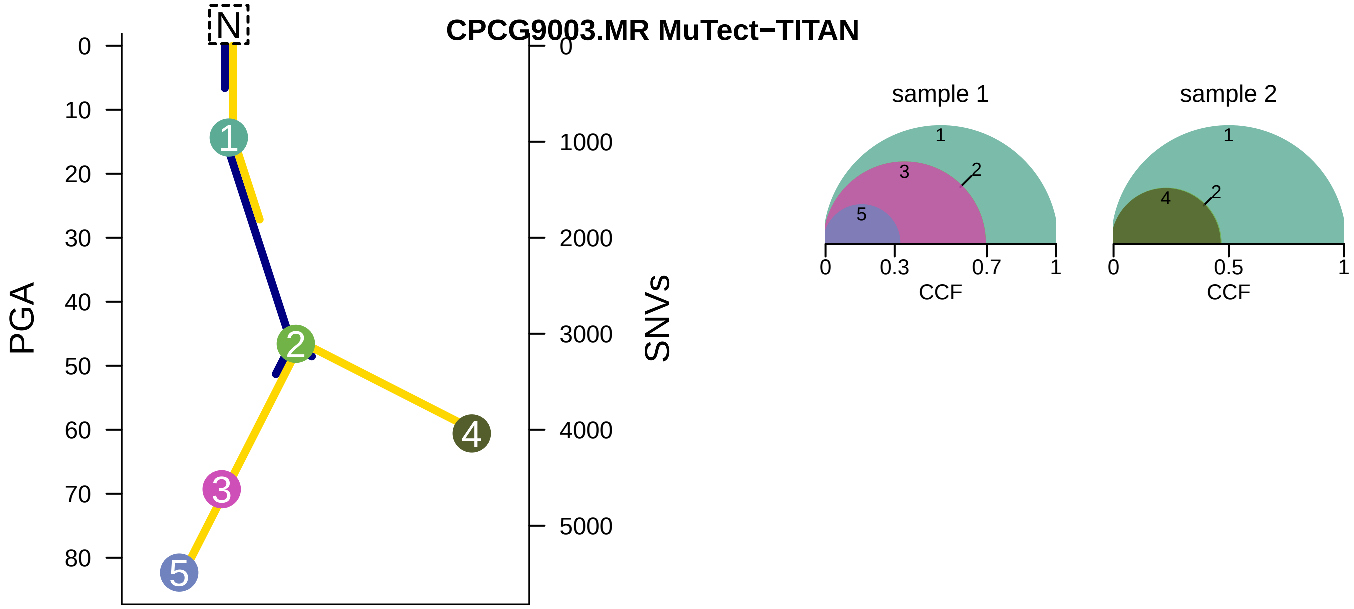

CPCG9003.MR MuTect−FACETS

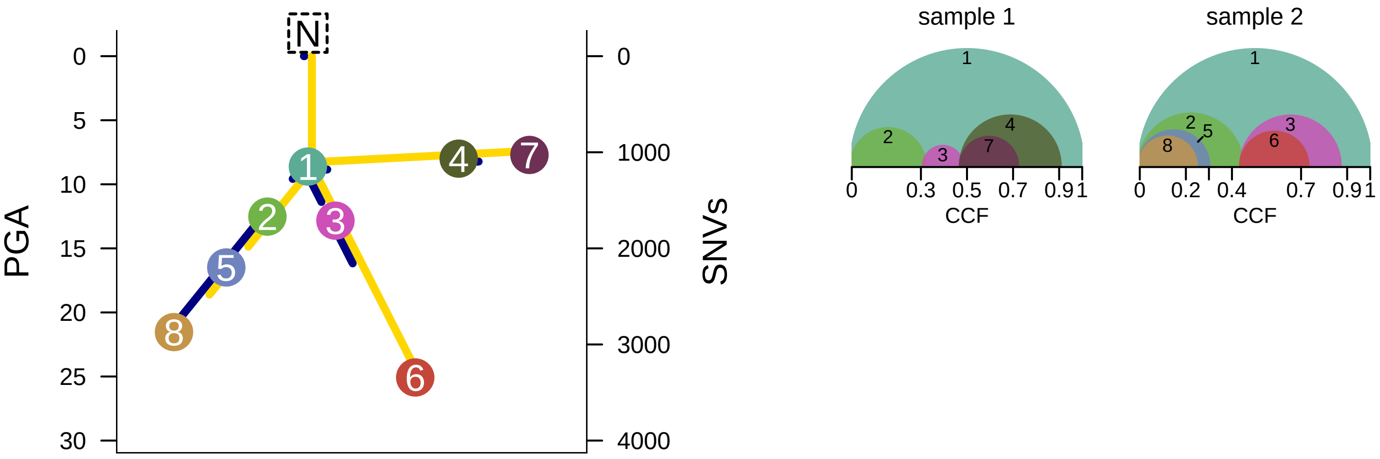

CPCG9003.MR SomaticSniper−Battenberg

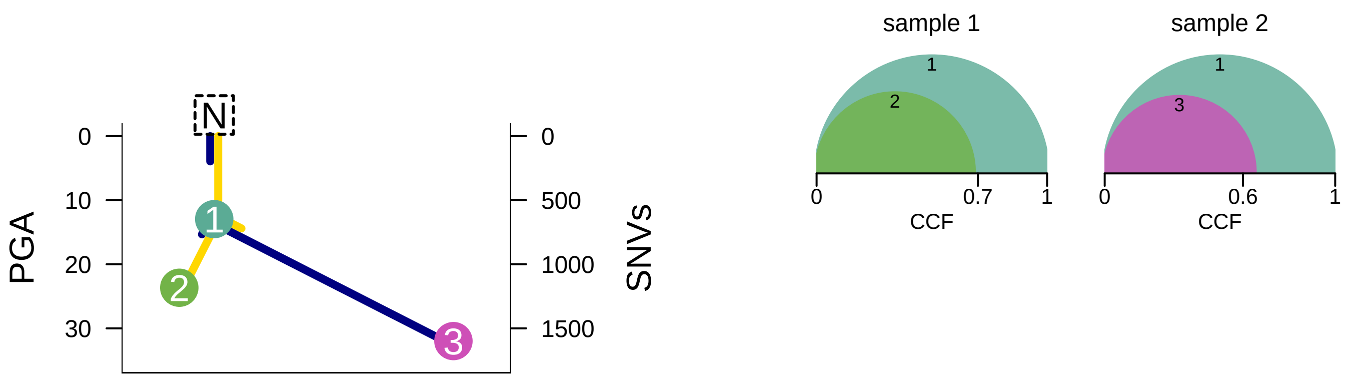

CPCG9003.MR SomaticSniper−TITAN

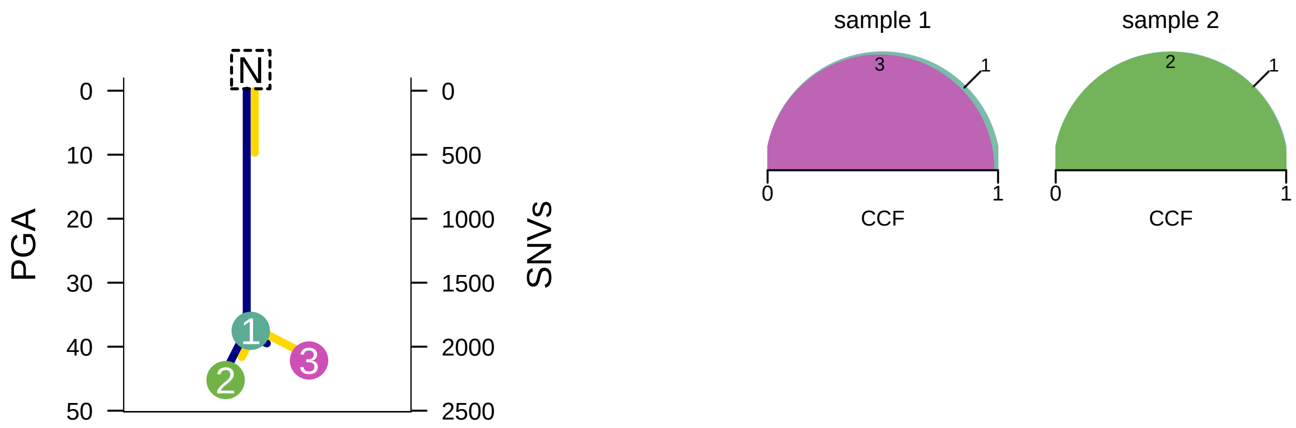

CPCG9003.MR SomaticSniper−FACETS

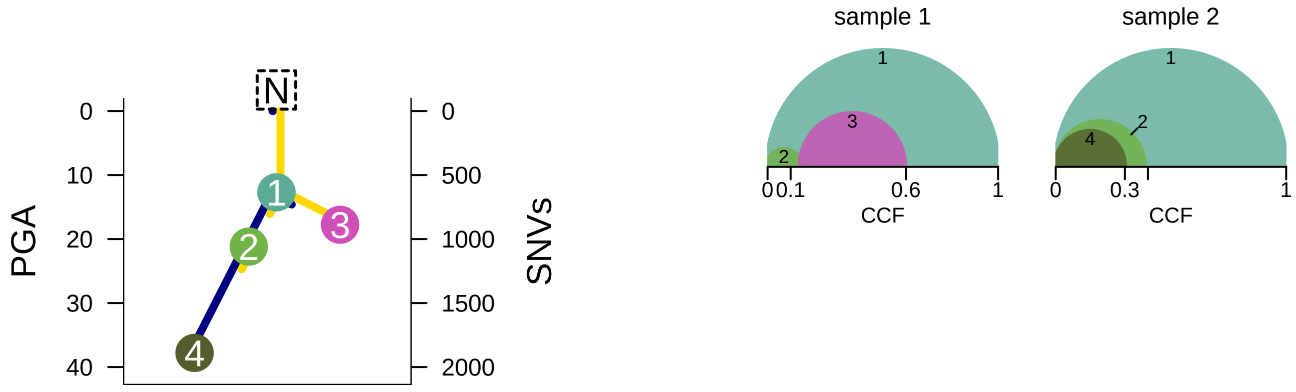

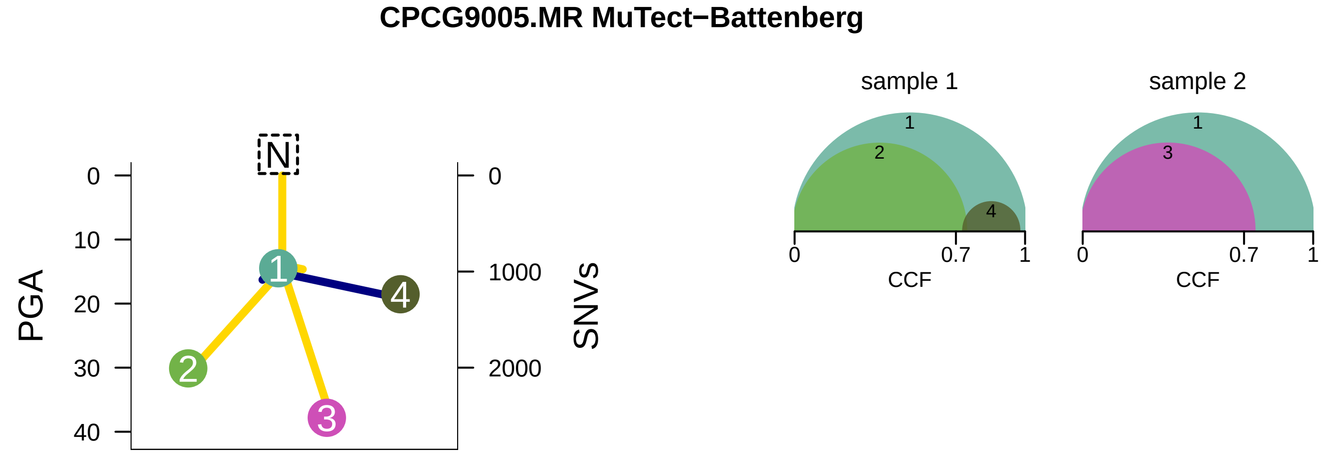
CPCG9005.MR MuTect−Battenberg
N
1
2
3
4
PGA
0
10
20
30
40
SNVs
0
1000
2000
sample 1
sample 2
CCF
0
0.7
1

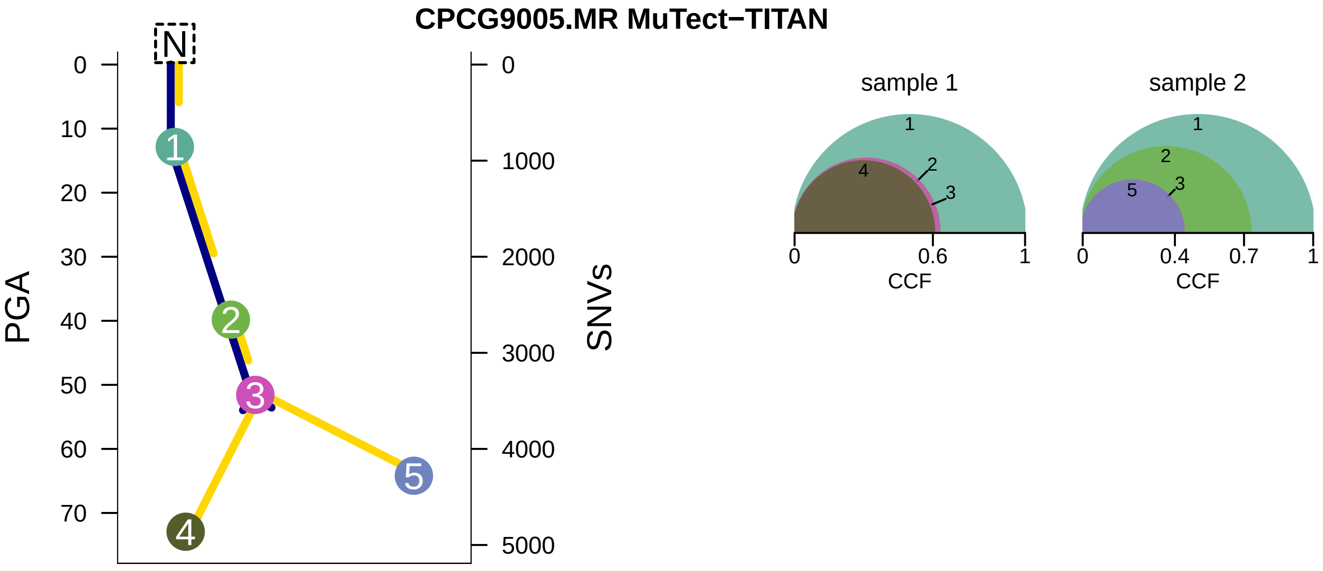
CPCG9005.MR MuTect−TITAN
N
1
2
3
4
5
PGA
0
10
20
30
40
50
60
70
SNVs
0
1000
2000
3000
4000
5000
sample 1
sample 2
CCF
0
0.6
0.4
0.7
1

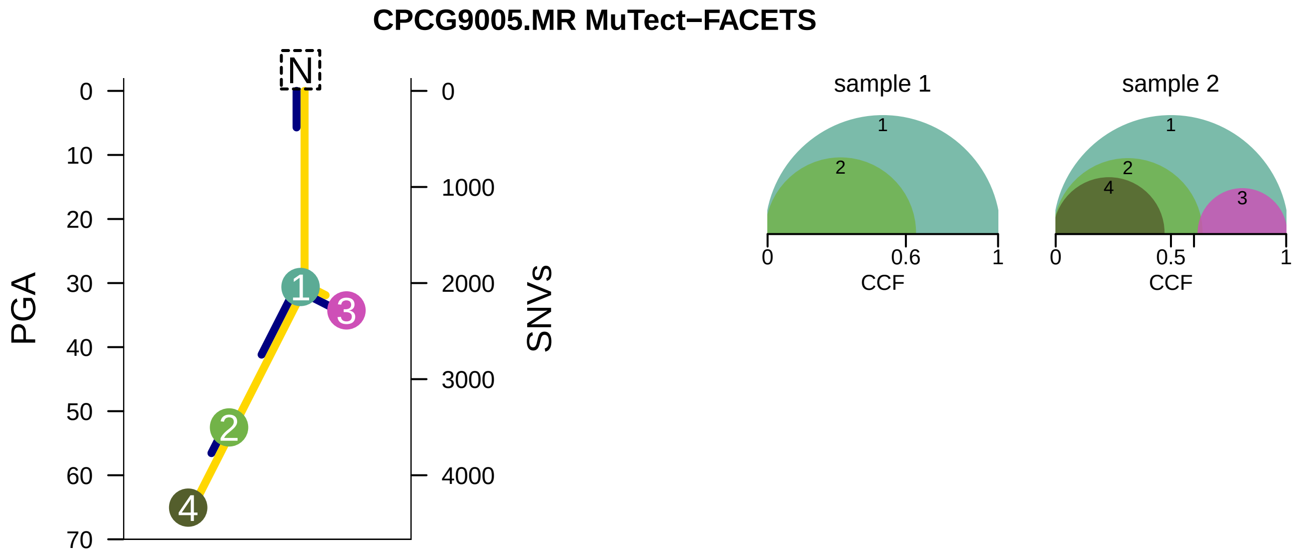
CPCG9005.MR MuTect−FACETS
N
1
2
3
4
PGA
0
10
20
30
40
50
60
70
SNVs
0
1000
2000
3000
4000
sample 1
sample 2
CCF
0
0.6
0.5
1

CPCG9005.MR SomaticSniper−Battenberg

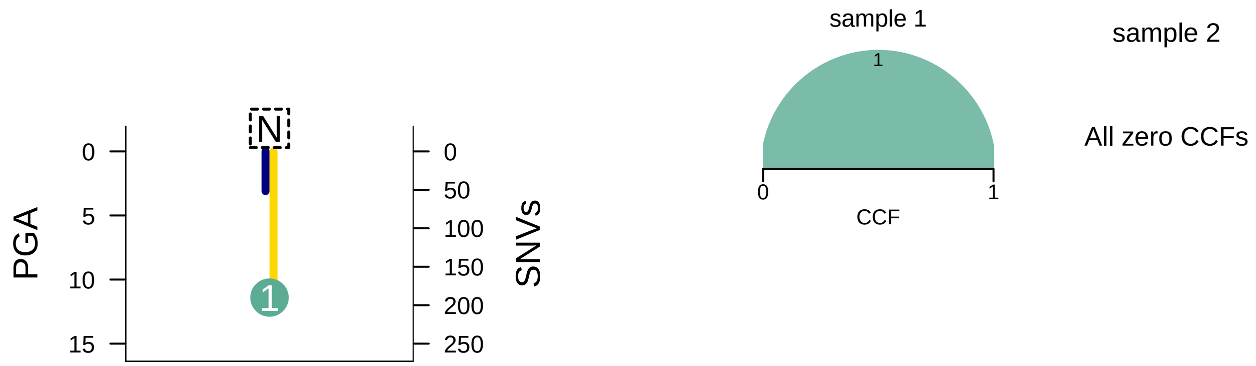

CPCG9005.MR SomaticSniper−TITAN

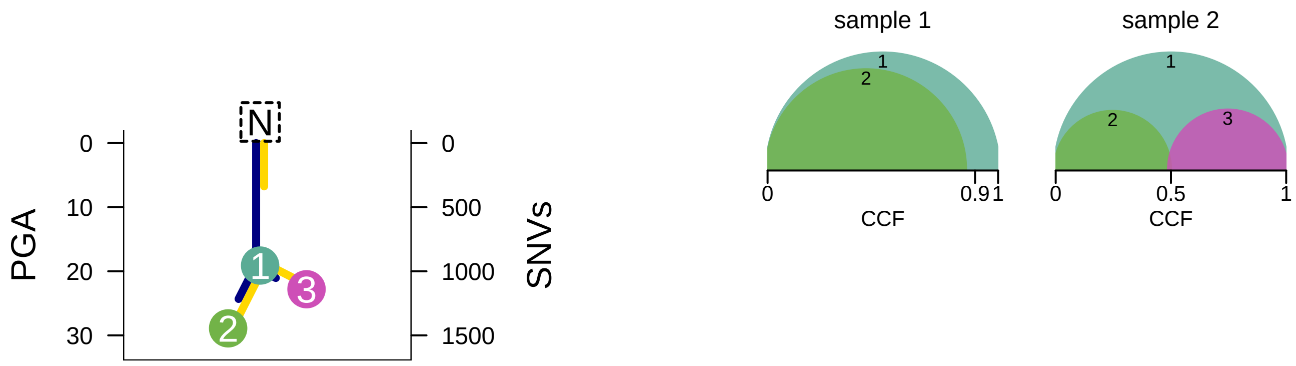

CPCG9005.MR SomaticSniper−FACETS

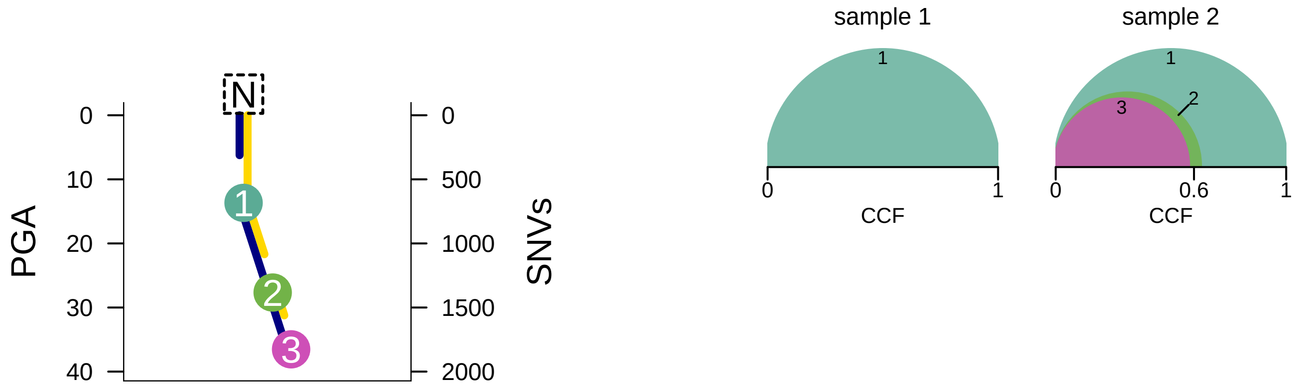

Supplement: Supplementary file 6 — Source Data [file 41467_2020_20055_MOESM6_ESM.zip › SRCHeterogeneity-SourceData/SamplePhyloWGSArchitectureReports.zip!/SamplePhyloWGSArchitectureReports/PhyloWGSTrees.pdf]
